# The interactome of intact mitochondria by cross-linking mass spectrometry provides evidence for coexisting respiratory supercomplexes*[Fn FN2]

**DOI:** 10.1074/mcp.RA117.000470

**Published:** 2017-12-08

**Authors:** Fan Liu, Philip Lössl, Beverley M. Rabbitts, Robert S. Balaban, Albert J. R. Heck

**Affiliations:** From the ‡Biomolecular Mass Spectrometry and Proteomics. Bijvoet Centre for Biomolecular Research and Utrecht Institute for Pharmaceutical Sciences, University of Utrecht, Padualaan 8, 3584 CH, Utrecht, The Netherlands;; §Netherlands Proteomics Center, Padualaan 8, 3584 CH, Utrecht, The Netherlands;; ¶Leibniz Institute of Molecular Pharmacology (FMP Berlin), Robert-Rössle-Straβe 10, 13125 Berlin, Germany;; ‖Laboratory of Cardiac Energetics, Systems Biology Center, National Heart, Lung, and Blood Institute, National Institutes of Health, Bethesda, MD

## Abstract

Mitochondria exert an immense amount of cytophysiological functions, but the structural basis of most of these processes is still poorly understood. Here we use cross-linking mass spectrometry to probe the organization of proteins in native mouse heart mitochondria. Our approach provides the largest survey of mitochondrial protein interactions reported so far. In total, we identify 3,322 unique residue-to-residue contacts involving half of the mitochondrial proteome detected by bottom-up proteomics. The obtained mitochondrial protein interactome gives insights in the architecture and submitochondrial localization of defined protein assemblies, and reveals the mitochondrial localization of four proteins not yet included in the MitoCarta database. As one of the highlights, we show that the oxidative phosphorylation complexes I-V exist in close spatial proximity, providing direct evidence for supercomplex assembly in intact mitochondria. The specificity of these contacts is demonstrated by comparative analysis of mitochondria after high salt treatment, which disrupts the native supercomplexes and substantially changes the mitochondrial interactome.

The mitochondrion is a complex, dynamic organelle, the characterization of which is fundamental to understand human disease and pharmacology (1–3). Genomics, transcriptomics, proteomics, and metabolomics have provided comprehensive inventories of the various mitochondrial biomolecules (1, 2, 4–6). While these technologies have enabled large-scale analyses of mitochondrial protein functions (7) and the identification of direct interaction partners of selected proteins (8), they are limited in their ability to capture protein complex structures and higher-order protein interaction networks. Monitoring these organizational aspects, however, is essential to improve our understanding of cellular processes since many of them are governed by protein nanomachineries or extended protein interaction networks (9, 10). Mitochondria host a wide array of such supramolecular systems, exemplified by the electron transport chain (ETC)^1^, complex V (CV, also called the F1F0 ATPase), and metabolic multienzyme complexes (metabolons), which were early on identified and monitored by conventional biochemical approaches (11–15). Our understanding of several mitochondrial protein machineries has increased in recent years, mostly due to advancements in the field of cryo electron microscopy (cryoEM) (16–20). However, to enable these high-resolution structural studies, the mitochondrial protein complexes of interest were typically extracted from their native environment, potentially resulting in the disruption or alteration of native protein contacts and conformations. Especially assemblies of membrane proteins, making up about a third of the mitochondrial proteome (21), have mainly been investigated following detergent solubilization, which can introduce artifacts (22). Probing of protein complex architectures in a more native-like context may be achieved by cryo electron tomography (cryoET), but the densely packed mitochondrial matrix so far hinders visualization of many structural details by cryoET (23). Therefore, cryoET studies have largely been conducted on isolated mitochondrial membranes and focused on a few abundant and distinctly shaped protein assemblies such as ETC complex I (CI) and CV (23–25).

Insights into the systemwide organization of proteins in intact mitochondria can potentially be obtained by cross-linking mass spectrometry (XL-MS). In XL-MS, native protein contacts are captured using a cross-linker, which is typically a small organic molecule composed of a spacer arm and two functional groups that are reactive toward specific residue side chains. After proteolytic digestion of the cross-linked sample, residue-to-residue cross-links can be localized by MS-based peptide sequencing. A cross-link can only occur if the distance between the residues can be bridged by the cross-linker. Detected cross-links, therefore, reveal maximum residue-to-residue distance constraints within and in between proteins, providing insights into protein conformations, protein complex architectures, and protein interaction networks (26, 27). Owing to recent technical and methodological advances, cross-links can nowadays be identified in highly complex samples, such as human cells and cell lysates (28–32). With regard to mitochondria, XL-MS thus holds the promise to close in upon the *in vivo* architecture of specific supramolecular protein assemblies, the overall interaction network of mitochondrial proteins, as well as their submitochondrial localizations. This potential has been illustrated in a recent study, using an in-house synthesized cross-linker and manually scripted MS methods (33). By combining cross-link identifications from 11 mitochondrial preparations from 4 different tissues, Schweppe *et al.* reported 1,920 unique residue-to-residue connections. However, the required use of a large amount of heterogeneous input material, from mitochondria with different proteomes (34), illustrates that proteome-wide XL-MS studies still suffer from limited sensitivity and depth. To address this issue, we recently presented the XlinkX v2.0 workflow, which substantially increases the analytical depth of proteome-wide XL-MS (30).

Here we apply this workflow to intact mouse heart mitochondria. Through 3,322 unique residue-to-residue connections, we assessed the sensitivity, specificity, structural validity, and subcompartmental integrity of the XL-MS approach, and annotated four proteins to be mitochondrial, that were not yet part of the MitoCarta 2.0 database (35). Furthermore, we performed in-depth structural interactomic analysis focusing on two main aspects: the supramolecular organization of oxidative phosphorylation (OXPHOS) complexes (*i.e.* CI-CV) and the condition-dependent profiles of these interactions. Our cross-links confirmed the interaction between individual complexes of the previously proposed CI-CIII-CIV supercomplex (17–19) but also showed that CII and CV reside close to CI, CIII, and CIV in the inner mitochondrial membrane, suggesting that all five complexes coexist in close proximity in intact heart mitochondria. To confirm the specificity of these interactions, we compared the native mitochondrial protein interaction network with that detected after high salt treatment. This comparison revealed a disruption of protein assemblies that were strongly based on electrostatic interactions, in particular the ETC supercomplexes and CV, and an extensive reorganization of the mitochondrial interactome. Detailed analysis of the specific interacting protein pairs and residue sites suggests multiple stoichiometries and/or conformations of disruption-sensitive respiratory supercomplexes in intact mitochondria.

## EXPERIMENTAL PROCEDURES

### 

#### 

##### Mitochondrial Isolation

The mitochondrial isolation buffer, called HEENK (10 mm HEPES, 1 mm EDTA, 1 mm EGTA, 10 mm NaCl, 150 mm KCl, pH 7.1, ∼300 mOsm/l), was carefully formulated to preserve native mitochondrial structures. Sucrose, which can alter cristae structure (36) and respiration (37), and Tris, which can interfere with amine-reactive cross-linkers, were avoided. In KCl isolation buffer pH 7.1, the matrix pH of resting mitochondria is around 7.8 (38). The isolation buffer contained the experimentally determined matrix concentration of potassium (39), sodium concentration in the experimentally determined range for healthy mitochondria (40) and osmolarity in the range of that measured for cellular cytoplasm or mammalian blood serum (41). Calcium can cause pore formation in the inner mitochondrial membrane (IMM) and increase reactive oxygen species generation in mitochondria (42), and thus including chelators was essential; EGTA and EDTA have been demonstrated to permit intact mitochondrial structure (43). HEENK was used for all steps from organ perfusion until cross-linker quenching.

Standard differential centrifugation methods were used to prepare mouse heart mitochondria, with changes made to the isolation buffer described above (44). We used C57BL/6N male mice (Taconic Farms #B6-M; 7–9 weeks old). They were anesthetized with 3% isoflurane prior to surgery. A laparotomy was performed, ∼300U heparin sulfate injected into the inferior vena cava, the diaphragm cut, and the heart punctured. The heart was perfused with 1 m KCl, followed by addition of HEENK. The heart was removed from the animal, and all subsequent steps were conducted on ice in HEENK. The heart was weighed (usually 0.14–0.18 g), chopped with a scissors in 3 ml HEENK containing 1 mm phenylmethanesulfonyl fluoride (from 0.1 m stock in isopropanol) and 1:100 dilution of protease inhibitor mixture in DMSO (Sigma). The heart pieces were ground with a VirTis VirtiShear (two rounds of 5 s at 40% of max speed on ice), and lysed in a 5 ml Dounce homogenizer (10 passes of a tight-fitting glass piston). The homogenate was spun slowly in microcentrifuge tubes in a tabletop microcentrifuge (600 × *g* for 5 min at 4 °C), the cell pellet set aside, and the mitochondrial supernatant transferred to clean microcentrifuge tubes. The mitochondrial supernatant was further cleared with a second slow spin and those pellets set aside with the cell pellets from before. The cleared mitochondrial supernatant was spun fast (9,000 × *g* for 5 min at 4 °C), the supernatant discarded, and the mitochondrial pellet was saved. The cell pellets were resuspended in 3 ml HEENK with protease inhibitors and subjected to a total of three rounds of homogenizing and slow-fast spins; only the first round received two slow spins, and the final round was done in HEENK without protease inhibitors. The mitochondrial pellets were combined into 0.5 ml HEENK, resuspended using a 1 ml Dounce homogenizer, and the protein concentration determined (using Bradford dye, Usb Corporation, and a Shimadzu UV-2700 spectrophotometer). The yield of mitochondria was typically ∼1 mg protein per mouse heart. The complex IV content of the mitochondria was typically 1.4–1.7 nmol cytochrome per mg protein, as determined by optical absorption of the difference spectrum (reduced using Na hydrosulfite, minus oxidized in aerated buffer) at 605 nm. An aliquot corresponding to 1 mg mitochondrial protein was transferred to a microcentrifuge tube and spun fast. The mitochondrial pellet was then used in cross-linking or other studies. When the mitochondrial pellet produced was inspected by electron microscopy, it showed good purity and submitochondrial structural integrity (Supplemental Fig. S1).

##### Supercomplex Disruption

Where indicated, the mitochondrial pellets were disrupted as follows. First, pellets were frozen at −80 °C and thawed on ice then resuspended to 1.5 mg/ml in disruption buffer (10 mm HEPES, 2 mm EDTA, 5 m NaCl, pH 7.75). These were incubated for 1 h at 30 °C with mild shaking at 20 rpm in a tabletop incubator (Labnet Vortemps 56). The disruption was stopped by 4X dilution in HEENK and spun at 16,000 *g* for 10 min at 4 °C (in a Sorvall Biofuge Fresco). The disrupted mitochondrial pellet was resuspended to 0.5 mg/ml in HEENK and spun again to wash. The washed disrupted mitochondrial pellet was then used in cross-linking or other studies.

##### Chemical Cross-Linking

The mitochondrial pellet was resuspended to 1.5 mg/ml in HEENK plus 0.5 mm disuccinimidyl sulfoxide (DSSO) (Thermo Fisher Scientific, resuspended freshly in anhydrous DMSO to 50 mm) and incubated at 20 °C for 1 h. The reaction was stopped by addition of 100 mm Tris, pH 8.0, and fast spun. The pellet was washed in 1 ml of HEENK plus 100 mm Tris, pH 8.0, incubated at 20 °C for 10 min, and fast spun. At this point, less than 5 h had passed since the animal was sacrificed. The cross-linked mitochondrial pellet was stored at −80 °C.

##### Mass Spectrometry

The cross-linked mitochondrial pellet was solubilized in 8 m urea, 50 mm ammonium bicarbonate. Subsequently, the extracted proteins were prepared for and analyzed by liquid chromatography-mass spectrometry (LC-MS) in the same way as described previously (30, 31). Briefly, the cross-linked proteins were reduced with 4 mm DTT at 56 °C, alkylated with 8 mm iodoacteamide and, sequentially digested with Lys-C (4 h, 37 °C, 1:75 (w/w) enzyme:substrate ratio) and trypsin (overnight, 37 °C, 1:100 (w/w) enzyme:substrate ratio). The resulting peptide mixture was desalted using Sep-Pak C18 cartridges (Waters), dried under vacuum, reconstituted in 10% (v/v) formic acid, and fractionated by strong cation exchange (SCX) chromatography, essentially as described in (31). Briefly, 20 later strong cation exchange fractions, containing predominantly higher charged peptides (*z* ≥ 3) were analyzed by LC-MS using an ultra-HPLC Agilent 1200 system (Agilent Technologies), equipped with an in-house packed C18 column for reversed phase separation (column material: Poroshell 120 EC-C18, 2.7 μm (Agilent Technologies)) and coupled online to an Orbitrap Fusion mass spectrometer (Thermo Fisher Scientific). Mass analysis was performed using a previously described CID-MS2-MS3-ETD-MS2 aquisition method (30), which can be readily set up on any commercially available, ETD-enabled Orbitrap Fusion and Fusion Lumos mass spectrometers. MS1 and MS2 spectra were acquired in the Orbitrap mass analyzer (mass resolution at *m/z* 200 = 60,000 and 30,000, respectively) and MS3 spectra were acquired in the ion trap mass analyzer. Notably, MS3 acquisitions were only triggered when peak doublets with a specific mass difference (Δ = 31.9721 Da) were detected in the CID-MS2 spectra, as this is indicative for the presence of DSSO cross-linked peptides (30).

For bottom-up proteomics analysis, non-cross-linked mitochondrial proteins were extracted and digested as described above. Using the same LC-MS setup as described above, single-shot LC-MS/MS experiments were performed for peptide identification without further prefractionation.

##### Analysis of Cross-Linking Data

For structural analysis of the data, cross-links were mapped on publicly available (pseudo-)atomic structural models of murine proteins or their mammalian homologs (PDB codes indicated in the respective figures) using Pymol (Schrodinger LLC). If non-murine proteins were used for structural mapping, they were first compared with the murine proteins by pairwise sequence alignment using NCBI BLAST (45). All non-murine proteins showed a sequence homology of more than 75%, strongly indicating that their structures are highly similar to the murine proteins. Correspondingly, the cross-linked Lys residues readily aligned to identical or homologous residues in the non-murine structures.

Structural analysis of the CI-CIII-CIV supercomplex was performed using the tight CI-CIII_2_-CIV supercomplex (PDB code 5J7Z) and a complete CI structure (PDB code 5LNK), which were structurally aligned in Pymol to generate a more complete CI-CIII_2_-CIV supercomplex model. This model was also used to calculate alternative interaction spaces with the DisVis web server (46), assuming a maximum cross-linked Cα–Cα distance of 30 Å.

Interaction networks were generated using Cytoscape v3.4 (47) and xiNET (48).

##### Native Gel Electrophoresis

Blue native PAGE sample and gel running buffers and native unstained molecular weight marker were obtained commercially (Thermo Fisher Scientific). Mitochondria were resuspended in 1X native sample buffer at 10 mg/ml, digitonin (Sigma) added from a fresh 10% stock in anhydrous DMSO to a final sample concentration of 2% and mixed gently but thoroughly. Samples were incubated for 30 min on ice and spun at 13,000 *g* for 10 min at 4 °C. The supernatant was transferred to a tube with 1:10 volume of Coomassie sample additive and mixed thoroughly but gently.

A 3–12% bis-tris gradient native PAGE gel (Thermo Fisher Scientific) was prepared by flushing the wells with cathode buffer, the samples loaded (usually 10–50 μg of protein per well), and run at 4 °C for 475 Vh (not to exceed 250 V). For blue native PAGE, the final ∼45 min was run using cathode buffer that was diluted 1:4 in anode buffer to destain the gel. In cases where the gel was further stained, it was incubated overnight with 1 mg/ml Coomassie Brilliant Blue G-250 (Bio-Rad) in 7% acetic acid in 50% MeOH, then destained with three changes of 3% H_3_PO_4_ in 50% MeOH and rinsed in H_2_O before scanning.

##### Immunoblotting and Scanning

The gels for immunoblotting were incubated for 10–20 min at 20 °C in denaturing 1X transfer buffer (using Bio-Rad product 1704273, 1X buffer contained 20% MeOH). Proteins were transferred to 0.2 μm PVDF membrane (Bio-Rad) using the Bio-Rad Trans-blot Turbo apparatus set to 2.5 A or 25 V for 10 min, and the membrane was rinsed in dH_2_O. For blue native gels, the membrane was destained with three changes of 25% acetic acid in 50% MeOH and rinsed again with dH_2_O. The membrane was dried in air, reactivated with 100% MeOH for 10 s, rehydrated in dH_2_O for >10 min, and blocked in a hybrid iBind flex FD solution containing 0.5X buffer and 0.5X additive (using Thermo Fisher buffer kit SLF2019). Membranes were probed by capillary action in the Thermo Fisher iBind Flex device using the hybrid solution, with primary antibodies diluted 1:500 and secondary antibodies diluted 1:1,000 with added 0.0375% SDS. Antibodies used were mouse monoclonal IgG anti-CIII UQCRC1 (Abcam ab110252, 1 mg/ml) and AlexaFluor 488 goat anti-mouse IgG, highly cross-adsorbed, (Invitrogen A11029, 2 mg/ml). Probed membranes were washed in dH_2_O and dried before scanning. Gels (above) and membranes were scanned for Alexa 488 (excitation 488 nm laser, emission 520BP40 filter) and/or Coomassie G-250 (excitation 633 nm laser, emission 560LP filter), on a GE Healthcare Typhoon 9400 variable mode imager at a resolution of 50 μm and photomultiplier tube setting of 300–600.

##### Experimental Design and Statistical Rationale

We performed two biological replicates (mitochondria purified from two individual mice) for both the XL-MS experiments of native and salt-treated mitochondria. Peak list (.mgf files) is generated in Proteome Discoverer (version 1.4) to convert each RAW file into three MGF files, respectively containing the CID-MS2, ETD-MS2, and CID-MS3 data. During MGF conversion, the CID- and ETD-MS2 spectra were additionally deconvoluted to charge state 1 using the MS2 Spectrum Processor add-on node in Proteome discoverer v1.4. The MGF files were used as input to identify cross-linked peptides with standalone XlinkX v2.0 (30), which was operated using the following setting: MS ion mass tolerance: 10 ppm; MS2 ion mass tolerance: 20 ppm; MS3 ion mass tolerance, 0.6 Da; fixed modification: Cys carbamidomethylation; variable modification: Met oxidation; enzymatic digestion: trypsin; allowed number of missed cleavages: 3. All MS2 and MS3 spectra were searched against concatenated target-decoy databases generated based on the MitoCarta 2.0 database, containing 1,520 target sequence entries (35) or, for the full proteome search, the Swiss-Prot database of *Mus musculus* proteins (retrieved August 2016, containing 16,747 target sequences). Cross-links were reported at a 2% false discovery rate based on a target–decoy calculation strategy.

Bottom-up proteomics data were analyzed using MaxQuant software (version 1.5.6.5) (49). The searching parameters are: MS ion mass tolerance: 10 ppm, MS2 ion mass tolerance: 20 ppm; fixed modification: Cys carbamidomethylation; variable modification: Met oxidation; enzymatic digestion: trypsin; allowed number of missed cleavages: 2; database: MitoCarta 2.0; Estimation of false discovery rate: 1%. intensity-based absolute quantification calculation (50) was enabled to determine to relative abundances of proteins. Label-free quantification (51) was performed to calculate the changes of protein abundances between native and salt-treated mitochondria. Significance is measured by protein fold change > 2 and *p* < 0.05.

## RESULTS

### 

#### 

##### XL-MS of Intact Mitochondria Provides Deep Interactome Coverage and Targets All Submitochondrial Compartments

XL-MS experiments were performed on intact mitochondria isolated from hearts of young adult C57BL/6N male mice. After fixating the native protein structures and interactions by cross-linking with the lysine-reactive DSSO, the reaction was quenched and the cross-linked proteins were analyzed using our previously described XlinkX v2.0 workflow (30). Based on mitochondrial preparations from two separate hearts, imposing a 2% false discovery rate, we obtained 3,322 unique Lys-Lys connections, covering 359 mitochondrial proteins according to the mouse MitoCarta 2.0 database (35) (all sites are listed in Supplementary Data). Compared with a recently published dataset (33), this represents a 73% increase regarding the number of unique Lys-Lys connections, providing the largest cross-link repository reported to date for mitochondria. A more detailed comparison of the two cross-linking datasets is presented in Supplemental Fig. S2. The overlap in unique Lys-Lys cross-links between the two studies is relatively low, likely due to the differences in size, spacer arm length, and physicochemical properties of the applied cross-linkers (PIR in (33) *versus* DSSO in this work). Furthermore, distinct data acquisition and data analysis pipelines may also contribute to the differences. The overlap of protein–protein pairs and proteins detected in cross-links is substantially higher. We think the two datasets are complementary, as these two workflows likely capture different aspects of the mitochondrial interactome.

To gauge the depth and sensitivity of our XL-MS approach, we analyzed non-cross-linked mitochondria by bottom-up proteomics, obtaining protein abundance estimates using the well-established label-free intensity-based absolute quantification approach (50). Of the 359 proteins in our cross-linking dataset, 344 were also detected and quantified by bottom-up proteomics. In total, the proteomic analysis identified 729 proteins, demonstrating that our XL-MS approach covers ∼47% of the mitochondrial proteins detected by bottom-up proteomics (Supplementary Data). As further discussed in Supplemental Fig. S3, cross-links are, as expected, enriched in highly abundant proteins. However, it is noteworthy that the proteins, for which cross-links could be identified, span a dynamic range of at least four orders of magnitude, demonstrating the current analytical depth of our XL-MS approach.

**Fig. 1.**
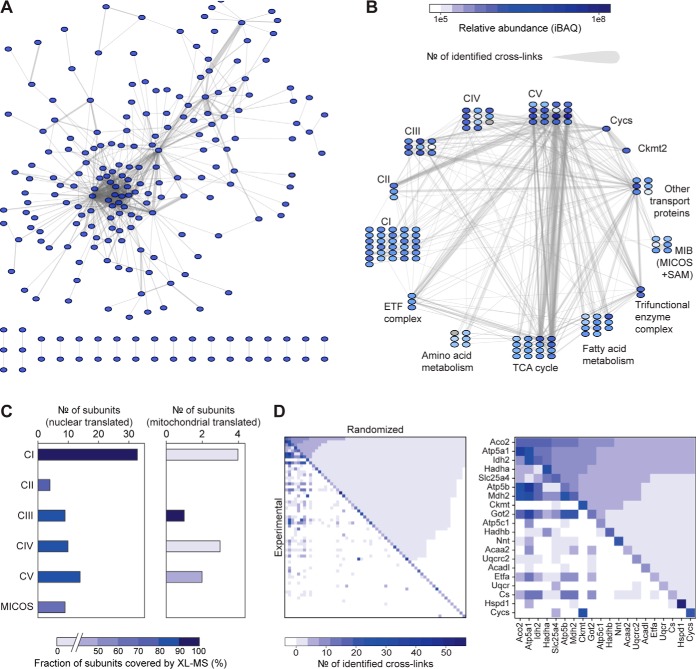
**The XL-MS-based mitochondrial interactome.** (*A*) Overview of all protein–protein contacts observed by XL-MS. (*B*) Contacts observed between selected groups of mitochondrial proteins. Shown are only proteins with interprotein links. All abbreviations are explained in the main text, except for ETF = electron transfer flavoprotein, Cycs = cytochrome *c*, Ckmt2 = mitochondrial creatine kinase, MICOS = mitochondrial contact site and cristae organizing system, SAM = sorting and assembly machinery. (*C*) Cross-link coverage for subunits of the major protein complexes in the inner mitochondrial membrane. (*D*) Matrix of pairwise protein–protein contacts either observed by XL-MS (“Experimental”) or calculated assuming random cross-link formation (“Randomized”, *i.e.* only based on protein abundance and number of Lys residues). Proteins are sorted based on their intensity-based absolute quantification values. The right plot shows an expanded view of the 20 most abundant proteins of the left plot.

The cross-links uncover a highly interconnected network of mitochondrial protein interactions with 60% of the detected cross-links (2,041 out of 3,322) being formed between different proteins (Fig. 1*A*). This observation is significantly different from our previous proteome-wide XL-MS studies on cell lysates of *Escherichia coli* and HeLa, where only 10–20% of the cross-links connect different proteins (30, 31). The higher percentage of interprotein cross-links in intact mitochondria is likely due to the higher protein concentrations in intact organelles and cells. Cells have an average protein density of 1,000 mg/ml (52), which is almost 1,000 times higher than in most *in vitro* or lysate preparations, and especially the mammalian mitochondrial matrix is known to be densely packed with proteins (23). Furthermore, some of the interprotein cross-links probably reflect transient and weak protein interactions as these are more likely to be preserved when proteins are cross-linked in their unperturbed organellular environment. This makes XL-MS analysis of isolated organelles to some extent complementary to the majority of traditional structural and biochemical techniques, which often infer protein interactions based on experiments performed in *in vitro* environment (*e.g.* after affinity purification or *in vitro* recombination of proteins).

Reassuringly, the DSSO cross-linker, likely due to its relatively small size, efficiently penetrates the mitochondrial membranes, allowing us to probe protein interactions in all compartments of the mitochondria, *i.e.* the outer mitochondrial membrane (OMM), the intermembrane space (IMS), the IMM, and the matrix. Examples of cross-linked protein assemblies with different submitochondrial localizations are displayed in Fig. 1*B*, including the OMM-IMM-spanning mitochondrial intermembrane space bridging complex, the IMM-spanning complexes involved in the OXPHOS pathway, and several matrix-localized citric acid-cycle-related enzymes.

The identified cross-links touch upon several levels of mitochondrial protein organization. First, they cover 75% of the mitochondrial protein complexes described in the CORUM database (53) of human protein complexes (better annotated than the mouse database), which is commonly used as a standard reference in interactome studies (54–56). A closer examination of several multisubunit complexes localized in the IMM reveals that our cross-linking data cover 80–100% of subunits reported in the CORUM database, especially nuclear translated subunits (Fig. 1*C*). The lower coverage for mitochondrial translated subunits is likely due to the fact that most of these subunits constitute the hydrophobic core of the studied IMM-spanning protein complexes, which are largely buried from the protein surface and contain relatively few Lys residues.

Second, the identified cross-links provide connectivity information for known protein complexes that have not yet been described by high-resolution structures, such as the multimeric pyruvate dehydrogenase complexes, the 2-oxoglutarate dehydrogenase complexes, and the mitochondrial intermembrane space bridging complex (Supplemental Figs. S4*A* and S4*B*). Moreover, several cross-links confirm protein interactions observed in a complementary affinity purification-MS-based study (8). For instance, we identified four cross-links between Lyrm5 and electron transferring flavoprotein complex, and two cross-links between Coq3 and Coq6 (Supplemental Figs. S4*C* and 4*D*). These cross-links indicate that the observed interactions are genuine and, though not at the center of this study, that our dataset may provide valuable input to guide future computational modeling/docking studies or aid the EM-based characterization of mitochondrial protein complexes.

Notwithstanding the high degree of interconnectivity, we argue that our cross-linking profile is a consequence of the highly ordered mitochondrial architecture rather than a random unorganized protein pool. To confirm this hypothesis, we compared our cross-linking data to a randomized binary interaction network. The randomized network was created based on the established protein abundances (measured by intensity-based absolute quantification) and number of reactive sites (measured by the number of Lys residues), assuming that proteins are randomly distributed in the mitochondria and the interaction of a residue pair is merely defined by their abundances. This comparison shows that the experimentally detected network deviates significantly from the randomized interaction model (Fig. 1*D*). A more detailed examination of individual protein complexes evidences this point further. For instance, Immt (Mic60) and Atp5b are two IMM proteins that display numerous cross-links (129 links for Immt and 294 links for Atp5b). However, Immt primarily cross-links with other mitochondrial intermembrane space bridging complex subunits and IMM-spanning proteins whereas Atp5b cross-links to many metabolic proteins in the mitochondrial matrix. The disparate behavior of Immt and Atp5b is indicative of their function and location in the membrane: Immt is involved in formation and maintenance of mitochondrial cristae structure (57, 58), whereas Atp5b is the stable matrix-facing beta subunit of F1F0 ATPase and therefore likely to be in close connection with proteins involved in energy demanding metabolic functions.

Collectively, our XL-MS study revealed a dense mitochondrial protein interaction network, covering most of the annotated protein complexes in the CORUM database. The cross-links reflect the distinct organization of intact mitochondria and thus render valuable information on the higher-order organization of proteins and the architecture of protein complexes, as described hereafter.

##### XL-MS Reveals Mitochondrial and Submitochondrial Localization of Proteins

**Fig. 2.**
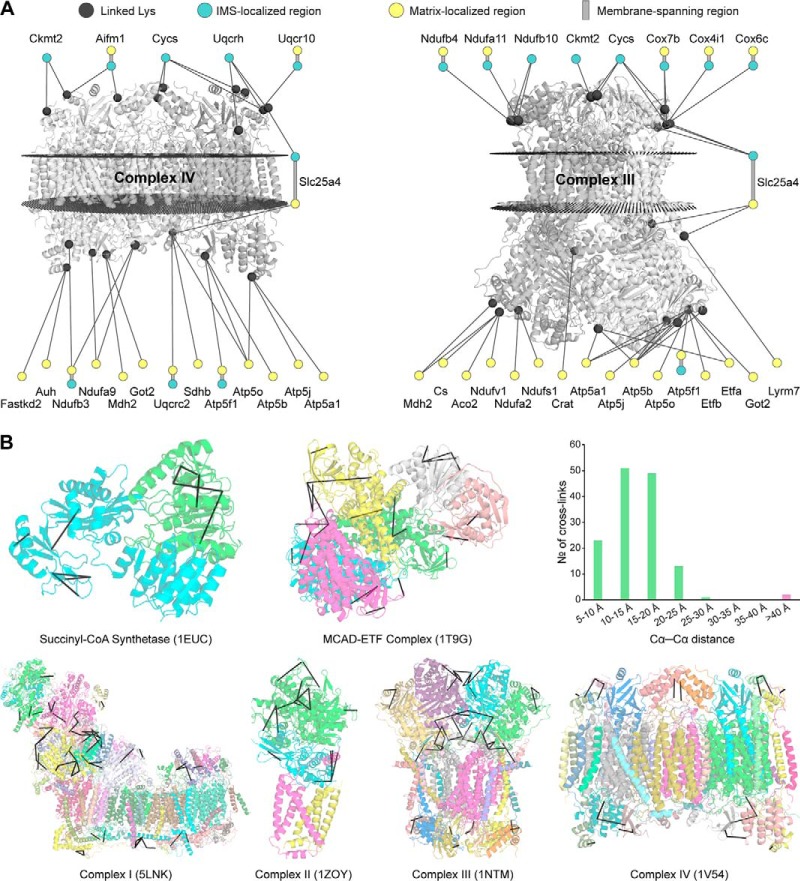
**Subcompartment localization of example proteins and validation of the XL-MS approach.** (*A*) Submitochondrial interaction networks of the ETC complexes CIII and CIV. Only interaction partners with known submitochondrial localizations are displayed. Those localizations are indicated using color. (*B*) Mapping of the detected cross-links (black lines) onto high-resolution structures of selected mitochondrial protein complexes. If the cross-link location is ambiguous (*e.g.* in homodimers) all possible locations are displayed. The distribution of observed Cα–Cα distances is shown in the top right corner. Here, every unique cross-link was counted once.

Since we detected cross-links in all submitochondrial compartments, we tested whether cross-linking affects the integrity of the mitochondrial membranes. We inspected the interaction networks of the IMM-localized ETC complexes III and IV, which exhibit IMS- as well as matrix-facing subunits. Focusing on their cross-links to proteins with unambiguously localized Lys residues, we could validate that none of the identified cross-links traverses across the IMM (Fig. 2*A*). This suggests that XL-MS preserves the membrane integrity and can thus provide information on the submitochondrial localization of proteins.

Next, we queried whether our dataset contained proteins not yet known to localize to mitochondria. Making use of XlinkX's capability to identify cross-links against unrestricted sequence databases, we re-searched our data against the full mouse proteome and filtered for proteins that show at least two unique Lys-Lys connections to known mitochondrial proteins. This analysis yielded four proteins that are currently not present in the mouse MitoCarta 2.0 database: Mmaa, Junb, Stau1, and Mum1 (Supplementary Data). Mmaa has been reported previously as a binding partner of mitochondrial methylmalonyl-CoA mutase (59). Our cross-linking results further confirmed its localization at the mitochondrial matrix side (Supplemental Fig. S5).

Stau1 is a dsRNA-binding protein that was reported to localize to the endoplasmic reticulum (60). Here we identified two cross-links, which were confirmed by 20 independent MS sequencing events, connecting Stau1 with the mitochondrial Acetyl-CoA acetyltransferase (Acat1), a soluble enzyme localized in the mitochondrial matrix. Consistent with its mitochondrial matrix localization, we observed cross-links between Acat1 to several established mitochondrial proteins (Supplementary Data). Our data suggest that, in addition to the endoplasmic reticulum, Stau1 is localized in the mitochondrial matrix and may potentially have a functional interaction with Acat1.

Junb and Mum1 are known as nuclear proteins involved in cell cycle regulation and DNA damage response, respectively (61, 62). Neither of them has been associated with mitochondria so far, although Junb activity was reported to counteract mitochondrial stress (63). Our data indicate that both proteins can localize to mitochondria, possibly as a result of dual targeting, which is quite common among mitochondrial proteins (64). Specifically, we found Junb cross-linked to the trifunctional enzyme complex in the IMM and Mum1 cross-linked to CV and mitochondrial 3-ketoacyl-CoA thiolase.

##### XL-MS Directly Captures Native Protein Structures

After interrogating the mitochondrial organization, we also assessed the structural validity of the cross-links based on the residue-to-residue distance limit imposed by the DSSO cross-linker. For this purpose, we selected six stable protein complexes (Succinyl-CoA synthetase, MCAD-electron transferring flavoprotein complex, and ETC CI, CII, CIII, and CIV) and mapped the identified cross-links onto available high-resolution structures (Fig. 2*B*). Out of 139 mapped cross-links, 137 were formed between residues with Cα–Cα distances of less than 30 Å. This corresponds well with the expected distance range of DSSO, for it is known that the BS3 cross-linker, which has a similar spacer arm length as DSSO, can bridge Cα–Cα distances up to 30 Å or, in a few cases, even 40 Å (65). Therefore, 137 out of 139 cross-links can be regarded as structurally confirmed, which agrees well with the 2% false discovery rate of the cross-linking dataset. The two long-range cross-links, identified within CII and MCAD electron transferring flavoprotein, respectively, may either be false-positive identifications or indicate the presence of higher-order protein complexes and/or alternative conformers.

Furthermore, it is noteworthy that none of the mapped cross-links spans over intramembrane regions of the protein complexes, supporting the premise that under the cross-linking conditions applied the investigated mitochondria are intact.

##### XL-MS Reflects Higher-Order Organizations of ETC Protein Complexes In Vivo

Extrapolating from the high fidelity of the cross-links mapped to known complexes, we reason that links between individual complexes present direct evidence of their close spatial proximity, providing strong indications for their assembly into higher-order supercomplexes *in vivo*. To explore this point, we focused on the intercomplex links among the ETC complexes CI, CIII, and CIV. Assembly of these complexes into supercomplexes have been mainly visualized *in vitro* by a variety of biochemical methods, almost always involving detergent-based preparations, most popularly by digitonin native gels (15, 66, 67). Recently, the first cryoEM structures of isolated supercomplexes were reported, all exhibiting a CI-CIII_2_-CIV stoichiometry (17–19).

**Fig. 3.**
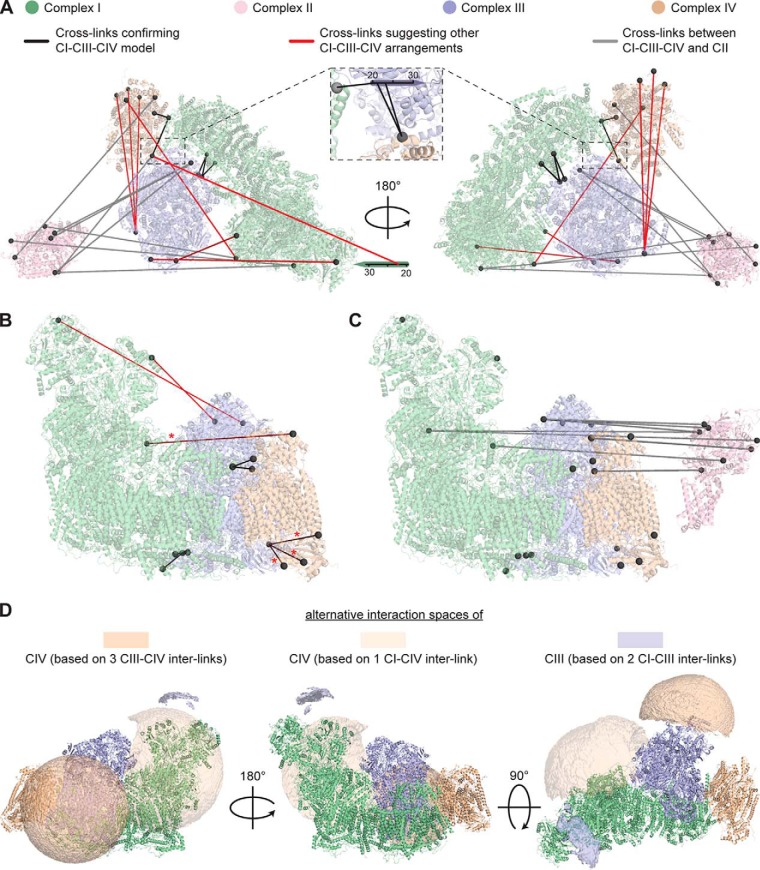
**Structural analysis of the ETC supercomplexes.** (*A*) Cross-links mapped onto a pseudo-atomic model of the CI-CIII_2_-CIV supercomplex (PDB codes 5J4Z and 5LNK, Methods) and a crystal structure of CII (PDB code 1ZOY). Structurally uncharacterized regions are represented by sequence bars. (*B*, *C*) 90° tilted view of A, showing the cross-links within CI-CIII_2_-CIV (*B*) and toward CII (*C*). Cross-links are color-coded as in *A*. Red asterisks indicate red cross-links that appear darker because they are overlaid by parts of the structure. (*D*) Alternative interaction space models of the ETC complexes CIII and CIV. The interaction spaces models were calculated based on cross-links that disagree with the CI-CIII_2_-CIV supercomplex model (red lines in *A*) and are shown as semitransparent surfaces.

Cross-linking efficiently captured the interactions of ETC complexes, as illustrated by immunoblot analysis (Supplemental Fig. S6) and XL-MS. For the XL-MS data analysis, we confine our scope to the CI, CIII, and CIV subunits that were characterized in the recent cryoEM studies (18, 68). Cross-links involving ETC complex subunits that are structurally uncharacterized by cryoEM will be discussed in the final section. We identified 15 intercomplex links, including five CIII-CIV interlinks, four CI-CIV interlinks, and six CI-CIII interlinks. Of these, eight cross-links either agree with the DSSO distance limit or, in cases where one linked residue is just outside the structurally characterized region, likely comply with the binding interfaces in the CI-CIII_2_-CIV supercomplex model. The other seven cross-links (three CIII-CIV links, two CI-CIV links, and two CI-CIII links) clearly violate the cross-linker distance restraint (Figs. 3*A* and 3*B*). Based on the violating intercomplex cross-links, we mapped alternative interaction spaces of CIII and CIV using the DisVis software (69). The resulting interaction spaces, so-called isosurfaces, summarize all center-of-mass localizations of CIII and CIV satisfying the cross-link distance restraints (Fig. 3*D*). Interestingly, cross-links involving CIV cannot be reconciled in one interaction space, suggesting at least two alternative locations. Furthermore, cross-links connecting CI and CIII suggest that CIII can position “on top” of the matrix side of complex I. Since all ETC complexes are transmembrane protein complexes, this localization points toward a strongly curved membrane or an altered conformation of CI's matrix-exposed region.

Next, we investigated the interactions between CII and the CI-CIII_2-_CIV supercomplex. Although CII is a canonical component of mitochondrial redox carriers, the interaction of CII with other ETC complexes has not been previously observed by cryoEM. In contrast, our cross-linking data provide strong evidence for this interaction, as we identified 8 Lys-Lys connections between CII and the other ETC complexes (Figs. 3*A* and 3*C*). All cross-links connect the matrix-facing iron-sulfur and flavoprotein subunits of CII with Lys residues located at the matrix side of the CI-CIII_2-_CIV supercomplex. This further strengthens the notion that our cross-linking experiment captured native arrangements of the ETC protein complexes in an intact IMM. The eight intercomplex links of CII (three CII-CI links, three CII-CIII links, and two CII-CIV links) suggest that CII has multiple contact sites. This could be due to the coexistence of several conformations and/or stoichiometries of CI-CII-CIII-CIV supercomplexes or subsections thereof. Moreover, all four ETC complexes were found cross-linked to CV, indicating that all five OXPHOS complexes are involved in supercomplexes, as we will further discuss below.

##### XL-MS Captures Interaction Changes of the OXPHOS Supercomplexes

To characterize the supercomplex binding interfaces more specifically, we attempted to disrupt the supercomplexes and profile the resulting interactomic changes by XL-MS. Supercomplex disruption in isolated mitochondria has previously been achieved by detergent solubilization, usually with triton or maltoside, in a variety of tissues and organisms (15) or a combination of low pH and succinate in plant mitochondria (70). We chose to avoid the use of detergent because it will likely affect mitochondrial membrane integrity. Furthermore, we did not obtain efficient supercomplex disruption when replicating the low pH and succinate treatment (Supplemental Fig. S7*A*). Therefore, we set out to develop an alternative method to effectively dissociate the supercomplexes while retaining individual complexes within the membrane. We reasoned that the classical use of high ionic strength, which can compete with protein–protein or protein–water electrostatic attractions (71), coupled to warm temperature to permit membrane protein diffusion, could be adopted for the purpose.

**Fig. 4.**
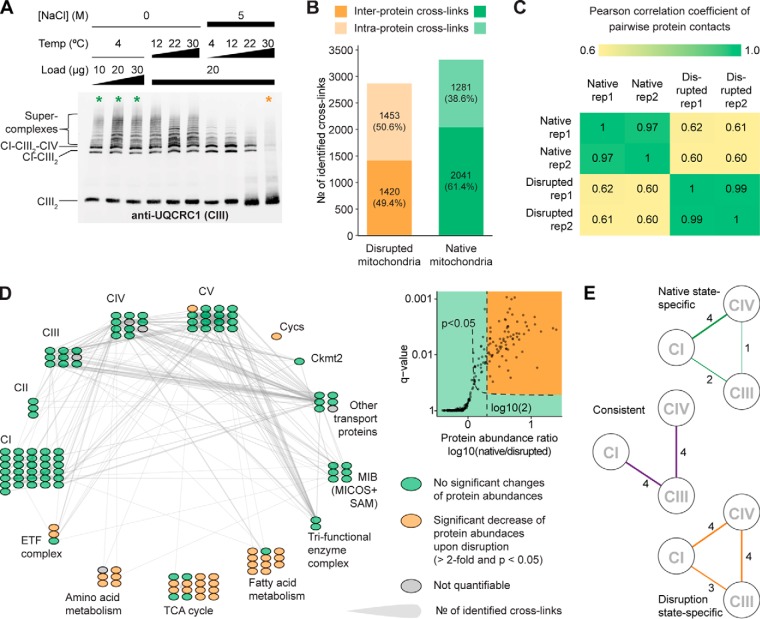
**Disruption of the ETC supercomplexes.** (*A*) Western blot analysis showing temperature and dose dependence of salt-induced supercomplex disruption. Colored asterisks indicate the untreated state (show in green) and the salt-treated state (shown in orange). (*B–E*) Comparative XL-MS analysis of native and disrupted mitochondria. (*B*) Number of cross-links identified in native and disrupted mitochondria. (*C*) Pearson correlation of the number of pairwise protein contacts between biological replicates of the same state (native *versus* native and disrupted *versus* disrupted) and different states (native *versus* disrupted). (*D*) Interaction network of disrupted mitochondria. Proteins are arranged as in Fig. 1*B*. Significant changes of protein abundances are indicated in color. Significance was measured by a one-sided *t* test (*p* < 0.05) and abundance changes were determined by label-free quantification. (*E*) Disruption-induced changes in the connectivity of CI, CIII, and CIV. The line width indicates the number of unique residue-to-residue cross-links between the displayed complexes. Lines are color-coded according to the origin of the cross-links (green: only detected in native mitochondria, orange: only detected in disrupted mitochondria, purple: detected in both conditions).

Optimal supercomplex dissociation was observed after incubating the mitochondria for one hour in 5 m NaCl at 30 °C as detected by the elimination of supercomplex bands in maltoside blue native gels (Fig. 4*A*). This result implies that the ETC supercomplexes are held together, at least in part, by electrostatic interactions, which can be disrupted at high NaCl concentrations. More modest treatments (including milder temperature or lower NaCl concentration, Supplemental Fig. S7*B*) were not sufficient to disrupt the supercomplexes, nor did a variety of other treatments screened (data not shown), arguing that once formed, they are held together tightly. The high salt treatment, however, enabled irreversible supercomplex dissociation, since re-association was not observed after returning the disrupted sample to normal conditions (Supplemental Fig. S7*C*).

We performed XL-MS on a disrupted aliquot of the same mitochondrial preparations used for the above analysis of native mitochondria. This yielded 2,873 cross-links, which is in a similar range as the 3,322 cross-links detected in native mitochondria (Supplementary Data). Globally, disruption substantially reduced the inter-to-intralink ratio from 1.59 to 0.98, indicating a salt-induced loss of protein–protein contacts (Fig. 4*B*). We also performed correlation analysis between native and disrupted mitochondria, taking into account the number of unique Lys-Lys cross-links in each protein pair. Our results show that biological replicates of the same condition (average correlation coefficient R ∼ 0.98) correlate significantly higher than the two different biological conditions (average correlation coefficient R ∼ 0.61), demonstrating that the mitochondrial interactome changed dramatically upon disruption (Fig. 4*C* and Supplemental Fig. S8*A*).

To assess the salt-induced disruption effect on native protein complex structures, we first mapped the cross-links detected after disruption onto the same structural models that we initially used for validating our XL-MS approach (see Fig. 2B). The fraction of cross-links agreeing with the published structures has modestly decreased (83.2% after disruption *versus* 98.6% using native mitochondria), suggesting that the disruption may partially alter protein complex architectures (Supplemental Figs. S8*B* and 8*C*).

Quantitative bottom-up proteomics of the disrupted sample revealed significantly reduced abundances of soluble proteins, such as Cycs (localized in the IMS), and many enzymes involved in the citric acid cycle and amino acid and fatty acid metabolism (localized in the matrix). This partially explains the substantial reduction of cross-links involving these proteins (Fig. 4*D*). However, all proteins of the OXPHOS complexes are retained in the salt-treated mitochondria (Fig. 4*D* and Supplementary Data), allowing further investigation of their interaction patterns.

We analyzed how the disruption affected the cross-links between ETC complexes CI, CIII, and CIV. Despite the efficient supercomplex disassembly seen with digitonin blue native gels, we still detected 19 intercomplex cross-links between CI, CIII, and CIV. Conceivably, these cross-links are detected because the ETC complexes are still kept in close proximity by the IMM, although many of their authentic interaction sites may have been displaced. The cross-links detected after salt treatment may thus represent non-native contact sites that are not sufficient to maintain the supercomplex structures in the detergent milieu used for the blue native gel approach, as the applied detergent digitonin is known for its potential to affect protein interactions (66). In support of this hypothesis, the percentage of overlapping intercomplex cross-links between the two conditions is low (0% for CI-CIV interaction, 44% for CI-CIII, and 44% for CIII-CIV interactions), indicating substantial alterations of their contact sites (Fig. 4*E* and Supplemental Fig. S9). It is important to note that disruption of the supercomplexes was associated with a loss of several CI-CIII-CIV cross-links. Specifically, four CI-CIV, two CI-CIII, and one CIII-CIV connections were lost upon disruption. These data, coupled with the major supercomplex disruption seen on blue native gel, support the notion that the interactions captured by the salt-susceptible cross-links may represent electrostatic interactions that are important in the native formation of supercomplexes. Interestingly, all of the consistently detected cross-links involve CIII subunits (Fig. 4*E* and Supplemental Fig. S9*D*). This suggests that the CIII exhibits the most stable binding interfaces that are, however, not sufficient to maintain the CI-CIII-CIV supercomplex on native gels.

**Fig. 5.**
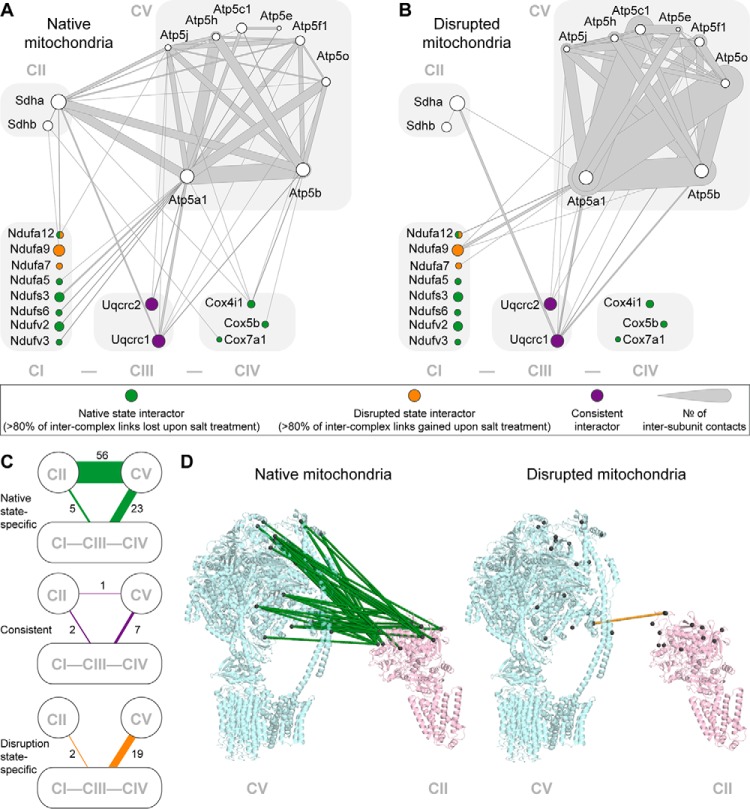
**Interactions among the OXPHOS complexes in native and disrupted mitochondria.** (*A*, *B*) Cross-links among structurally characterized subunits of CII, CV, and the CI-CIII-CIV supercomplex in native (*A*) and disrupted (*B*) mitochondria. Shown are only subunits included in published high-resolution structures of mammalian OXPHOS complexes. Subunit coloring indicates their interaction pattern. Ndufa12 has two colors because it shows two distinct sets of interaction partners in native and disrupted state. (*C*) OXPHOS connectivity maps. The line width indicates the number of unique residue-to-residue cross-links. Lines are color-coded according to the origin of the cross-links (green: only detected in native mitochondria, orange: only detected in disrupted mitochondria, purple: detected in both conditions). (*D*) Structural representation of CII-CV cross-links in both conditions (PDB codes 1ZOY and 5ARA).

In addition to the CI-CIII-CIV supercomplex, we also examined the cross-links between CI-CIII-CIV and the other two essential components of OXPHOS: CII and CV (Figs. 5*A* and 5*B*). For CI, the CI-CII and CI-CV interfaces have both completely changed, now involving a vastly different set of interacting subunits. For CIV, all CIV-CII and CIV-CV interactions have been abolished. For CIII, in contrast, CIII-CII as well as CIII-CV cross-links have largely remained constant, confirming that CIII exhibits the most resilient binding interfaces. Cumulatively, both CII and CV show substantial changes in their cross-linking pattern to CI-CIII-CIV (22 and 14% cross-link overlap, Fig. 5*C*), suggesting that all complexes are still in close proximity but have undergone substantial rearrangements upon disruption. The most striking disruption effect is presented by the CII-CV interface. Here, the number of cross-links dropped from 57 in native mitochondria to only 1 after salt treatment, indicating an almost complete dissociation of these two protein assemblies (Fig. 5*D*). Collectively, these results show that salt treatment globally affects the spatial organization of the OXPHOS complexes.

**Fig. 6.**
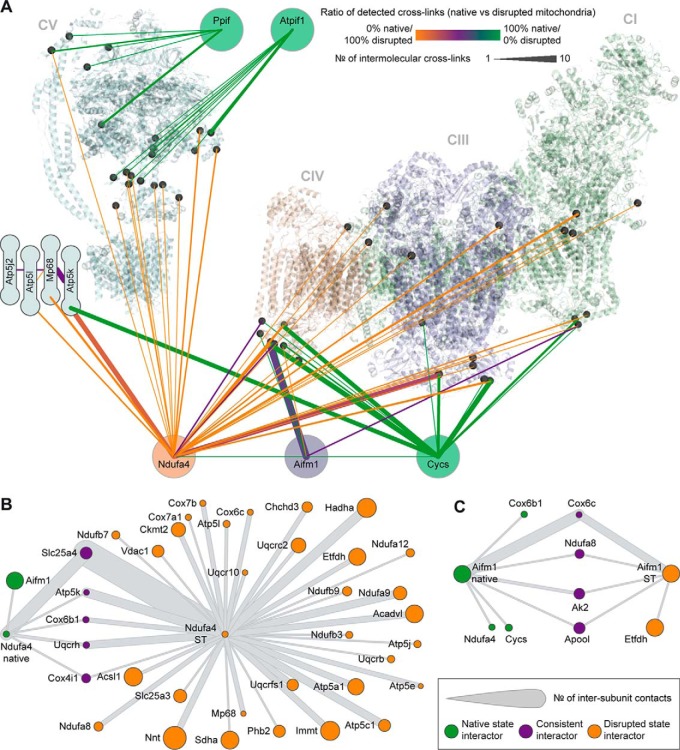
**Effects of mitochondrial disruption on selected interaction partners of the OXPHOS complexes.** (*A*) Differential regulation of protein interaction patterns upon disruption. The CI-CIII_2_-CIV supercomplex (*right*) and CV (*left*) are shown as semitransparent cartoon models. Structurally uncharacterized ATP synthase subunits are schematically depicted. Lys residues involved in cross-links are displayed as black spheres. The line color indicates under which condition the respective connection is prevalently detected. The line width indicates the number of unique residue-to-residue cross-links. CII showed only one interlink to the interacting proteins (to Ndufa4 in disrupted mitochondria) and is therefore not depicted. (*B*, *C*) Interaction networks of Ndufa4 (*B*) and Aifm1 (*C*) in native and salt-treated mitochondria.

To expand our analysis further, we examined the specific changes among well-known interaction partners and structurally uncharacterized subunits of the OXPHOS complexes: the electron carrier Cycs, the CI assembly factor Aifm1, the structurally uncharacterized CIV subunit Ndufa4, the CV inhibitory factor Atpif, and the CV chaperone PPif (Fig. 6). By comparing the interactions of these proteins in salt-treated and untreated mitochondria, we discovered several specific behaviors. For instance, Aifm1 retained most of its interactions upon disruption, whereas Cycs, Atpif1, and Ppif lost all their cross-links after salt treatment (Fig. 6*A*), likely because they show a more than fourfold reduction in protein abundance in disrupted mitochondria (Supplementary Data). Indeed, it is well-known that Cycs is released from the IMS upon mitochondrial perturbation (36).

In addition to cross-links that were retained or lost, salt treatment also introduced disruption-specific interactions. Most of these cross-links involved Ndufa4, a subunit of CIV that is not present in any high-resolution structure. In untreated mitochondria, Ndufa4 was found cross-linked to CIII (two links), CIV (two links), and CV (one link) at the IMS surface. In disrupted mitochondria, however, it gained many more cross-links in both the IMS and the matrix (Figs. 6*A* and 6*B*). This indicates that Ndufa4 is partially relocated from the IMS to the matrix upon disruption. Therefore, we conclude that this protein is sensitive to electrostatic disruptions, prone to relocalization, and able to engage in promiscuous binding when perturbed. These observations may explain why Ndufa4 has not yet been captured by crystallography or cryoEM and was initially annotated as a subunit of CI, which has been reassigned as a subunit of CIV only recently (72). This contrasts with the ETC-binding chaperone Aifm1, which exhibits an interactome that is largely resistant to disruption (Figs. 6*A* and 6*C*).

## DISCUSSION

### 

#### 

##### XL-MS Approach of Intact Mitochondria

Our XL-MS approach relies on the combination of the commercially available gas-phase cleavable cross-linker DSSO, hybrid MS acquisition methods available on the Orbitrap Fusion and Fusion Lumos mass spectrometers and the publicly available cross-link search engine XlinkX v2.0 (30). Using this strategy, we identified the most comprehensive mitochondrial interaction network to date that, as shown by statistical analysis and comparative analysis of salt-treated mitochondria, is highly specific and reproducible. The presented dataset can be used to predict several metabolon structures and protein complexes, as well as constraints or validation points for (pseudo-) atomic structural models obtained with cryoEM approaches. This latter application of XL-MS is illustrated in recent cryoEM studies of complete CI, which also relied on XL-MS-generated constraints (68, 73).

The structural validity of the detected links was proven by comparing them to available high-resolution structures of stable protein complexes and by showing that cross-links are confined to specific submitochondrial compartments. The XL-MS approach thus retains native protein structures and mitochondrial membrane integrity.

Compared with previous proteome-wide interactome studies by XL-MS or other biochemical approaches, the herein presented data highlight three main gains. First, our XL-MS study covers a large percentage of mitochondrial proteins detected by bottom-up proteomics. A major drawback of previous proteome-wide XL-MS approaches, including our own, has been the limited depth, capturing mainly interactions of highly abundant proteins. While the detected mitochondrial cross-links are still enriched at high protein abundance levels, we cover ∼50% of the detected mitochondrial proteome over an abundance range spanning at least four orders of magnitude.

Second, XL-MS reveals a highly interconnected mitochondrial interactome, allowing the characterization of supramolecular assemblies. This contrasts our previous XL-MS studies on cell lysates, where mostly the interactions of very stable complexes could be examined. Applying cross-linkers to intact cells or subcellular compartments instead of lysates captures potential interactors based on the *in situ* distance proximity of proteins. This concept of XL-MS is somewhat similar to other proximity labeling techniques, most notably BioID and APEX (74, 75). On the one hand, BioID relies on the fusion of a biotin ligase to a protein of interest that biotinylates proximal proteins (76). APEX, on the other hand, involves a genetically targetable peroxidase enzyme APEX/APEX2 to biotinylate nearby proteins (77–79). BioID and APEX share several key similarities with XL-MS. Since distance proximities are captured when the cells or organelles are intact, these approaches are capable of visualizing weak and/or transient protein contacts. Moreover, all three strategies are highly beneficial for studying insoluble proteins and membrane-associated proteins, two classes of proteins that are refractory to many conventional biochemical approaches. Notwithstanding these conceptual similarities, BioID/APEX are closer to classical proteomics approaches, conferring proximity information through the identification of linear peptides. In contrast, XL-MS renders specific maximum distance limits between residues since covalent connections between two peptides are identified, Therefore, XL-MS may provide a complementary picture of the architecture and higher-order organization of (membrane) protein assemblies, such as the OXPHOS complex. Nevertheless, it is noteworthy that similar to BioID/APEX, XL-MS experiments merely reveal protein pairs that are spatially close, which does not necessarily imply a functionally relevant interaction. Careful analysis of the detected cross-links is indispensable to obtain relevant structural and functional insights.

Third, XL-MS allows efficient interactome profiling in different biological conditions. Currently, such interactome studies are mostly conducted using affinity purifications and BioID (55, 80, 81); however, these approaches are extremely labor intensive since they only target a single protein of interest at a time. In contrast, XL-MS allows simultaneous investigation of various endogenous protein complexes, thus making it very attractive to profile interactome changes in different biological scenarios. In this study, two parallel XL-MS analyses, on untreated *versus* salt-treated mitochondria, permitted side-by-side interaction networks immediately visualizing the changes in the interactome. Focusing on the protein complexes involved in OXPHOS, we observed significant alterations. Moreover, the described features of XL-MS enabled us to localize the condition-dependent binding sites of OXPHOS interactors (Ppif1, Atpif1, Aifm1, Ndufa4, and many others) and track the salt-induced relocalization of Ndufa4. Collectively, these benchmarks illustrate the great potential of XL-MS experiments for the detailed profiling of higher-order molecular organizations and interactomic changes under various physiological and pathological conditions.

##### Native Architectures of ETC and OXPHOS

One of our prime interests concerns the native architecture of the ETC components, particularly the potential formation of supercomplexes in intact mitochondria. The hypothetical binding between ETC complexes has fascinated researchers since the theory was proposed in 1955 (82). Several physiological roles of the supercomplexes have been hypothesized, including kinetic arguments related to substrate (coenzyme Q and cytochrome *c*) sequestration and/or channeling, regulation of activities (electron transport, hydrogen pumping, and reactive oxygen species production) through concerted conformational changes and stabilization of the individual complexes through sharing subunits with chaperone/assembly roles (83). In fact, there does appear to be functional crosstalk between the ETC complexes (84, 85), the simplest explanation for which is direct physical contacts between them.

Despite the clear importance of this question to bioenergetics, empirical evidence to support or reject the existence of supercomplexes has mainly been provided by *in vitro* experiments using native gel electrophoresis (15, 66, 67), liquid chromatography separation, immunoprecipitation, and cryoEM (17–19, 86). All these studies assessed supercomplex levels after partial detergent solubilization of the mitochondrial membranes and proteins, almost exclusively with digitonin present, casting doubt on the *in vivo* relevance of the results.

Here we identified numerous individual cross-links between all OXPHOS complexes in intact mitochondria. However, with the binary tool of XL-MS used in this study, a definitive measure of supercomplex structures is difficult to establish without some type of perturbation to evaluate multiple protein interactions. To this goal, we developed a new methodology to disrupt the supercomplexes, as measured with conventional gel-based methods, using a high-salt treatment. The treatment disrupted the detected supercomplexes while preserving the ETC content in the IMM. Using this approach, we show that the cross-link pattern of the ETC complexes significantly changed. Cross-links that were detected after salt treatment show that the protein complexes remain in close proximity within the IMM but have lost many of their authentic contact sites (*e.g.* those based on electrostatic forces). This results in a significant supercomplex destabilization, complying with the absence of supercomplex bands on blue native gels.

With extensive comparative analyses and structural validation in this study, the supercomplex interactions detected in intact mitochondria should be considered genuine. The data suggest that all the OXPHOS complexes in the densely populated IMM are in close contact and not restricted to isolated regions. These interactions are consistent, arising either from “fully assembled” CI-CII-CIII-CIV-CV supercomplexes or smaller supercomplexes each with a subset of the OXPHOS complexes. The only structurally characterized supercomplex comprises CI-CIII_2_-CIV (17–19), and only parts of the observed cross-links were consistent with the cryoEM-based structures. This suggests that the OXPHOS supercomplexes can adopt additional stoichiometries and architectures in intact mitochondria. Conceivably, the CI-CIII_2_-CIV structures seen with cryoEM represent the most stable conformers out of a variety of natively coexisting supercomplexes. This hypothesis is supported by biochemical and structural evidence for the existence of CI-CIII_2_-CIV_n_ supercomplexes with varying stoichiometries (66, 86), a CI_2_-CIII_2_-CIV_2_ megacomplex (87), and higher-order “respiratory strings” of multiple supercomplexes (67, 68). As such, our XL-MS data add to the body evidence implying that protein–protein interactions play a much more important role in coordination or stability of the complex activities observed in other settings (84, 85).

## DATA AVAILABILITY

The mass spectrometry proteomics data have been deposited to the ProteomeXchange Consortium via the PRIDE (88) partner repository with the dataset identifier PXD006816.

## Supplementary Material

Supplemental Data

## Footnotes

^1^ The abbreviations used are:

CI: complex I
CII: complex II
CIII: complex III
CIV: complex IV
CV: complex V
CID: collision-induced dissociation
CORUM: comprehensive resource of mammalian protein complexes
cryoEM: cryo electron microscopy
cryoET: cryo electron tomography
DSSO: disuccinimidyl sulfoxide
ETC: electron transport chain
ETD: electron transfer dissociation
IMM: inner mitochondrial membrane
IMS: intermembrane space
MCAD: medium-chain specific acyl-CoA dehydrogenase
MS: mass spectrometry
OMM: outer mitochondrial membrane
OXPHOS: oxidative phosphorylation
PIR: protein interaction reporter
PRIDE: proteomics identifications database
XL-MS: cross-linking mass spectrometry.
