# Supplementary material for: The interactome of intact mitochondria by cross-linking mass spectrometry provides evidence for coexisting respiratory supercomplexes
Source: Mol Cell Proteomics. 2017 Dec 8;17(2):216–32. doi: 10.1074/mcp.RA117.000470 (PMC5795388; doi:10.1074/mcp.RA117.000470)
Supplement: Supplemental Data [file supp_17_2_216__index.html]

The interactome of intact mitochondria by cross-linking mass spectrometry provides evidence for co-existing respiratory supercomplexes — Revealing the mitochondrial interactome by XL-MS — The interactome of intact mitochondria by cross-linking mass spectrometry provides evidence for coexisting respiratory supercomplexes — Revealing mitochondrial interactome by XL-MS — Supplemental Data 

# The interactome of intact mitochondria by cross-linking mass spectrometry provides evidence for coexisting respiratory supercomplexes

## Supplemental Data

- Supplementary Figures - Supplementary Figures
- Supplementary Tables - All Supplementary Tables
- Annotated spectra of cross-linked peptides - Annotated spectra of cross-linked peptides
