# Supplementary material for: The interactome of intact mitochondria by cross-linking mass spectrometry provides evidence for coexisting respiratory supercomplexes
Source: Mol Cell Proteomics. 2017 Dec 8;17(2):216–32. doi: 10.1074/mcp.RA117.000470 (PMC5795388; doi:10.1074/mcp.RA117.000470)
Supplement: Supplemental Data [file supp_RA117.000470_133922_0_supp_23974_8zf8c5.pdf]

Supplementary Figures

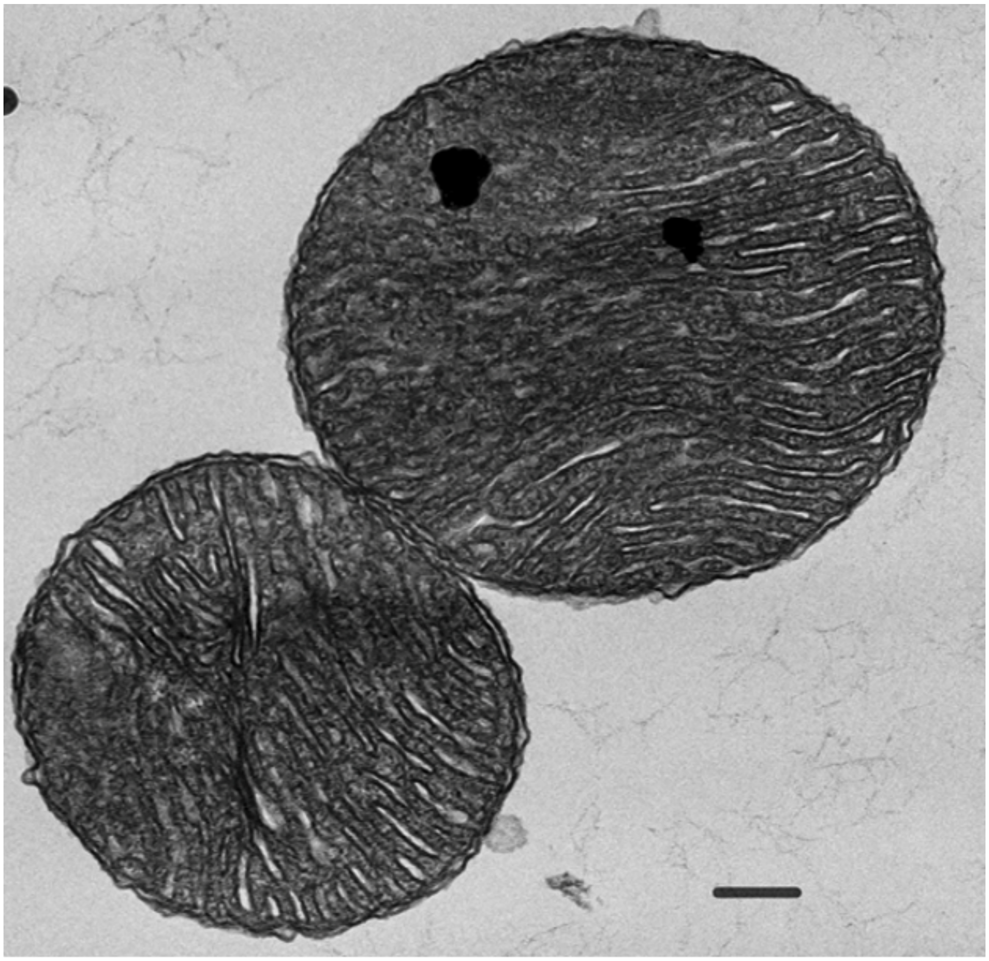

Supplementary Figure S1. EM micrograph of purified mitochondria displaying sub-mitochondrial structural integrity.

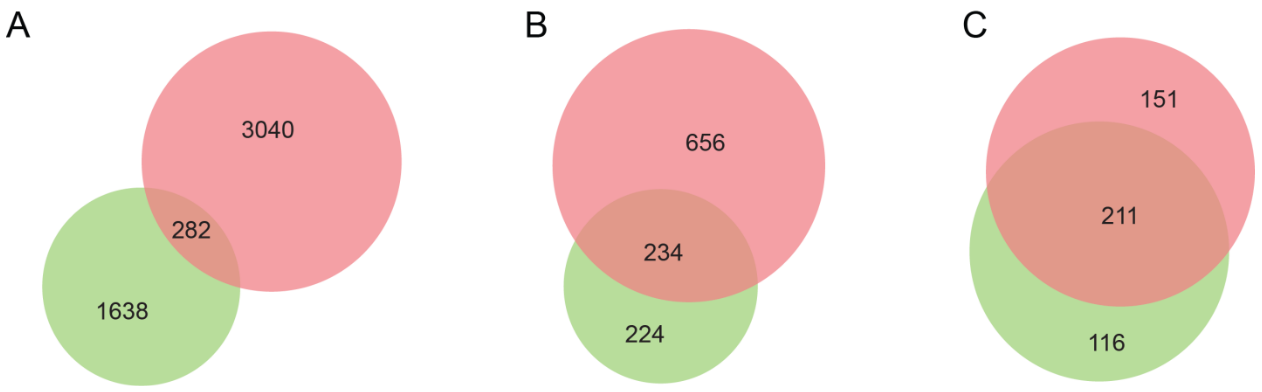

Supplementary Figure S2. Comparison of the cross-links reported by Schweppe *et al*. (green) (1) and this study (red). Venn diagrams displaying the overlap between the two datasets are presented in terms of A) unique Lys-Lys cross-links, B) protein-protein interaction pairs and 3) proteins involved in the cross-links. The overlap of unique Lys-Lys cross-links between the two studies is relatively low, likely due to the differences in size, spacer arm length and physicochemical properties of the applied cross-linkers (PIR in (1) *versus* DSSO in this work). Furthermore, different data acquisition and data analysis pipelines may also contribute to the differences. The overlap of protein-protein pairs and protein detected to be cross-linked is relatively higher.

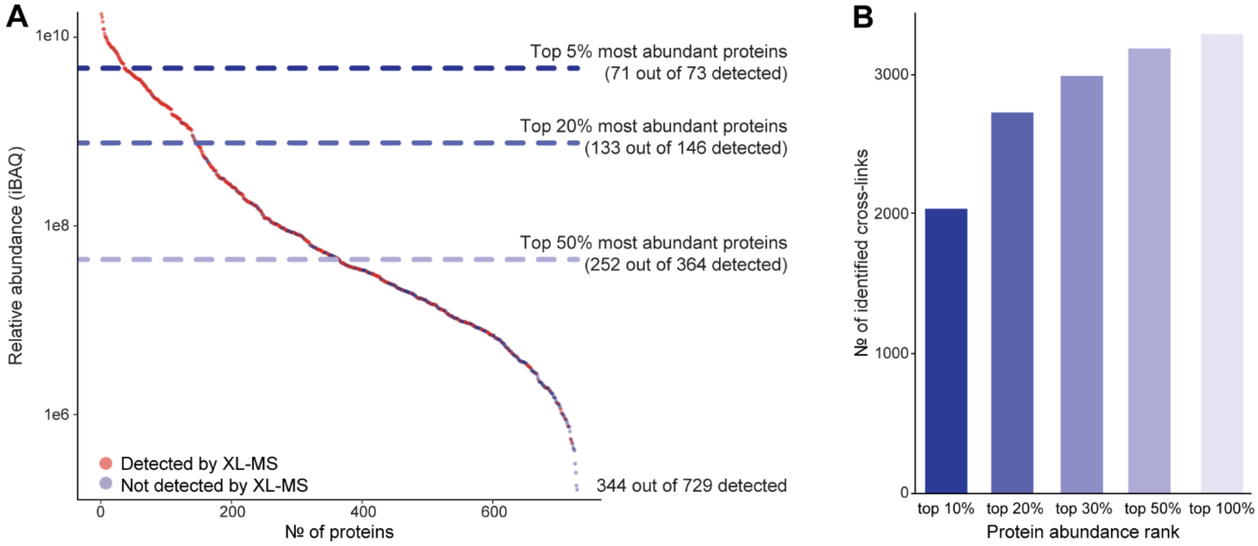

Supplementary Figure S3. Depth of the mitochondrial proteome achieved by XL-MS. (A) The abundance of mitochondrial proteins as estimated by standard bottom-up proteomics (i.e., iBAQ values). Proteins that are included in the XL-MS dataset are shown as red spheres, covering a protein dynamic range of about 4 orders of magnitude. (B) Correlation between the number of identified cross-links and the protein abundance. 60% of the detected cross-links are within the 10% most abundant proteins, however, unique cross-links are also detected for much lower abundant proteins.

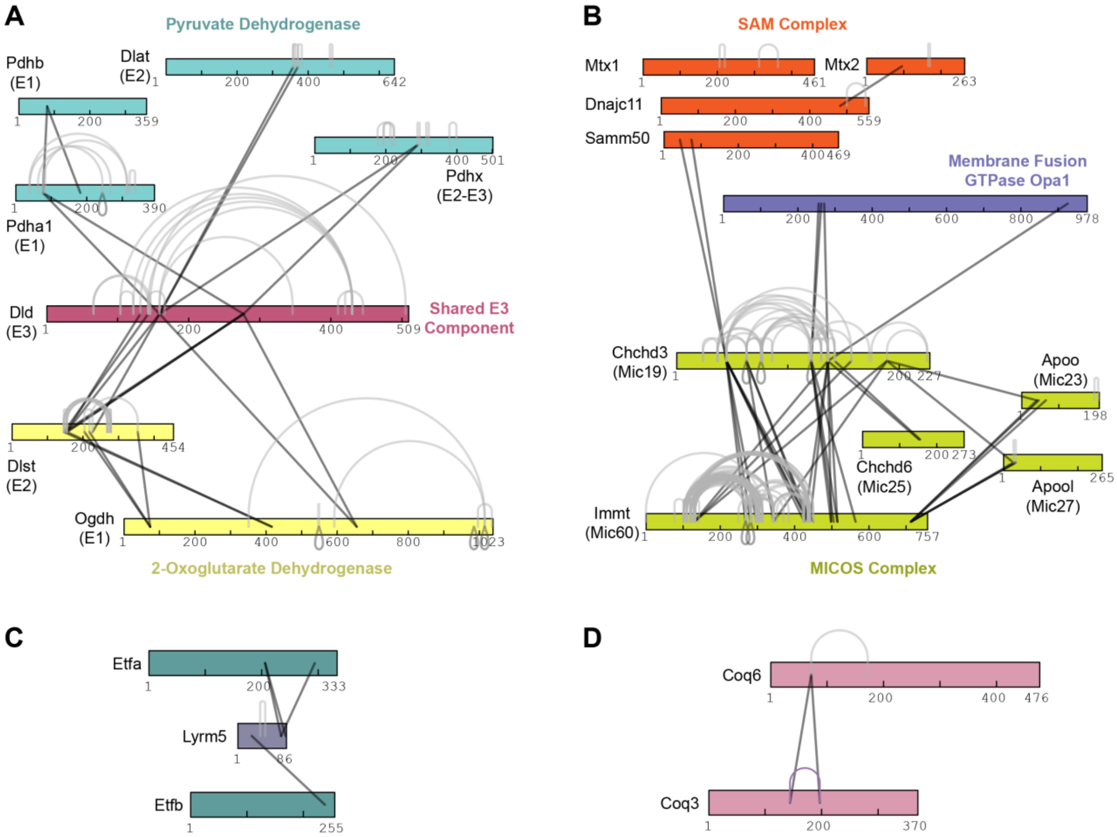

Supplementary Figure S4. Representative examples of mitochondrial protein complexes detected by XL-MS for which no high-resolution structural models are available. (A) Interaction map of the TCA cycle-related enzyme complexes pyruvate dehydrogenase and 2-oxoglutarate dehydrogenase. Both enzyme complexes consist of three basic components; E1 (pyruvate dehydrogenase consisting of Pdha1 and Pdhb / 2-oxoglutarate dehydrogenase Ogdh), E2 (dihydrolipoyllysine-residue acetyltransferase Dlat / dihydrolipoyllysine-residue succinyltransferase Dlst) and E3, which is the same in both complexes (lipoamide dehydrogenase, Dld). Accordingly, Dld is found cross-linked to the E1 and E2 components of pyruvate dehydrogenase as well as to 2-oxoglutarate dehydrogenase. Moreover, we detect Pdhx, which anchors the pyruvate dehydrogenase E3 and E2 components. In agreement with the multimeric stoichiometry of these complexes, several homo-oligomeric cross-links were detected for Ogdh and Pdha1, evidenced by detected cross-links connecting homo-oligomeric subunits via the same lysine residue. (B) Interaction map of the MICOS complex. According to the current model, the MICOS complex consists of 7 subunits (2), 5 of which were captured in our XL-MS data. Moreover, our cross-links confirm previously described interactions of MICOS with the cristae structure-regulating GTPase Opa1 and with the SAM complex, which associates with MICOS to form the MIB complex (3, 4). (C, D) Cross-link mapping of Lyrm-Etfa/Etfb and Coq6-Coq8 interactions. The former interaction has been described to play an essential role in shuttling electrons to coenzyme Q while the latter belongs to the recently proposed coenzyme Q (CoQ) complex (5).

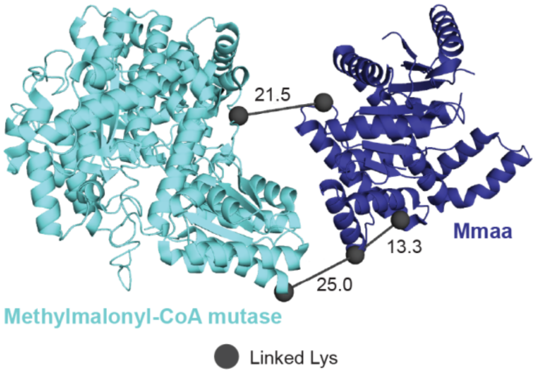

Supplementary Figure S5. Cross-link data confirms the mitochondrial localization of Mmaa. Although Mmaa (shown in dark blue) is not included in MitoCarta v2.0, its mitochondrial localization is confirmed by two cross-links to the methylmalonyl-CoA mutase enzyme (shown in cyan). Additionally, an intra-molecular cross-link within Mmaa could be verified. The proteins were manually oriented to illustrate that the distance limits of both inter-protein cross-links can be simultaneously satisfied.

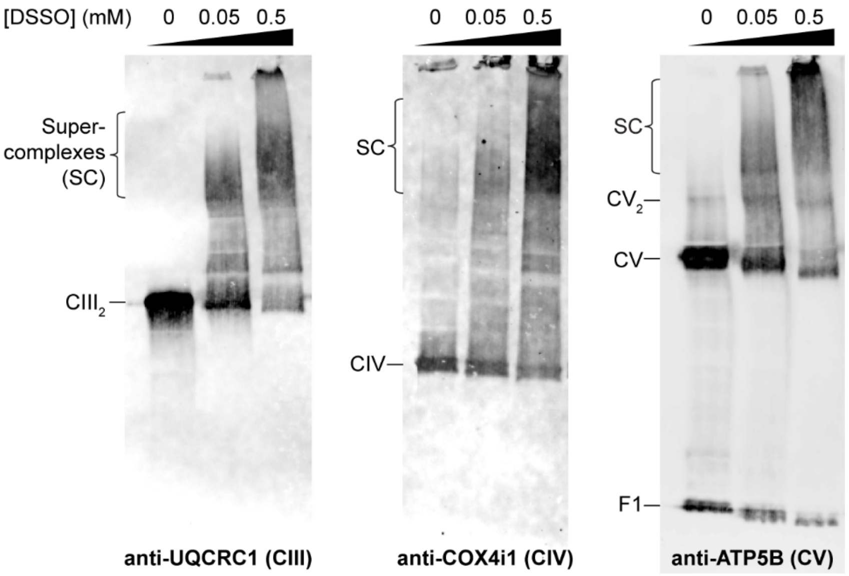

Supplementary Figure S6. Immunoblot analysis of oxidative phosphorylation complexes in response to cross-linking. Cross-linked mitochondria were solubilized with maltoside, and run on a clear native gel in the presence of triton and deoxycholate. Gels were soaked in SDS-containing buffer prior to transfer. Images were collected without pixel saturation, but displayed with linear adjustment to highlight lower intensity bands. In the absence of the DSSO cross-linker, CIII, CIV and CV appear as distinct bands. At the DSSO concentrations used for XL-MS analysis (0.5 mM DSSO, right lanes), high molecular weight species are detected with all three antibodies, indicating the stabilization of higher order structures.

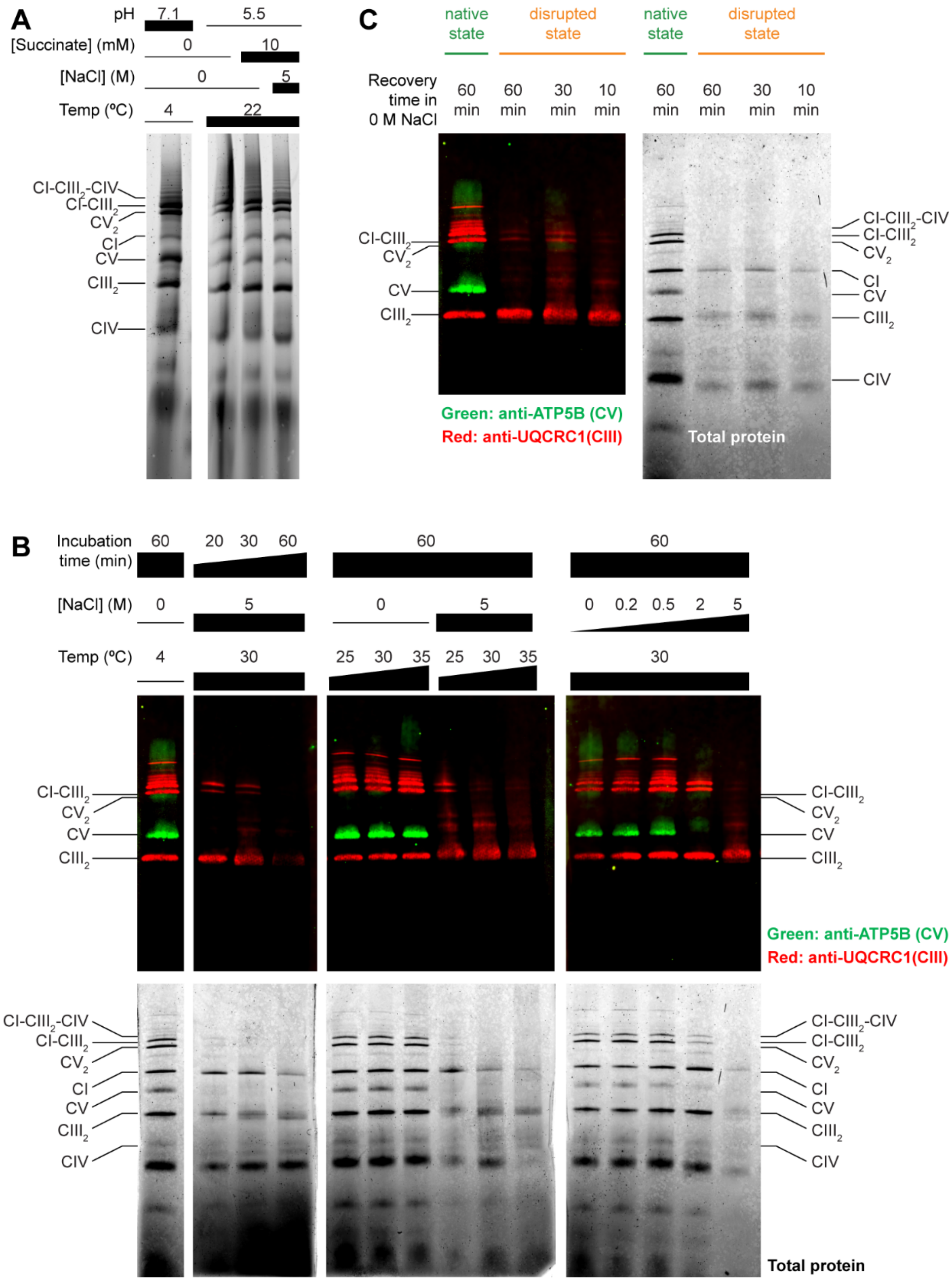

Supplementary Figure S7. Screening and optimizing supercomplex disruption methods. If not otherwise indicated, mitochondria were disrupted by incubation with 5M NaCl for 1 h at 30°C. All disrupted mitochondria were returned to original buffer prior to solubilization (to test for stable disruption of the supercomplexes). Cross-linked mitochondria were solubilized with digitonin and run on a clear native gel. Gels were soaked in SDS transfer buffer prior to blotting. Images were collected without pixel saturation, but displayed here with linear adjustment to

highlight lower intensity bands. Loading onto the gel was calculated based on Bradford assay prior to disruption. (A) Effect of succinate and low-pH treatment on supercomplexes. All lanes have 50 μg protein. (B) Optimization of salt-induced supercomplex disruption. All lanes have 20 μg protein. (C) Assessment of the stability of the disrupted state. All lanes have 20 μg protein. Disrupted mitochondria were washed with HEENK buffer only, and allowed to incubate at room temperature for the time indicated. The native state control sample (no salt treatment) had a parallel 60 min recovery time. This mimics the 60 min incubation at RT that would follow disruption/washing during the crosslinking reaction. Supercomplexes remained fully dissociated during this time.

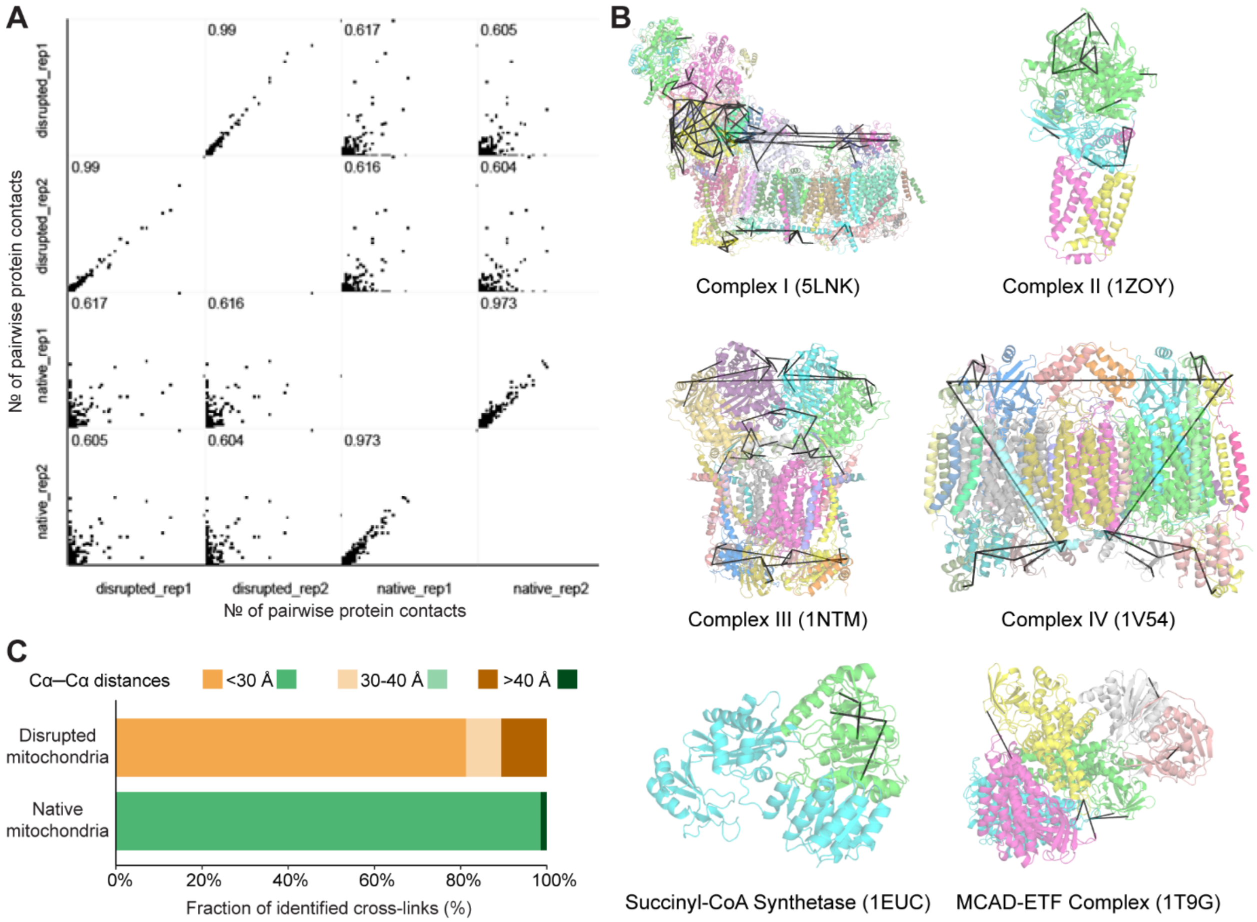

Supplementary Figure S8. Disruption-induced changes of mitochondrial protein contacts detected by XL-MS. (A) Multi scatter plot of the number of pairwise protein contacts between biological replicates of the same state (native vs. native and disrupted vs. disrupted) and different states (native vs. disrupted), corresponding to Figure 4C. (B) Mapping of the cross-links (black lines) detected in disrupted mitochondria onto high-resolution structures of selected mitochondrial protein complexes. The color scheme is the same as in Figure 2A. (C) The distributions of Cα–Cα distances in both untreated and disrupted mitochondria.

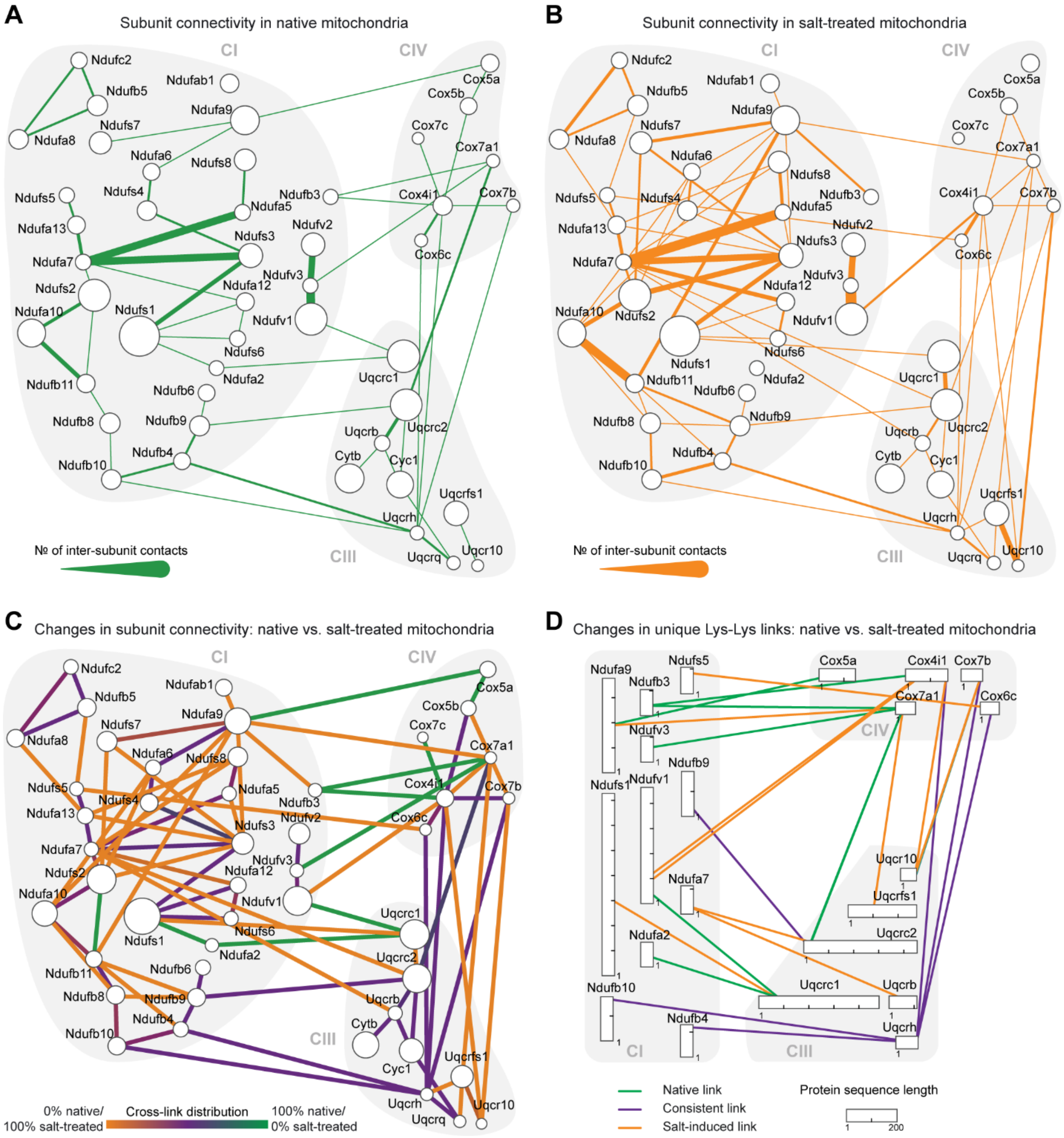

Supplementary Figure S9. Interaction pattern of CI, CIII and CIV in native and disrupted mitochondria. Shown are only subunits that are included in published structural models (PDB codes 5J4Z and 5LNK). (A-B) Inter-protein cross-links among structurally characterized subunits of CI, CIII and CIV in native (A) and disrupted (B) mitochondria. The line width indicates the number of cross-link sites between the connected subunits. (C) Changes of CI-CIII-CIV interactions upon mitochondrial disruption. The color code indicates which fraction of inter-subunit cross-links was detected in the native (gradient toward green) and the disrupted state (gradient toward orange), respectively. (D) Sequence map of cross-links connecting CI, CIII and

CIV. Note that the majority of cross-links is specific to either the native or the disrupted state. The sequence bars correspond to the protein size.

References accompanying the supplementary figures

1. Schweppe DK, Chavez JD, Lee CF, Caudal A, Kruse SE, Stuppard R, et al. Mitochondrial protein interactome elucidated by chemical cross-linking mass spectrometry. Proceedings of the National Academy of Sciences of the United States of America. 2017;114(7):1732-7.
2. van der Laan M, Horvath SE, Pfanner N. Mitochondrial contact site and cristae organizing system. Curr Opin Cell Biol. 2016;41:33-42.
3. Ding C, Wu Z, Huang L, Wang Y, Xue J, Chen S, et al. Mitofilin and CHCHD6 physically interact with Sam50 to sustain cristae structure. Sci Rep. 2015;5:16064.
4. Huynen MA, Muhlmeister M, Gotthardt K, Guerrero-Castillo S, Brandt U. Evolution and structural organization of the mitochondrial contact site (MICOS) complex and the mitochondrial intermembrane space bridging (MIB) complex. Biochim Biophys Acta. 2016;1863(1):91-101.
5. Floyd BJ, Wilkerson EM, Veling MT, Minogue CE, Xia C, Beebe ET, et al. Mitochondrial Protein Interaction Mapping Identifies Regulators of Respiratory Chain Function. Molecular cell. 2016;63(4):621-32.
