# Supplementary material for: The interactome of intact mitochondria by cross-linking mass spectrometry provides evidence for coexisting respiratory supercomplexes
Source: Mol Cell Proteomics. 2017 Dec 8;17(2):216–32. doi: 10.1074/mcp.RA117.000470 (PMC5795388; doi:10.1074/mcp.RA117.000470)
Supplement: Supplemental Data [file supp_RA117.000470_133922_0_supp_23978_fzffwf.zip › spectra_annotation/mito_DR_spectra_annotation/100-1-9-1-18-1.pdf]

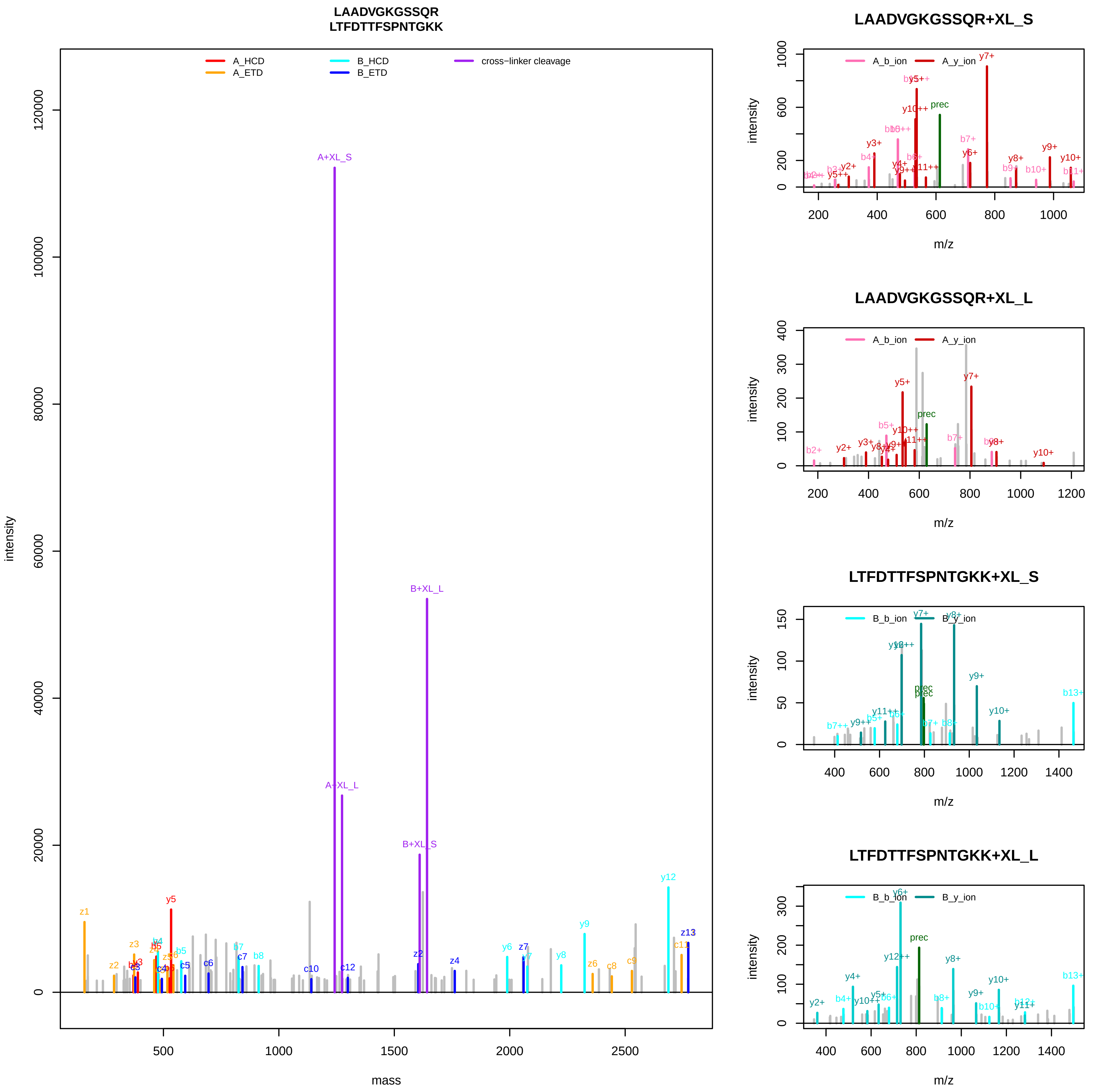

LAADVGKGSSQR
LTFDTTFSPNTGKK
A_HCD
A_ETD
B_HCD
B_ETD
cross−linker cleavage
A+XL_S
B+XL_L
A+XL_L
B+XL_S
intensity
mass
0
20000
40000
60000
80000
100000
120000
500
1000
1500
2000
2500
z1
z2
z3
y5
y12
z13
c11
y9
y8
y6
z7
z6
c8
c9
c10
c12
z2
z4
c6
c7
b7
b8
b5
c5
c4
c3
LAADVGKGSSQR+XL_S
A_b_ion
A_y_ion
y7+
prec
y10++
y3+
y9+
y8+
y10+
y6+
b7+
b4+
y2+
y5++
intensity
m/z
0
200
600
1000
200
400
600
800
1000
LAADVGKGSSQR+XL_L
A_b_ion
A_y_ion
y5+
y7+
prec
b5+
y10++
y3+
y2+
b2+
b7+
y8+
y10+
intensity
m/z
0
100
200
300
400
200
400
600
800
1000
1200
LTFDTTFSPNTGKK+XL_S
B_b_ion
B_y_ion
y7+
y8+
y12++
prec
y9+
y10+
b13+
y11++
y9++
b7++
intensity
m/z
0
50
100
150
400
600
800
1000
1200
1400
LTFDTTFSPNTGKK+XL_L
B_b_ion
B_y_ion
y6+
prec
y12++
y8+
y4+
y10+
b13+
y9+
y2+
b4+
y10++
b8+
y11+
intensity
m/z
0
100
200
300
400
600
800
1000
1200
1400
