# Supplementary material for: The interactome of intact mitochondria by cross-linking mass spectrometry provides evidence for coexisting respiratory supercomplexes
Source: Mol Cell Proteomics. 2017 Dec 8;17(2):216–32. doi: 10.1074/mcp.RA117.000470 (PMC5795388; doi:10.1074/mcp.RA117.000470)
Supplement: Supplemental Data [file supp_RA117.000470_133922_0_supp_23978_fzffwf.zip › spectra_annotation/mito_DR_spectra_annotation/105-1-10-1-14-1.pdf]

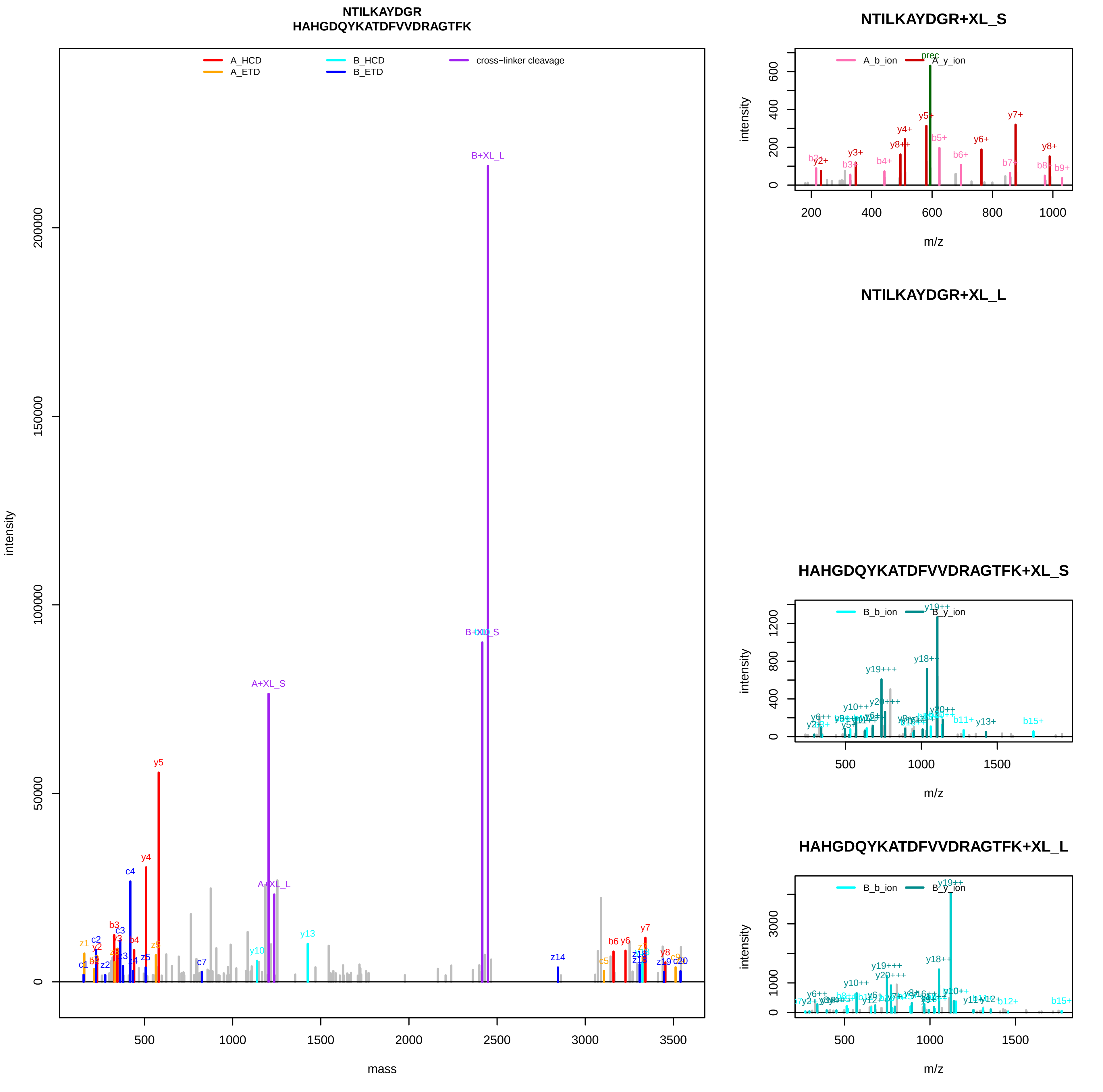

NTILKAYDGR
HAHGDQYKATDFVVDRAGTFK
A_HCD
A_ETD
B_HCD
B_ETD
cross−linker cleavage
B+XL_L
B+XL_S
A+XL_S
A+XL_L
y5
y4
c4
b3
c3
b4
y13
y10
c7
z14
b6
y6
y7
y8
c20
intensity
mass
0
50000
100000
150000
200000
500
1000
1500
2000
2500
3000
3500
NTILKAYDGR+XL_S
A_b_ion
A_y_ion
prec
m/z
NTILKAYDGR+XL_L
HAHGDQYKATDFVVDRAGTFK+XL_S
B_b_ion
B_y_ion
y19++
y18++
y19+++
y20+++
y20++
b11+
y13+
b15+
HAHGDQYKATDFVVDRAGTFK+XL_L
y18++
y19+++
y20+++
y10++
y10++
b12+
b15+
