# Supplementary material for: The interactome of intact mitochondria by cross-linking mass spectrometry provides evidence for coexisting respiratory supercomplexes
Source: Mol Cell Proteomics. 2017 Dec 8;17(2):216–32. doi: 10.1074/mcp.RA117.000470 (PMC5795388; doi:10.1074/mcp.RA117.000470)
Supplement: Supplemental Data [file supp_RA117.000470_133922_0_supp_23978_fzffwf.zip › spectra_annotation/mito_DR_spectra_annotation/107-1-3-1-13-1.pdf]

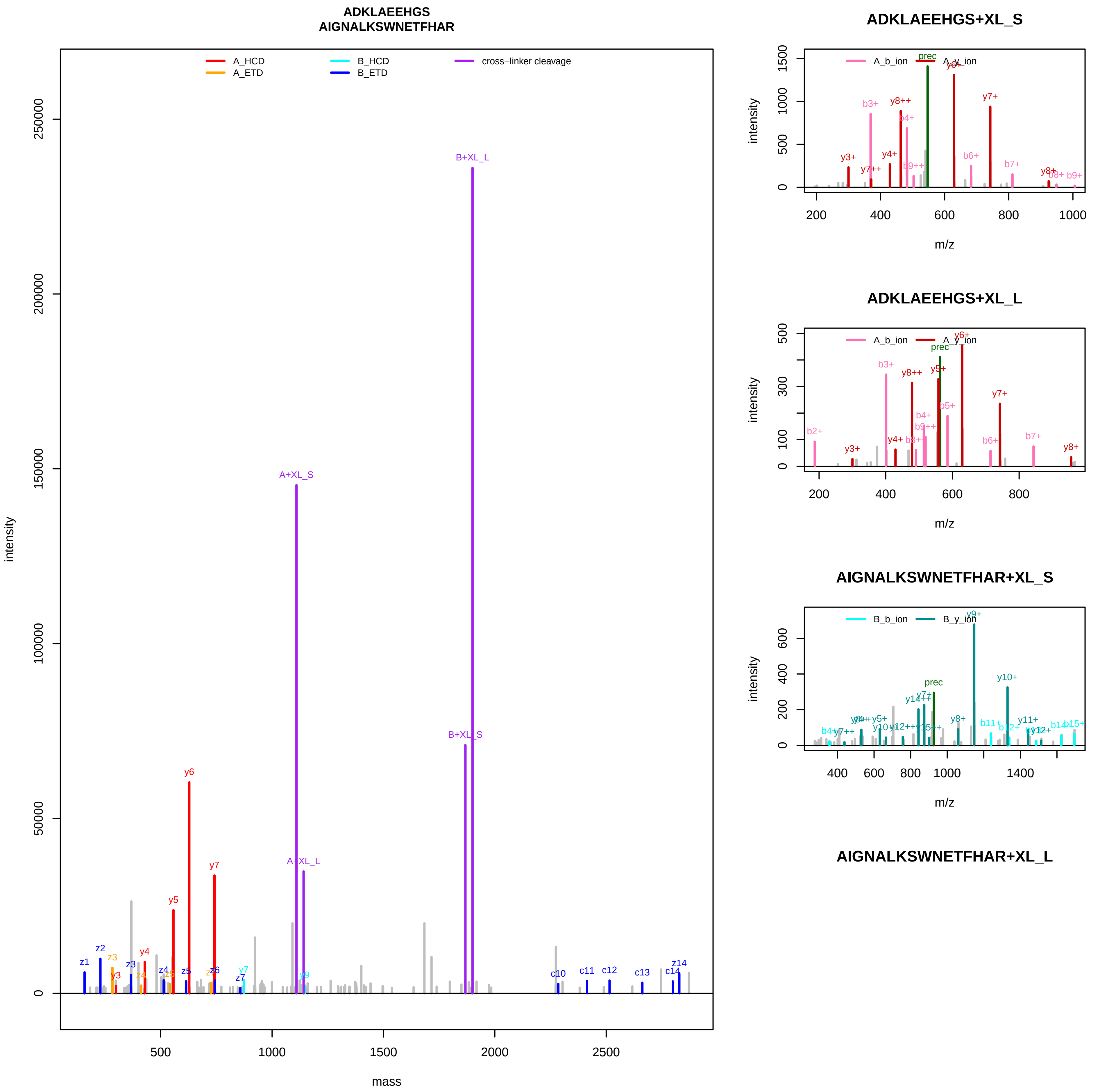

ADKLAEEHGS
AIGNALKSWNETFHAR
A_HCD
A_ETD
B_HCD
B_ETD
cross−linker cleavage
intensity
mass
B+XL_L
A+XL_S
B+XL_S
A+XL_L
y6
y7
y5
y4
y3
z1
z2
z3
z4
z5
z6
z7
y7
y9
c10
c11
c12
c13
c14
z14
ADKLAEEHGS+XL_S
A_b_ion
A_y_ion
prec
m/z
ADKLAEEHGS+XL_L
AIGNALKSWNETFHAR+XL_S
B_b_ion
B_y_ion
y9+
y10+
AIGNALKSWNETFHAR+XL_L
