# Supplementary material for: The interactome of intact mitochondria by cross-linking mass spectrometry provides evidence for coexisting respiratory supercomplexes
Source: Mol Cell Proteomics. 2017 Dec 8;17(2):216–32. doi: 10.1074/mcp.RA117.000470 (PMC5795388; doi:10.1074/mcp.RA117.000470)
Supplement: Supplemental Data [file supp_RA117.000470_133922_0_supp_23978_fzffwf.zip › spectra_annotation/mito_DR_spectra_annotation/107-1-9-1-1-1.pdf]

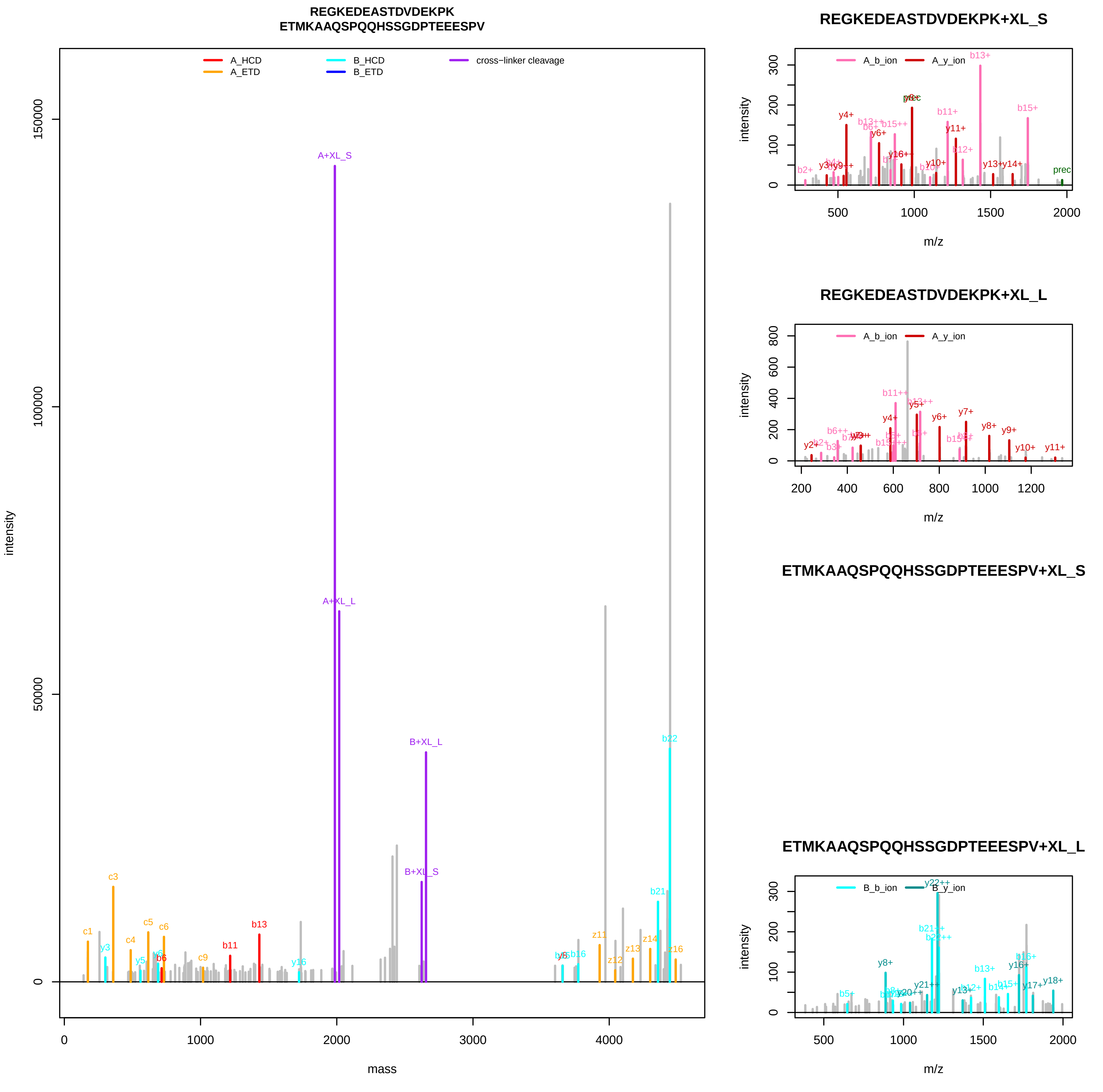

REGKEDEASTDVDEKPK
ETMKAAQSPQQHSSGDPTEEESPV
A_HCD
A_ETD
B_HCD
B_ETD
cross−linker cleavage
intensity
mass
A+XL_S
A+XL_L
B+XL_L
B+XL_S
b22
b21
b13
b11
b6
c1
c3
c4
c5
c6
c9
y3
y5
y16
z11
z12
z13
z14
z16
REGKEDEASTDVDEKPK+XL_S
REGKEDEASTDVDEKPK+XL_L
ETMKAAQSPQQHSSGDPTEEESPV+XL_S
ETMKAAQSPQQHSSGDPTEEESPV+XL_L
A_b_ion
A_y_ion
B_b_ion
B_y_ion
m/z
b13+
b15+
b11+
y4+
y11+
y10+
y13+
y14+
prec
