# Supplementary material for: The interactome of intact mitochondria by cross-linking mass spectrometry provides evidence for coexisting respiratory supercomplexes
Source: Mol Cell Proteomics. 2017 Dec 8;17(2):216–32. doi: 10.1074/mcp.RA117.000470 (PMC5795388; doi:10.1074/mcp.RA117.000470)
Supplement: Supplemental Data [file supp_RA117.000470_133922_0_supp_23978_fzffwf.zip › spectra_annotation/mito_DR_spectra_annotation/108-1-19-1-47-1.pdf]

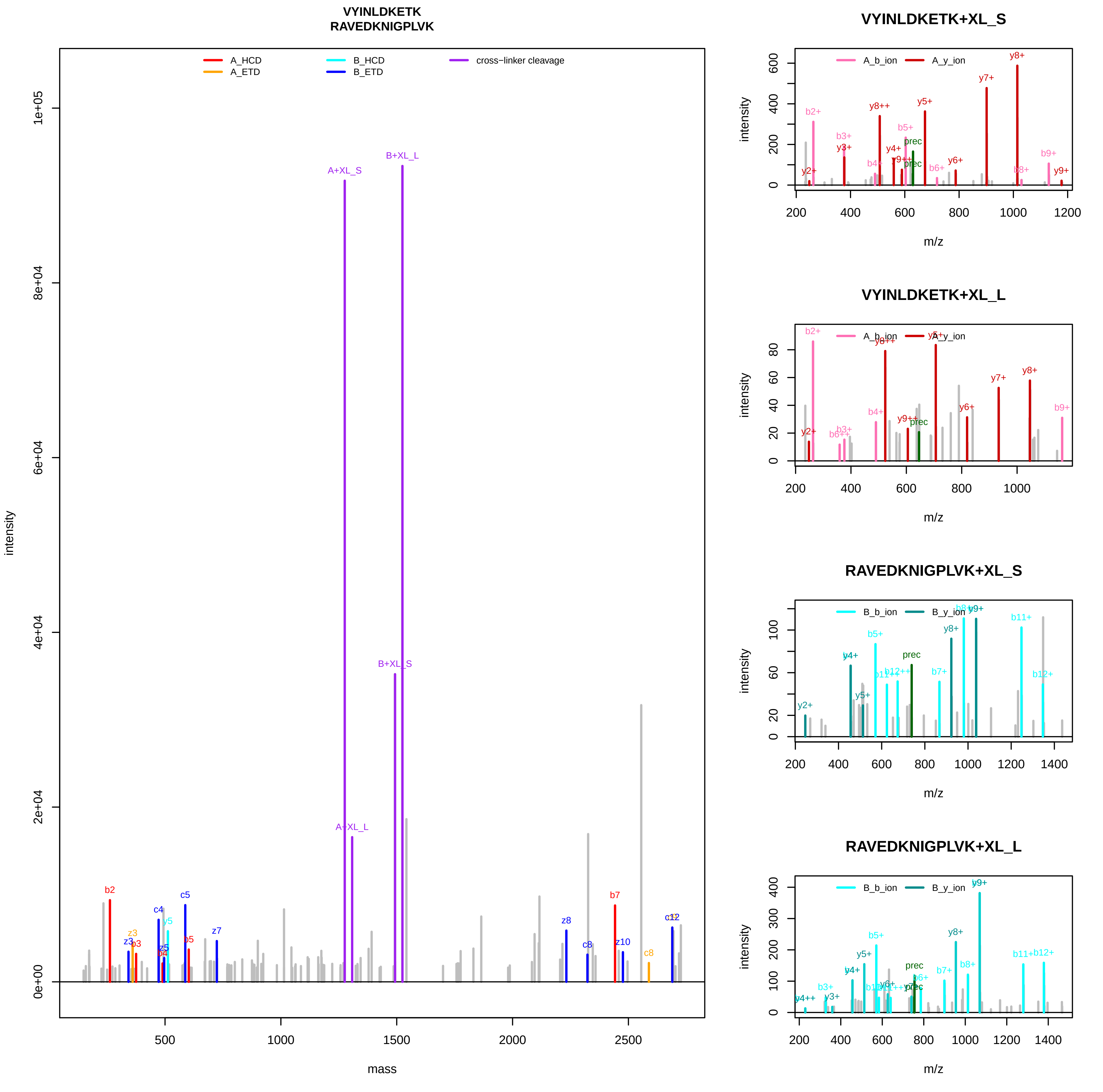

VYINLDKETK
RAVEDKNIGPLVK
A_HCD
A_ETD
B_HCD
B_ETD
cross−linker cleavage
A+XL_S
B+XL_L
B+XL_S
A+XL_L
b2
z3
b3
c4
y5
z5
b4
c5
b5
z7
z8
c8
b7
z10
c8
c12
1e+05
8e+04
6e+04
4e+04
2e+04
0e+00
intensity
500
1000
1500
2000
2500
mass
VYINLDKETK+XL_S
A_b_ion
A_y_ion
m/z
VYINLDKETK+XL_L
RAVEDKNIGPLVK+XL_S
B_b_ion
B_y_ion
RAVEDKNIGPLVK+XL_L
