# Supplementary material for: The interactome of intact mitochondria by cross-linking mass spectrometry provides evidence for coexisting respiratory supercomplexes
Source: Mol Cell Proteomics. 2017 Dec 8;17(2):216–32. doi: 10.1074/mcp.RA117.000470 (PMC5795388; doi:10.1074/mcp.RA117.000470)
Supplement: Supplemental Data [file supp_RA117.000470_133922_0_supp_23978_fzffwf.zip › spectra_annotation/mito_DR_spectra_annotation/112-1-2-1-7-1.pdf]

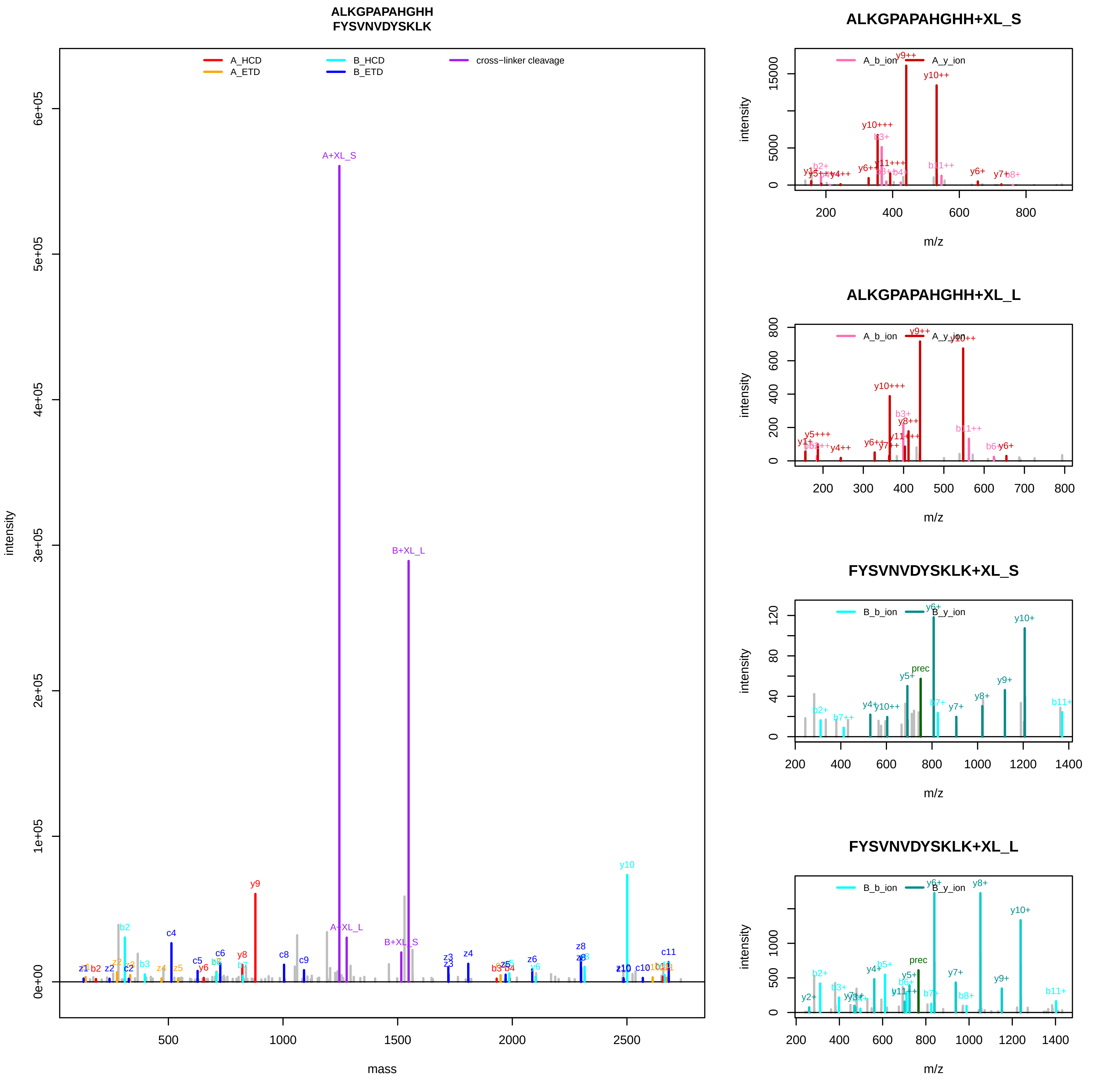
ALKGPAPAHGHH
FYSVNVDYSKLK
A_HCD
A_ETD
B_HCD
B_ETD
cross−linker cleavage
A+XL_S
B+XL_L
A+XL_L
B+XL_S
y9
y10
c4
c11
z4
intensity
mass
ALKGPAPAHGHH+XL_S
A_b_ion
A_y_ion
y9++
y10++
y10+++
b3+
b11++
m/z
ALKGPAPAHGHH+XL_L
A_b_ion
A_y_ion
y9++
y10++
y10+++
b3+
y8++
b11++
m/z
FYSVNVDYSKLK+XL_S
B_b_ion
B_y_ion
y6+
y10+
prec
y5+
y9+
y8+
y7+
b7+
b2+
b11+
m/z
FYSVNVDYSKLK+XL_L
B_b_ion
B_y_ion
y6+
y8+
y10+
prec
b5+
y4+
y7+
y9+
b2+
b11+
m/z
