# Supplementary material for: The interactome of intact mitochondria by cross-linking mass spectrometry provides evidence for coexisting respiratory supercomplexes
Source: Mol Cell Proteomics. 2017 Dec 8;17(2):216–32. doi: 10.1074/mcp.RA117.000470 (PMC5795388; doi:10.1074/mcp.RA117.000470)
Supplement: Supplemental Data [file supp_RA117.000470_133922_0_supp_23978_fzffwf.zip › spectra_annotation/mito_DR_spectra_annotation/117-1-10-1-119-1.pdf]

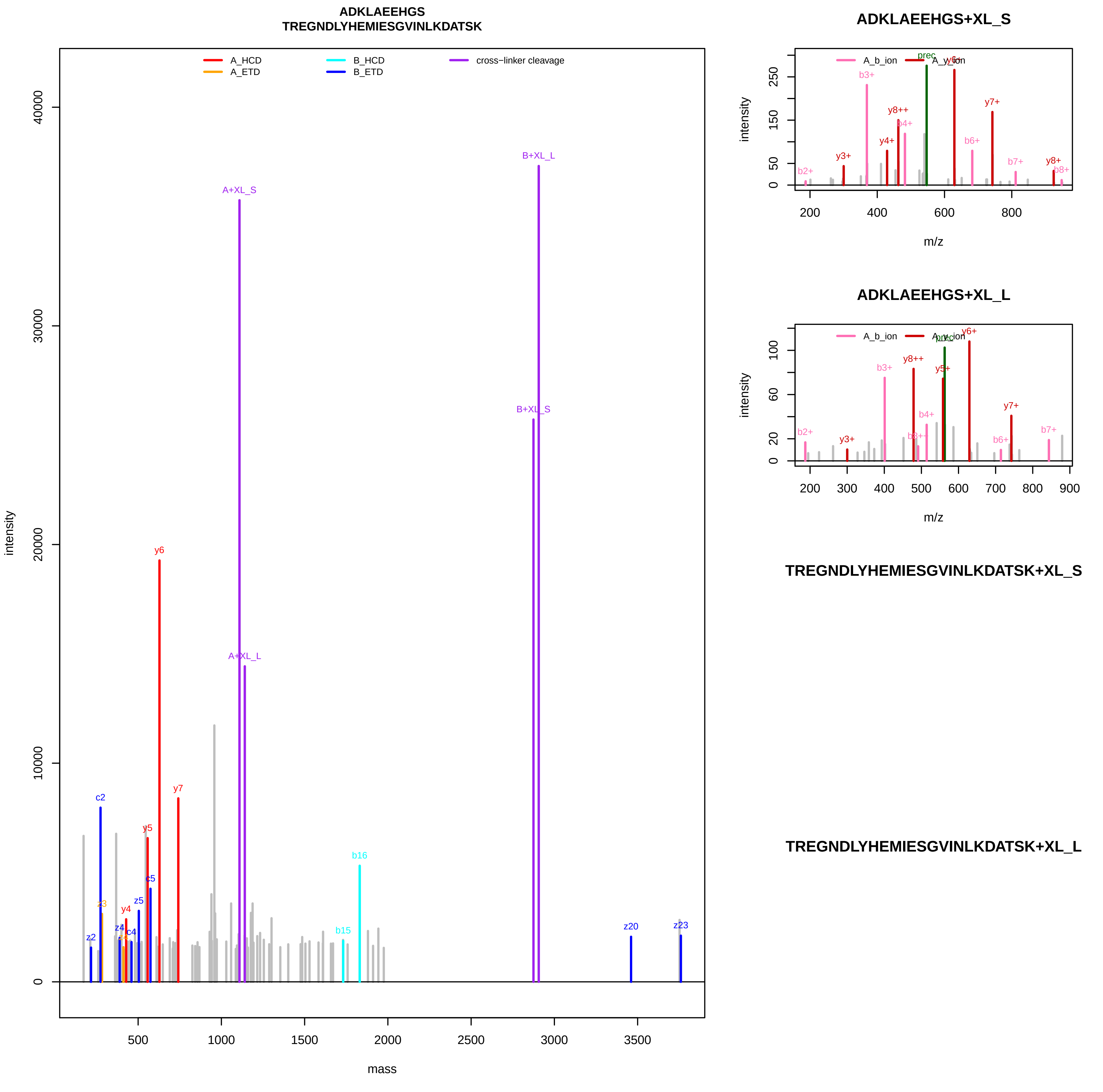

ADKLAEEHGS
TREGNDLYHEMIESGVINLKDATSK
A_HCD
A_ETD
B_HCD
B_ETD
cross−linker cleavage
intensity
mass
B+XL_L
A+XL_S
B+XL_S
A+XL_L
y6
y7
c2
y5
c5
b16
z3
y4
z5
z4
c4
z2
b15
z20
z23
ADKLAEEHGS+XL_S
A_b_ion
A_y_ion
prec
b3+
y8++
b4+
y4+
y7+
y6+
b6+
y3+
b7+
y8+
b2+
b8+
m/z
ADKLAEEHGS+XL_L
y5+
TREGNDLYHEMIESGVINLKDATSK+XL_S
TREGNDLYHEMIESGVINLKDATSK+XL_L
