# Supplementary material for: The interactome of intact mitochondria by cross-linking mass spectrometry provides evidence for coexisting respiratory supercomplexes
Source: Mol Cell Proteomics. 2017 Dec 8;17(2):216–32. doi: 10.1074/mcp.RA117.000470 (PMC5795388; doi:10.1074/mcp.RA117.000470)
Supplement: Supplemental Data [file supp_RA117.000470_133922_0_supp_23978_fzffwf.zip › spectra_annotation/mito_DR_spectra_annotation/120-1-14-1-15-1.pdf]

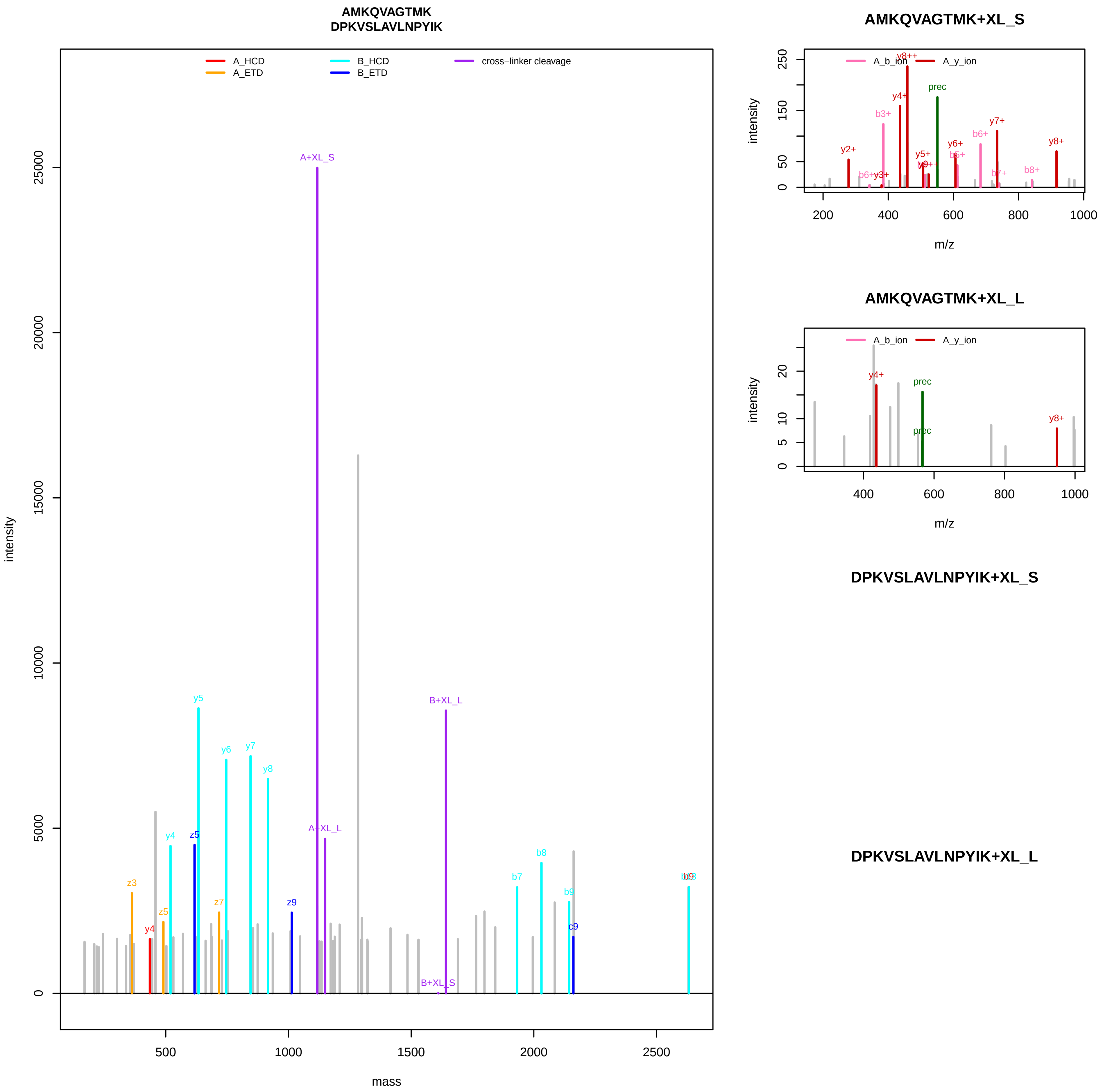
AMKQVAGTMK
DPKVSLAVLNPYIK
A_HCD
A_ETD
B_HCD
B_ETD
cross−linker cleavage
A+XL_S
A+XL_L
B+XL_L
B+XL_S
y4
y5
y6
y7
y8
z3
z5
z7
z9
c9
b7
b8
b9
intensity
mass
AMKQVAGTMK+XL_S
A_b_ion
A_y_ion
prec
y2+
y3+
y4+
y5+
y6+
y7+
y8+
y8++
y9++
b3+
b5+
b6+
b6++
b7+
b8+
m/z
AMKQVAGTMK+XL_L
DPKVSLAVLNPYIK+XL_S
DPKVSLAVLNPYIK+XL_L
