# Supplementary material for: The interactome of intact mitochondria by cross-linking mass spectrometry provides evidence for coexisting respiratory supercomplexes
Source: Mol Cell Proteomics. 2017 Dec 8;17(2):216–32. doi: 10.1074/mcp.RA117.000470 (PMC5795388; doi:10.1074/mcp.RA117.000470)
Supplement: Supplemental Data [file supp_RA117.000470_133922_0_supp_23978_fzffwf.zip › spectra_annotation/mito_DR_spectra_annotation/122-1-25-1-118-1.pdf]

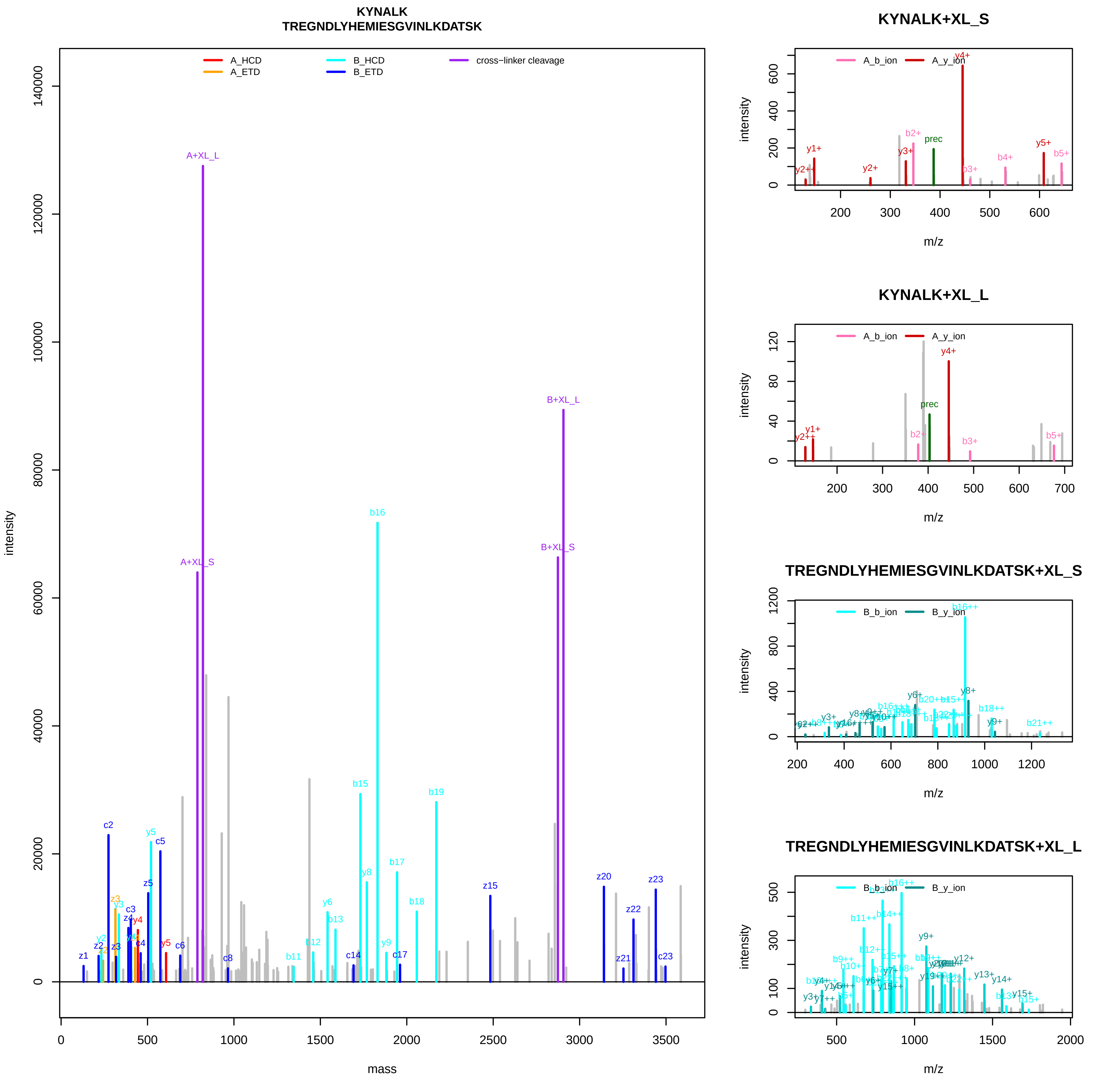

KYNALK
TREGNDLYHEMIESGVINLKDATSK
A_HCD
A_ETD
B_HCD
B_ETD
cross−linker cleavage
A+XL_L
A+XL_S
B+XL_L
B+XL_S
b16
b15
b19
b17
b18
y8
y6
b13
b12
b11
y9
c14
c17
c8
z15
z20
z21
z22
z23
c23
c2
c5
y5
z5
c3
z4
c4
y4
z3
y3
y2
z2
z1
c6
intensity
mass
0
20000
40000
60000
80000
100000
120000
140000
500
1000
1500
2000
2500
3000
3500
KYNALK+XL_S
A_b_ion
A_y_ion
y4+
y1+
y2+
b2+
prec
y3+
b3+
b4+
y5+
b5+
m/z
KYNALK+XL_L
TREGNDLYHEMIESGVINLKDATSK+XL_S
B_b_ion
B_y_ion
b16++
y8+
y6+
b18++
y9+
b21++
y3+
TREGNDLYHEMIESGVINLKDATSK+XL_L
y9+
y12+
y13+
y14+
y15+
b14++
b11++
b12++
b9++
b10++
