# Supplementary material for: The interactome of intact mitochondria by cross-linking mass spectrometry provides evidence for coexisting respiratory supercomplexes
Source: Mol Cell Proteomics. 2017 Dec 8;17(2):216–32. doi: 10.1074/mcp.RA117.000470 (PMC5795388; doi:10.1074/mcp.RA117.000470)
Supplement: Supplemental Data [file supp_RA117.000470_133922_0_supp_23978_fzffwf.zip › spectra_annotation/mito_DR_spectra_annotation/13-1-23-1-1-1.pdf]

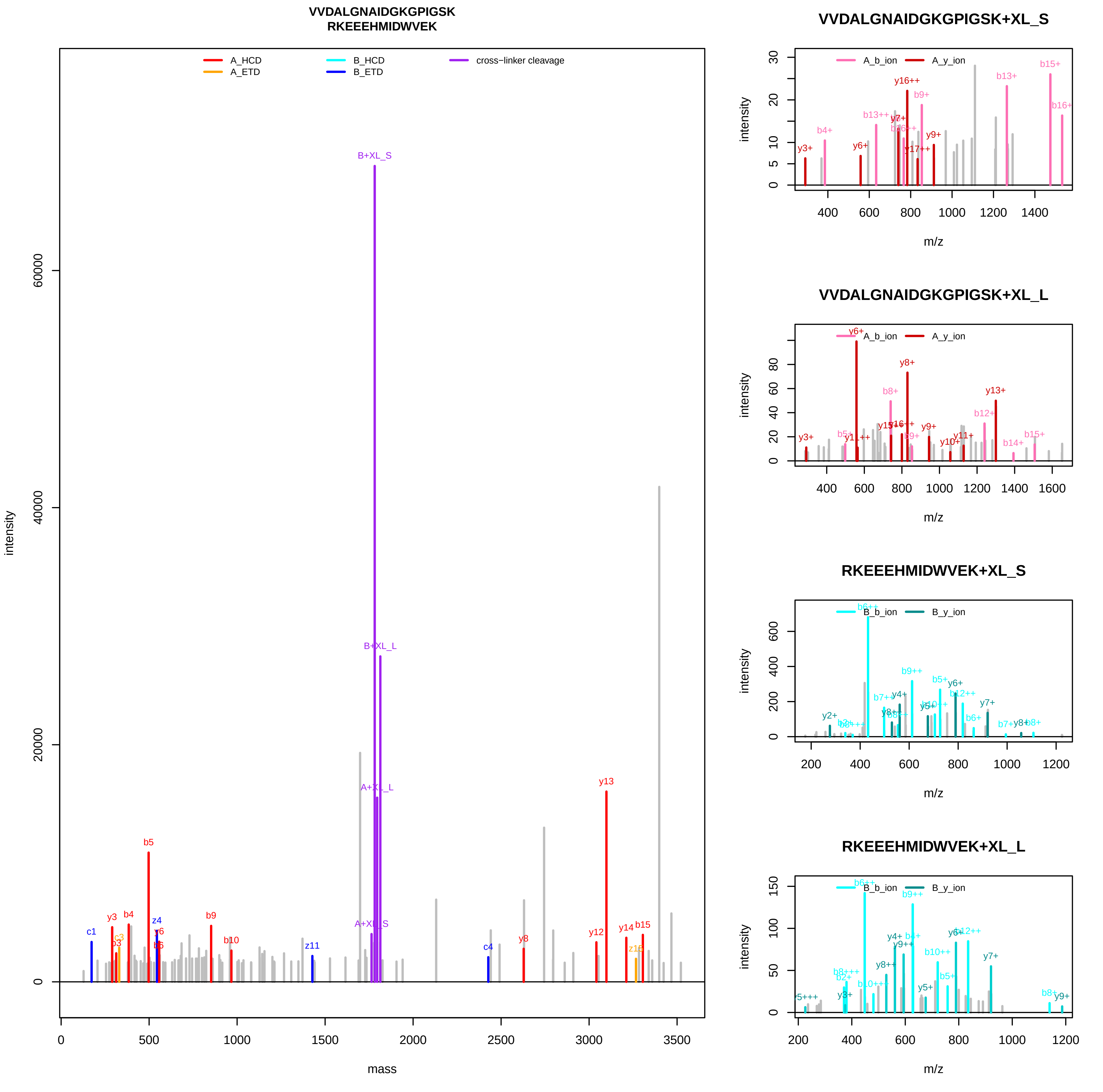

VVDALGNAIDGKGPIGSK
RKEEEHMIDWVEK
A_HCD
A_ETD
B_HCD
B_ETD
cross−linker cleavage
B+XL_S
B+XL_L
A+XL_L
A+XL_S
b5
y13
y3
b4
b9
b10
y12
y14
b15
c1
z4
z11
c4
y8
z16
intensity
mass
VVDALGNAIDGKGPIGSK+XL_S
A_b_ion
A_y_ion
b15+
b13+
y16++
b9+
b16+
b13++
b4+
y9+
y3+
y6+
intensity
m/z
VVDALGNAIDGKGPIGSK+XL_L
y6+
y8+
b8+
y13+
b12+
y3+
y9+
b14+
b15+
intensity
m/z
RKEEEHMIDWVEK+XL_S
B_b_ion
B_y_ion
b6++
b9++
b5+
y6+
y4+
y2+
y7+
intensity
m/z
RKEEEHMIDWVEK+XL_L
B_b_ion
B_y_ion
b6++
b9++
y4+
y8+
y7+
y3+
y5+
b8+
y9+
intensity
m/z
