# Supplementary material for: The interactome of intact mitochondria by cross-linking mass spectrometry provides evidence for coexisting respiratory supercomplexes
Source: Mol Cell Proteomics. 2017 Dec 8;17(2):216–32. doi: 10.1074/mcp.RA117.000470 (PMC5795388; doi:10.1074/mcp.RA117.000470)
Supplement: Supplemental Data [file supp_RA117.000470_133922_0_supp_23978_fzffwf.zip › spectra_annotation/mito_DR_spectra_annotation/13-1-7-1-8-1.pdf]

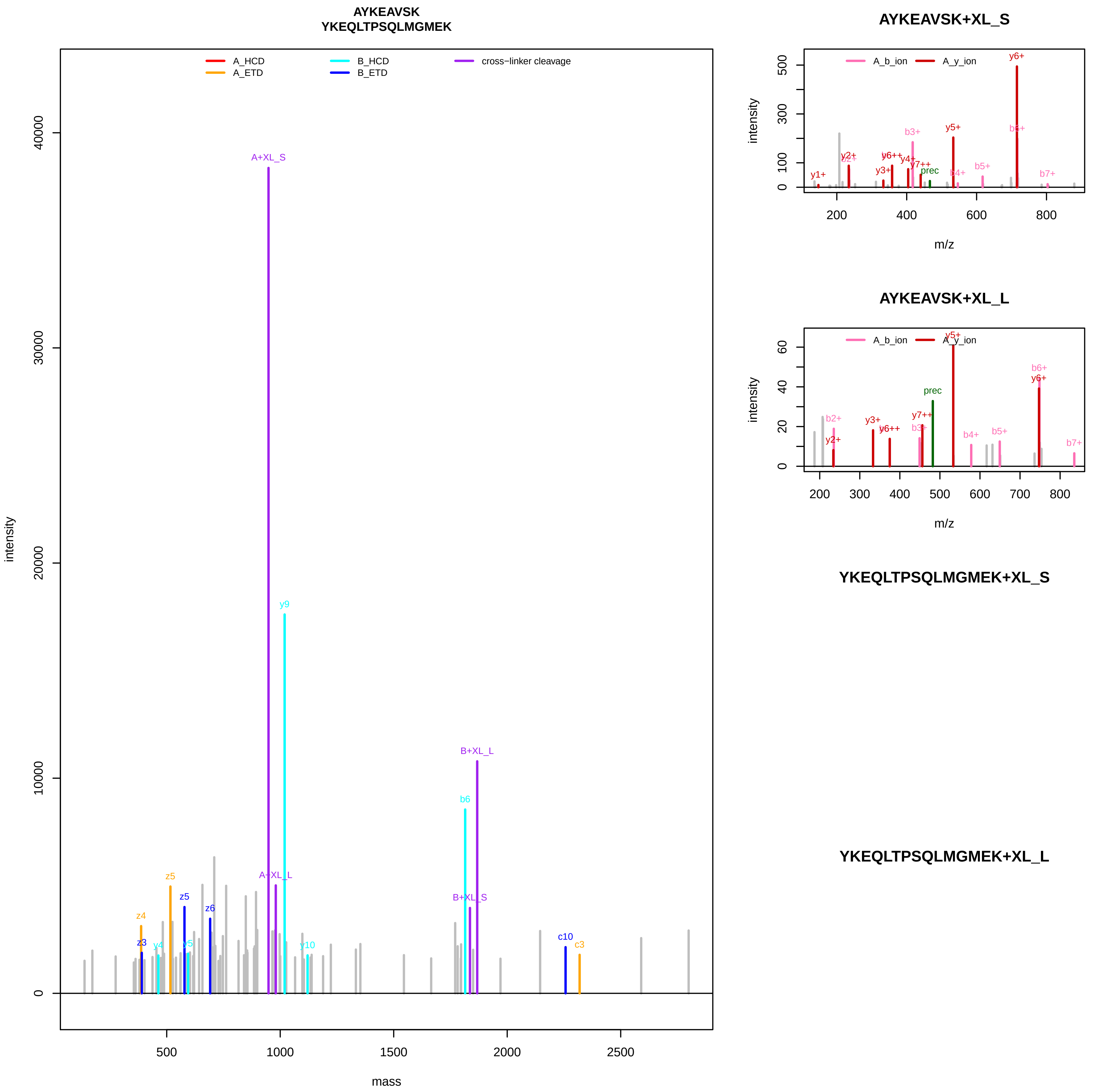

AYKEAVSK
YKEQLTPSQLMGMEK
A_HCD
A_ETD
B_HCD
B_ETD
cross−linker cleavage
A+XL_S
y9
B+XL_L
b6
A+XL_L
B+XL_S
z5
z4
z3
y4
y5
z6
y10
c10
c3
intensity
mass
AYKEAVSK+XL_S
A_b_ion
A_y_ion
y6+
b3+
y5+
b6+
y2+
b2+
y6++
y4+
y7++
y3+
prec
y1+
b4+
b5+
b7+
m/z
AYKEAVSK+XL_L
A_b_ion
A_y_ion
y5+
b6+
y6+
prec
b2+
y3+
y7++
y6++
b3+
y2+
b4+
b5+
b7+
m/z
YKEQLTPSQLMGMEK+XL_S
YKEQLTPSQLMGMEK+XL_L
