# Supplementary figures and images for: The interactome of intact mitochondria by cross-linking mass spectrometry provides evidence for coexisting respiratory supercomplexes (part 2 of 2)
Source: Mol Cell Proteomics. 2017 Dec 8;17(2):216–32. doi: 10.1074/mcp.RA117.000470 (PMC5795388; doi:10.1074/mcp.RA117.000470)

KWYYNAAGFNK  
HQILPKDQWTK

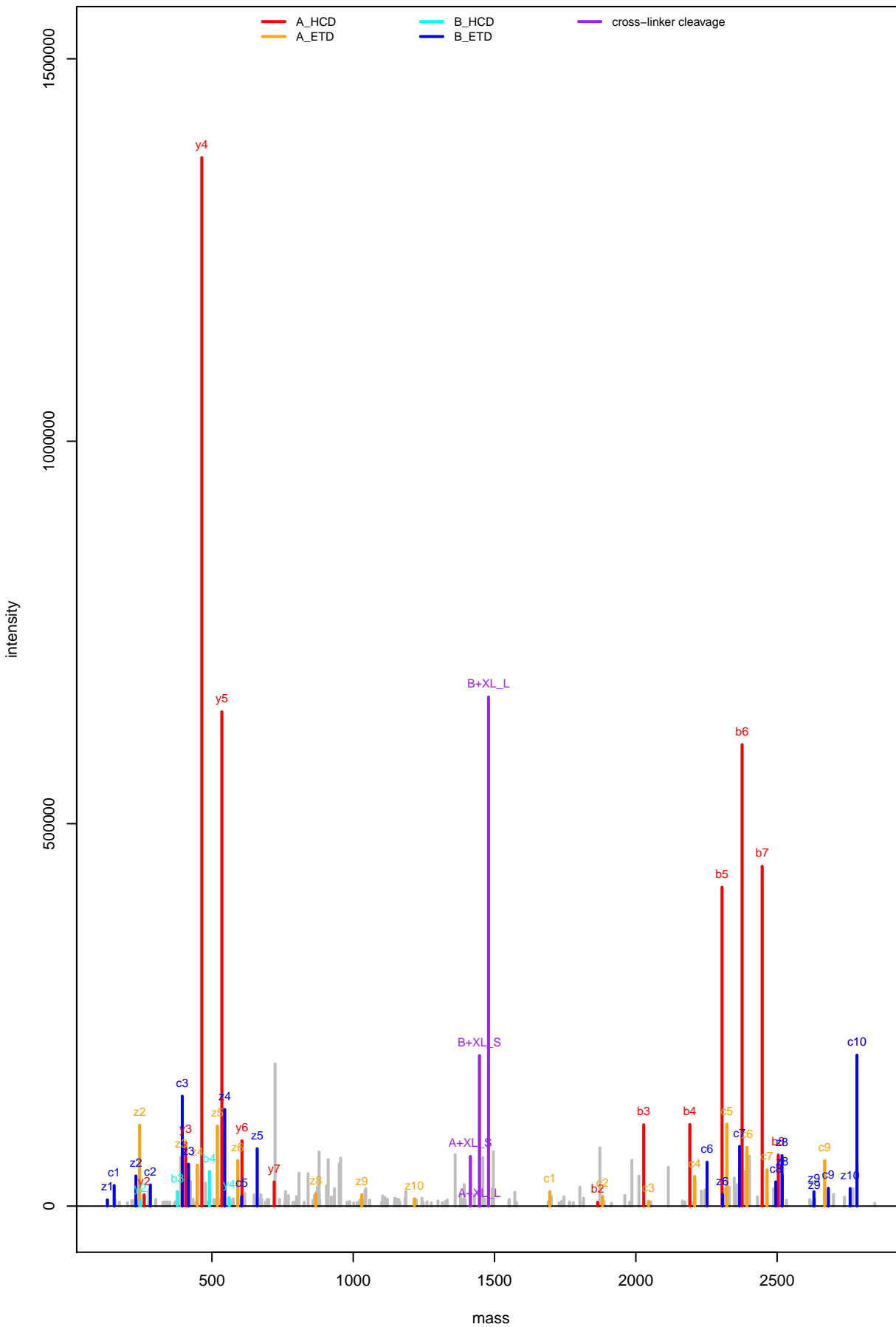

KWYYNAAGFNK+XL\_S

KWYYNAAGFNK+XL\_L

HQILPKDQWTK+XL\_S

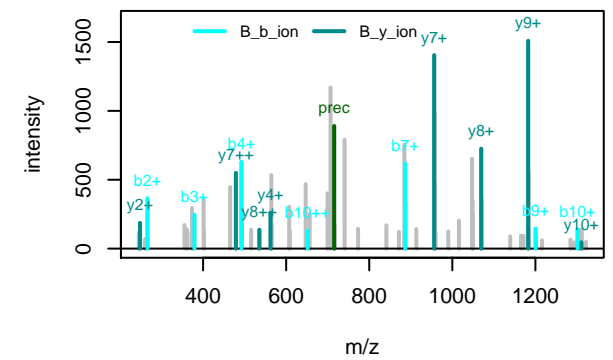

HQILPKDQWTK+XL\_L

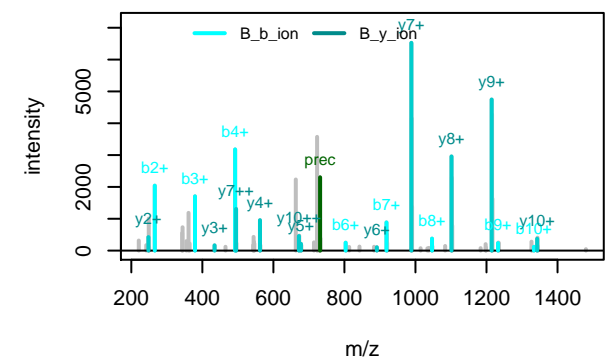

Supplement: Supplemental Data [file supp_RA117.000470_133922_0_supp_23978_fzffwf.zip › spectra_annotation/mito_DR_spectra_annotation/128-1-6-1-7-1.pdf]

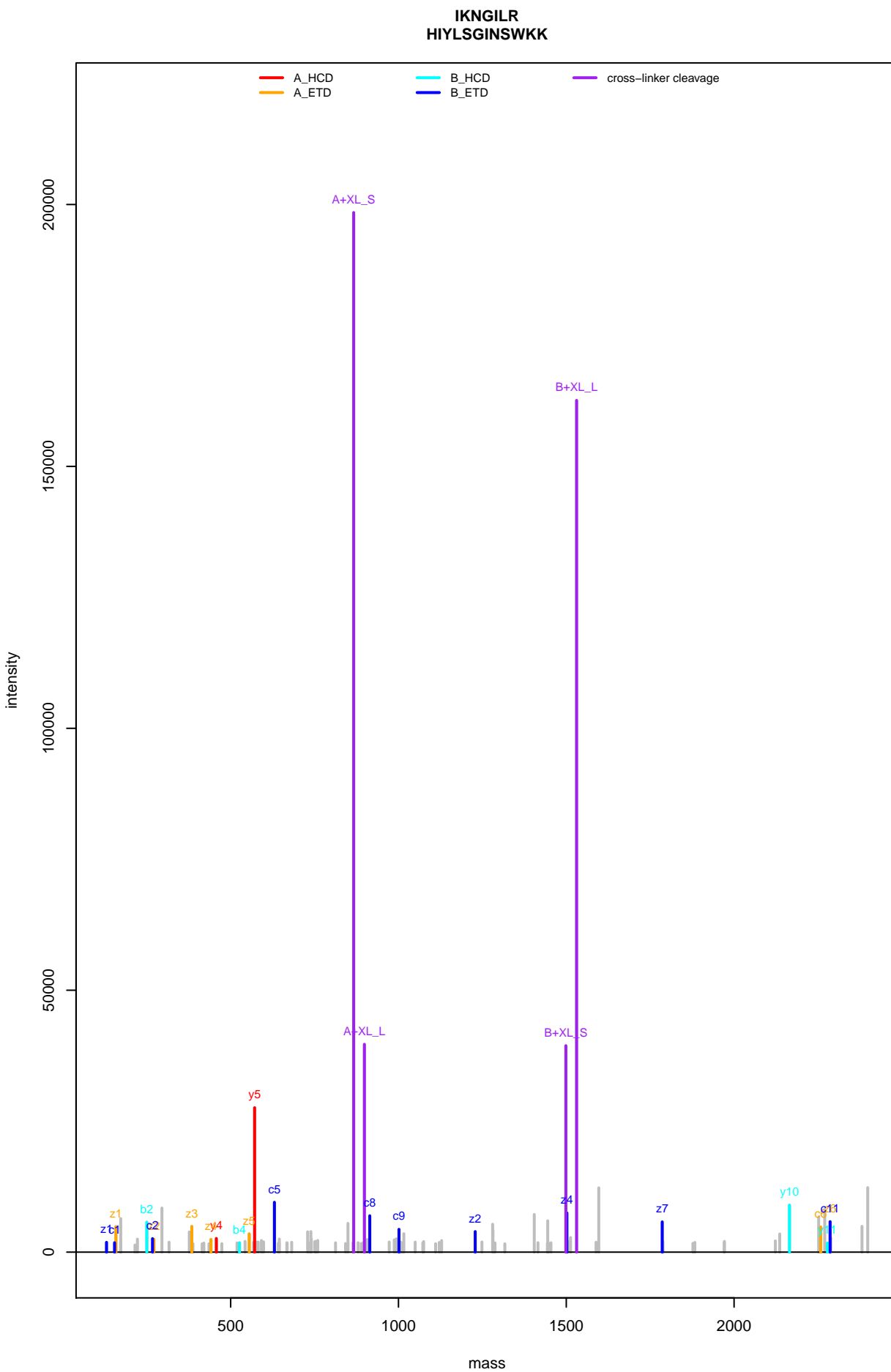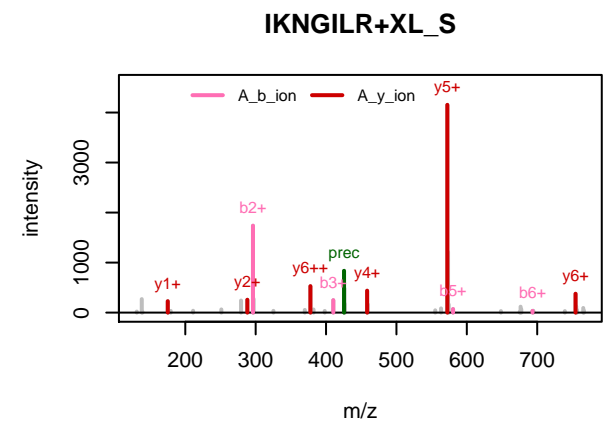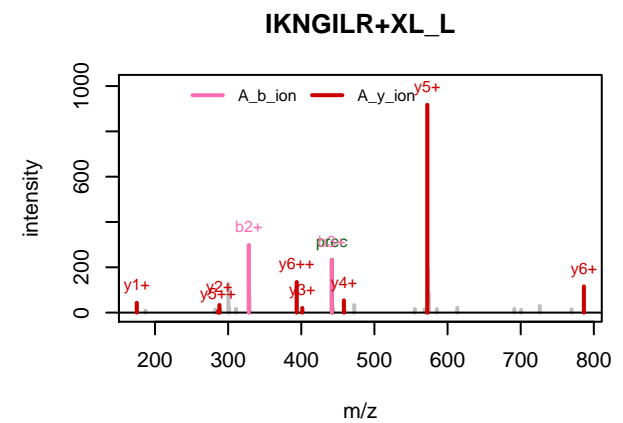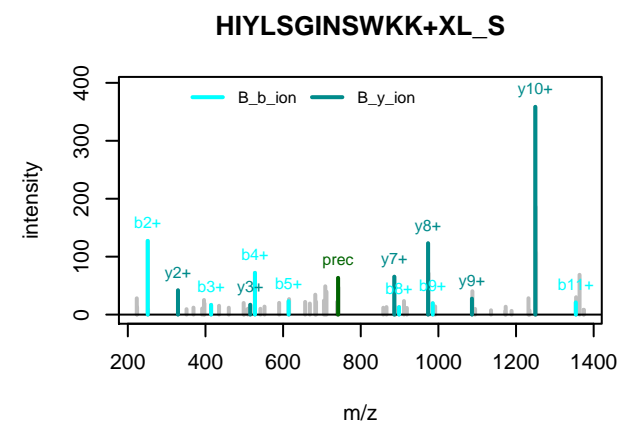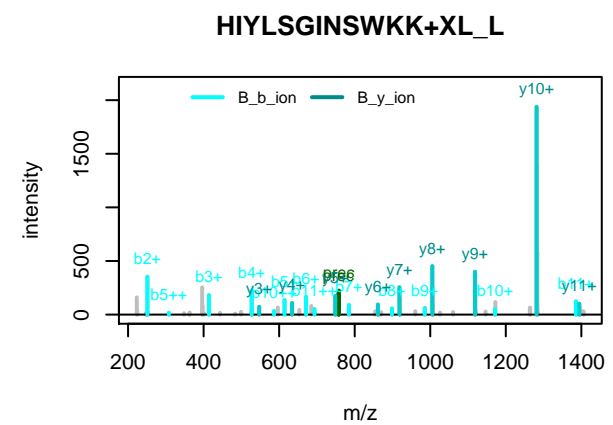

Supplement: Supplemental Data [file supp_RA117.000470_133922_0_supp_23978_fzffwf.zip › spectra_annotation/mito_DR_spectra_annotation/128-1-8-1-23-1.pdf]

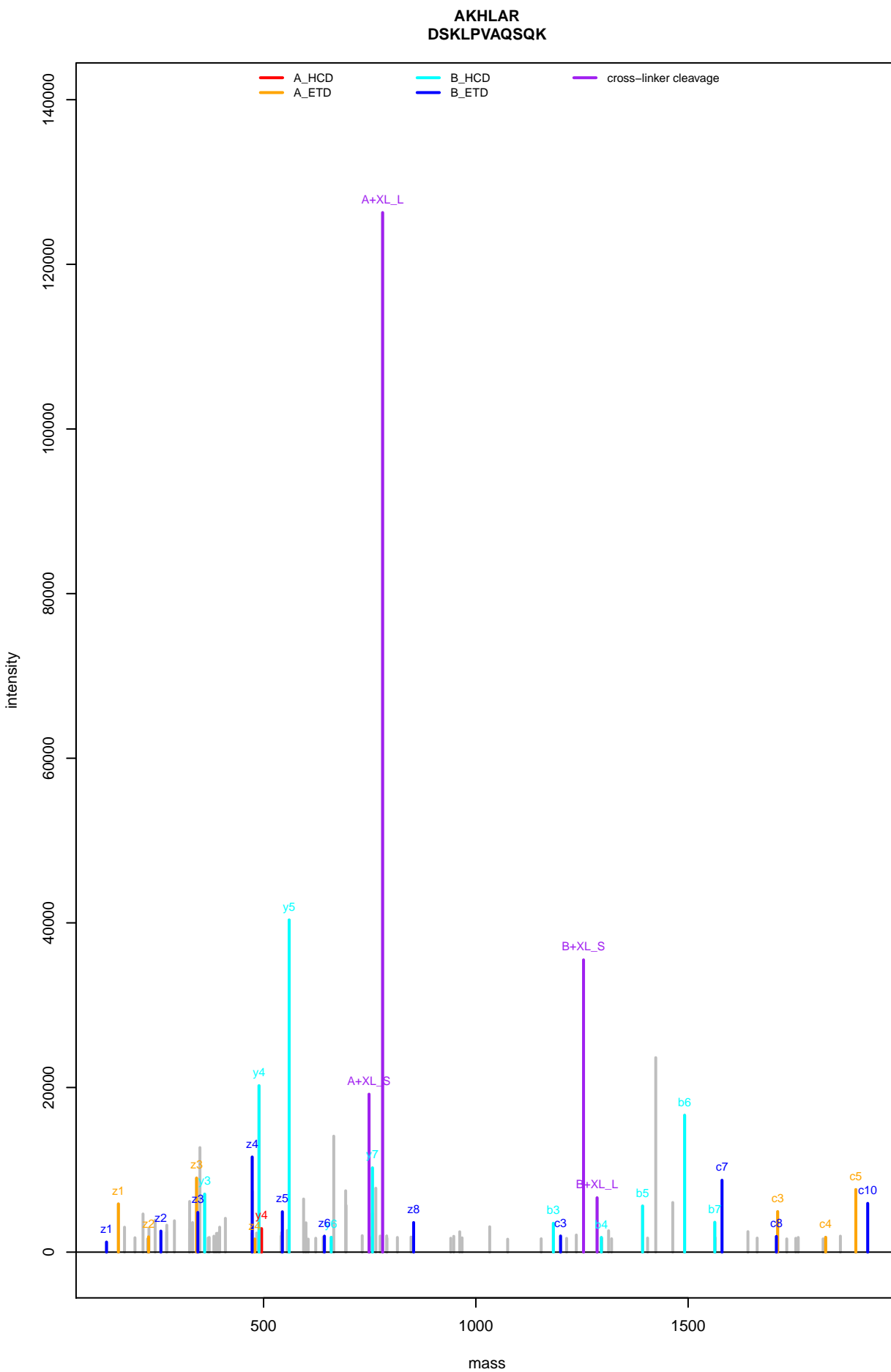

Supplement: Supplemental Data [file supp_RA117.000470_133922_0_supp_23978_fzffwf.zip › spectra_annotation/mito_DR_spectra_annotation/129-1-1-1-5-1.pdf]

# SHSLAKK KAGIFQSAK

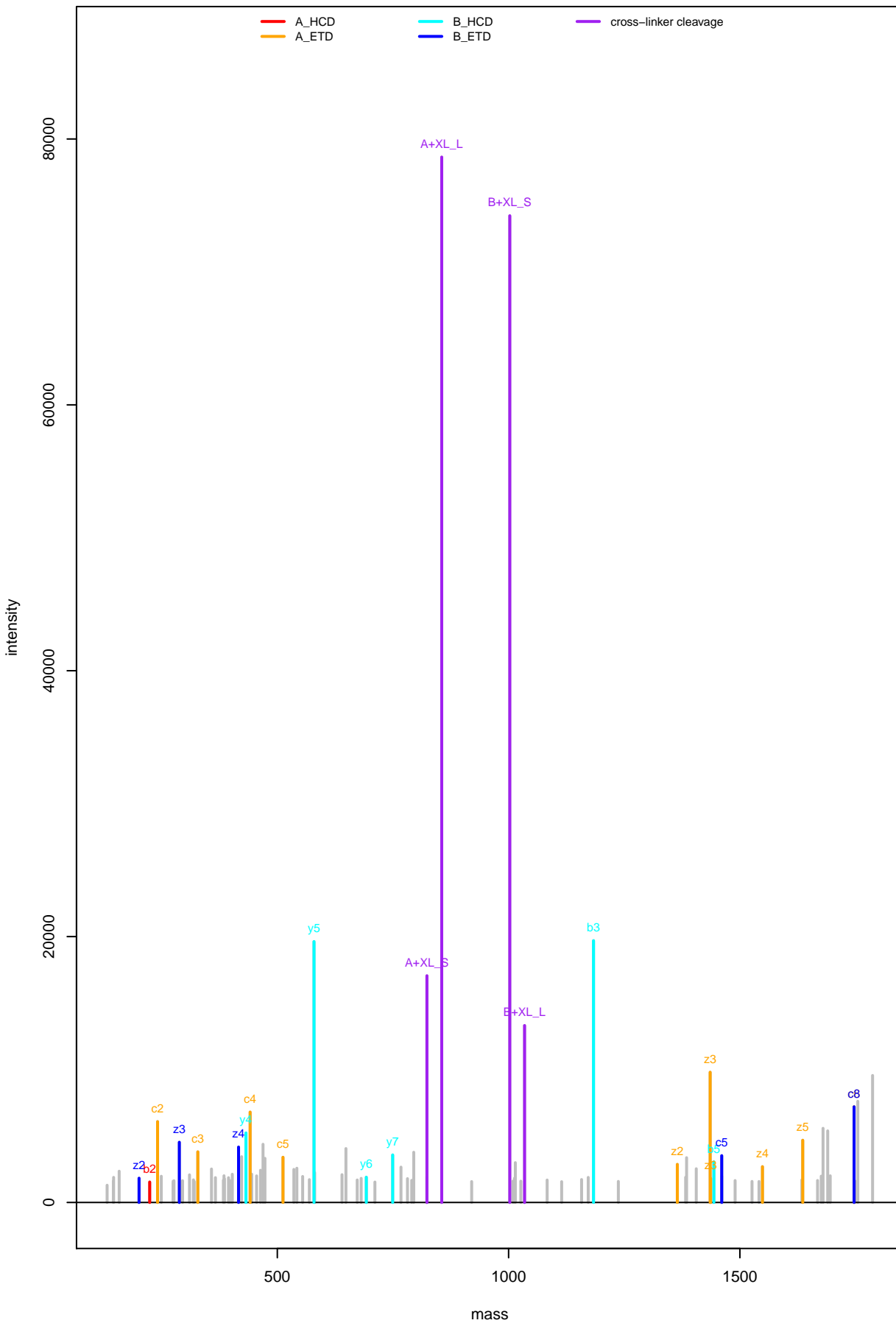

## SHSLAKK+XL\_S

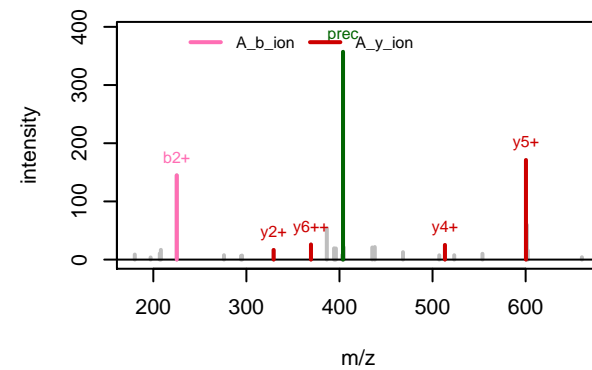

## SHSLAKK+XL\_L

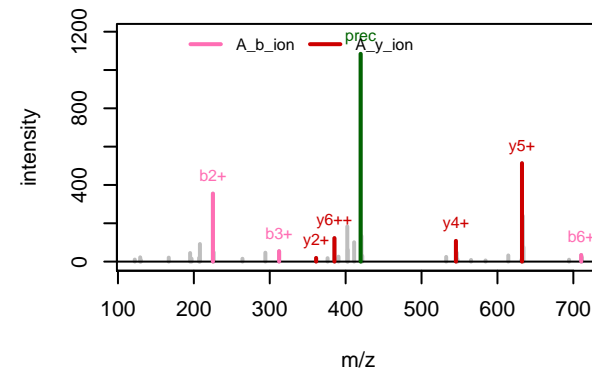

## KAGIFQSAK+XL\_S

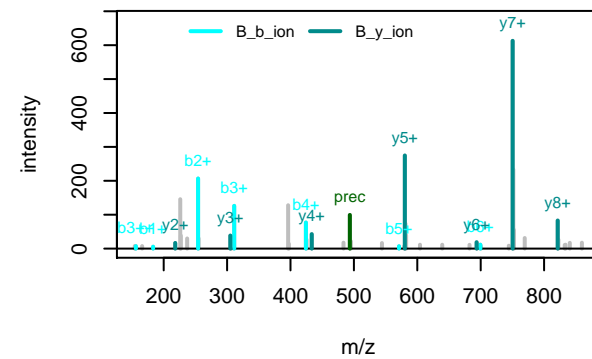

## KAGIFQSAK+XL\_L

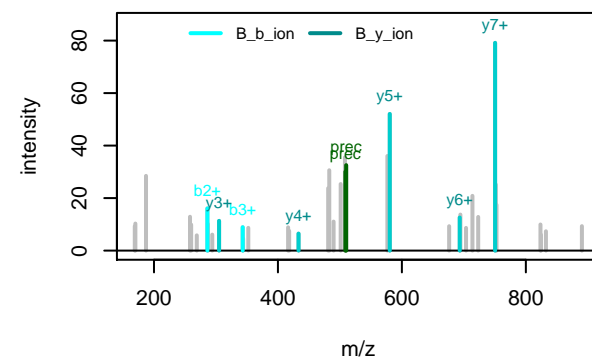

Supplement: Supplemental Data [file supp_RA117.000470_133922_0_supp_23978_fzffwf.zip › spectra_annotation/mito_DR_spectra_annotation/129-1-2-1-3-1.pdf]

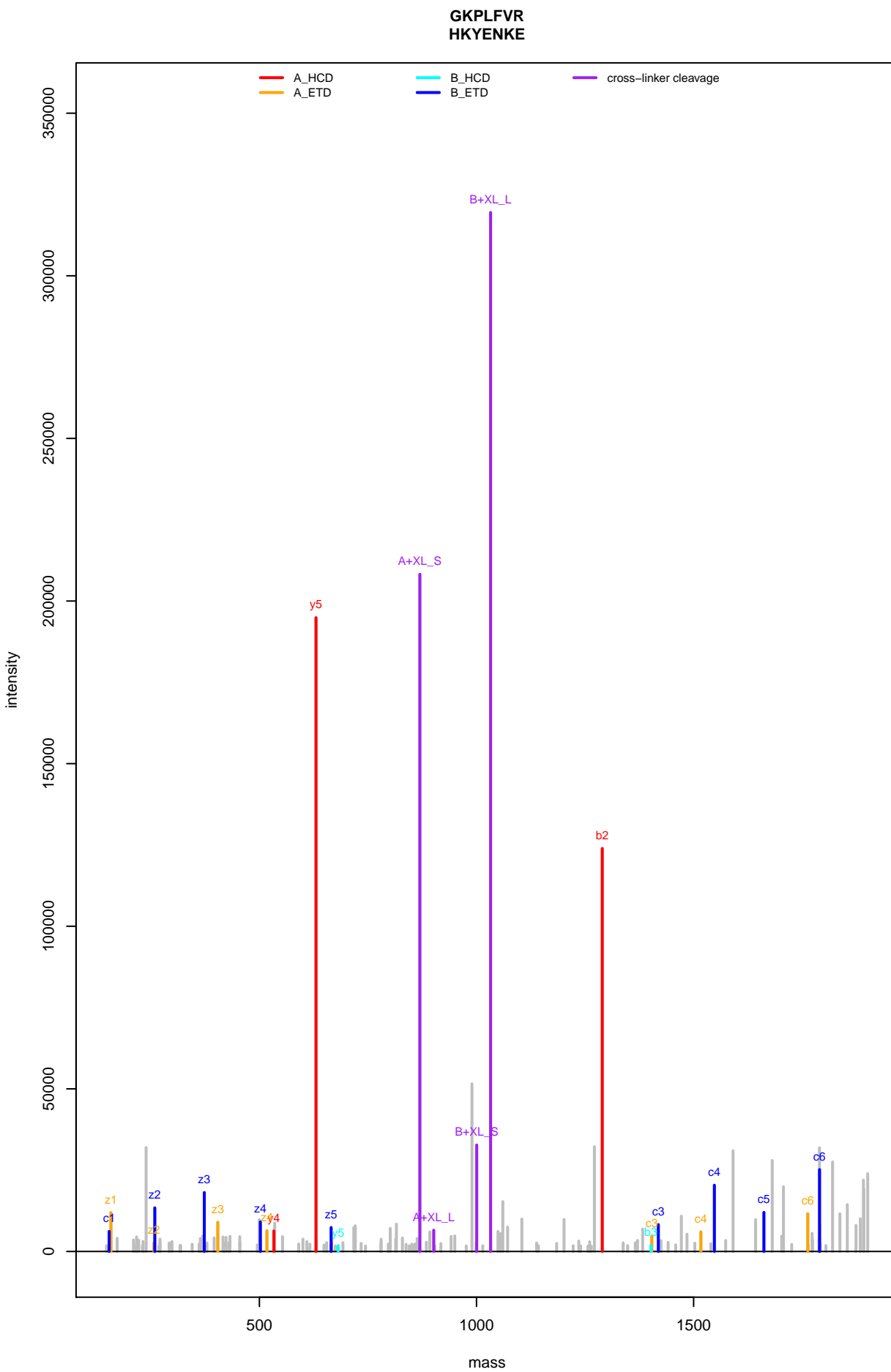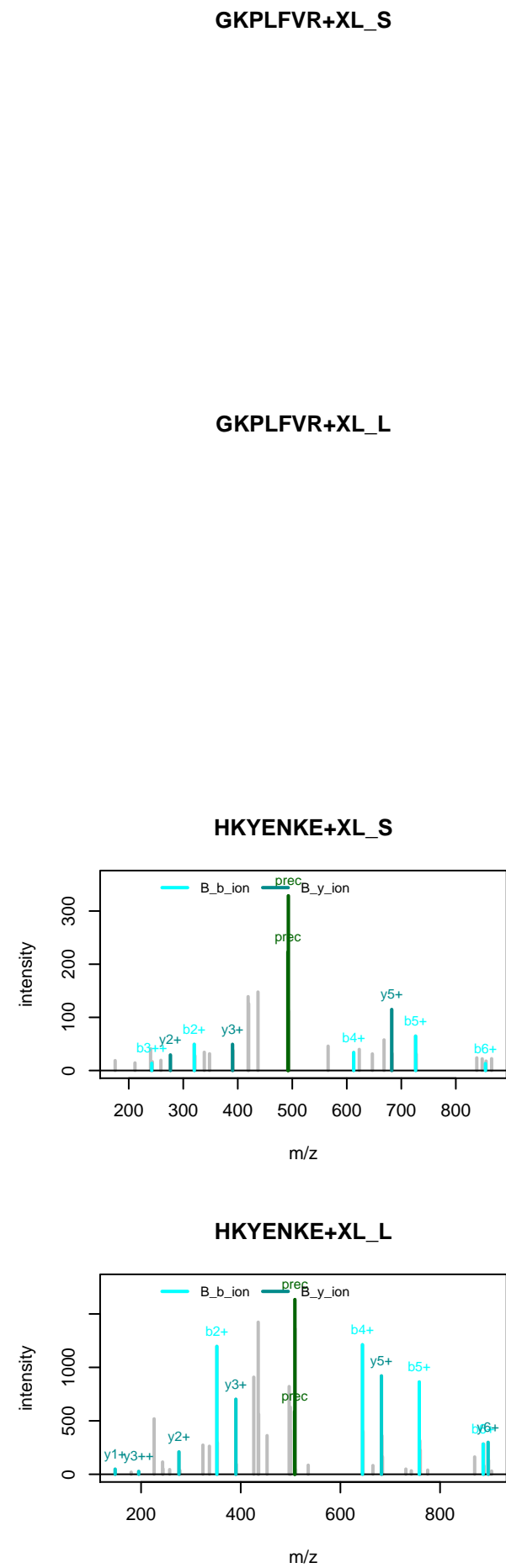

Supplement: Supplemental Data [file supp_RA117.000470_133922_0_supp_23978_fzffwf.zip › spectra_annotation/mito_DR_spectra_annotation/129-1-6-1-3-1.pdf]

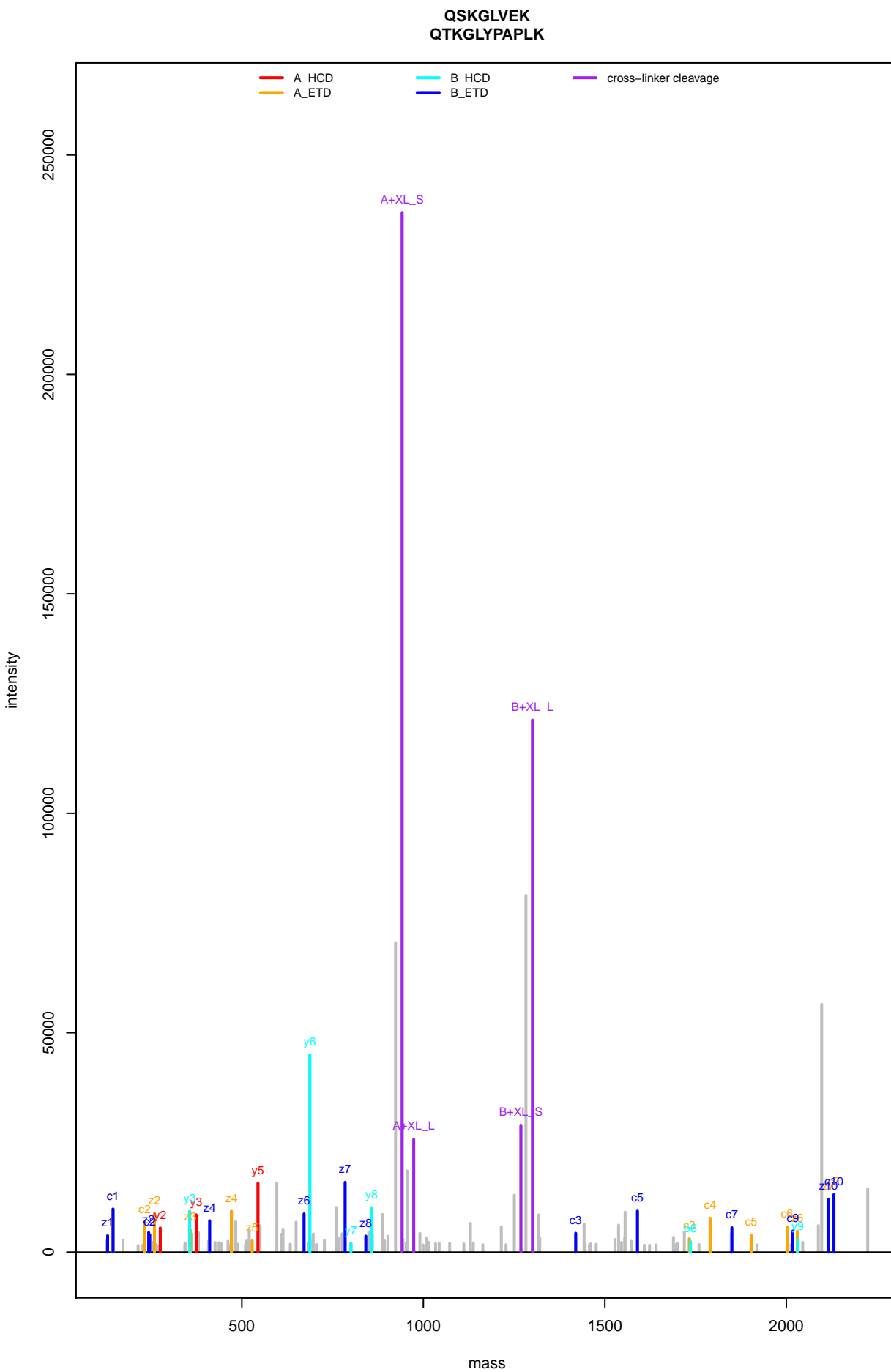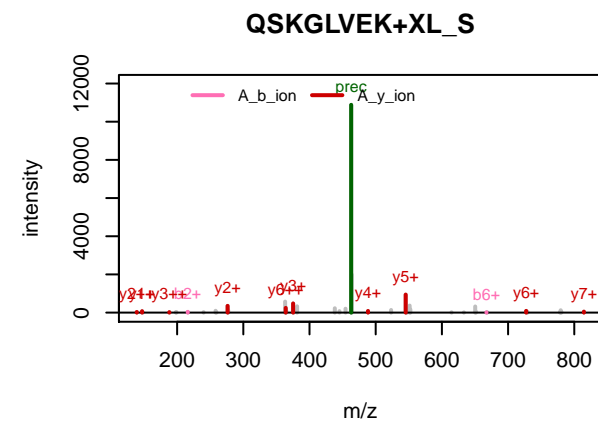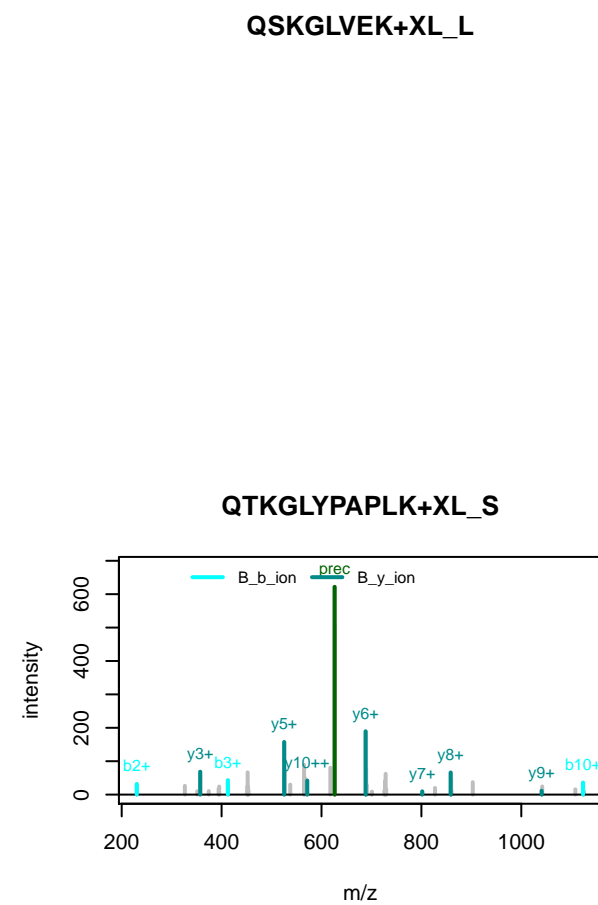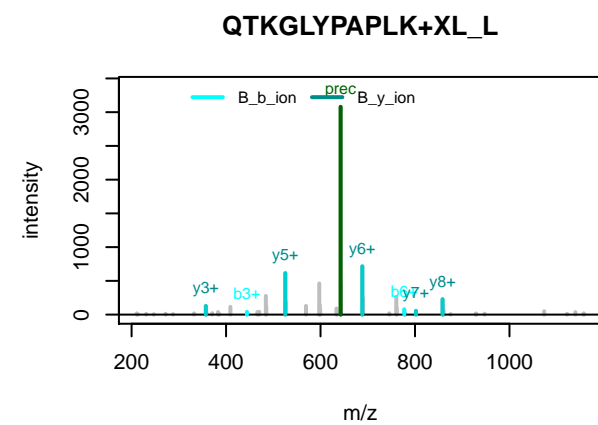

Supplement: Supplemental Data [file supp_RA117.000470_133922_0_supp_23978_fzffwf.zip › spectra_annotation/mito_DR_spectra_annotation/129-1-8-1-16-1.pdf]

# AKHLAR TKGDTPASAALAK

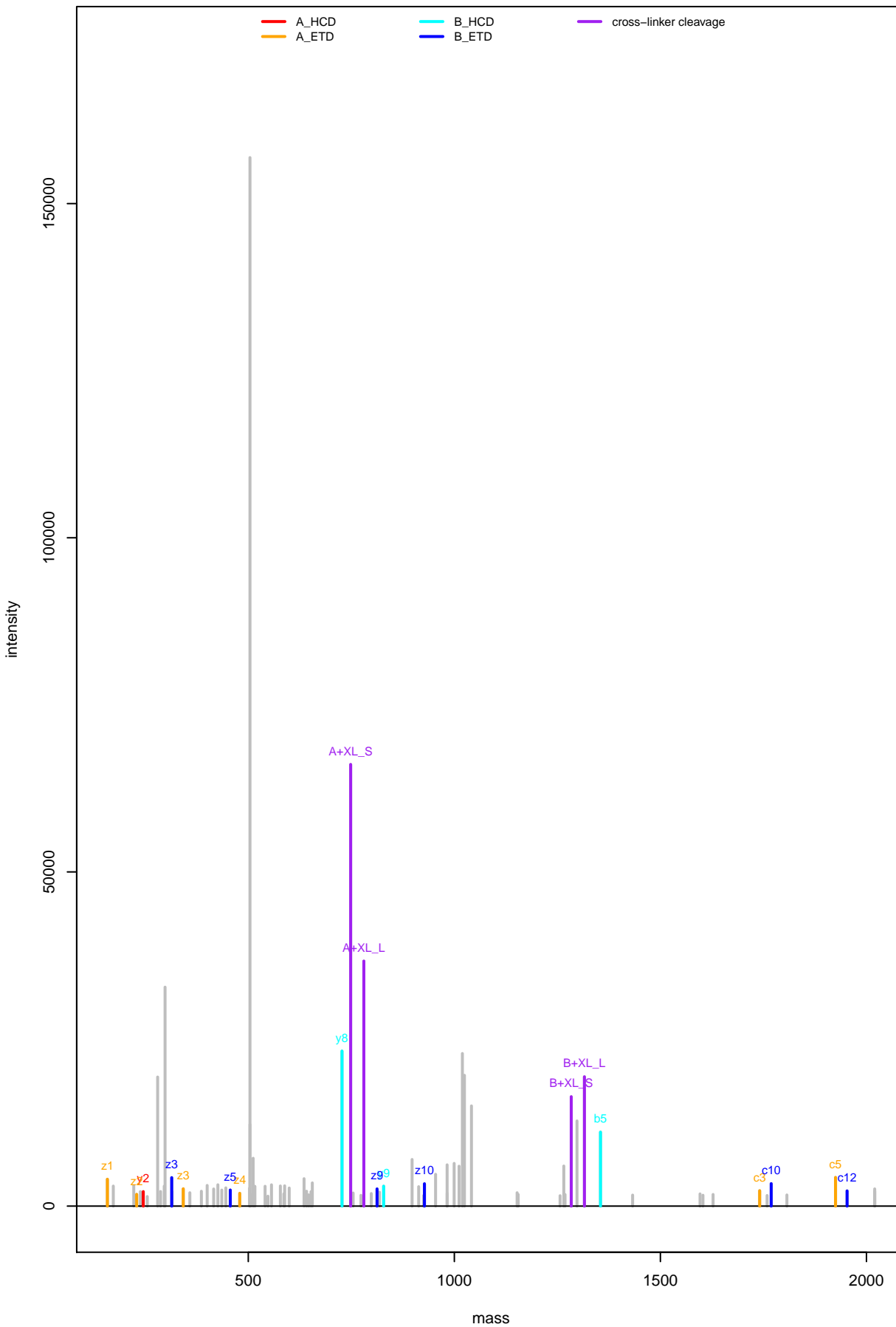

Supplement: Supplemental Data [file supp_RA117.000470_133922_0_supp_23978_fzffwf.zip › spectra_annotation/mito_DR_spectra_annotation/13-1-1-1-9-1.pdf]

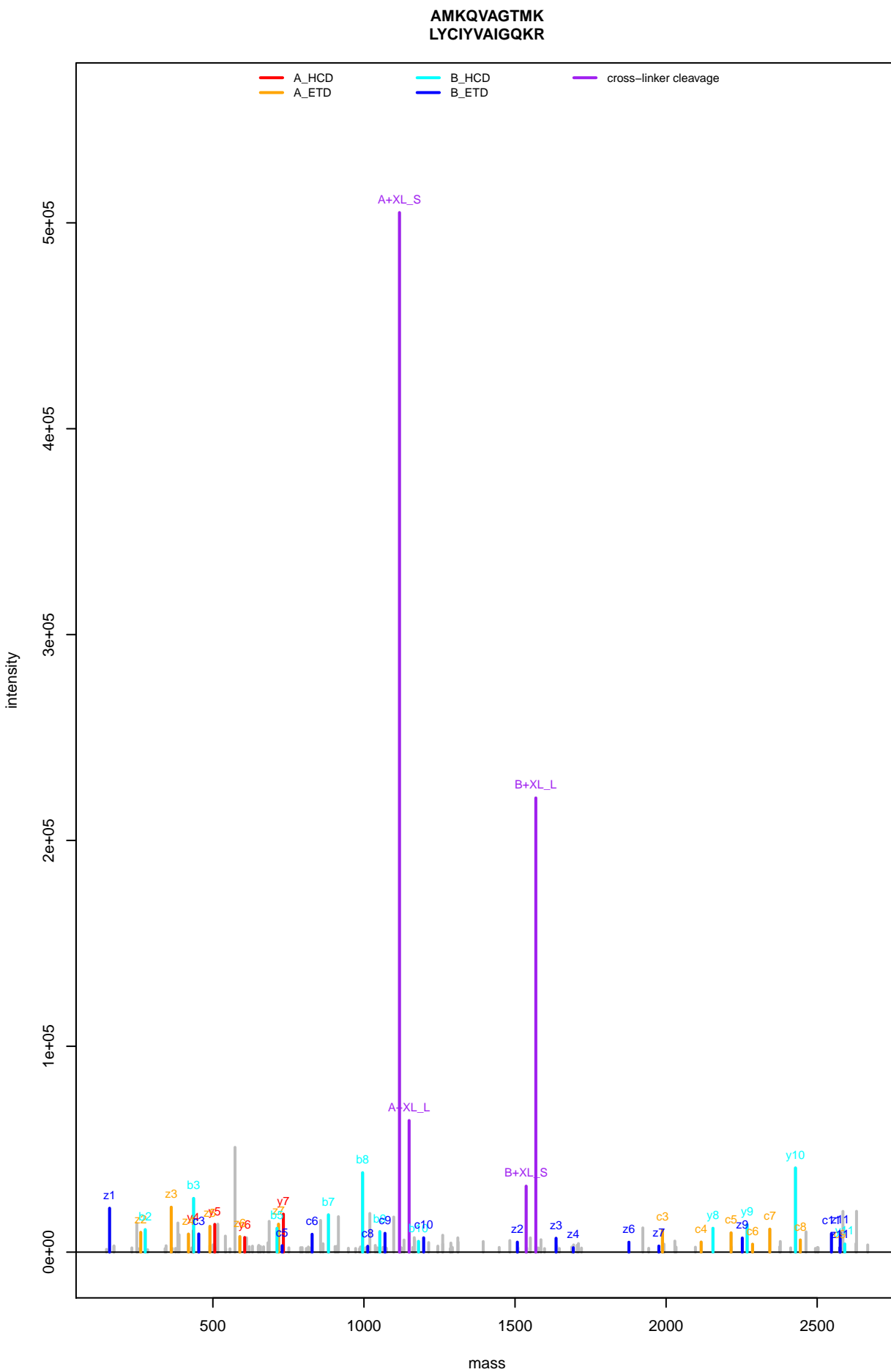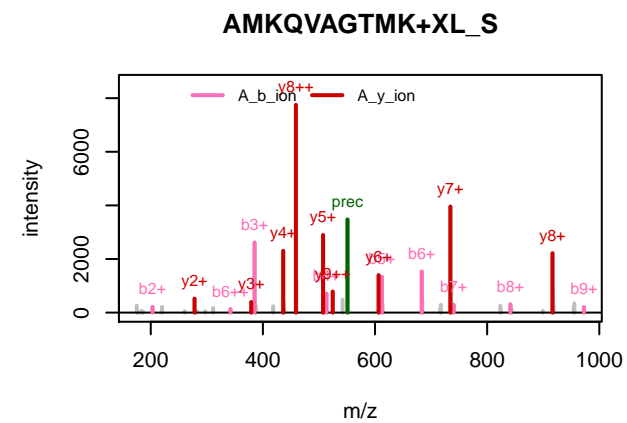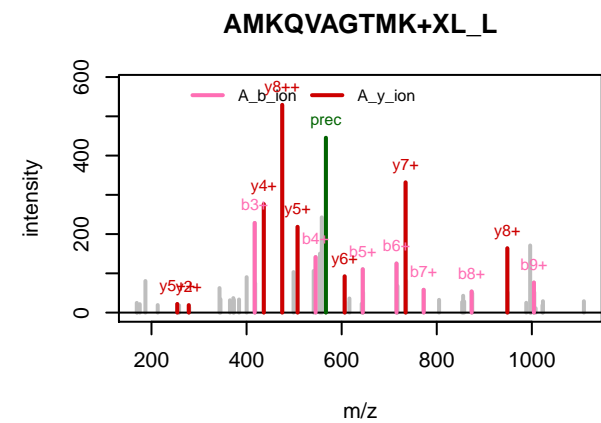

**LYCIYVAIGQKR+XL\_S**

**LYCIYVAIGQKR+XL\_L**

Supplement: Supplemental Data [file supp_RA117.000470_133922_0_supp_23978_fzffwf.zip › spectra_annotation/mito_DR_spectra_annotation/13-1-14-1-26-1.pdf]

# AVGEKEVR ISEQSDAKLK

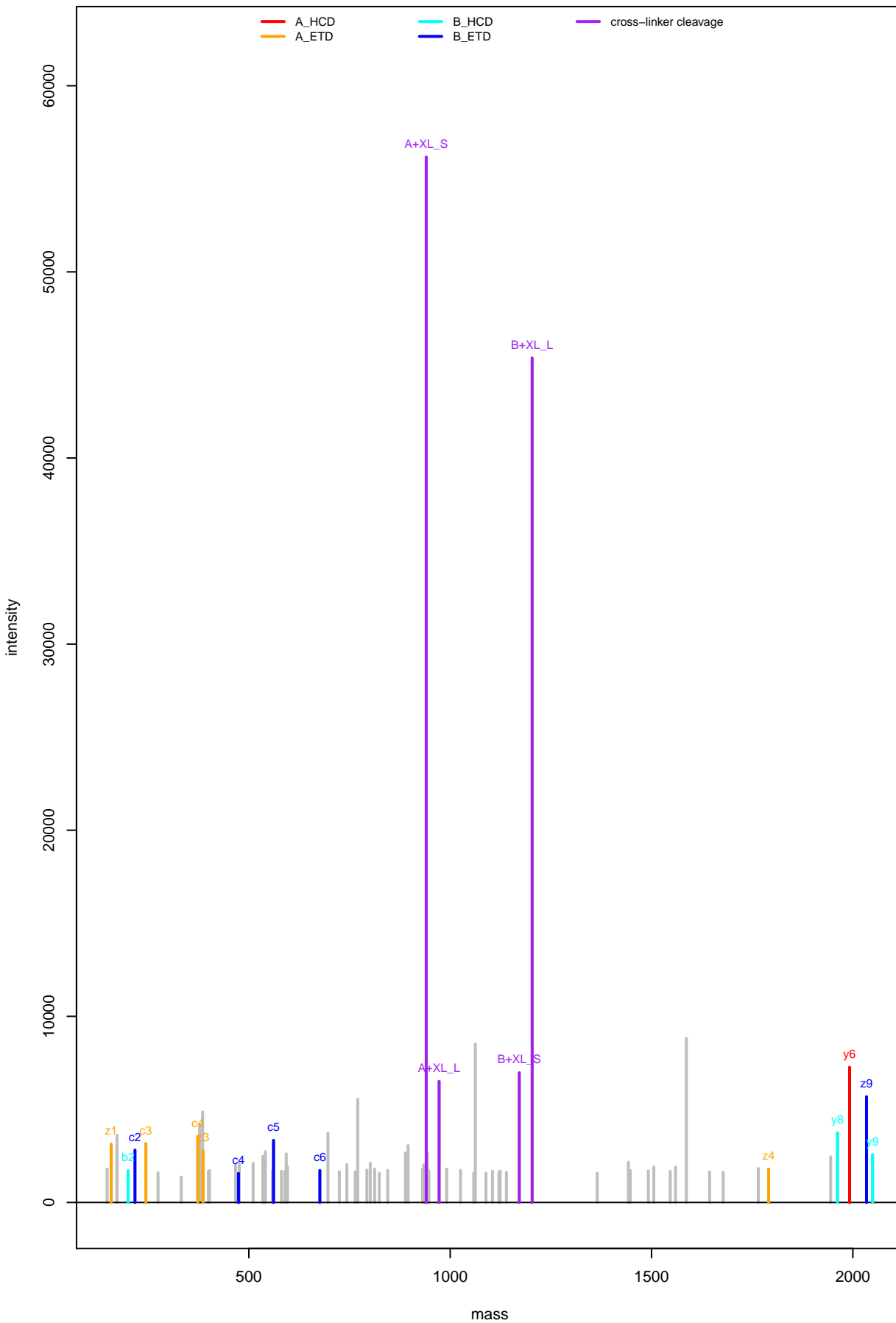

## AVGEKEVR+XL\_S

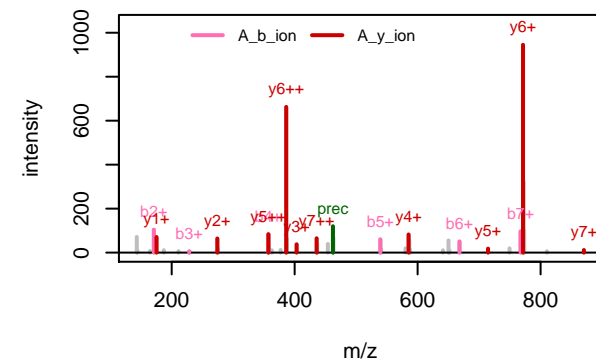

## AVGEKEVR+XL\_L

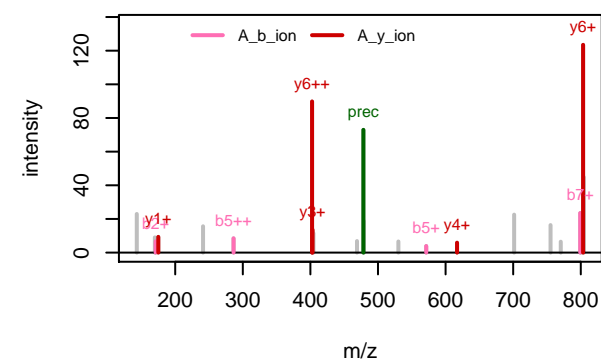

## ISEQSDAKLK+XL\_S

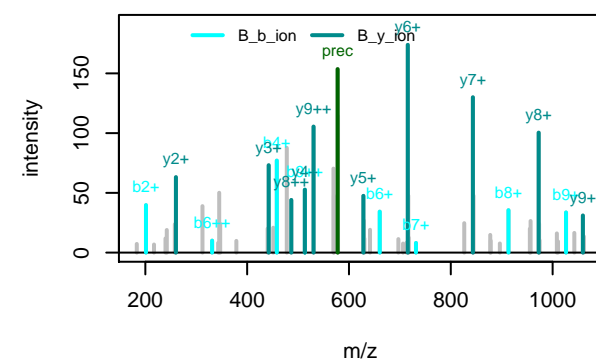

## ISEQSDAKLK+XL\_L

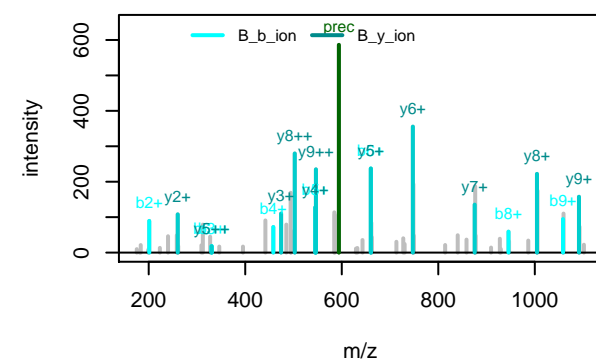

Supplement: Supplemental Data [file supp_RA117.000470_133922_0_supp_23978_fzffwf.zip › spectra_annotation/mito_DR_spectra_annotation/13-1-4-1-12-1.pdf]

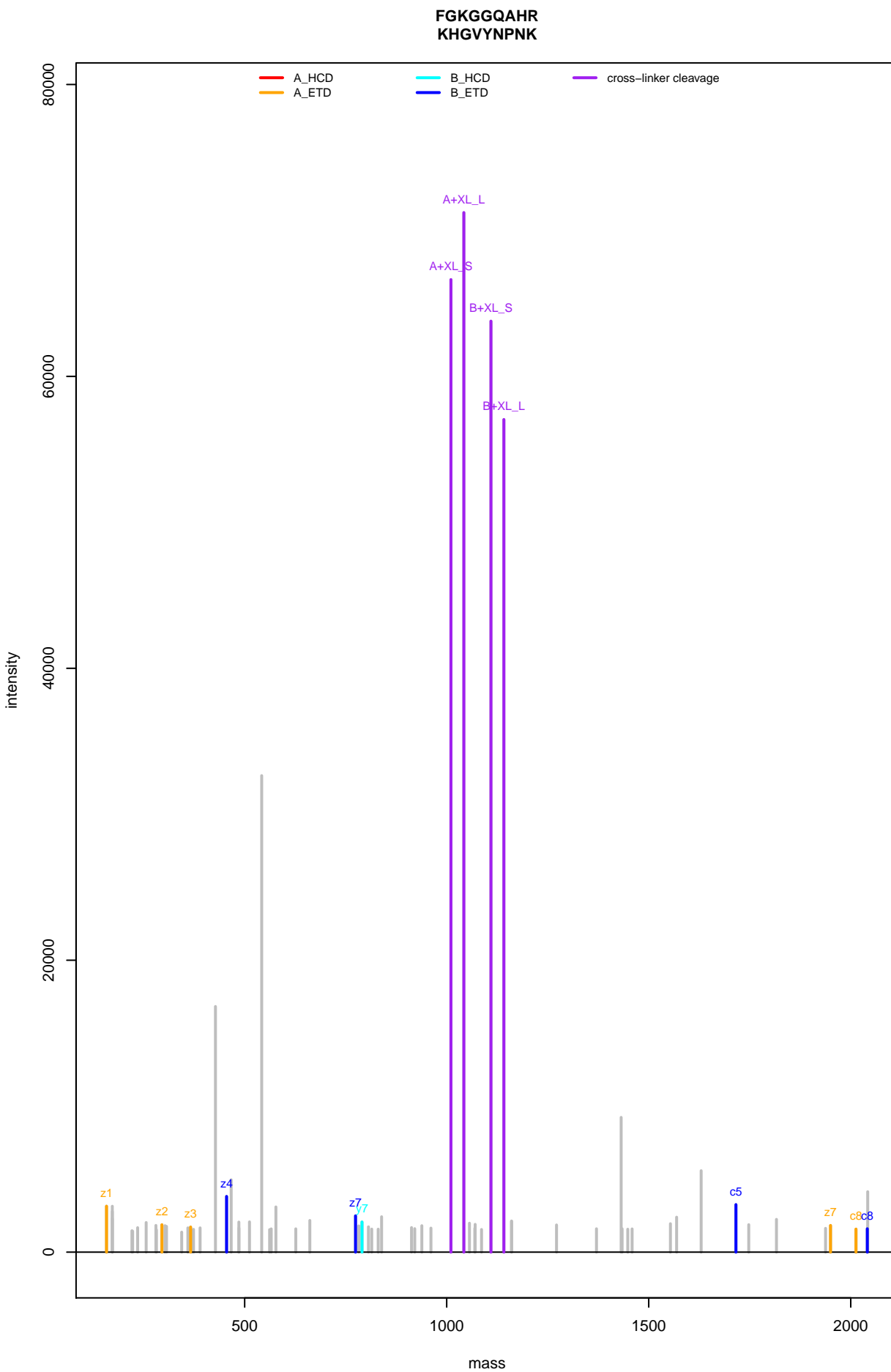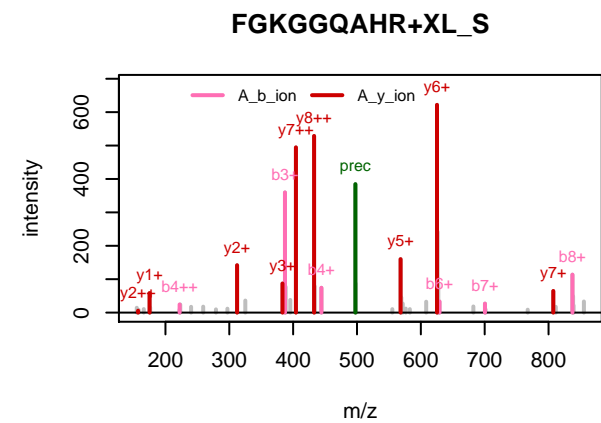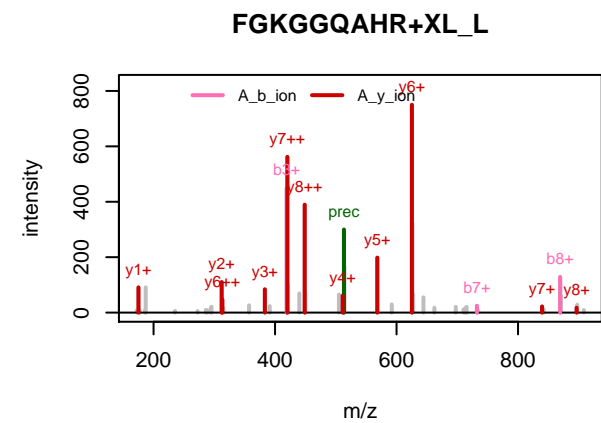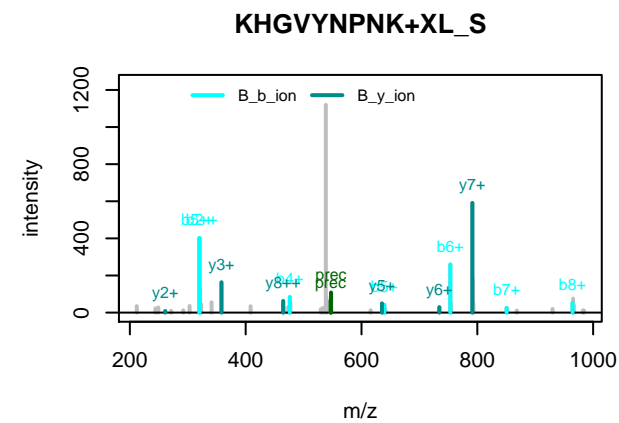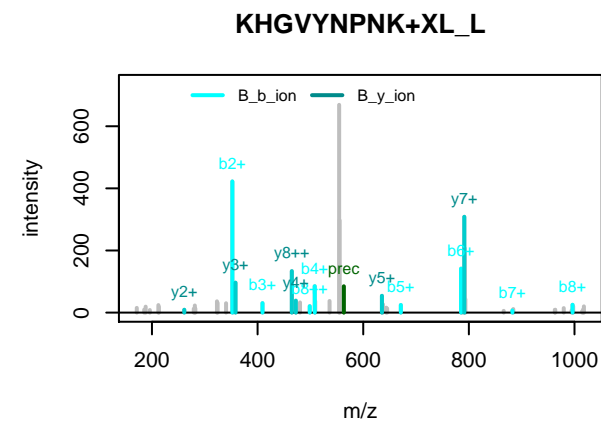

Supplement: Supplemental Data [file supp_RA117.000470_133922_0_supp_23978_fzffwf.zip › spectra_annotation/mito_DR_spectra_annotation/13-1-4-1-3-1.pdf]

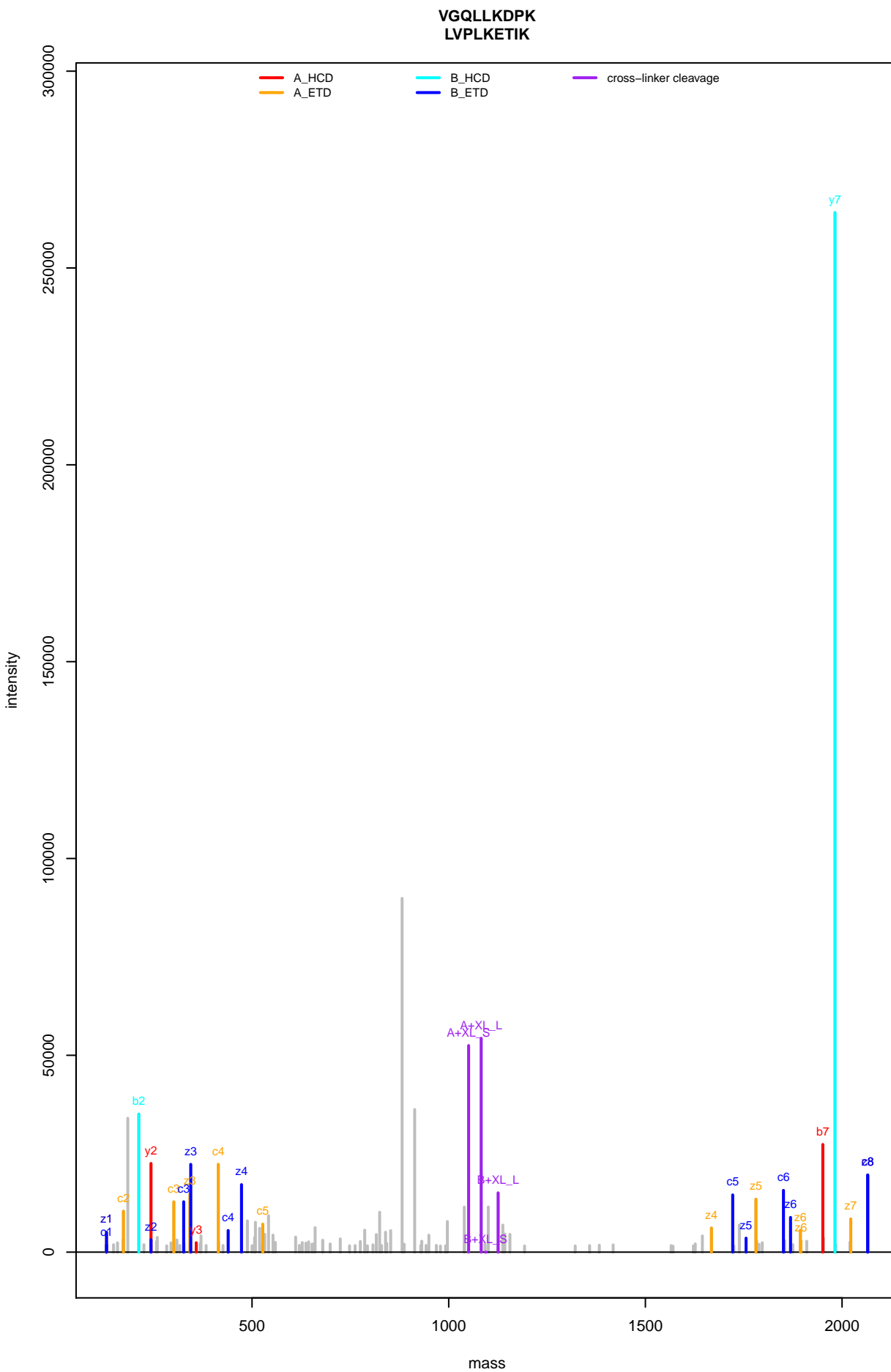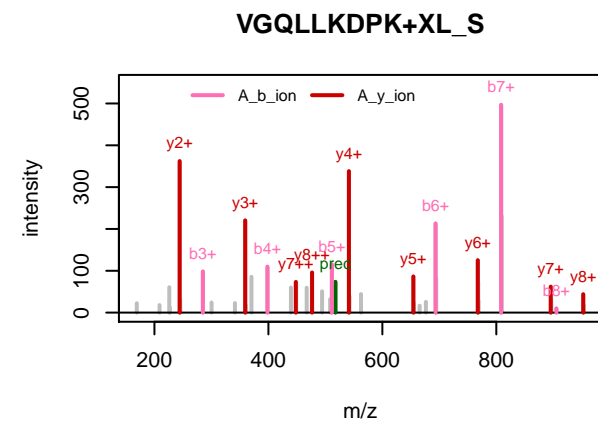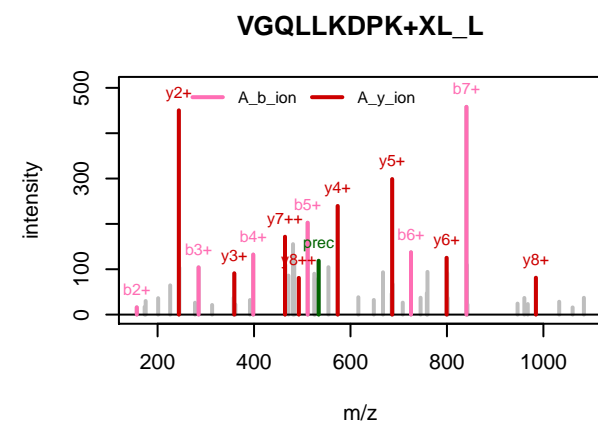

**LVPLKETIK+XL\_S**

**LVPLKETIK+XL\_L**

Supplement: Supplemental Data [file supp_RA117.000470_133922_0_supp_23978_fzffwf.zip › spectra_annotation/mito_DR_spectra_annotation/13-1-4-1-4-1.pdf]

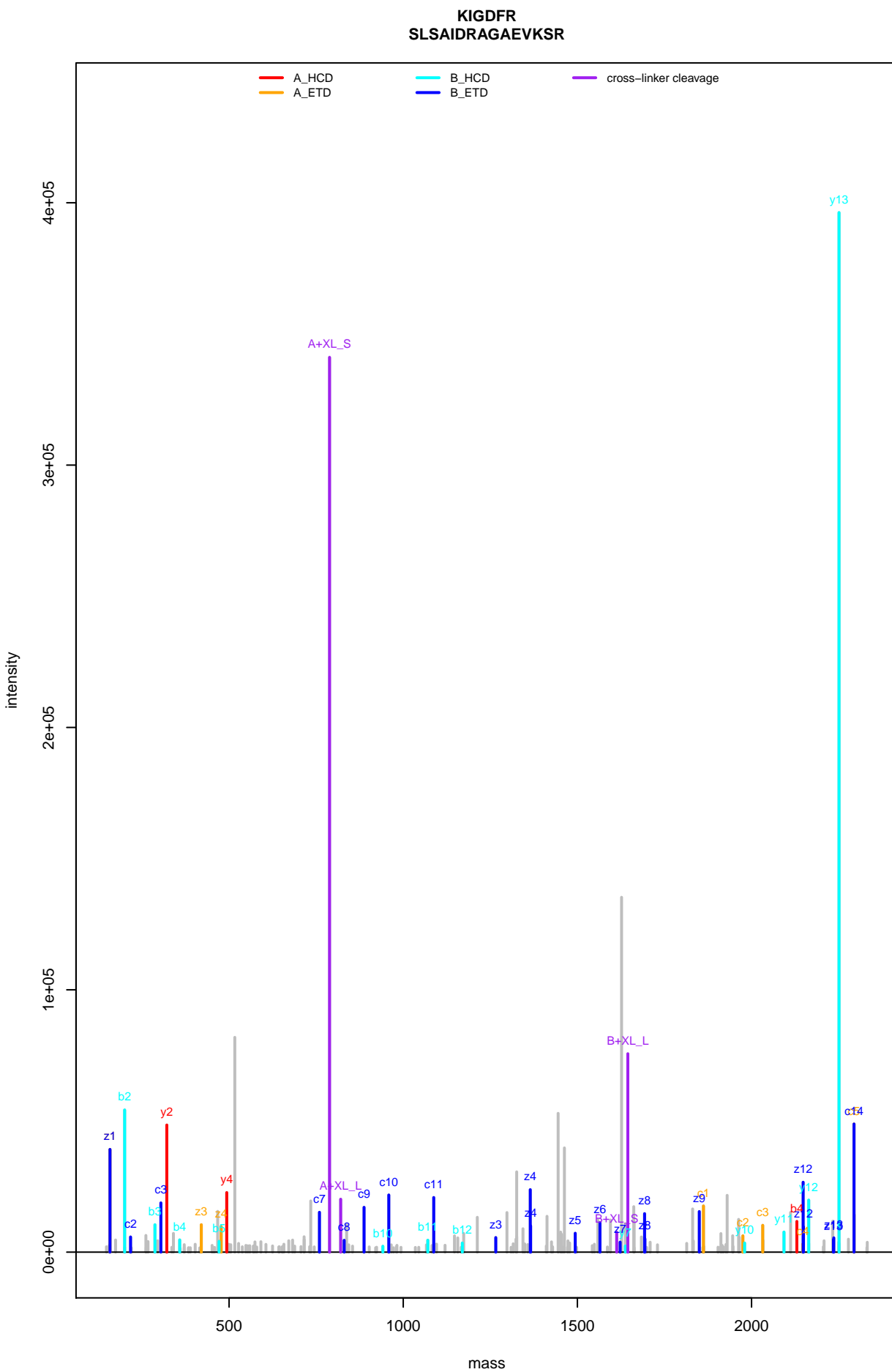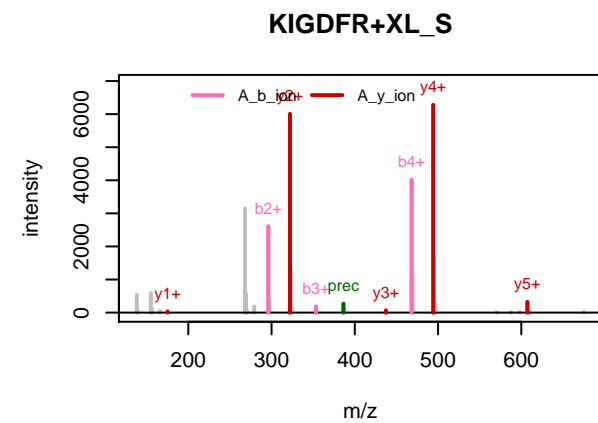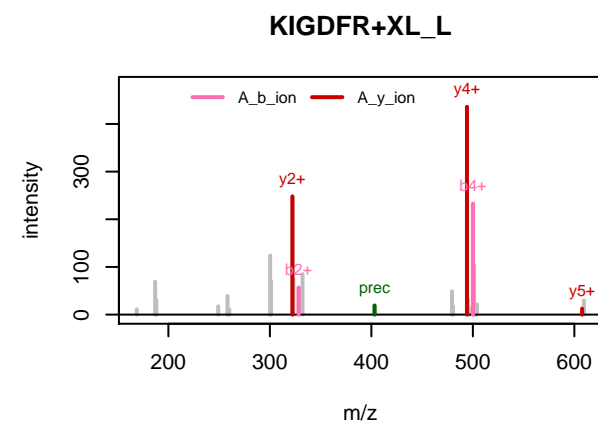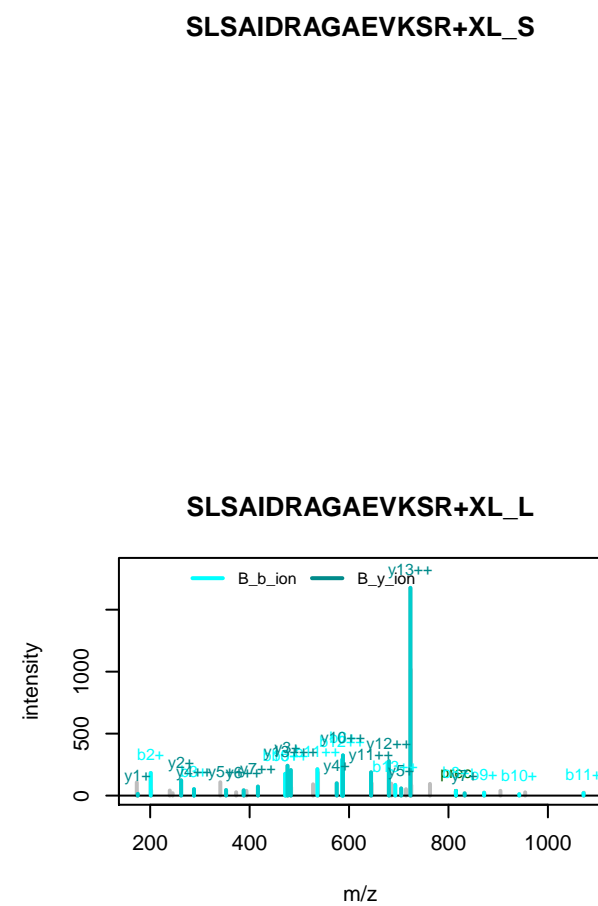

Supplement: Supplemental Data [file supp_RA117.000470_133922_0_supp_23978_fzffwf.zip › spectra_annotation/mito_DR_spectra_annotation/13-1-5-1-15-1.pdf]

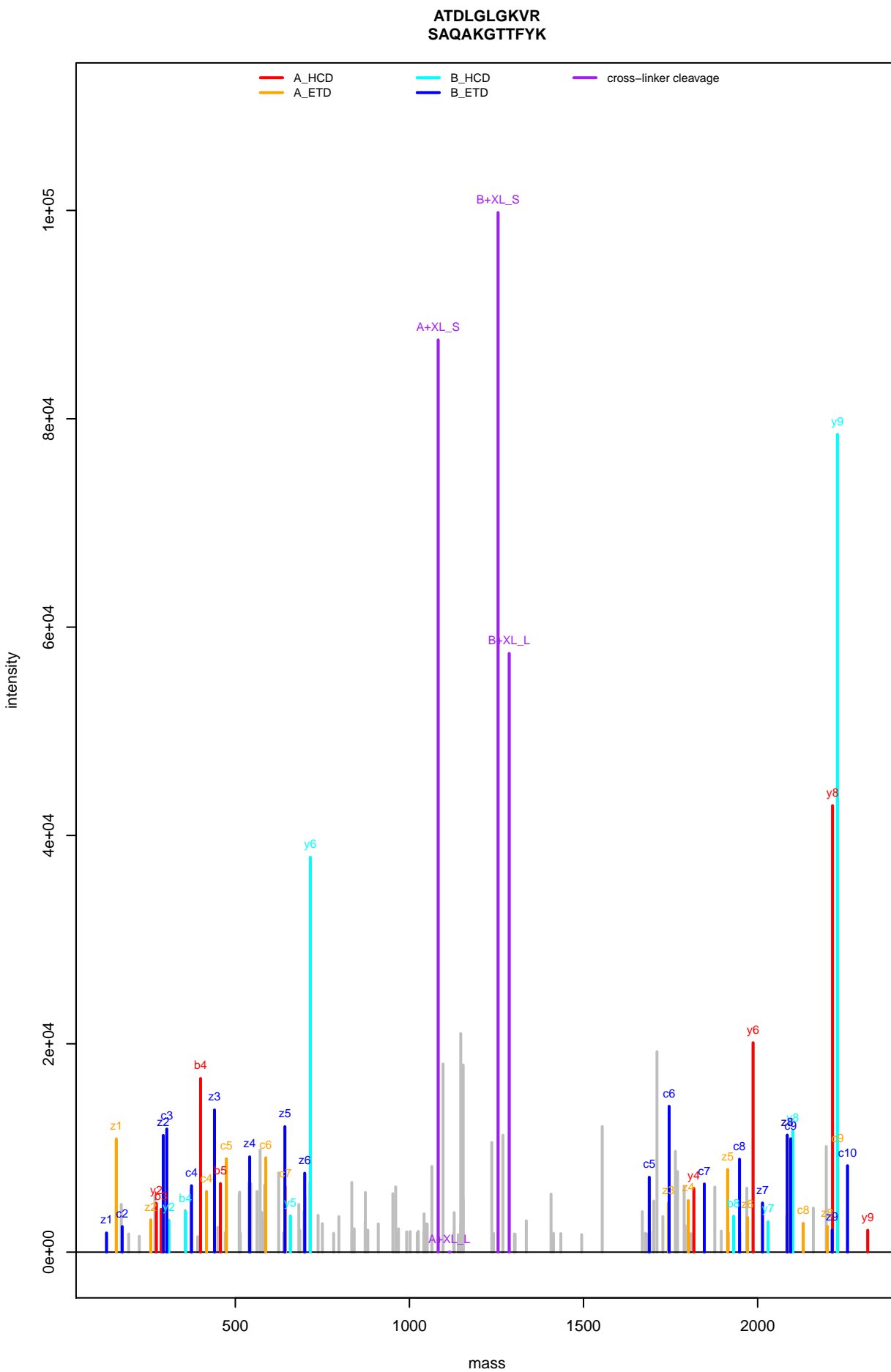

**ATDLGLGKVR+XL\_S**

**ATDLGLGKVR+XL\_L**

**SAQAKGTTFYK+XL\_S**

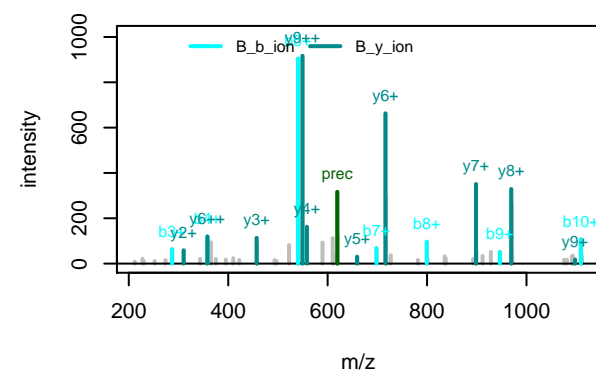

**SAQAKGTTFYK+XL\_L**

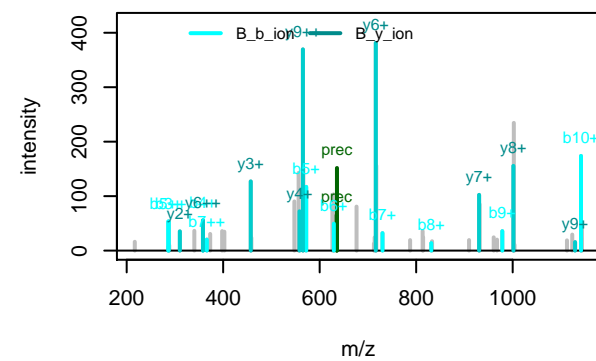

Supplement: Supplemental Data [file supp_RA117.000470_133922_0_supp_23978_fzffwf.zip › spectra_annotation/mito_DR_spectra_annotation/13-1-5-1-3-1.pdf]

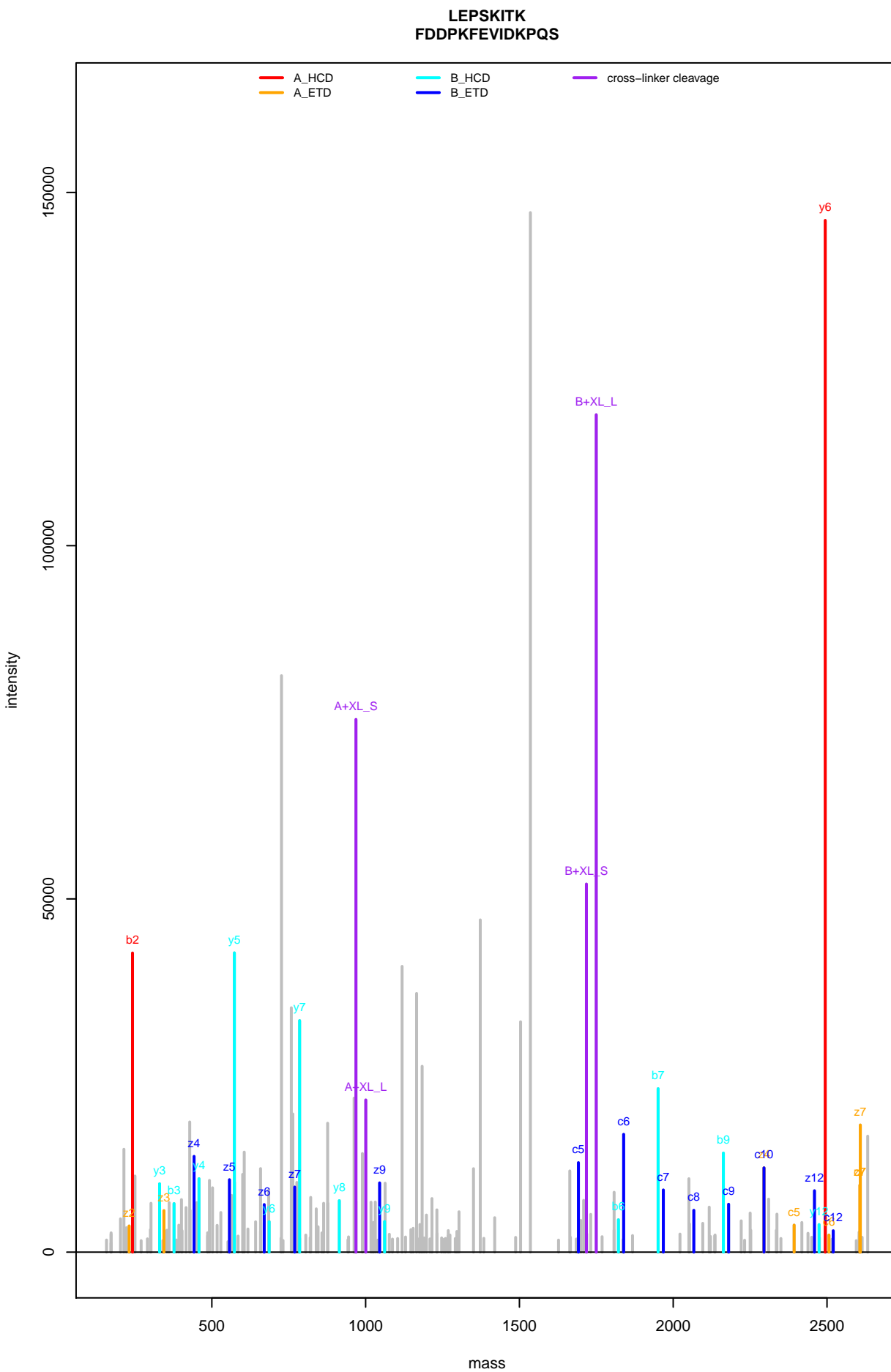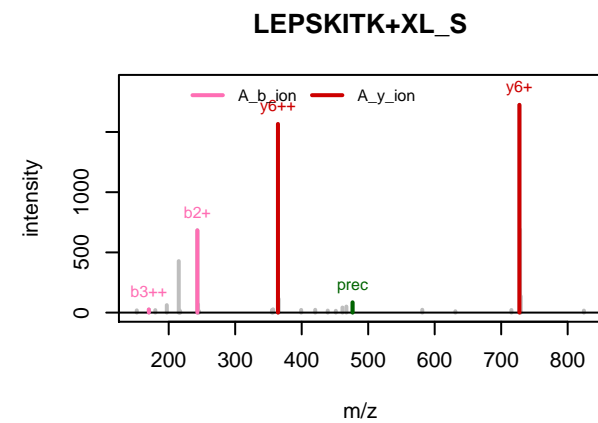

**LEPSKITK+XL\_L**

**FDDPKFEVIDKPQS+XL\_S**

**FDDPKFEVIDKPQS+XL\_L**

Supplement: Supplemental Data [file supp_RA117.000470_133922_0_supp_23978_fzffwf.zip › spectra_annotation/mito_DR_spectra_annotation/130-1-19-1-3-1.pdf]

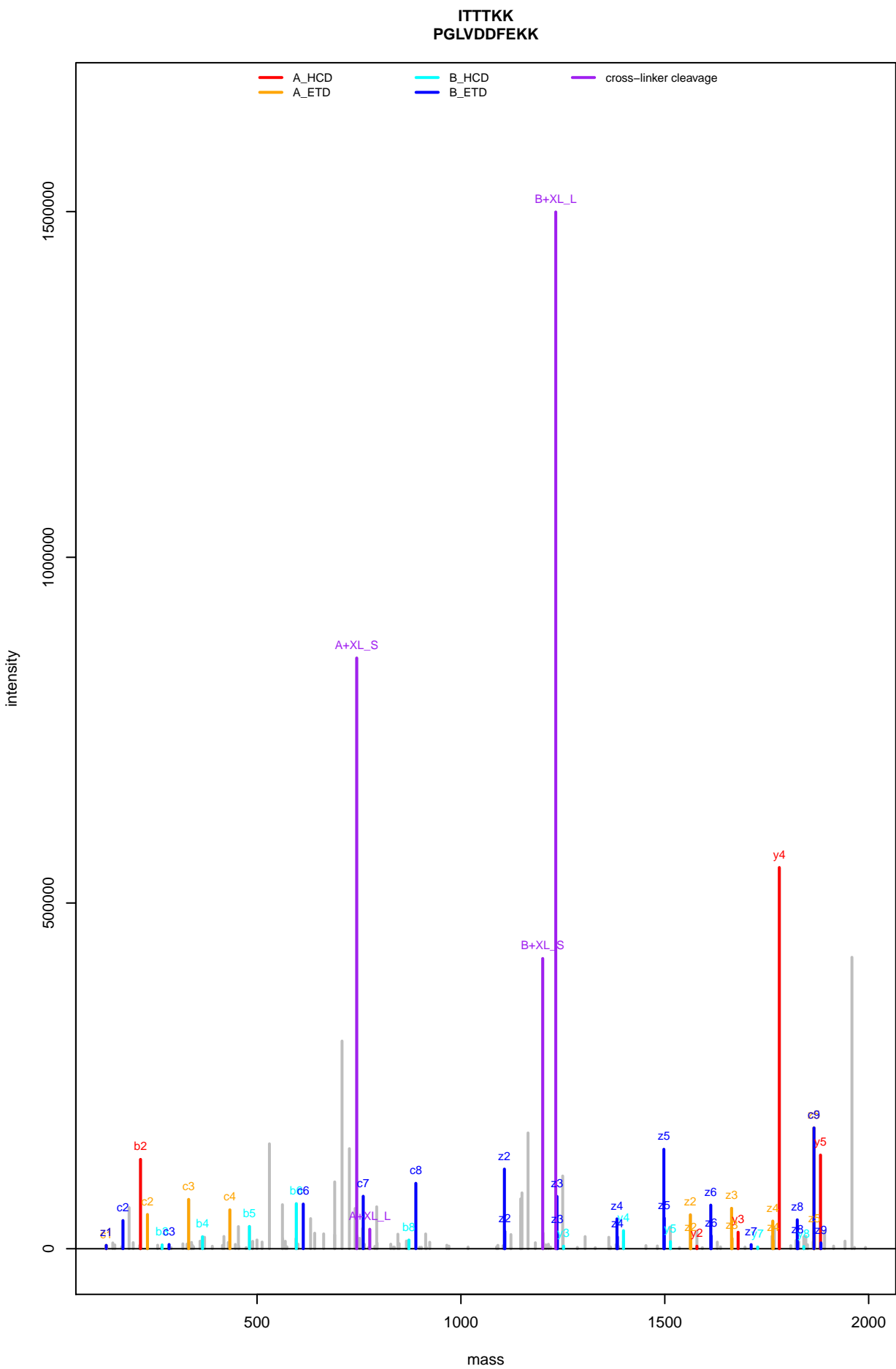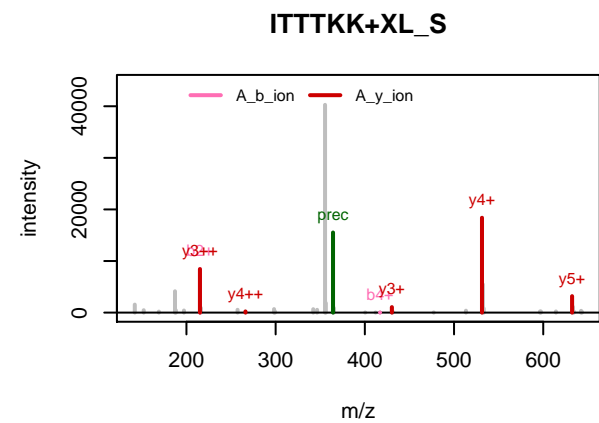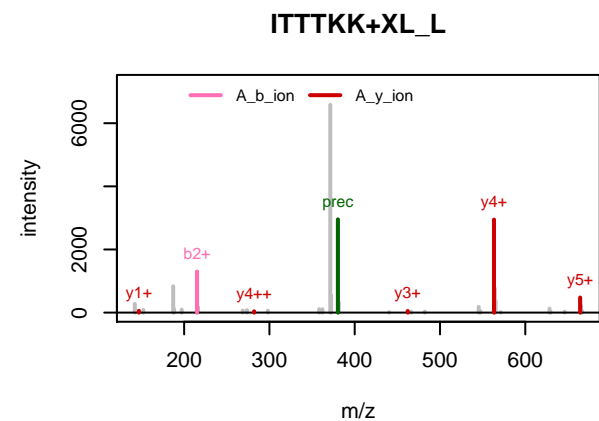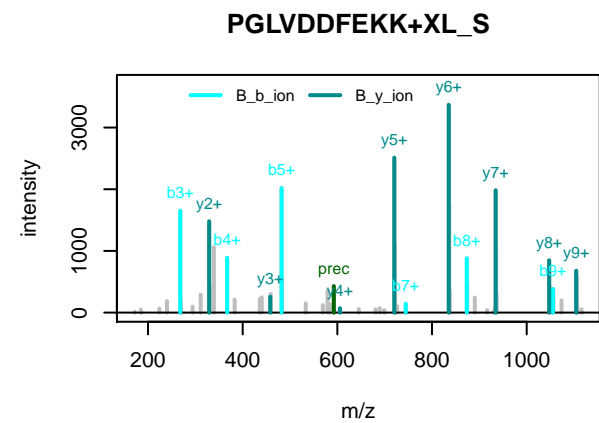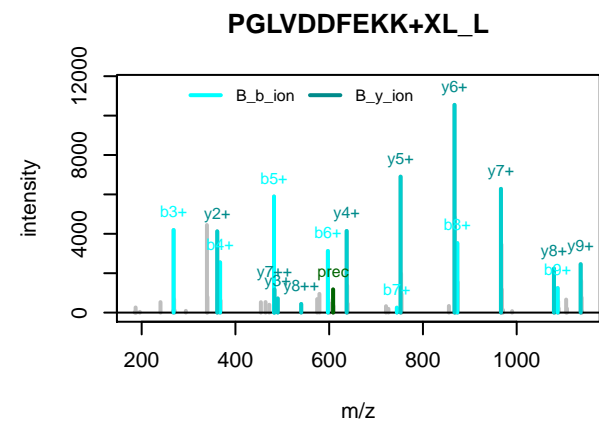

Supplement: Supplemental Data [file supp_RA117.000470_133922_0_supp_23978_fzffwf.zip › spectra_annotation/mito_DR_spectra_annotation/131-1-1-1-5-1.pdf]

TGDKPR  
LLLQVQHASKQISAEK

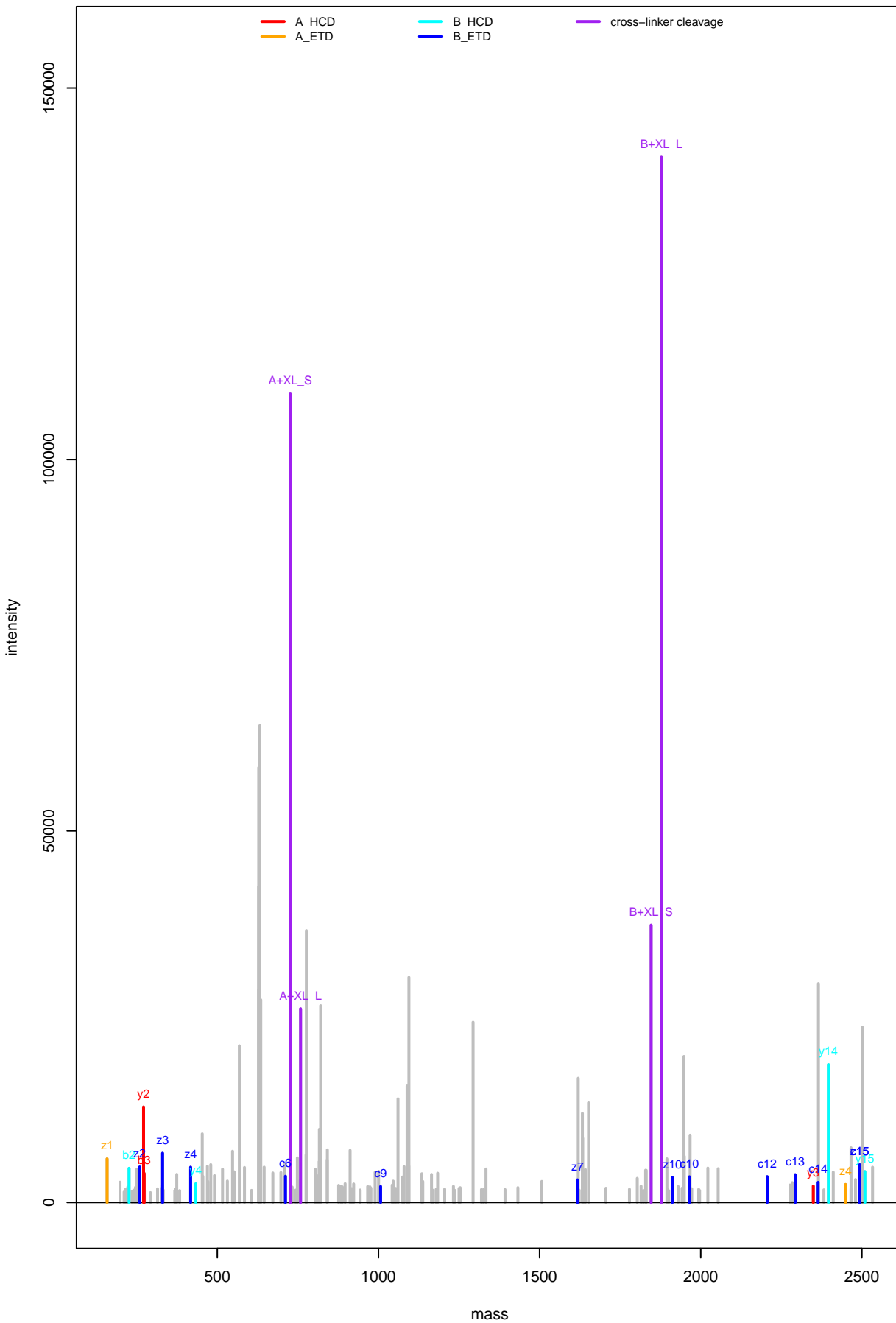

TGDKPR+XL\_S

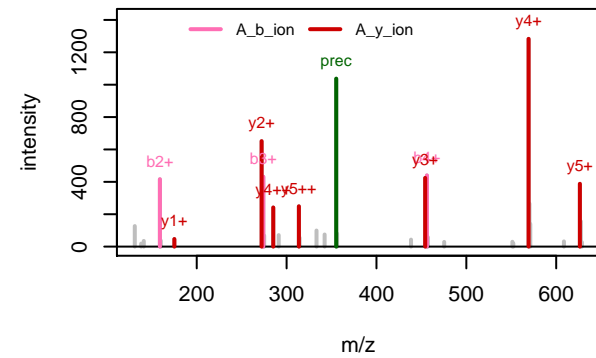

TGDKPR+XL\_L

LLLQVQHASKQISAEK+XL\_S

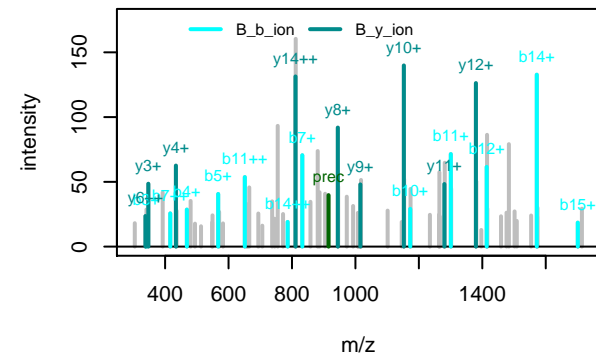

LLLQVQHASKQISAEK+XL\_L

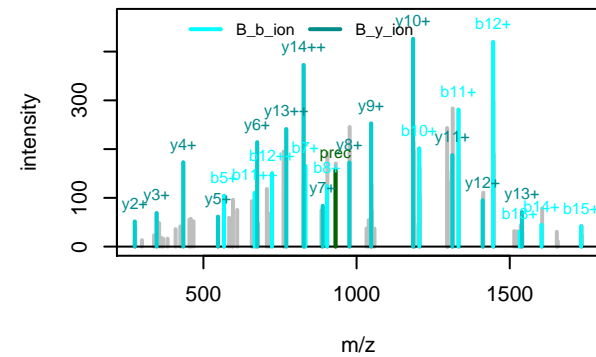

Supplement: Supplemental Data [file supp_RA117.000470_133922_0_supp_23978_fzffwf.zip › spectra_annotation/mito_DR_spectra_annotation/131-1-2-1-4-1.pdf]

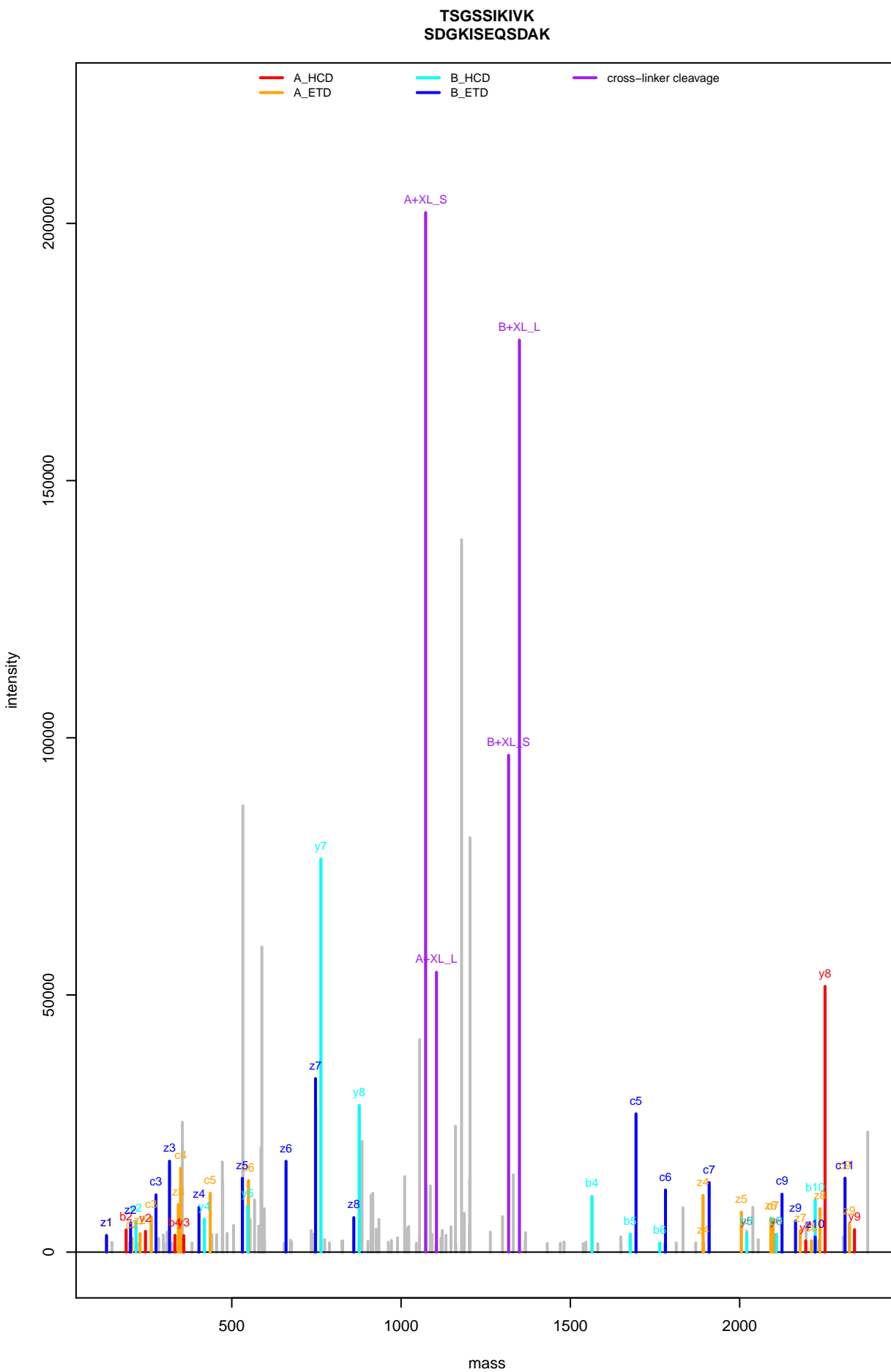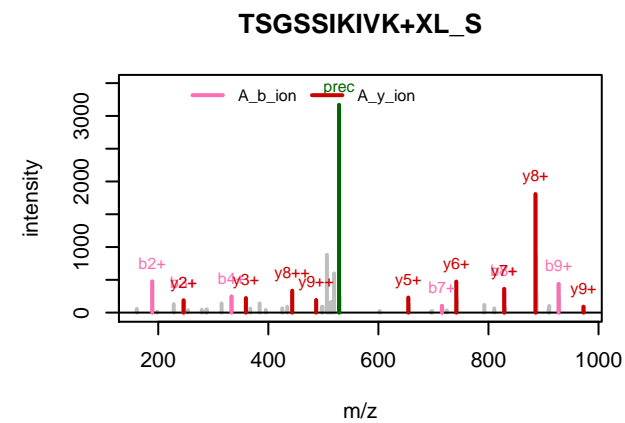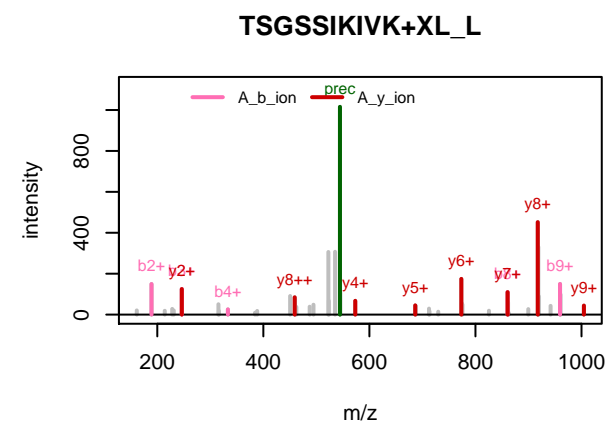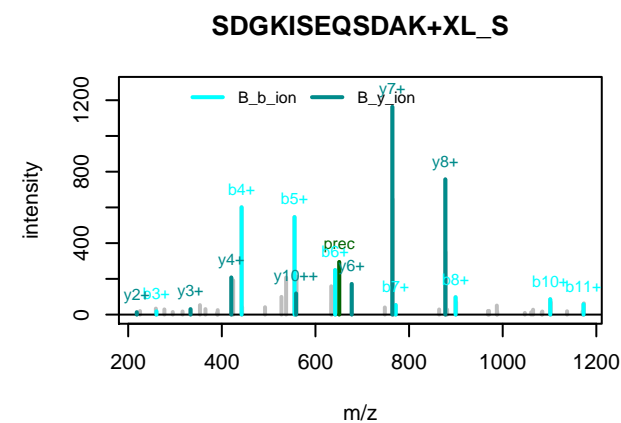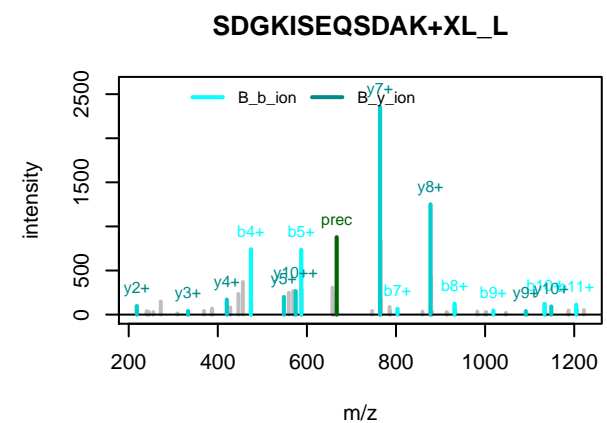

Supplement: Supplemental Data [file supp_RA117.000470_133922_0_supp_23978_fzffwf.zip › spectra_annotation/mito_DR_spectra_annotation/131-1-7-1-6-1.pdf]

# IREYKSK ANAECTSGSSIK

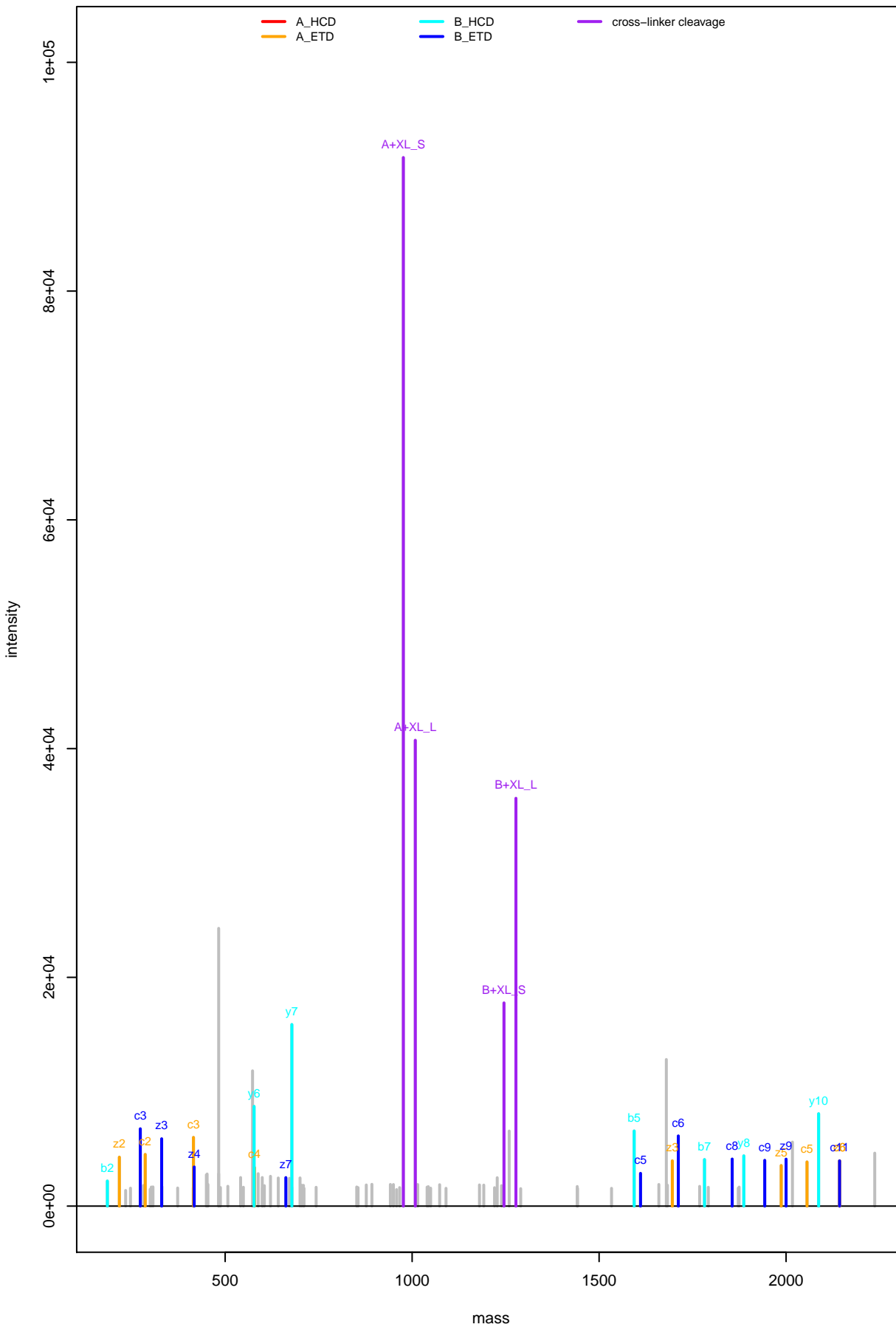

## IREYKSK+XL\_S

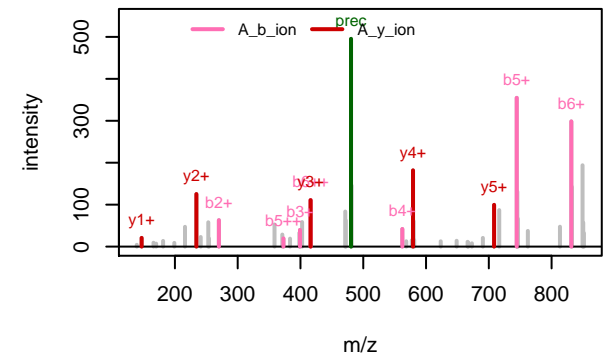

## IREYKSK+XL\_L

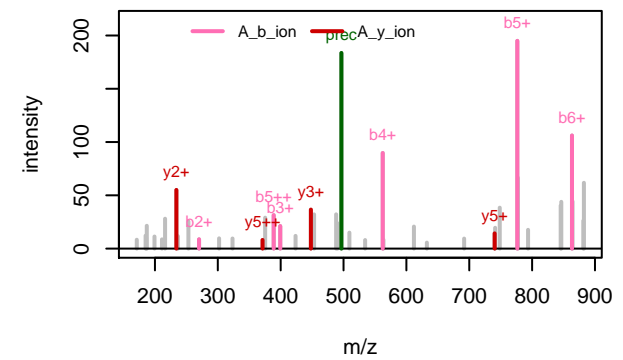

## ANAECTSGSSIK+XL\_S

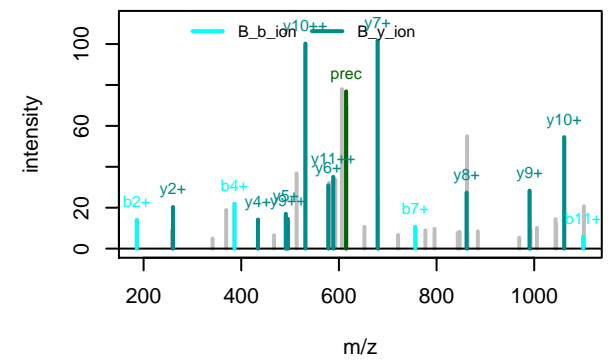

## ANAECTSGSSIK+XL\_L

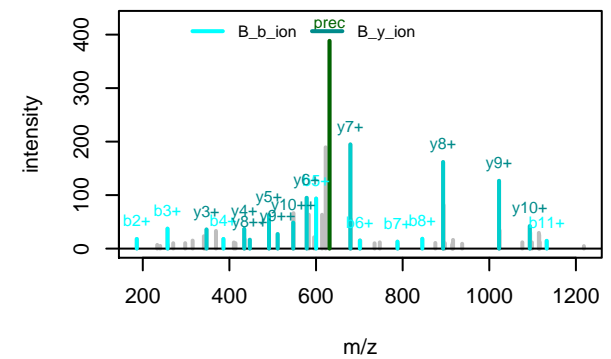

Supplement: Supplemental Data [file supp_RA117.000470_133922_0_supp_23978_fzffwf.zip › spectra_annotation/mito_DR_spectra_annotation/132-1-10-1-3-1.pdf]

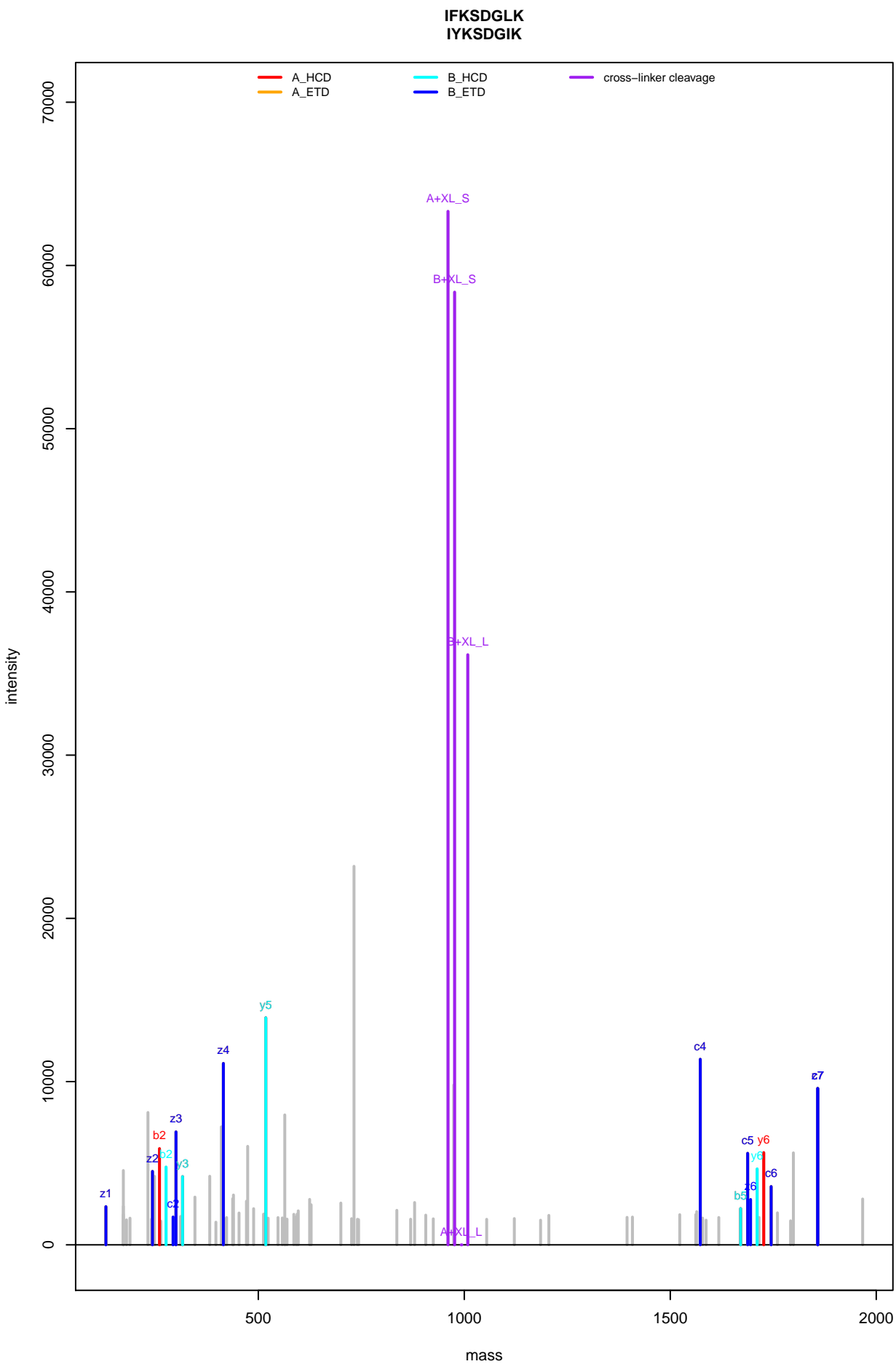

**IFKSDGLK+XL\_S**

**IFKSDGLK+XL\_L**

**IYKSDGIK+XL\_S**

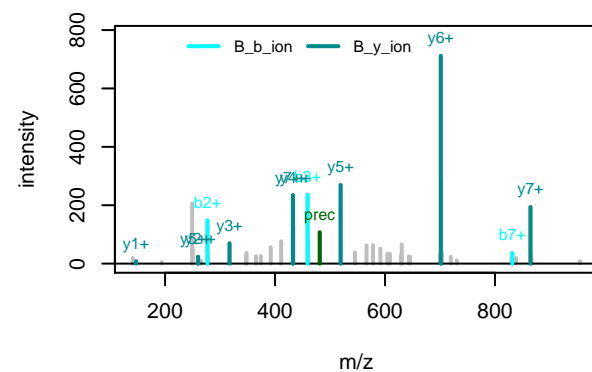

**IYKSDGIK+XL\_L**

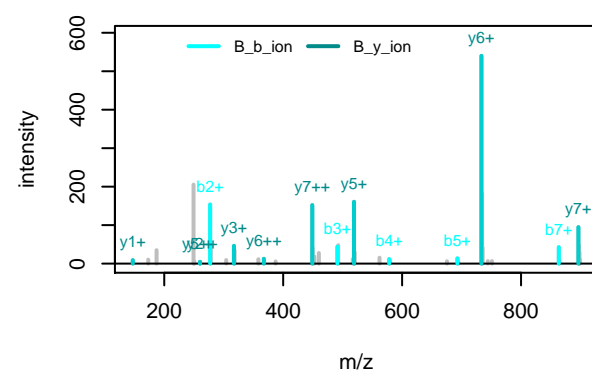

Supplement: Supplemental Data [file supp_RA117.000470_133922_0_supp_23978_fzffwf.zip › spectra_annotation/mito_DR_spectra_annotation/133-1-12-1-7-1.pdf]

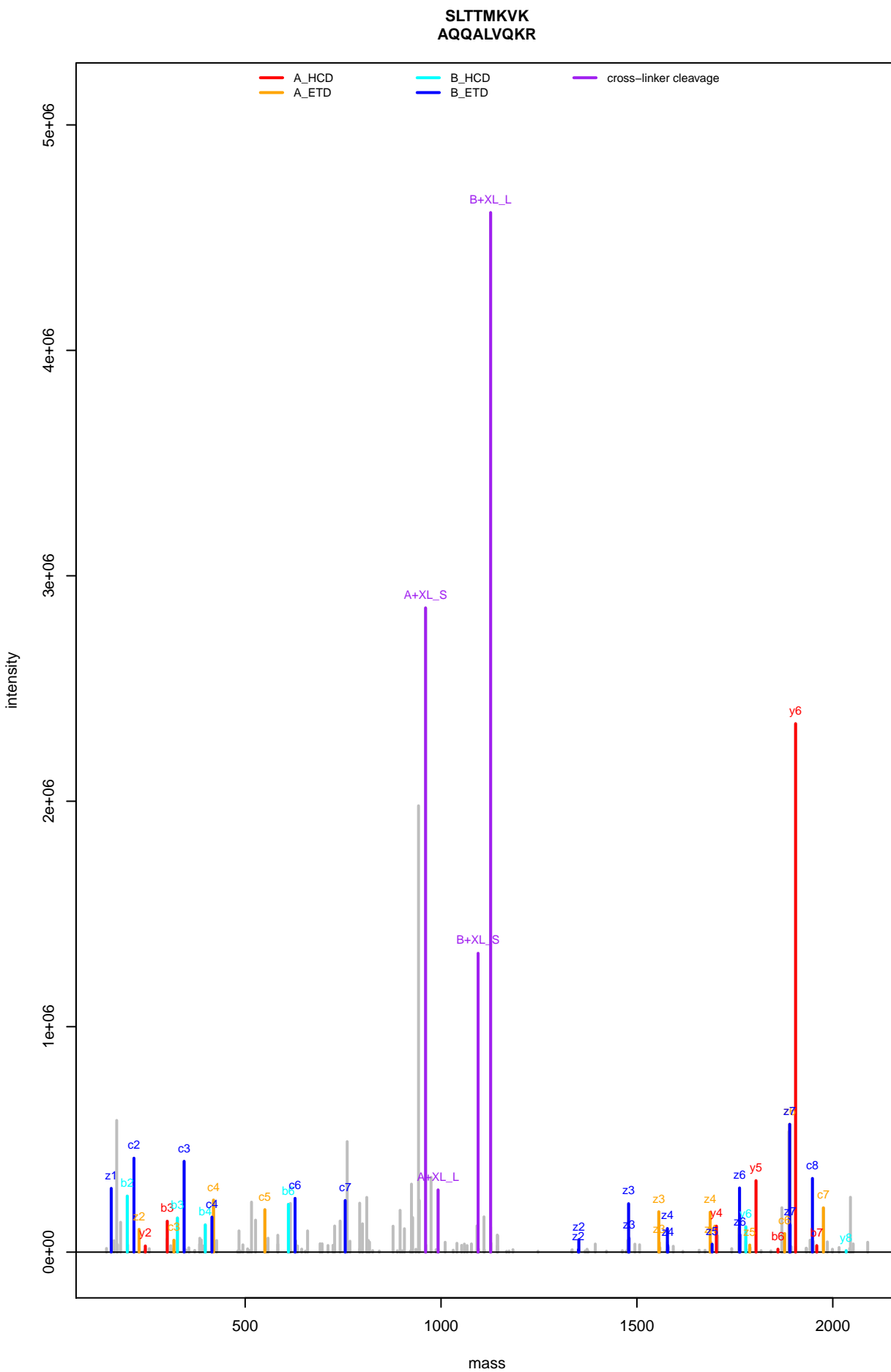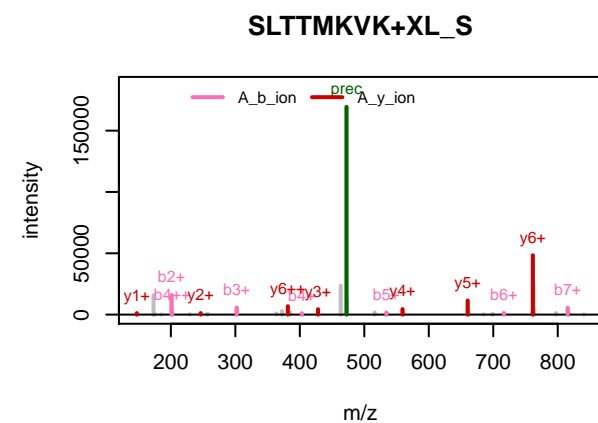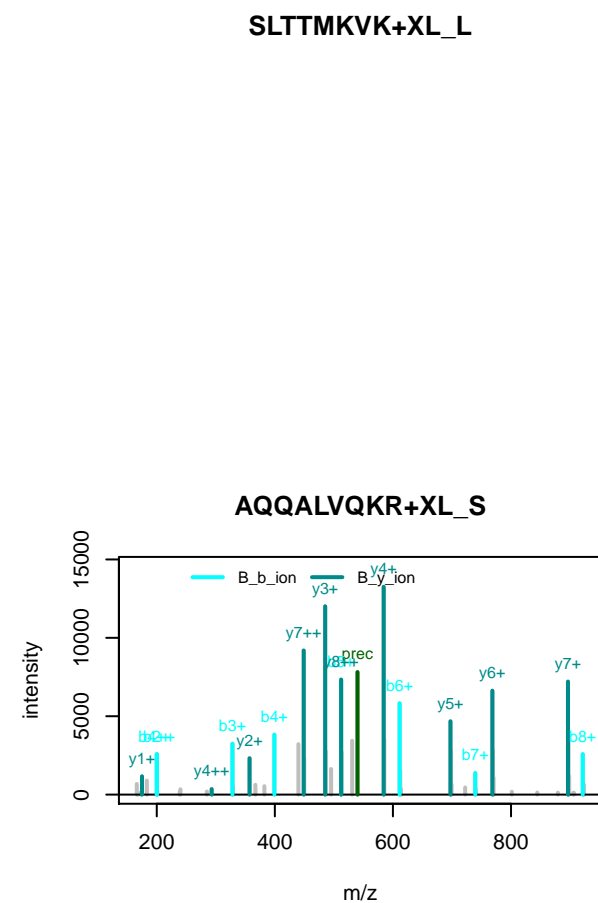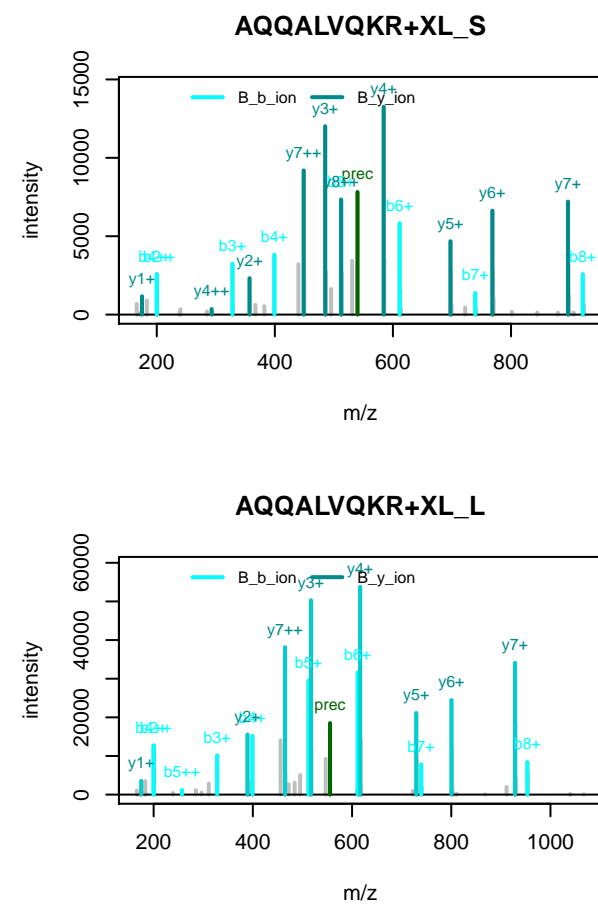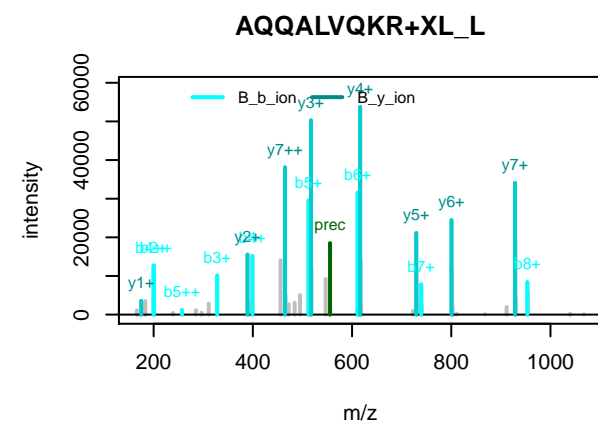

Supplement: Supplemental Data [file supp_RA117.000470_133922_0_supp_23978_fzffwf.zip › spectra_annotation/mito_DR_spectra_annotation/133-1-3-1-3-1.pdf]

TEFKANA EK  
SDGKISEQSDAK

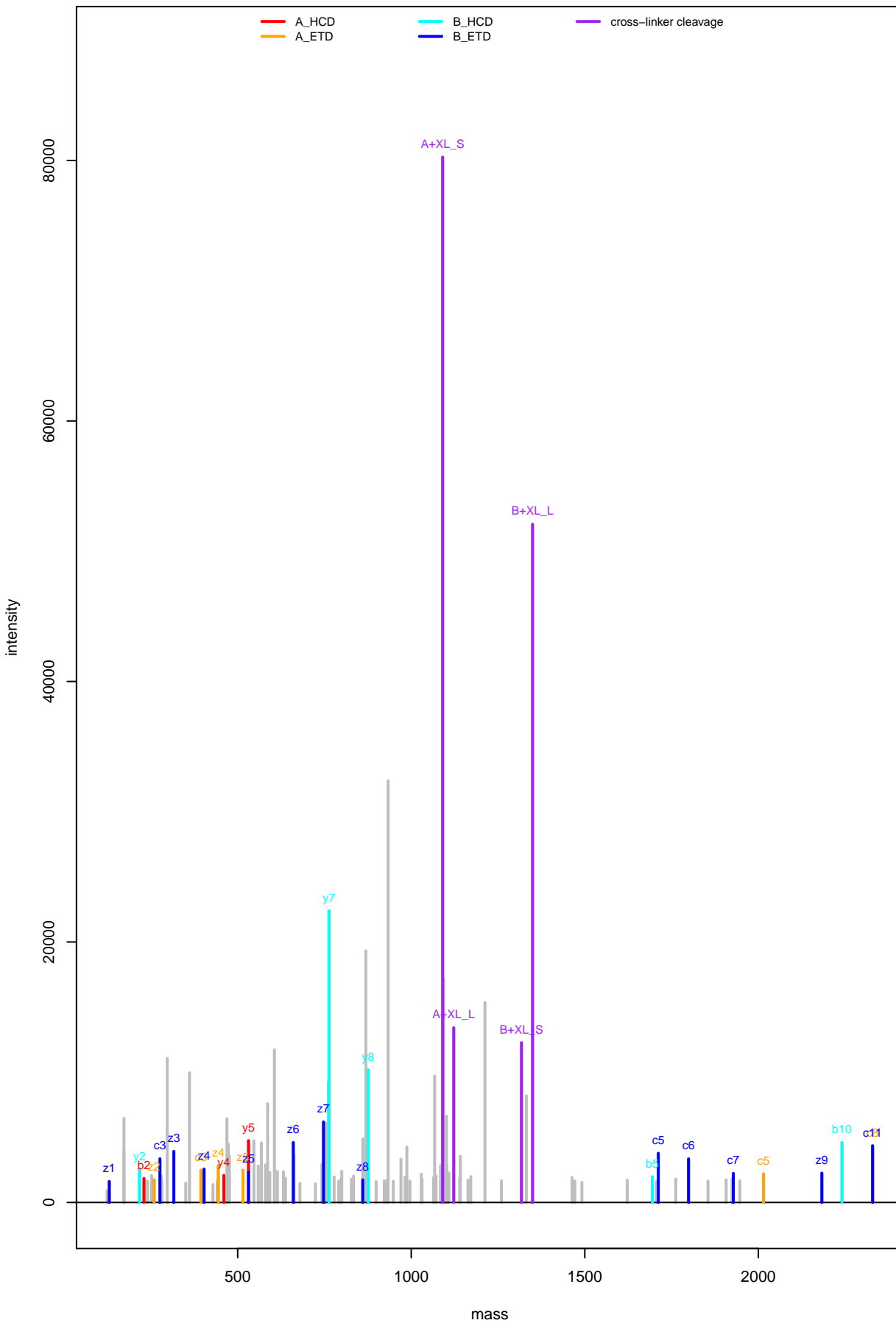

TEFKANA EK+XL\_S

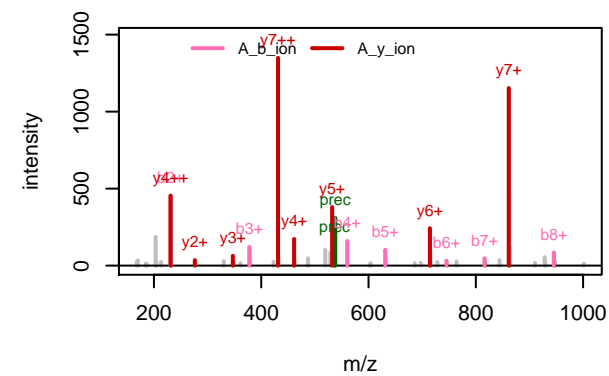

TEFKANA EK+XL\_L

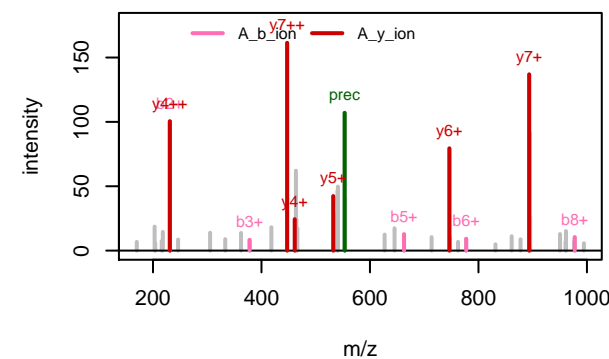

SDGKISEQSDAK+XL\_S

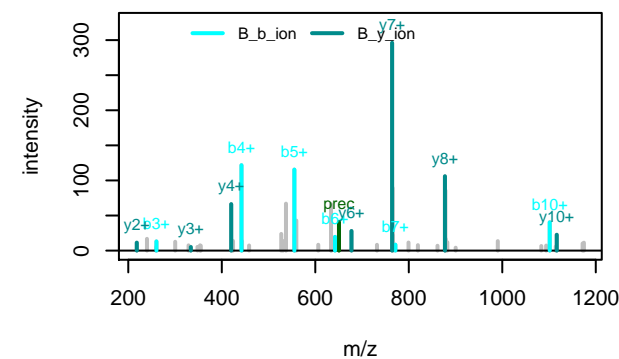

SDGKISEQSDAK+XL\_L

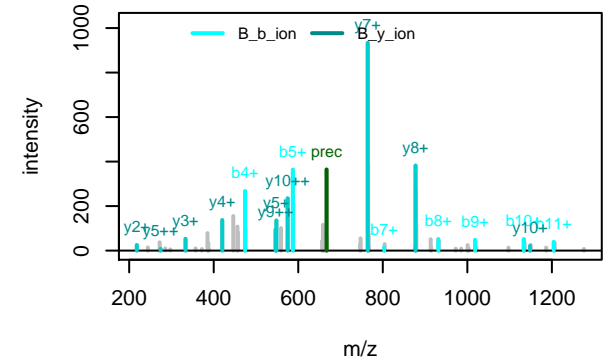

Supplement: Supplemental Data [file supp_RA117.000470_133922_0_supp_23978_fzffwf.zip › spectra_annotation/mito_DR_spectra_annotation/133-1-3-1-6-1.pdf]
