# Supplementary figures and images for: The interactome of intact mitochondria by cross-linking mass spectrometry provides evidence for coexisting respiratory supercomplexes (part 1 of 2)
Source: Mol Cell Proteomics. 2017 Dec 8;17(2):216–32. doi: 10.1074/mcp.RA117.000470 (PMC5795388; doi:10.1074/mcp.RA117.000470)

KIARDEGSK  
LAADVKGSSQR

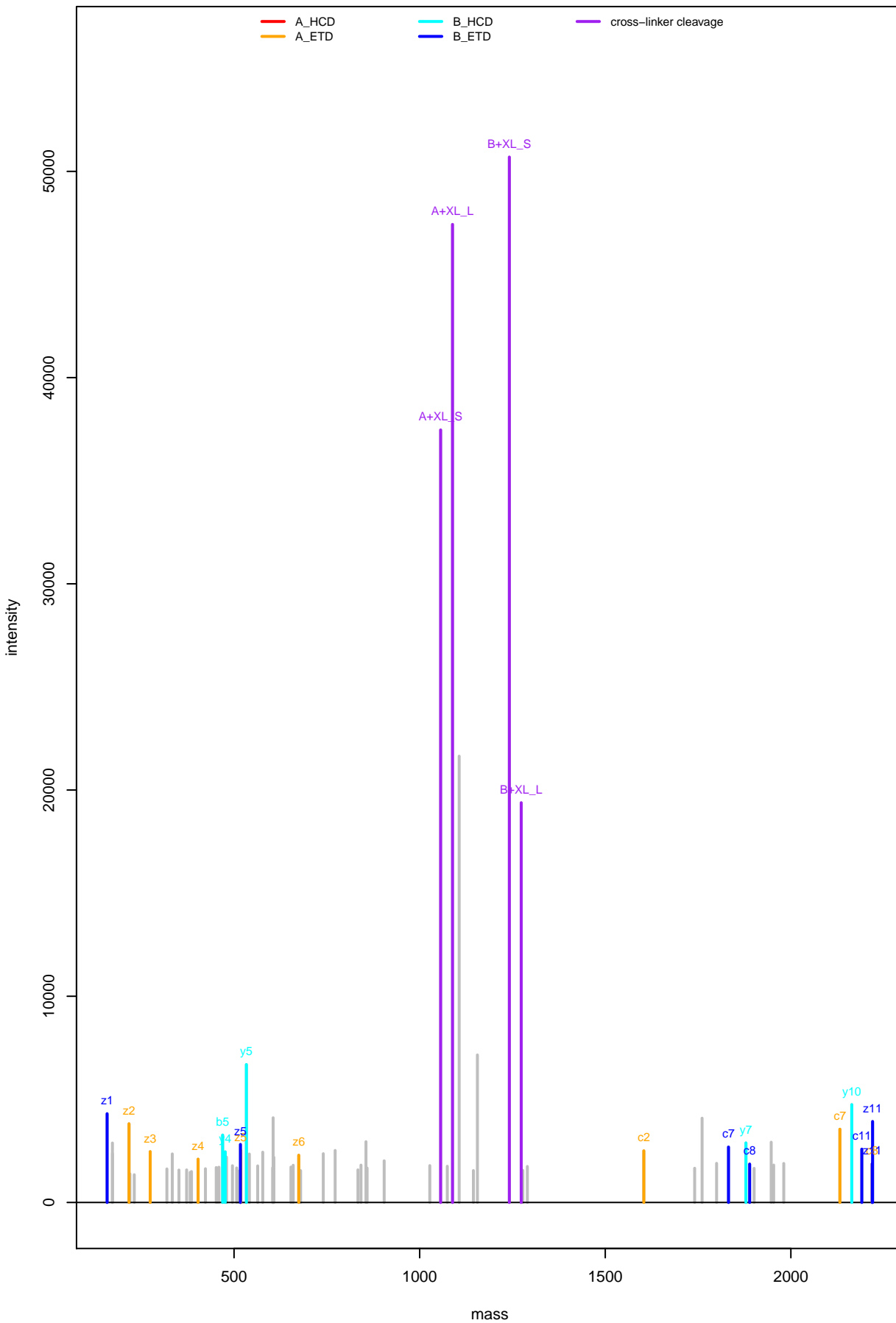

KIARDEGSK+XL\_S

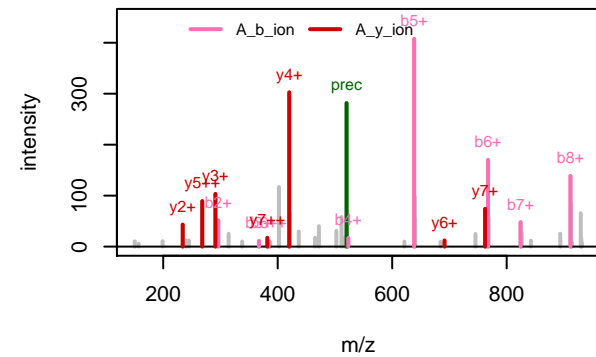

KIARDEGSK+XL\_L

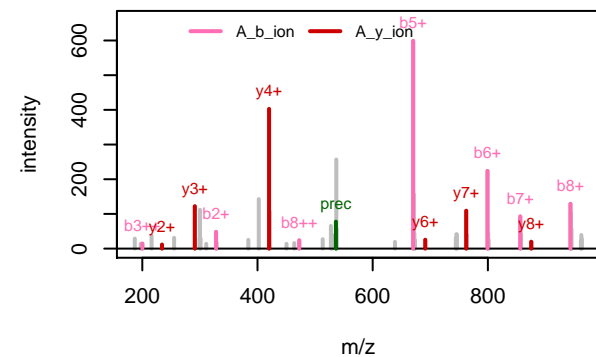

LAADVKGSSQR+XL\_S

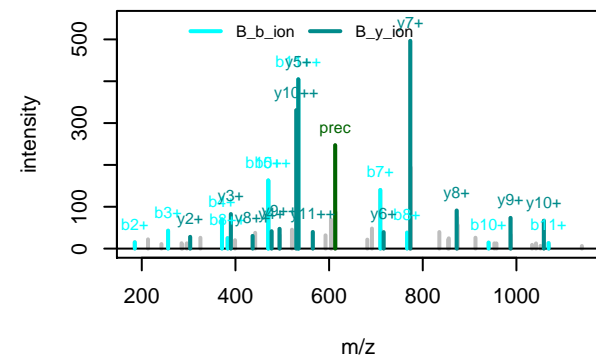

LAADVKGSSQR+XL\_L

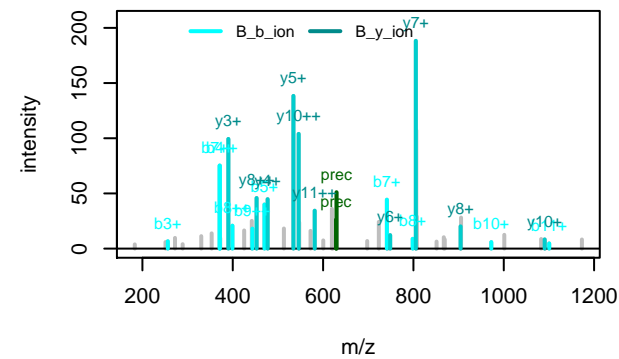

Supplement: Supplemental Data [file supp_RA117.000470_133922_0_supp_23978_fzffwf.zip › spectra_annotation/mito_DR_spectra_annotation/1-1-15-1-7-1.pdf]

LEPSKITK  
MVAAAKYAR

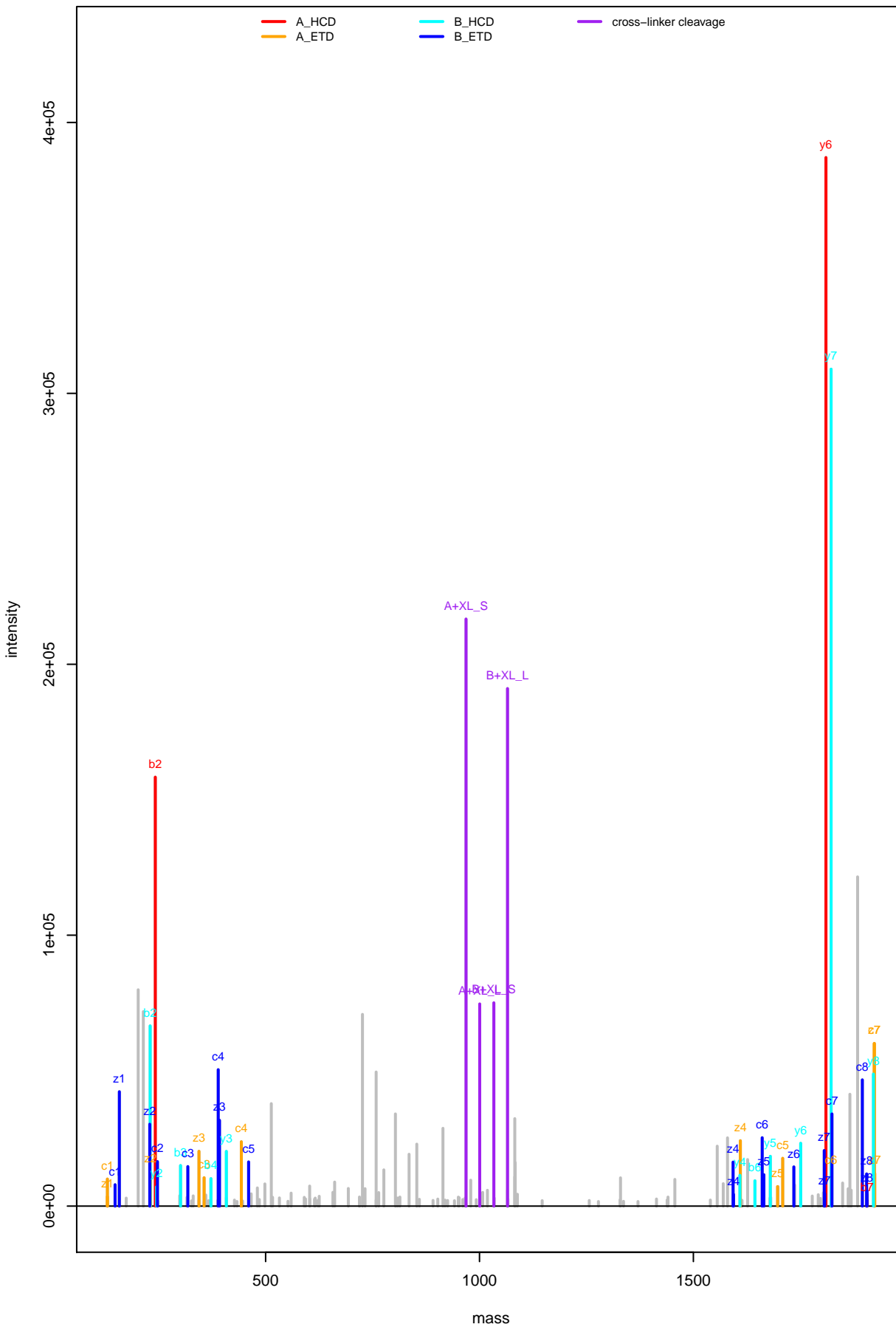

LEPSKITK+XL\_S

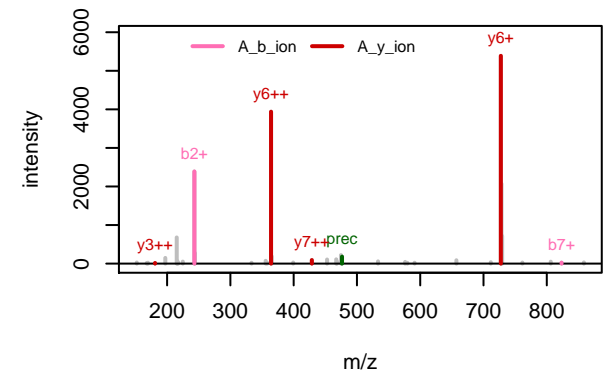

LEPSKITK+XL\_L

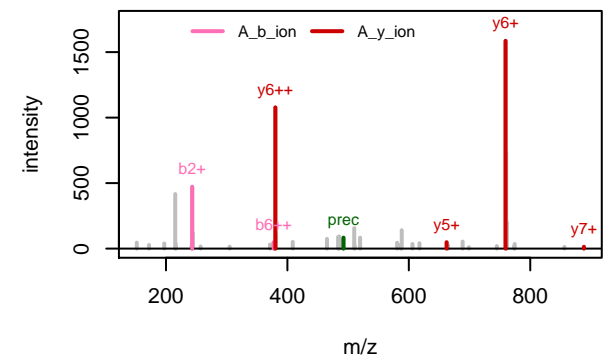

MVAAAKYAR+XL\_S

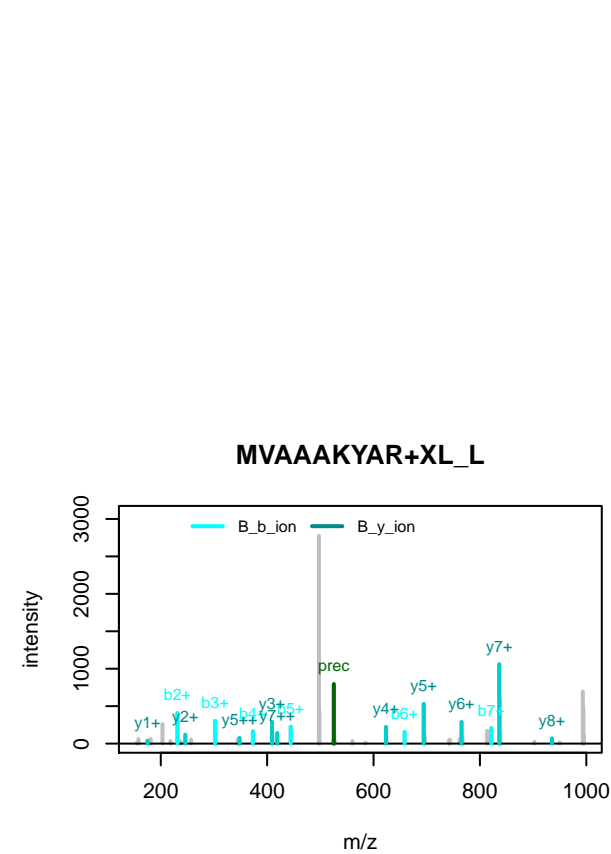

Supplement: Supplemental Data [file supp_RA117.000470_133922_0_supp_23978_fzffwf.zip › spectra_annotation/mito_DR_spectra_annotation/1-1-19-1-5-1.pdf]

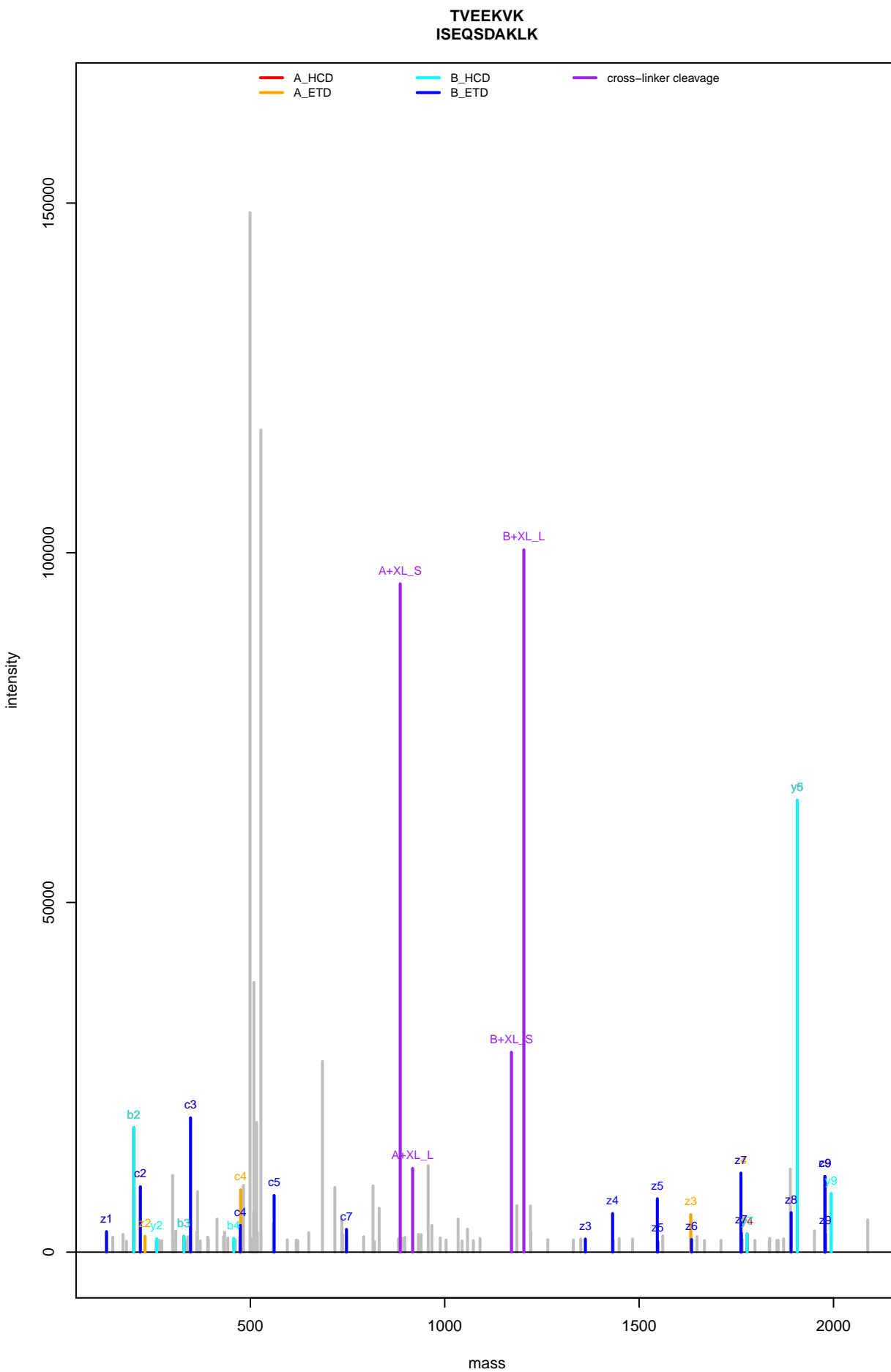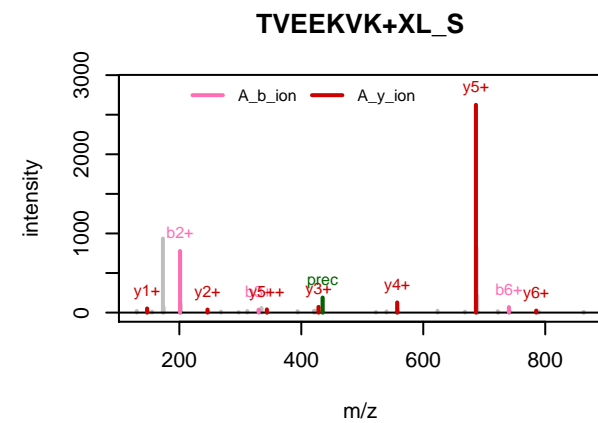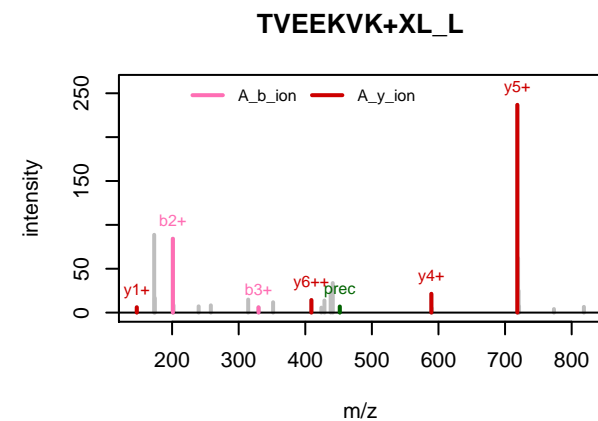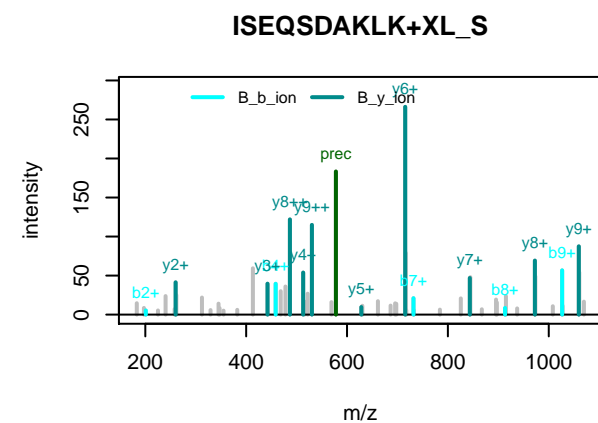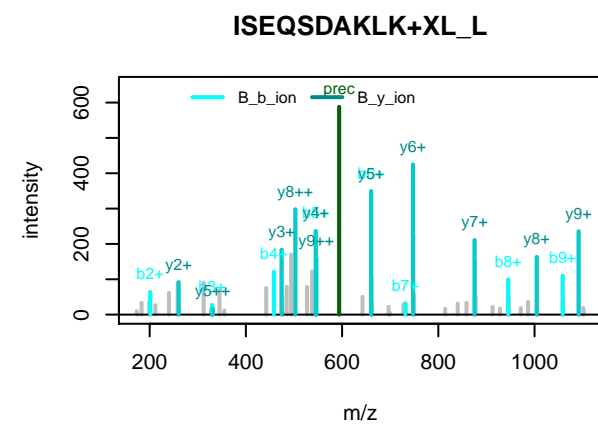

Supplement: Supplemental Data [file supp_RA117.000470_133922_0_supp_23978_fzffwf.zip › spectra_annotation/mito_DR_spectra_annotation/1-1-8-1-12-1.pdf]

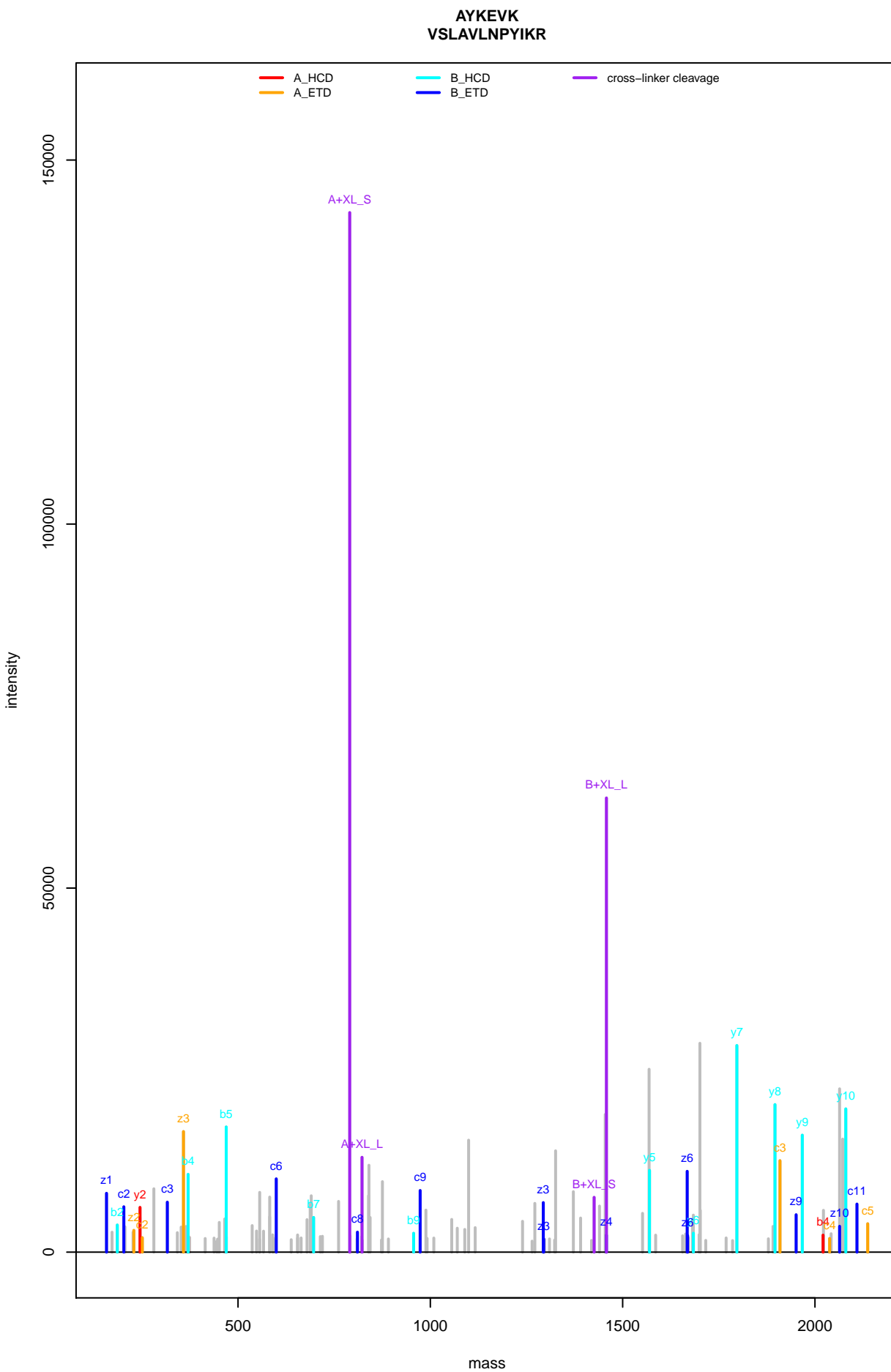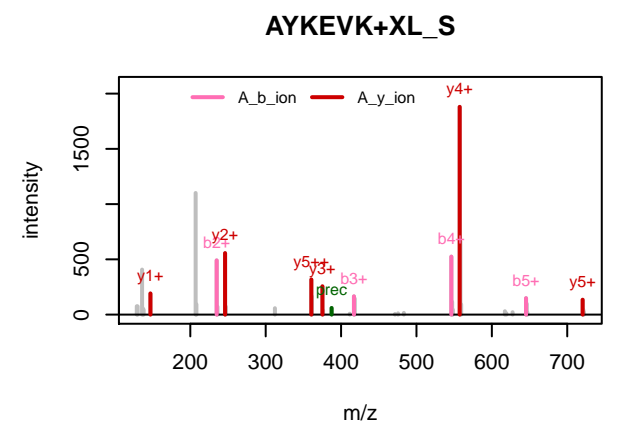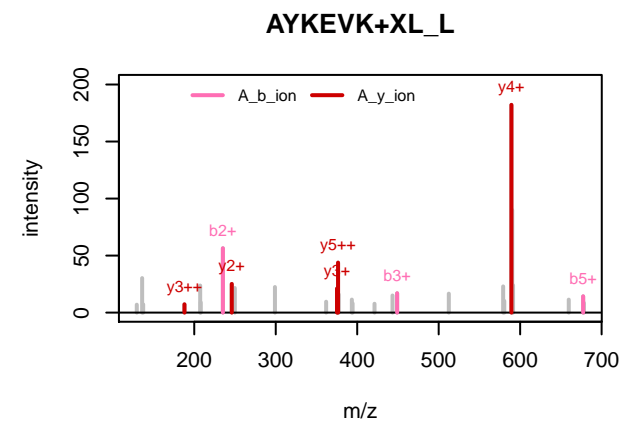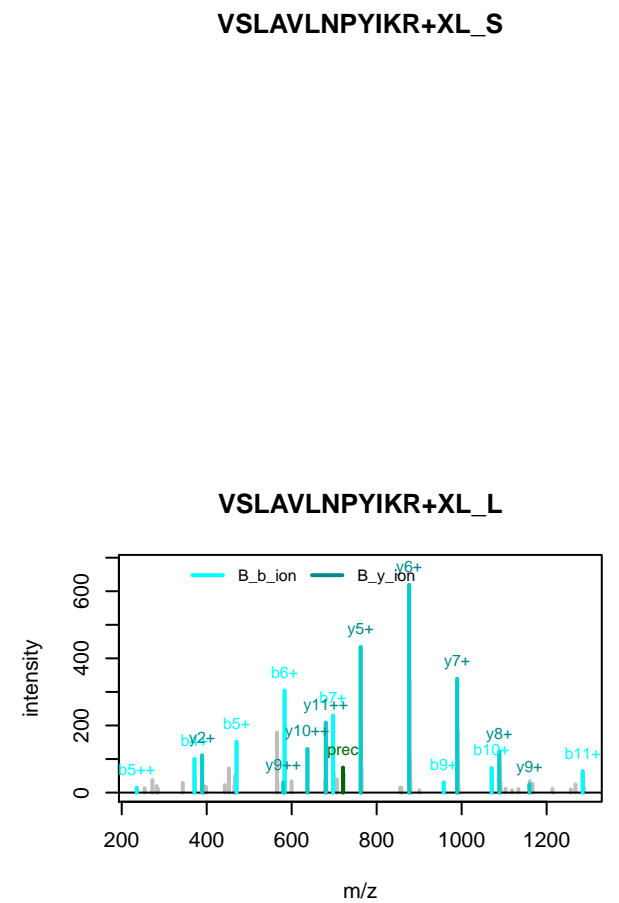

Supplement: Supplemental Data [file supp_RA117.000470_133922_0_supp_23978_fzffwf.zip › spectra_annotation/mito_DR_spectra_annotation/10-1-2-1-6-1.pdf]

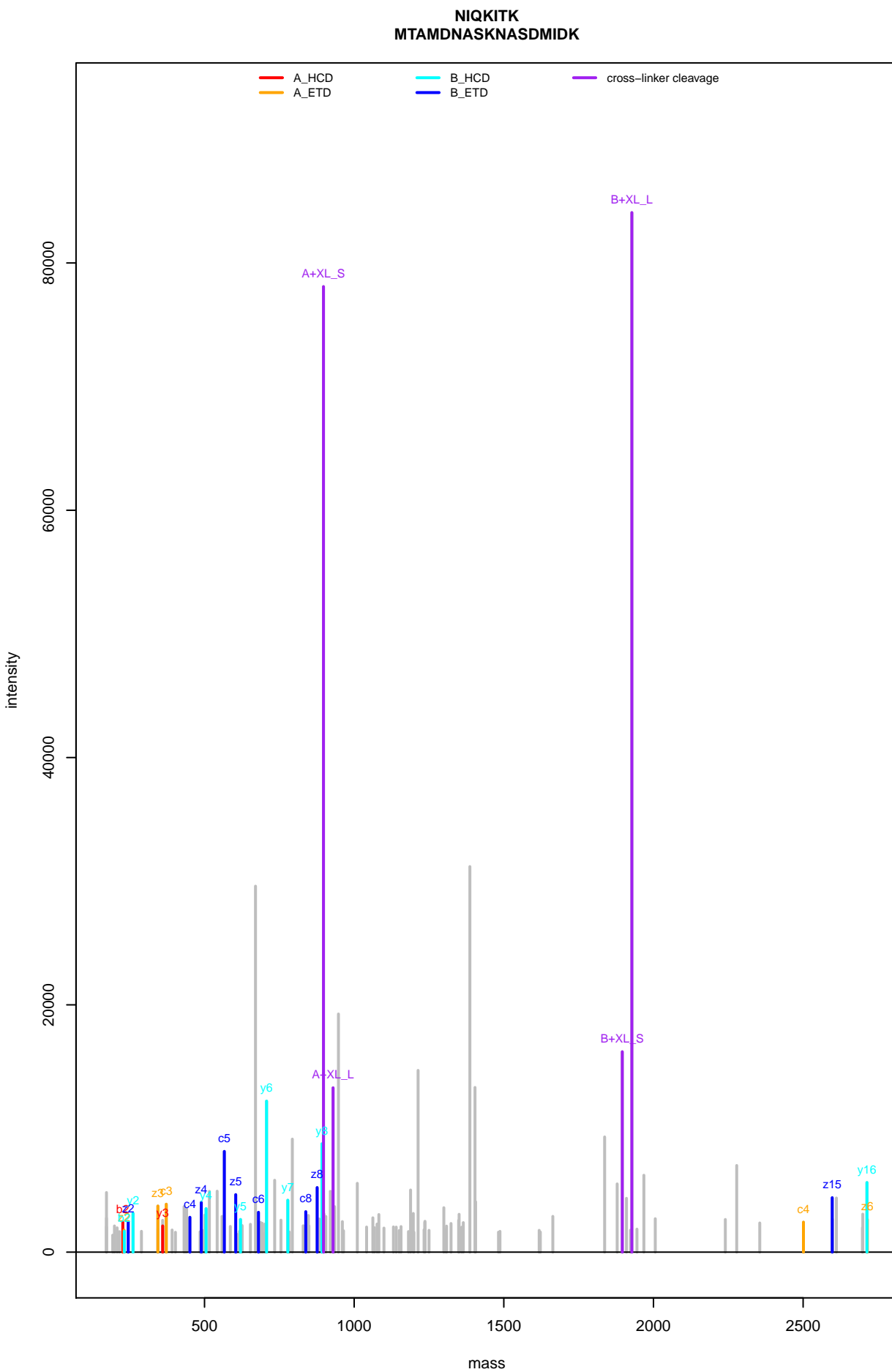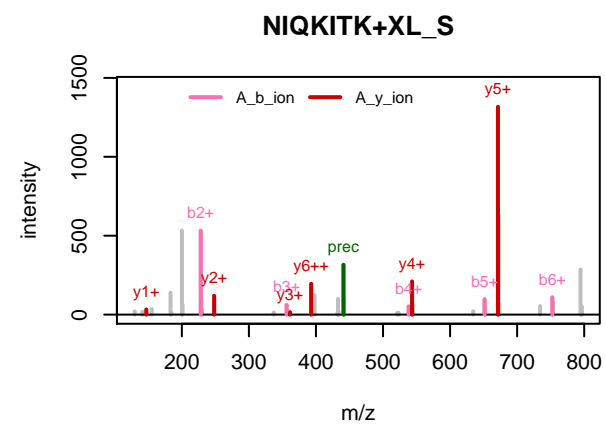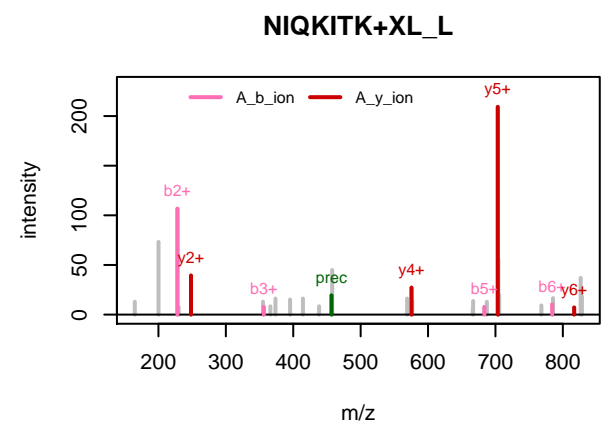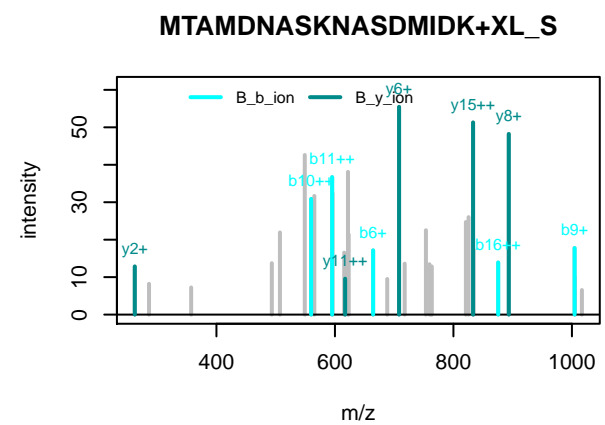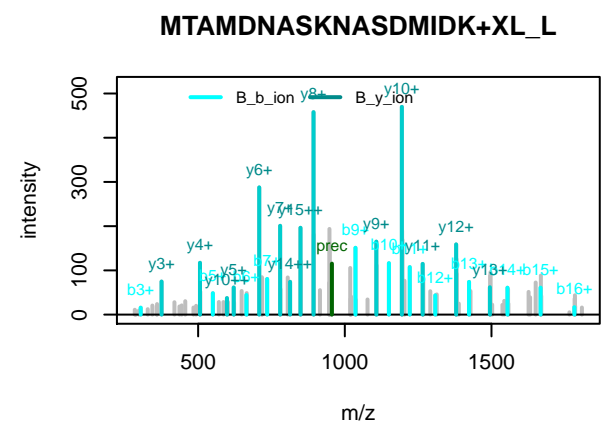

Supplement: Supplemental Data [file supp_RA117.000470_133922_0_supp_23978_fzffwf.zip › spectra_annotation/mito_DR_spectra_annotation/100-1-2-1-1-1.pdf]

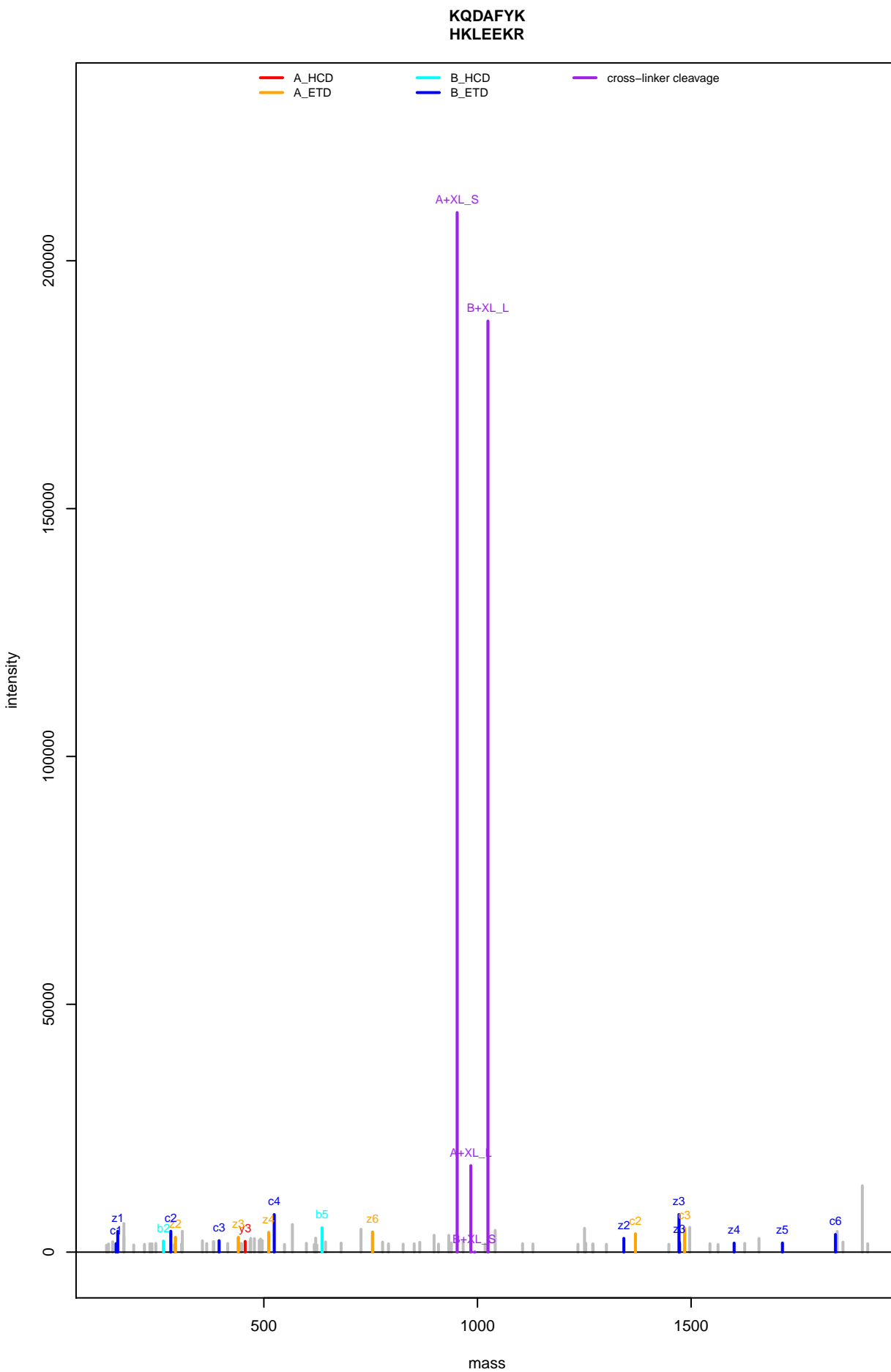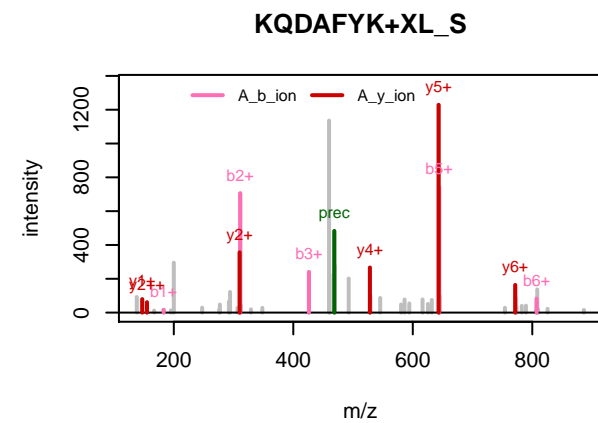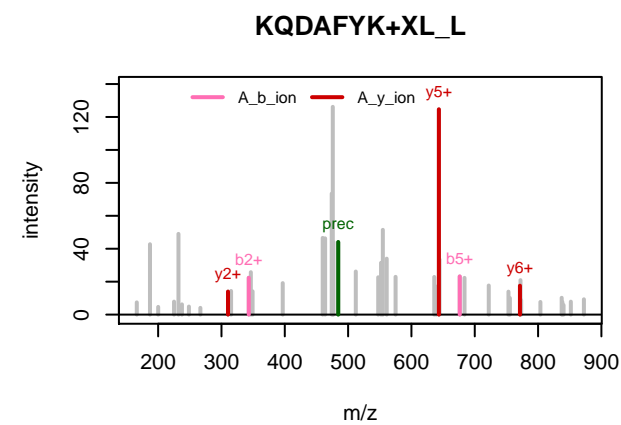

**HKLEEK+XL\_S**

**HKLEEK+XL\_L**

Supplement: Supplemental Data [file supp_RA117.000470_133922_0_supp_23978_fzffwf.zip › spectra_annotation/mito_DR_spectra_annotation/100-1-4-1-5-1.pdf]

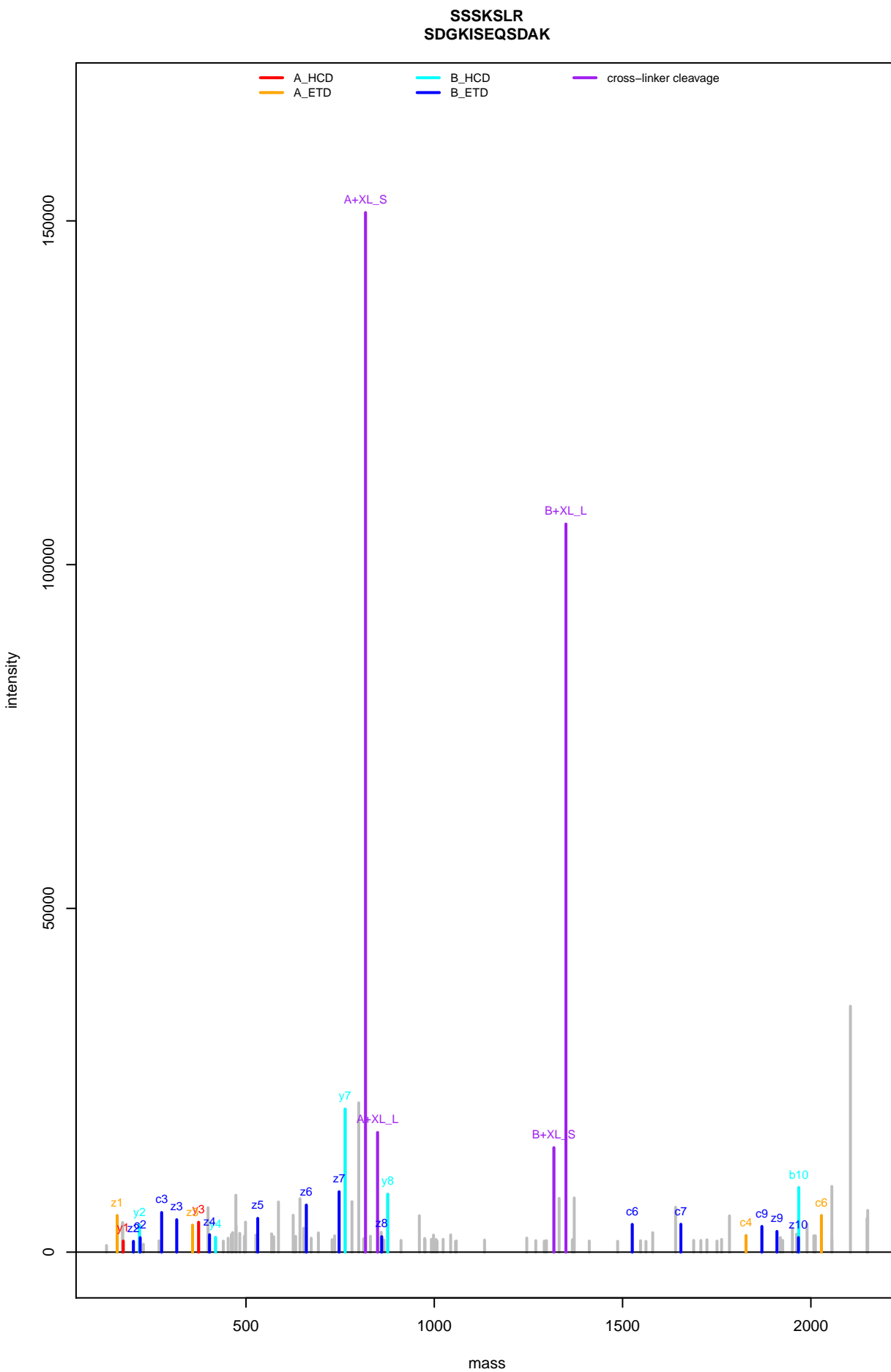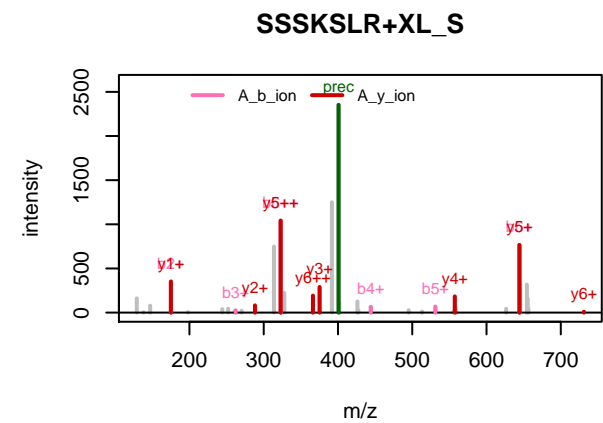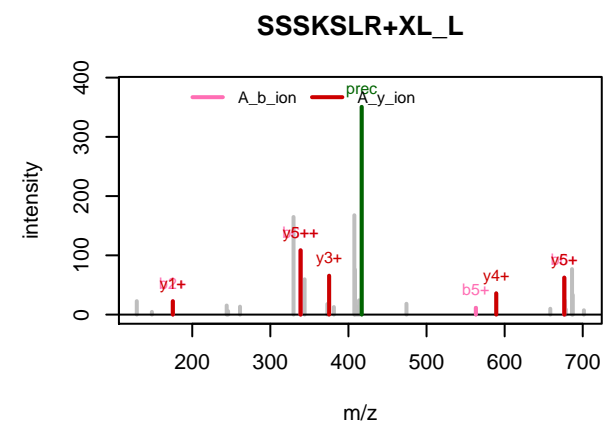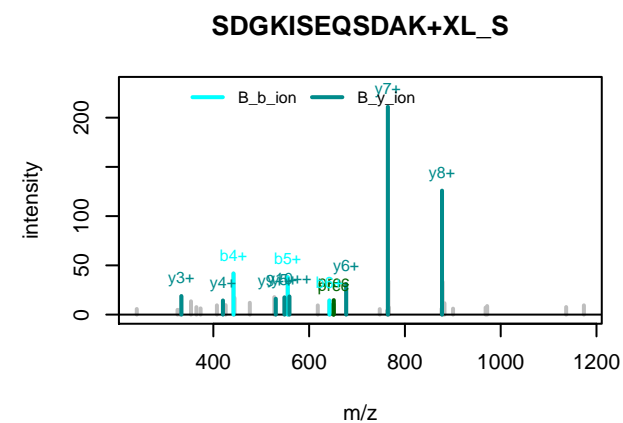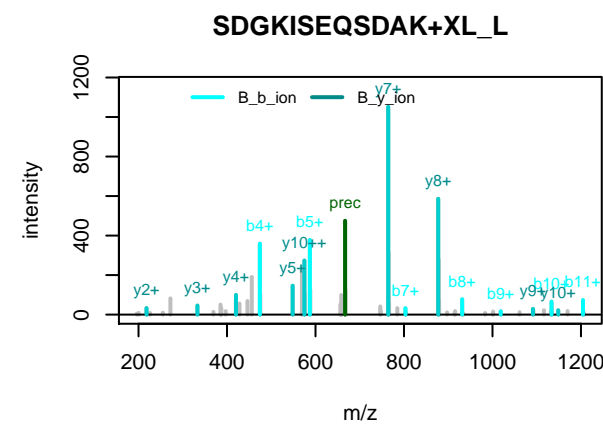

Supplement: Supplemental Data [file supp_RA117.000470_133922_0_supp_23978_fzffwf.zip › spectra_annotation/mito_DR_spectra_annotation/100-1-6-1-6-1.pdf]

# IKREQK LKIWYRR

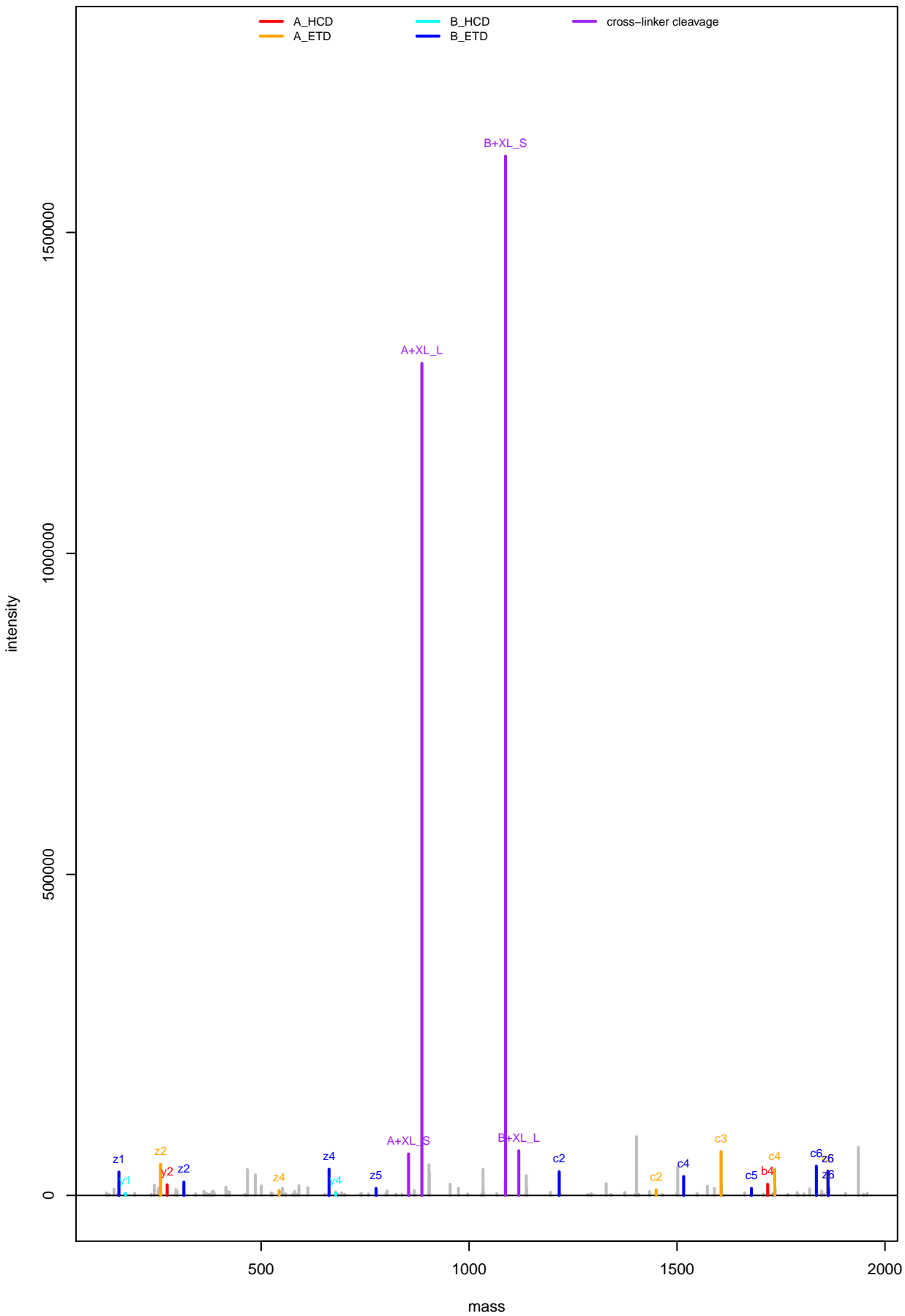

## IKREQK+XL\_S

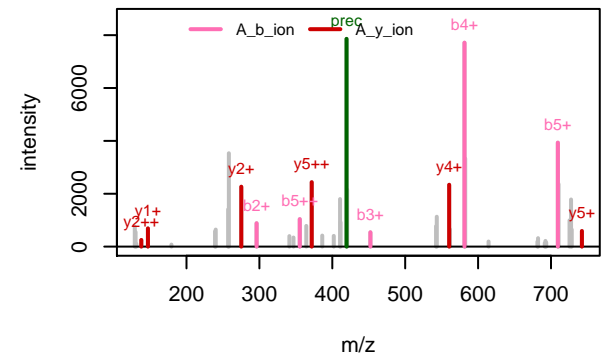

## IKREQK+XL\_L

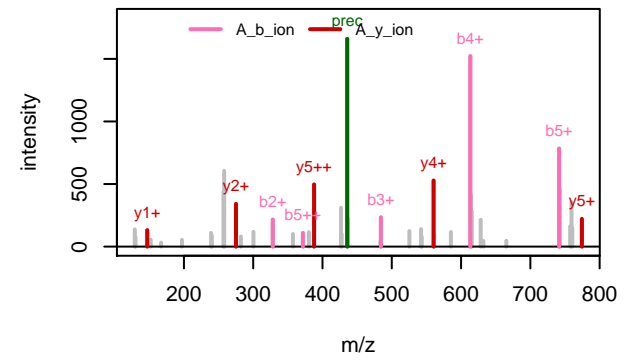

## LKIWYRR+XL\_S

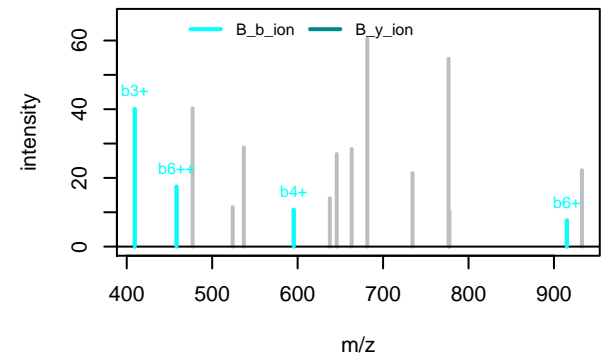

## LKIWYRR+XL\_L

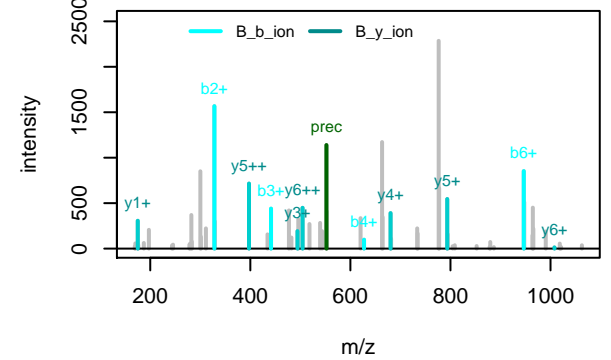

Supplement: Supplemental Data [file supp_RA117.000470_133922_0_supp_23978_fzffwf.zip › spectra_annotation/mito_DR_spectra_annotation/100-1-9-1-1-1.pdf]

**KNNPEPWNK  
YEEDKFYLEPYLK**

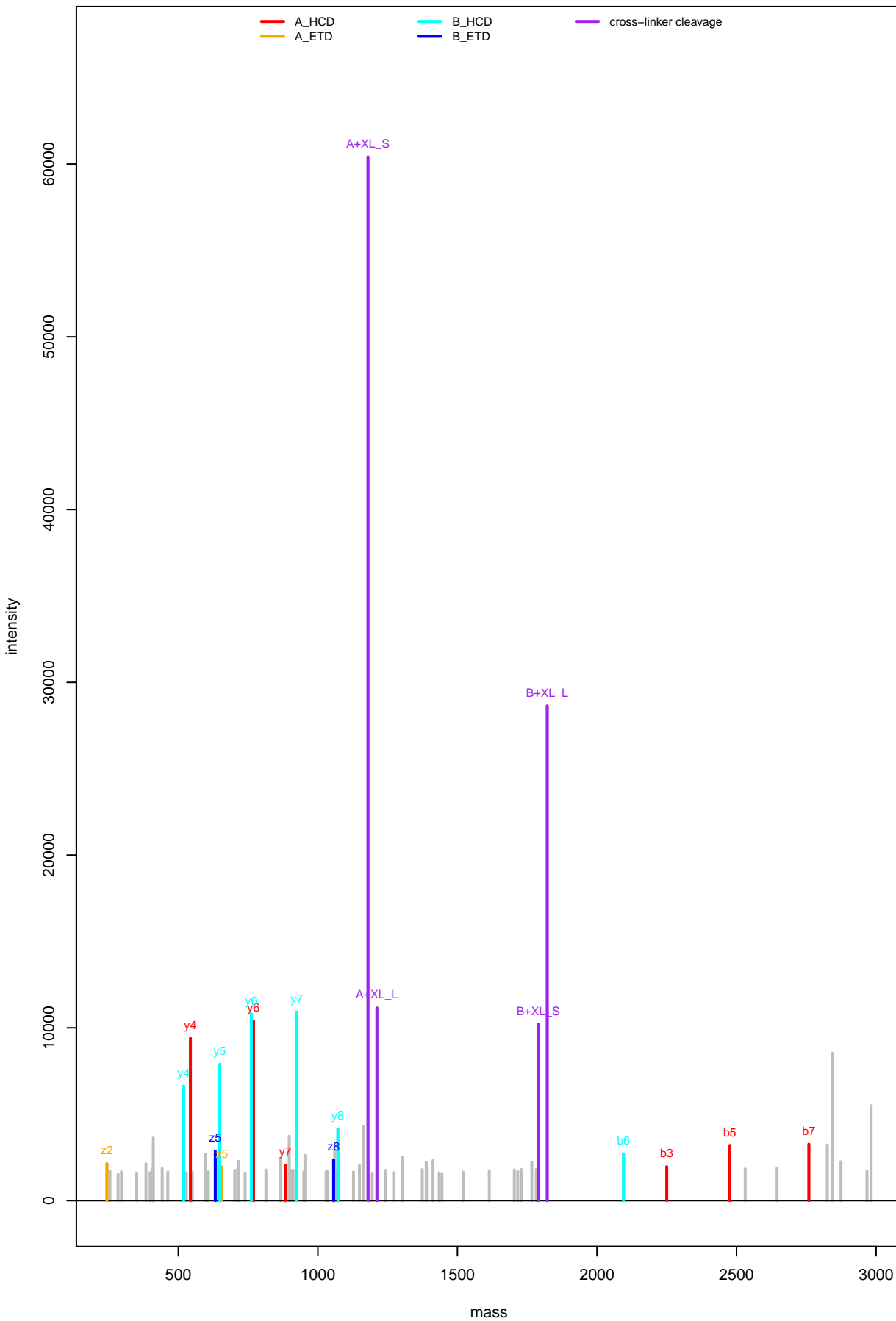

**KNNPEPWNK+XL\_S**

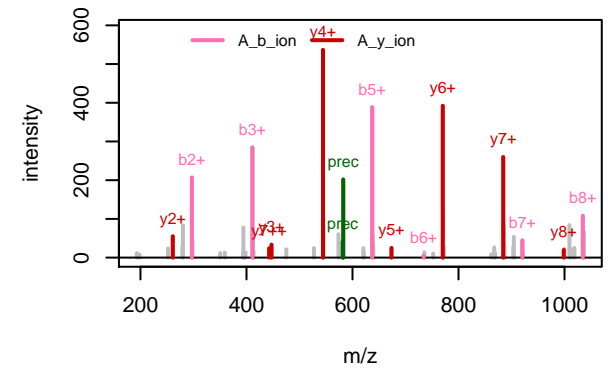

**KNNPEPWNK+XL\_L**

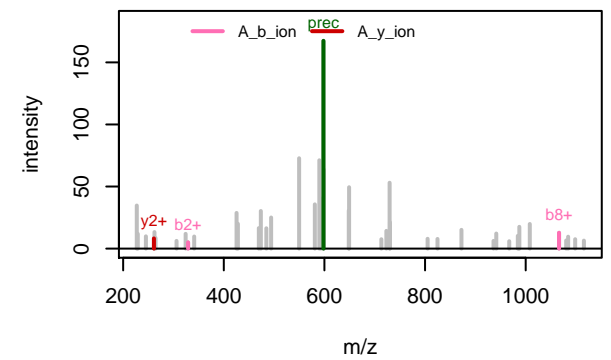

**YEEDKFYLEPYLK+XL\_S**

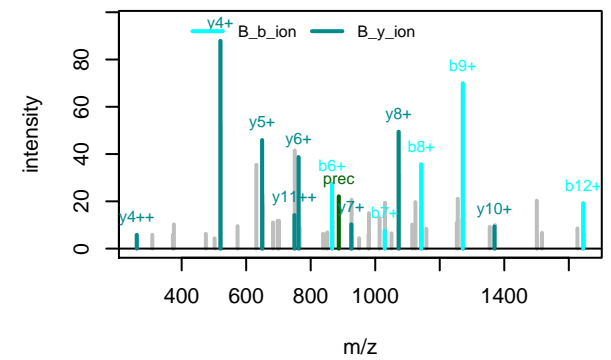

**YEEDKFYLEPYLK+XL\_L**

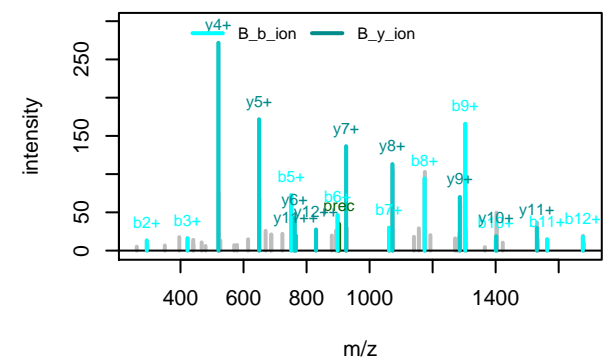

Supplement: Supplemental Data [file supp_RA117.000470_133922_0_supp_23978_fzffwf.zip › spectra_annotation/mito_DR_spectra_annotation/101-1-1-1-7-1.pdf]

# LEPSKITK SDGKISEQSDAK

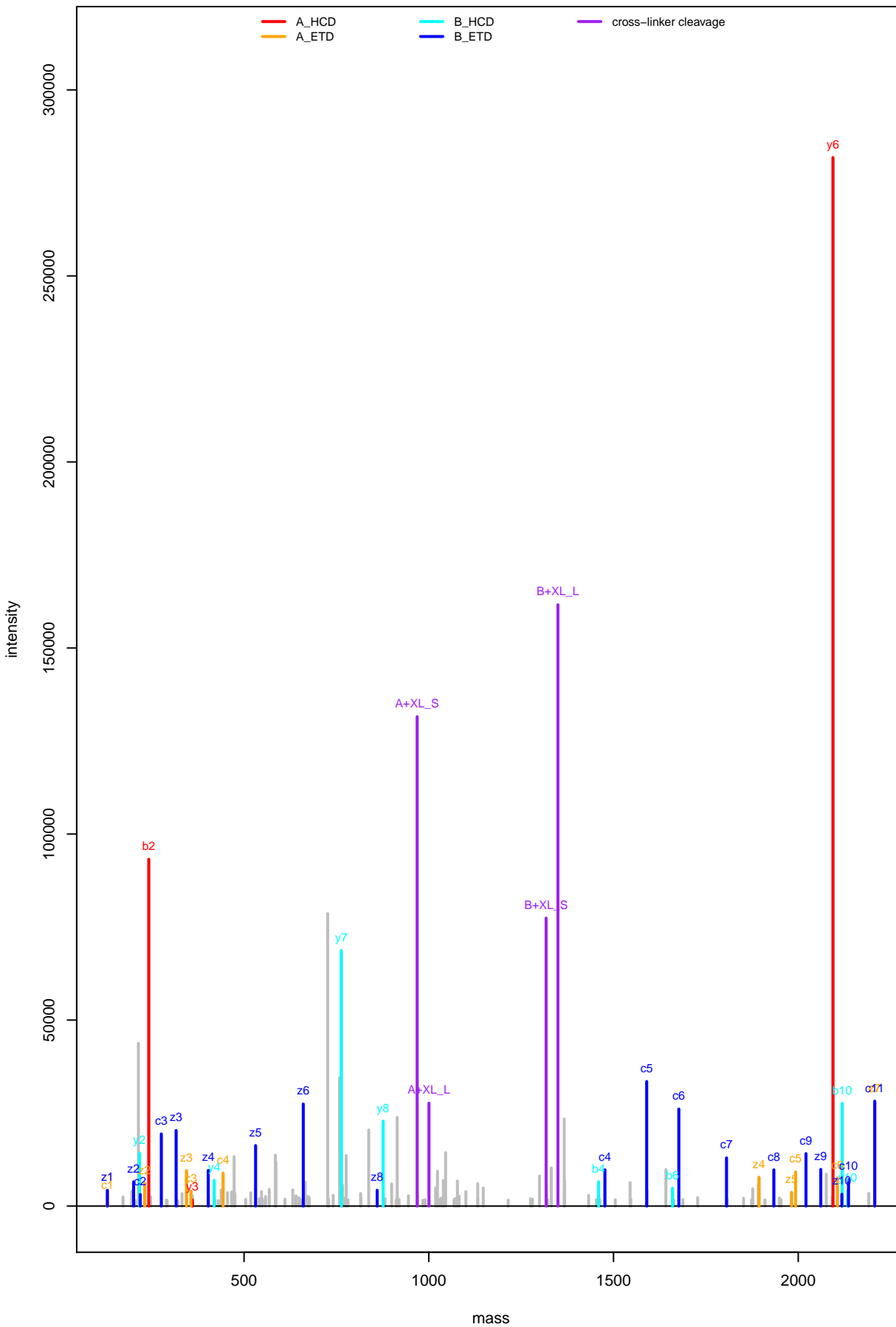

## LEPSKITK+XL\_S

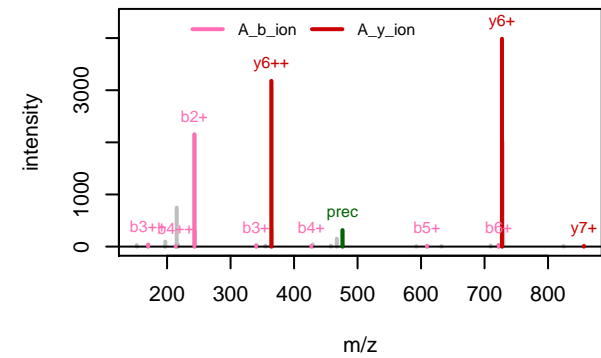

## LEPSKITK+XL\_L

## SDGKISEQSDAK+XL\_S

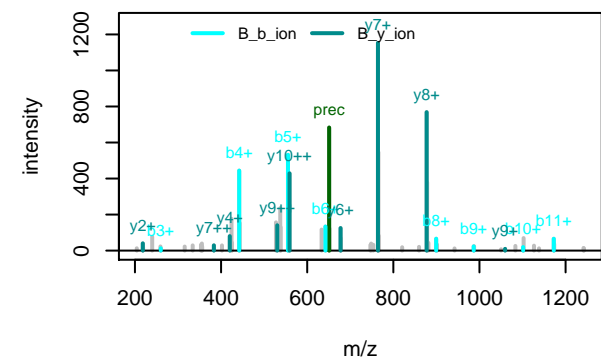

## SDGKISEQSDAK+XL\_L

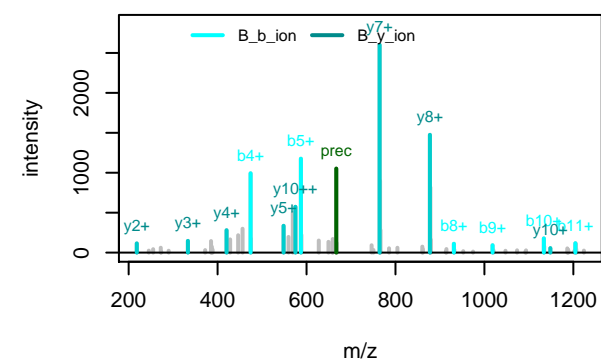

Supplement: Supplemental Data [file supp_RA117.000470_133922_0_supp_23978_fzffwf.zip › spectra_annotation/mito_DR_spectra_annotation/101-1-19-1-6-1.pdf]

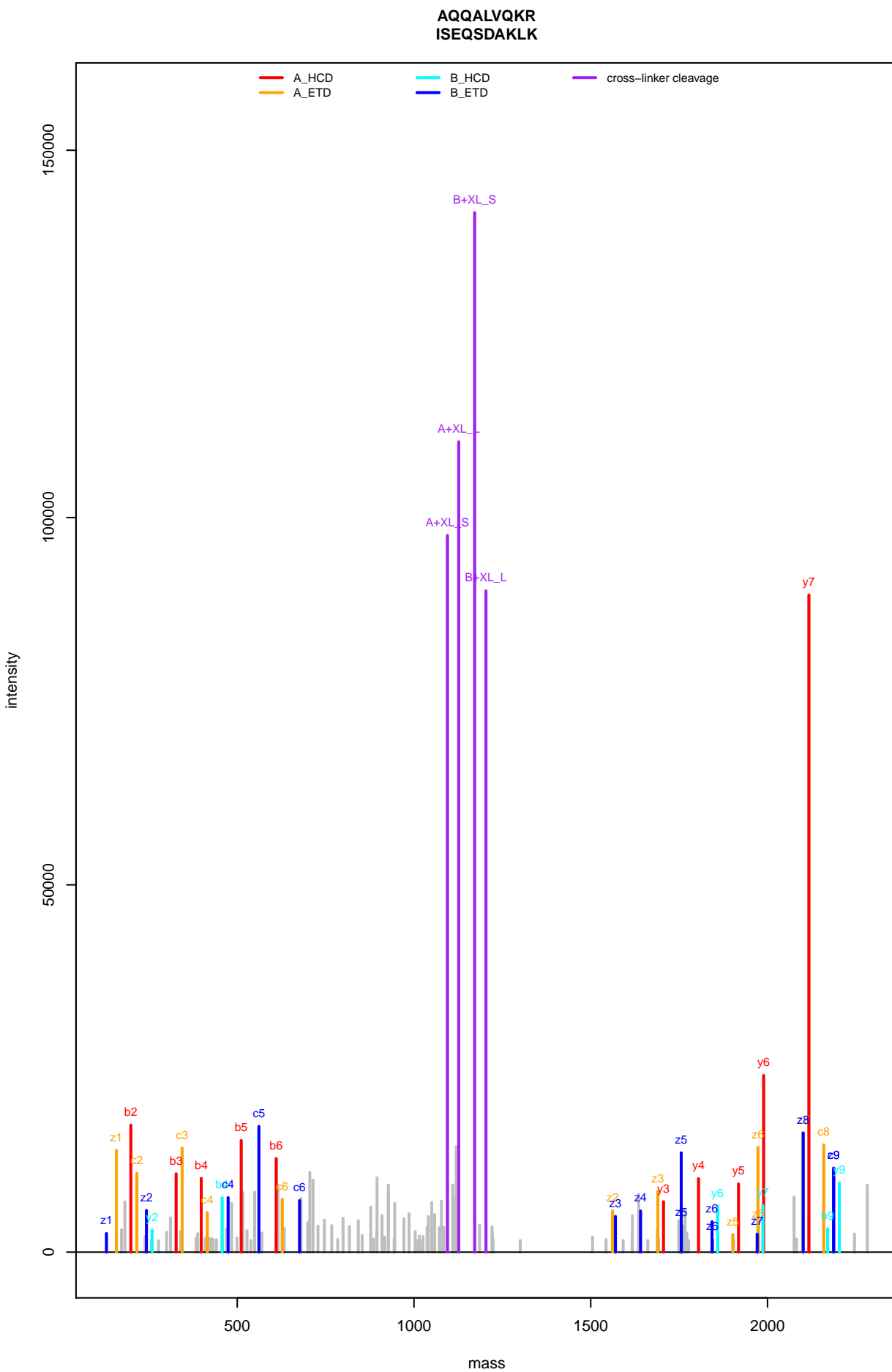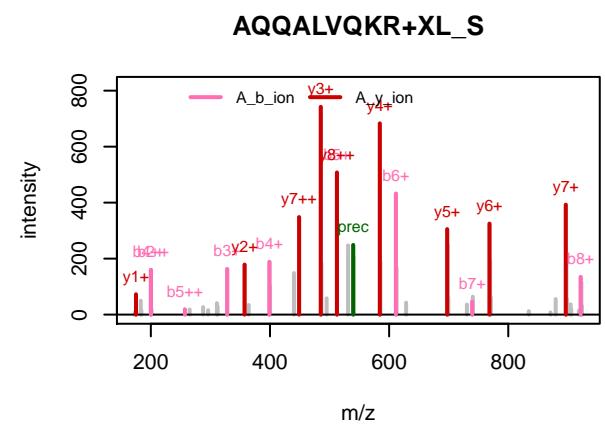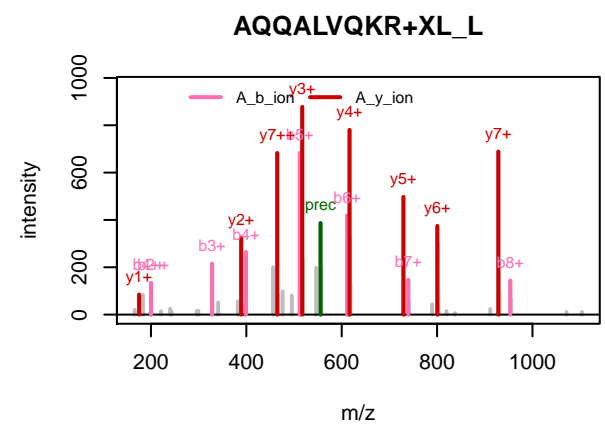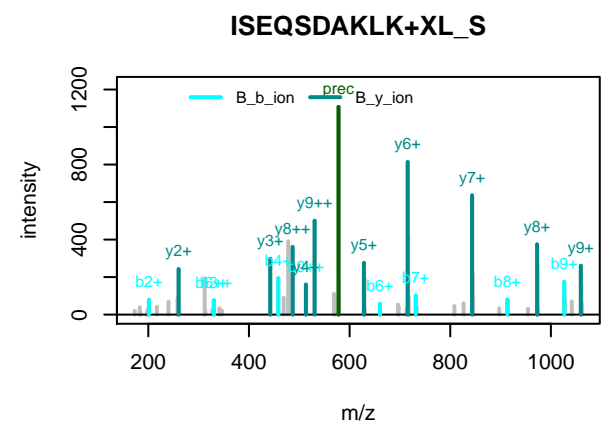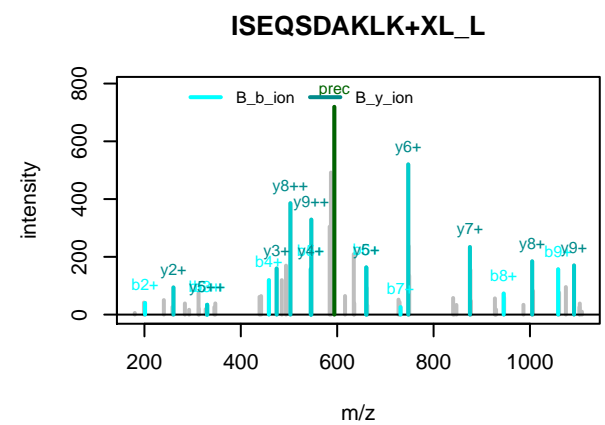

Supplement: Supplemental Data [file supp_RA117.000470_133922_0_supp_23978_fzffwf.zip › spectra_annotation/mito_DR_spectra_annotation/101-1-3-1-12-1.pdf]

HKLEEKR  
KVQAAQSEAK

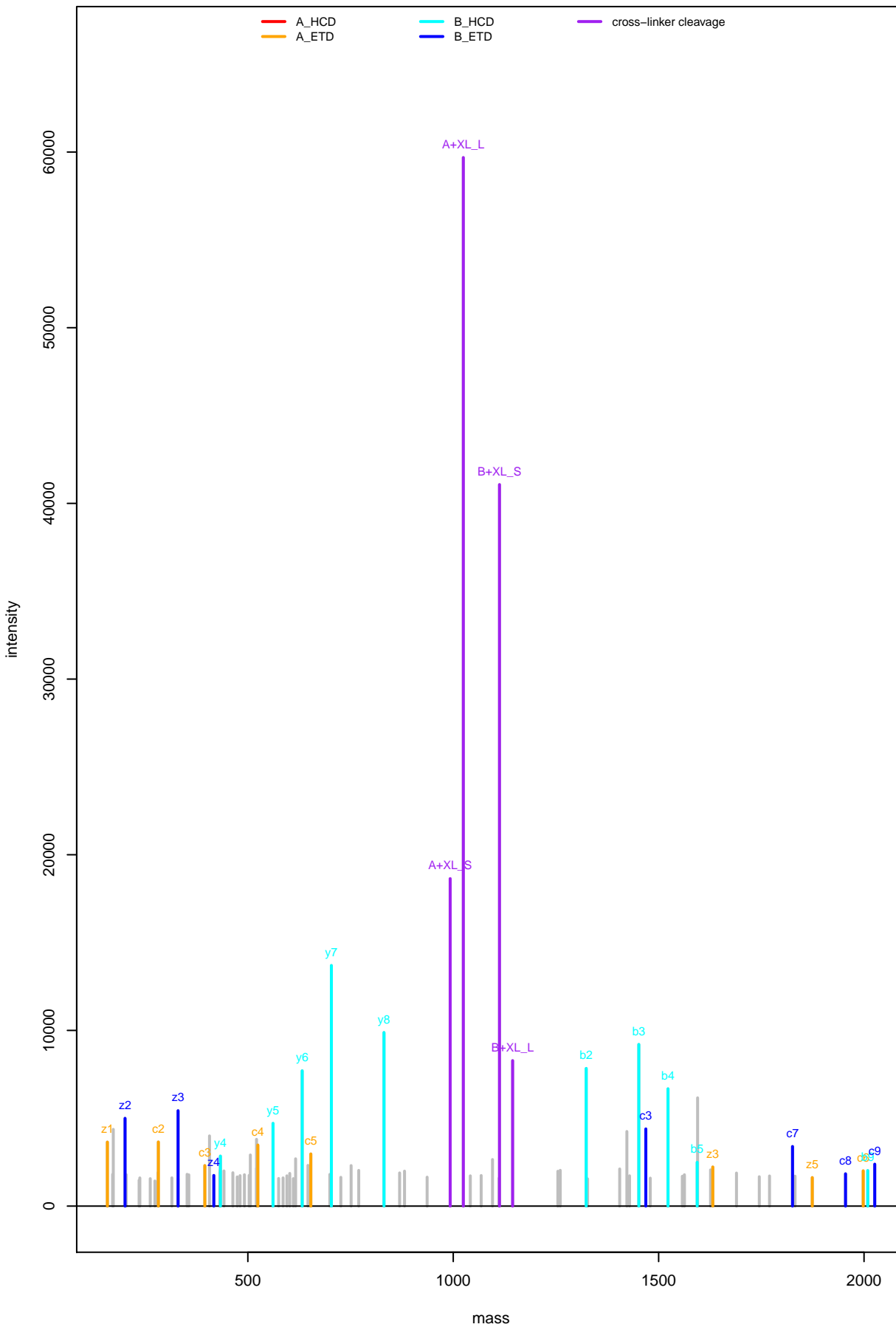

HKLEEKR+XL\_S

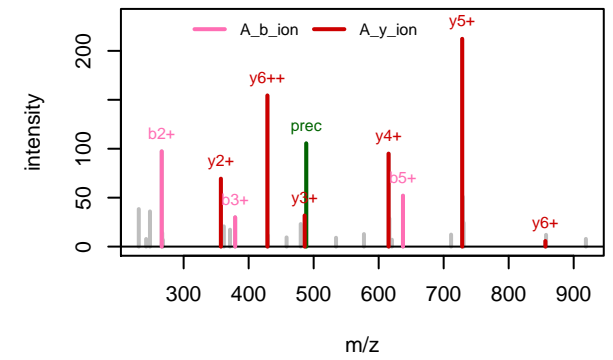

HKLEEKR+XL\_L

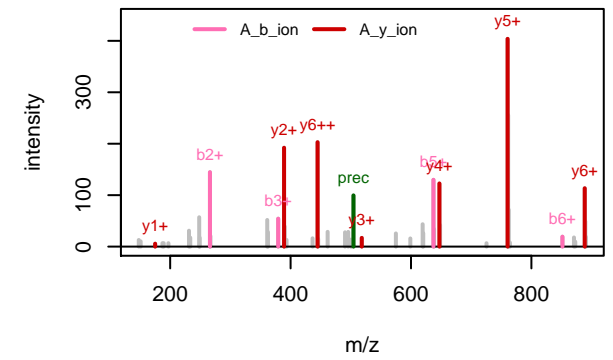

KVQAAQSEAK+XL\_S

KVQAAQSEAK+XL\_L

Supplement: Supplemental Data [file supp_RA117.000470_133922_0_supp_23978_fzffwf.zip › spectra_annotation/mito_DR_spectra_annotation/101-1-5-1-7-1.pdf]

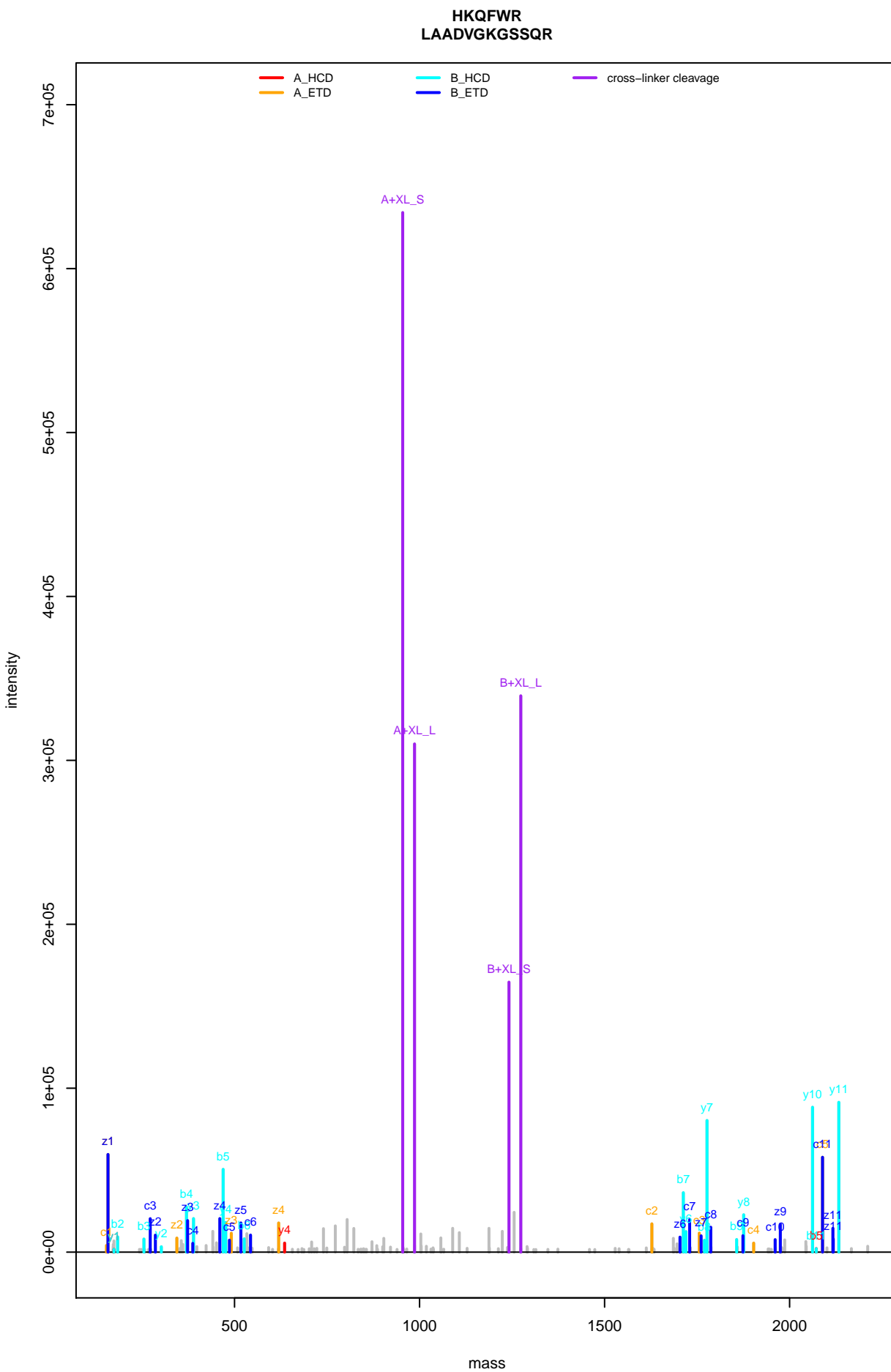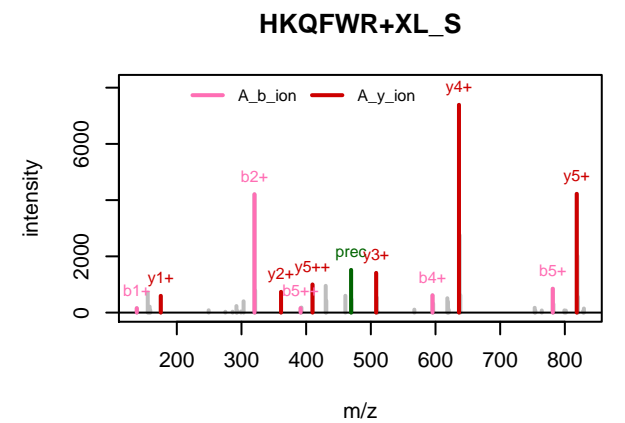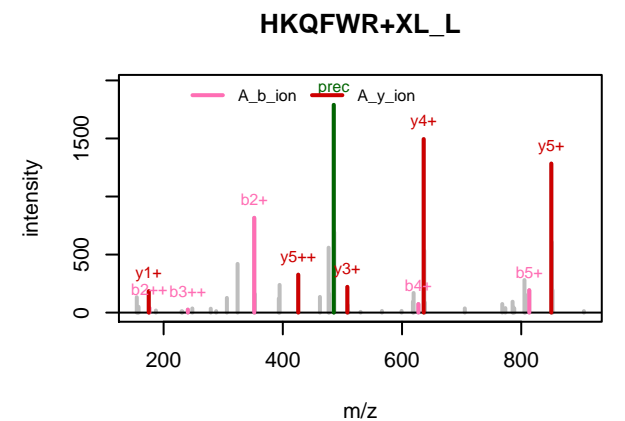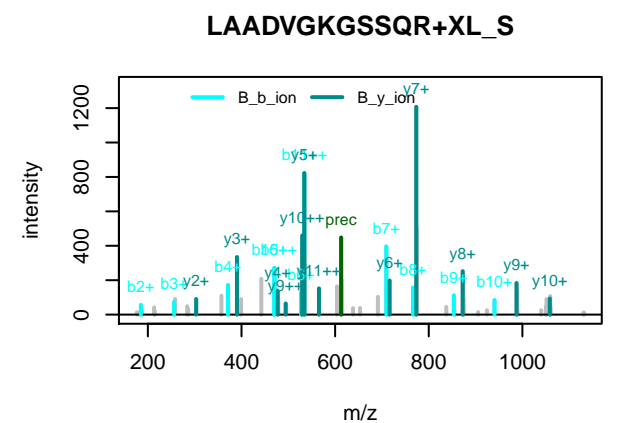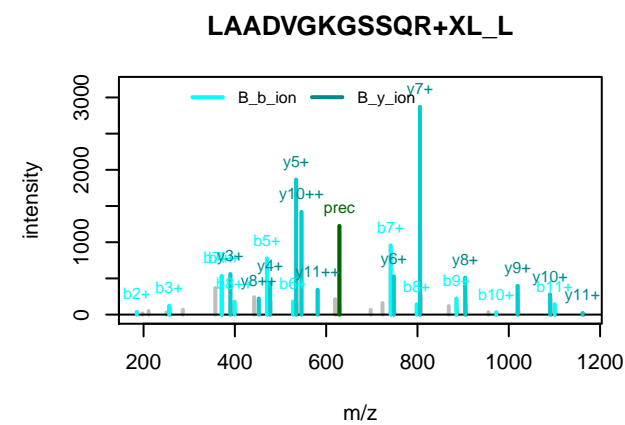

Supplement: Supplemental Data [file supp_RA117.000470_133922_0_supp_23978_fzffwf.zip › spectra_annotation/mito_DR_spectra_annotation/101-1-8-1-7-1.pdf]

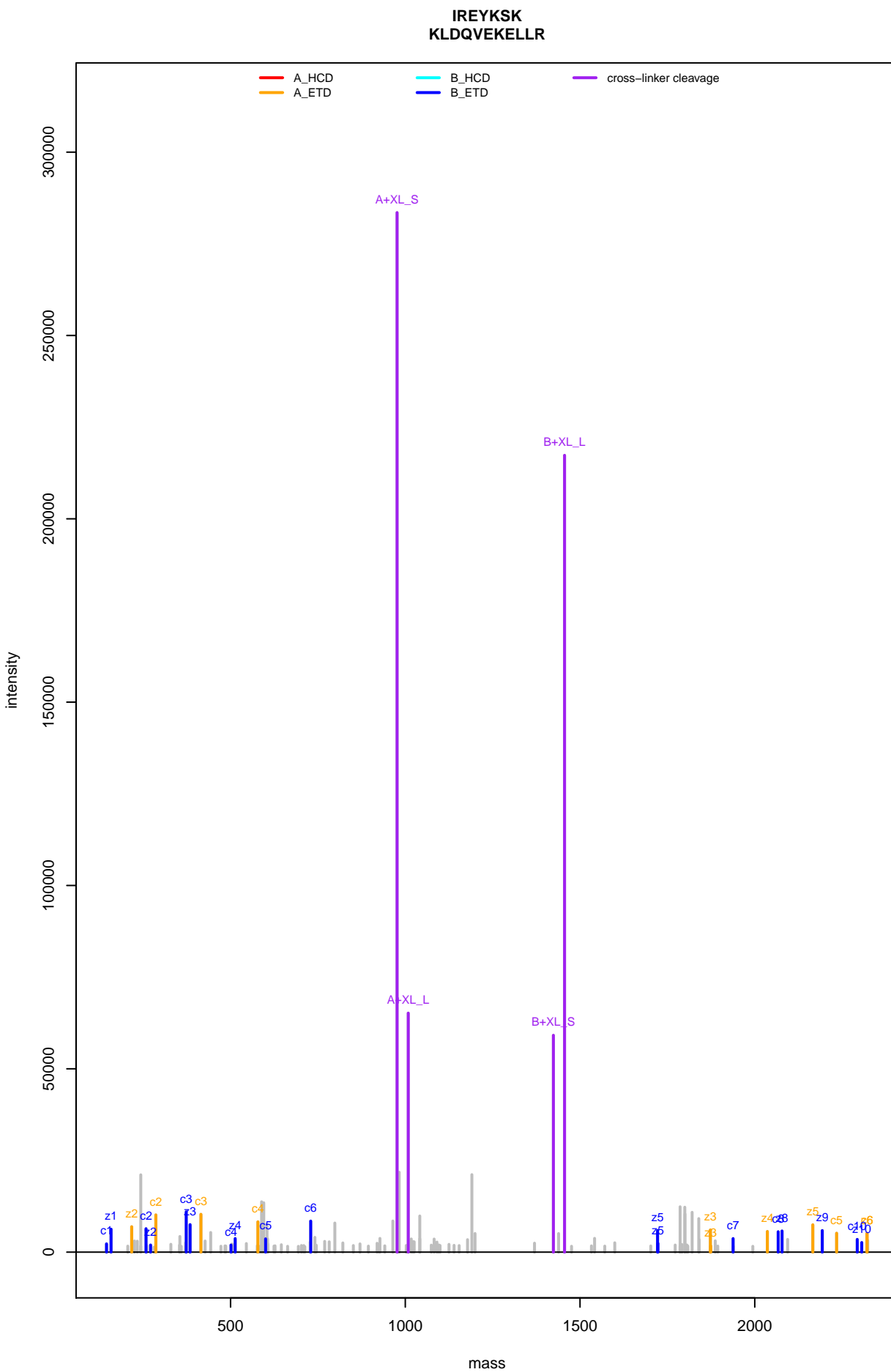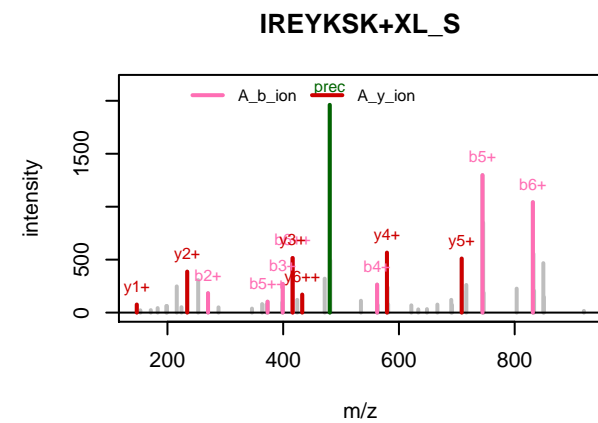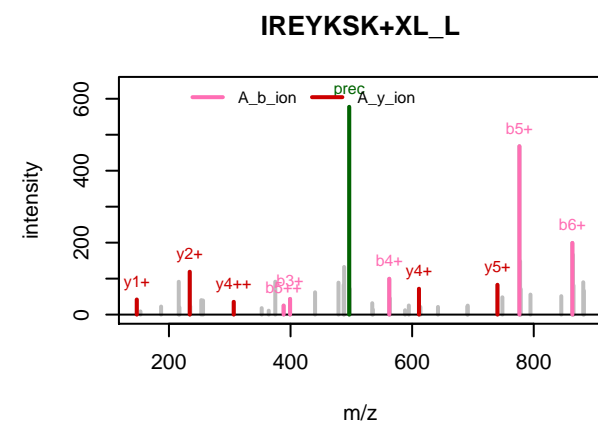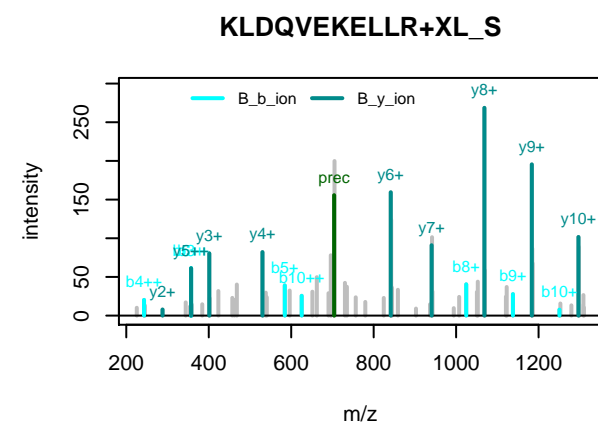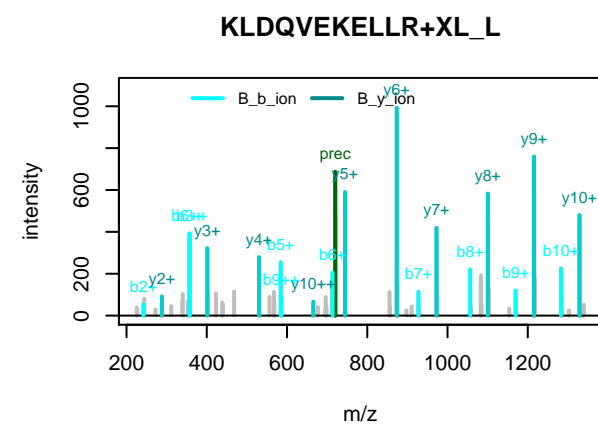

Supplement: Supplemental Data [file supp_RA117.000470_133922_0_supp_23978_fzffwf.zip › spectra_annotation/mito_DR_spectra_annotation/102-1-10-1-14-1.pdf]

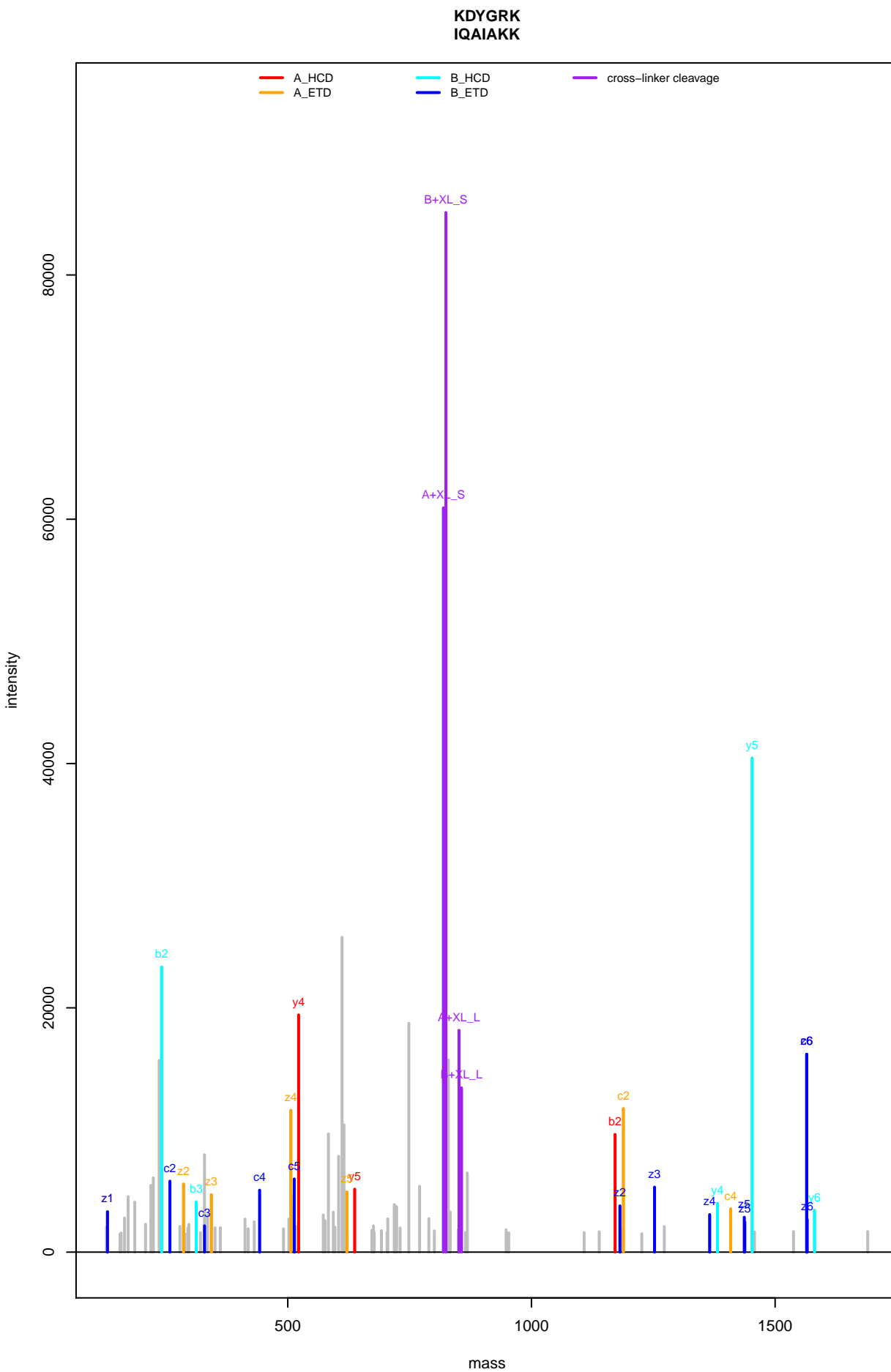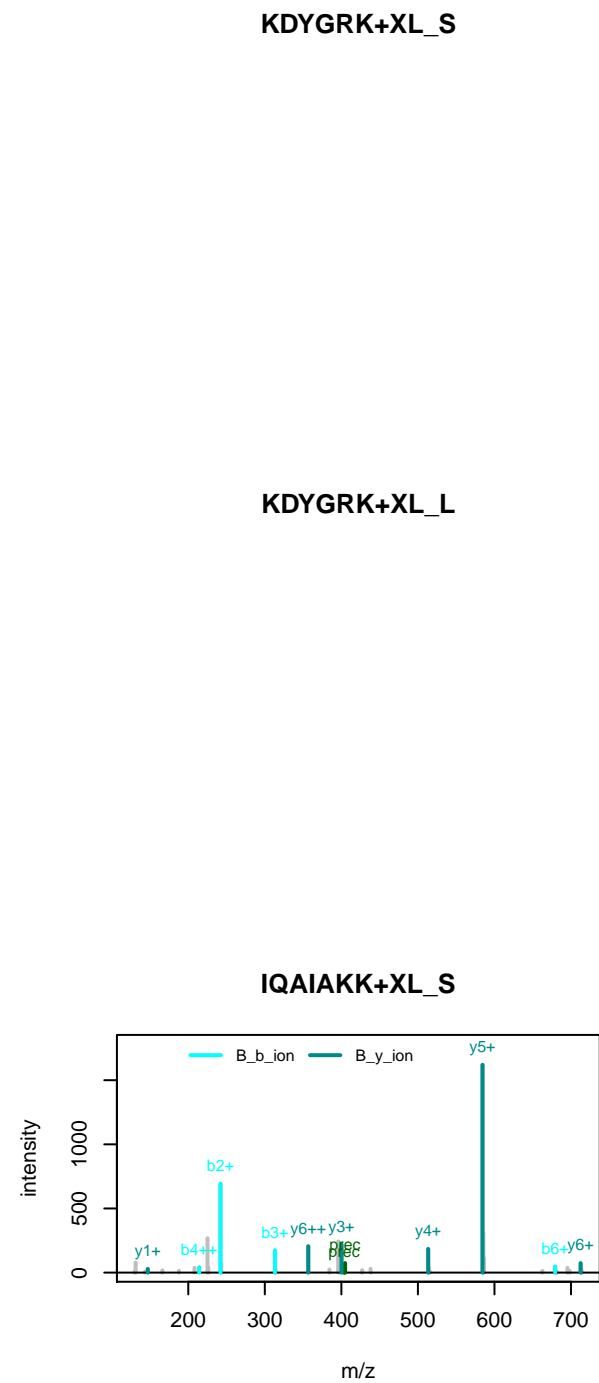

**IQAIKK+XL\_L**

Supplement: Supplemental Data [file supp_RA117.000470_133922_0_supp_23978_fzffwf.zip › spectra_annotation/mito_DR_spectra_annotation/102-1-10-1-23-1.pdf]

IREYKSK  
YTALVDQEEKEDVK

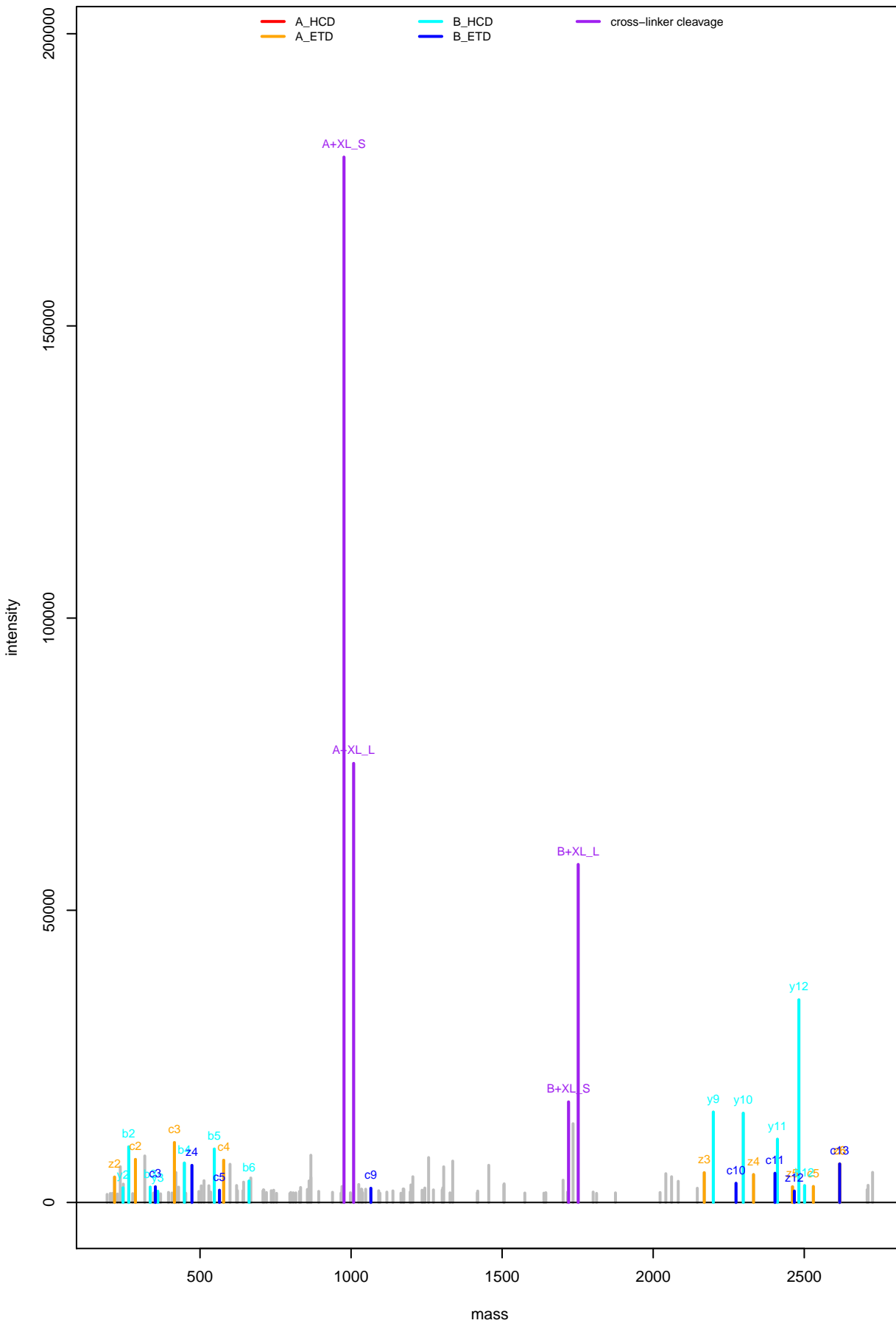

IREYKSK+XL\_S

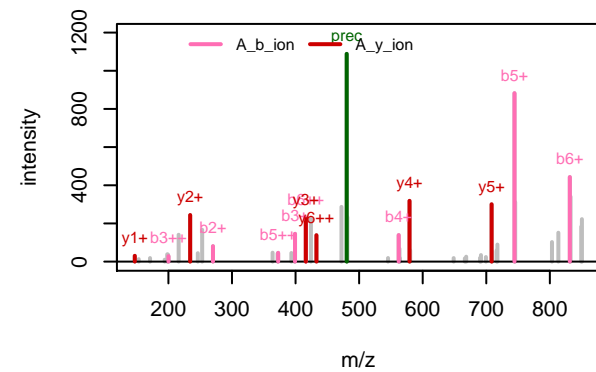

IREYKSK+XL\_L

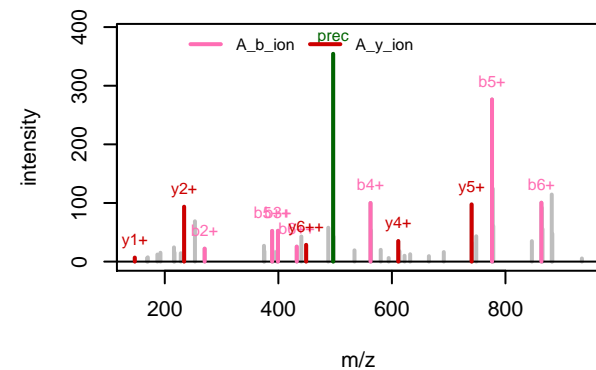

YTALVDQEEKEDVK+XL\_S

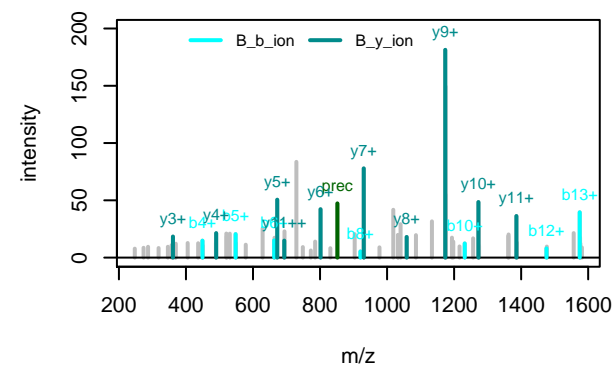

YTALVDQEEKEDVK+XL\_L

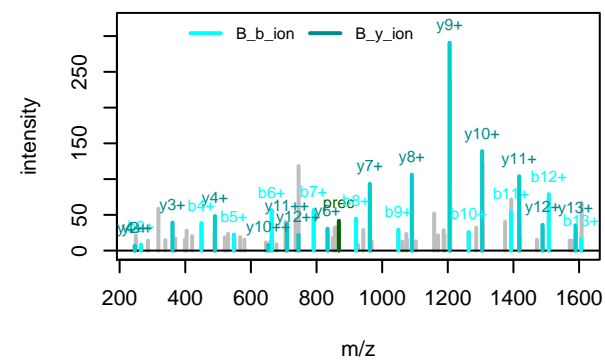

Supplement: Supplemental Data [file supp_RA117.000470_133922_0_supp_23978_fzffwf.zip › spectra_annotation/mito_DR_spectra_annotation/102-1-10-1-9-1.pdf]

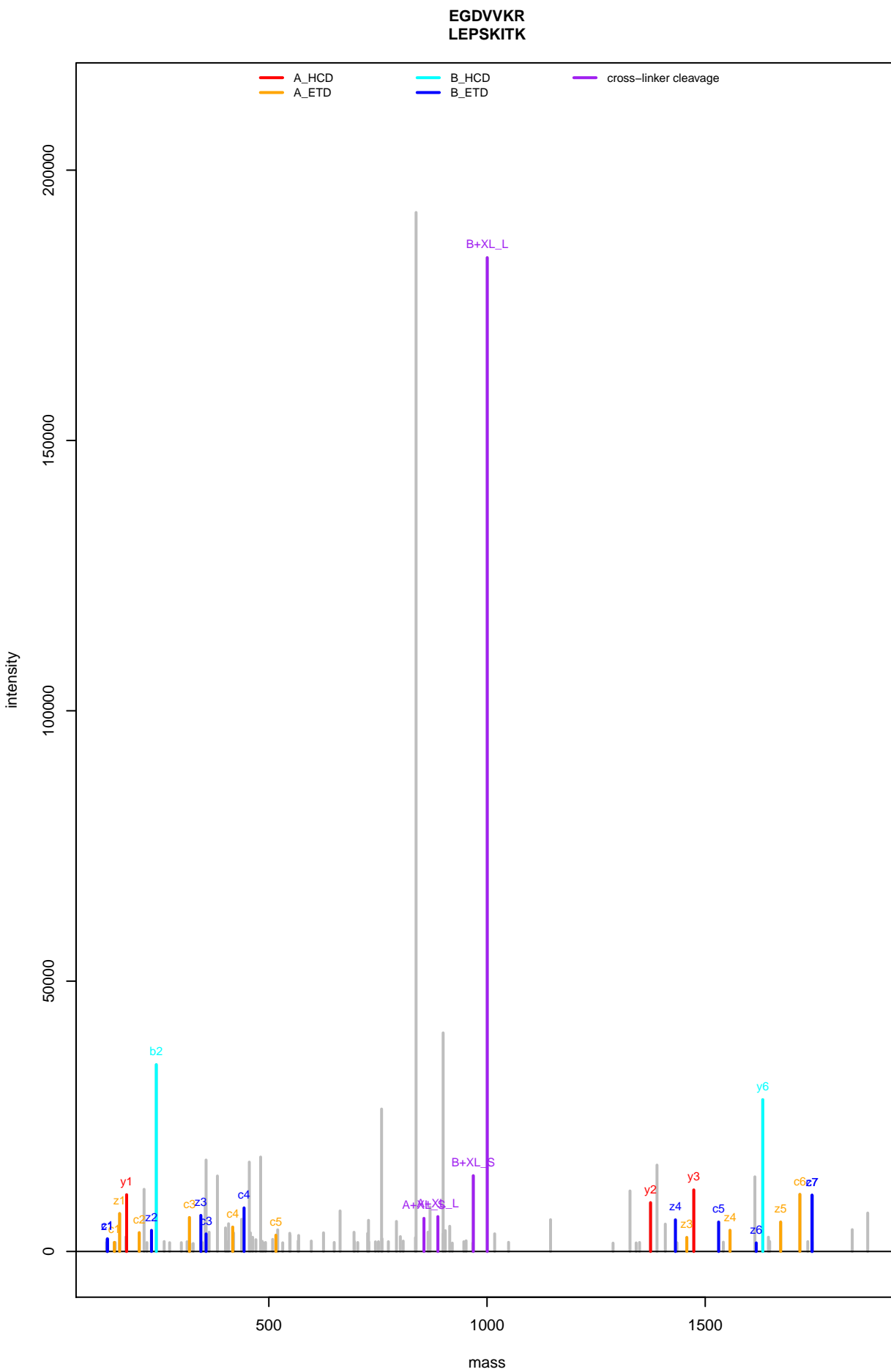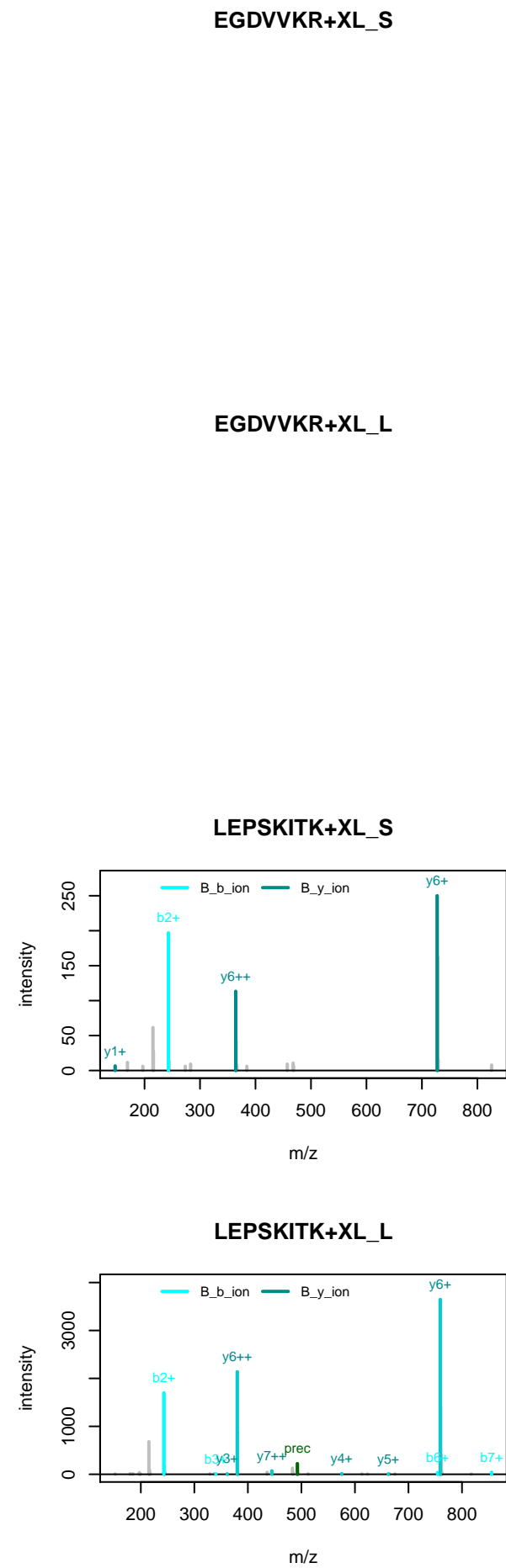

Supplement: Supplemental Data [file supp_RA117.000470_133922_0_supp_23978_fzffwf.zip › spectra_annotation/mito_DR_spectra_annotation/103-1-14-1-19-1.pdf]

ITKSMoK  
SIKNIQK

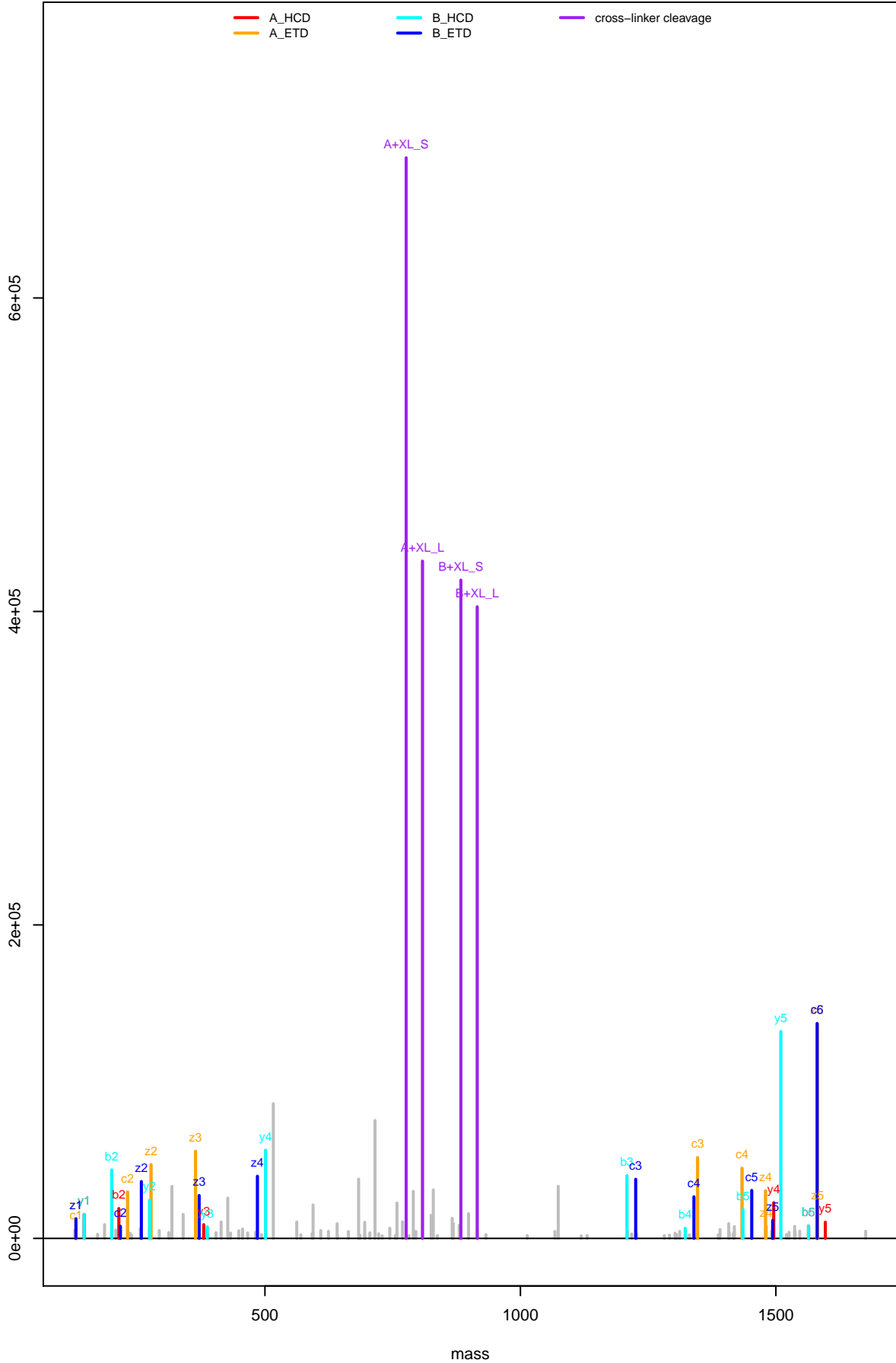

## ITKSMoK+XL\_S

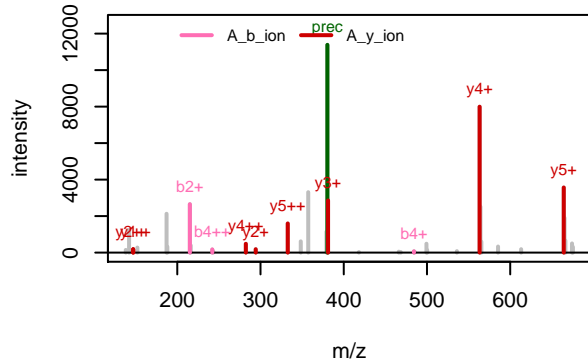

## ITKSMoK+XL\_L

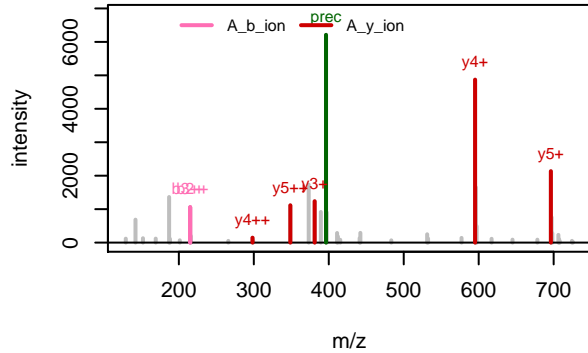

**SIKNIQK+XL\_S**

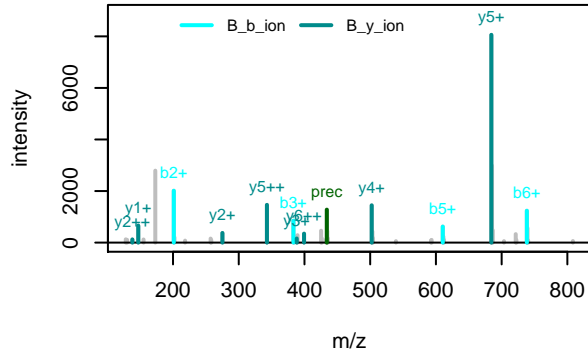

## SIKNIQK+XL L

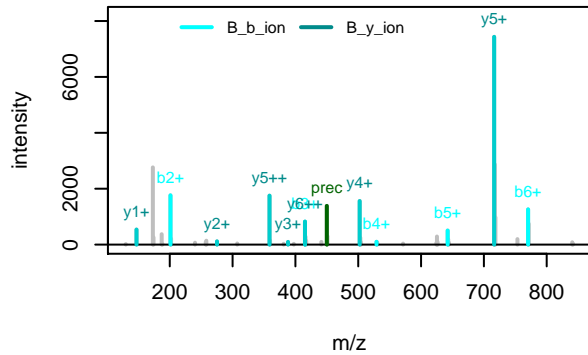

Supplement: Supplemental Data [file supp_RA117.000470_133922_0_supp_23978_fzffwf.zip › spectra_annotation/mito_DR_spectra_annotation/104-1-1-1-2-1.pdf]

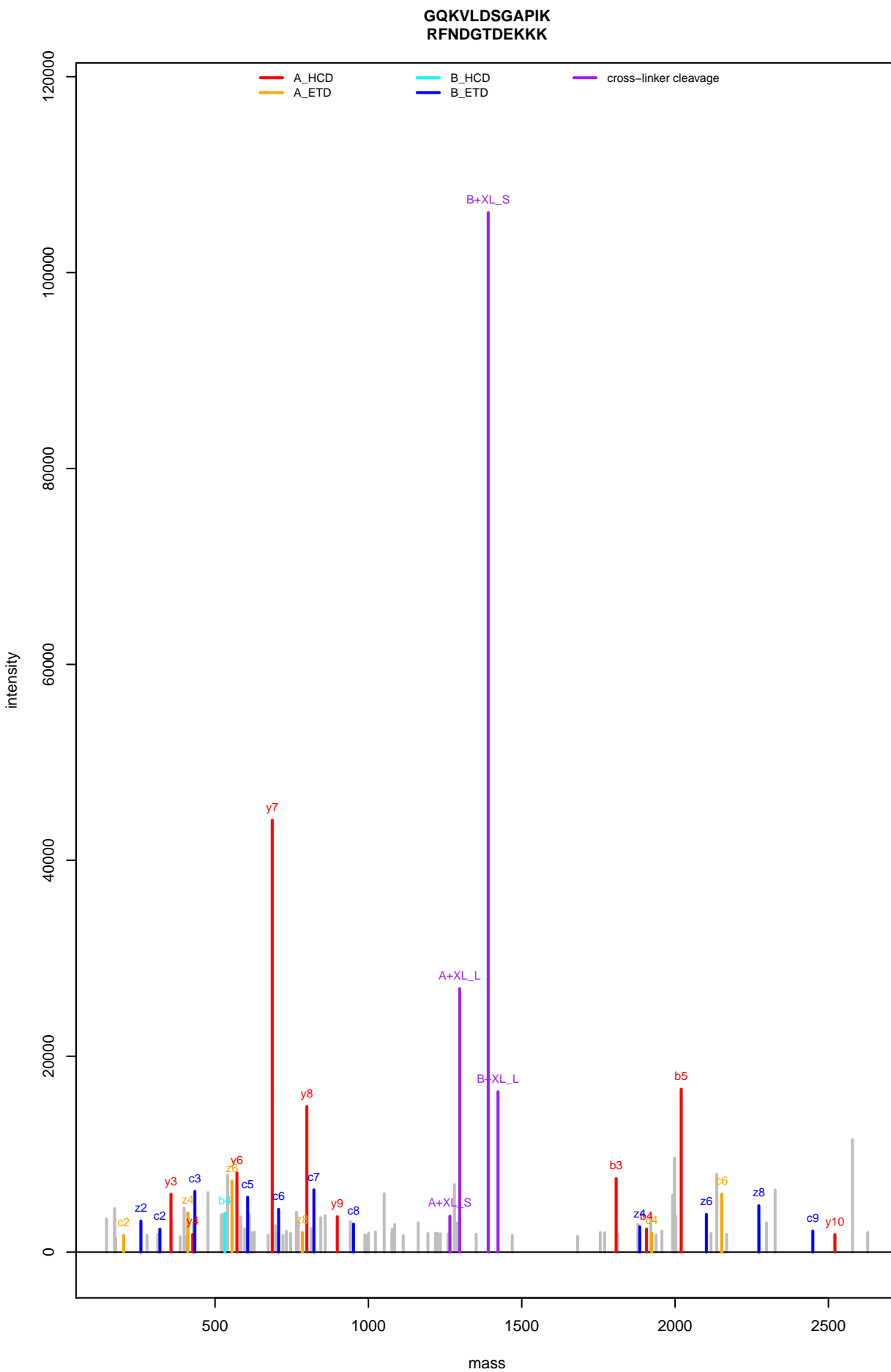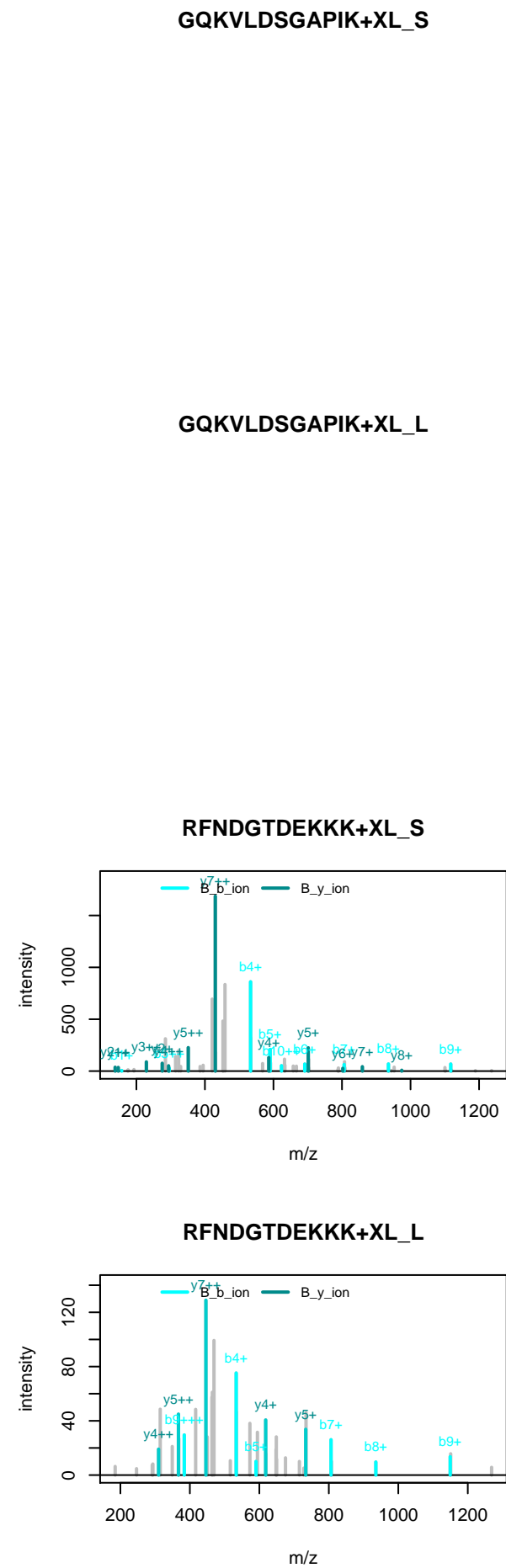

Supplement: Supplemental Data [file supp_RA117.000470_133922_0_supp_23978_fzffwf.zip › spectra_annotation/mito_DR_spectra_annotation/104-1-2-1-15-1.pdf]

**LKKEGPDF  
YKQIFLGGVDRHK**

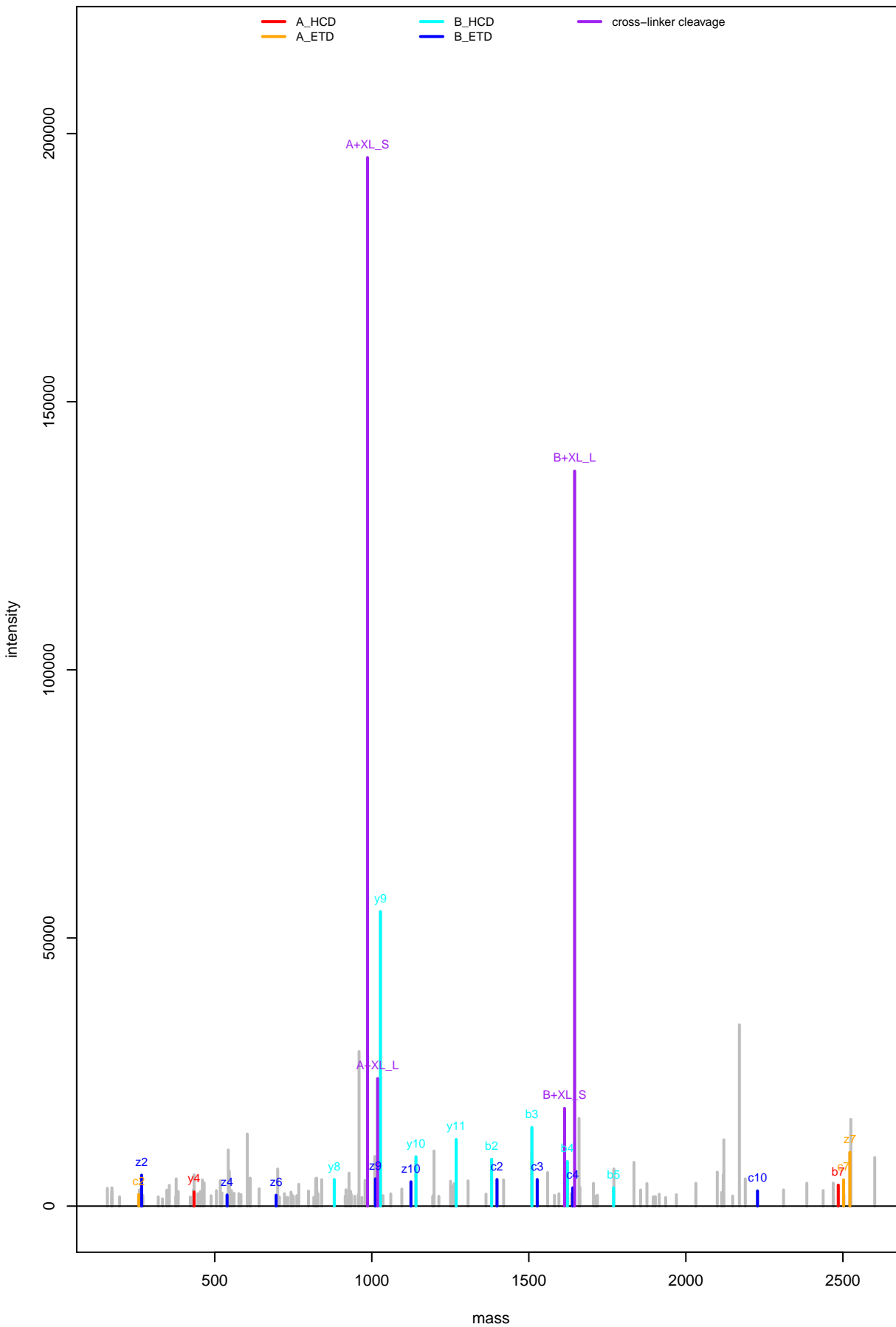

**LKKEGPDF+XL\_S**

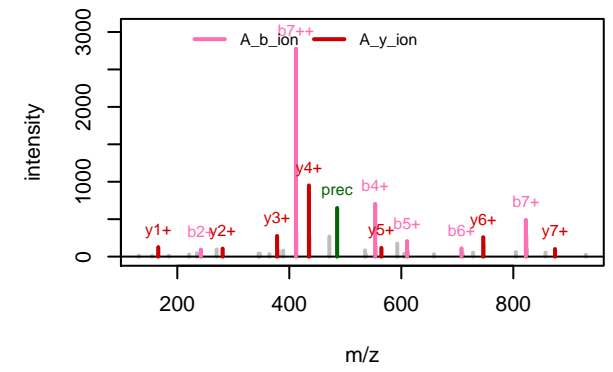

**LKKEGPDF+XL\_L**

**YKQIFLGGVDRHK+XL\_S**

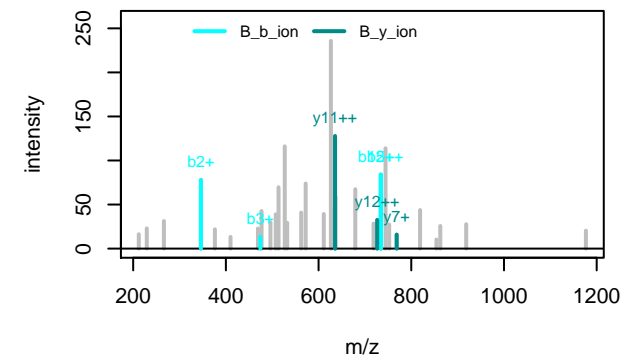

**YKQIFLGGVDRHK+XL\_L**

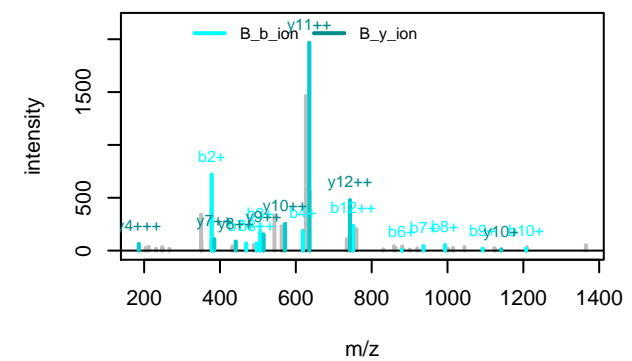

Supplement: Supplemental Data [file supp_RA117.000470_133922_0_supp_23978_fzffwf.zip › spectra_annotation/mito_DR_spectra_annotation/104-1-6-1-11-1.pdf]

# KAYAEFYR DHCVAHKLFK

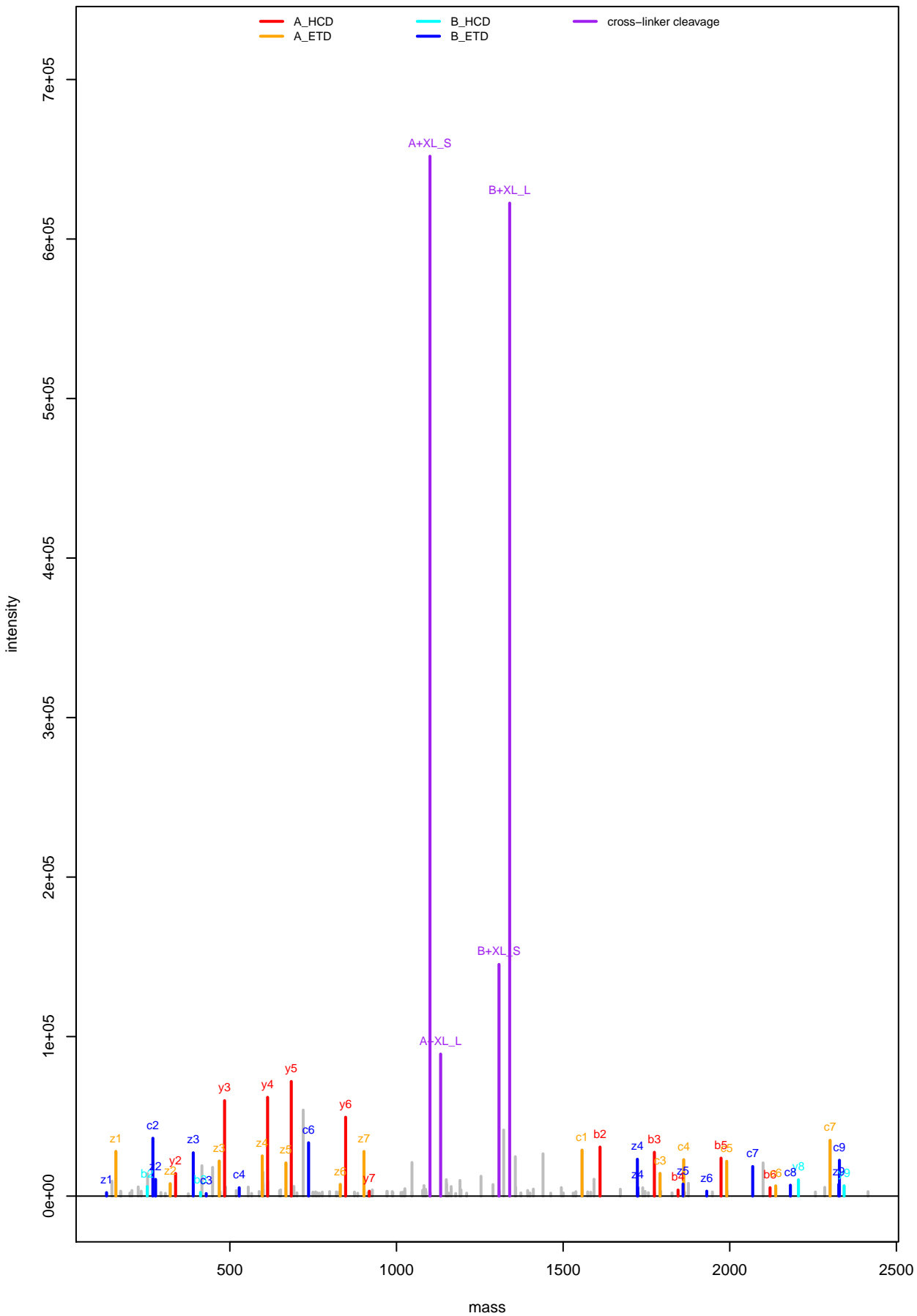

## KAYAEFYR+XL\_S

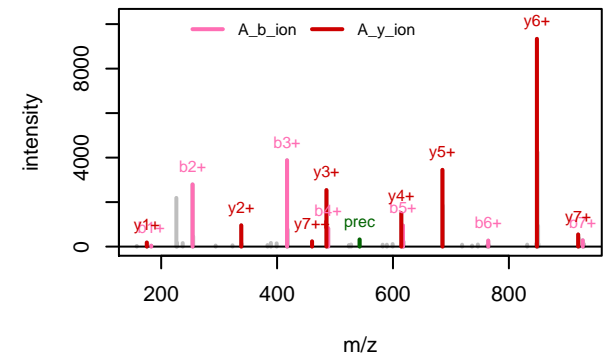

## KAYAEFYR+XL\_L

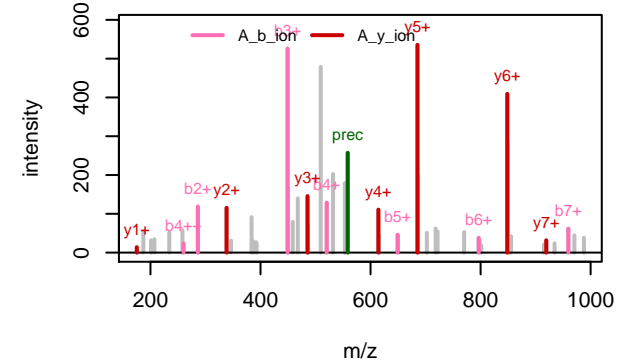

## DHCVAHKLFK+XL\_S

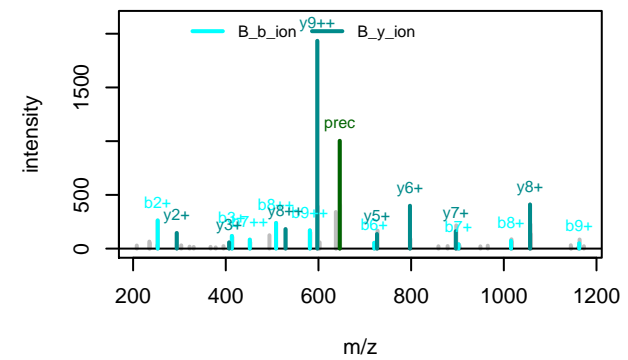

## DHCVAHKLFK+XL\_L

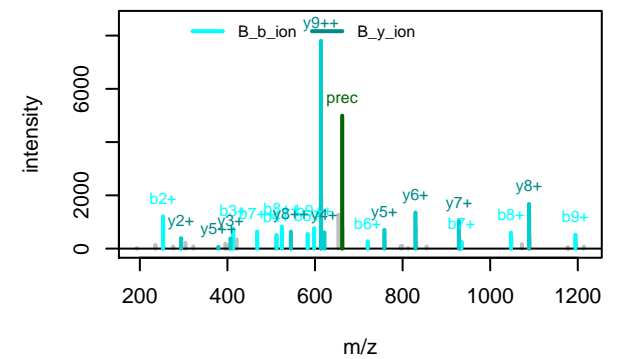

Supplement: Supplemental Data [file supp_RA117.000470_133922_0_supp_23978_fzffwf.zip › spectra_annotation/mito_DR_spectra_annotation/105-1-10-1-4-1.pdf]

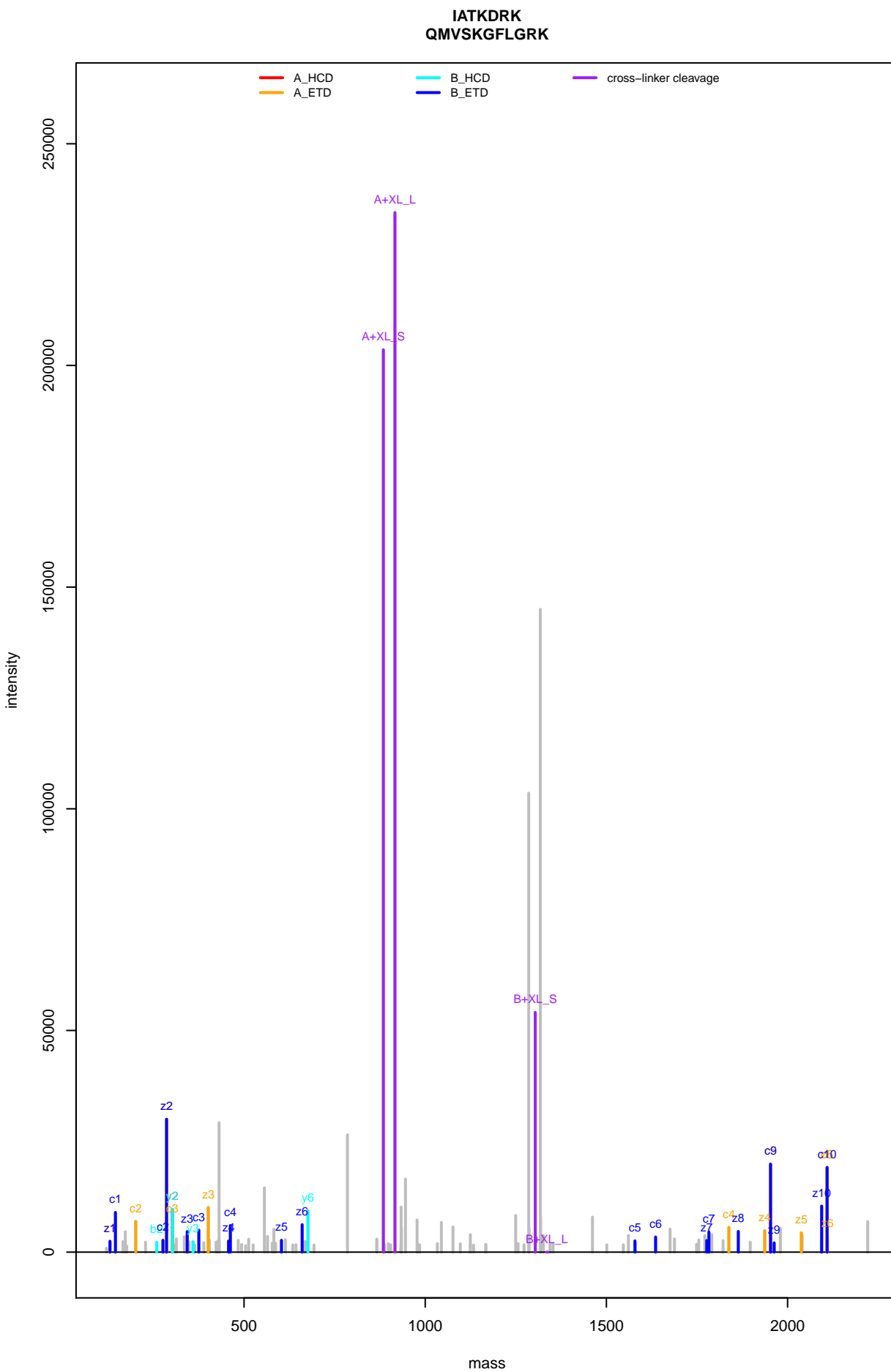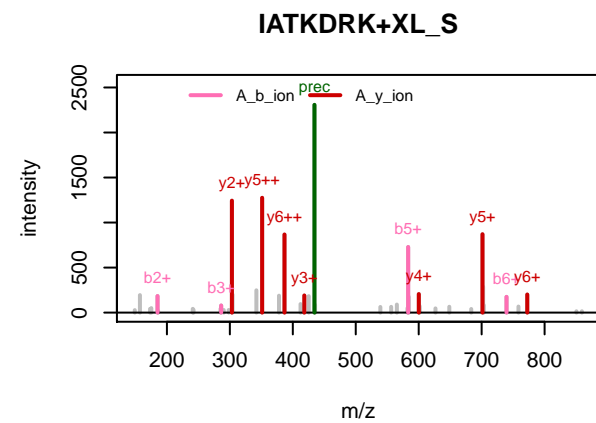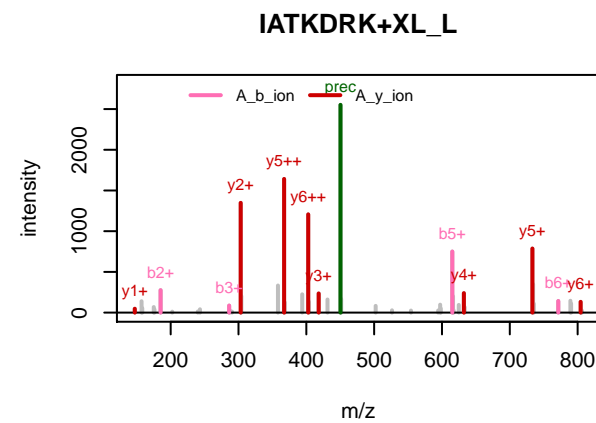

**QMVSKGFLGRK+XL\_S**

**QMVSKGFLGRK+XL\_L**

Supplement: Supplemental Data [file supp_RA117.000470_133922_0_supp_23978_fzffwf.zip › spectra_annotation/mito_DR_spectra_annotation/105-1-4-1-6-1.pdf]

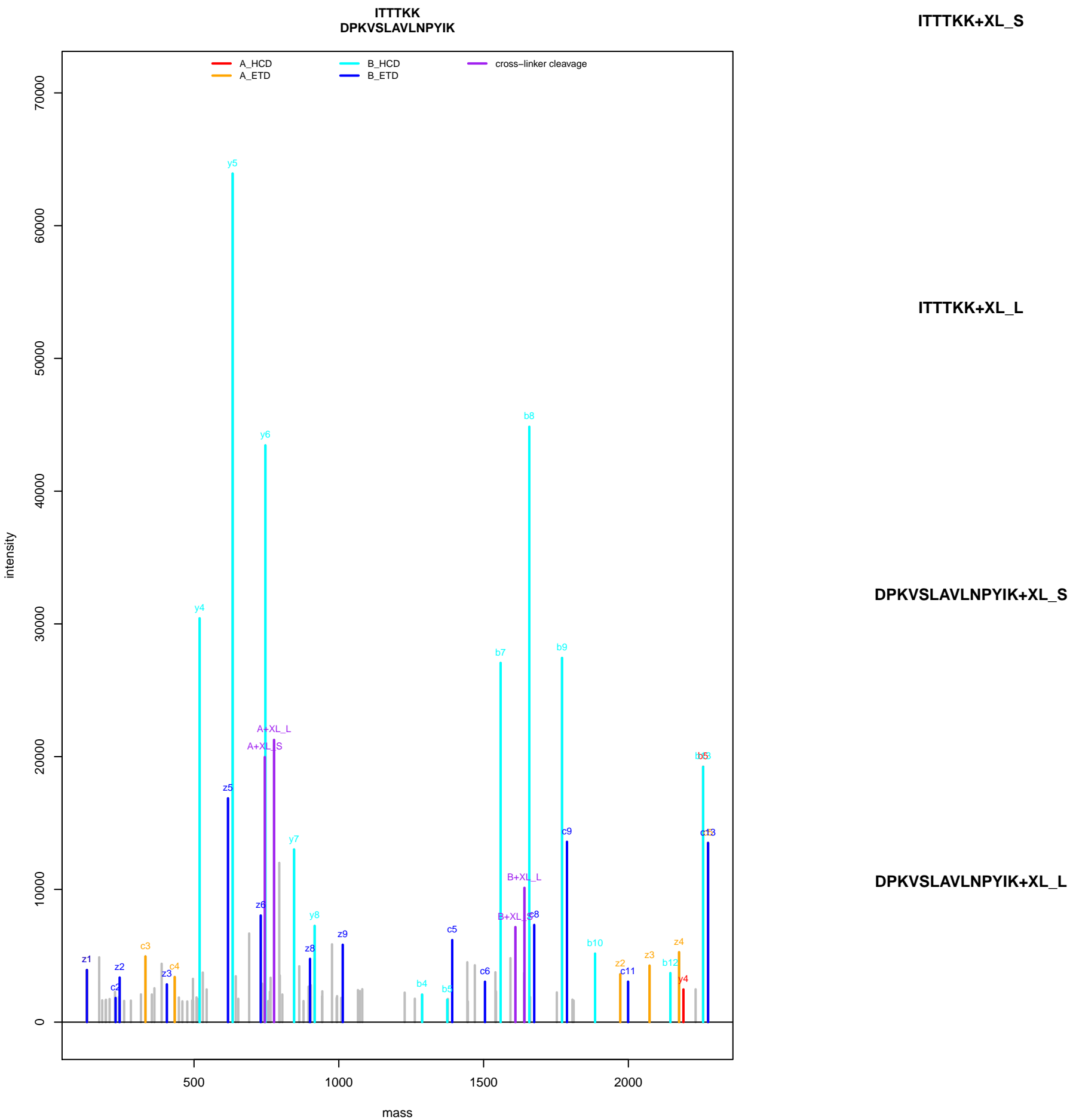

Supplement: Supplemental Data [file supp_RA117.000470_133922_0_supp_23978_fzffwf.zip › spectra_annotation/mito_DR_spectra_annotation/106-1-1-1-15-1.pdf]

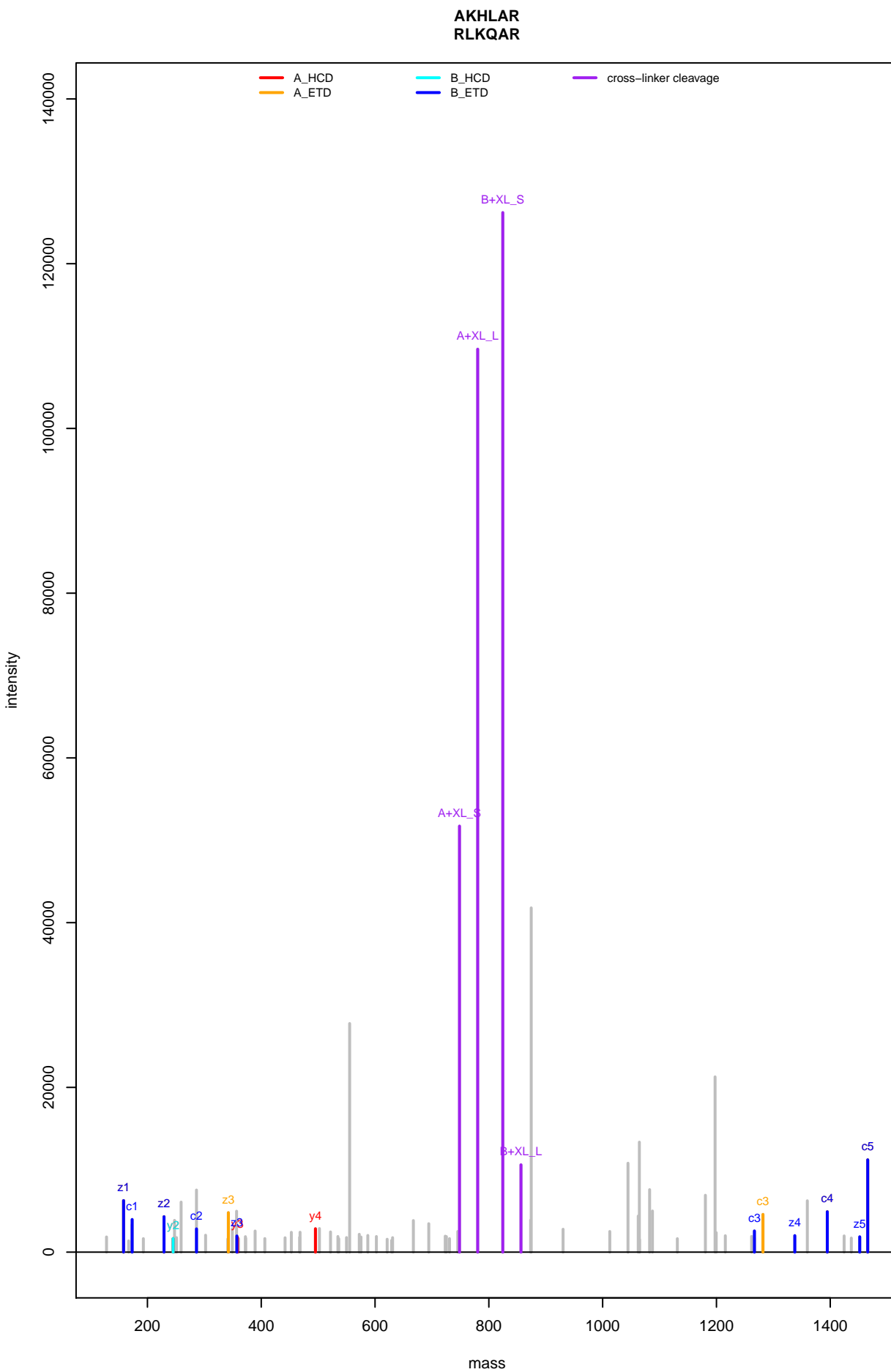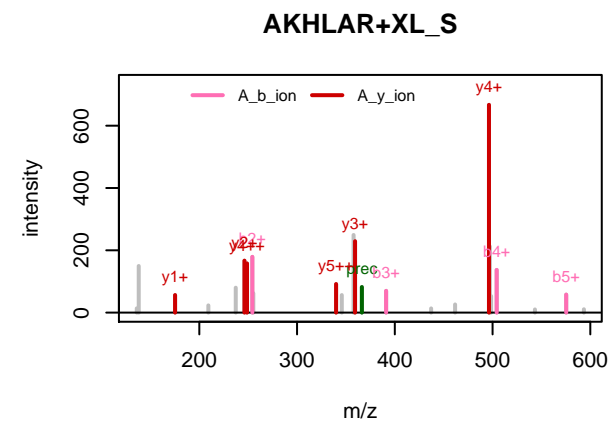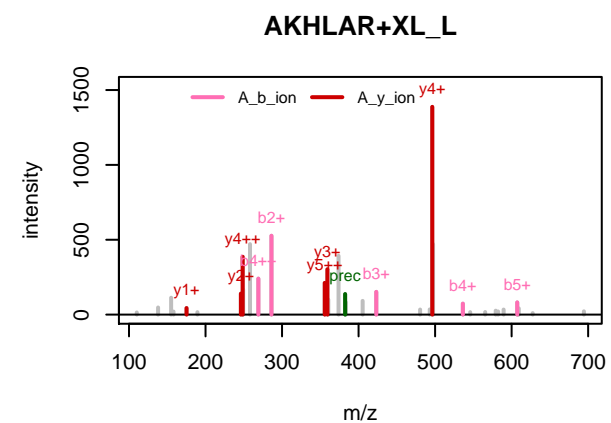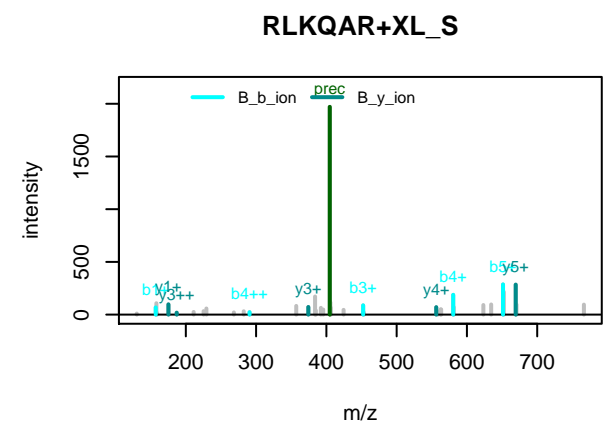

**RLKQAR+XL\_L**

Supplement: Supplemental Data [file supp_RA117.000470_133922_0_supp_23978_fzffwf.zip › spectra_annotation/mito_DR_spectra_annotation/107-1-1-1-10-1.pdf]

**QILGQAKK  
LAADVKGSSQR**

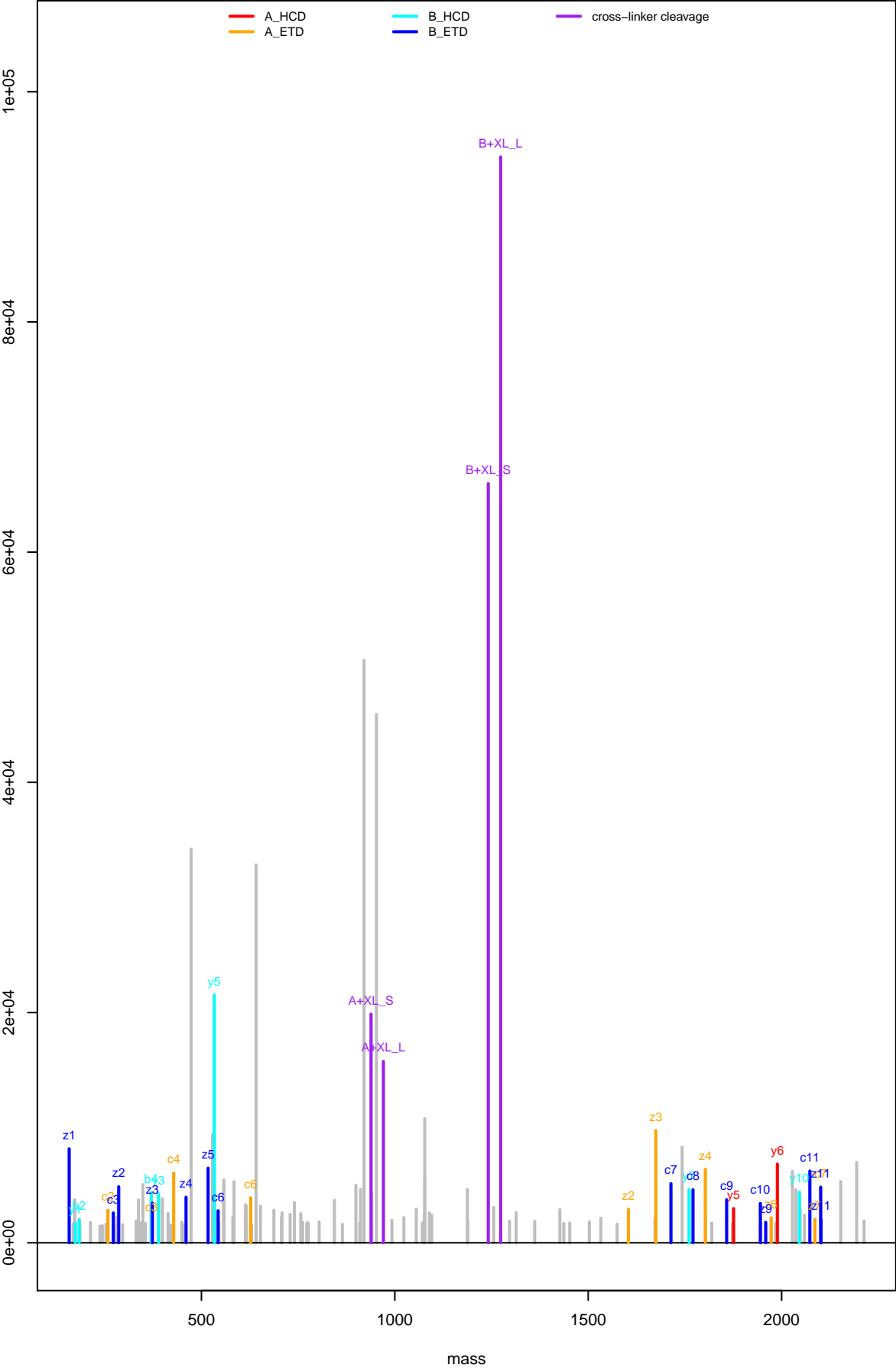

**QILGQAKK+XL\_S**

**QILGQAKK+XL\_L**

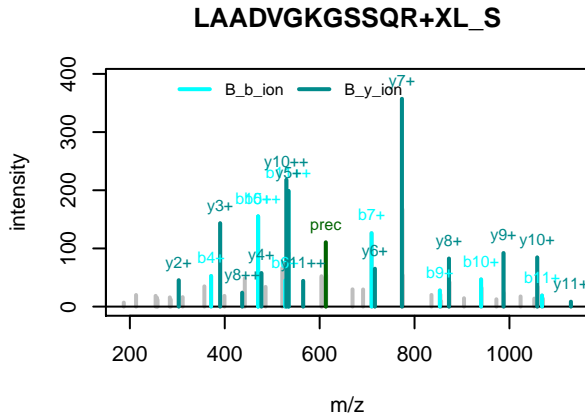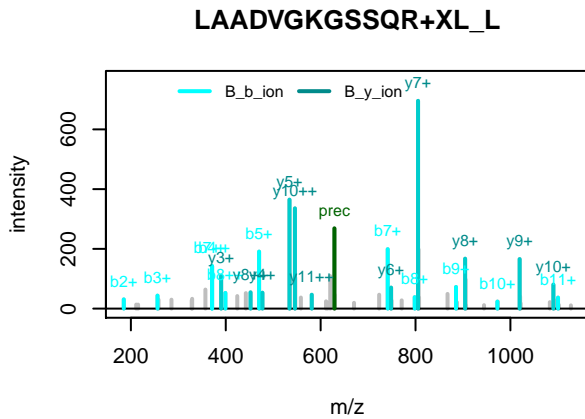

Supplement: Supplemental Data [file supp_RA117.000470_133922_0_supp_23978_fzffwf.zip › spectra_annotation/mito_DR_spectra_annotation/107-1-5-1-9-1.pdf]

KYNALK  
ANVAKPGLVDDFEK

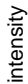

**KYNALK+XL\_S**

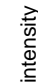

**KYNALK+XL\_L**

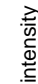

**ANVAKPGLVDDFEK+XL\_S**

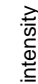

**ANVAKPGLVDDFEK+XL\_L**

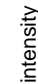

Supplement: Supplemental Data [file supp_RA117.000470_133922_0_supp_23978_fzffwf.zip › spectra_annotation/mito_DR_spectra_annotation/107-1-6-1-10-1.pdf]

QMVSKGFLGRK  
GFYIQEGSKNK

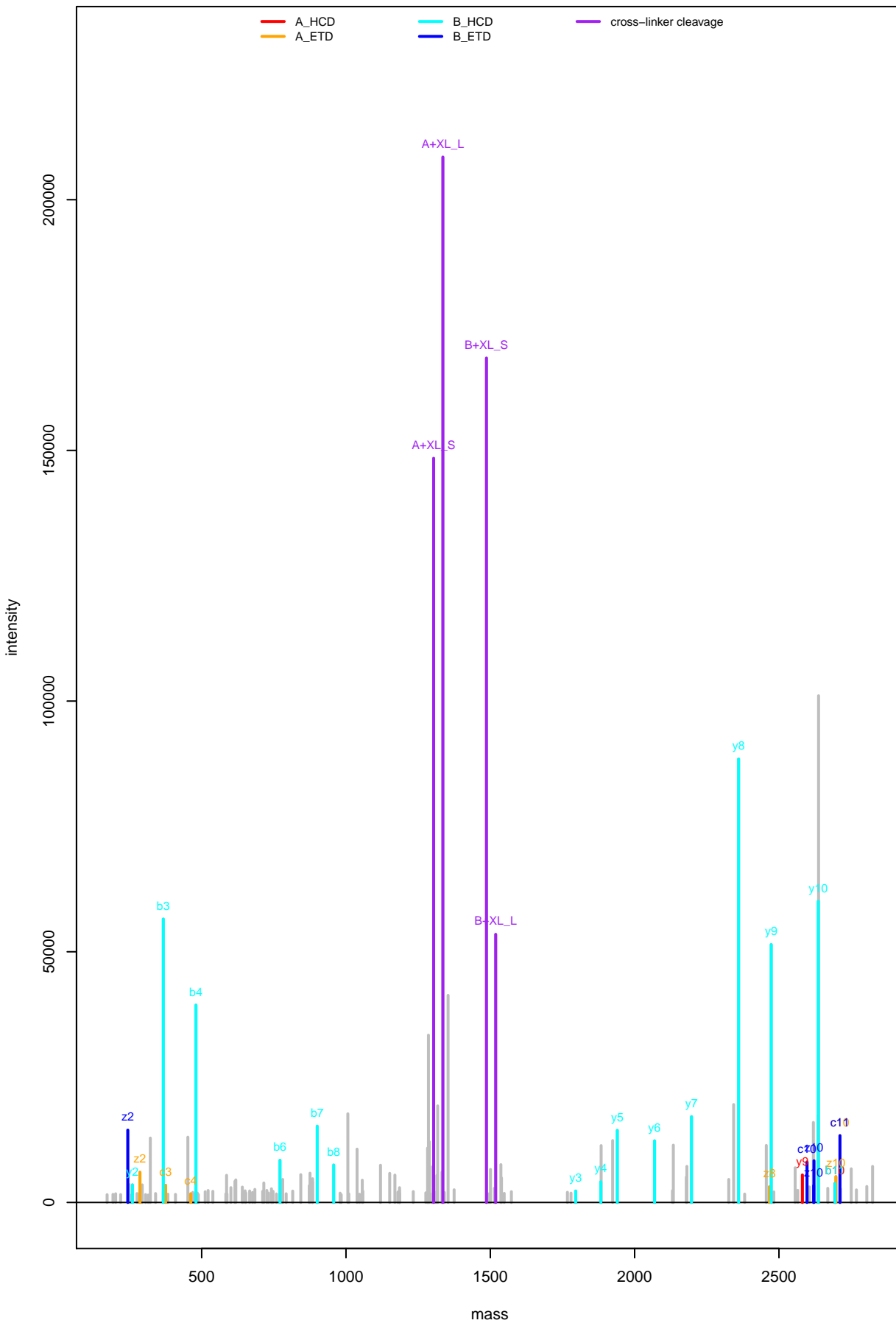

QMVSKGFLGRK+XL\_S

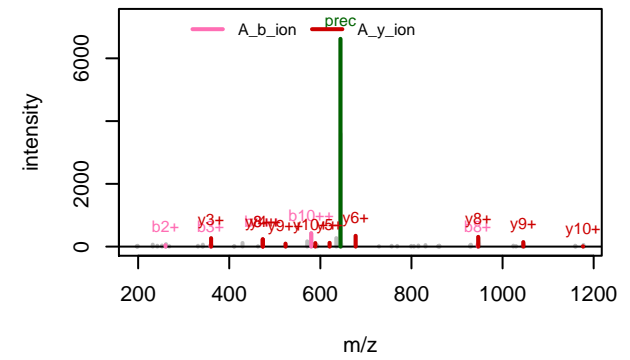

QMVSKGFLGRK+XL\_L

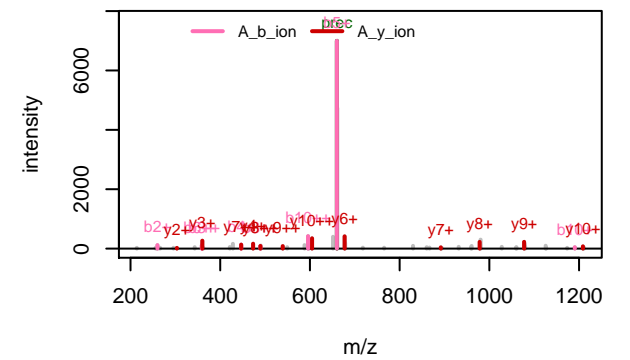

GFYIQEGSKNK+XL\_S

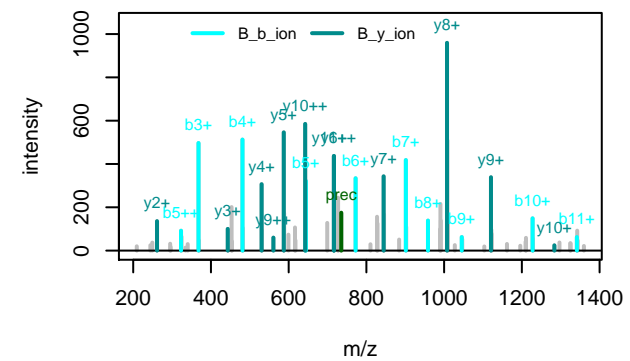

GFYIQEGSKNK+XL\_L

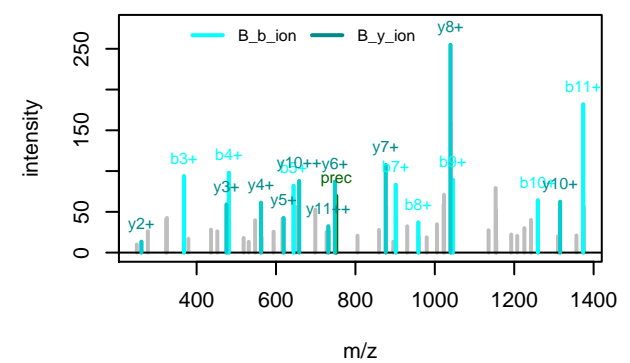

Supplement: Supplemental Data [file supp_RA117.000470_133922_0_supp_23978_fzffwf.zip › spectra_annotation/mito_DR_spectra_annotation/107-1-6-1-4-1.pdf]

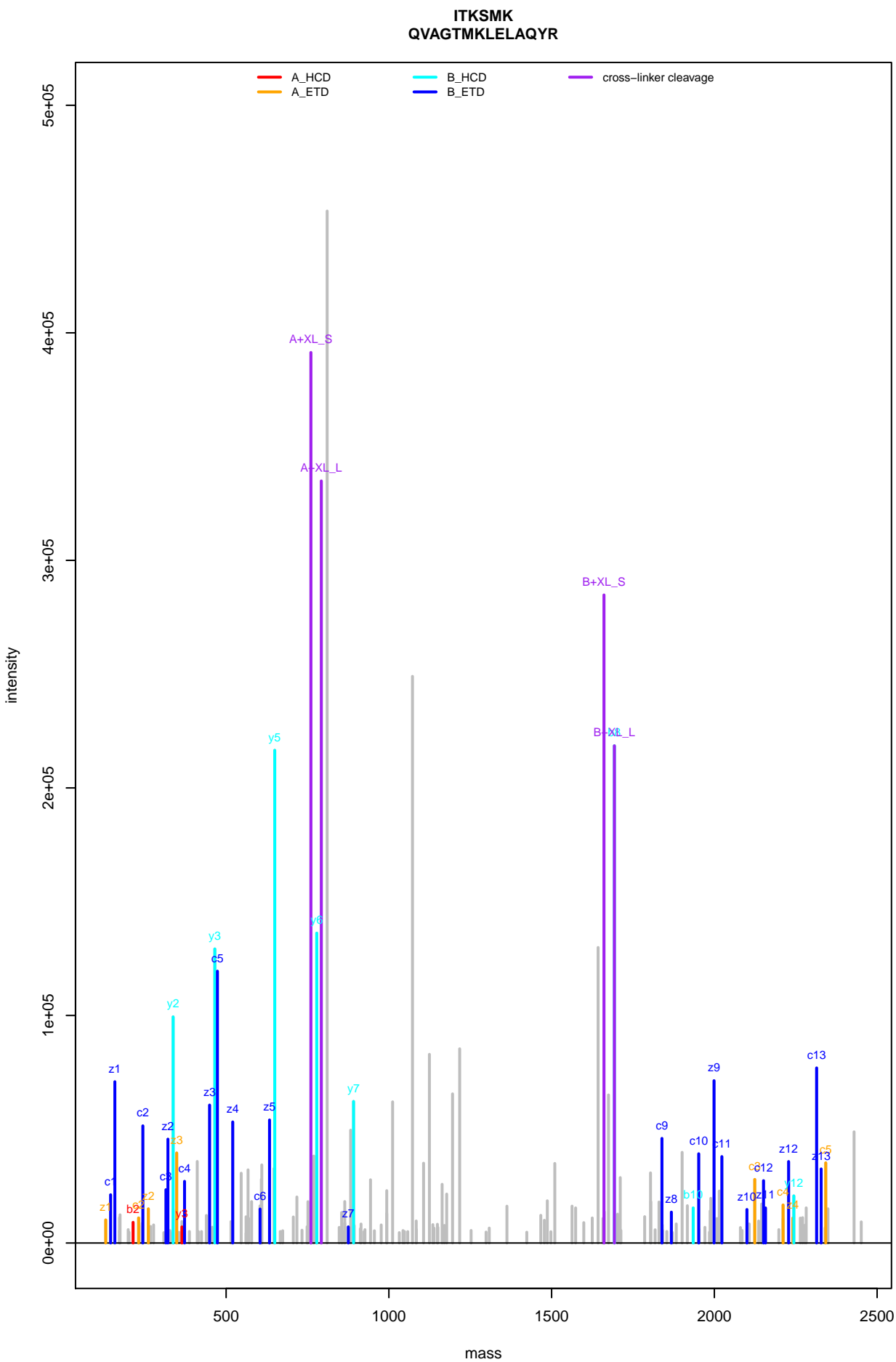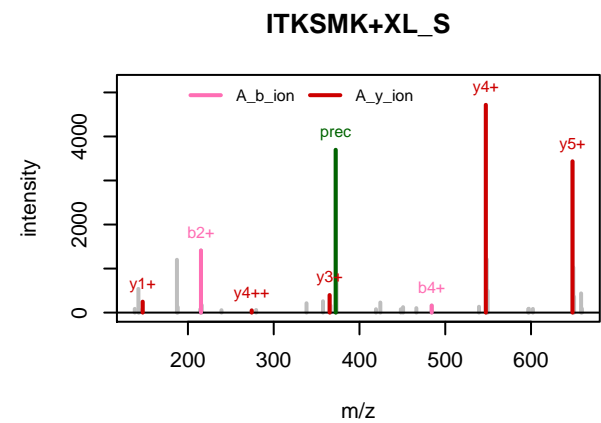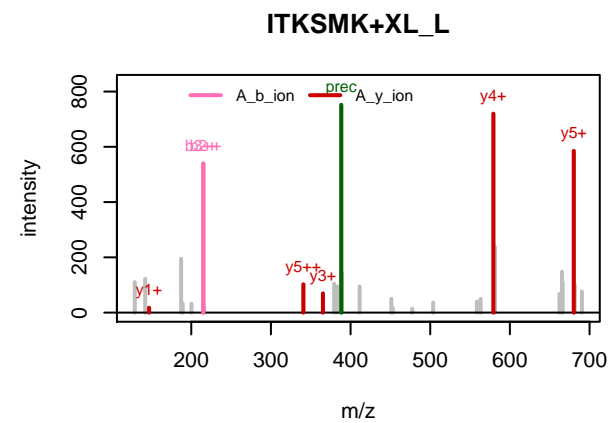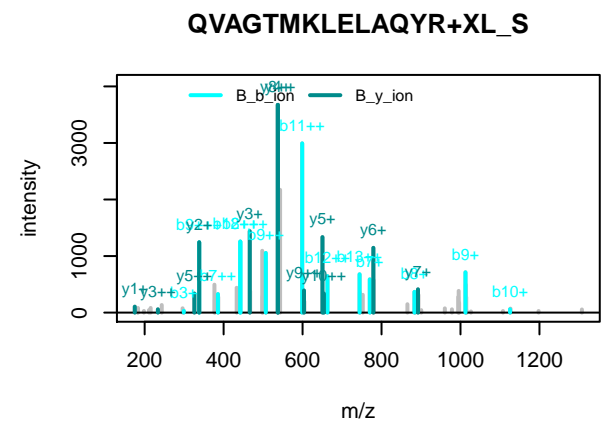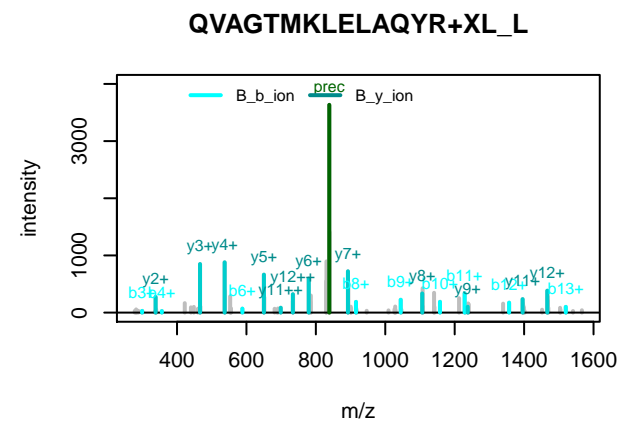

Supplement: Supplemental Data [file supp_RA117.000470_133922_0_supp_23978_fzffwf.zip › spectra_annotation/mito_DR_spectra_annotation/108-1-1-1-20-1.pdf]

**SVISYKTEEK  
EVMIVGVGEKIK**

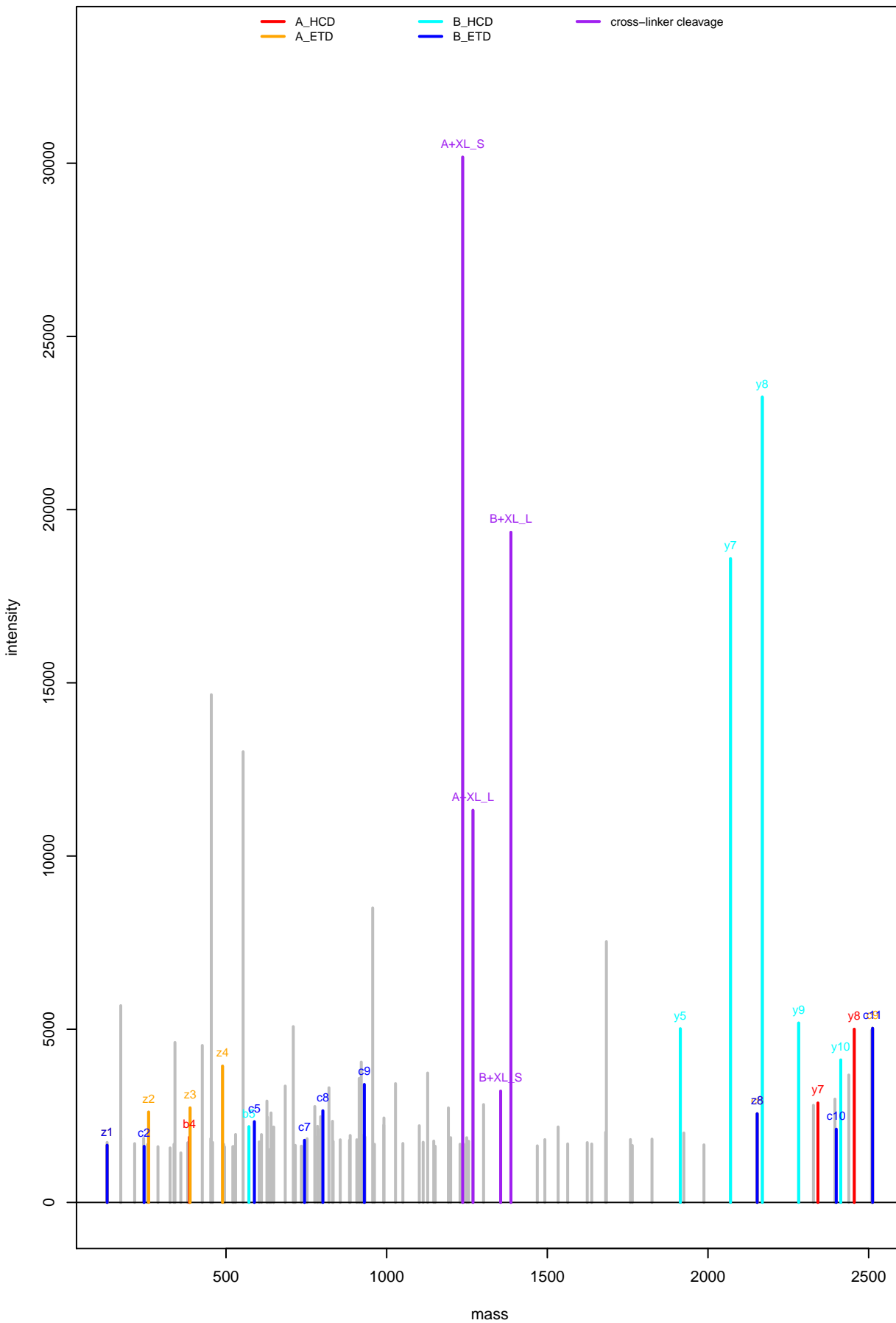

## SVISYKTEEK+XL\_S

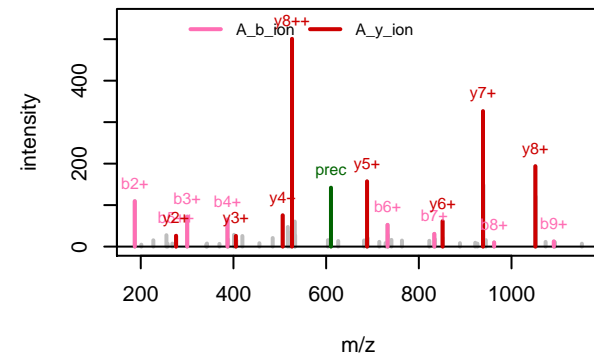

## SVISYKTEEK+XL\_L

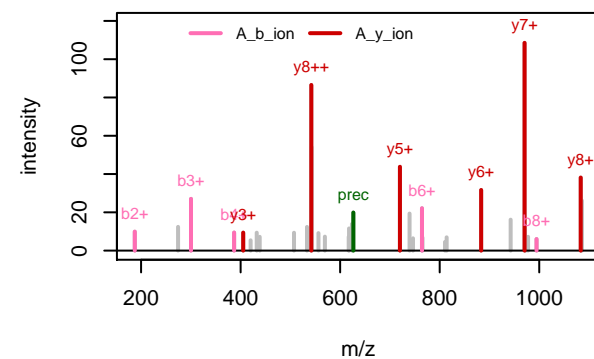

**EVMIVGVGEKIK+XL\_S**

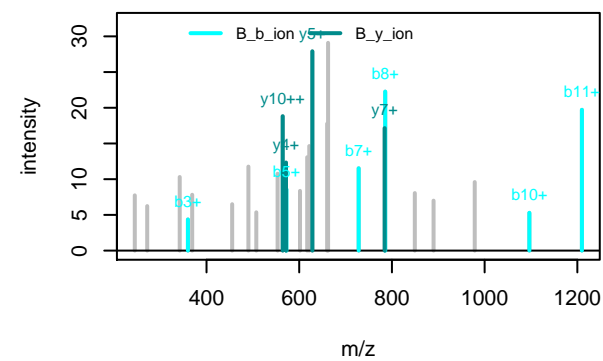

**EVMIVGVGEKIK+XL\_L**

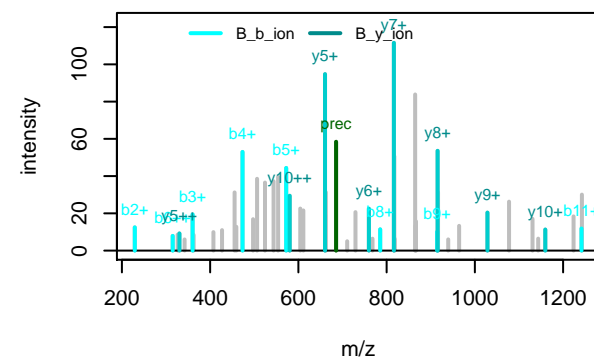

Supplement: Supplemental Data [file supp_RA117.000470_133922_0_supp_23978_fzffwf.zip › spectra_annotation/mito_DR_spectra_annotation/108-1-2-1-6-1.pdf]

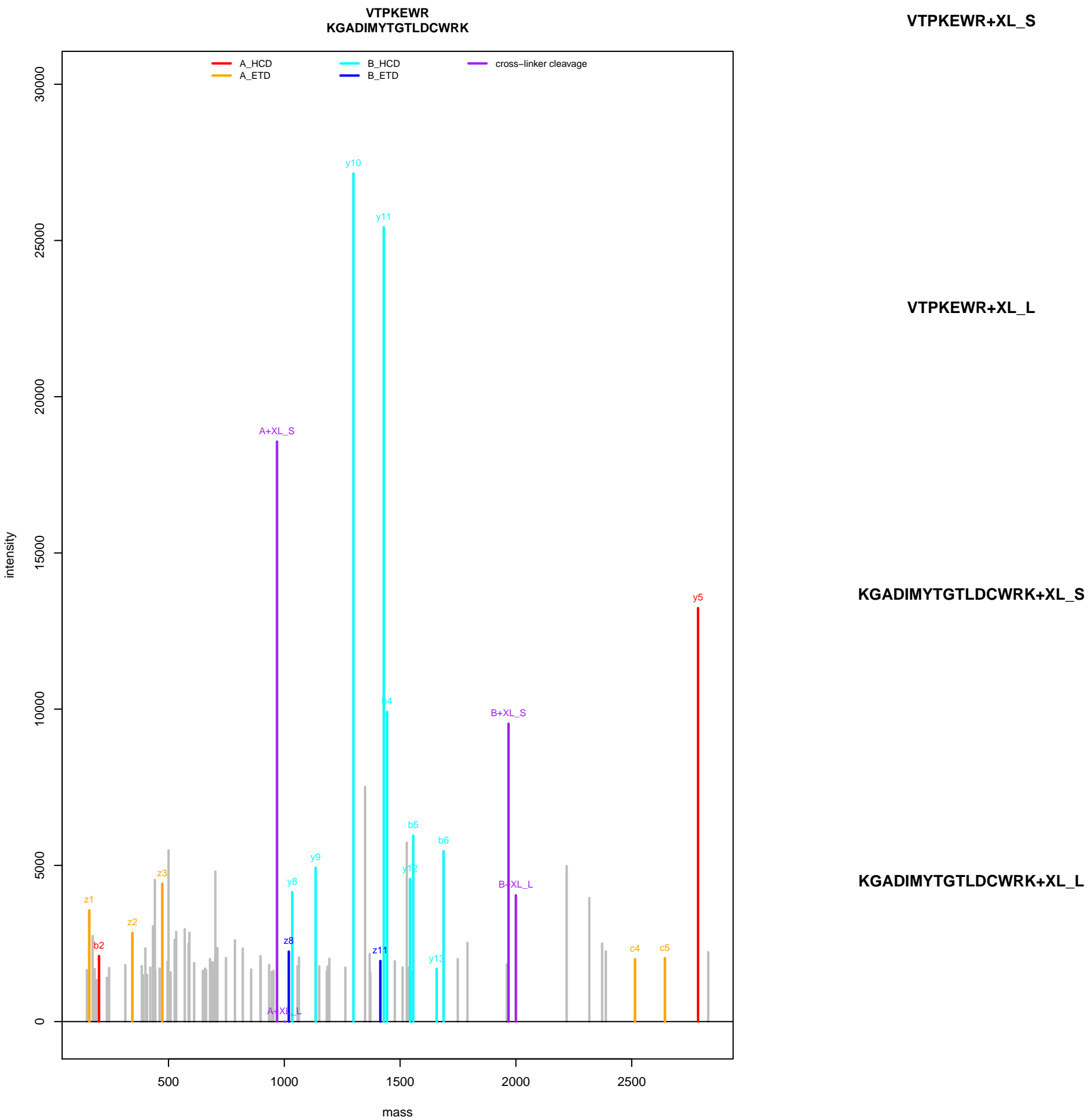

Supplement: Supplemental Data [file supp_RA117.000470_133922_0_supp_23978_fzffwf.zip › spectra_annotation/mito_DR_spectra_annotation/108-1-3-1-5-1.pdf]

# ALKEAR NNPEPWNLGPNEQYK

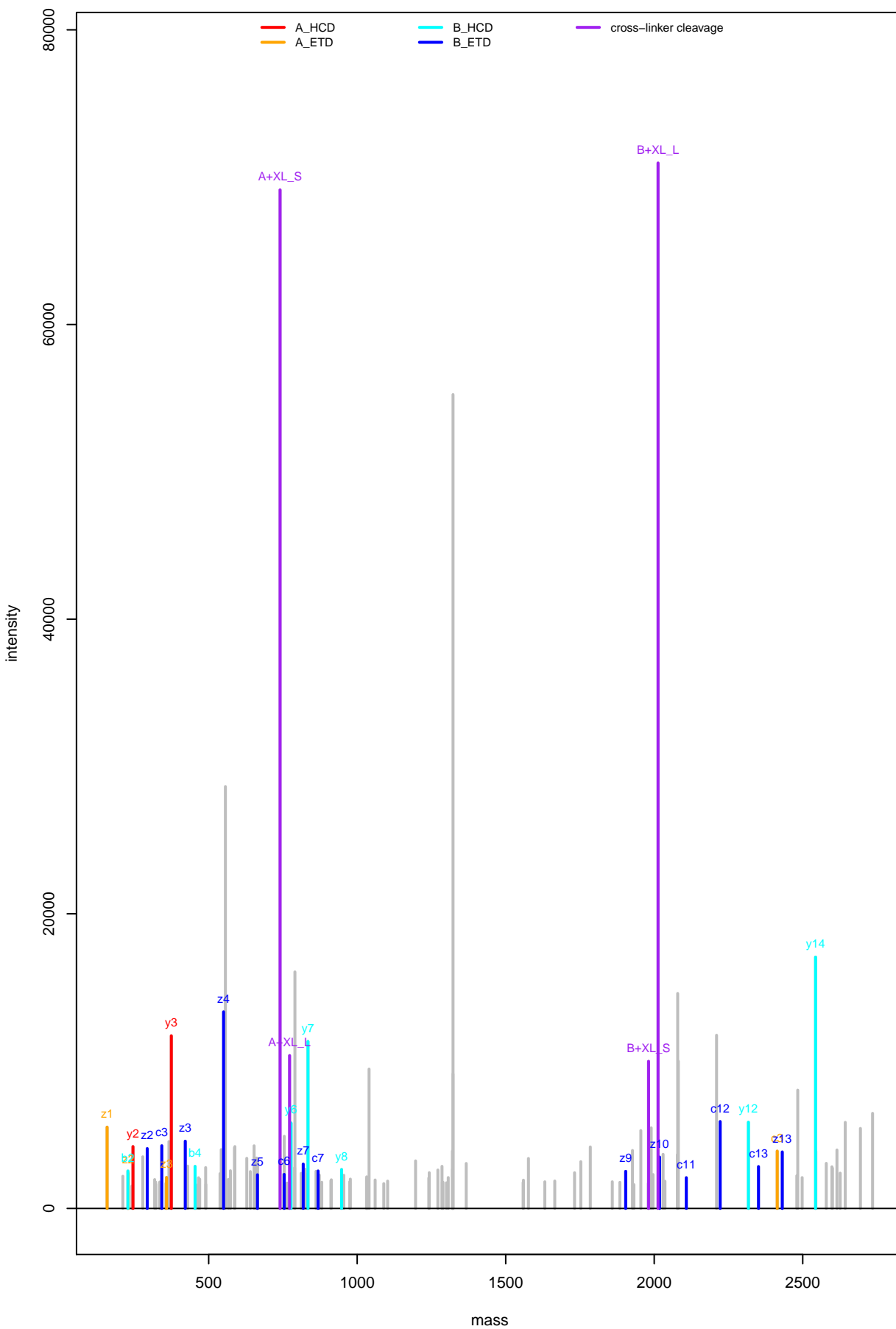

## ALKEAR+XL\_S

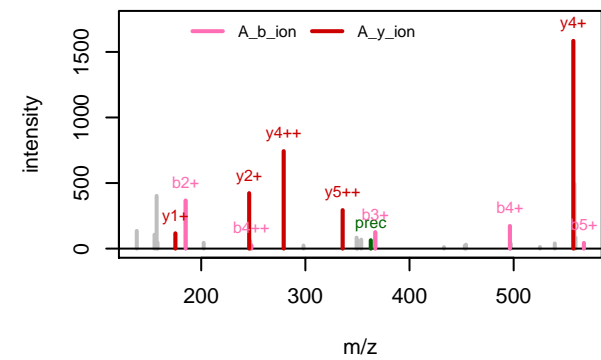

## ALKEAR+XL\_L

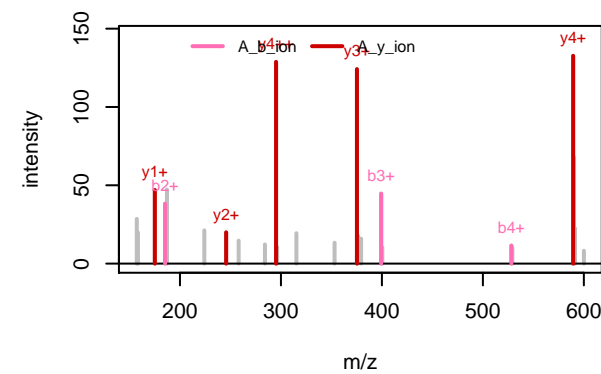

## NNPEPWNLGPNEQYK+XL\_S

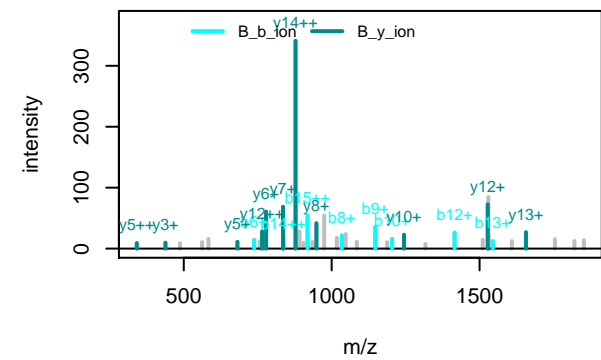

## NNPEPWNLGPNEQYK+XL\_L

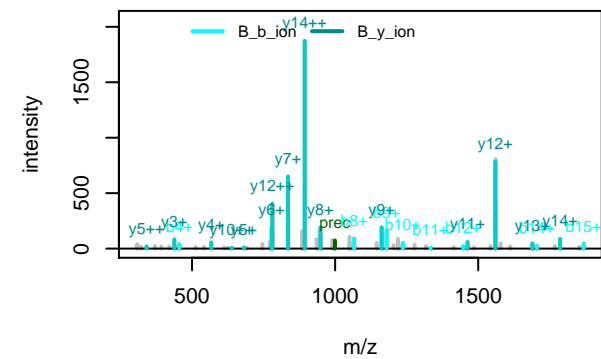

Supplement: Supplemental Data [file supp_RA117.000470_133922_0_supp_23978_fzffwf.zip › spectra_annotation/mito_DR_spectra_annotation/108-1-4-1-5-1.pdf]

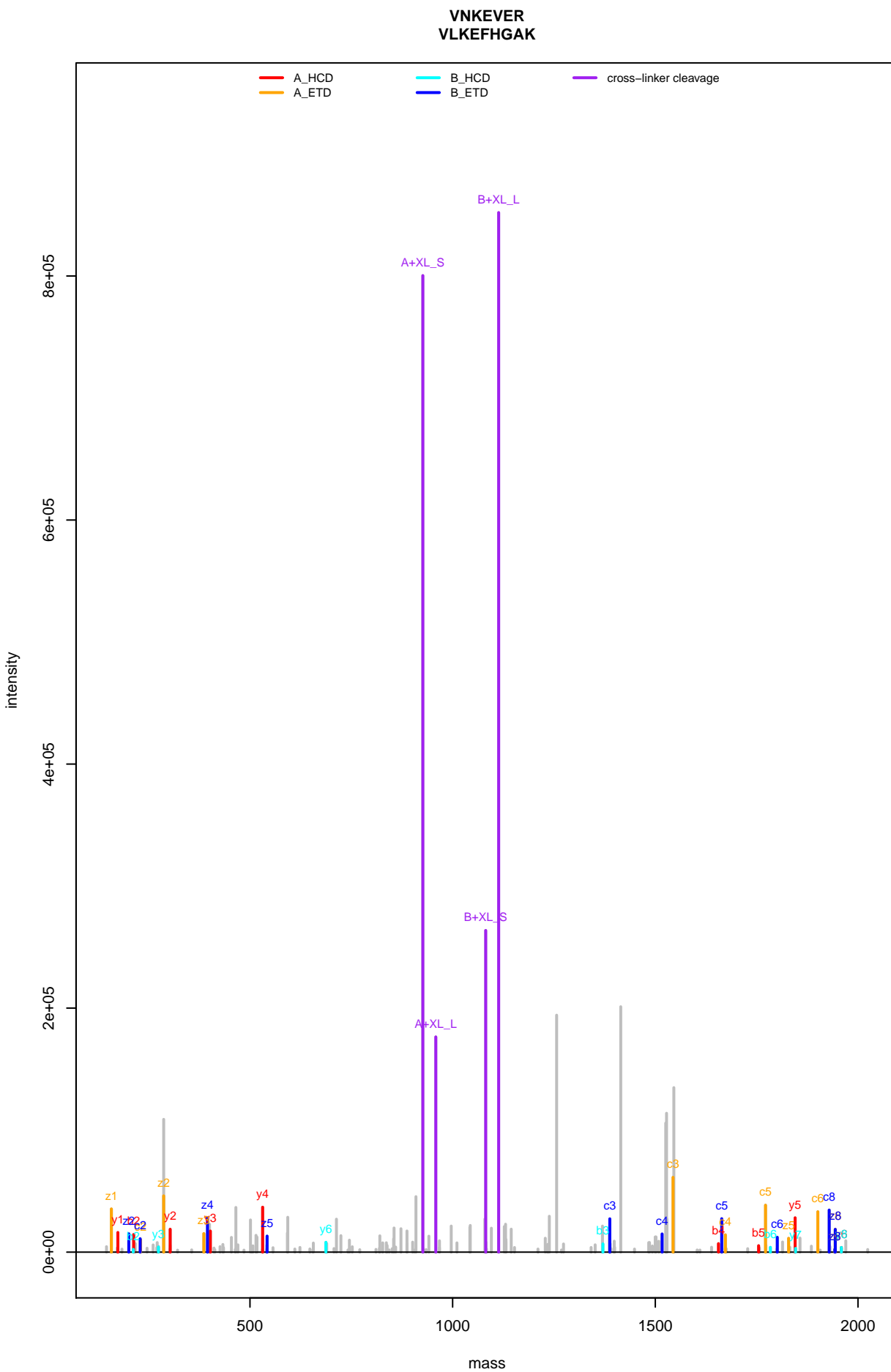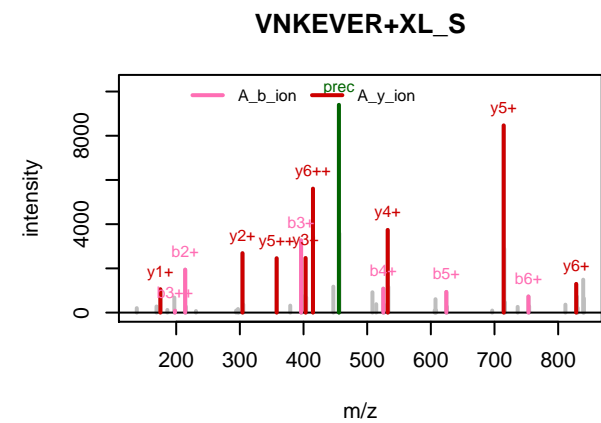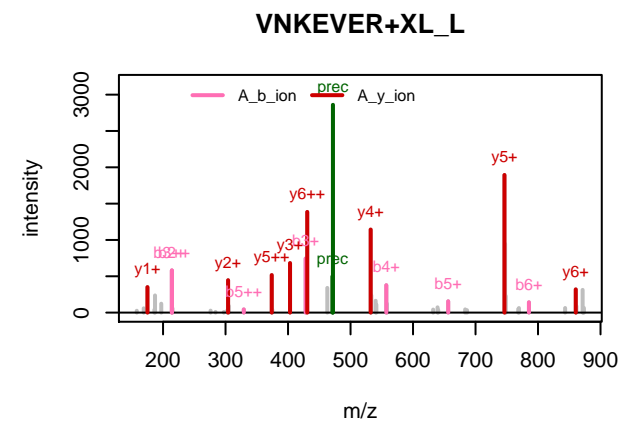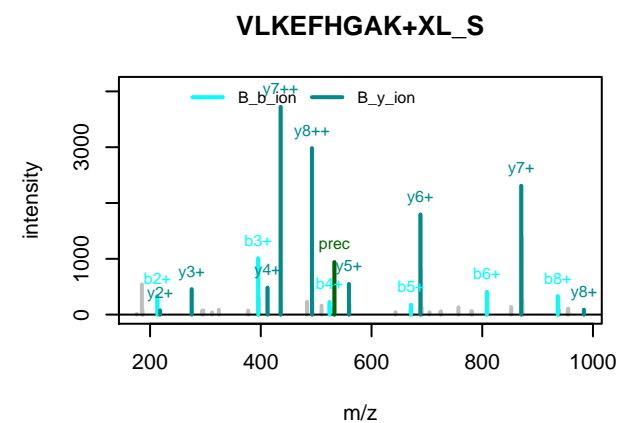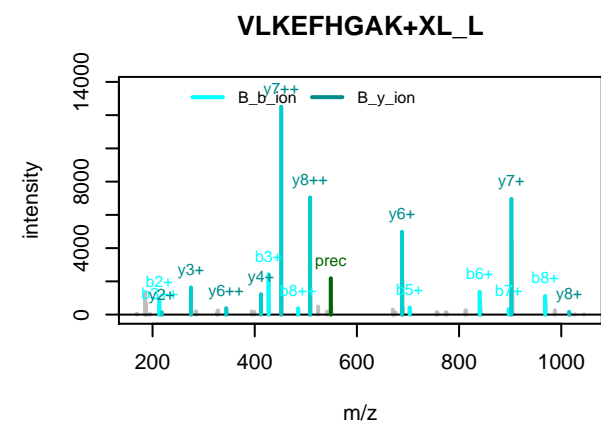

Supplement: Supplemental Data [file supp_RA117.000470_133922_0_supp_23978_fzffwf.zip › spectra_annotation/mito_DR_spectra_annotation/108-1-7-1-7-1.pdf]

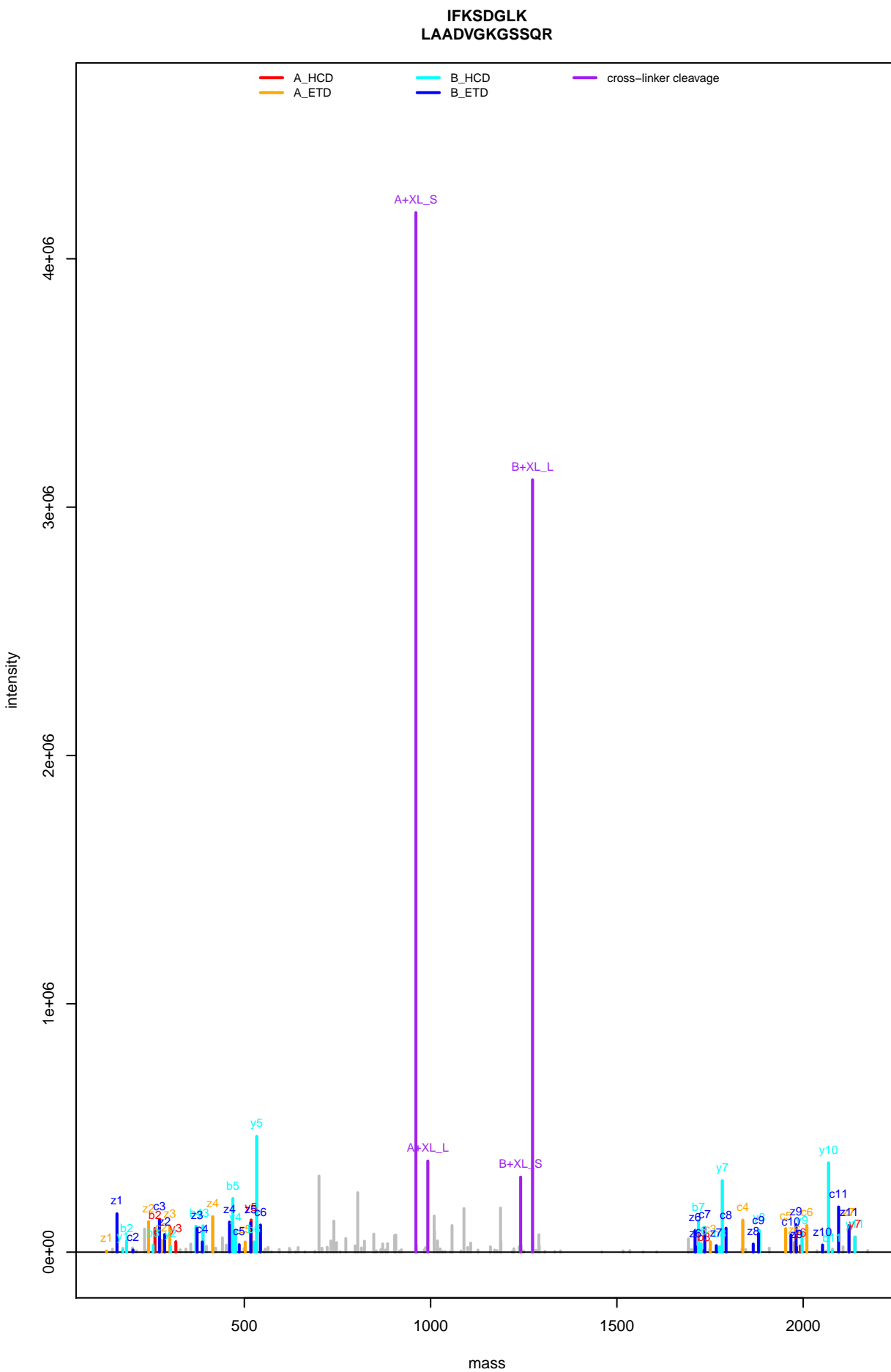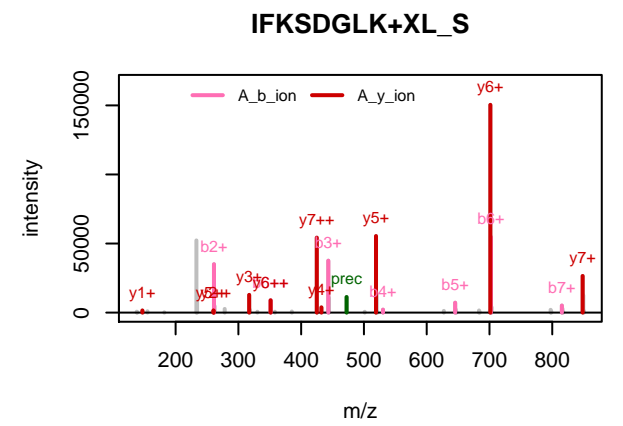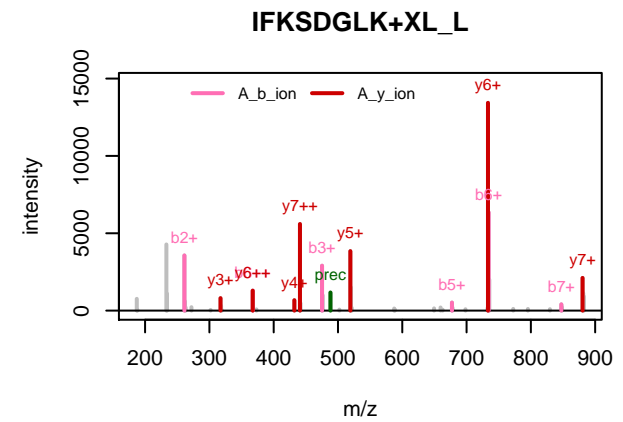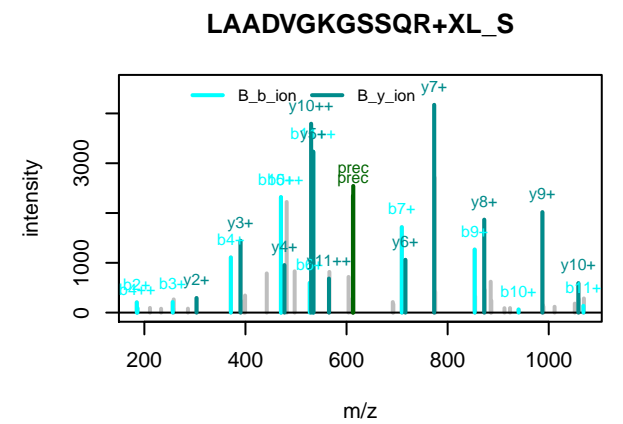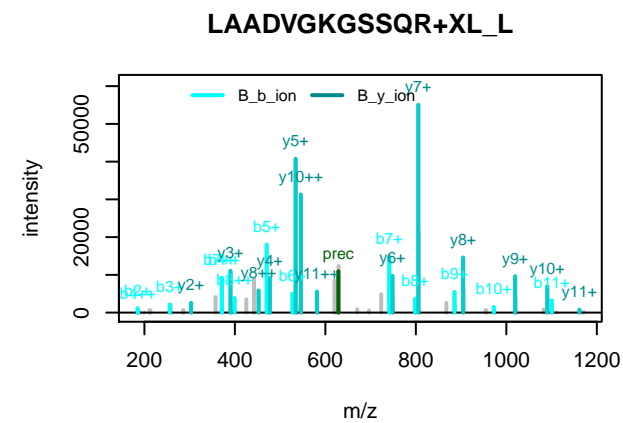

Supplement: Supplemental Data [file supp_RA117.000470_133922_0_supp_23978_fzffwf.zip › spectra_annotation/mito_DR_spectra_annotation/109-1-12-1-9-1.pdf]

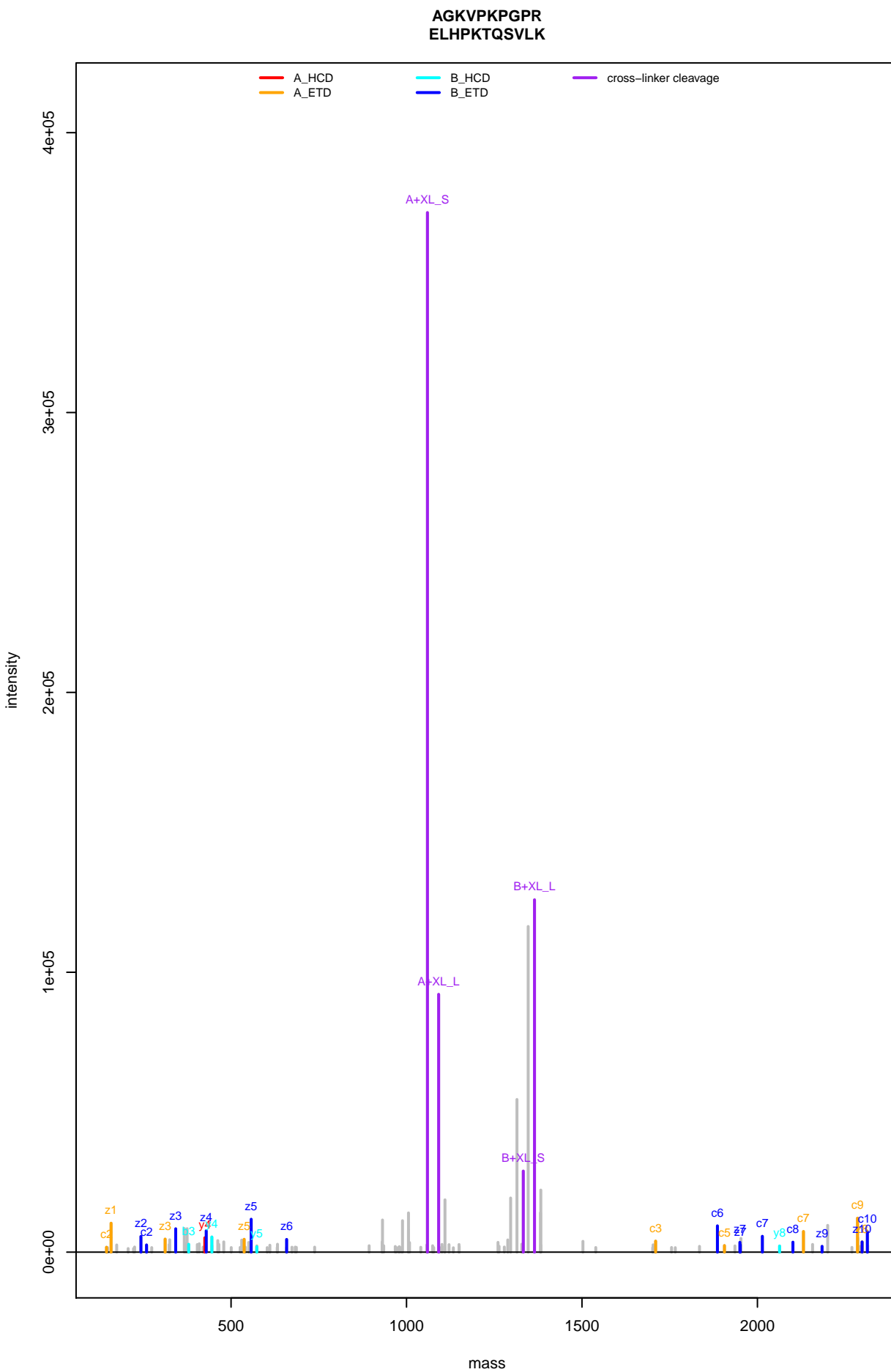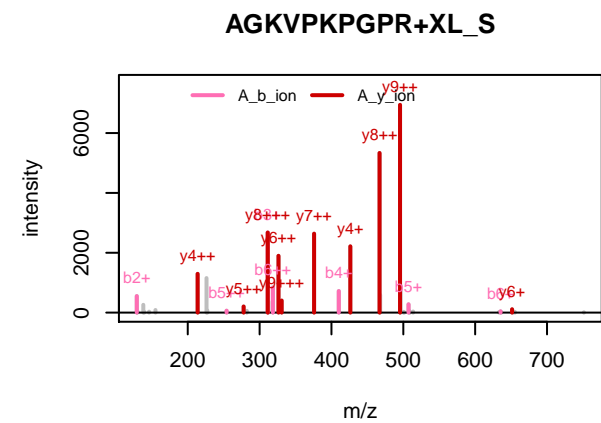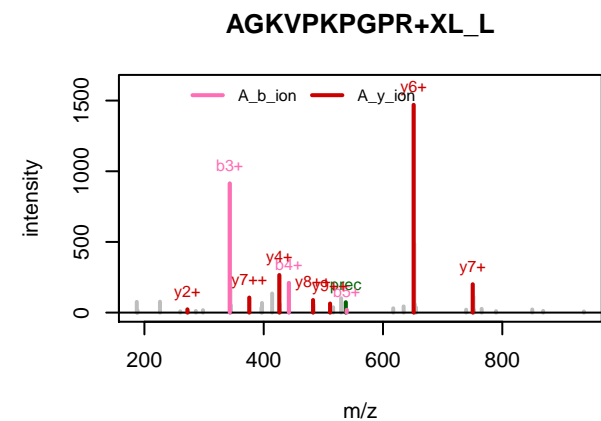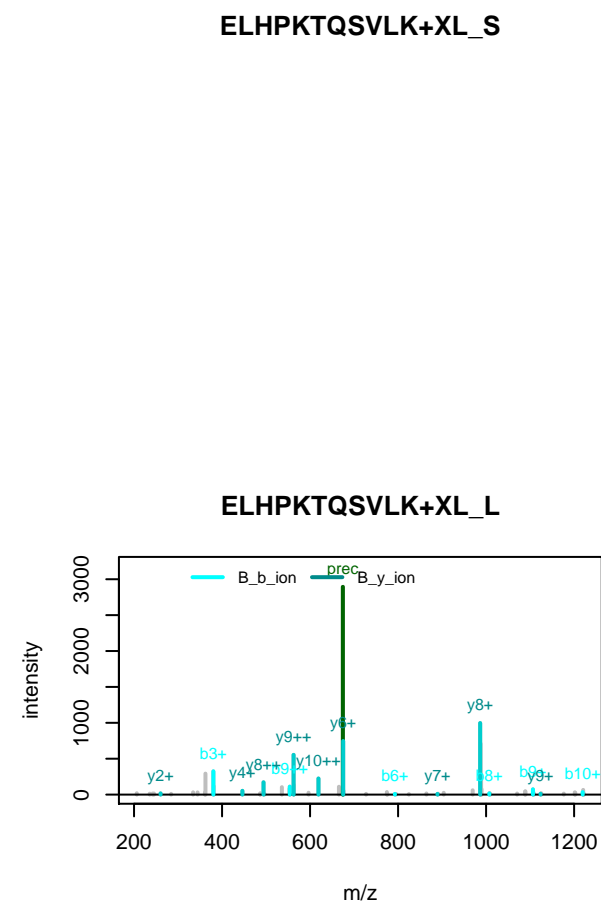

Supplement: Supplemental Data [file supp_RA117.000470_133922_0_supp_23978_fzffwf.zip › spectra_annotation/mito_DR_spectra_annotation/109-1-14-1-5-1.pdf]

LEPSKITK  
ADKLAEEHGS

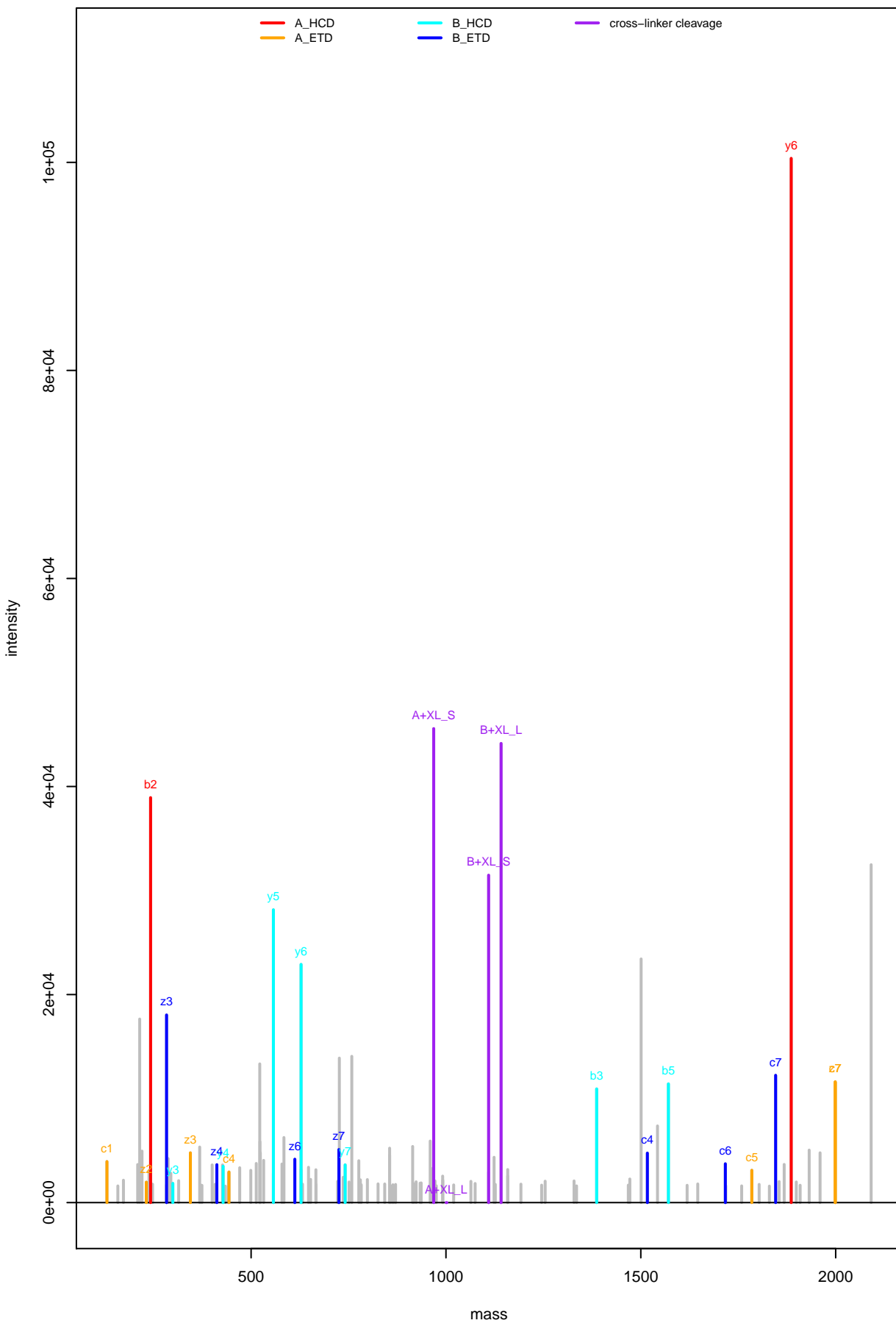

## LEPSKITK+XL\_S

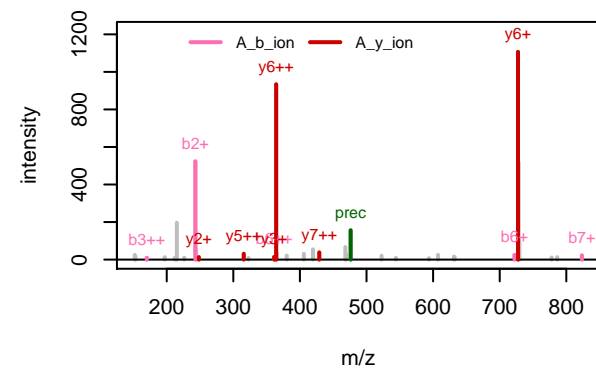

## LEPSKITK+XL\_L

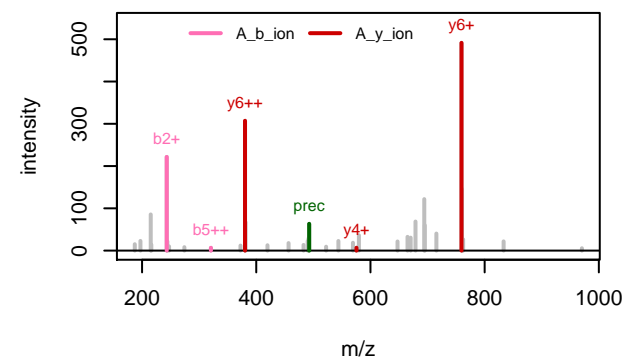

## ADKLAEEHGS+XL\_S

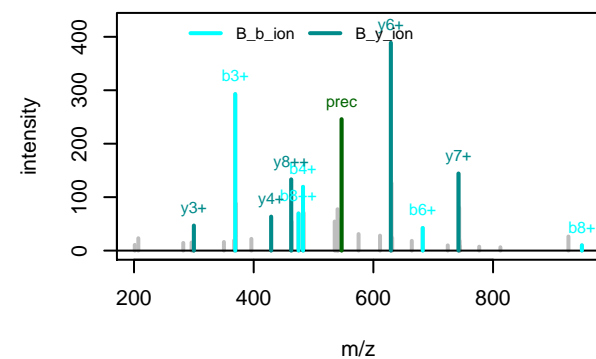

## ADKLAEEHGS+XL\_L

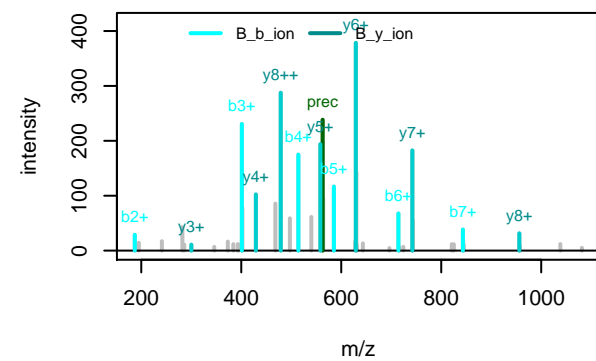

Supplement: Supplemental Data [file supp_RA117.000470_133922_0_supp_23978_fzffwf.zip › spectra_annotation/mito_DR_spectra_annotation/109-1-24-1-3-1.pdf]

NIQKITK  
MVAAAKYAR

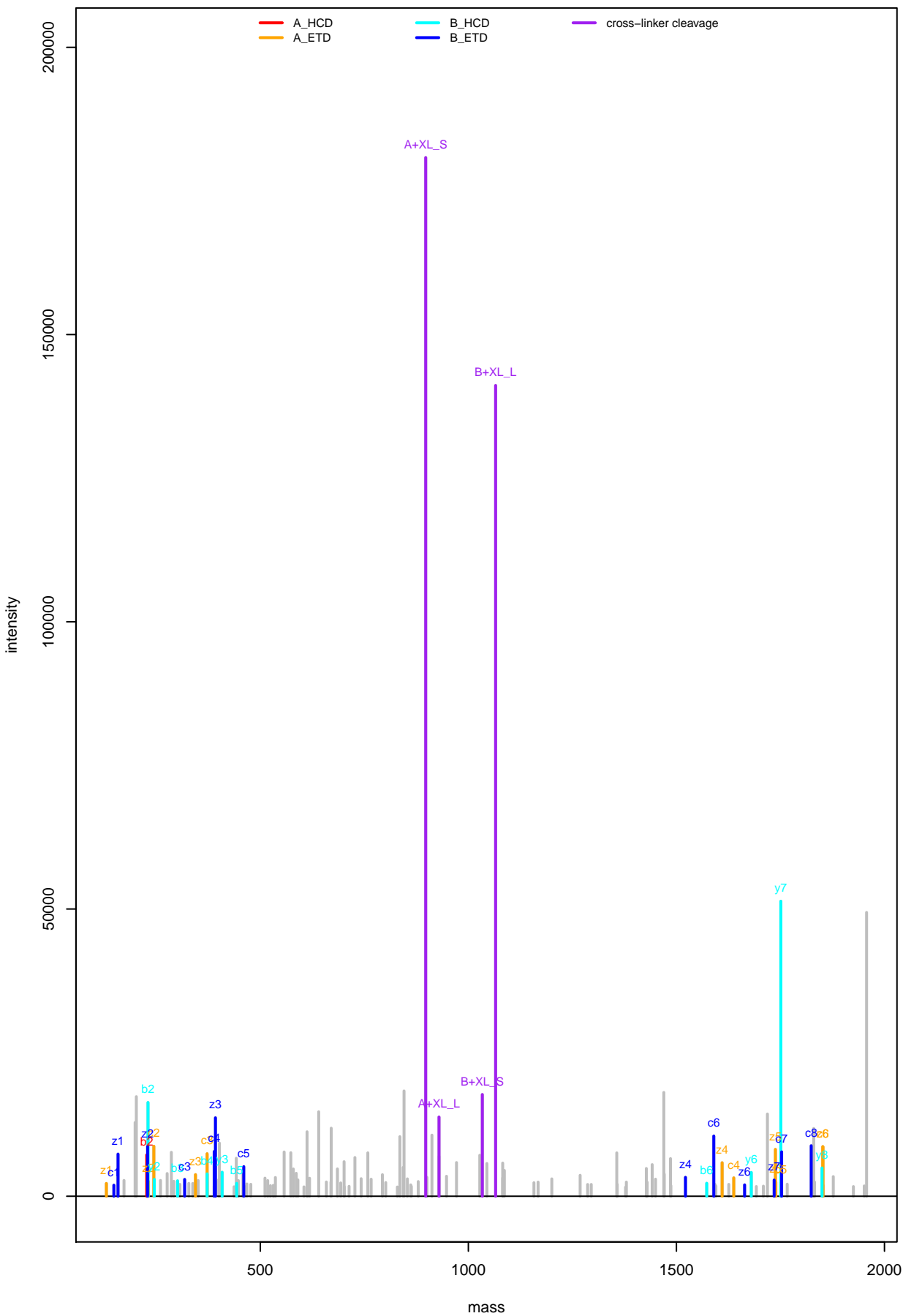**NIQKITK+XL\_S**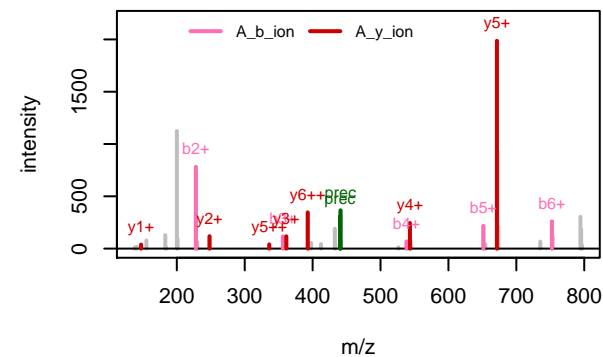**NIQKITK+XL\_L**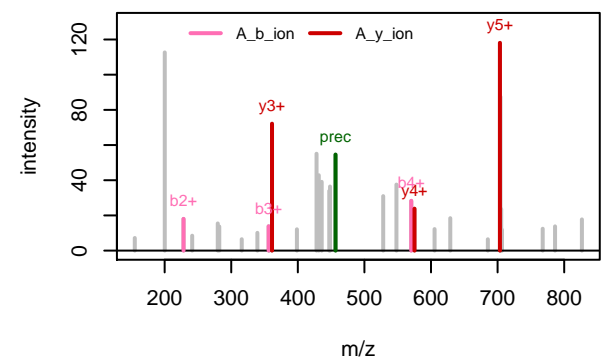

## MVAALKYAR+XL\_S

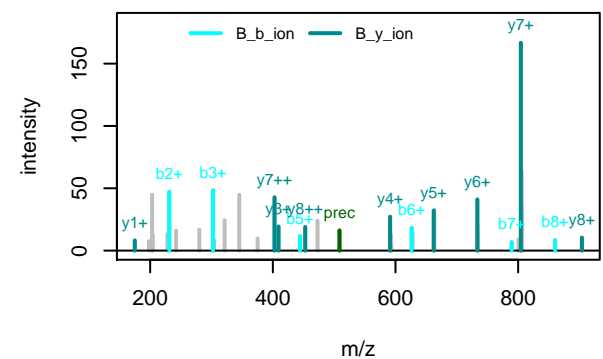MVA<sup>AA</sup>KYAR+XL\_L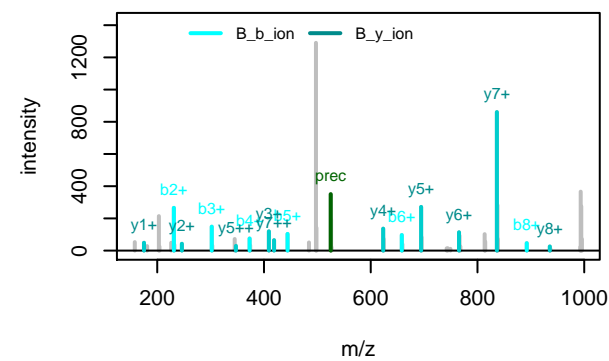

Supplement: Supplemental Data [file supp_RA117.000470_133922_0_supp_23978_fzffwf.zip › spectra_annotation/mito_DR_spectra_annotation/109-1-4-1-3-1.pdf]

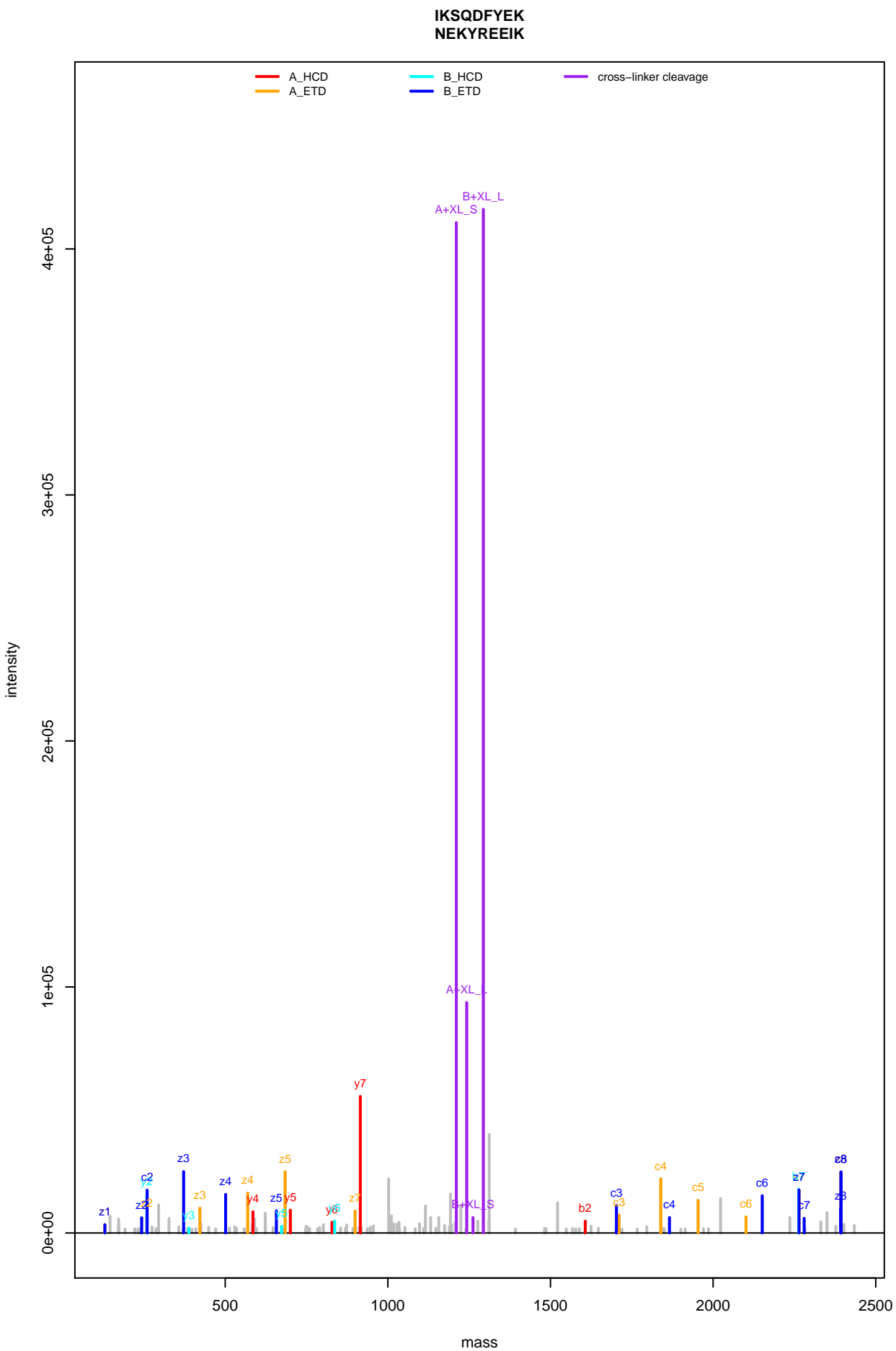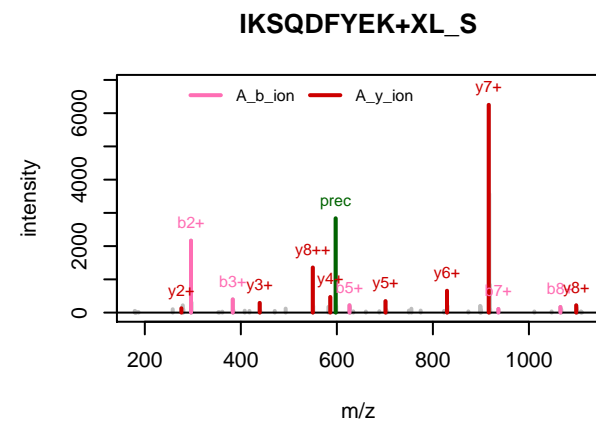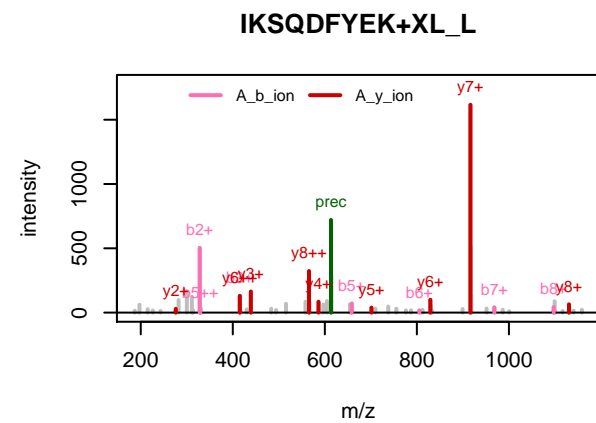

**NEKYREEIK+XL\_S**

**NEKYREEIK+XL\_L**

Supplement: Supplemental Data [file supp_RA117.000470_133922_0_supp_23978_fzffwf.zip › spectra_annotation/mito_DR_spectra_annotation/109-1-6-1-4-1.pdf]

# ADIKAPEDK SDGKISEQSDAK

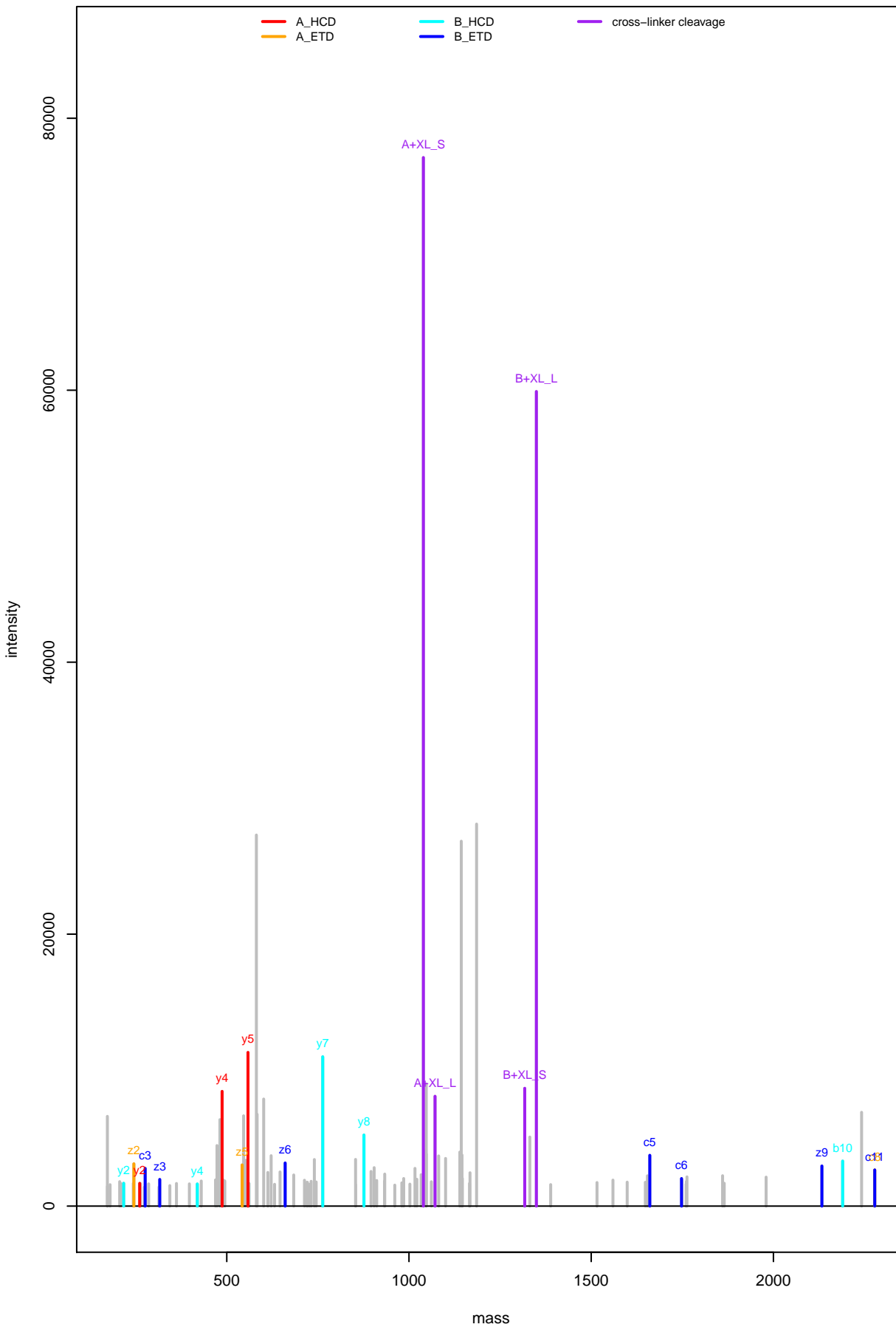

## ADIKAPEDK+XL\_S

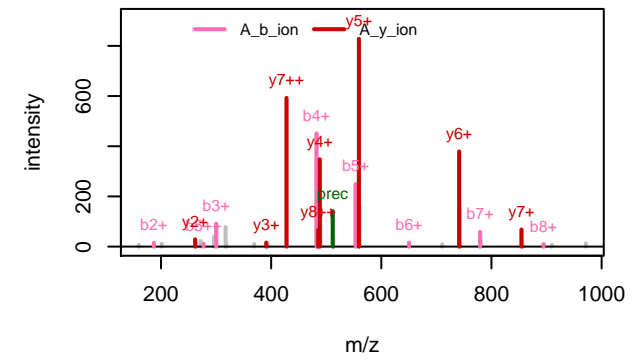

## ADIKAPEDK+XL\_L

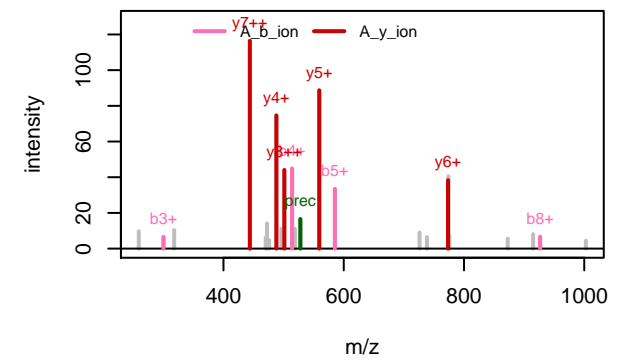

## SDGKISEQSDAK+XL\_S

## SDGKISEQSDAK+XL\_L

Supplement: Supplemental Data [file supp_RA117.000470_133922_0_supp_23978_fzffwf.zip › spectra_annotation/mito_DR_spectra_annotation/11-1-1-1-6-1.pdf]

**RLDELKR**  
**NNPEPWNKLGPNQYK**

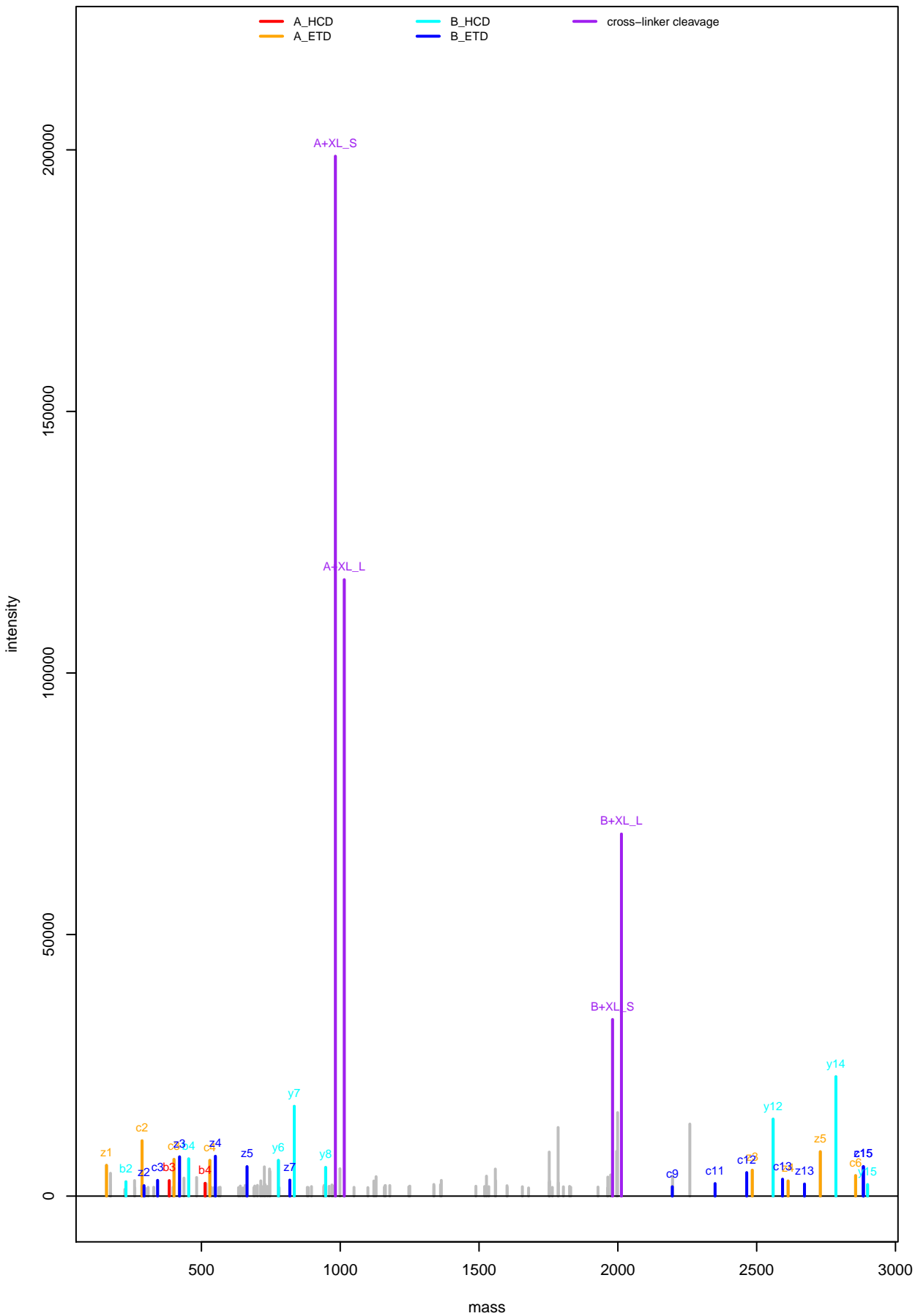

**RLDELKR+XL\_S**

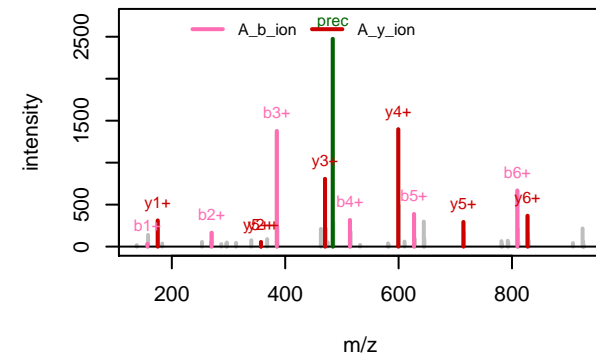

**RLDELKR+XL\_L**

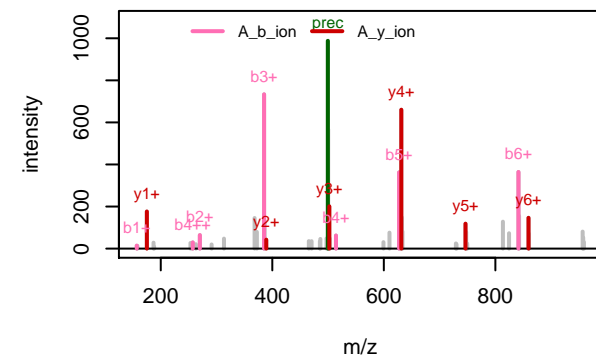

**NNPEPWNKLGPNQYK+XL\_S**

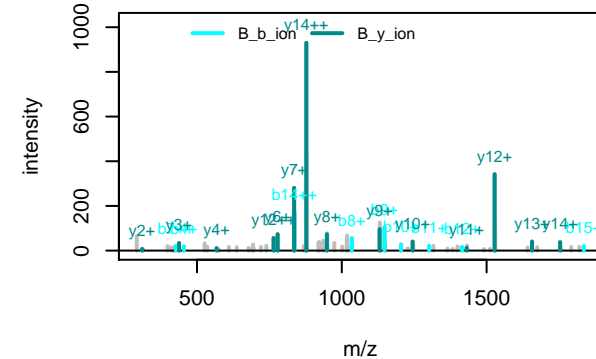

**NNPEPWNKLGPNQYK+XL\_L**

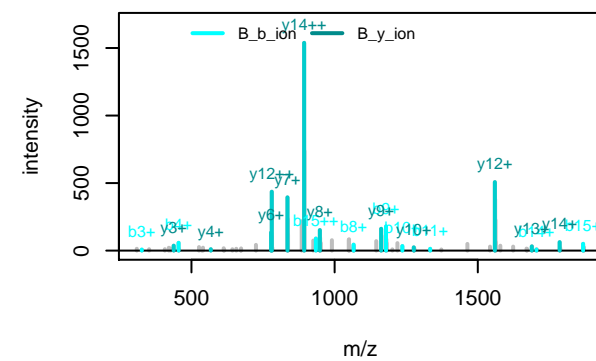

Supplement: Supplemental Data [file supp_RA117.000470_133922_0_supp_23978_fzffwf.zip › spectra_annotation/mito_DR_spectra_annotation/11-1-10-1-4-1.pdf]

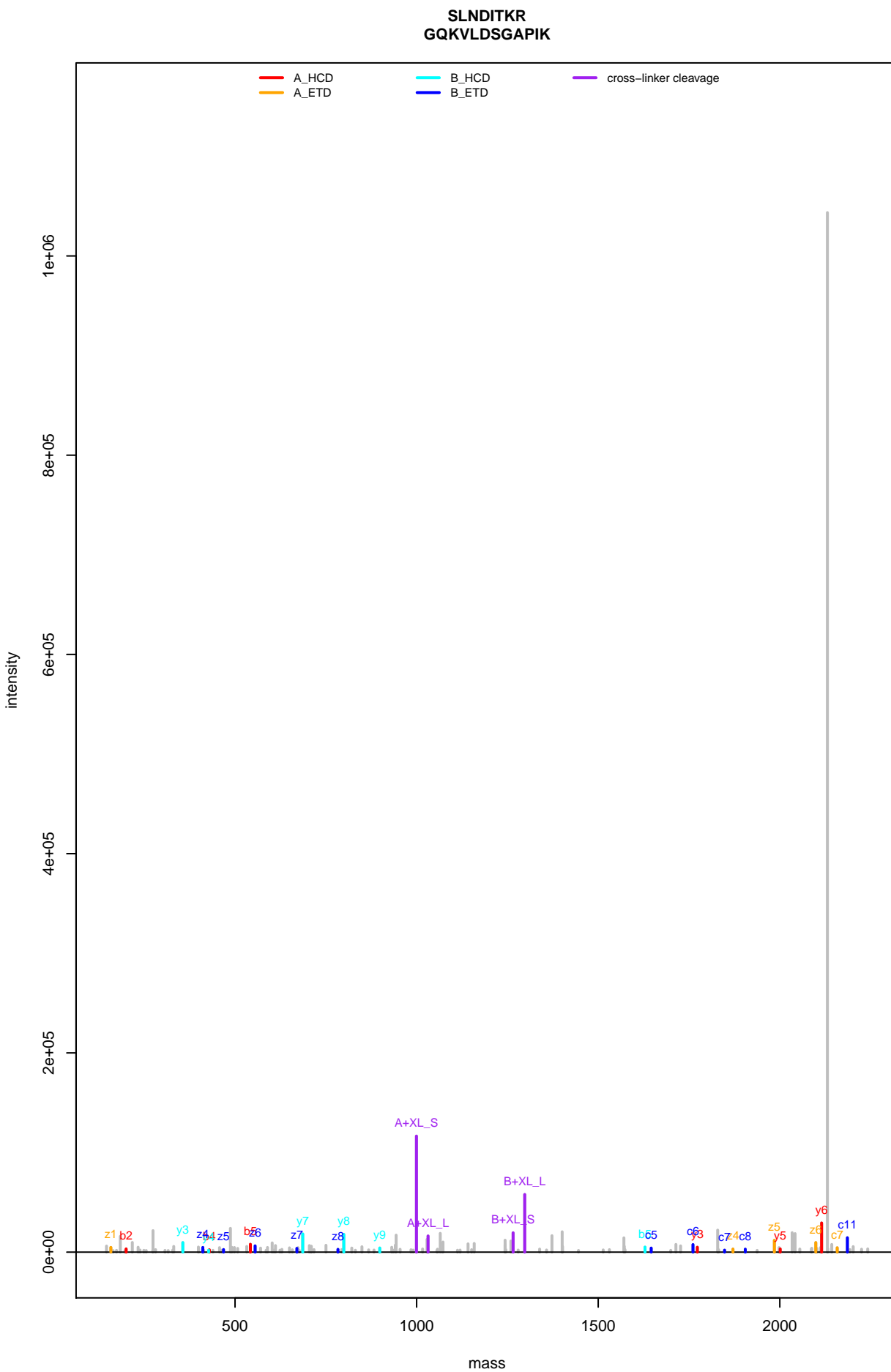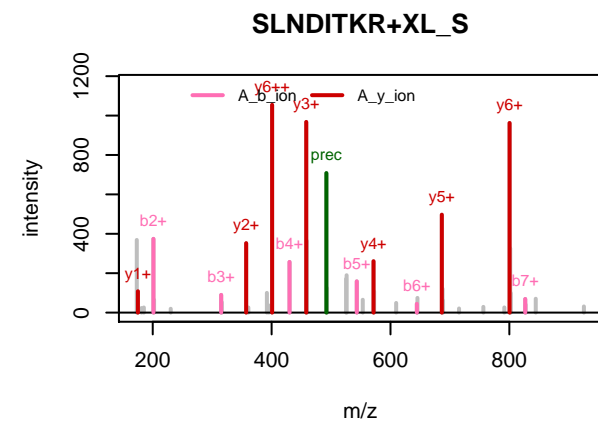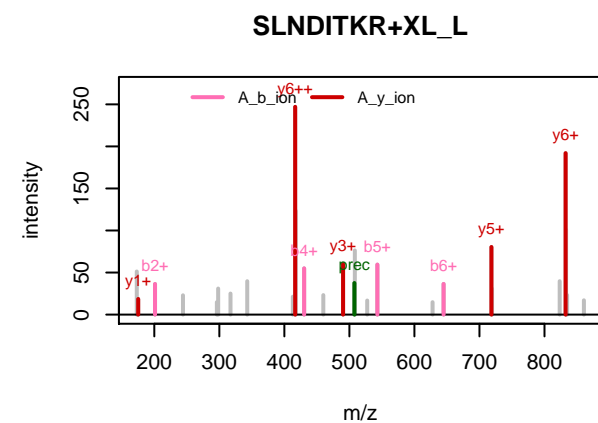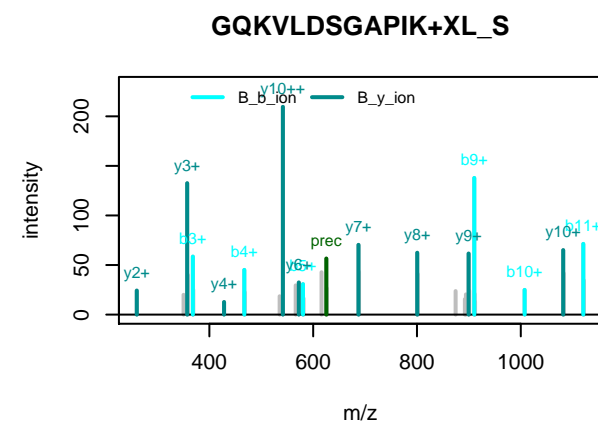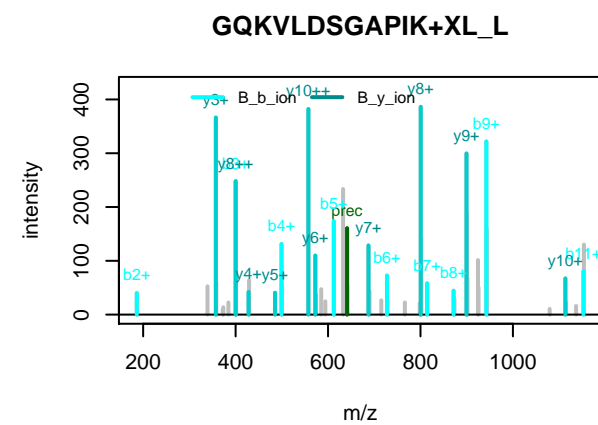

Supplement: Supplemental Data [file supp_RA117.000470_133922_0_supp_23978_fzffwf.zip › spectra_annotation/mito_DR_spectra_annotation/11-1-12-1-1-1.pdf]

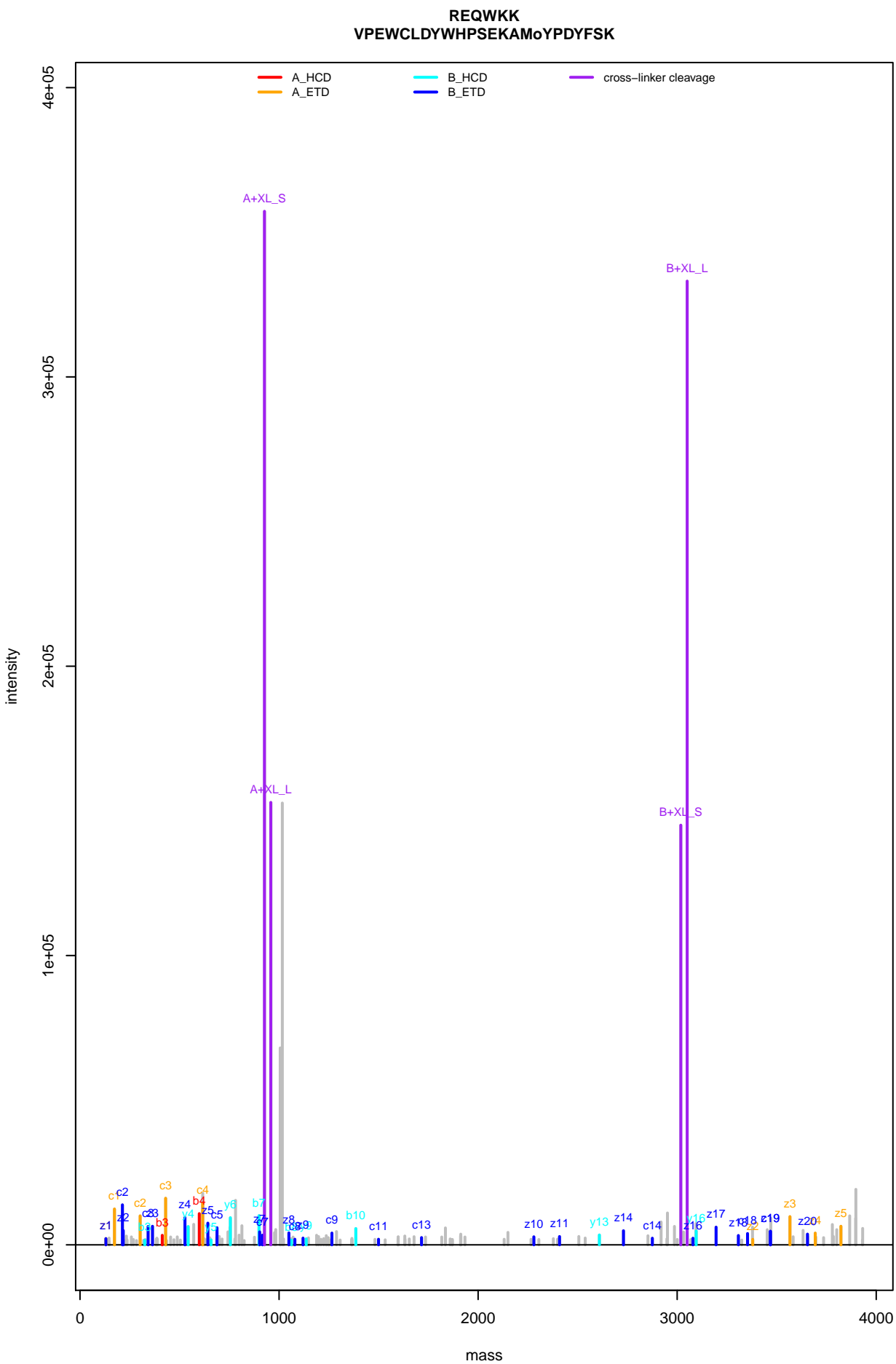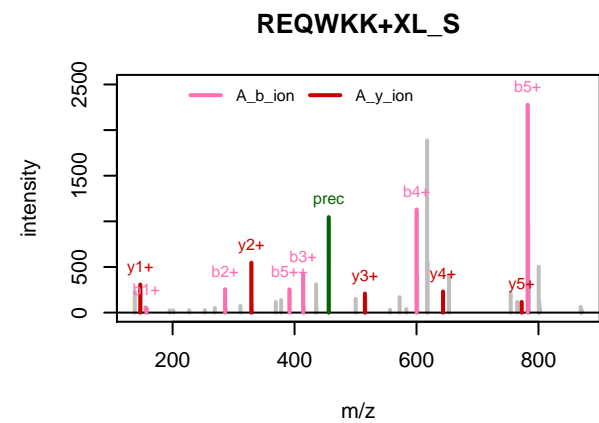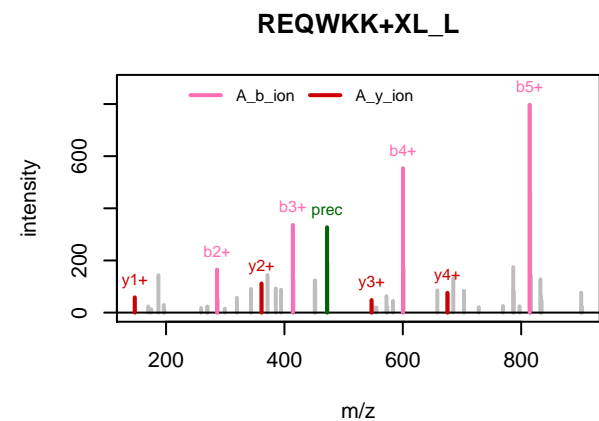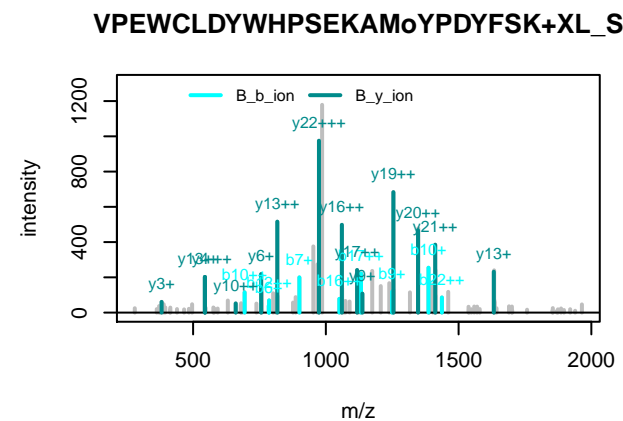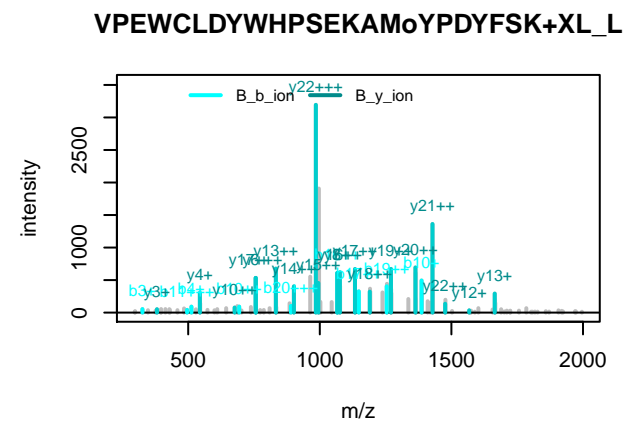

Supplement: Supplemental Data [file supp_RA117.000470_133922_0_supp_23978_fzffwf.zip › spectra_annotation/mito_DR_spectra_annotation/11-1-39-1-11-1.pdf]

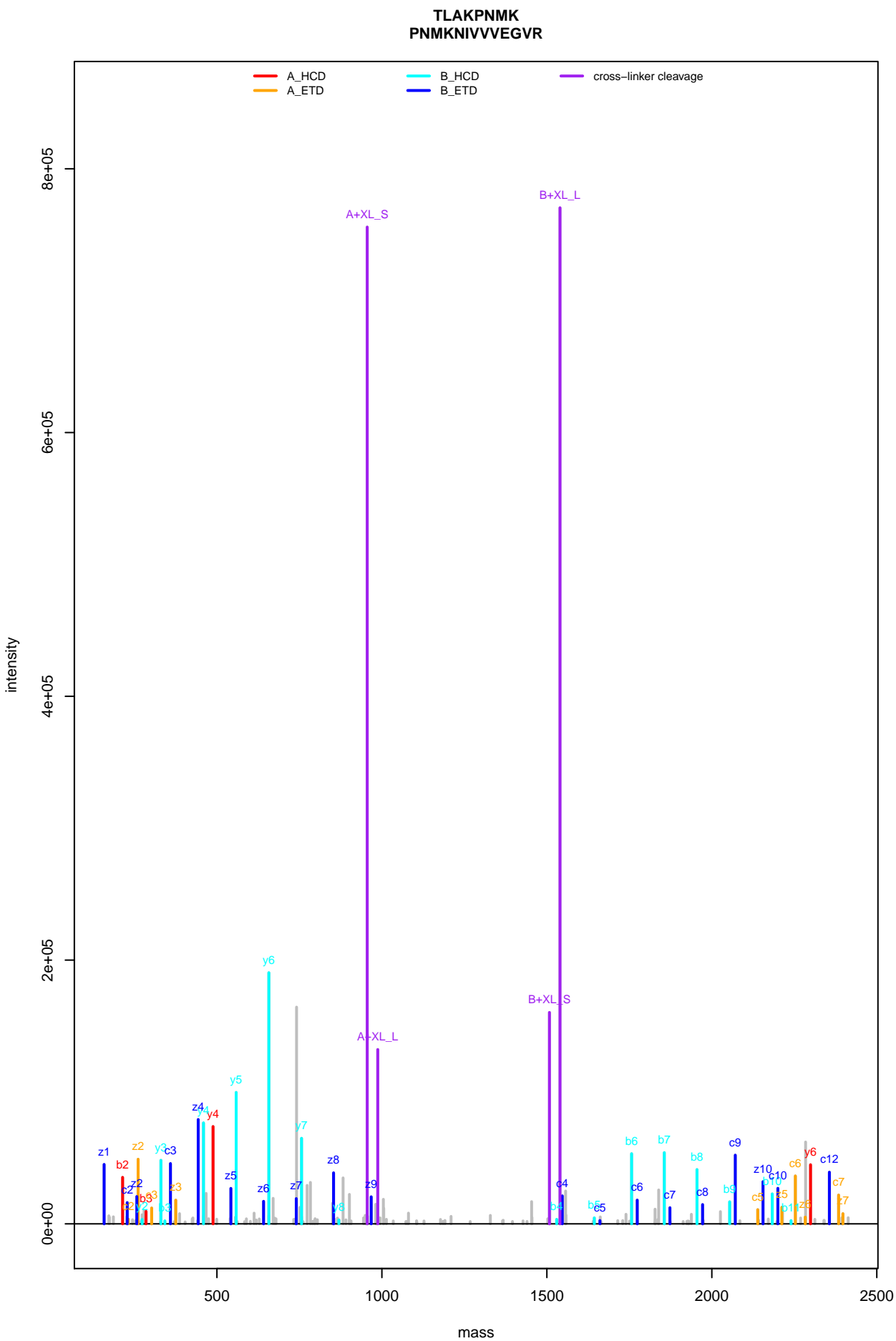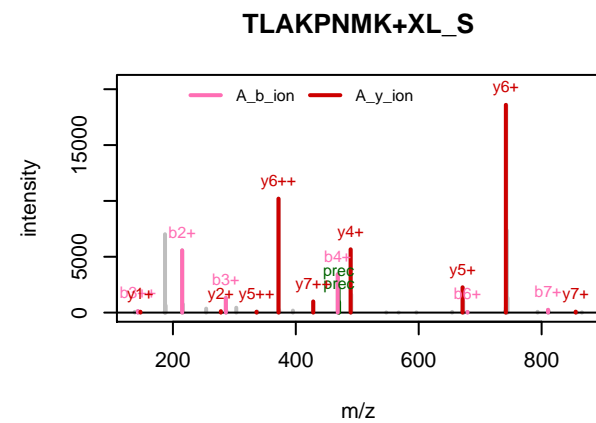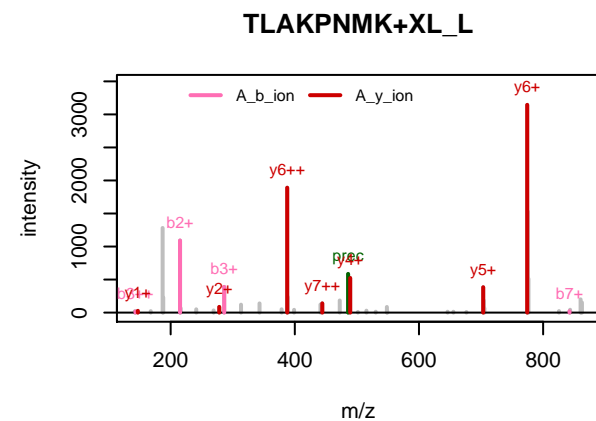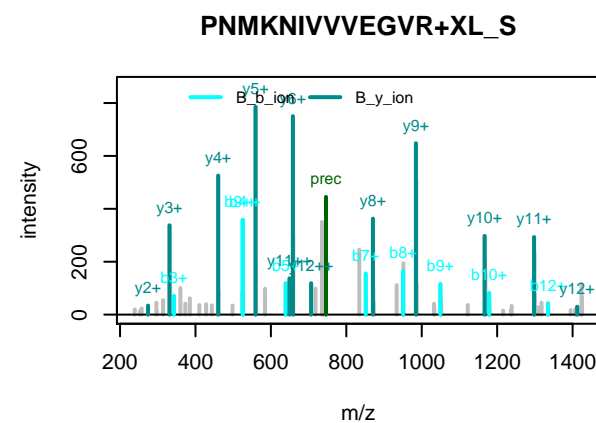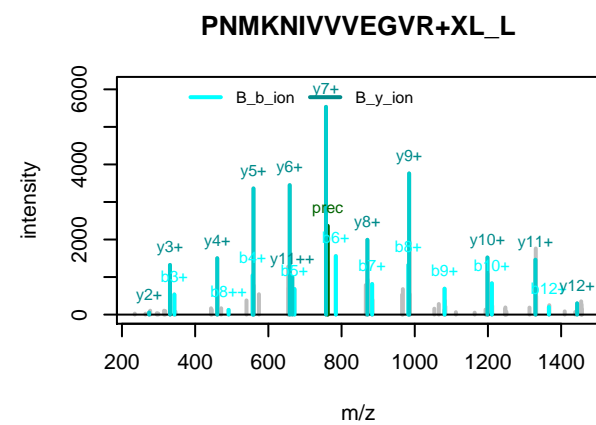

Supplement: Supplemental Data [file supp_RA117.000470_133922_0_supp_23978_fzffwf.zip › spectra_annotation/mito_DR_spectra_annotation/11-1-5-1-7-1.pdf]

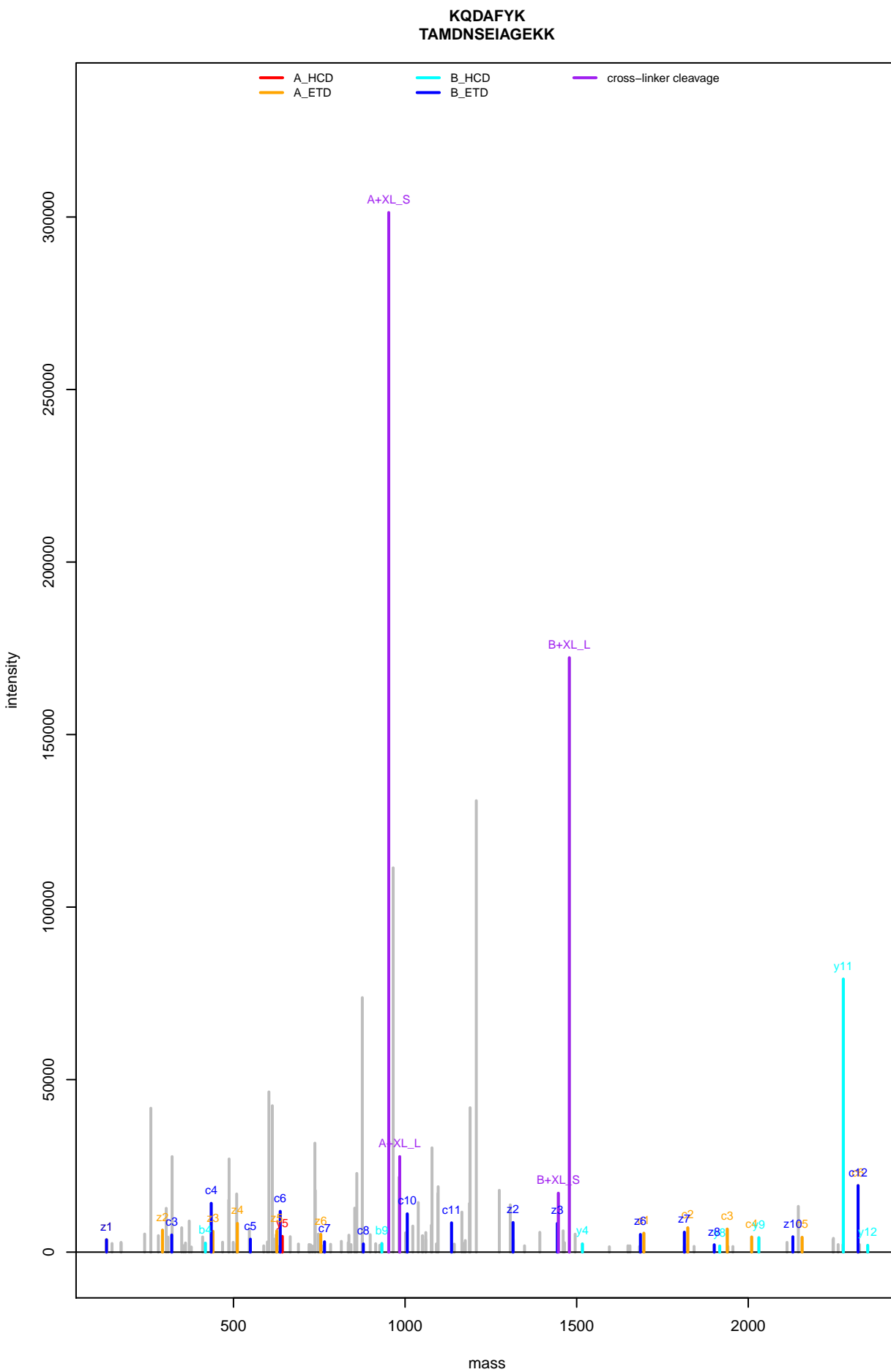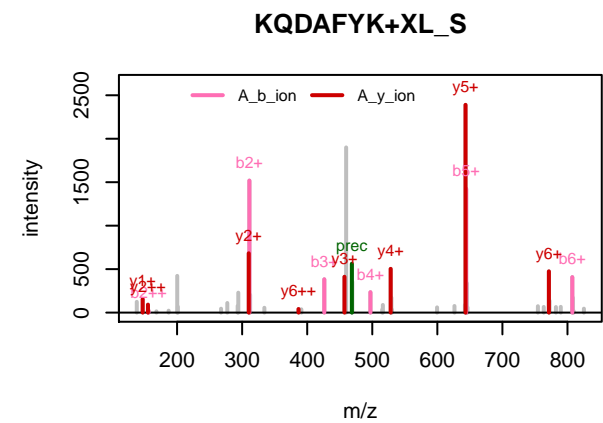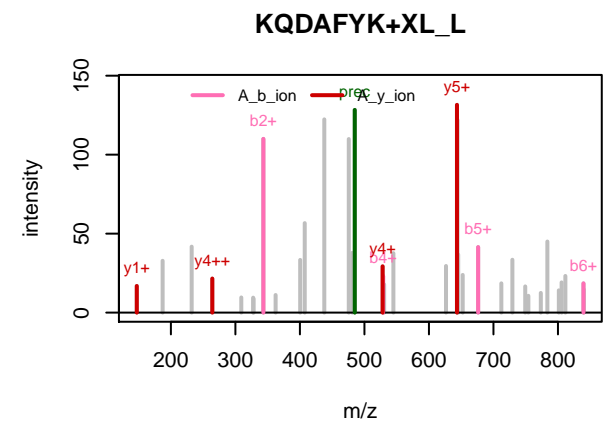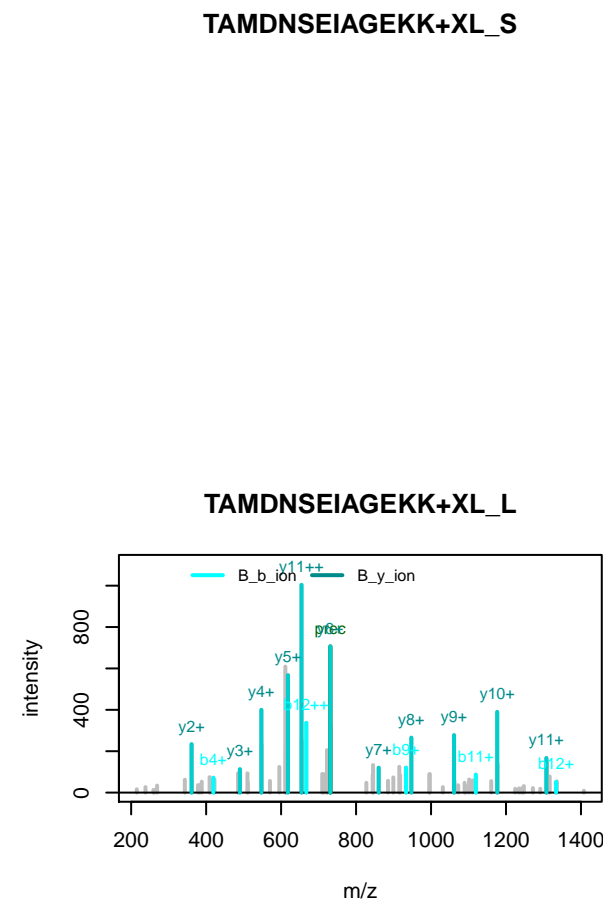

Supplement: Supplemental Data [file supp_RA117.000470_133922_0_supp_23978_fzffwf.zip › spectra_annotation/mito_DR_spectra_annotation/110-1-4-1-3-1.pdf]

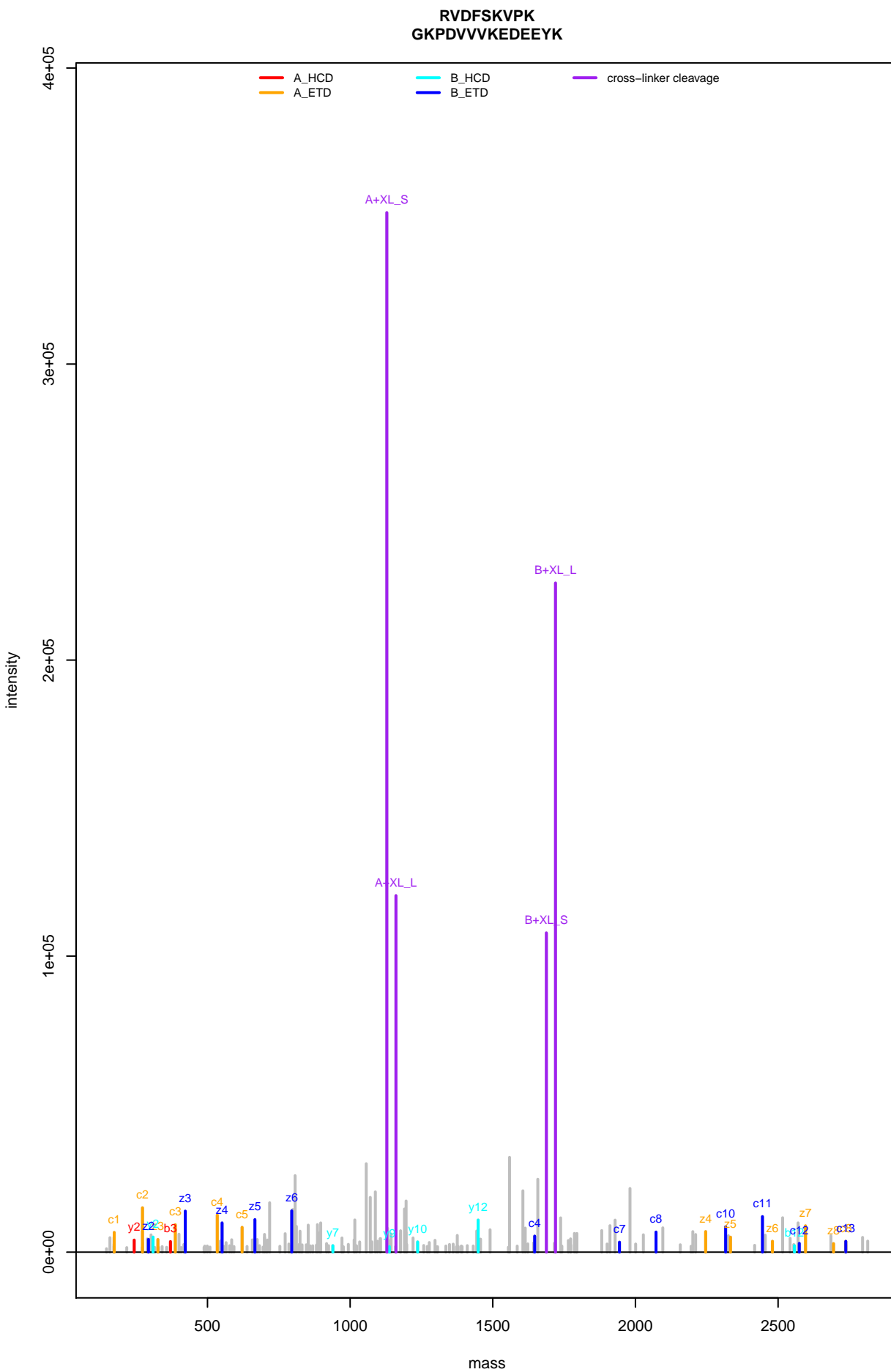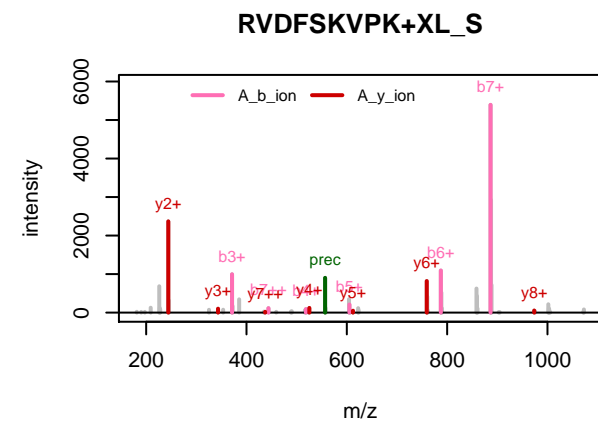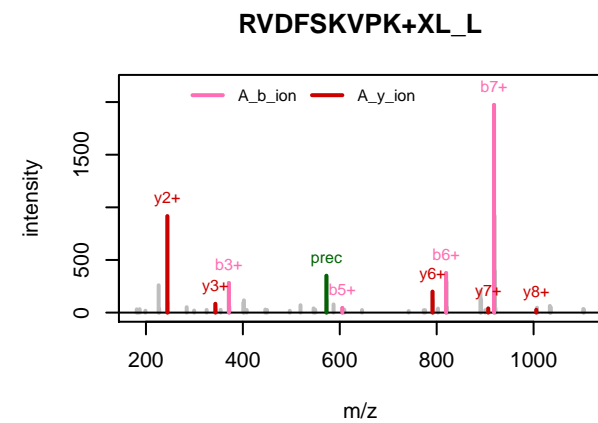

**GKPDVVVKEDEEYK+XL\_S**

**GKPDVVVKEDEEYK+XL\_L**

Supplement: Supplemental Data [file supp_RA117.000470_133922_0_supp_23978_fzffwf.zip › spectra_annotation/mito_DR_spectra_annotation/111-1-10-1-11-1.pdf]

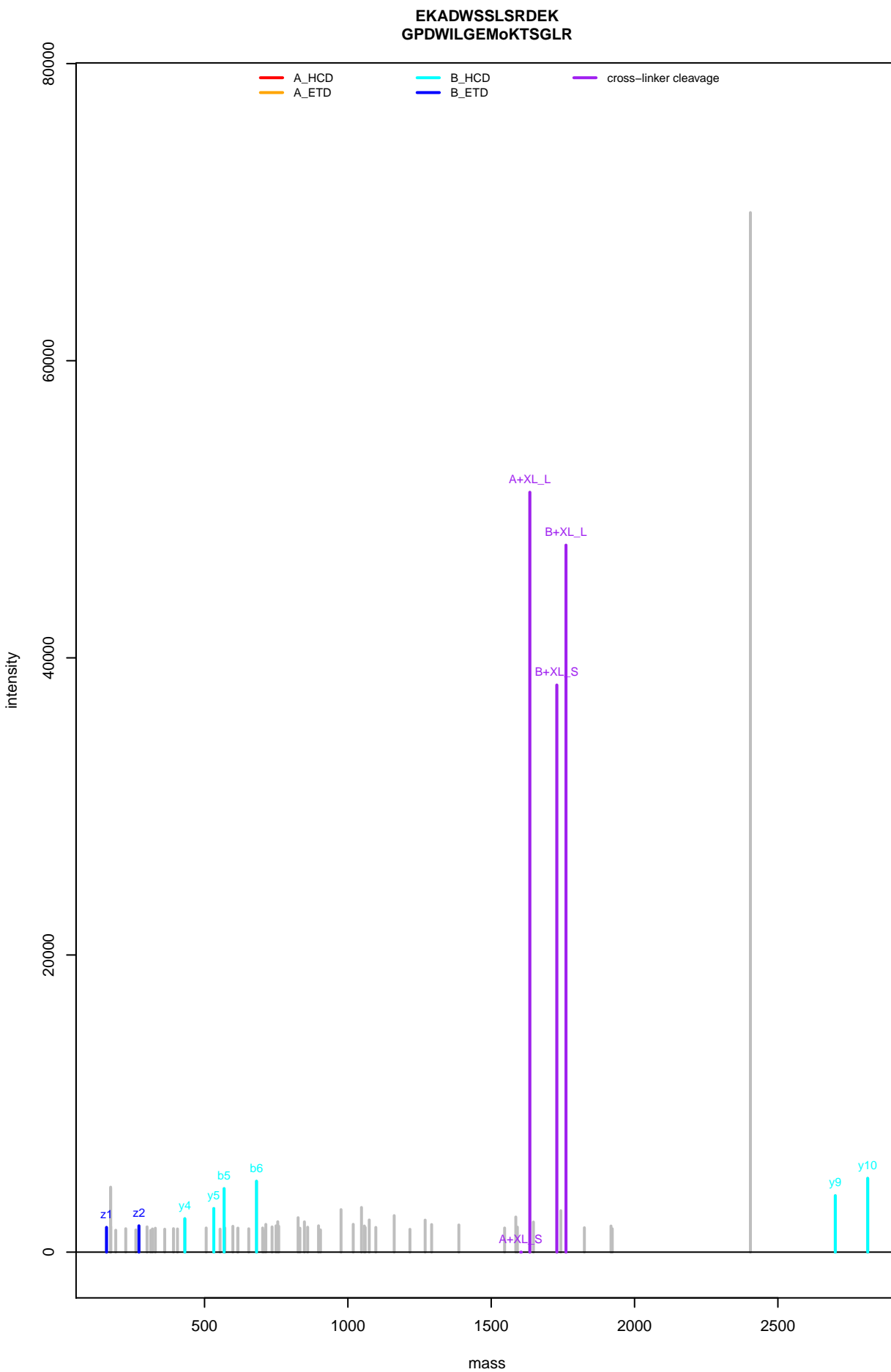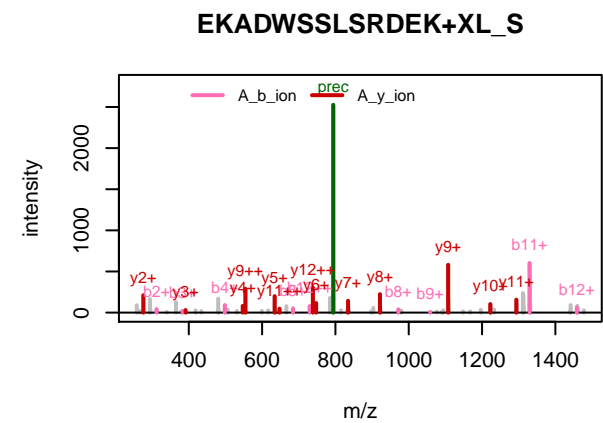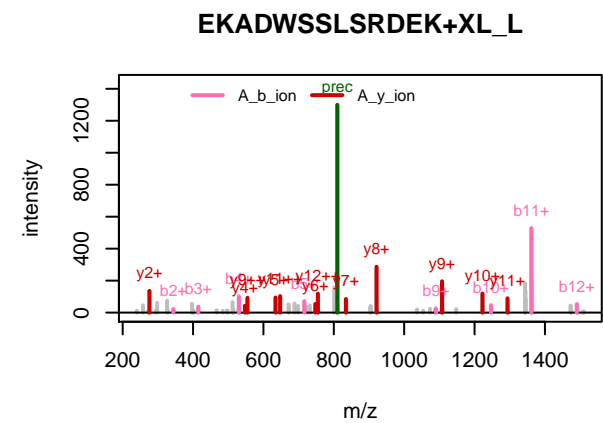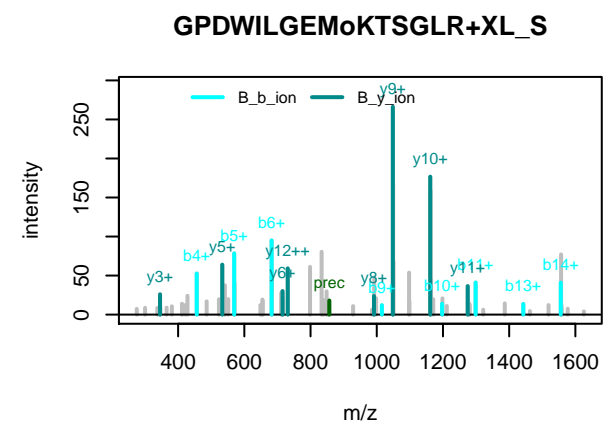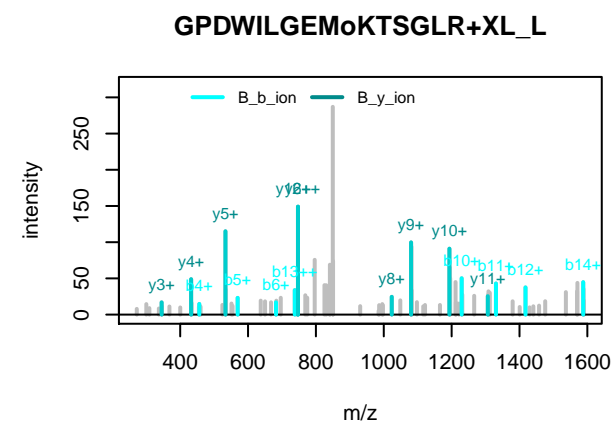

Supplement: Supplemental Data [file supp_RA117.000470_133922_0_supp_23978_fzffwf.zip › spectra_annotation/mito_DR_spectra_annotation/111-1-28-1-16-1.pdf]

ADKLAEEHGS  
PGLVDDFEKK

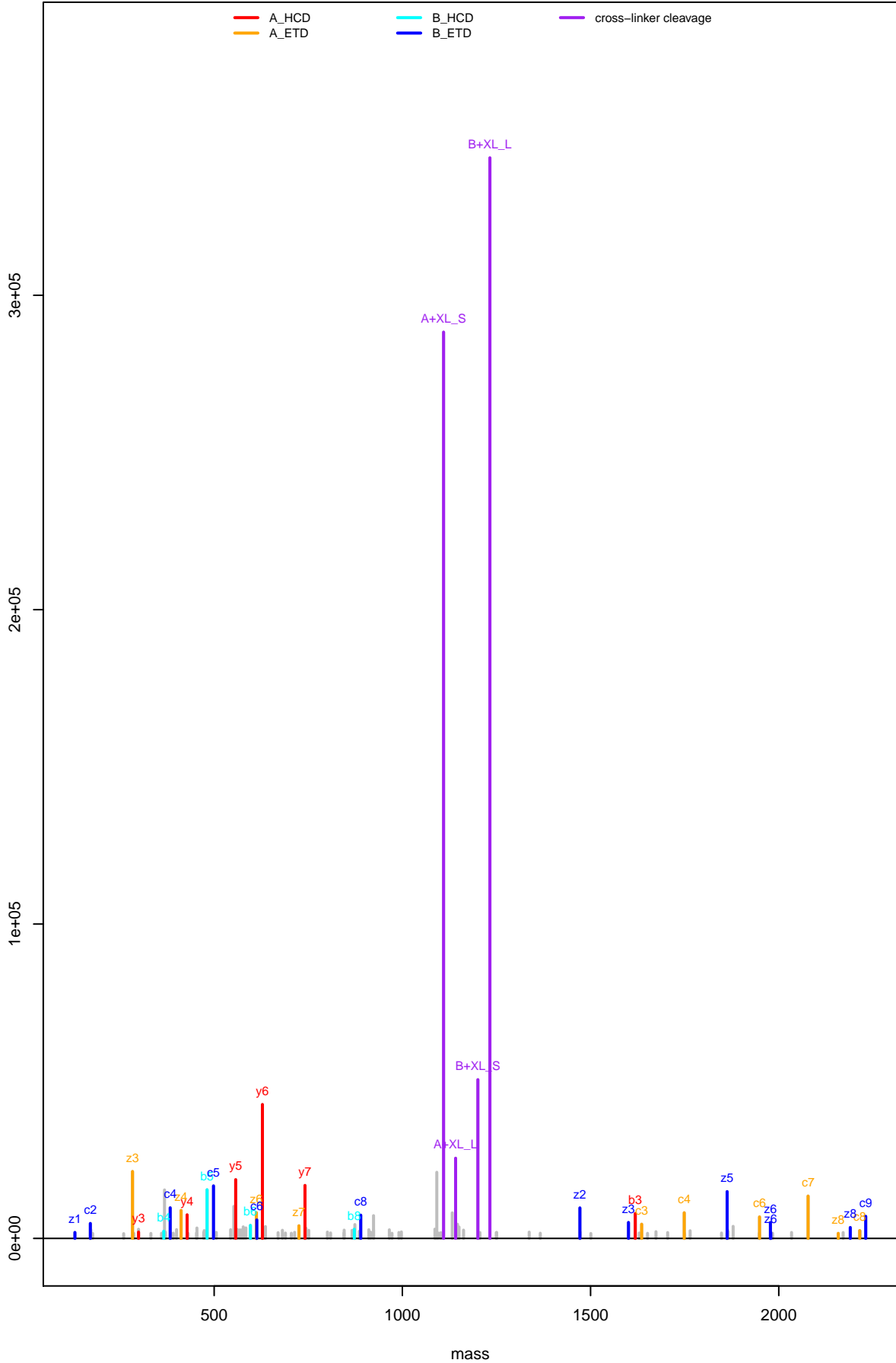

## ADKLAEEHGS+XL S

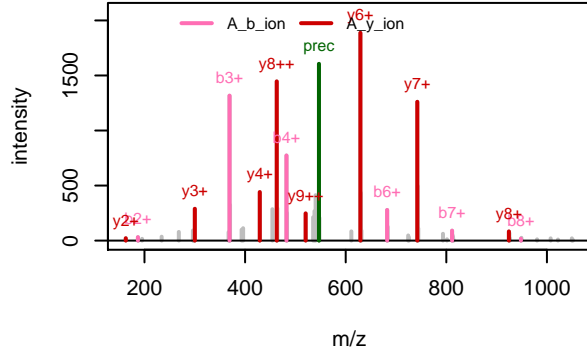

## ADKLAEEHGS+XL L

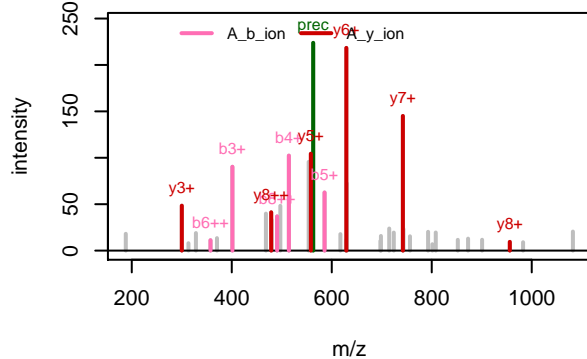

**PGLVDDFEKK+XL S**

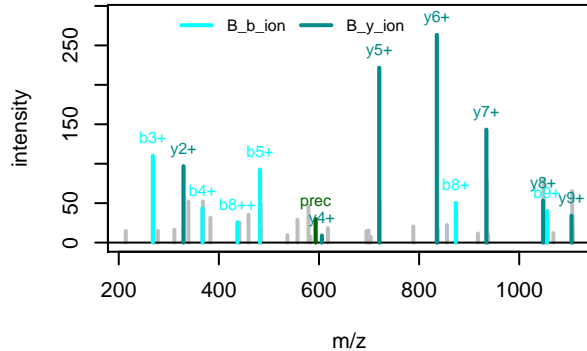

**PGLVDDFEKK+XL L**

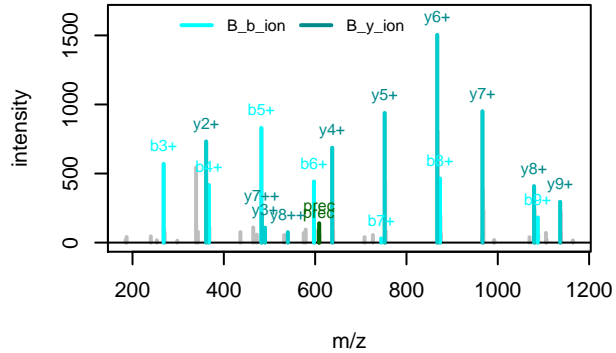

Supplement: Supplemental Data [file supp_RA117.000470_133922_0_supp_23978_fzffwf.zip › spectra_annotation/mito_DR_spectra_annotation/111-1-3-1-5-1.pdf]

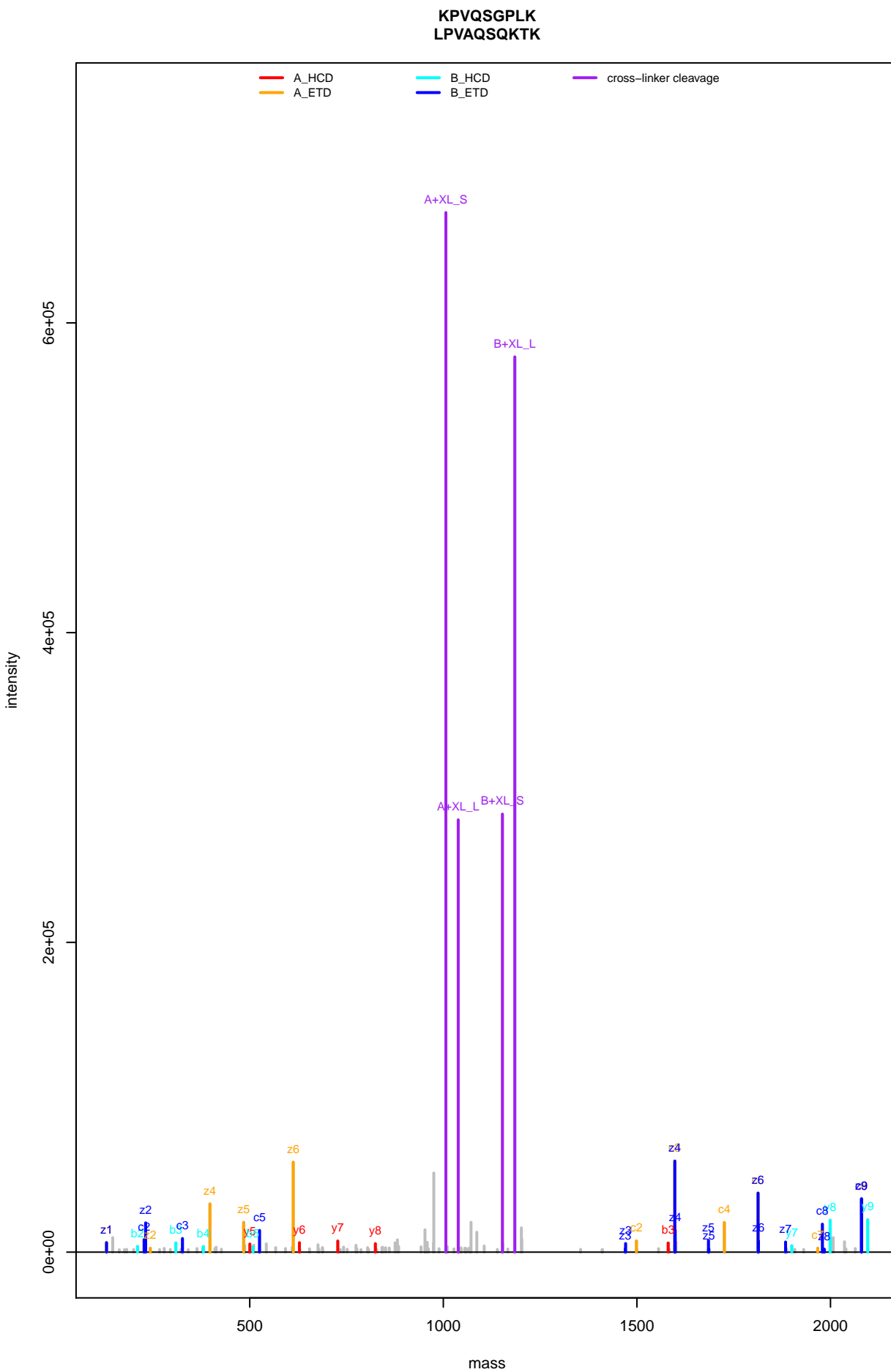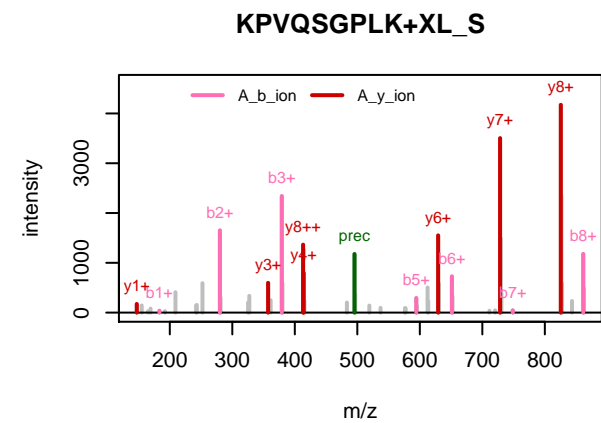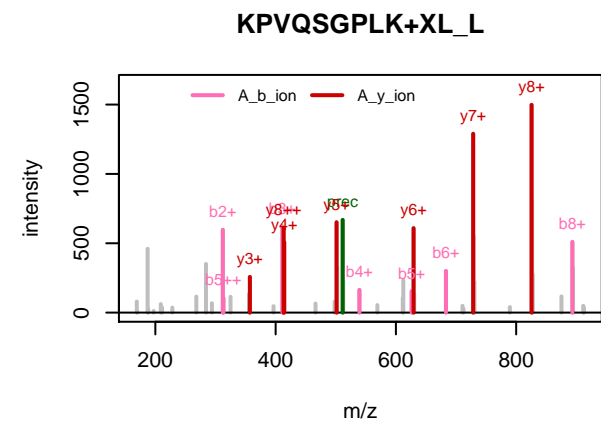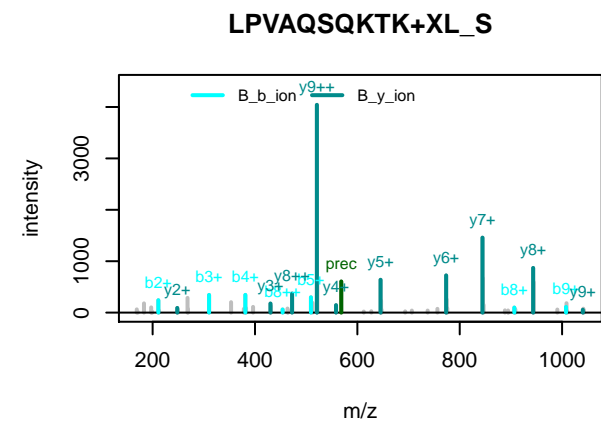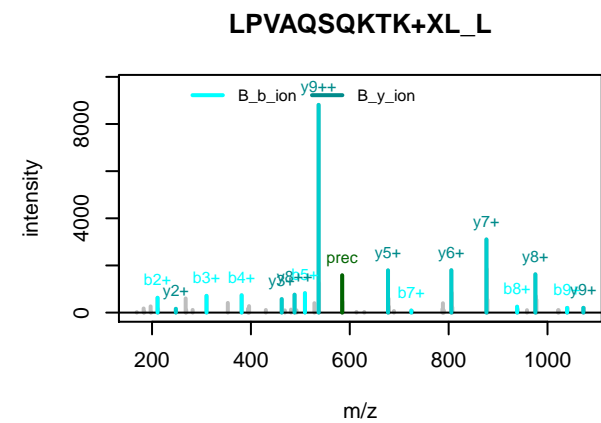

Supplement: Supplemental Data [file supp_RA117.000470_133922_0_supp_23978_fzffwf.zip › spectra_annotation/mito_DR_spectra_annotation/111-1-42-1-4-1.pdf]

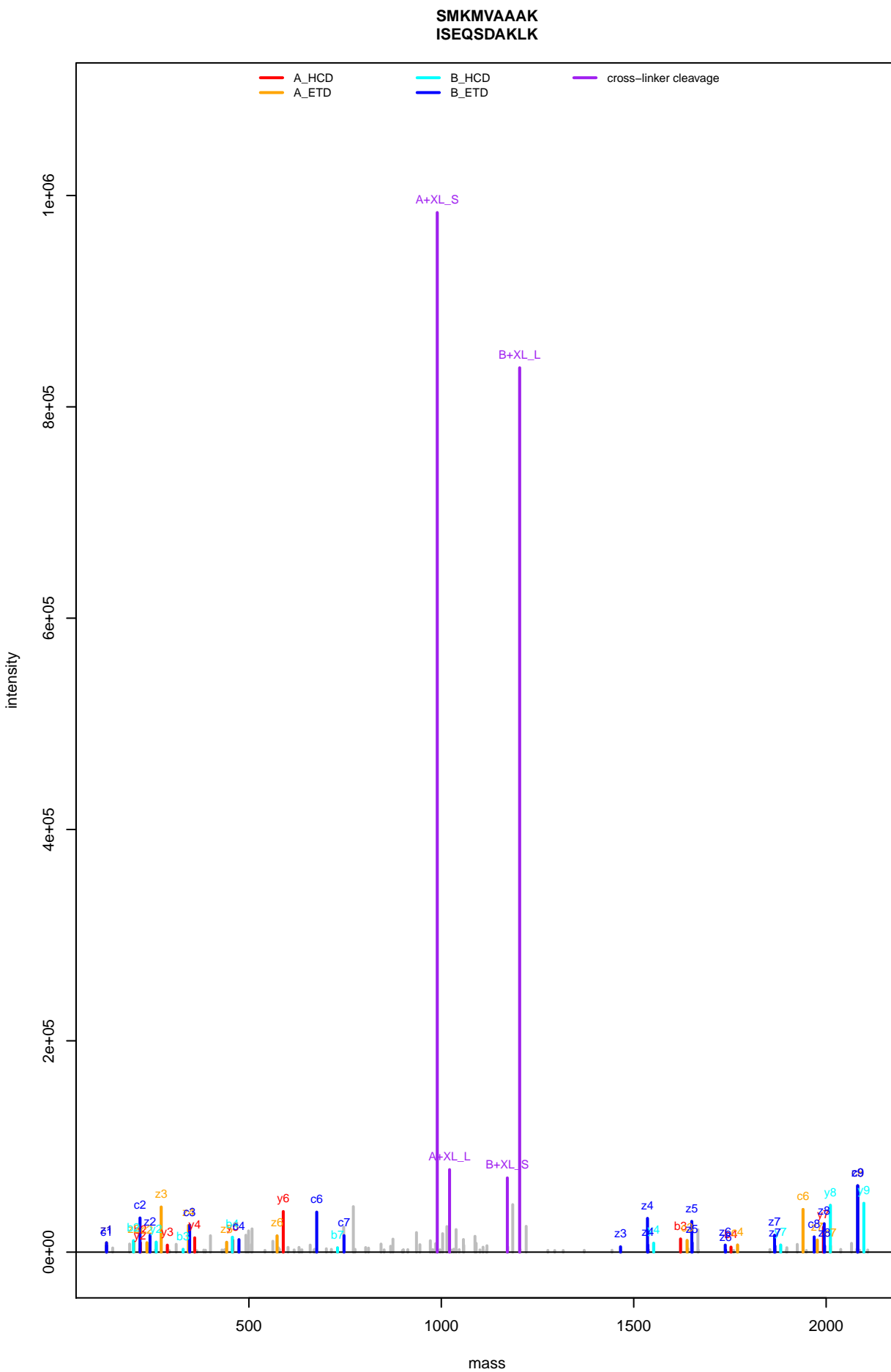

Supplement: Supplemental Data [file supp_RA117.000470_133922_0_supp_23978_fzffwf.zip › spectra_annotation/mito_DR_spectra_annotation/112-1-3-1-12-1.pdf]

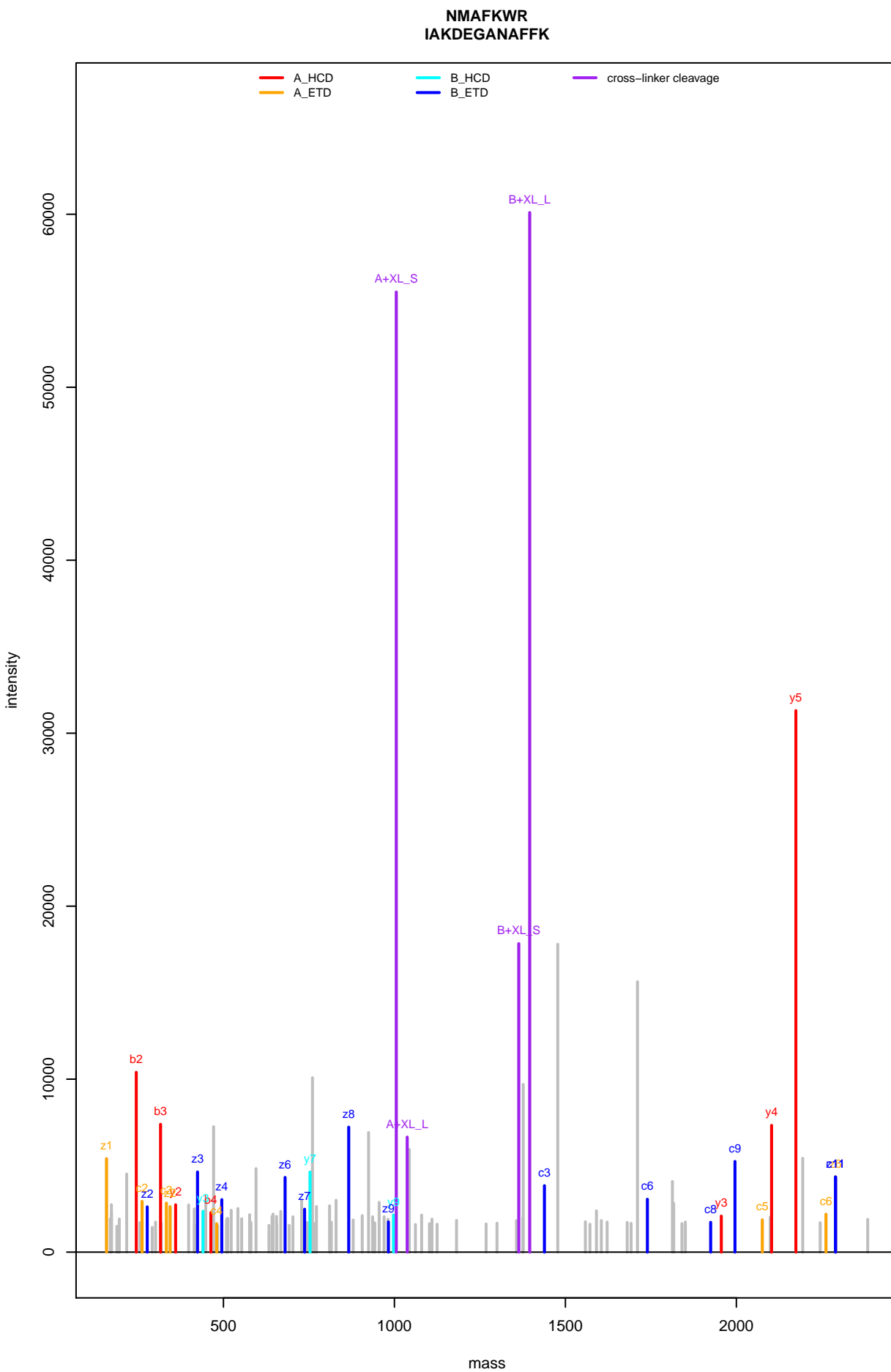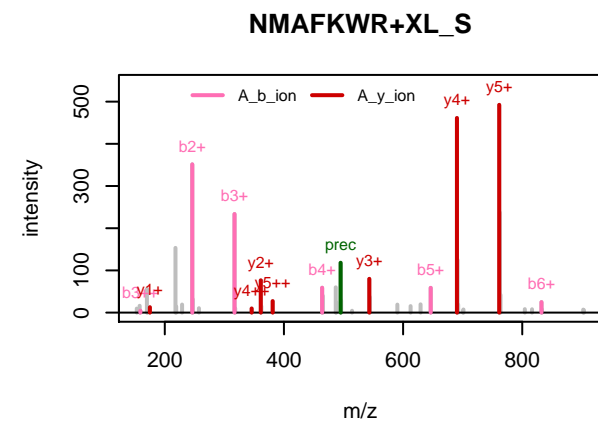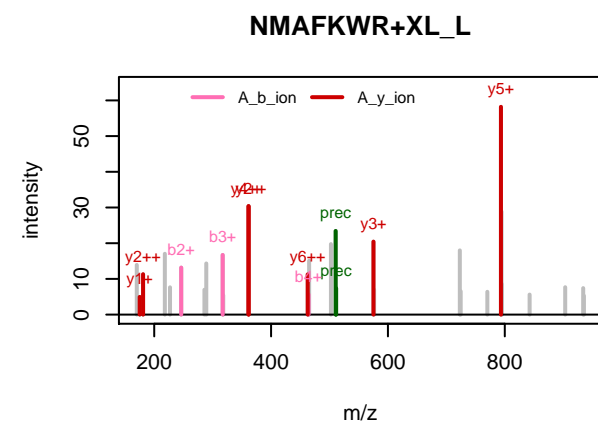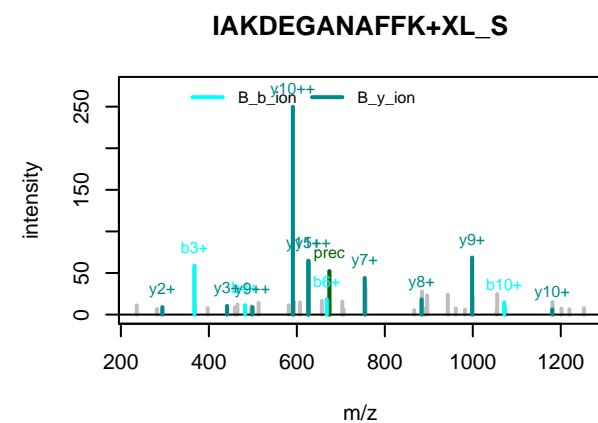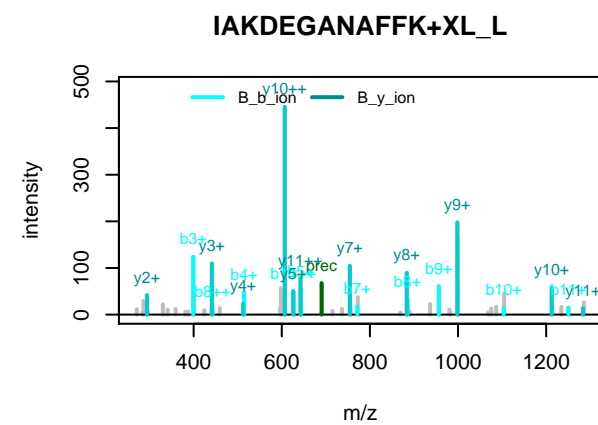

Supplement: Supplemental Data [file supp_RA117.000470_133922_0_supp_23978_fzffwf.zip › spectra_annotation/mito_DR_spectra_annotation/112-1-5-1-6-1.pdf]

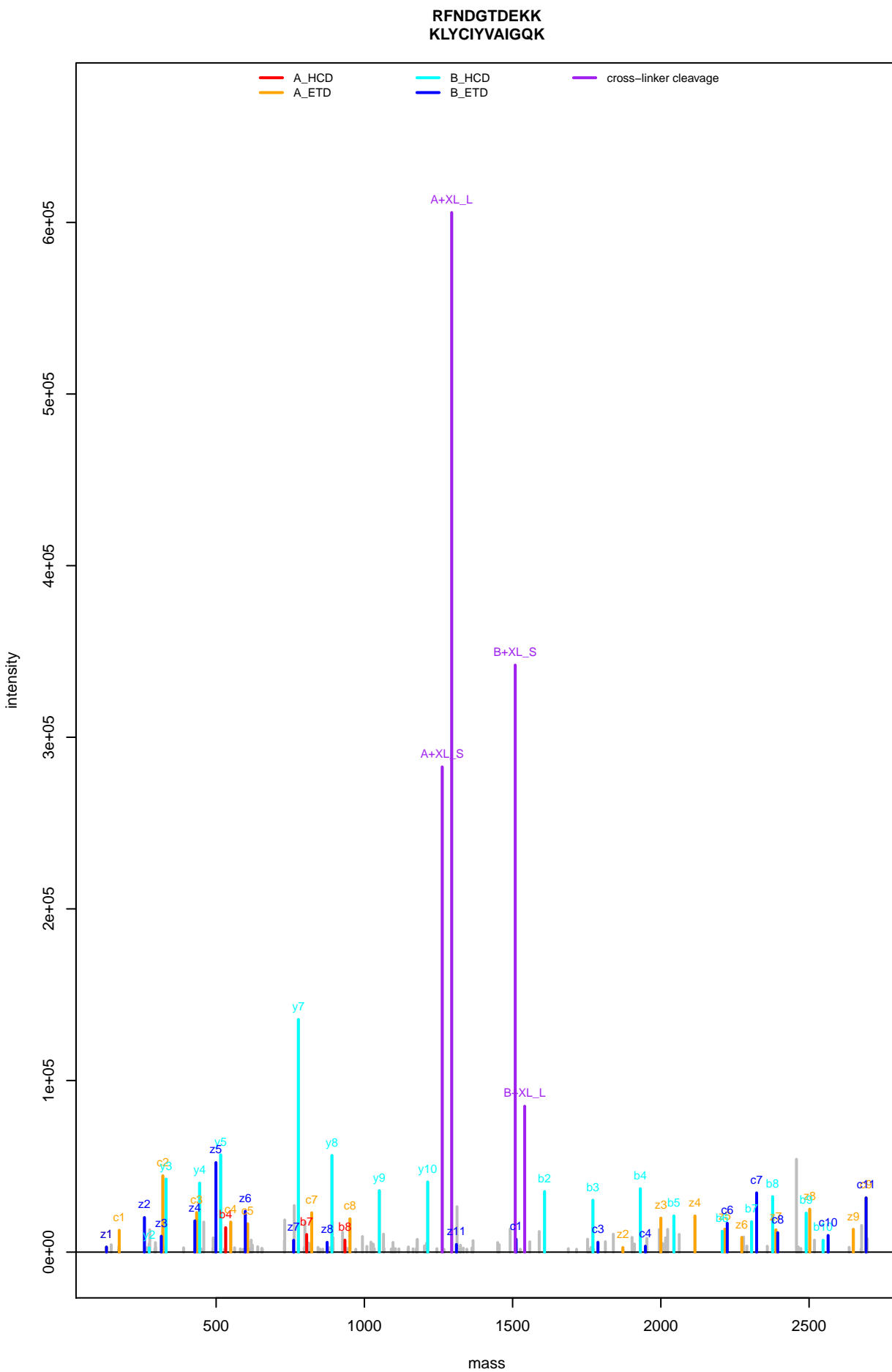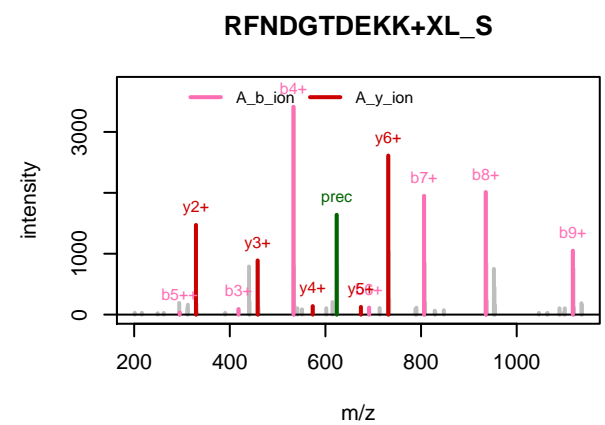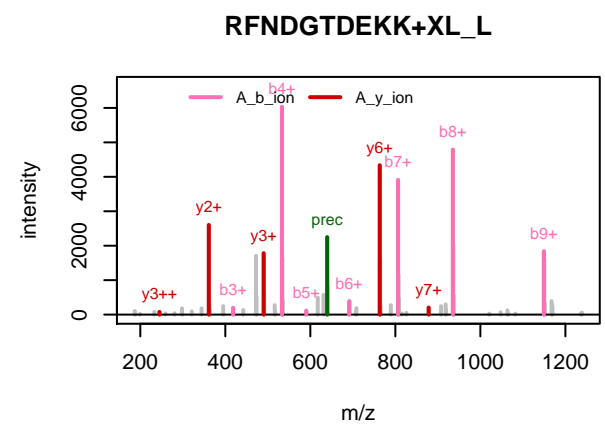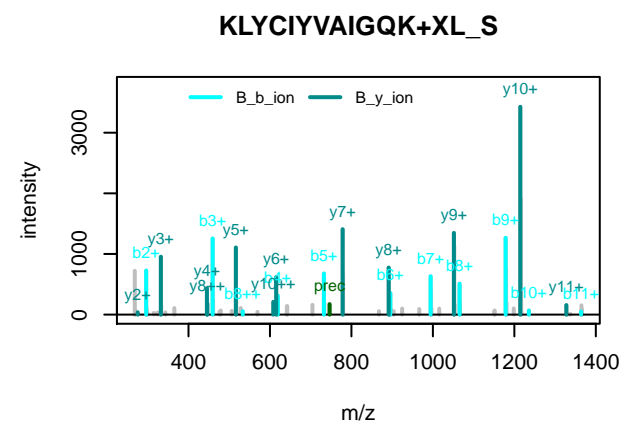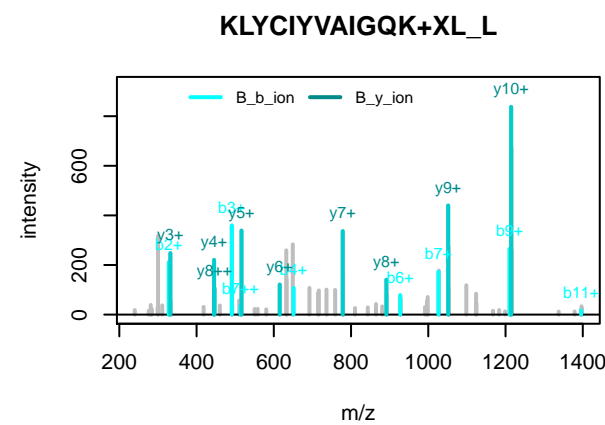

Supplement: Supplemental Data [file supp_RA117.000470_133922_0_supp_23978_fzffwf.zip › spectra_annotation/mito_DR_spectra_annotation/112-1-6-1-18-1.pdf]

EESI-KR  
EVLEDFAEDEGEKK

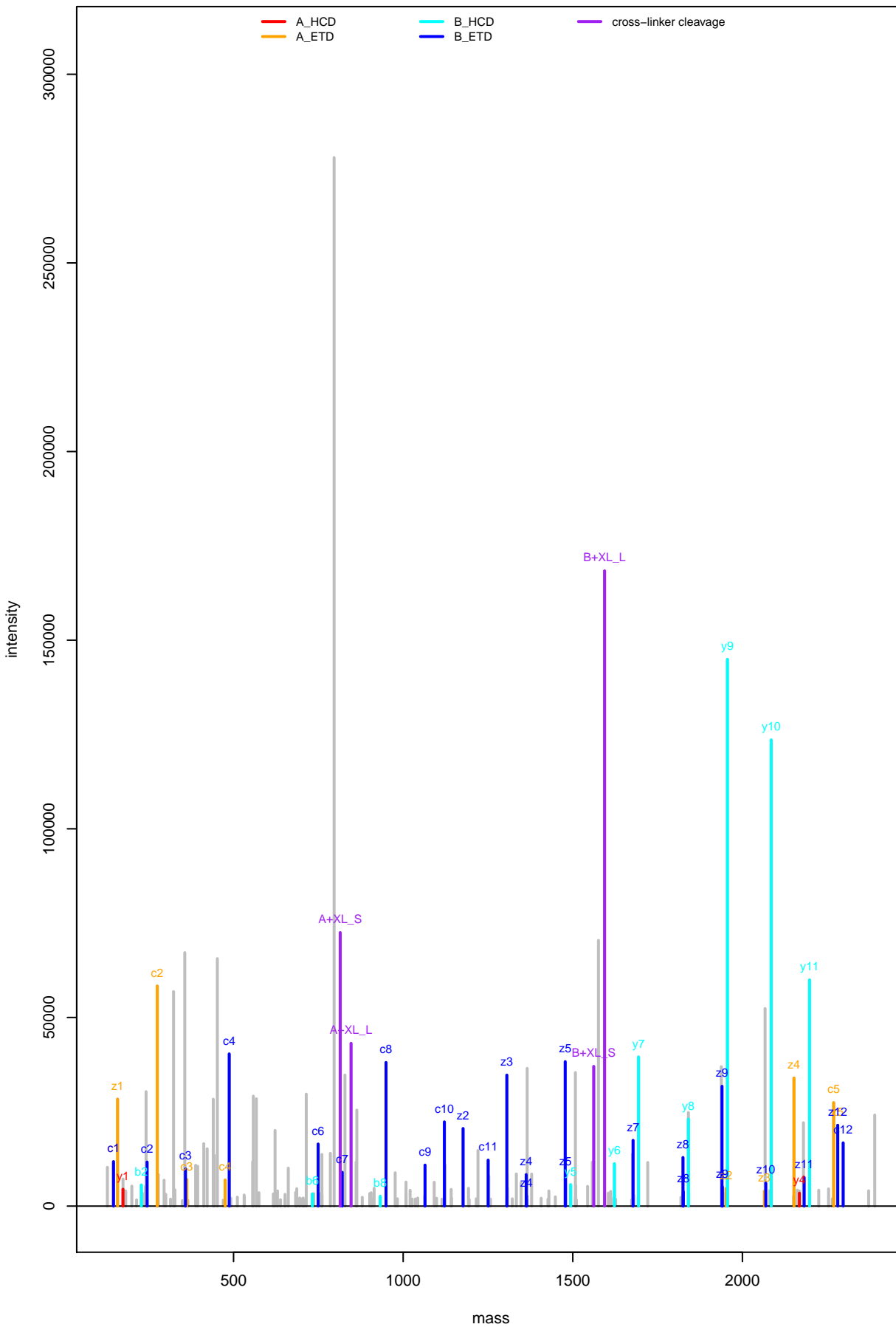

EESI-KR+XL\_S

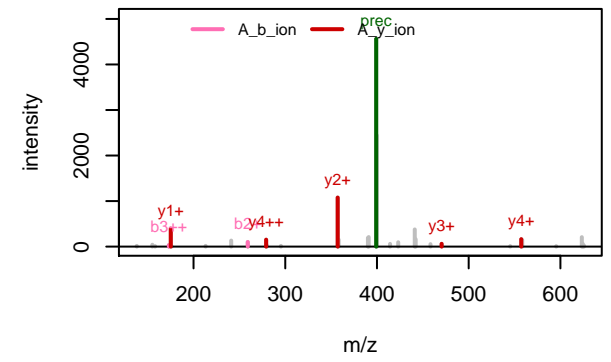

EESI-KR+XL\_L

EVLEDFAEDEGEKK+XL\_S

EVLEDFAEDEGEKK+XL\_L

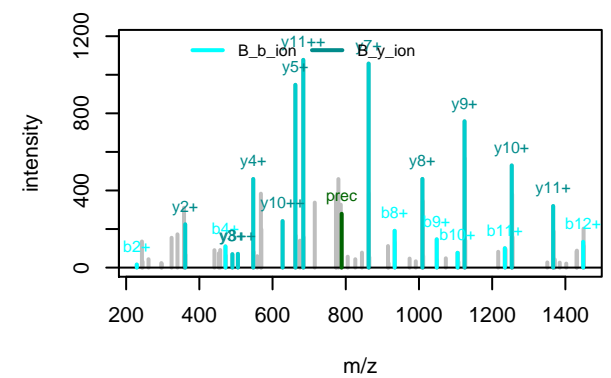

Supplement: Supplemental Data [file supp_RA117.000470_133922_0_supp_23978_fzffwf.zip › spectra_annotation/mito_DR_spectra_annotation/112-1-7-1-10-1.pdf]

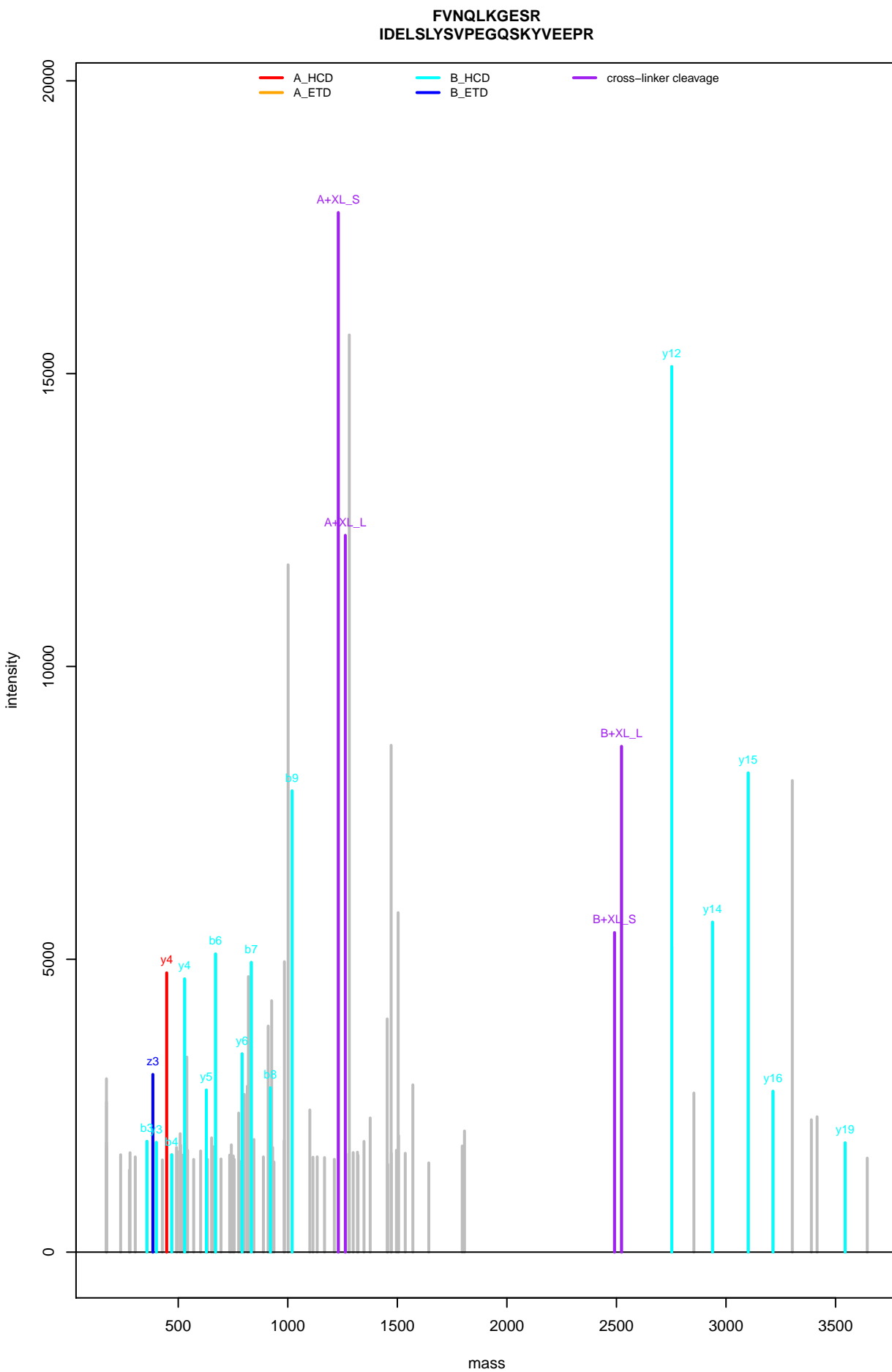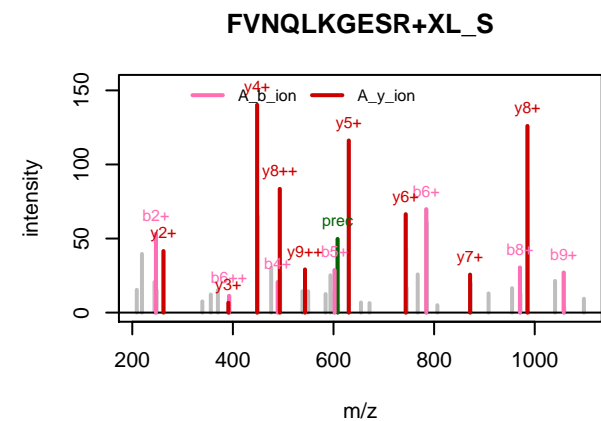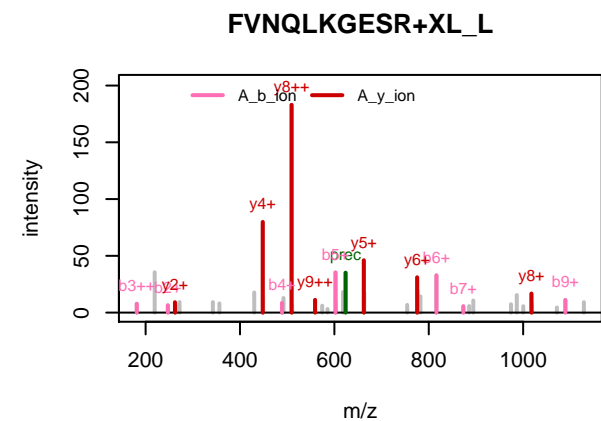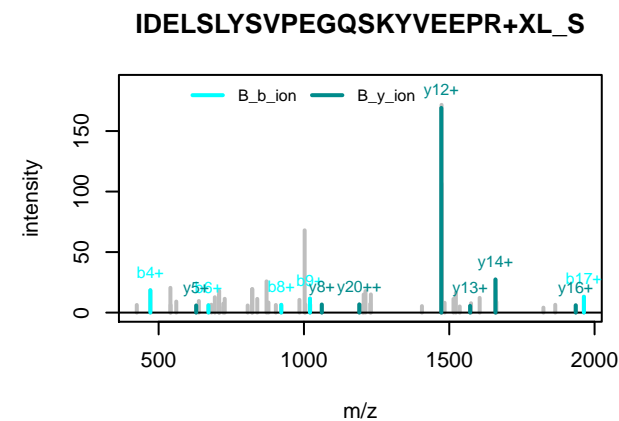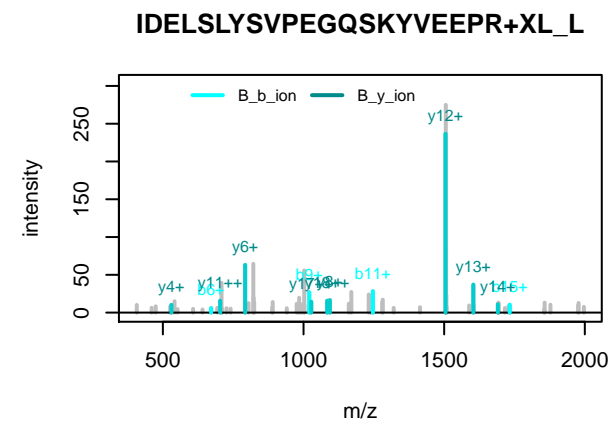

Supplement: Supplemental Data [file supp_RA117.000470_133922_0_supp_23978_fzffwf.zip › spectra_annotation/mito_DR_spectra_annotation/113-1-10-1-24-1.pdf]

ADKLAEEHGS  
GEMDTFPTFKFDDPK

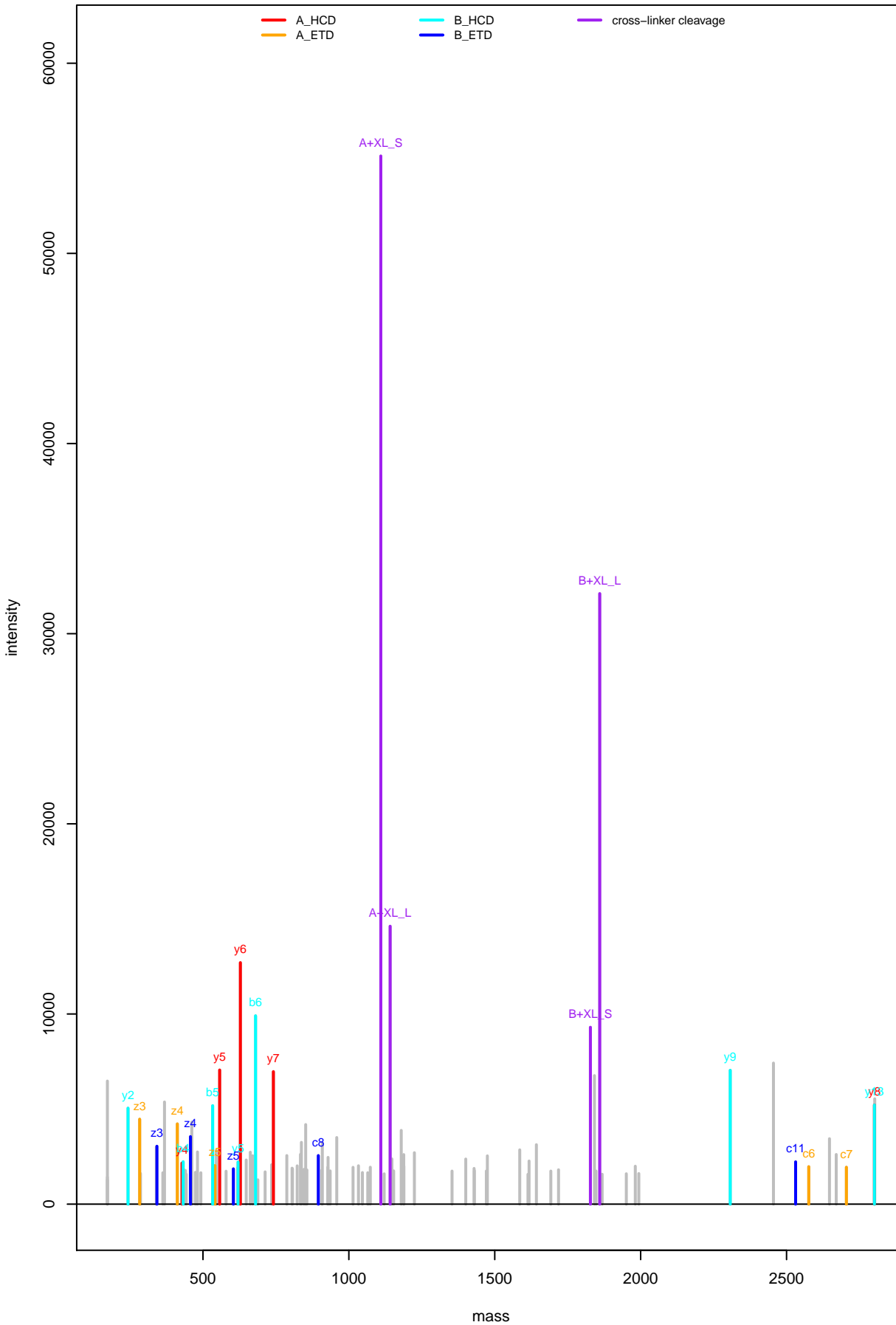

Supplement: Supplemental Data [file supp_RA117.000470_133922_0_supp_23978_fzffwf.zip › spectra_annotation/mito_DR_spectra_annotation/113-1-3-1-3-1.pdf]

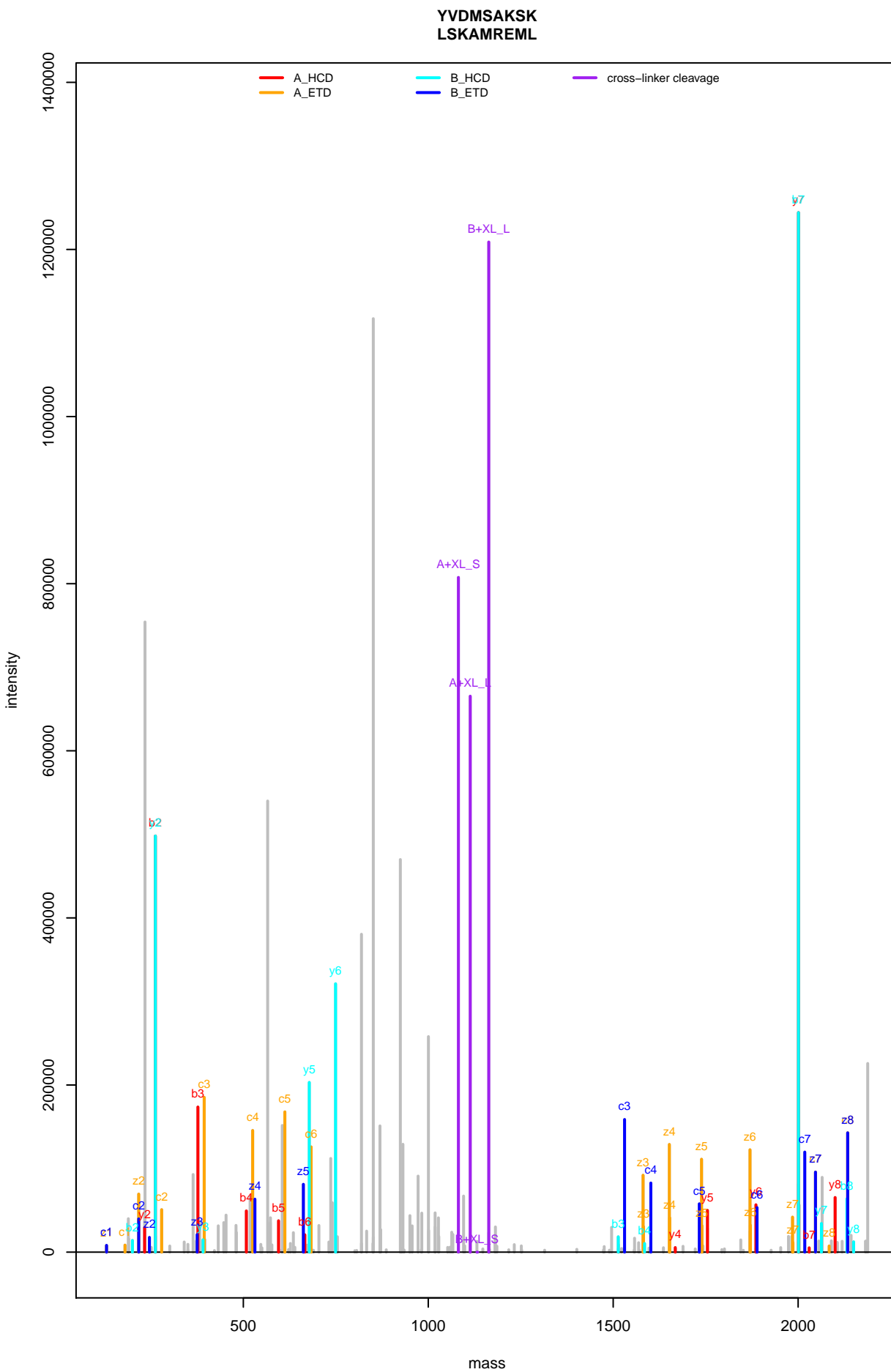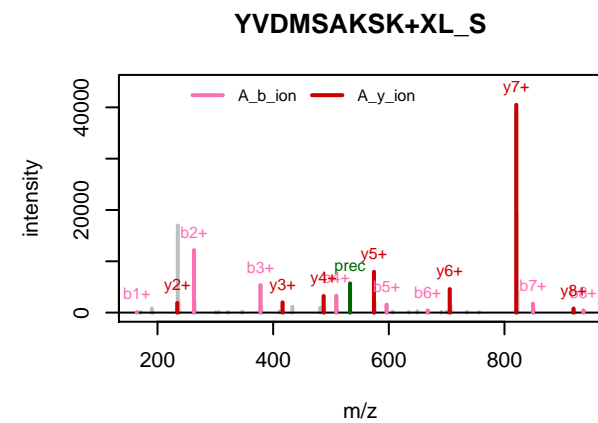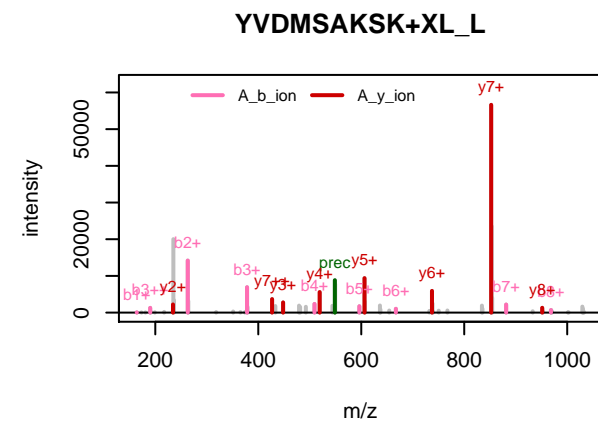

**LSKAMREML+XL\_S**

**LSKAMREML+XL\_L**

Supplement: Supplemental Data [file supp_RA117.000470_133922_0_supp_23978_fzffwf.zip › spectra_annotation/mito_DR_spectra_annotation/113-1-4-1-2-1.pdf]

**ITTTKK  
SISVQKEKETIAK**

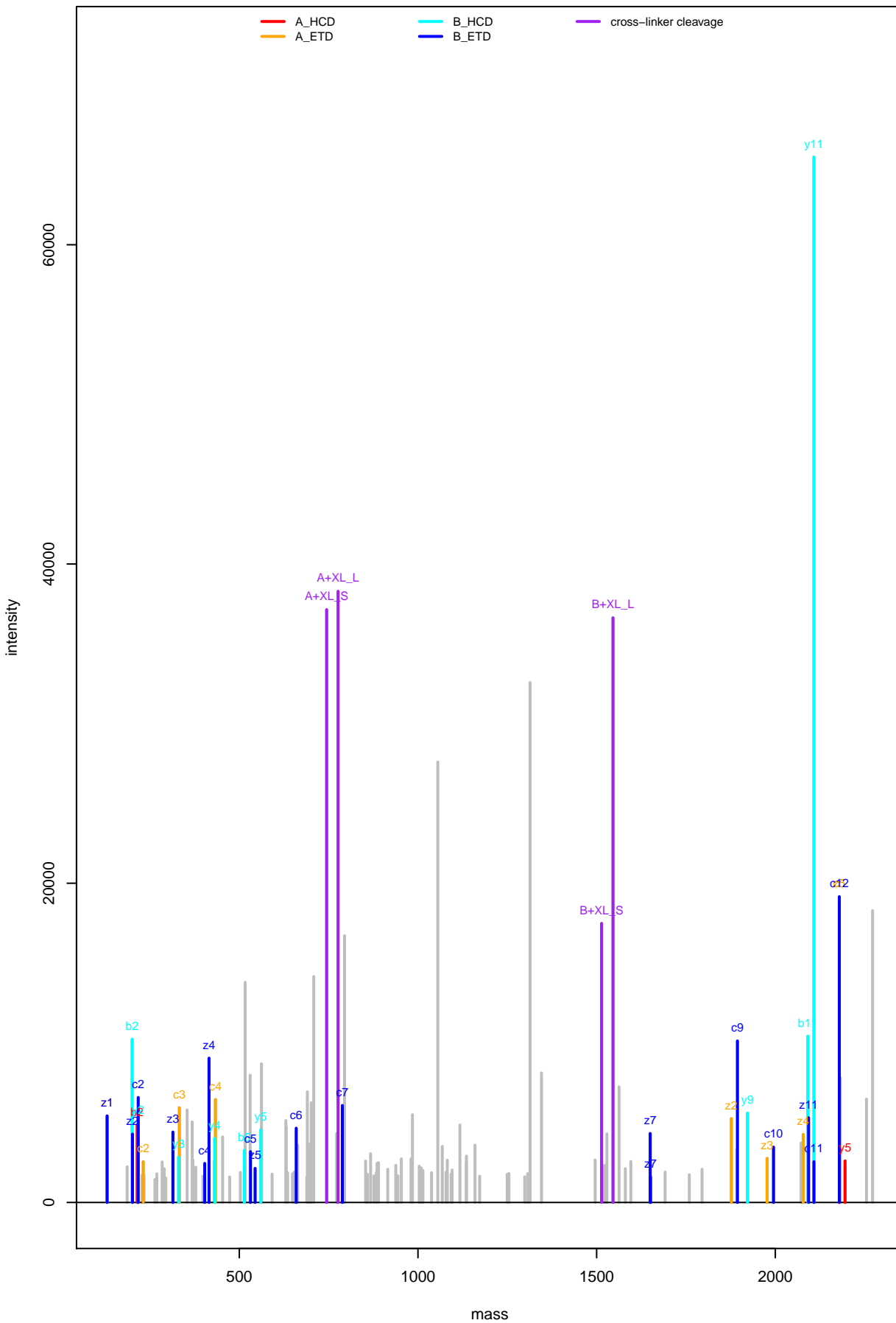

**ITTTKK+XL\_S**

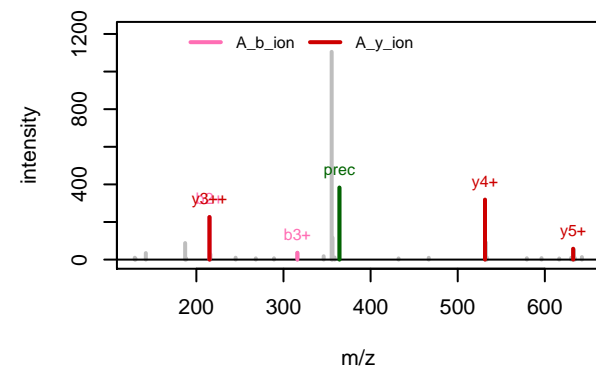

**ITTTKK+XL\_L**

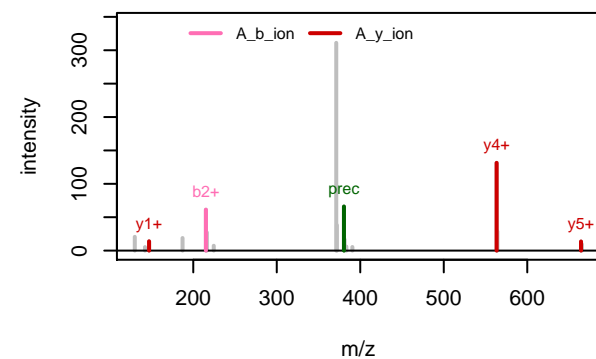

**SISVQKEKETIAK+XL\_S**

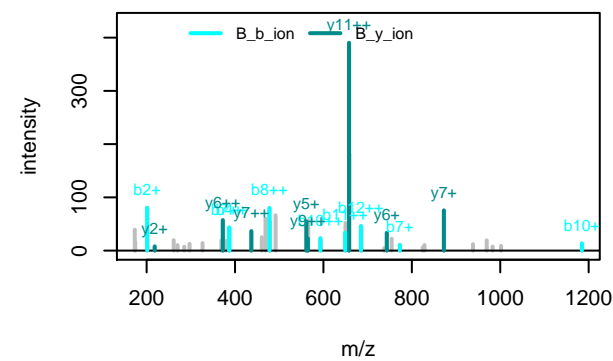

**SISVQKEKETIAK+XL\_L**

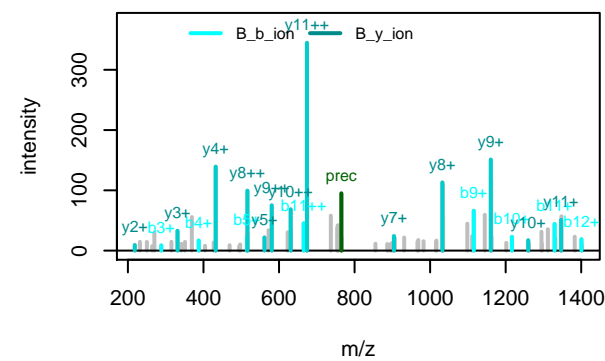

Supplement: Supplemental Data [file supp_RA117.000470_133922_0_supp_23978_fzffwf.zip › spectra_annotation/mito_DR_spectra_annotation/114-1-1-1-11-1.pdf]

MKTIIEDAK  
TAMDNSEIAGEKK

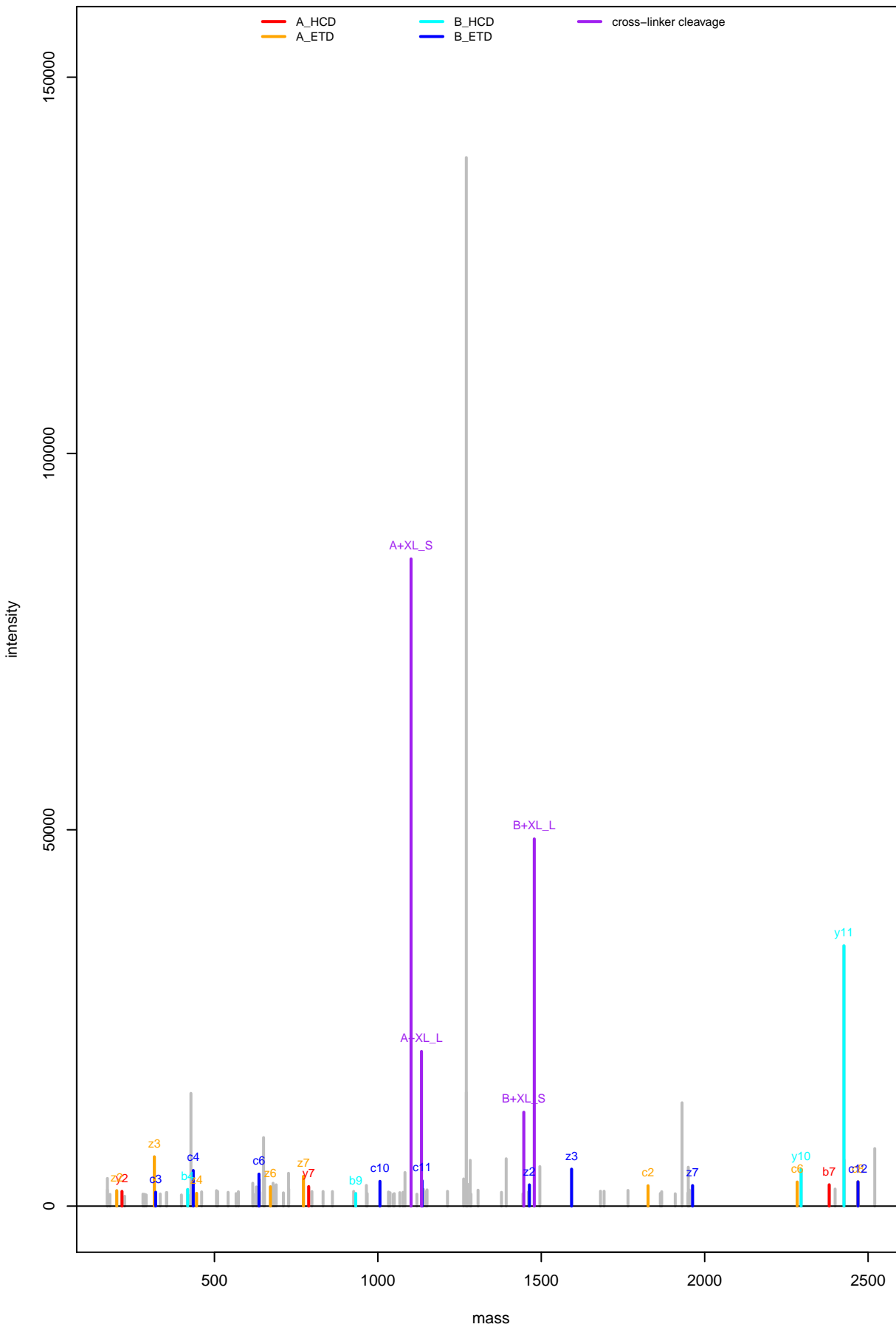

MKTIIEDAK+XL\_S

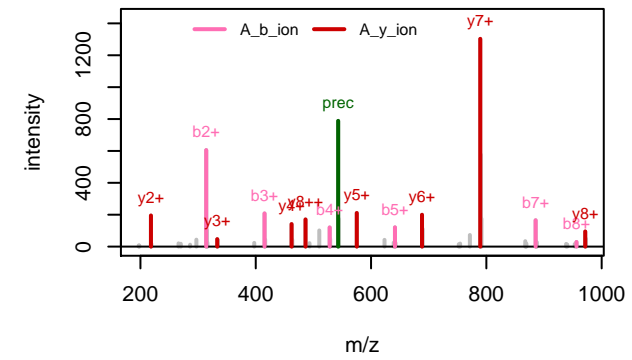

MKTIIEDAK+XL\_L

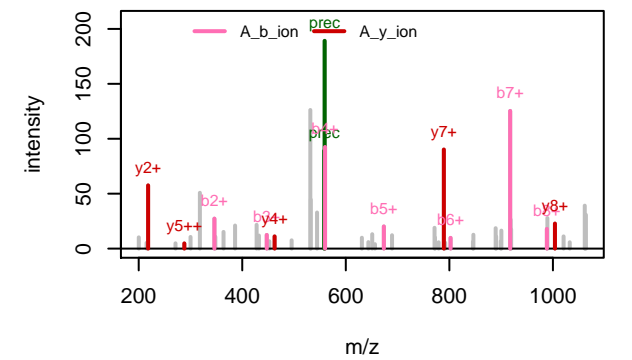

TAMDNSEIAGEKK+XL\_S

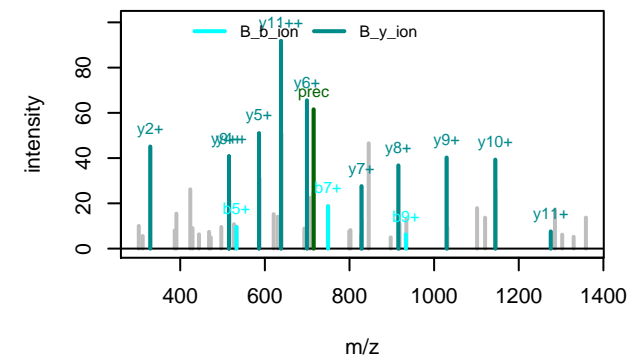

TAMDNSEIAGEKK+XL\_L

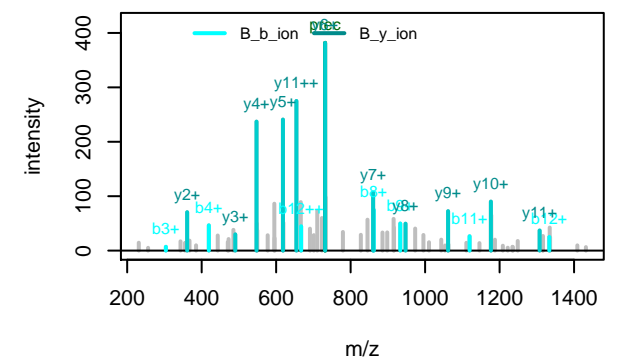

Supplement: Supplemental Data [file supp_RA117.000470_133922_0_supp_23978_fzffwf.zip › spectra_annotation/mito_DR_spectra_annotation/114-1-10-1-3-1.pdf]

LEPSKITK  
QMKNEVAALTAAGK

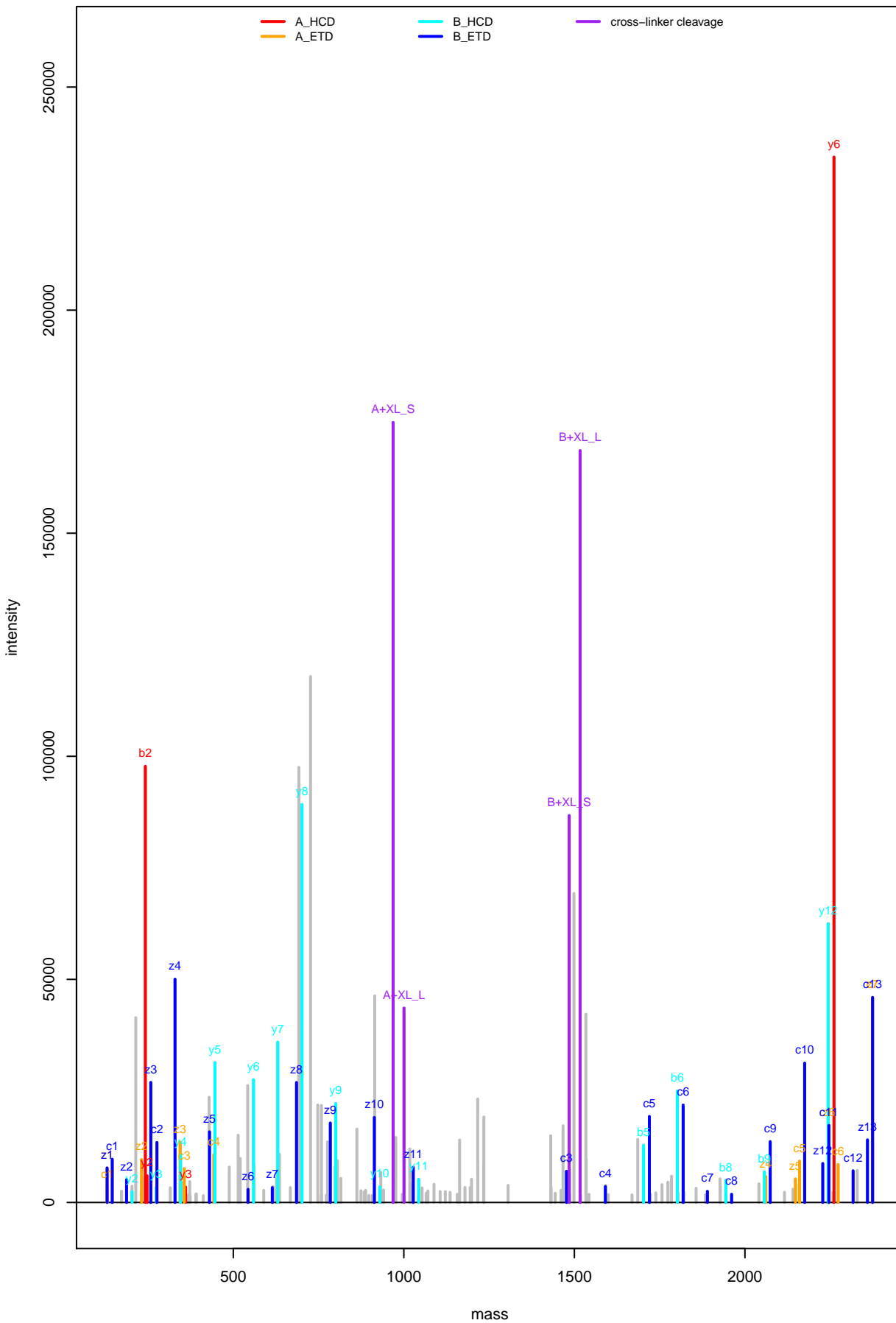

LEPSKITK+XL\_S

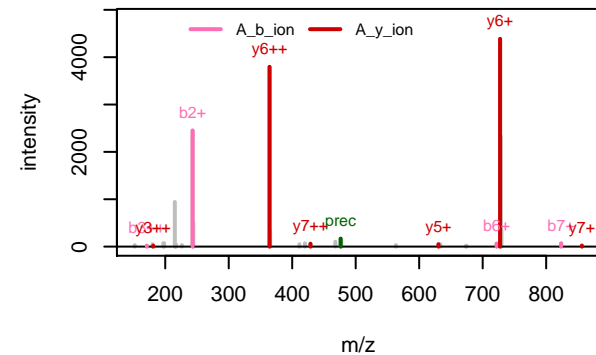

LEPSKITK+XL\_L

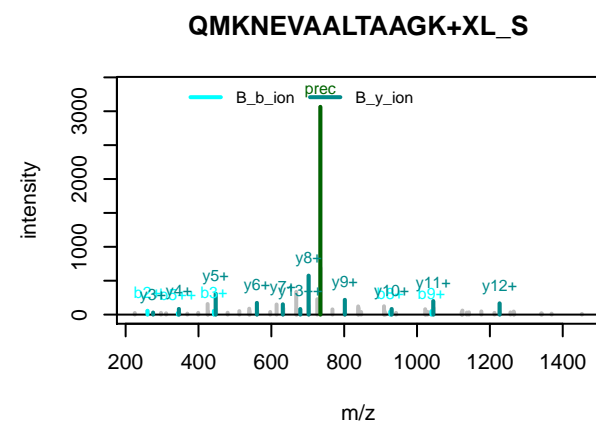

QMKNEVAALTAAGK+XL\_L

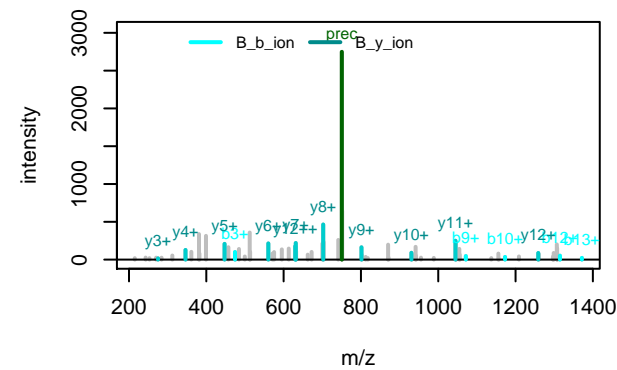

Supplement: Supplemental Data [file supp_RA117.000470_133922_0_supp_23978_fzffwf.zip › spectra_annotation/mito_DR_spectra_annotation/114-1-19-1-5-1.pdf]

LEPSKITK  
TSGSSIKIVK

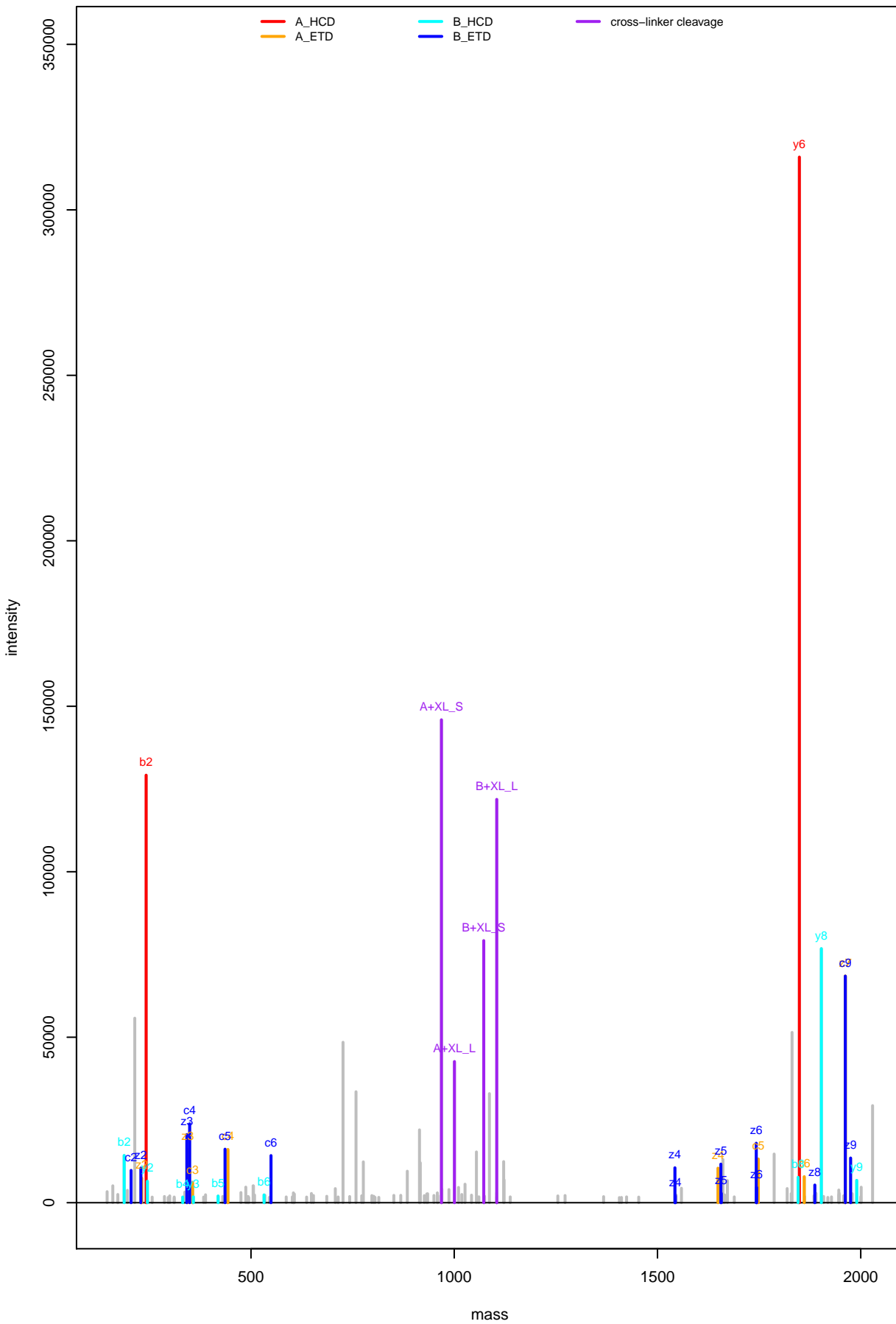

LEPSKITK+XL\_S

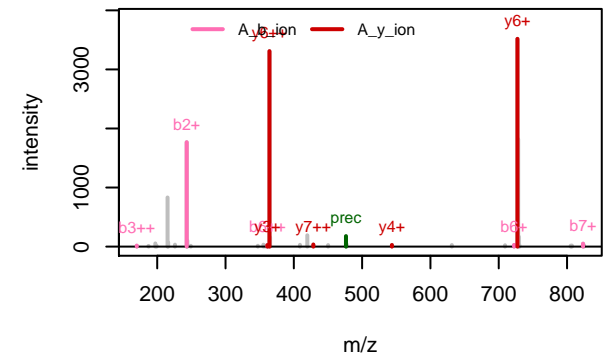

LEPSKITK+XL\_L

TSGSSIKIVK+XL\_S

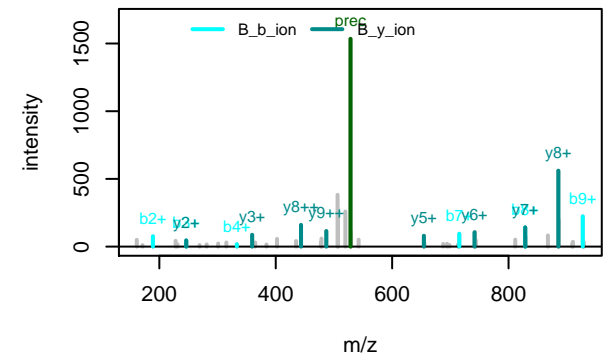

TSGSSIKIVK+XL\_L

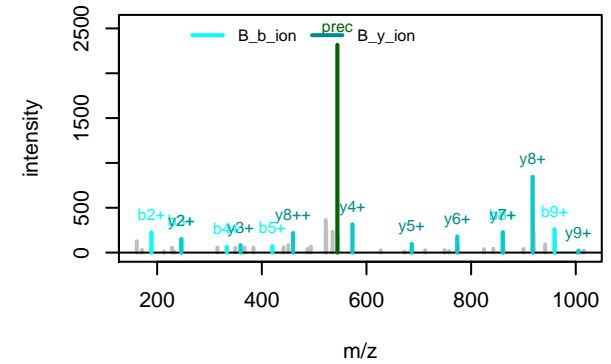

Supplement: Supplemental Data [file supp_RA117.000470_133922_0_supp_23978_fzffwf.zip › spectra_annotation/mito_DR_spectra_annotation/114-1-19-1-7-1.pdf]

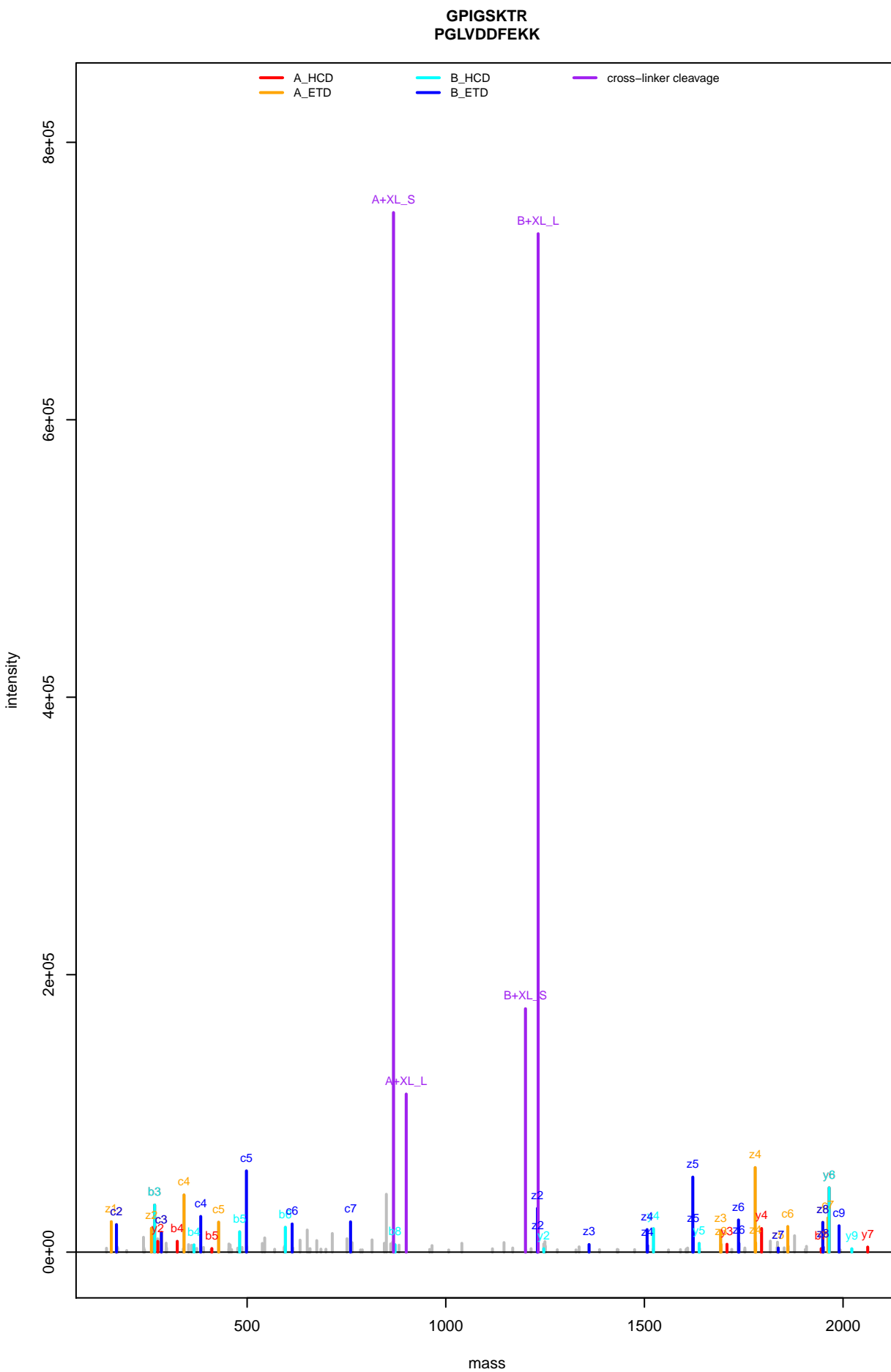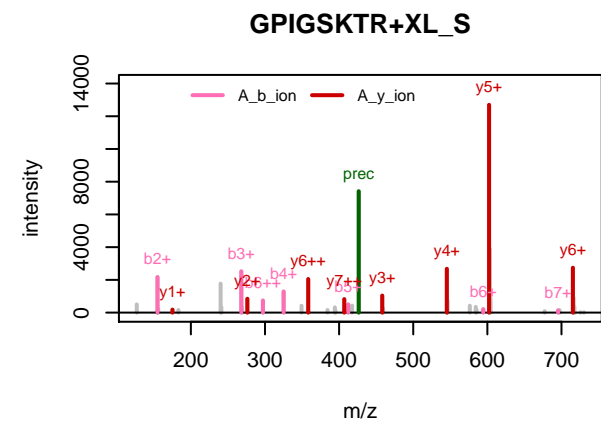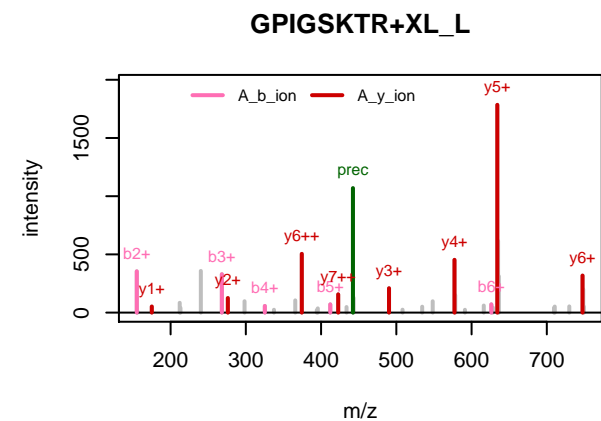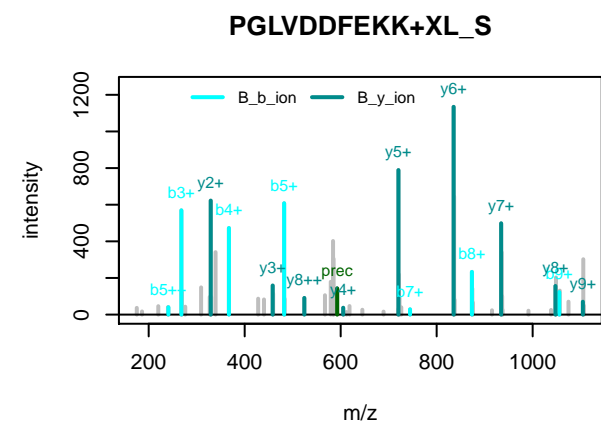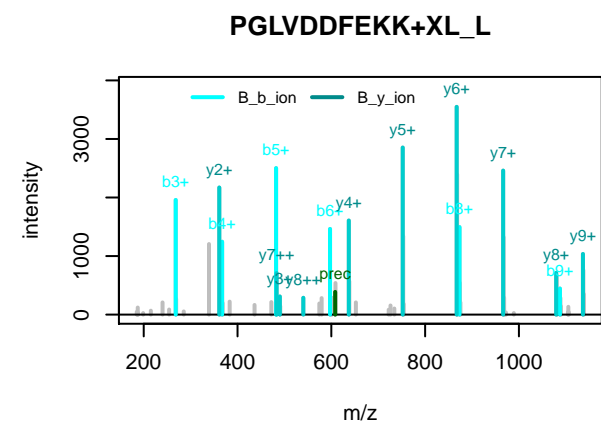

Supplement: Supplemental Data [file supp_RA117.000470_133922_0_supp_23978_fzffwf.zip › spectra_annotation/mito_DR_spectra_annotation/115-1-10-1-5-1.pdf]

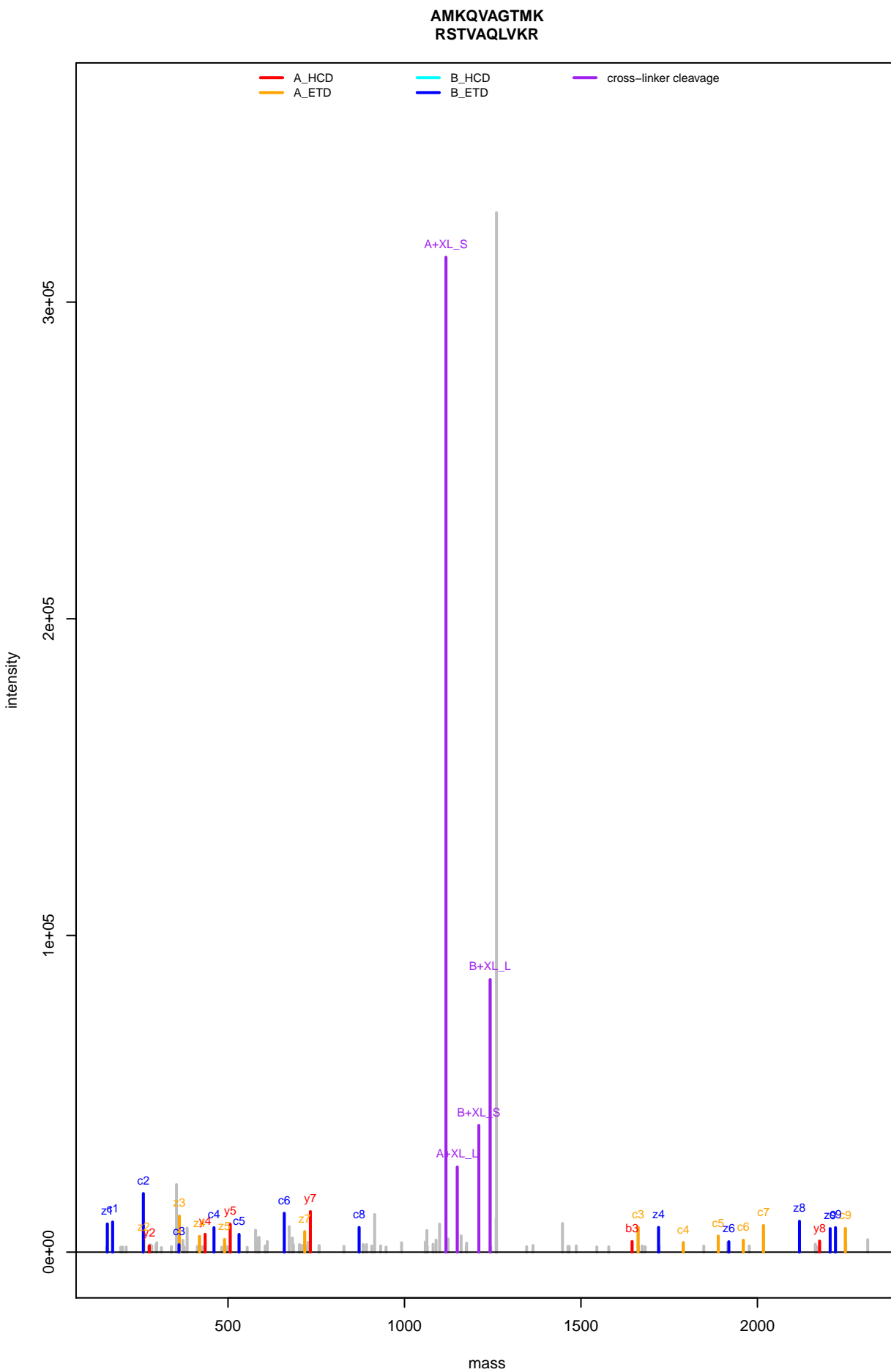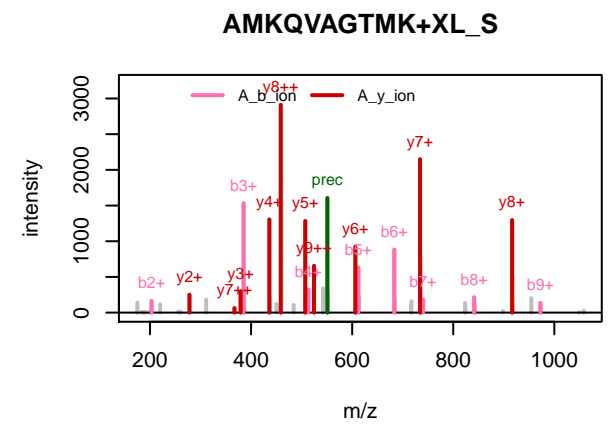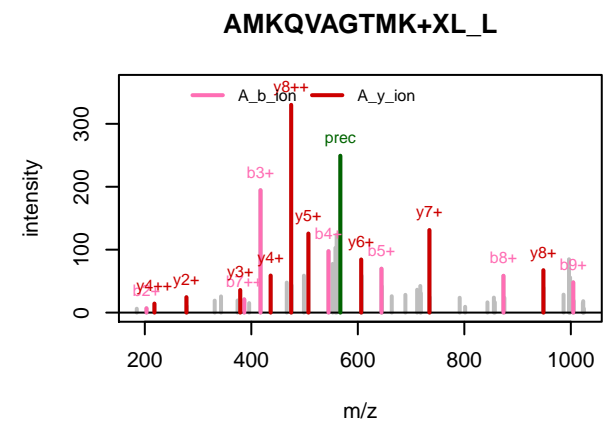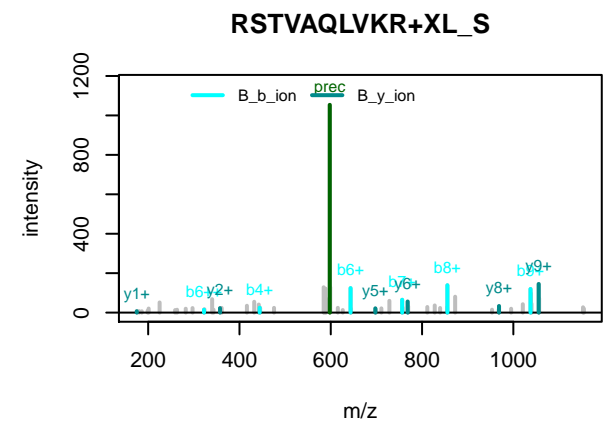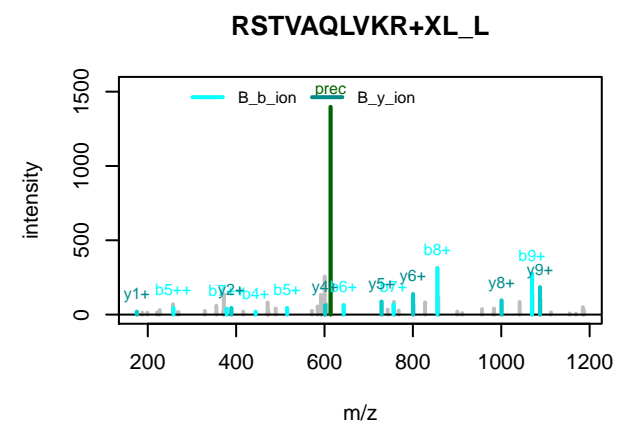

Supplement: Supplemental Data [file supp_RA117.000470_133922_0_supp_23978_fzffwf.zip › spectra_annotation/mito_DR_spectra_annotation/116-1-10-1-7-1.pdf]

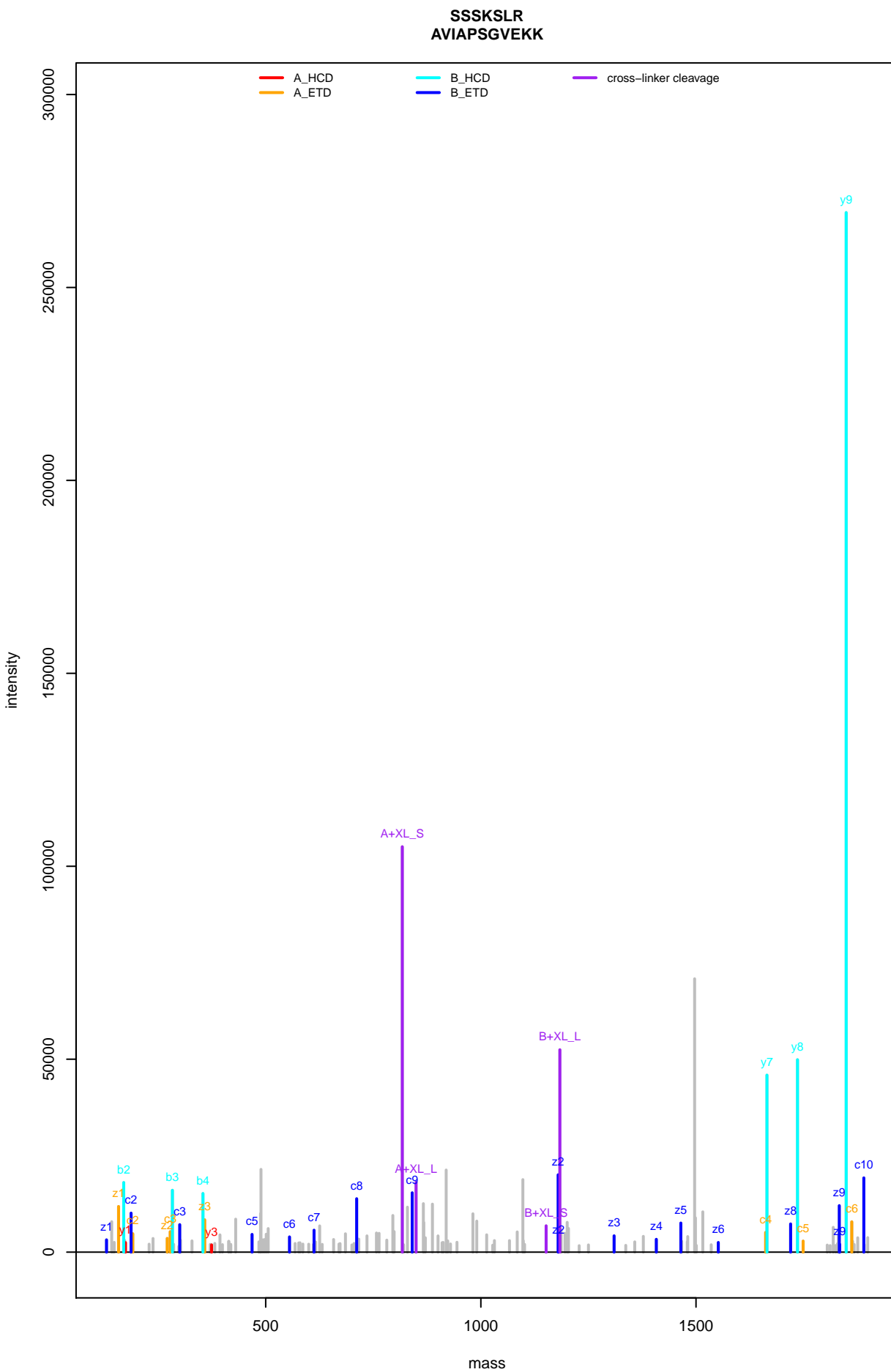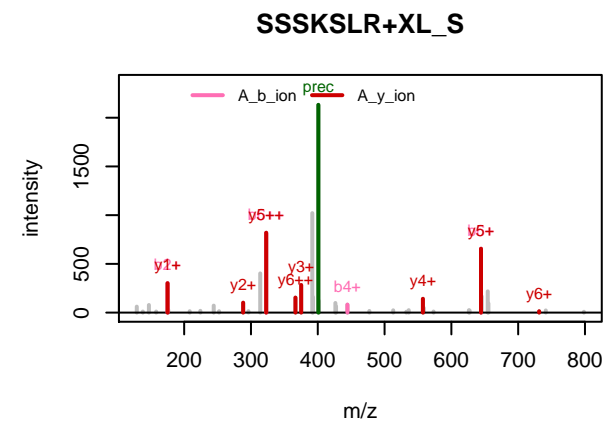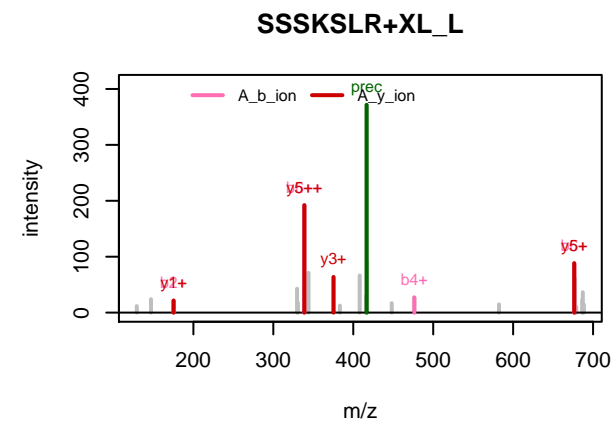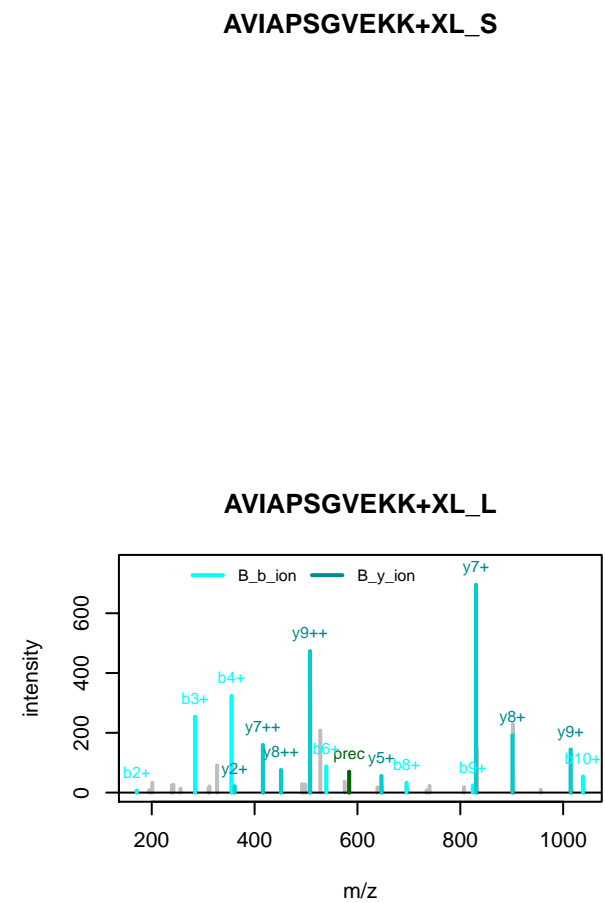

Supplement: Supplemental Data [file supp_RA117.000470_133922_0_supp_23978_fzffwf.zip › spectra_annotation/mito_DR_spectra_annotation/116-1-6-1-7-1.pdf]

# AERELKPAR GYLDKLEPSK

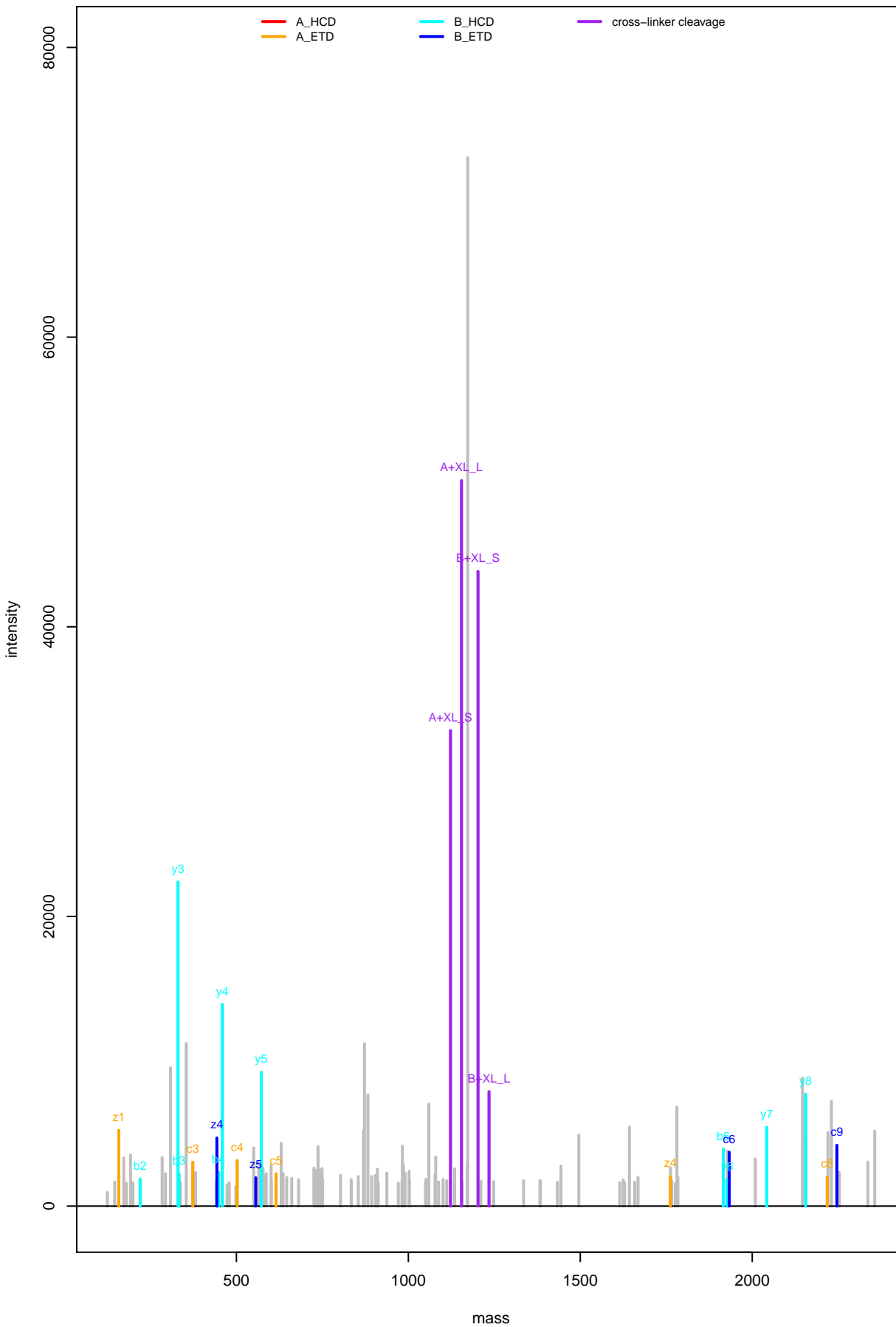

## AERELKPAR+XL\_S

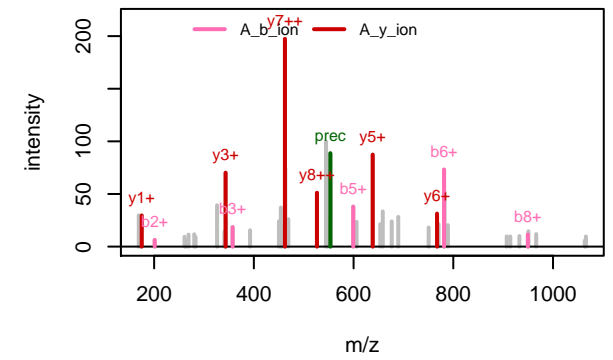

## AERELKPAR+XL\_L

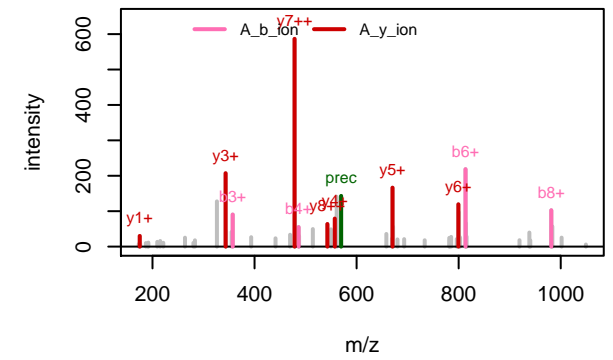

## GYLDKLEPSK+XL\_S

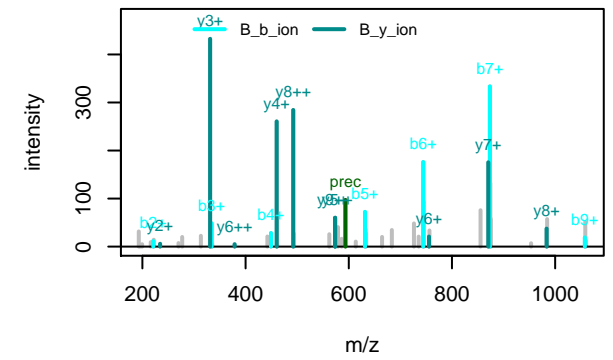

## GYLDKLEPSK+XL\_L

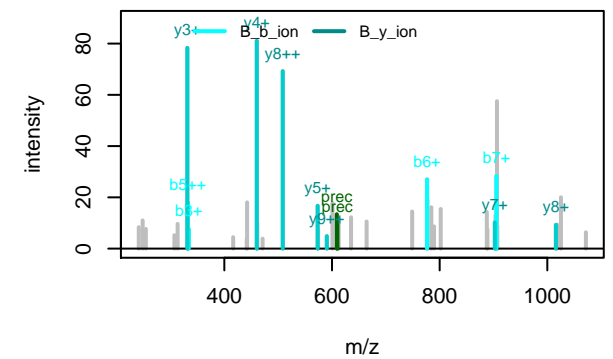

Supplement: Supplemental Data [file supp_RA117.000470_133922_0_supp_23978_fzffwf.zip › spectra_annotation/mito_DR_spectra_annotation/116-1-7-1-25-1.pdf]

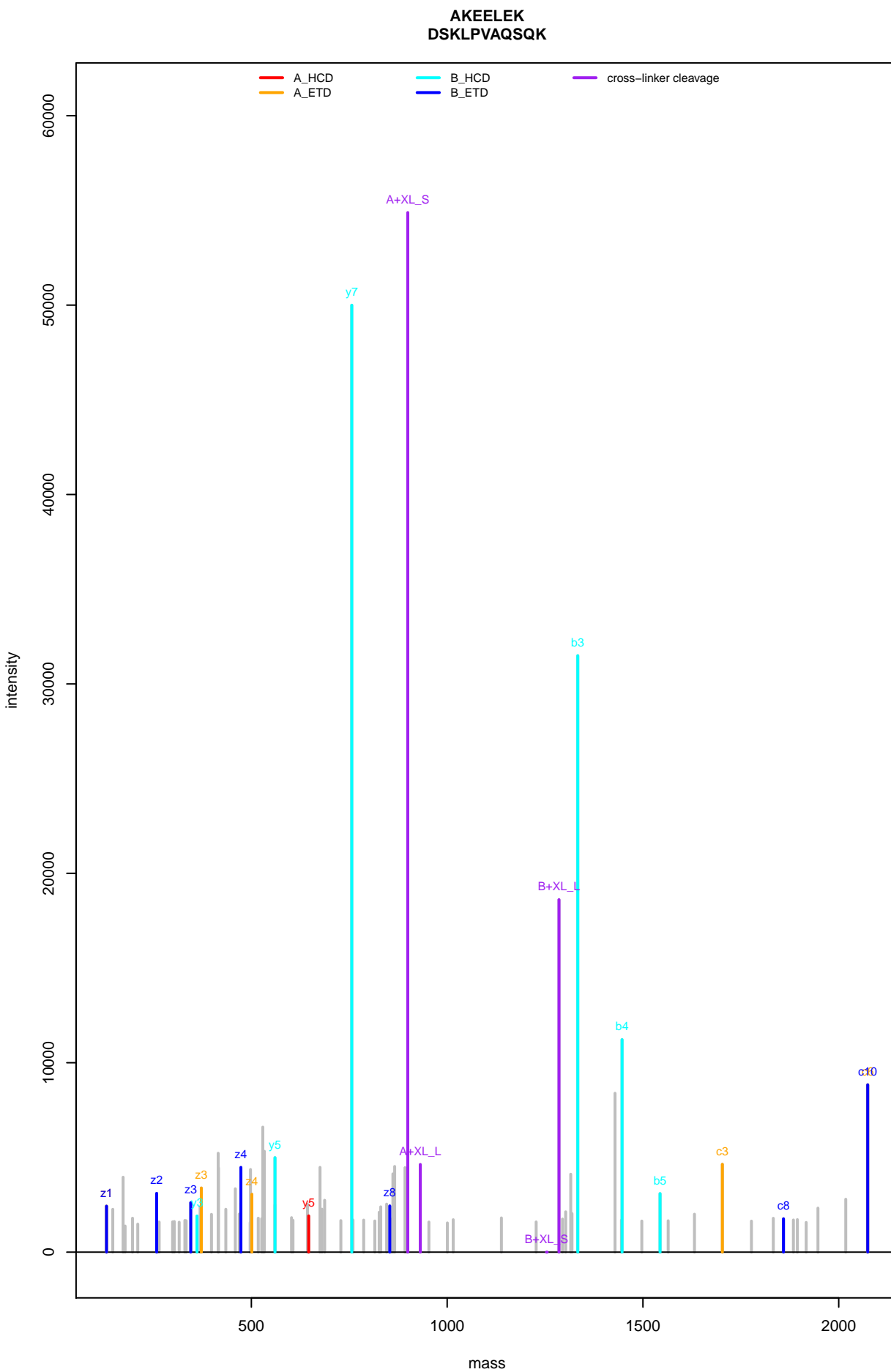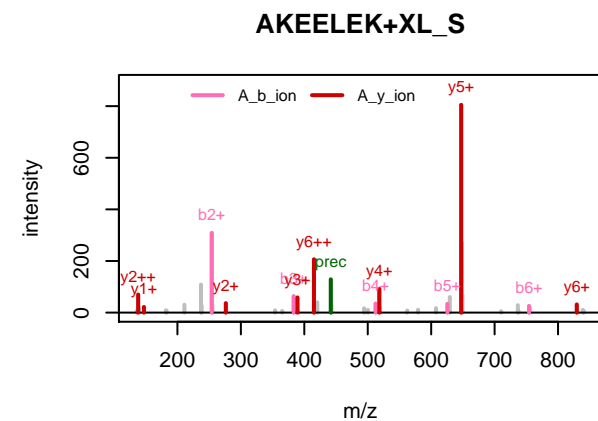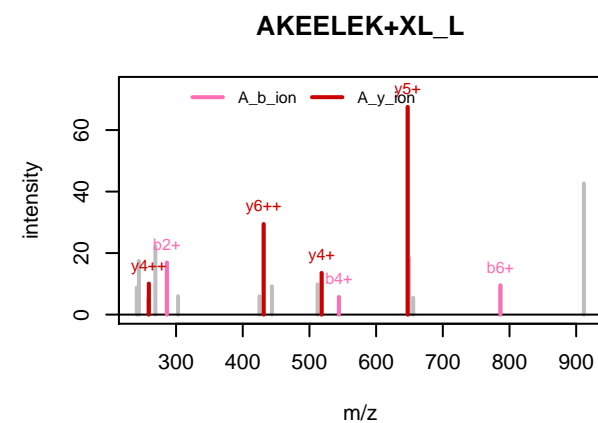

**DSKLPVAQSQK+XL\_S**

**DSKLPVAQSQK+XL\_L**

Supplement: Supplemental Data [file supp_RA117.000470_133922_0_supp_23978_fzffwf.zip › spectra_annotation/mito_DR_spectra_annotation/117-1-3-1-5-1.pdf]

LVPLKETIK  
GTKNRPGNALEK

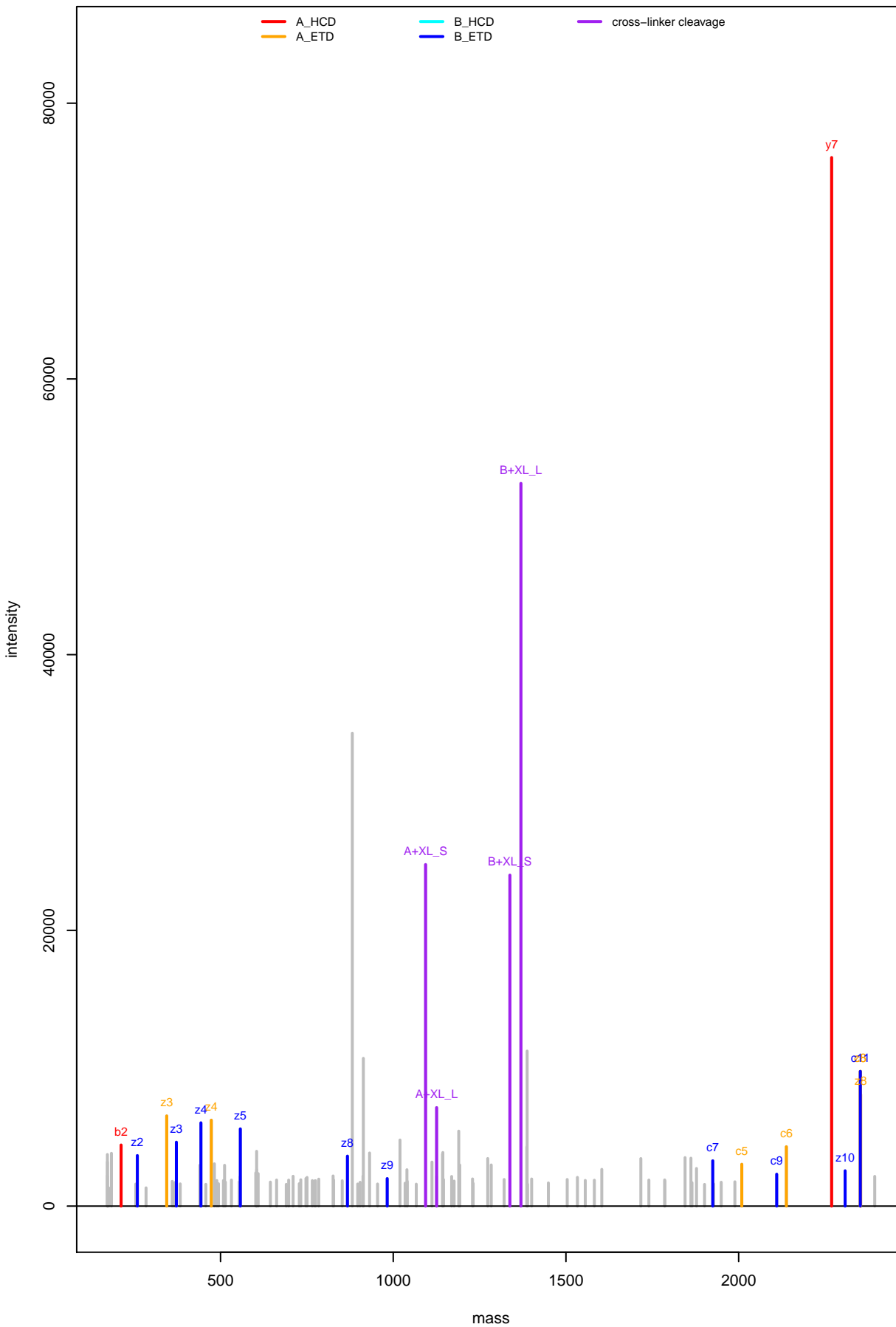

LVPLKETIK+XL\_S

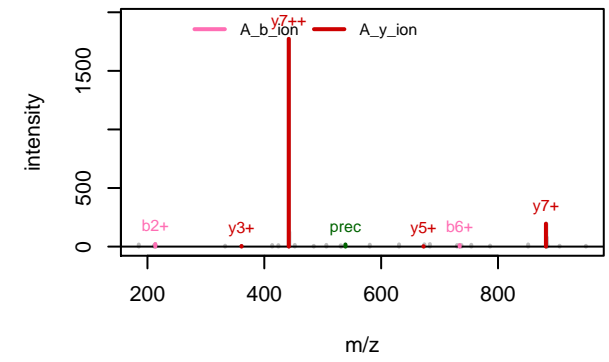

LVPLKETIK+XL\_L

GTKNRPGNALEK+XL\_S

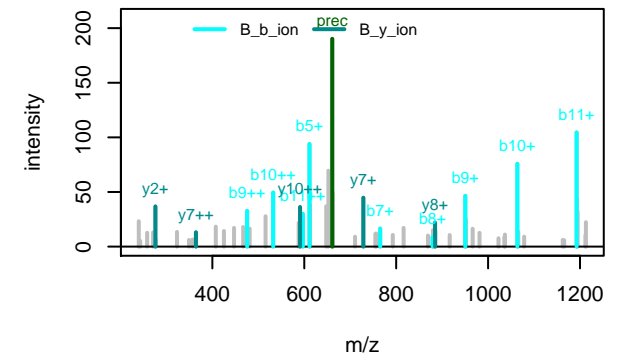

GTKNRPGNALEK+XL\_L

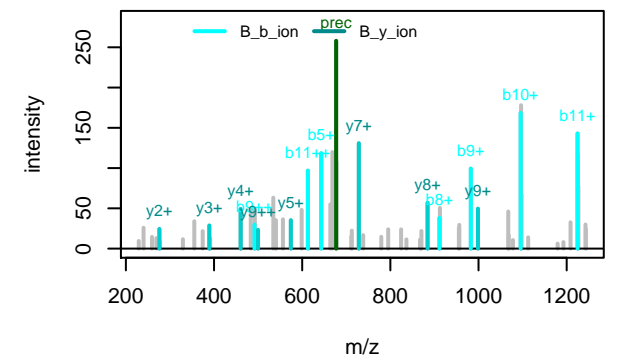

Supplement: Supplemental Data [file supp_RA117.000470_133922_0_supp_23978_fzffwf.zip › spectra_annotation/mito_DR_spectra_annotation/117-1-6-1-10-1.pdf]

**AKEELEK**  
**YSSVYGASVSEDLKR**

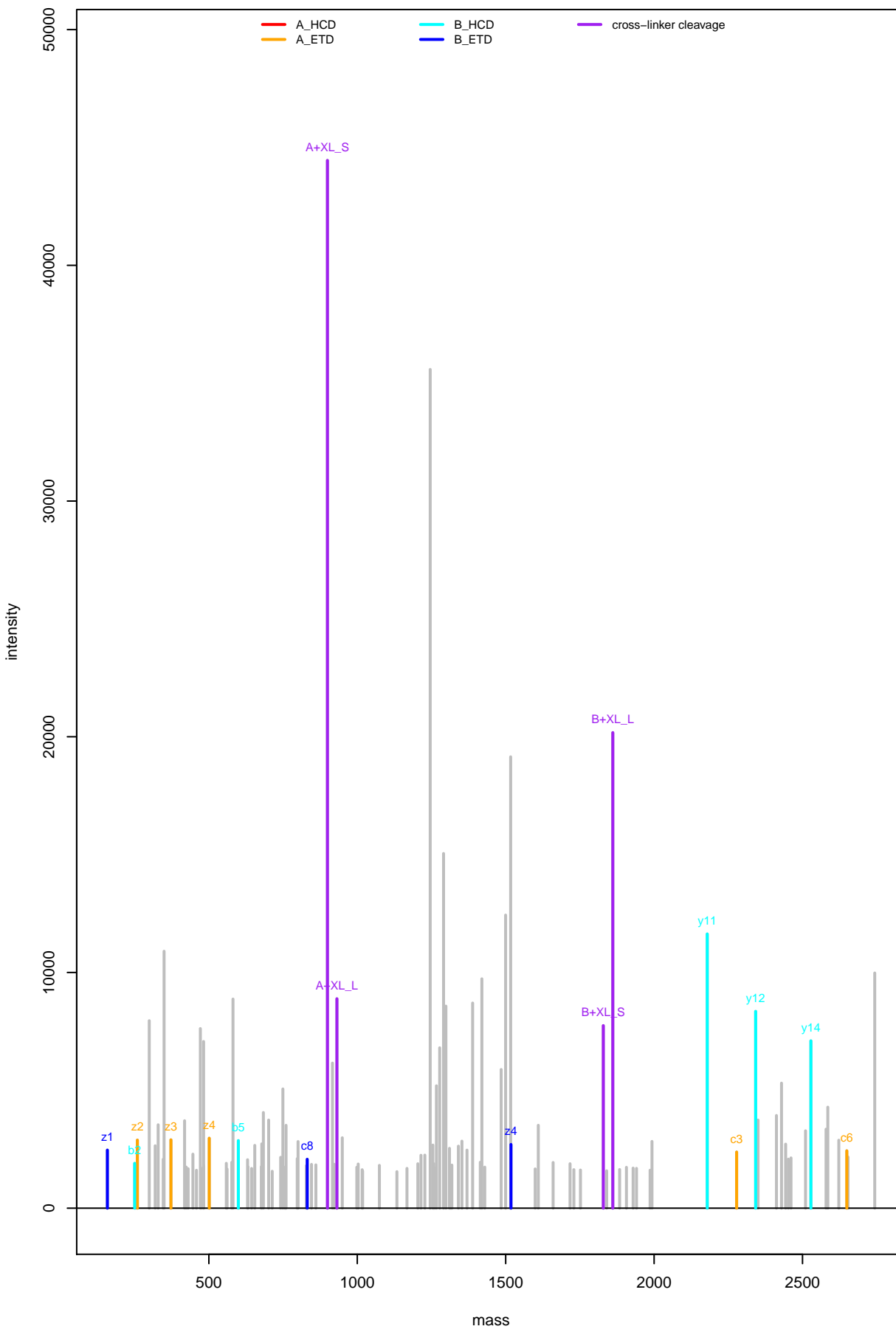

**AKEELEK+XL\_S**

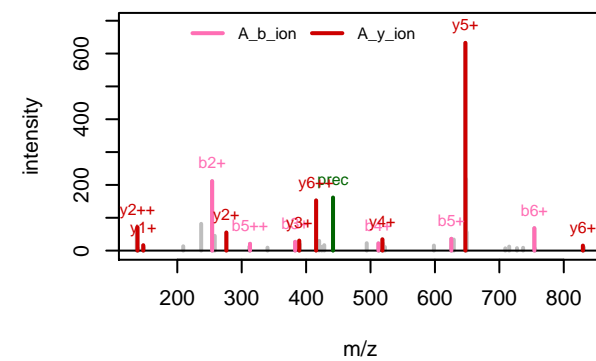

**AKEELEK+XL\_L**

**YSSVYGASVSEDLKR+XL\_S**

**YSSVYGASVSEDLKR+XL\_L**

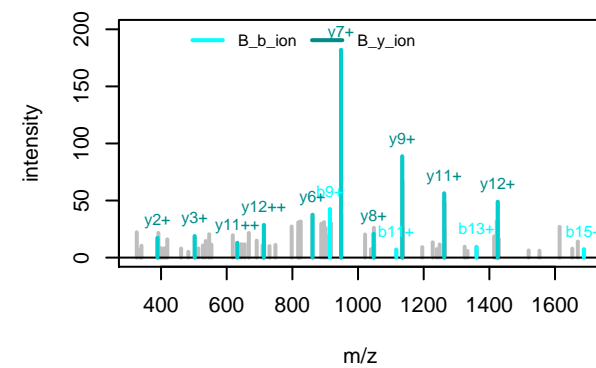

Supplement: Supplemental Data [file supp_RA117.000470_133922_0_supp_23978_fzffwf.zip › spectra_annotation/mito_DR_spectra_annotation/118-1-3-1-1-1.pdf]

MVAAAKYAR  
TSGSSIKIVK

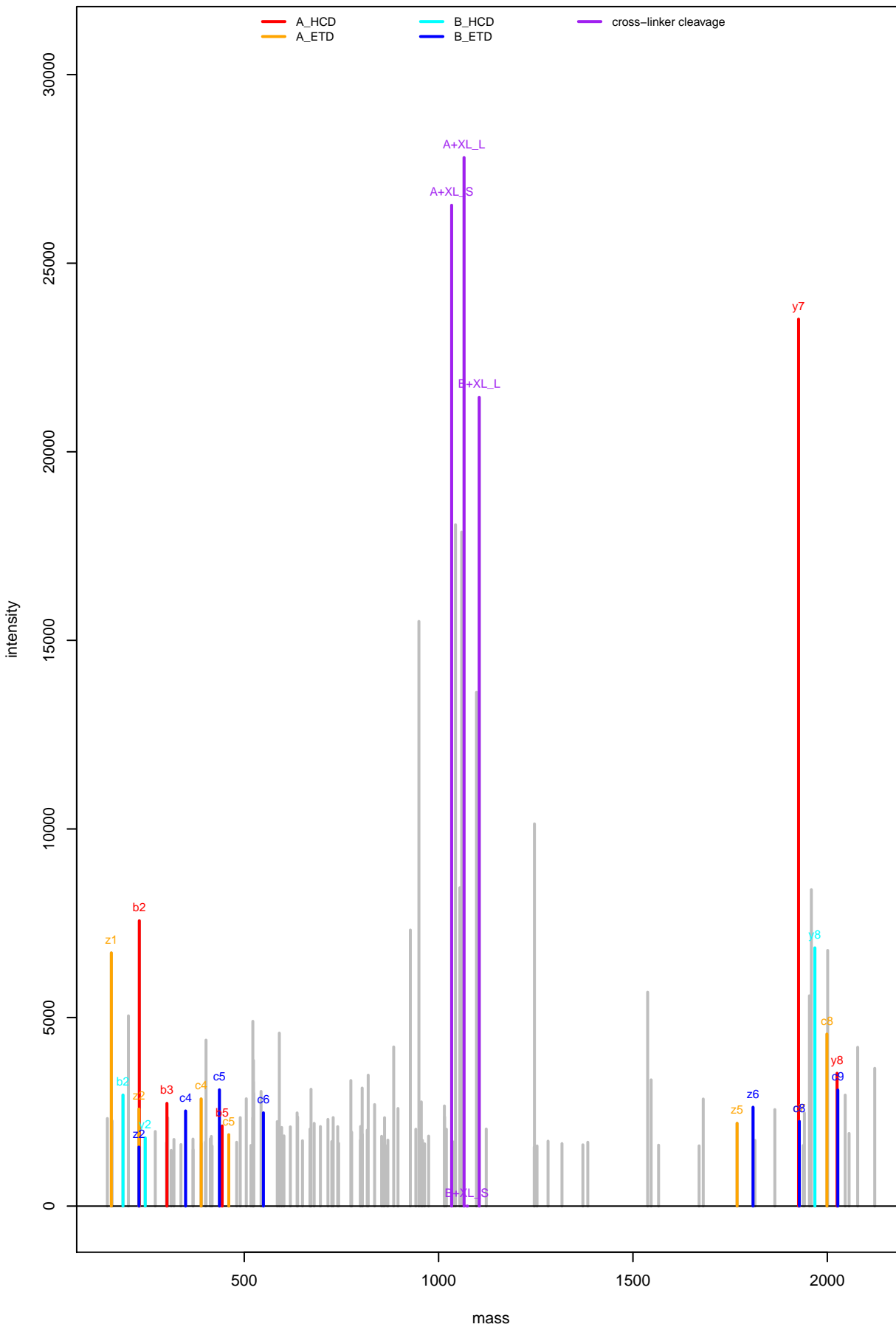

MVAAAKYAR+XL\_S

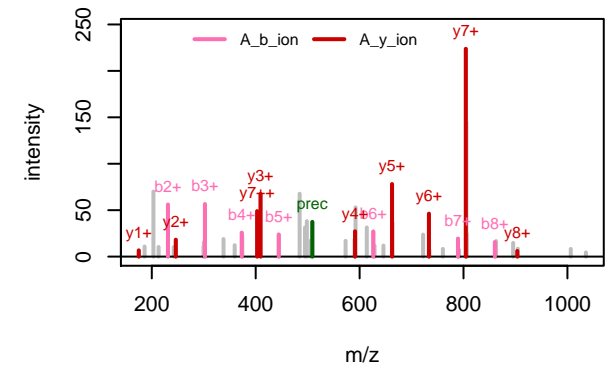

MVAAAKYAR+XL\_L

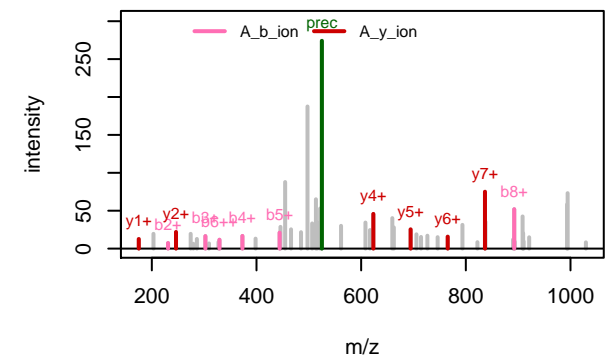

TSGSSIKIVK+XL\_S

TSGSSIKIVK+XL\_L

Supplement: Supplemental Data [file supp_RA117.000470_133922_0_supp_23978_fzffwf.zip › spectra_annotation/mito_DR_spectra_annotation/118-1-3-1-7-1.pdf]

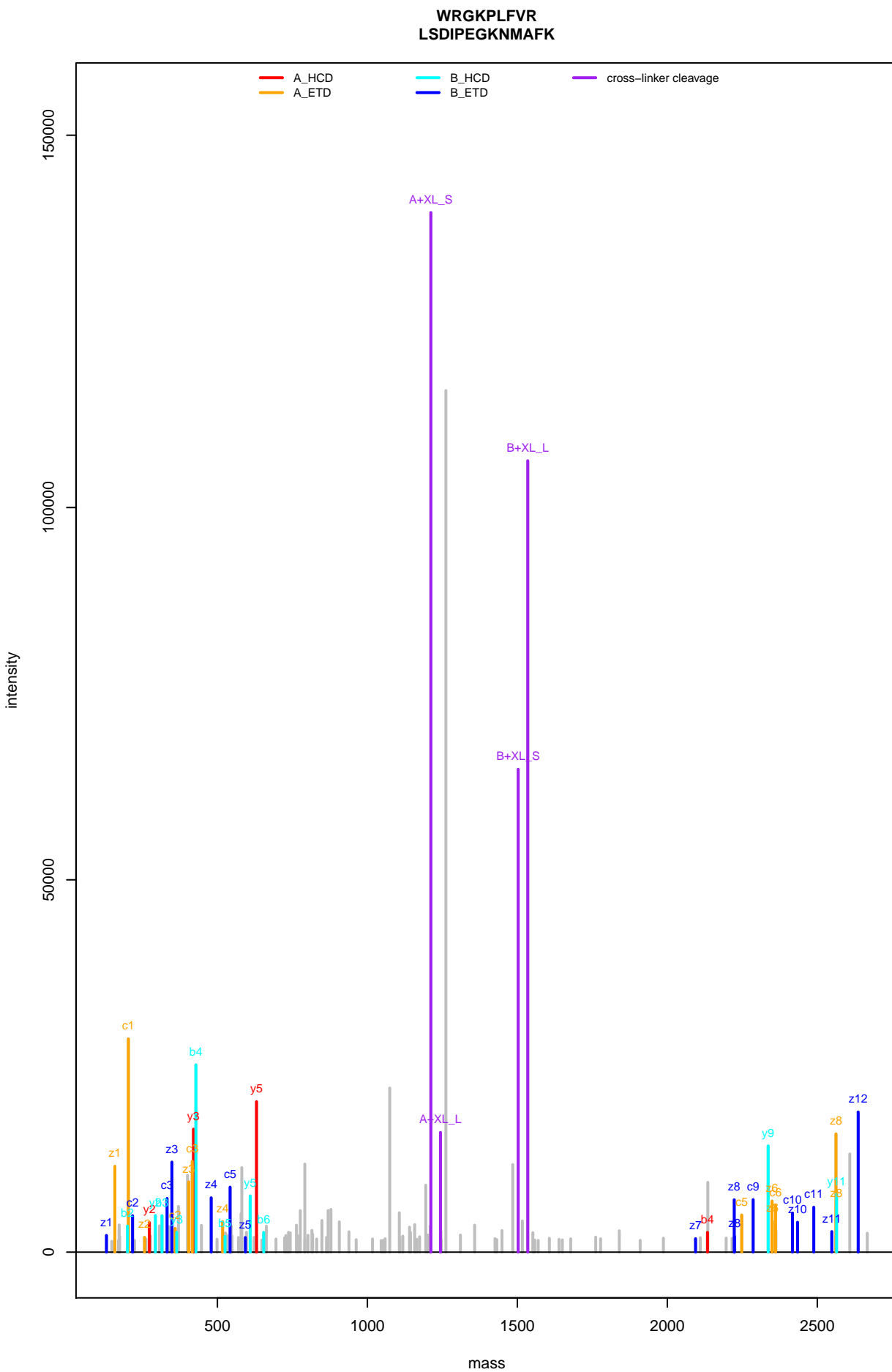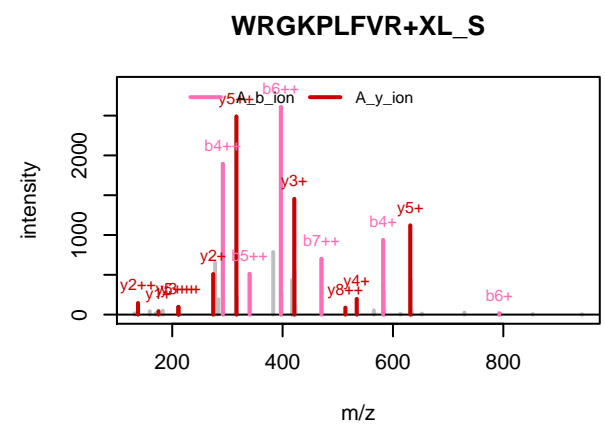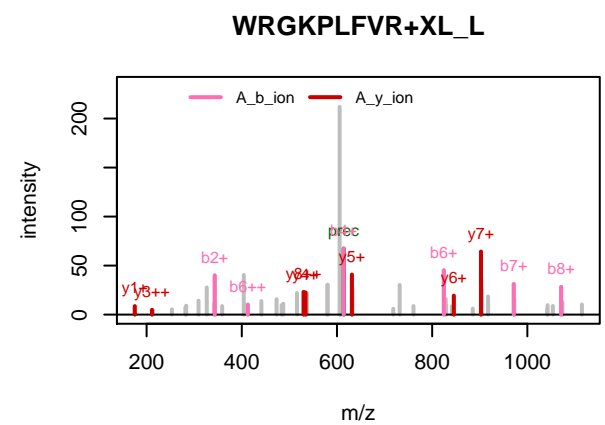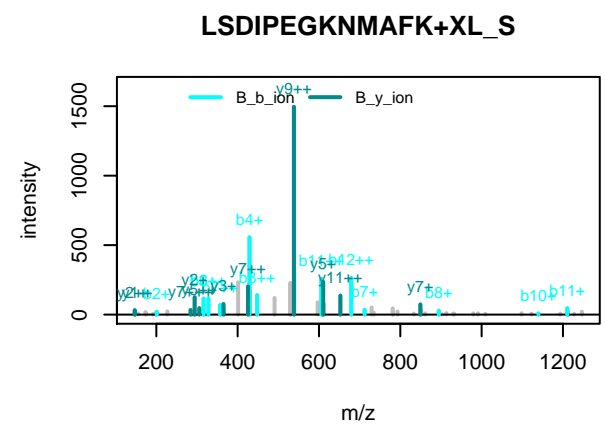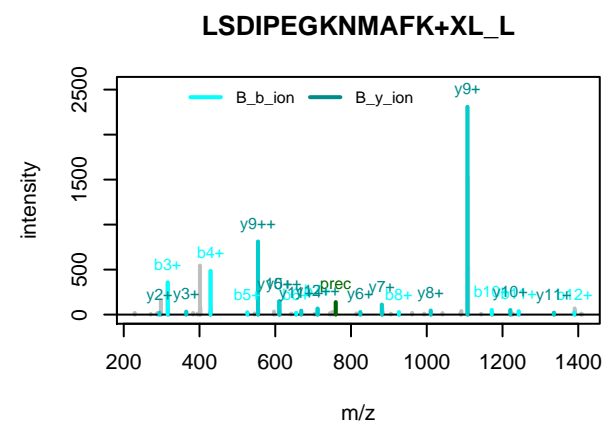

Supplement: Supplemental Data [file supp_RA117.000470_133922_0_supp_23978_fzffwf.zip › spectra_annotation/mito_DR_spectra_annotation/118-1-8-1-11-1.pdf]

# KESEHQR MKTIIEDAK

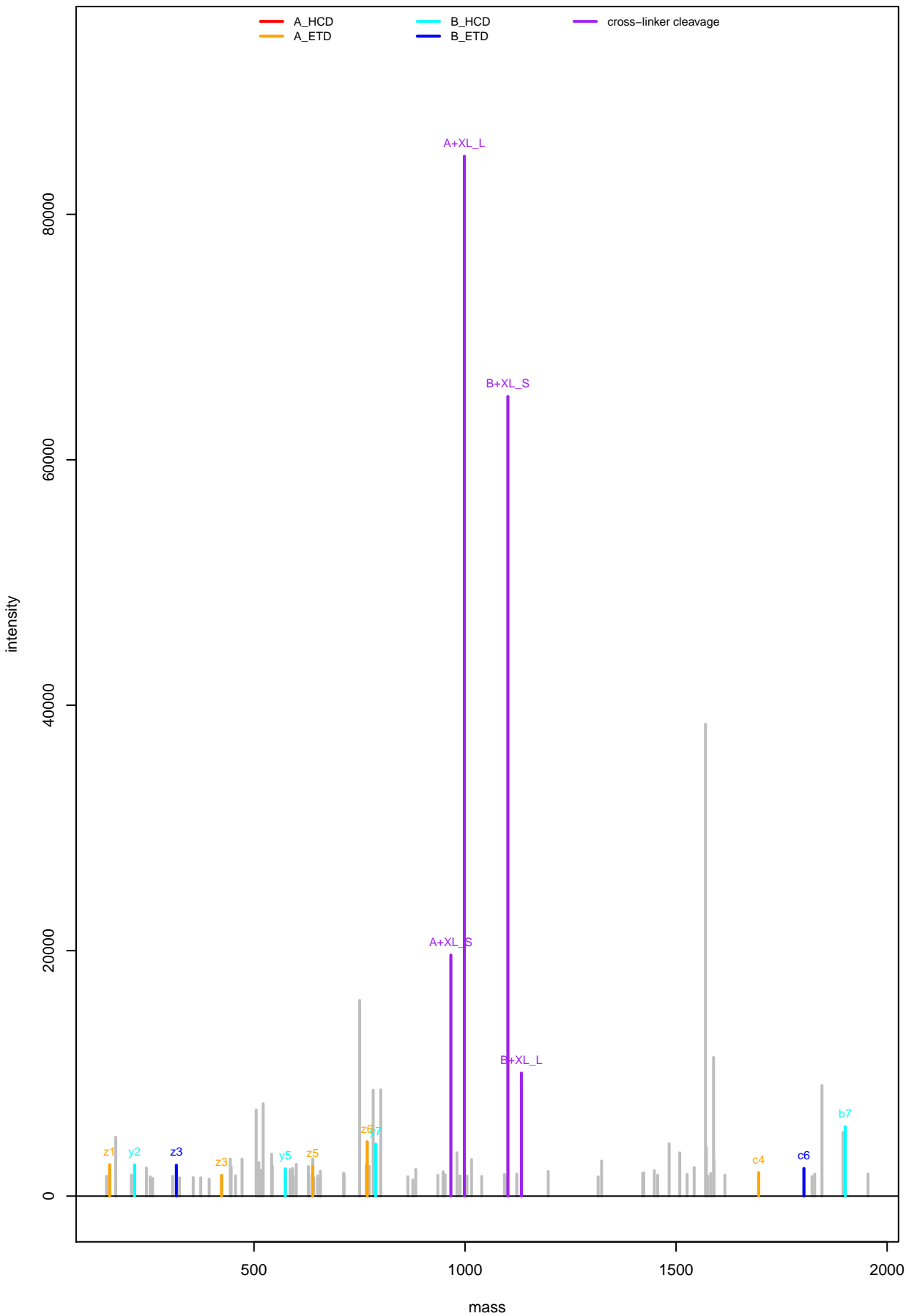

## KESEHQR+XL\_S

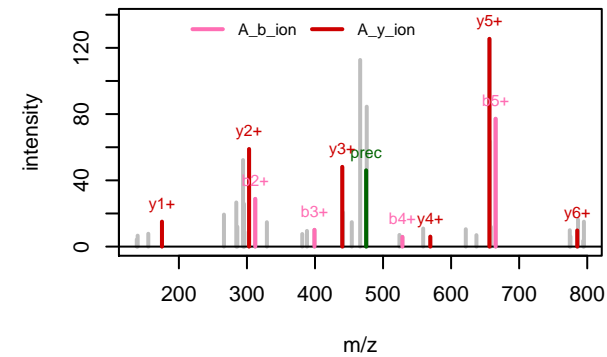

## KESEHQR+XL\_L

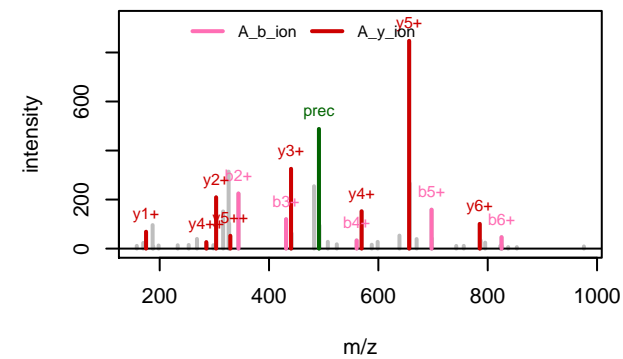

## MKTIIEDAK+XL\_S

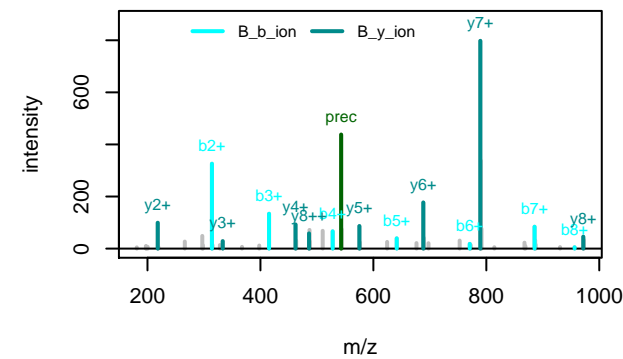

## MKTIIEDAK+XL\_L

Supplement: Supplemental Data [file supp_RA117.000470_133922_0_supp_23978_fzffwf.zip › spectra_annotation/mito_DR_spectra_annotation/119-1-1-1-13-1.pdf]

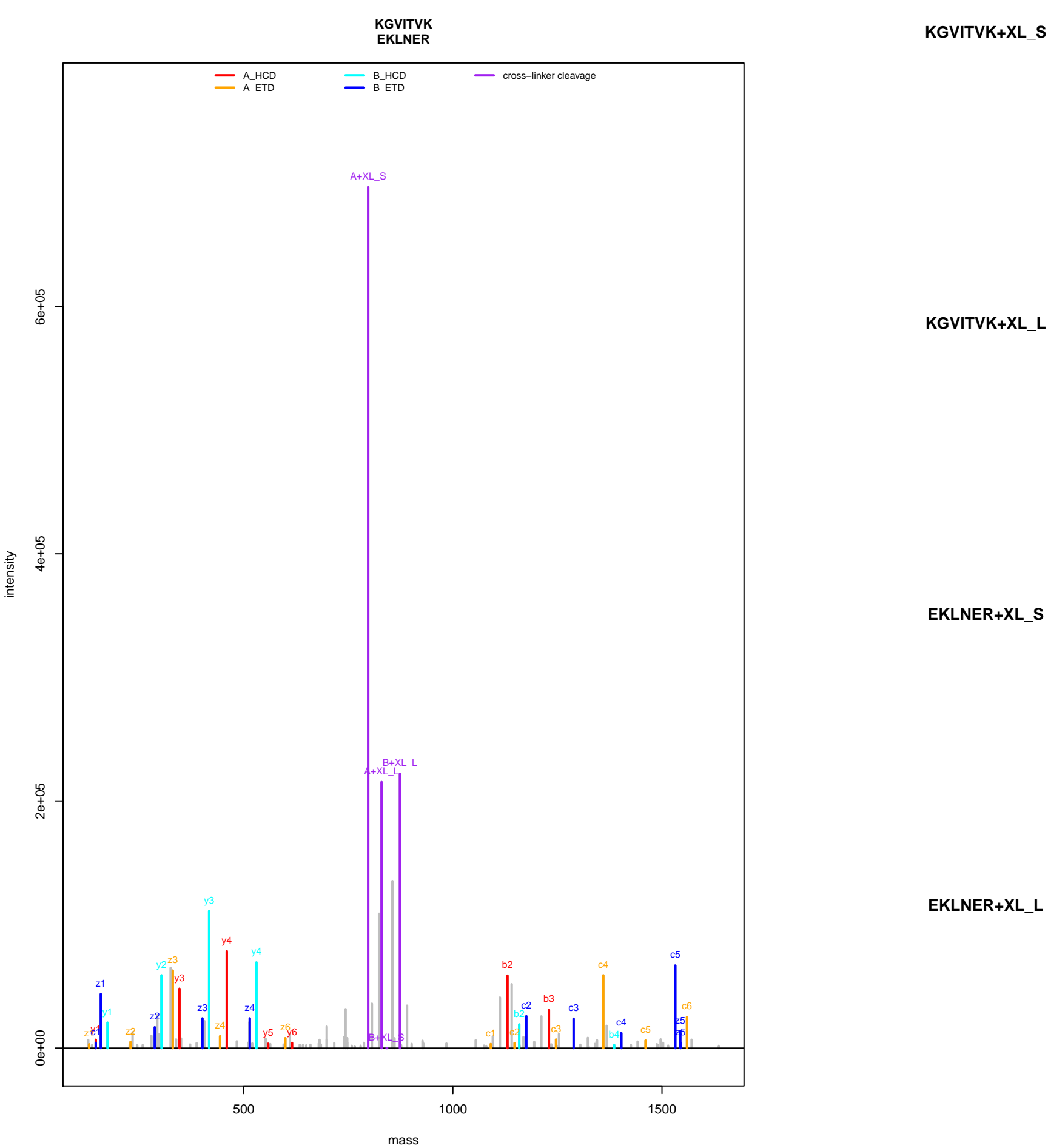

Supplement: Supplemental Data [file supp_RA117.000470_133922_0_supp_23978_fzffwf.zip › spectra_annotation/mito_DR_spectra_annotation/119-1-1-1-3-1.pdf]

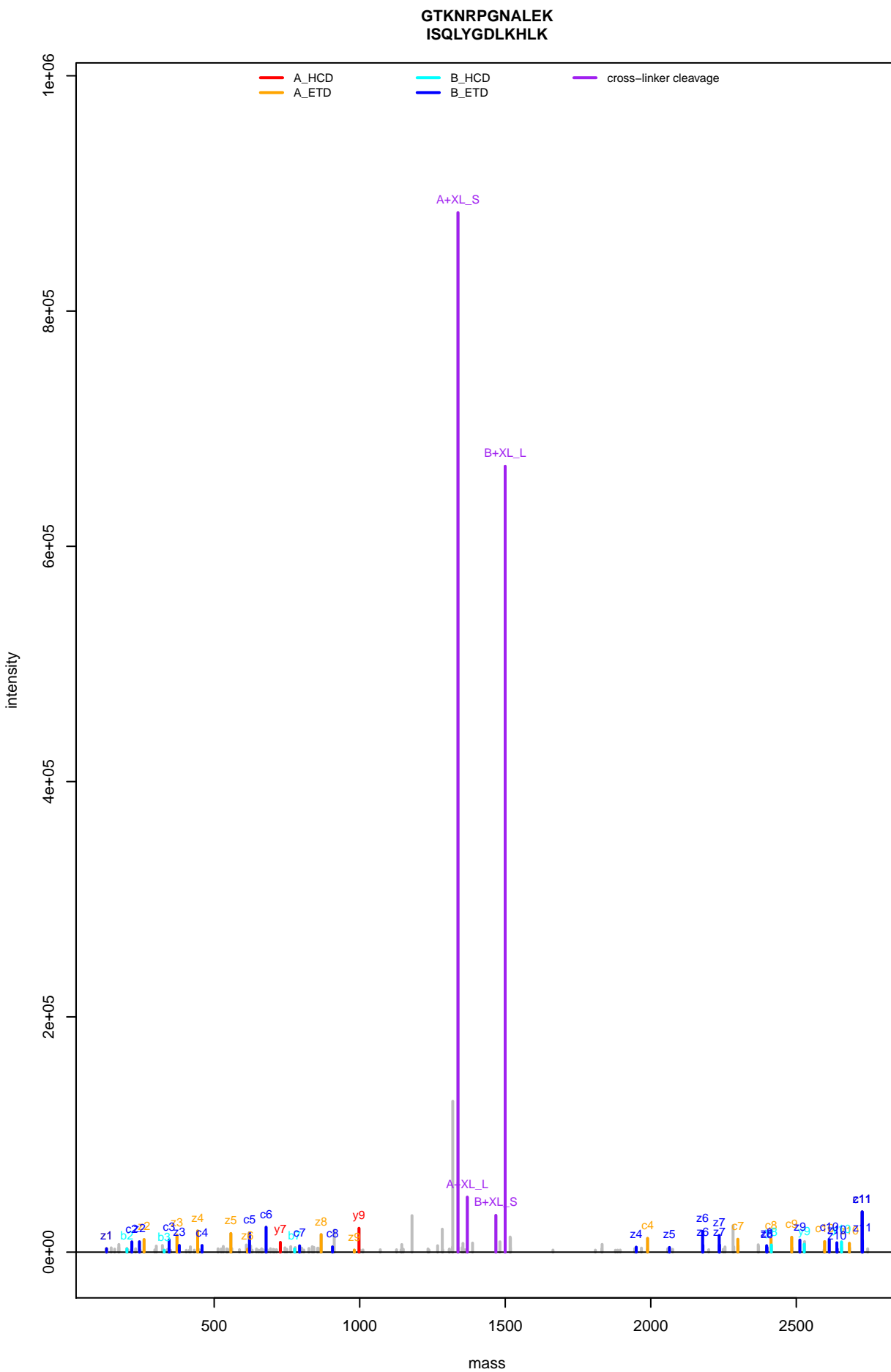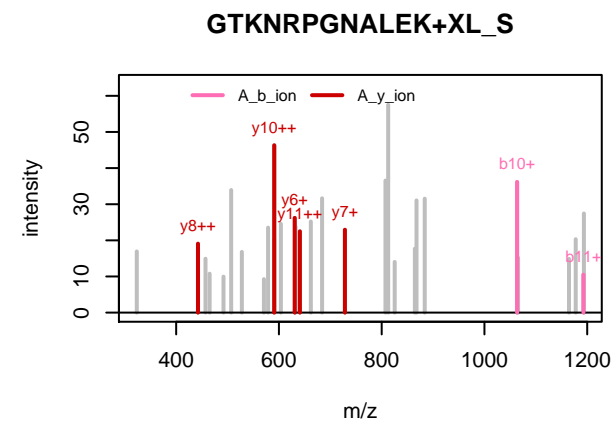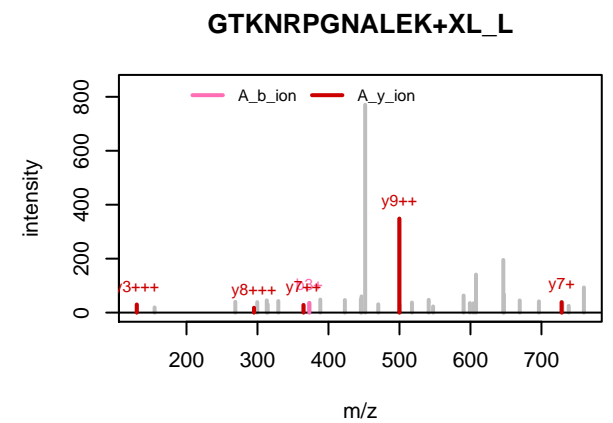

**ISQLYGDLKHLK+XL\_S**

**ISQLYGDLKHLK+XL\_L**

Supplement: Supplemental Data [file supp_RA117.000470_133922_0_supp_23978_fzffwf.zip › spectra_annotation/mito_DR_spectra_annotation/119-2-11-1-20-1.pdf]

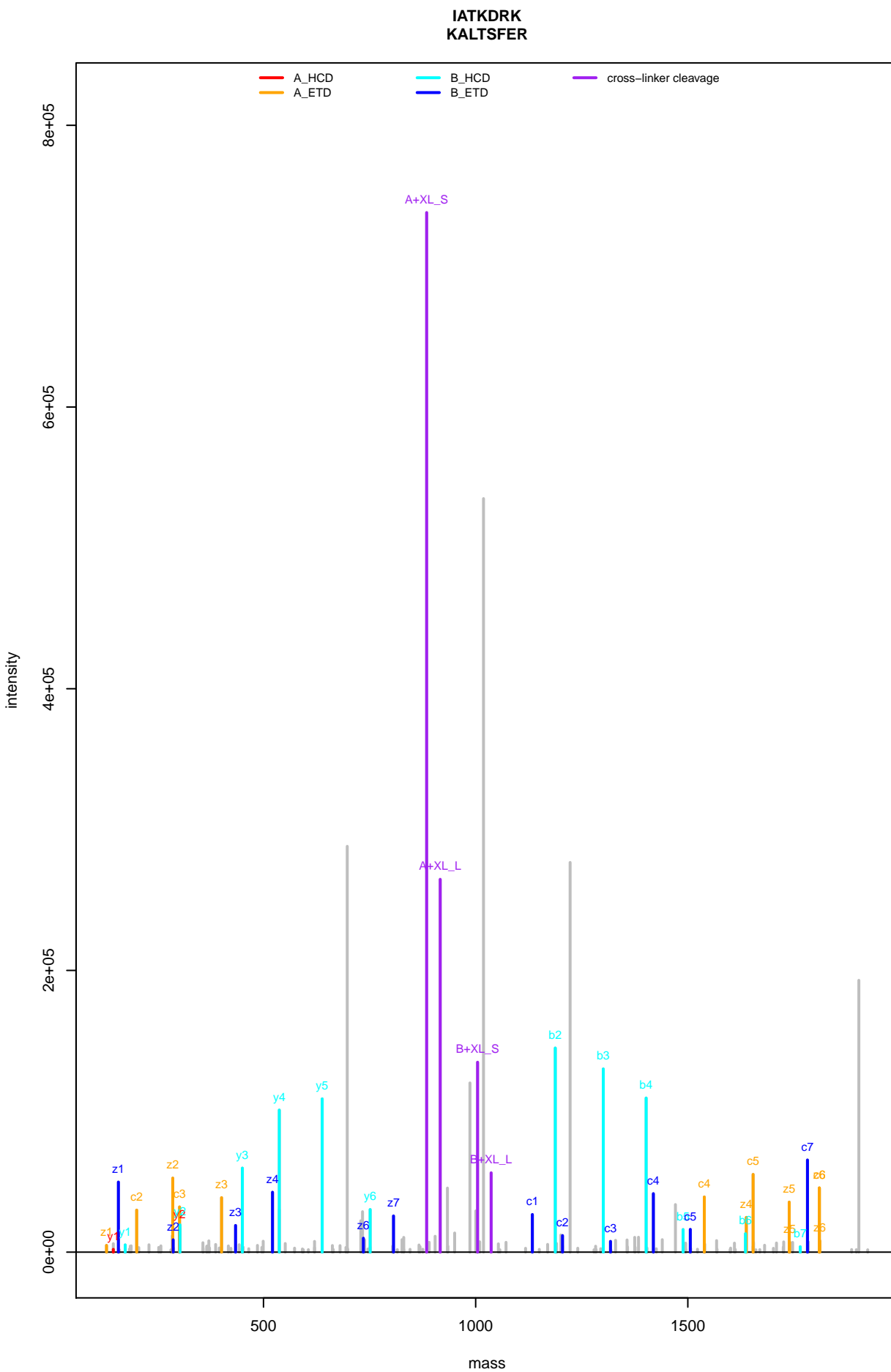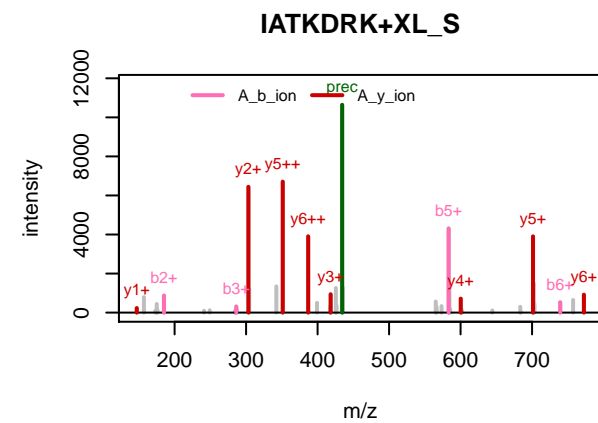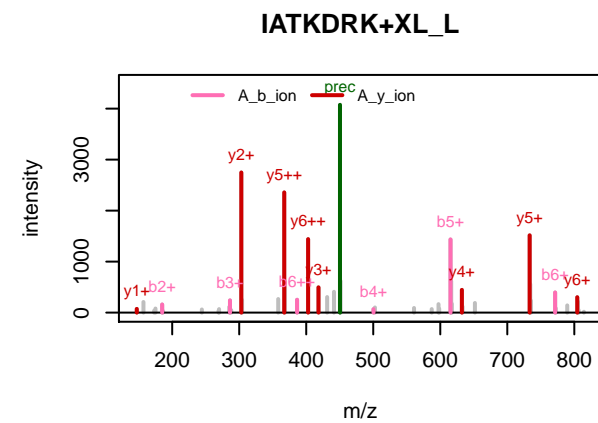

**KALTSFER+XL\_S**

**KALTSFER+XL\_L**

Supplement: Supplemental Data [file supp_RA117.000470_133922_0_supp_23978_fzffwf.zip › spectra_annotation/mito_DR_spectra_annotation/119-2-4-1-5-1.pdf]

# ITKSMK MVAAAKYAR

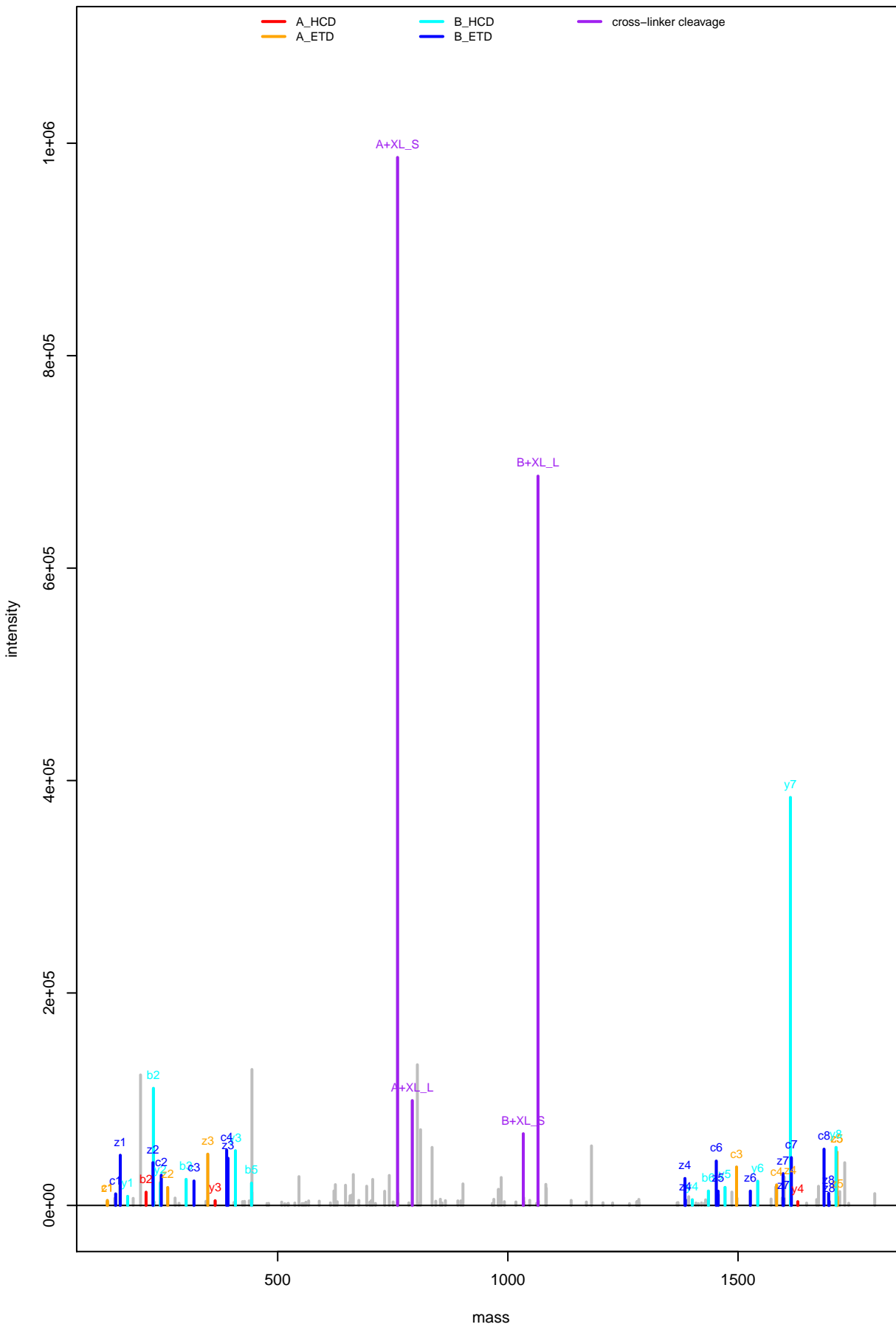

## ITKSMK+XL\_S

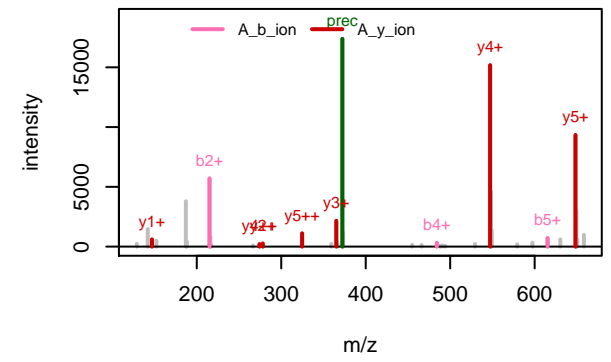

## ITKSMK+XL\_L

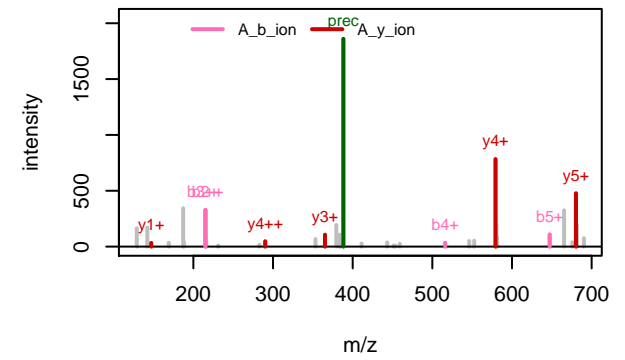

## MVAAAKYAR+XL\_S

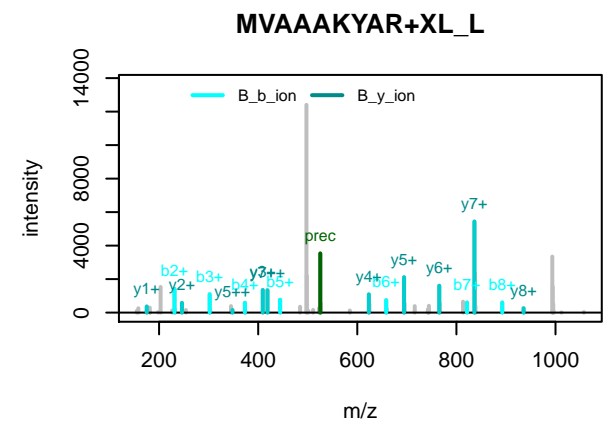

Supplement: Supplemental Data [file supp_RA117.000470_133922_0_supp_23978_fzffwf.zip › spectra_annotation/mito_DR_spectra_annotation/12-1-1-1-5-1.pdf]

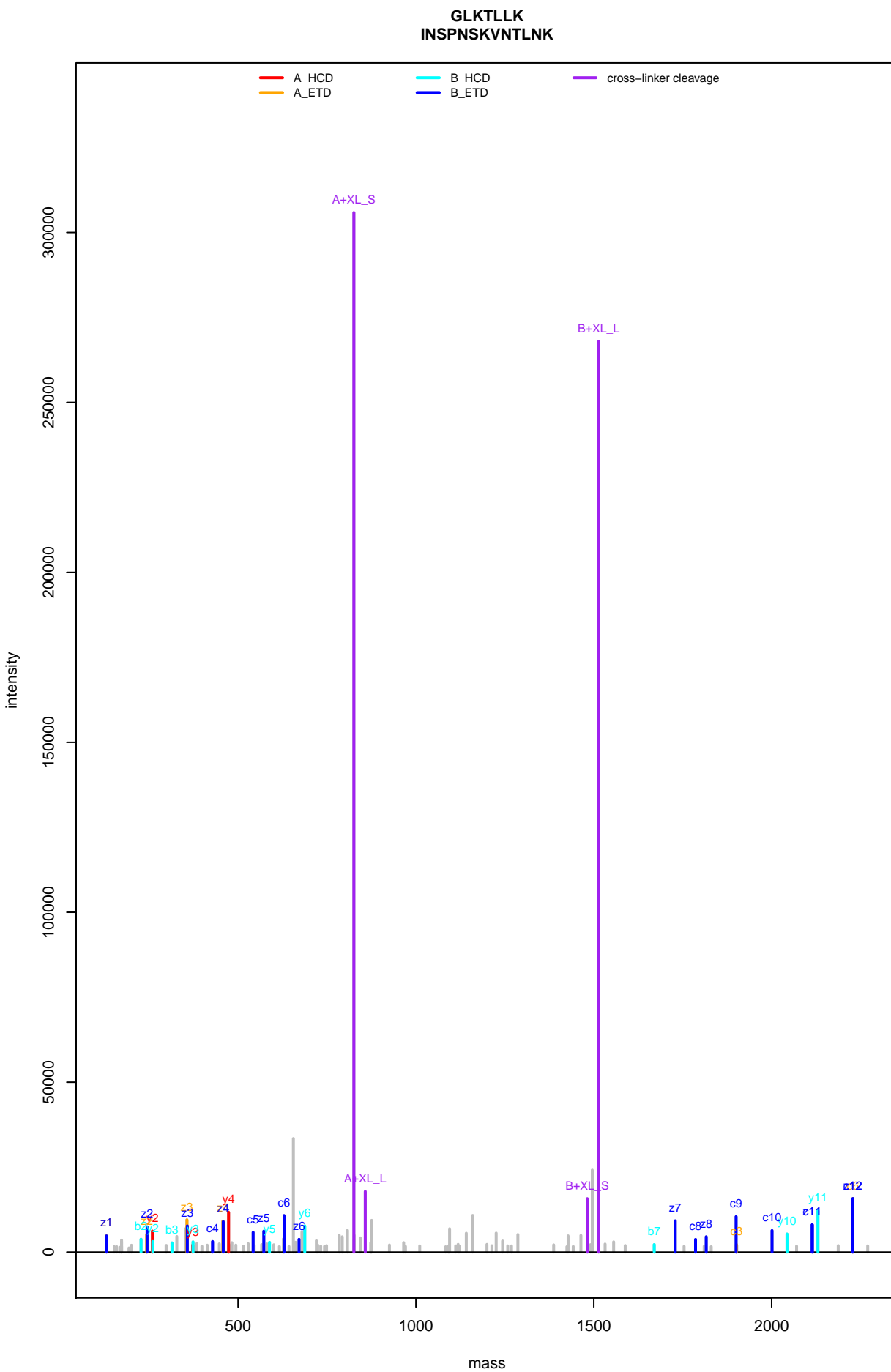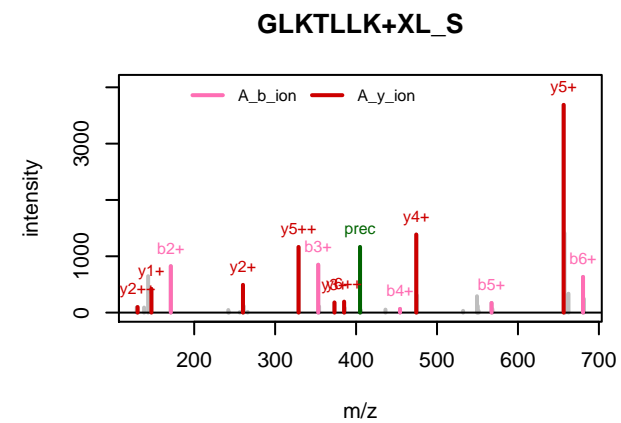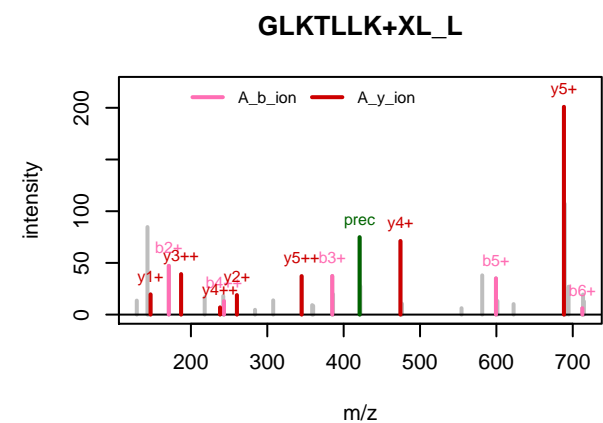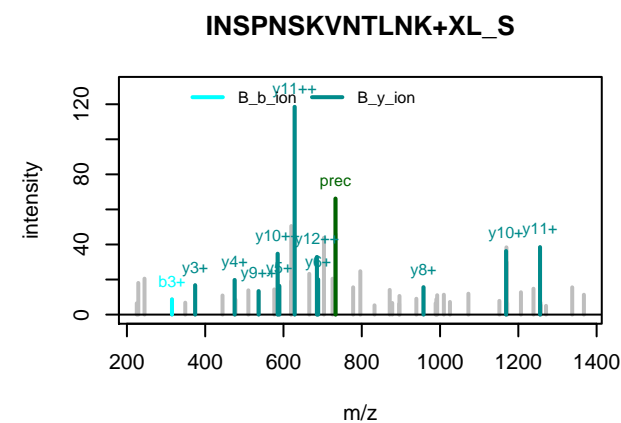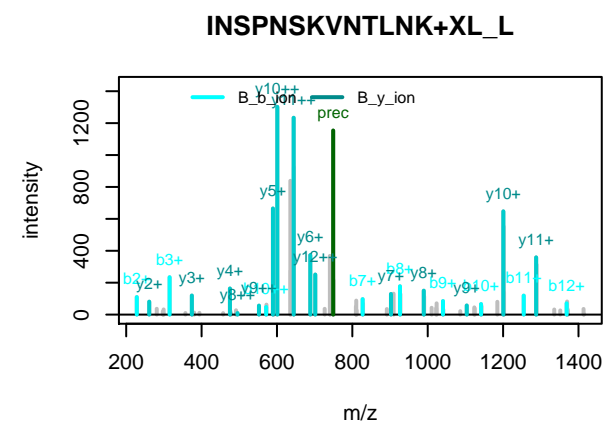

Supplement: Supplemental Data [file supp_RA117.000470_133922_0_supp_23978_fzffwf.zip › spectra_annotation/mito_DR_spectra_annotation/12-1-2-1-8-1.pdf]

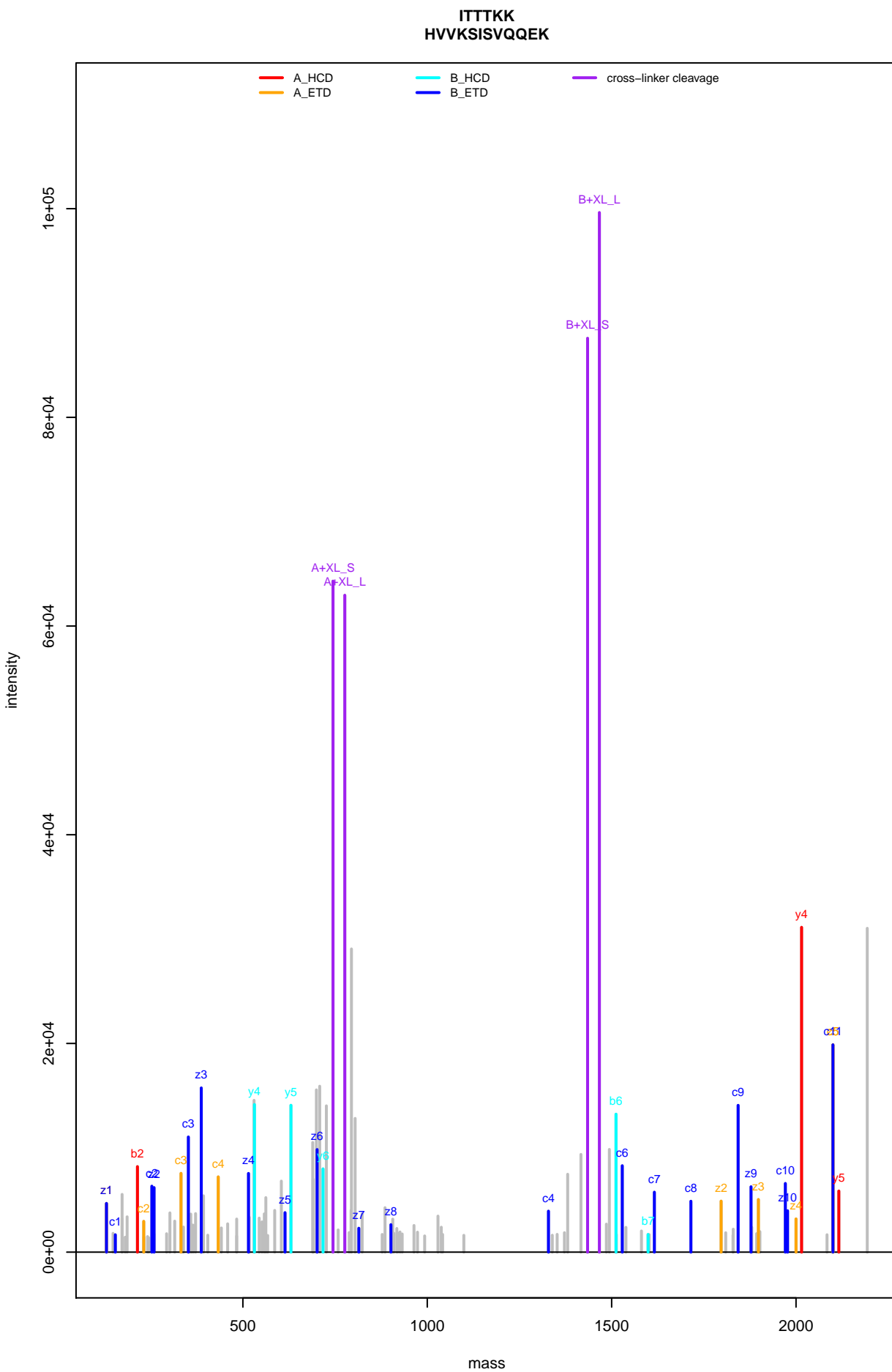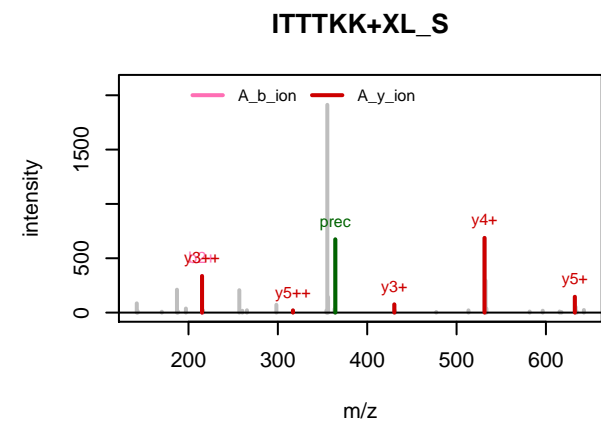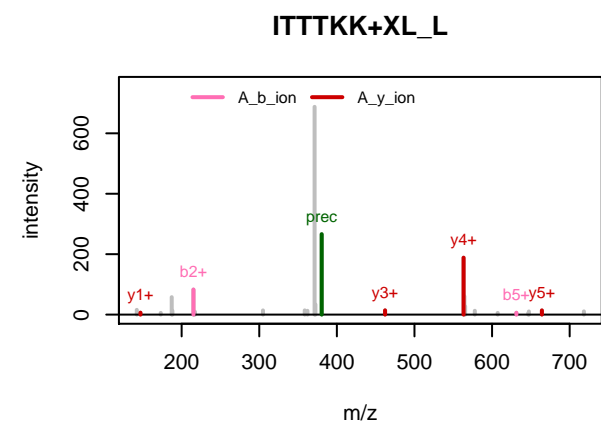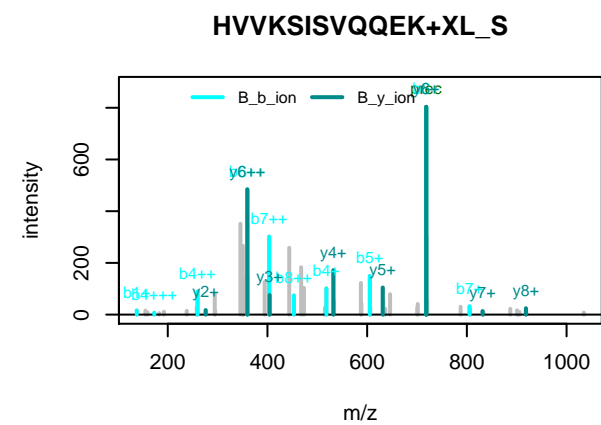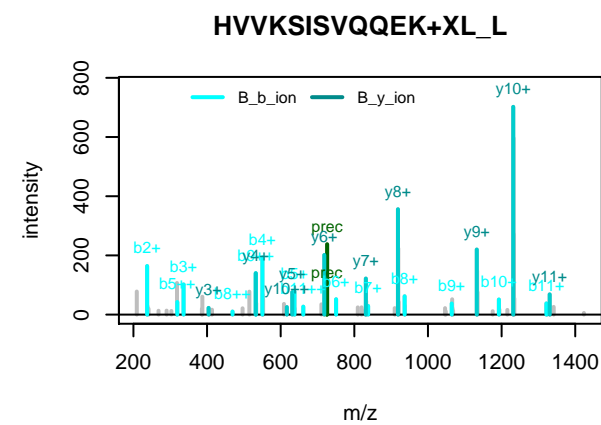

Supplement: Supplemental Data [file supp_RA117.000470_133922_0_supp_23978_fzffwf.zip › spectra_annotation/mito_DR_spectra_annotation/121-1-1-1-4-1.pdf]

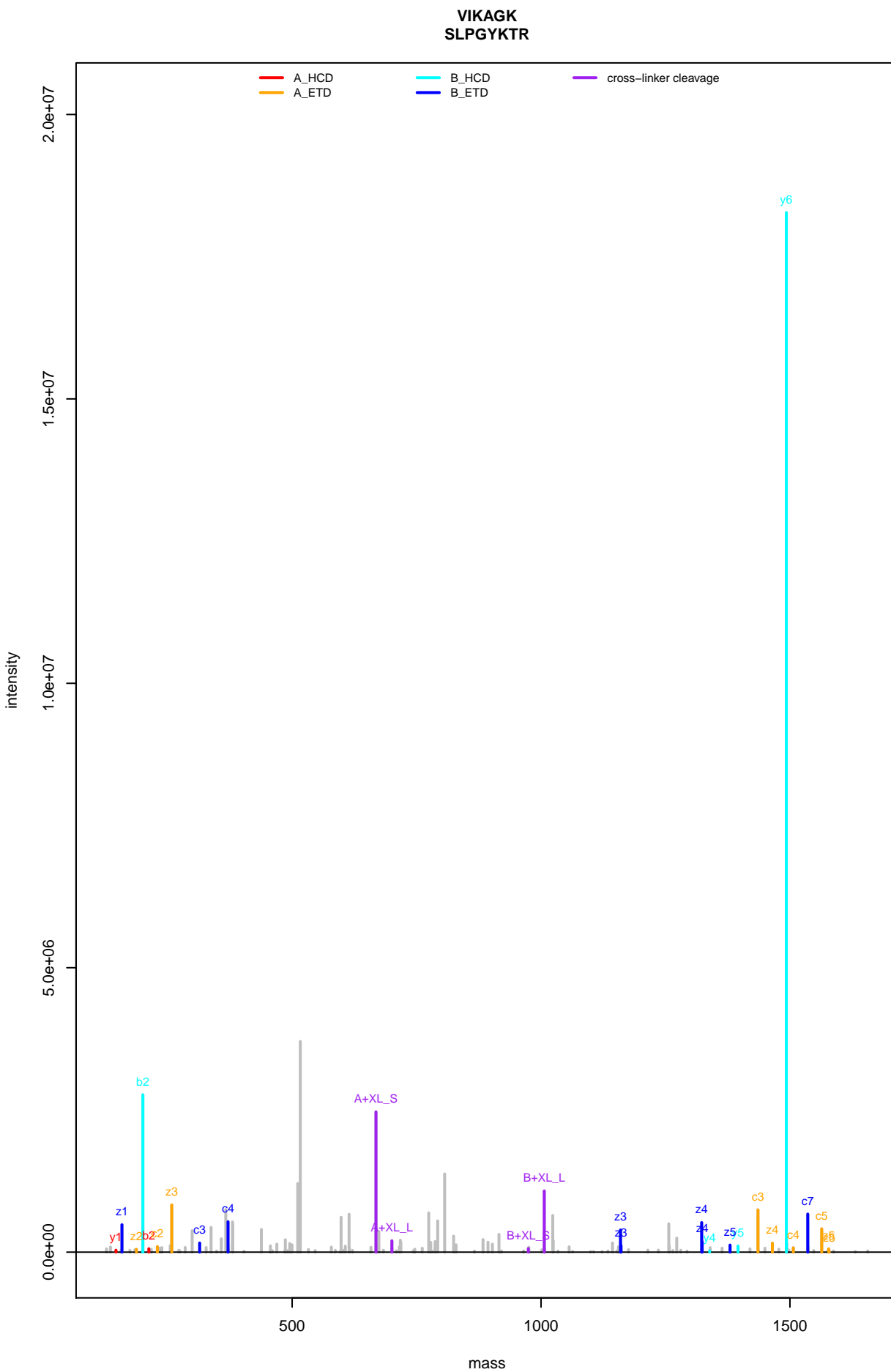

**VIKAGK+XL\_S**

**VIKAGK+XL\_L**

**SLPGYKTR+XL\_S**

**SLPGYKTR+XL\_L**

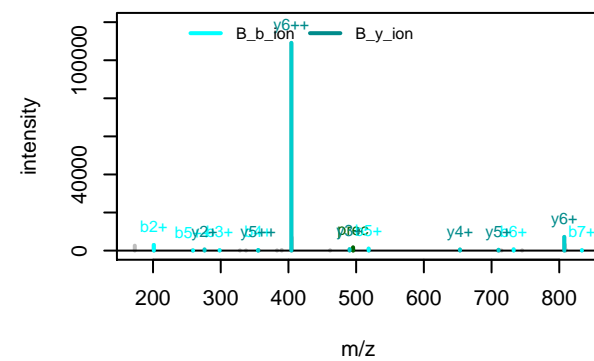

Supplement: Supplemental Data [file supp_RA117.000470_133922_0_supp_23978_fzffwf.zip › spectra_annotation/mito_DR_spectra_annotation/121-1-1-1-7-1.pdf]

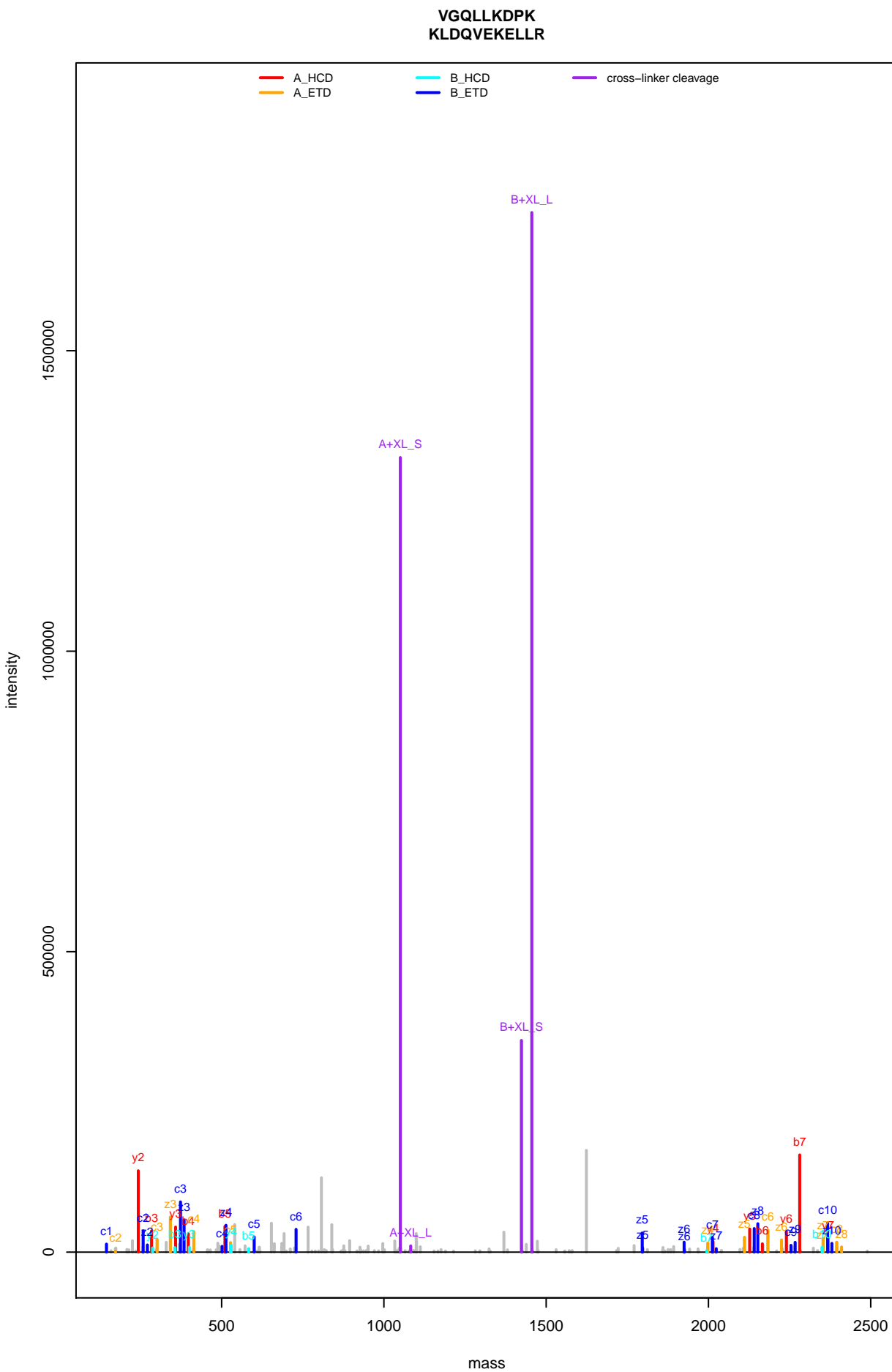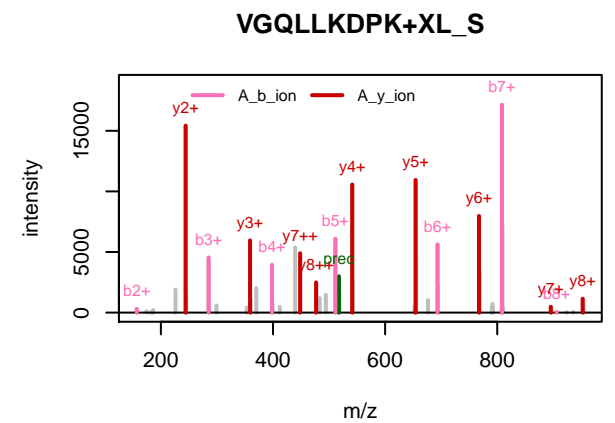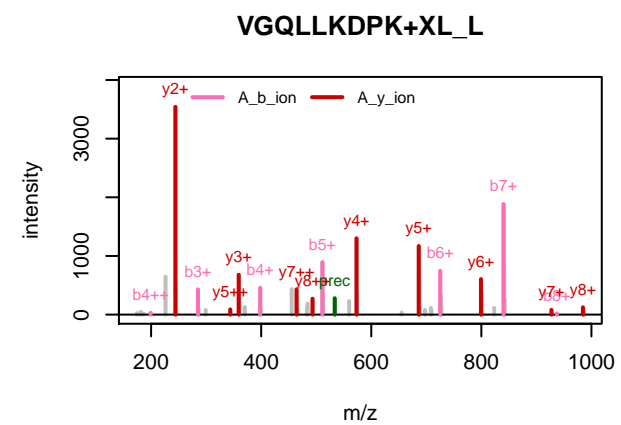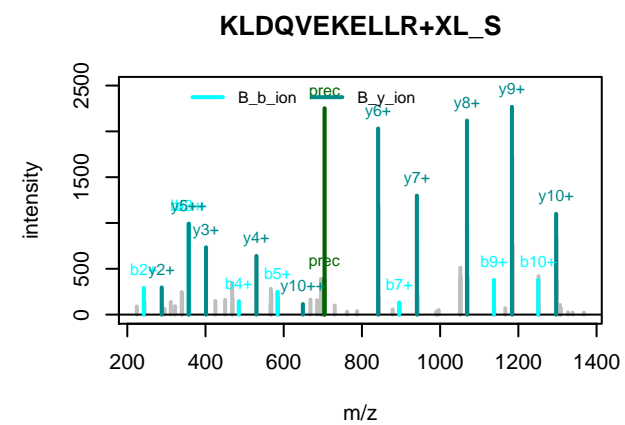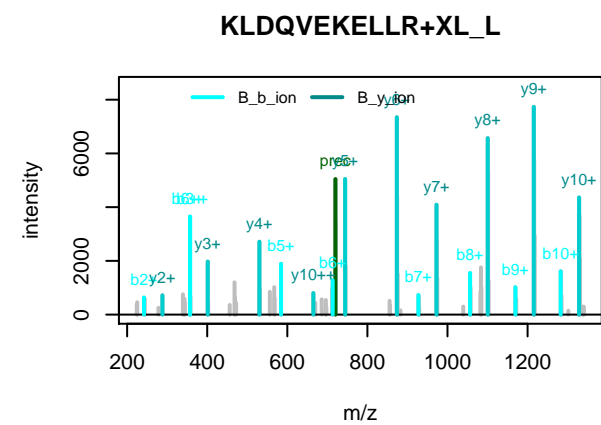

Supplement: Supplemental Data [file supp_RA117.000470_133922_0_supp_23978_fzffwf.zip › spectra_annotation/mito_DR_spectra_annotation/121-1-4-1-14-1.pdf]

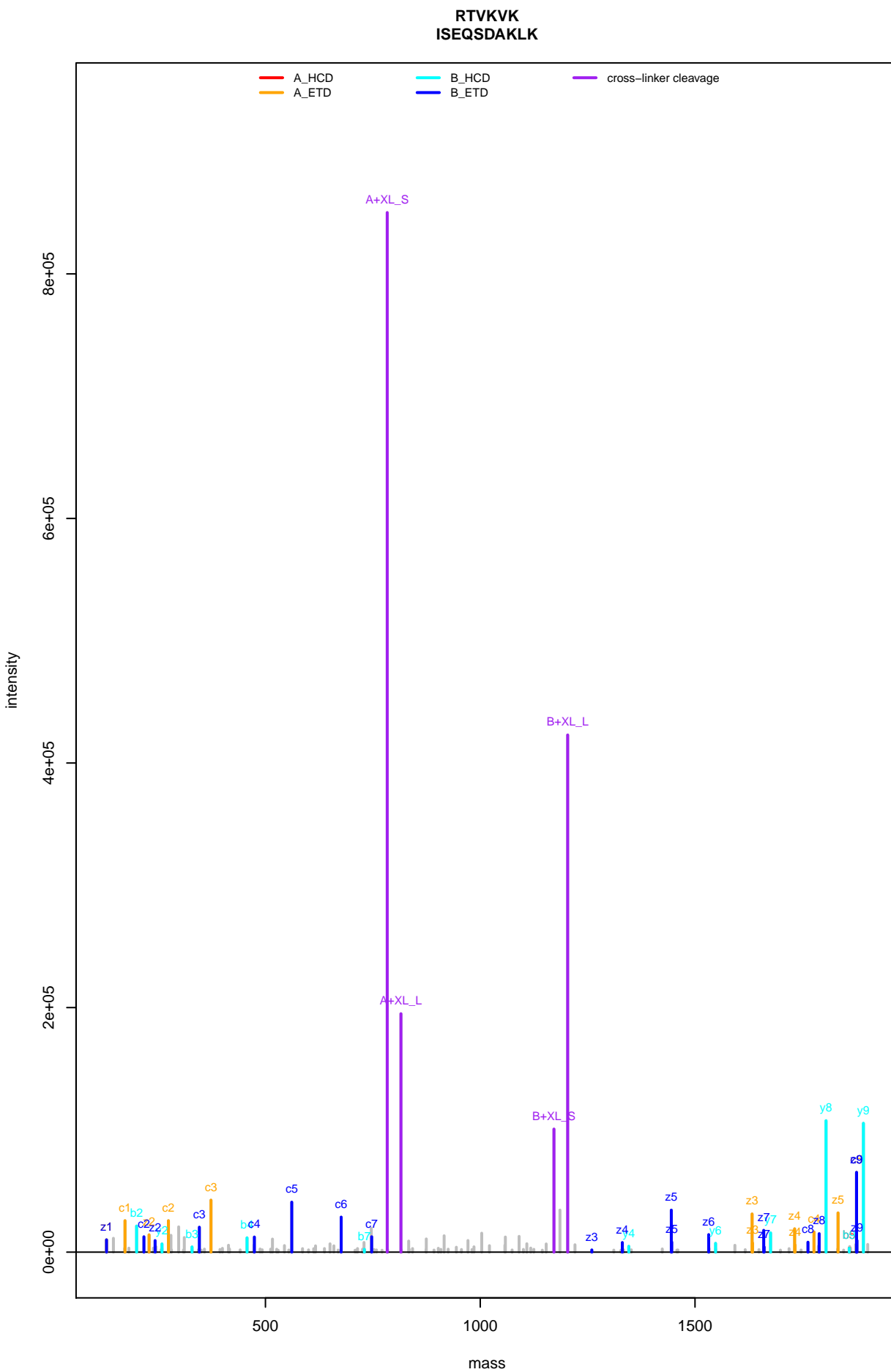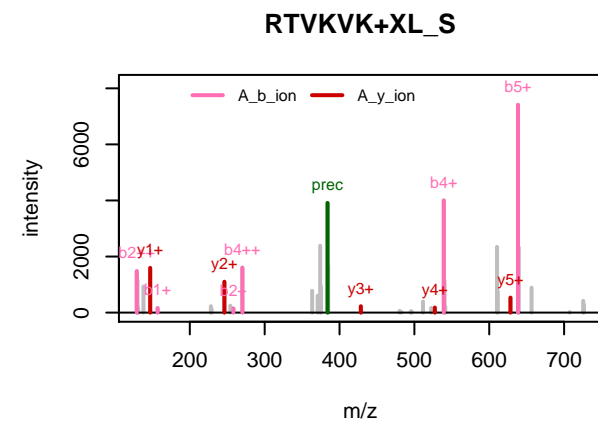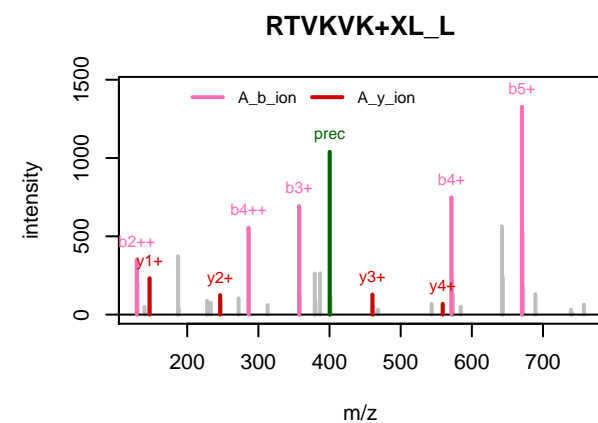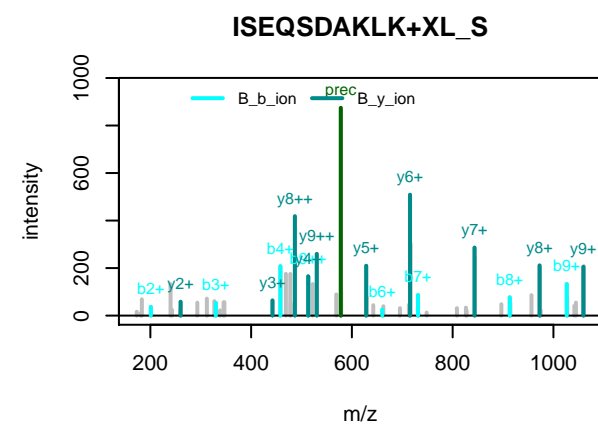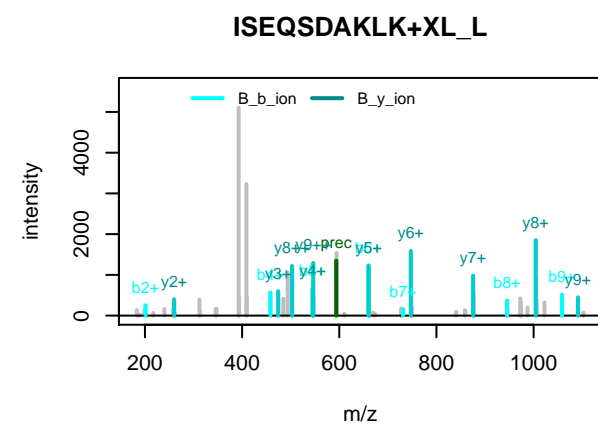

Supplement: Supplemental Data [file supp_RA117.000470_133922_0_supp_23978_fzffwf.zip › spectra_annotation/mito_DR_spectra_annotation/122-1-15-1-12-1.pdf]

VGQLLKDPK  
LIKEGDVVK

VGQLLKDPK+XL\_S

VGQLLKDPK+XL\_L

LIKEGDVVK+XL\_S

LIKEGDVVK+XL\_L

intensity

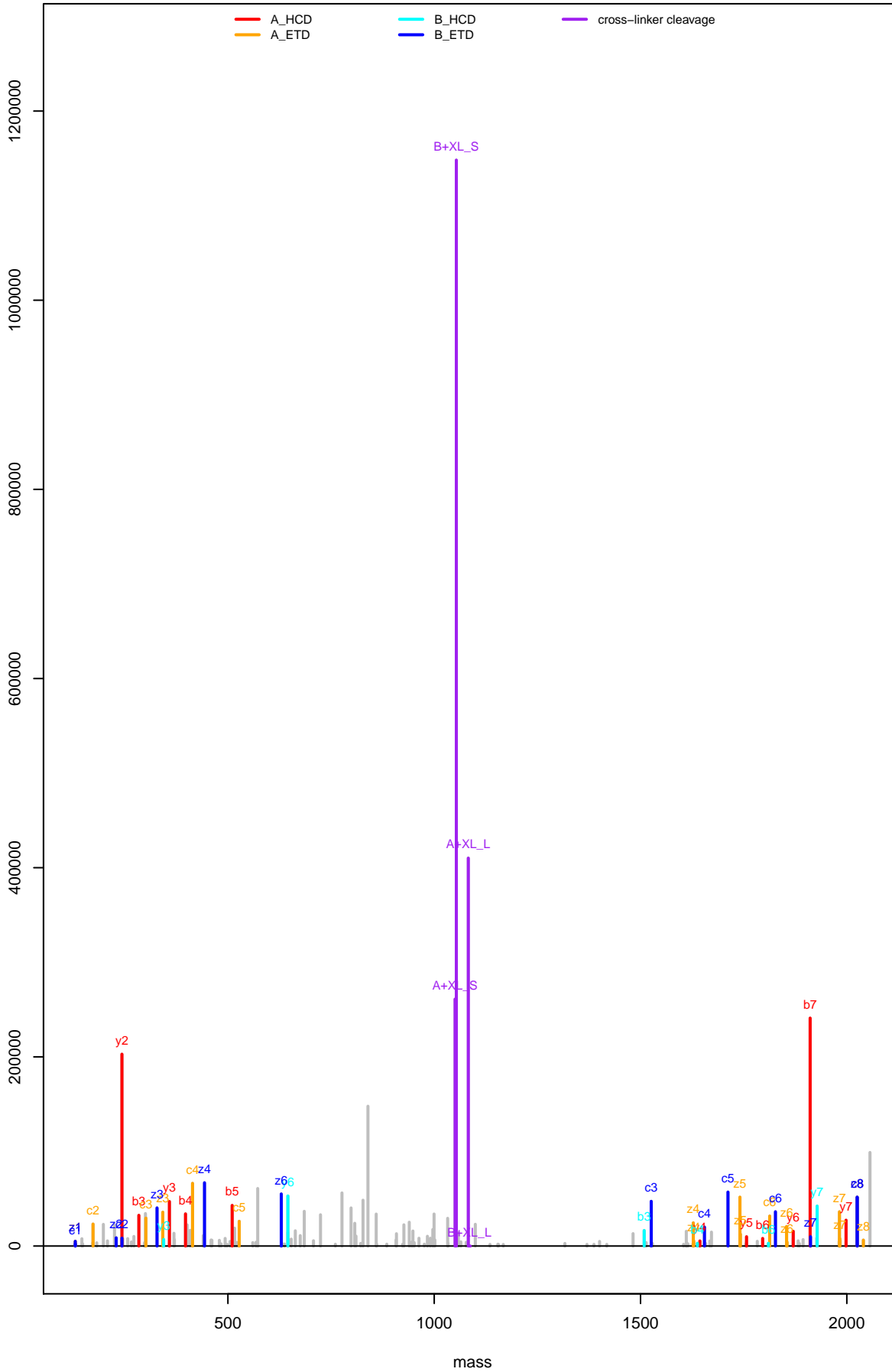

Supplement: Supplemental Data [file supp_RA117.000470_133922_0_supp_23978_fzffwf.zip › spectra_annotation/mito_DR_spectra_annotation/122-1-4-1-9-1.pdf]

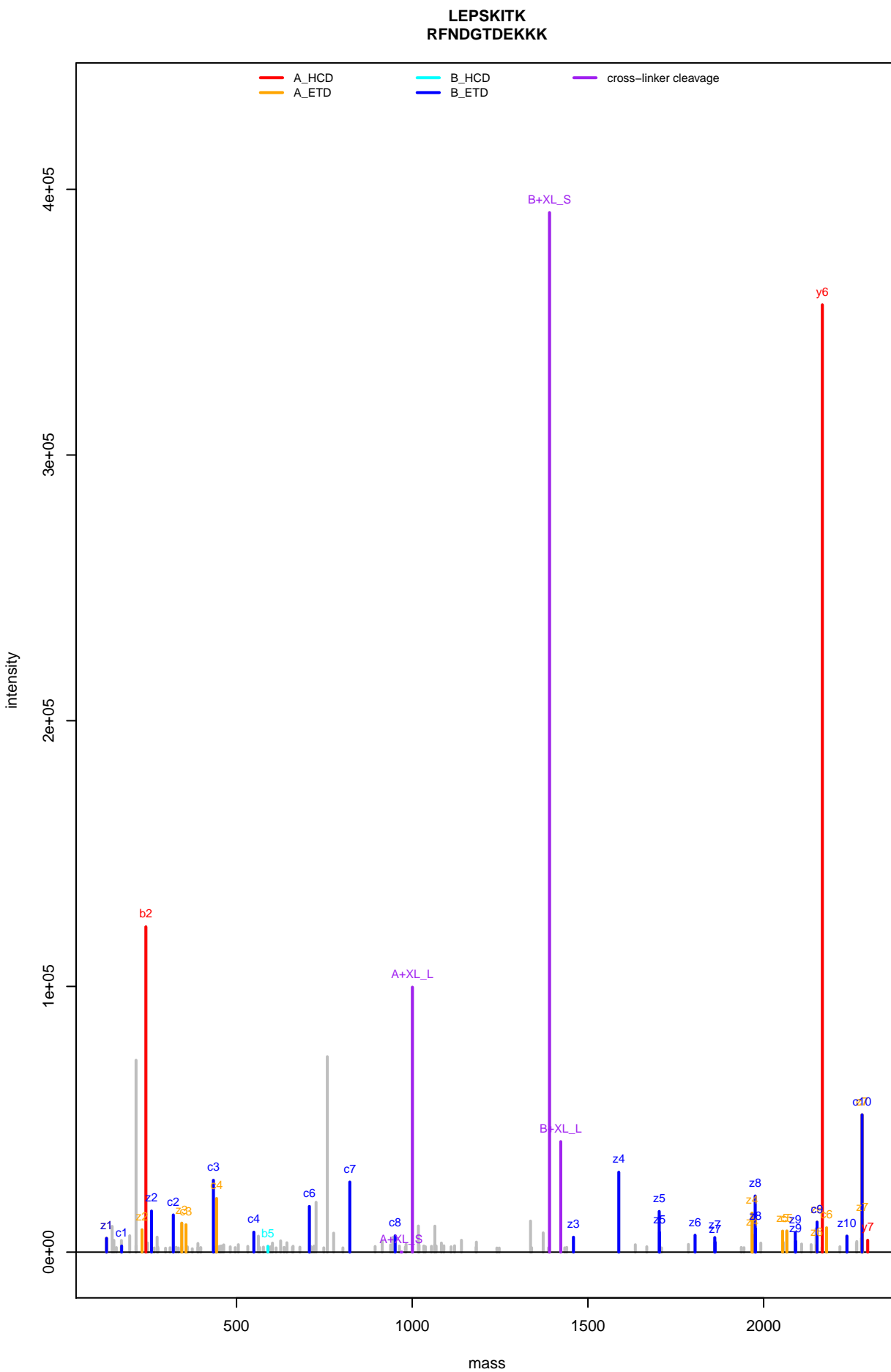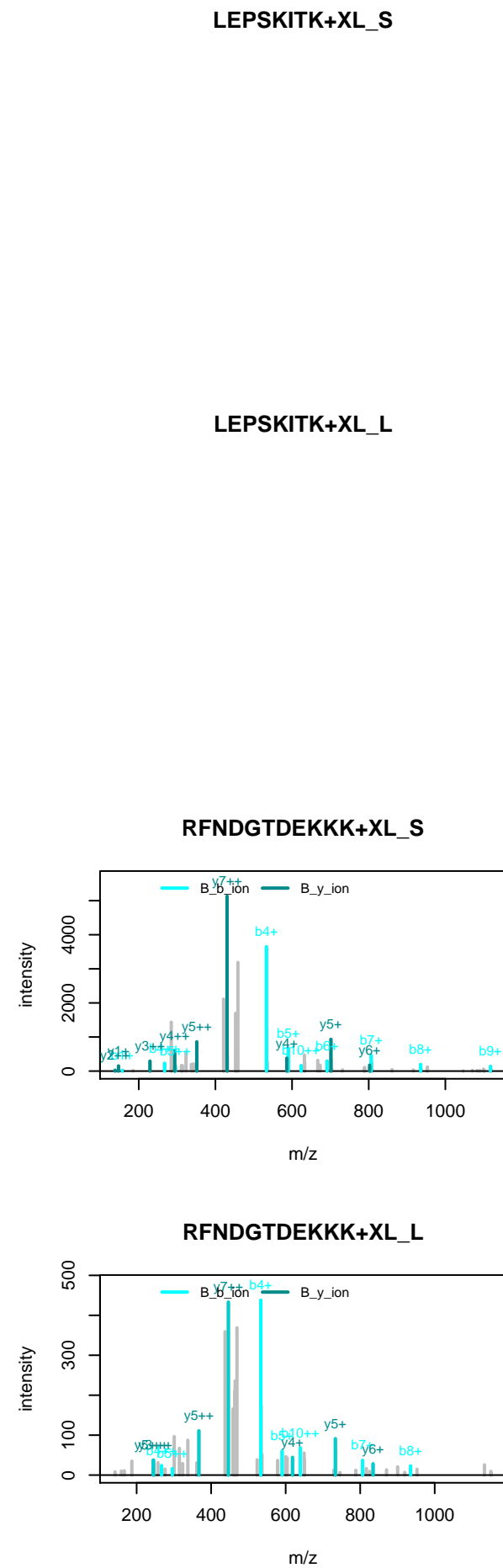

Supplement: Supplemental Data [file supp_RA117.000470_133922_0_supp_23978_fzffwf.zip › spectra_annotation/mito_DR_spectra_annotation/123-1-19-1-15-1.pdf]

SVISYKTEEK  
GLCGAIHSSVAKQMK

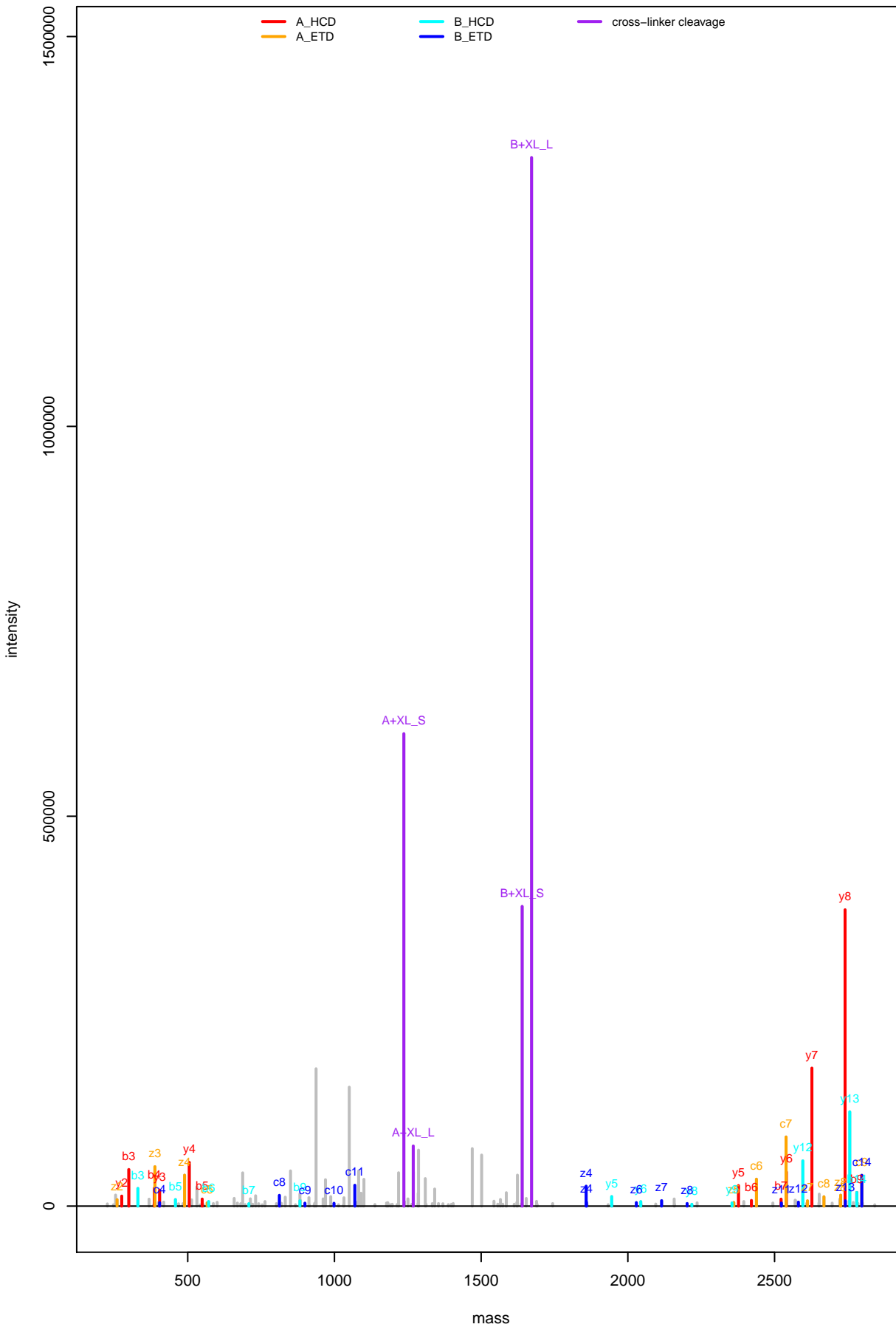

SVISYKTEEK+XL\_S

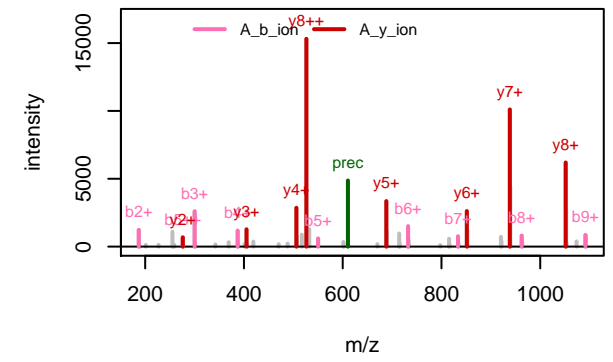

SVISYKTEEK+XL\_L

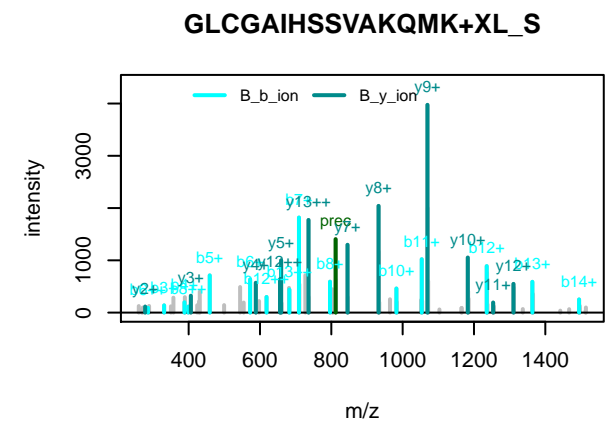

GLCGAIHSSVAKQMK+XL\_L

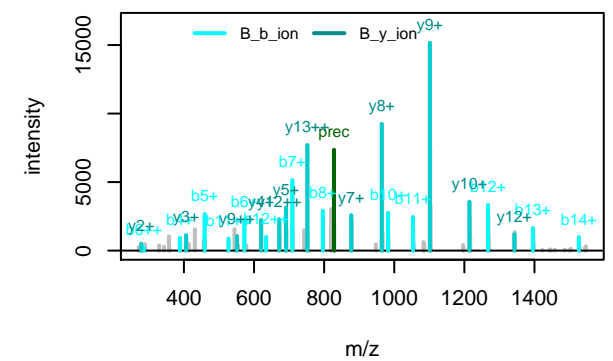

Supplement: Supplemental Data [file supp_RA117.000470_133922_0_supp_23978_fzffwf.zip › spectra_annotation/mito_DR_spectra_annotation/123-1-2-1-5-1.pdf]

APEDKKK  
SVISYKTEEK

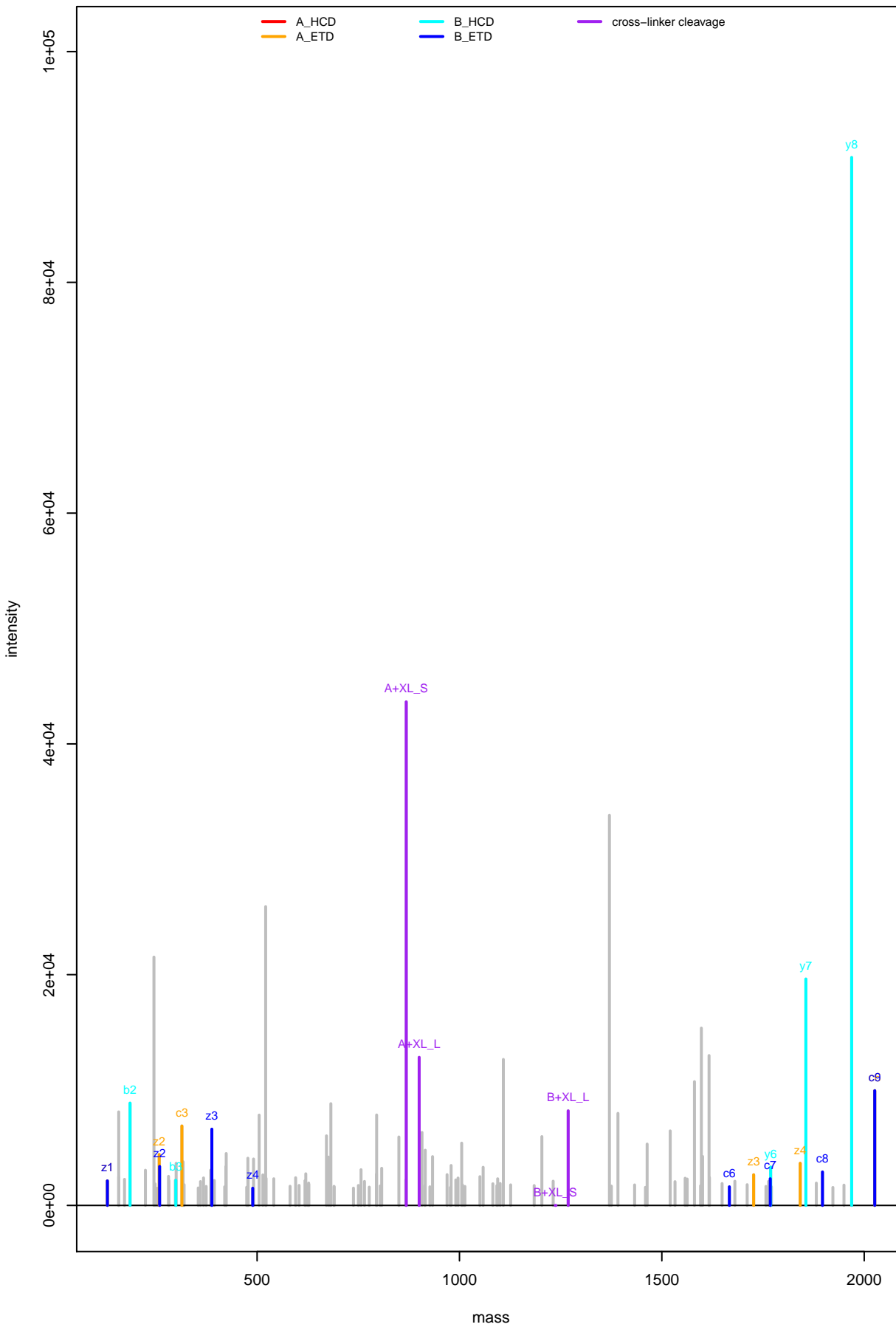

APEDKKK+XL\_S

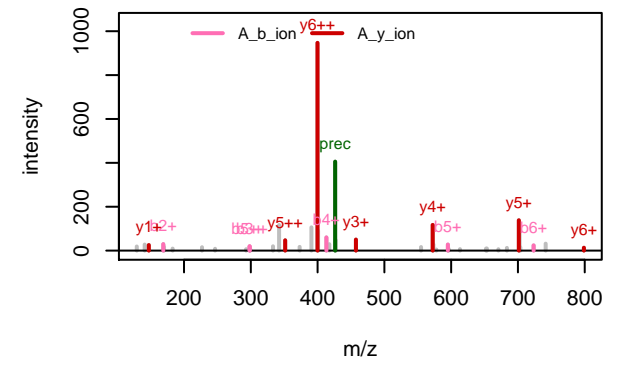

APEDKKK+XL\_L

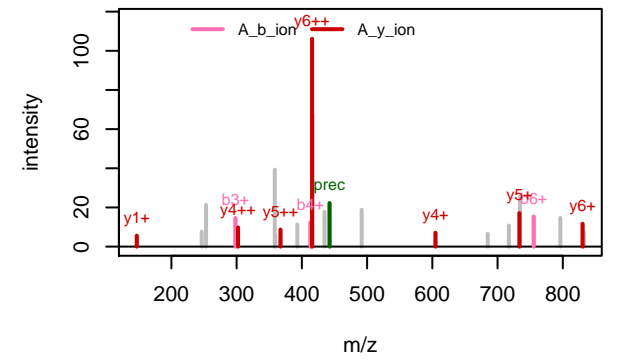

SVISYKTEEK+XL\_S

SVISYKTEEK+XL\_L

Supplement: Supplemental Data [file supp_RA117.000470_133922_0_supp_23978_fzffwf.zip › spectra_annotation/mito_DR_spectra_annotation/123-1-3-1-1-1.pdf]

LVPLKETIK  
RSTVAQLVKR

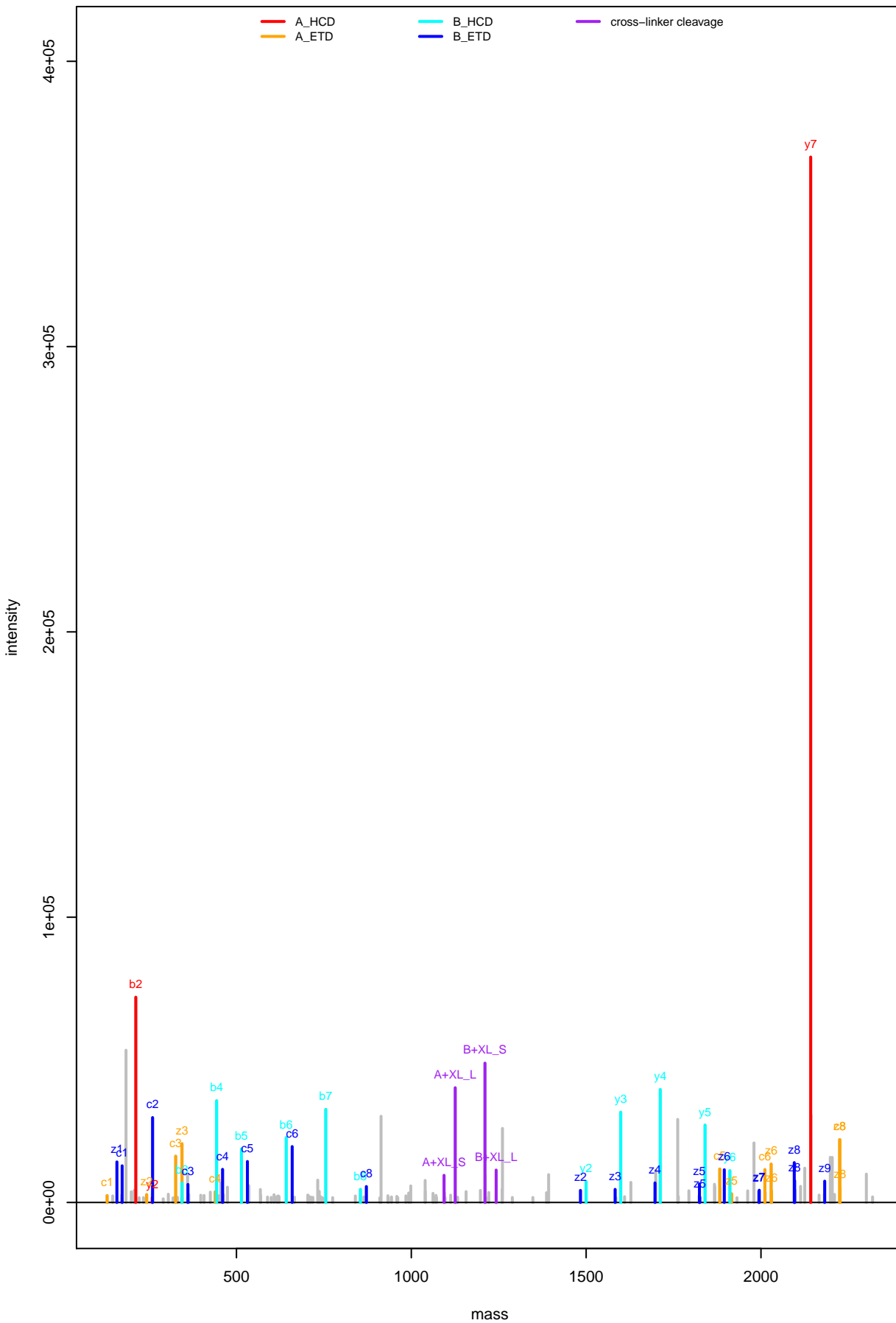

LVPLKETIK+XL\_S

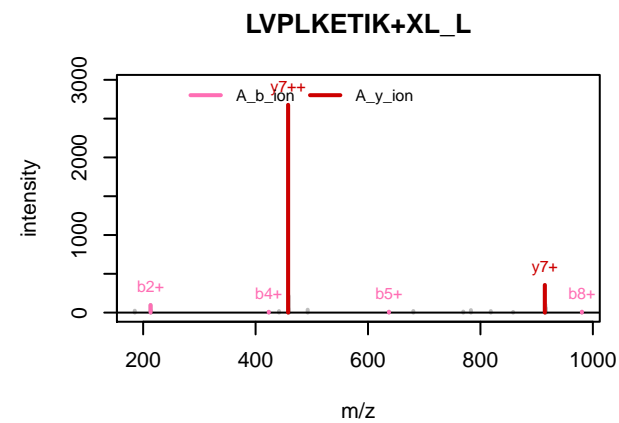

RSTVAQLVKR+XL\_S

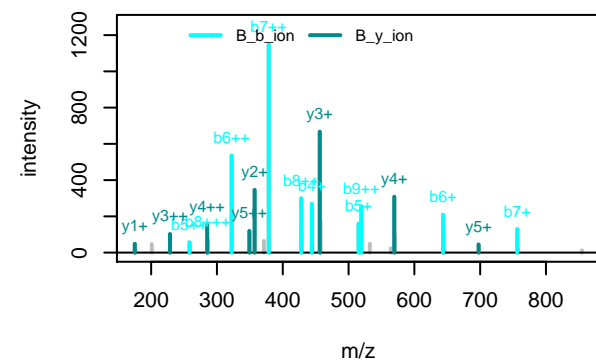

RSTVAQLVKR+XL\_L

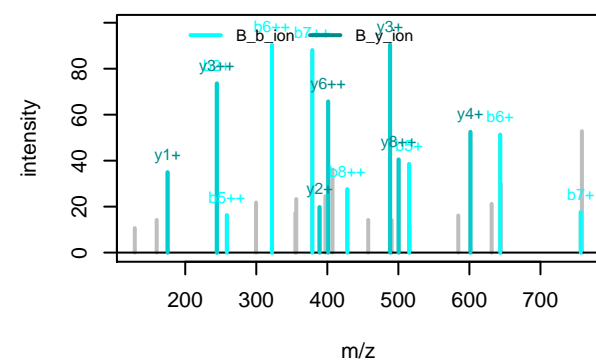

Supplement: Supplemental Data [file supp_RA117.000470_133922_0_supp_23978_fzffwf.zip › spectra_annotation/mito_DR_spectra_annotation/123-1-4-1-6-1.pdf]

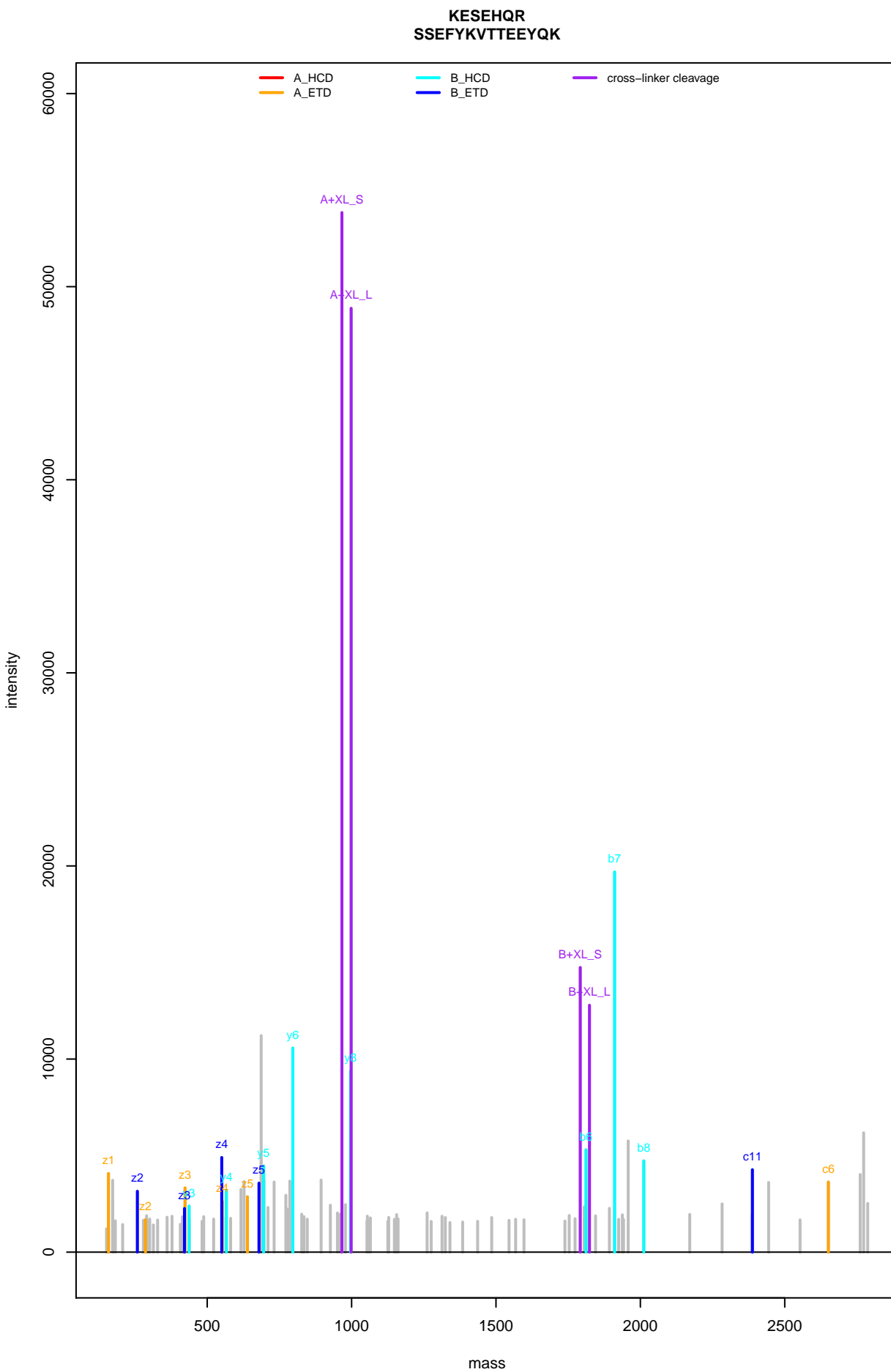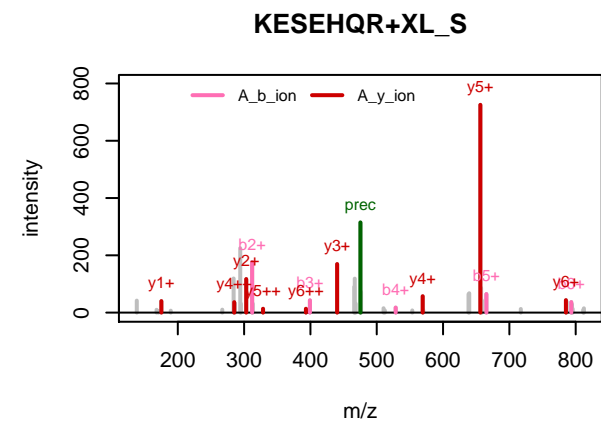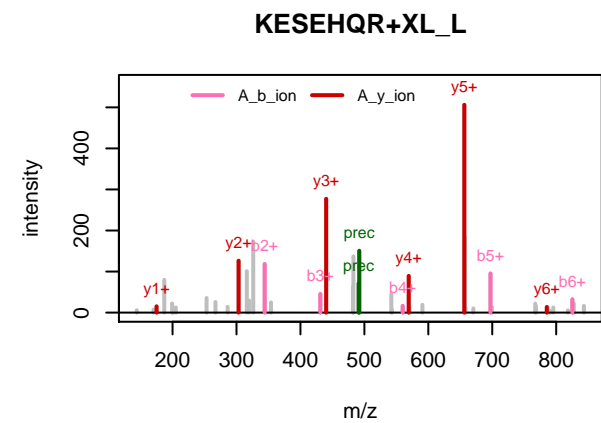

**SSEFYKVTTEEYQK+XL\_S**

**SSEFYKVTTEEYQK+XL\_L**

Supplement: Supplemental Data [file supp_RA117.000470_133922_0_supp_23978_fzffwf.zip › spectra_annotation/mito_DR_spectra_annotation/124-1-1-1-2-1.pdf]

VKSLNDITK  
YATALYSAASKEK

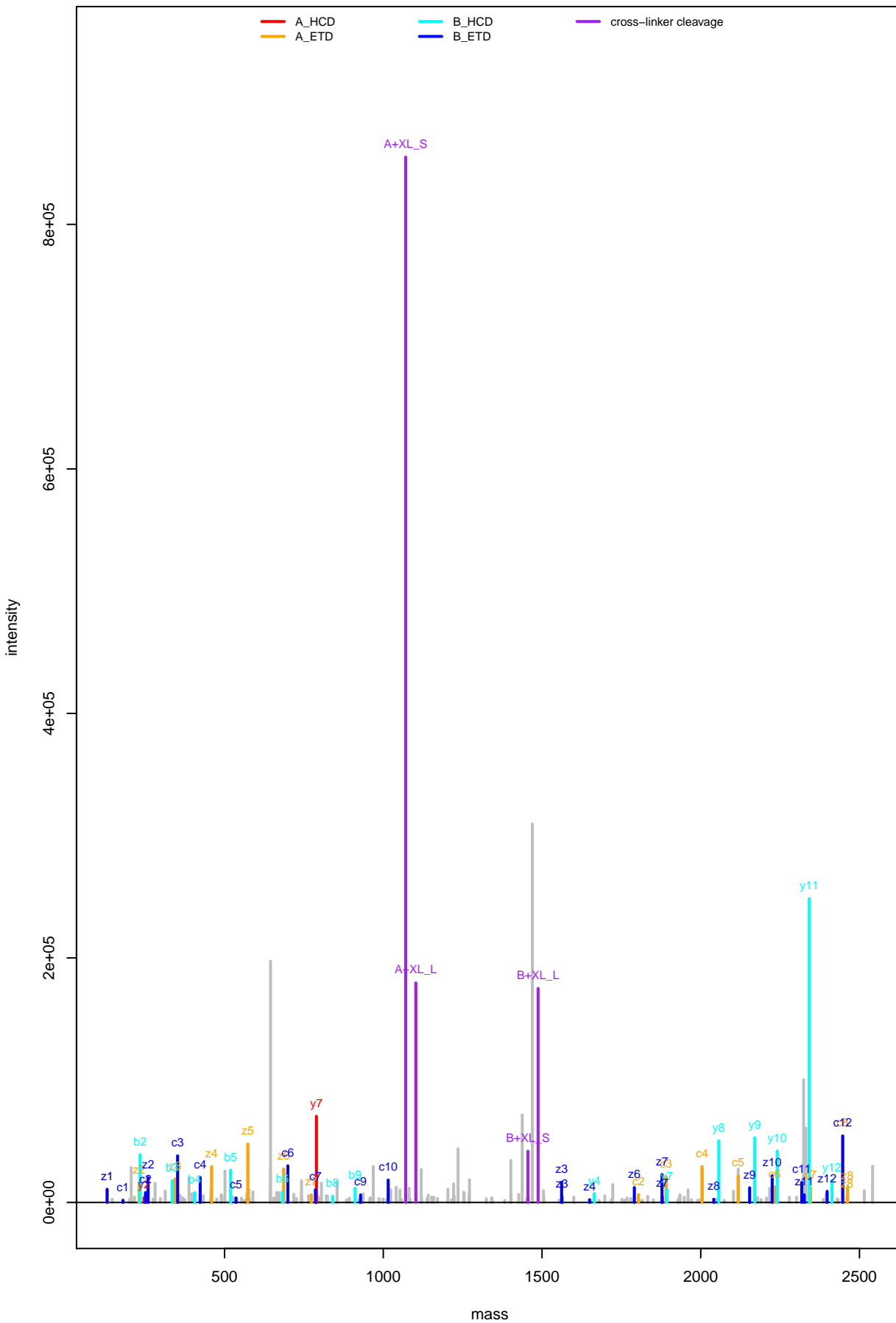

## VKSLNDITK+XL\_S

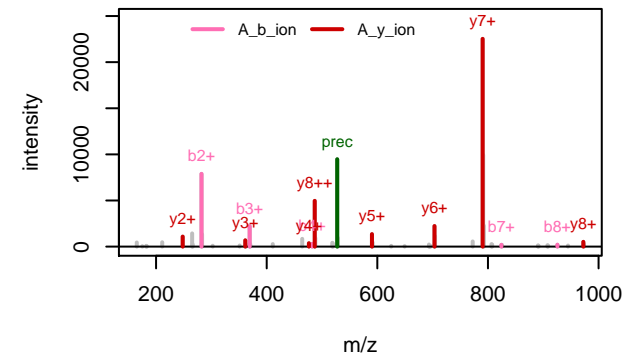

## VKSLNDITK+XL\_L

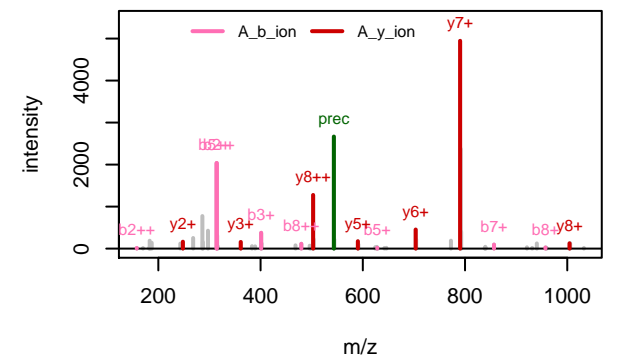

## YATALYSAASKEK+XL\_S

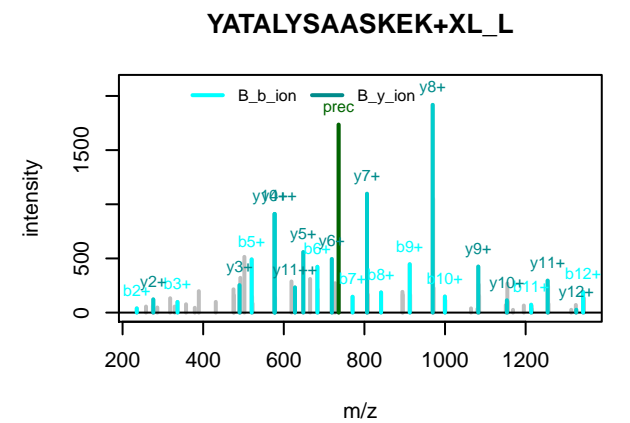

Supplement: Supplemental Data [file supp_RA117.000470_133922_0_supp_23978_fzffwf.zip › spectra_annotation/mito_DR_spectra_annotation/124-1-15-1-11-1.pdf]

LEPSKITK  
LEPSKITK

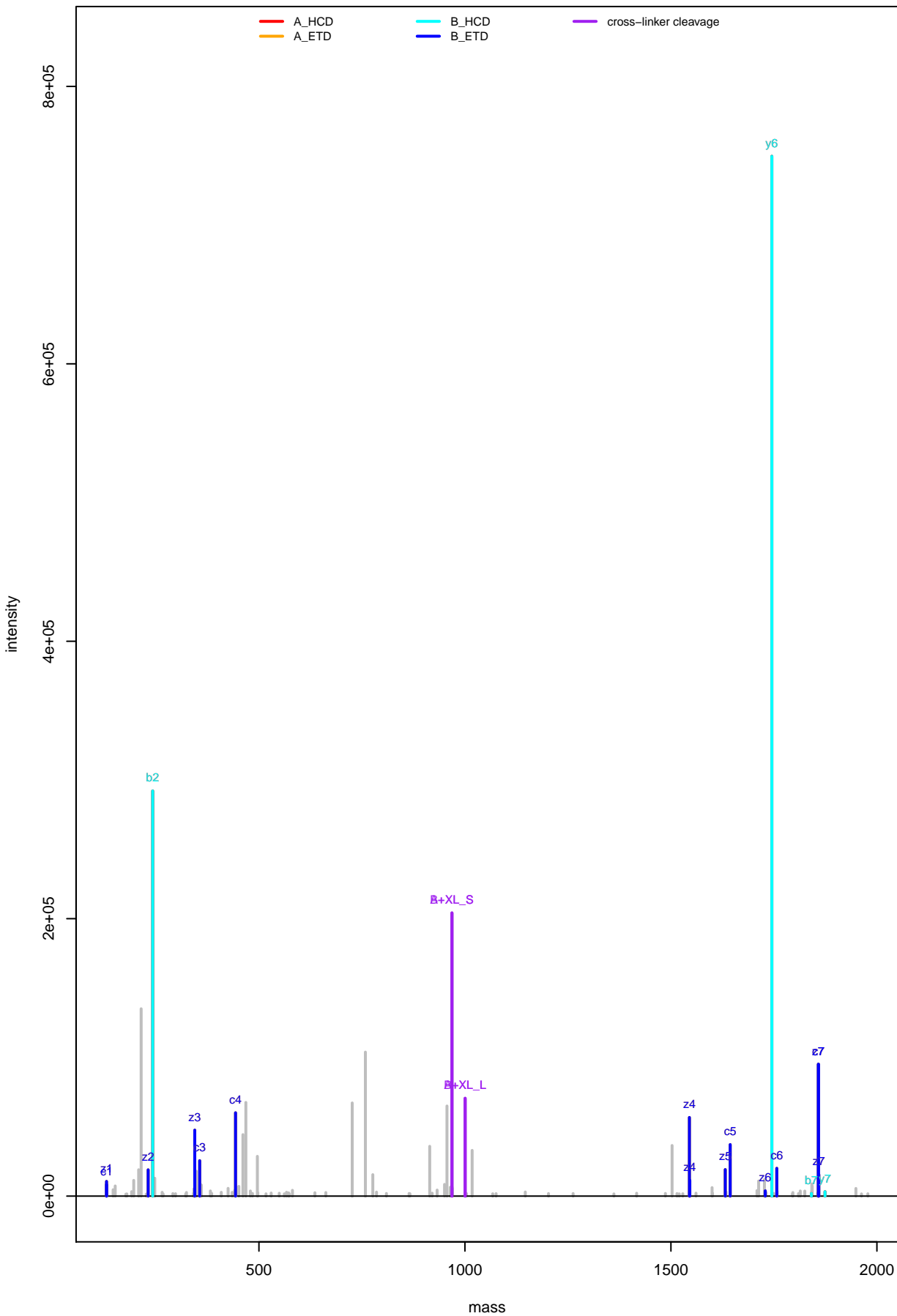

LEPSKITK+XL\_S

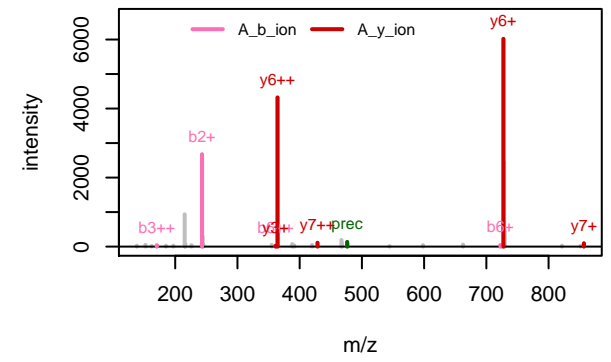

LEPSKITK+XL\_L

LEPSKITK+XL\_S

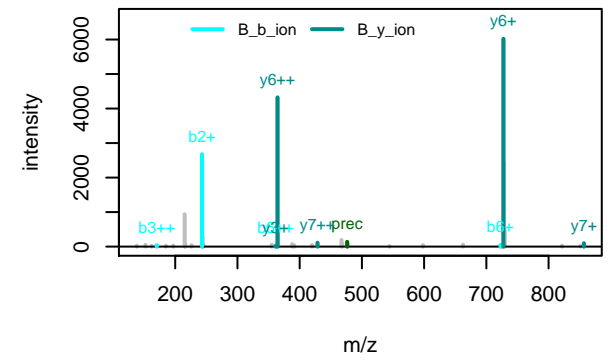

LEPSKITK+XL\_L

Supplement: Supplemental Data [file supp_RA117.000470_133922_0_supp_23978_fzffwf.zip › spectra_annotation/mito_DR_spectra_annotation/124-1-20-1-20-1.pdf]

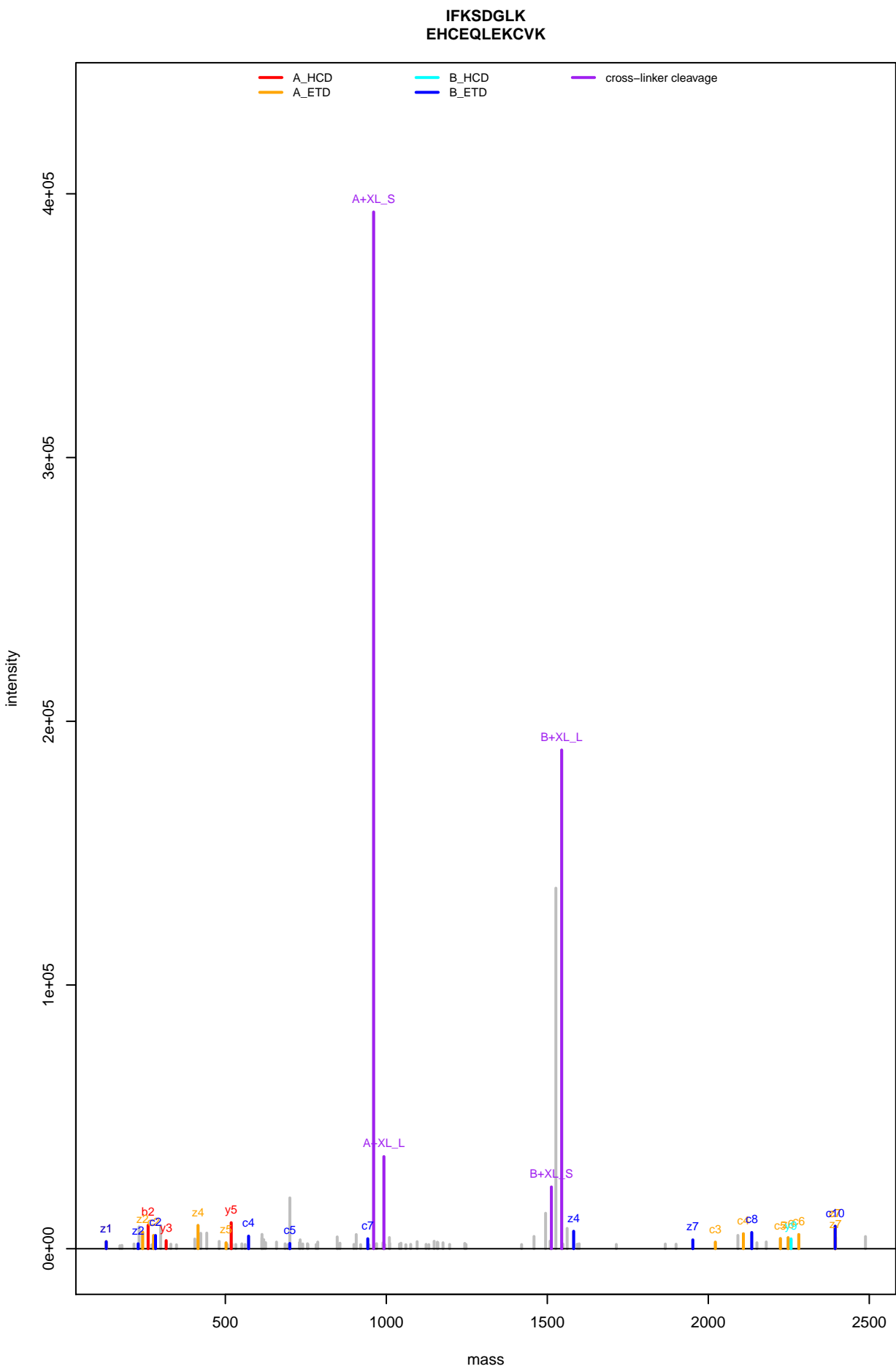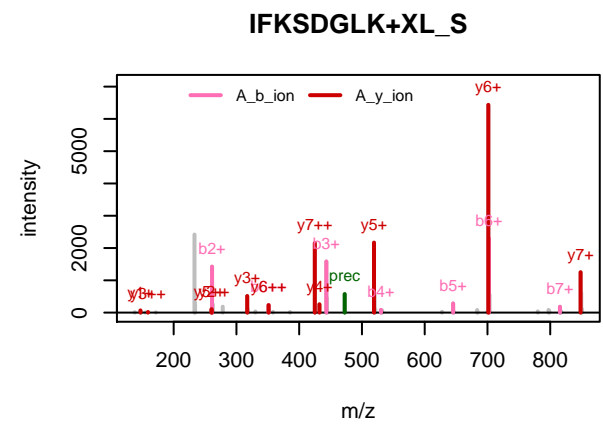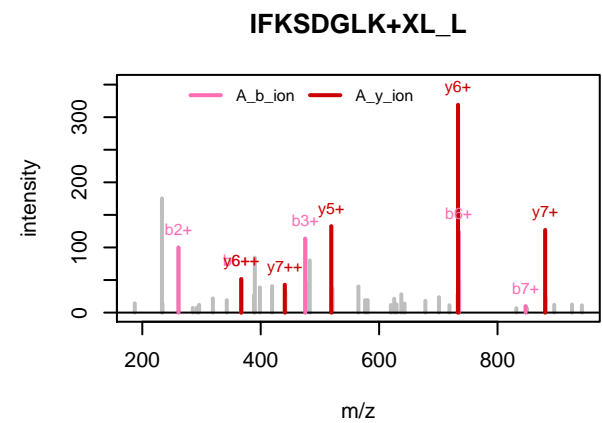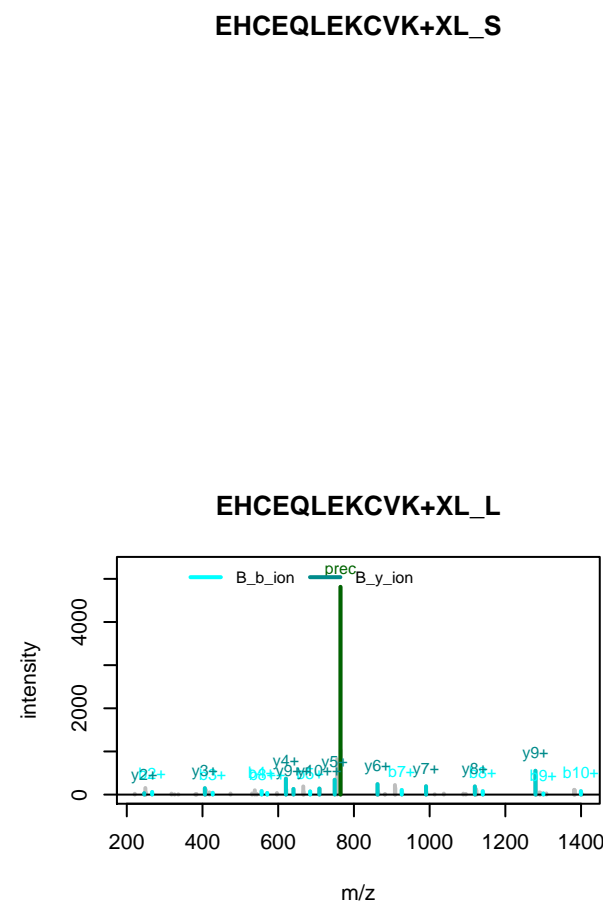

Supplement: Supplemental Data [file supp_RA117.000470_133922_0_supp_23978_fzffwf.zip › spectra_annotation/mito_DR_spectra_annotation/125-1-12-1-1-1.pdf]

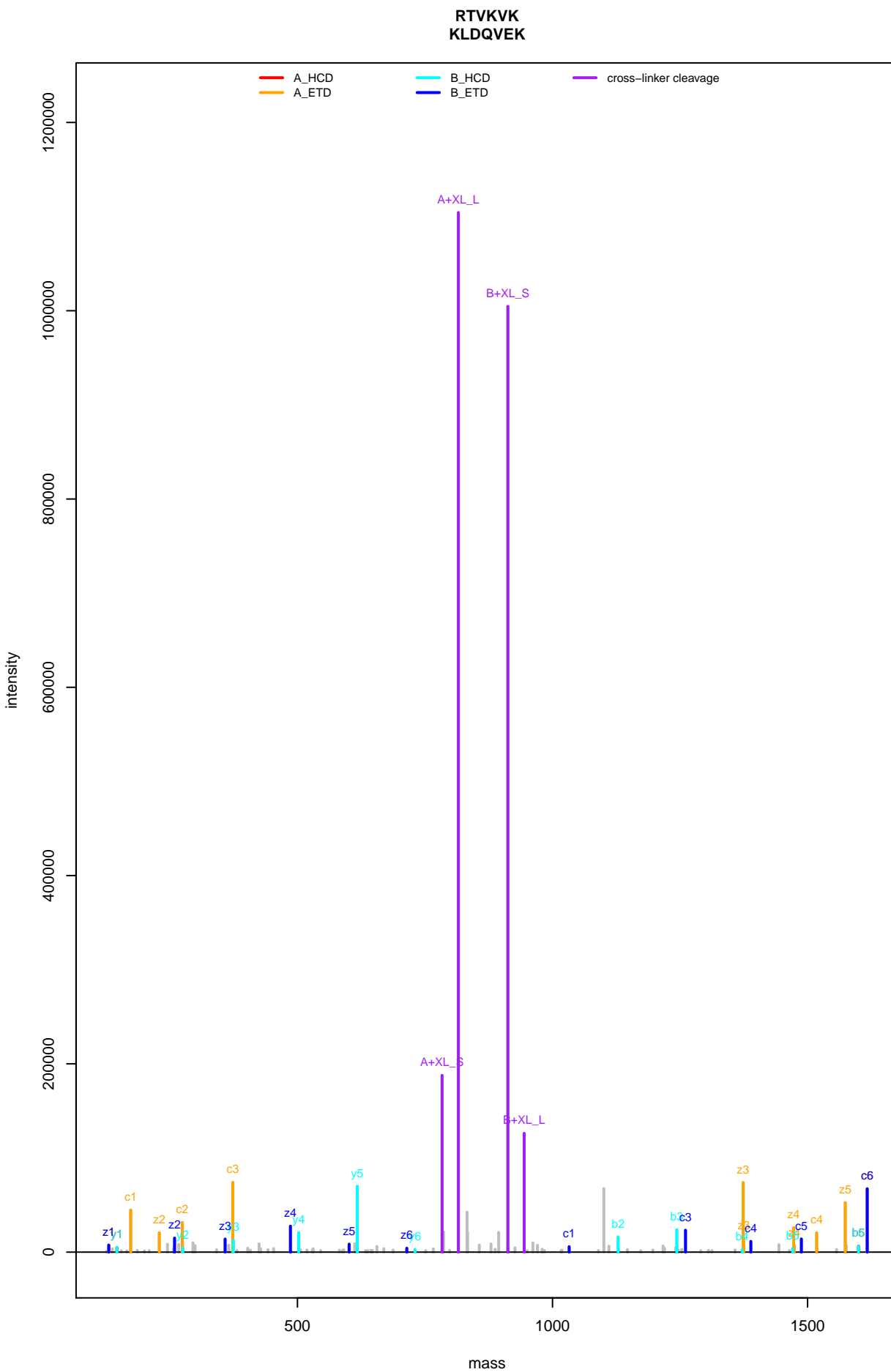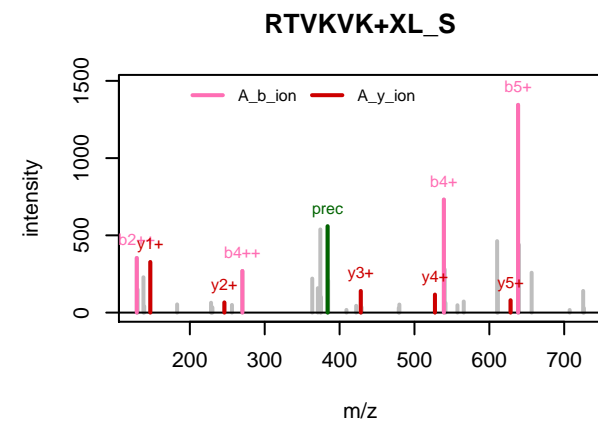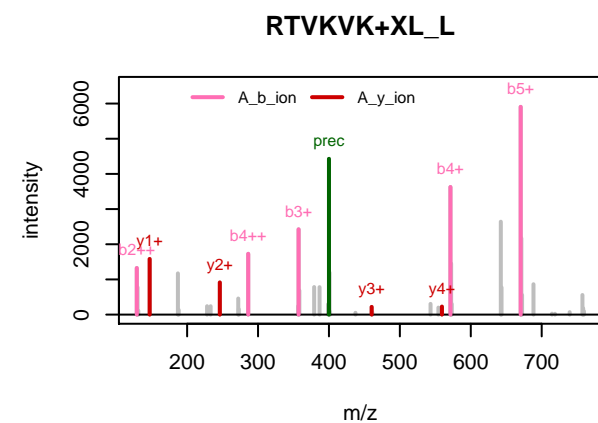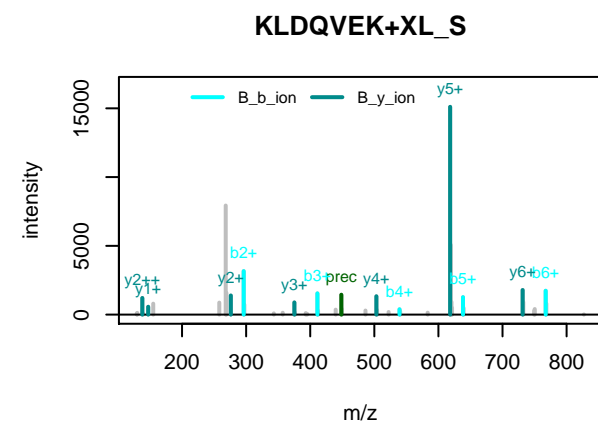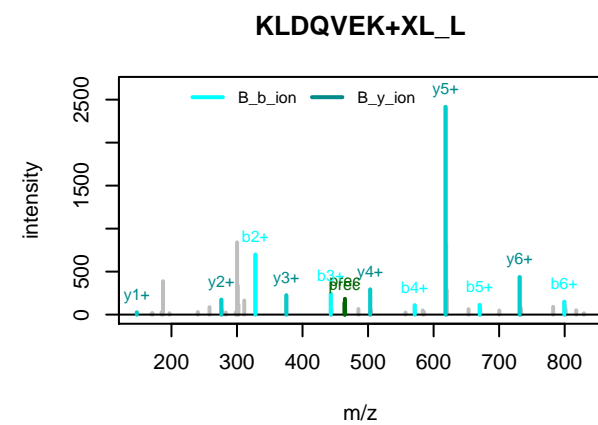

Supplement: Supplemental Data [file supp_RA117.000470_133922_0_supp_23978_fzffwf.zip › spectra_annotation/mito_DR_spectra_annotation/125-1-15-1-19-1.pdf]

NAEKYAEEDRR  
QATKDAGQISGLNVLR

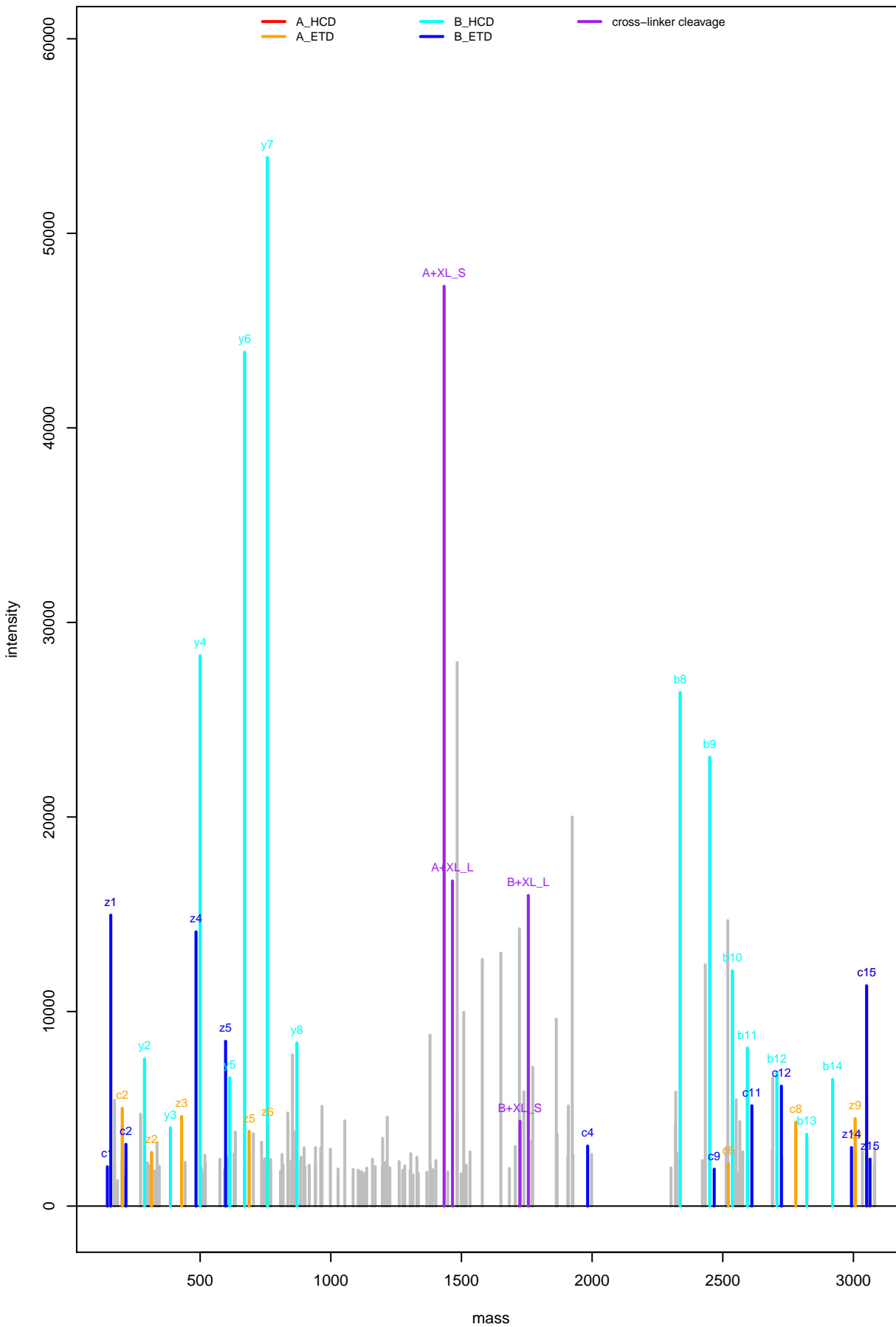

NAEKYAEEDRR+XL\_S

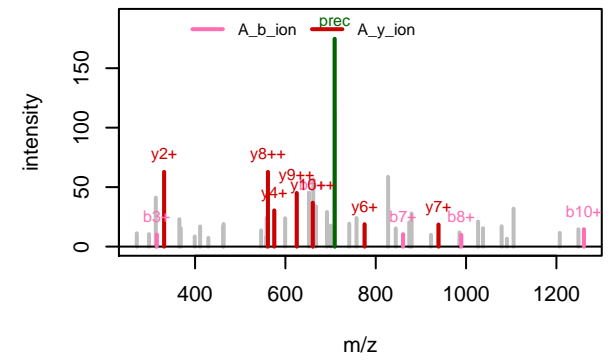

NAEKYAEEDRR+XL\_L

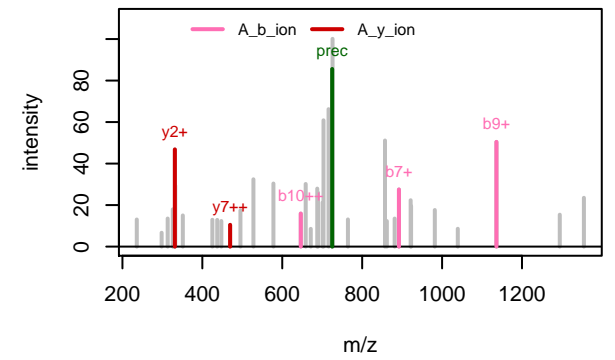

QATKDAGQISGLNVLR+XL\_S

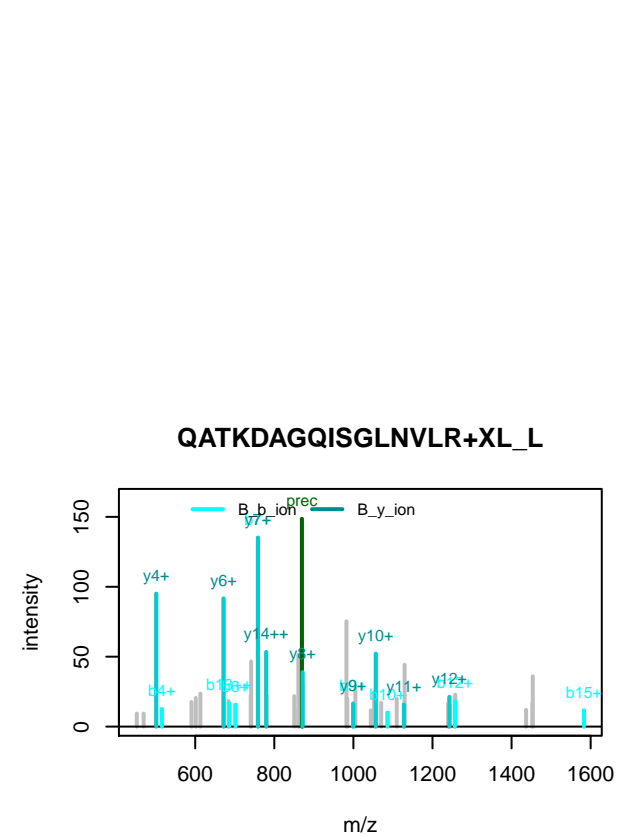

Supplement: Supplemental Data [file supp_RA117.000470_133922_0_supp_23978_fzffwf.zip › spectra_annotation/mito_DR_spectra_annotation/125-1-8-1-28-1.pdf]

IATKDRK  
TSKDTTASAVAVGLR

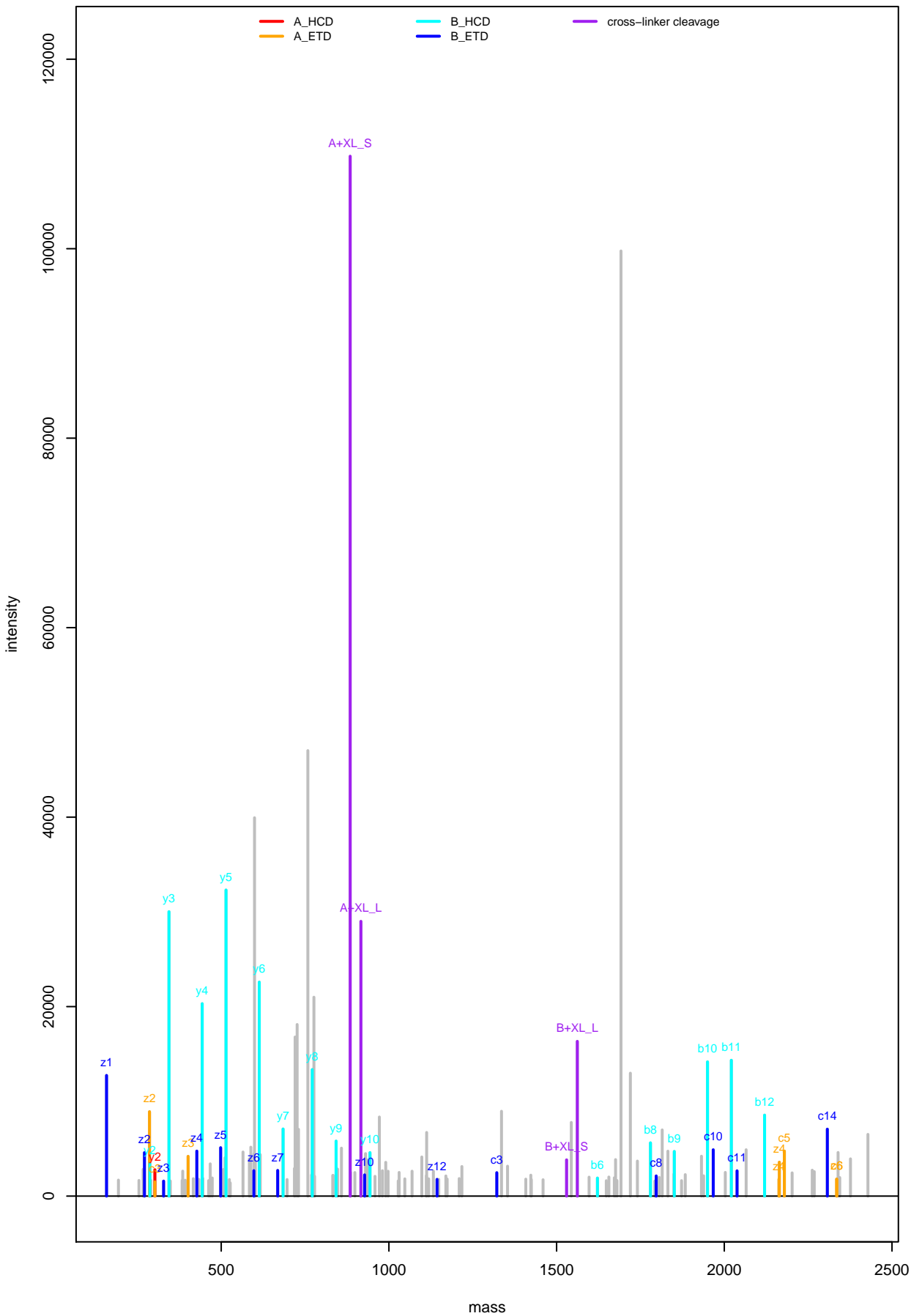

IATKDRK+XL\_S

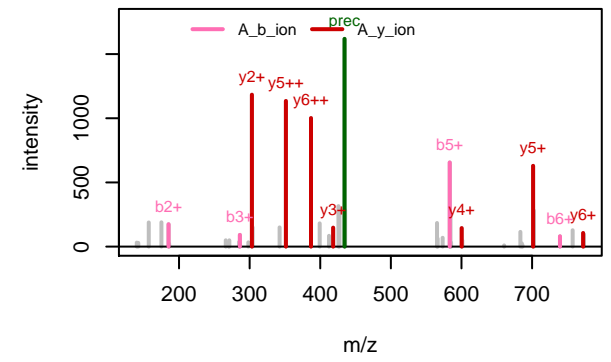

IATKDRK+XL\_L

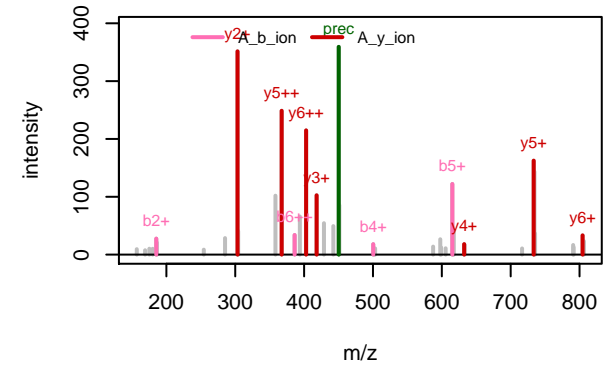

TSKDTTASAVAVGLR+XL\_S

TSKDTTASAVAVGLR+XL\_L

Supplement: Supplemental Data [file supp_RA117.000470_133922_0_supp_23978_fzffwf.zip › spectra_annotation/mito_DR_spectra_annotation/126-1-4-1-8-1.pdf]

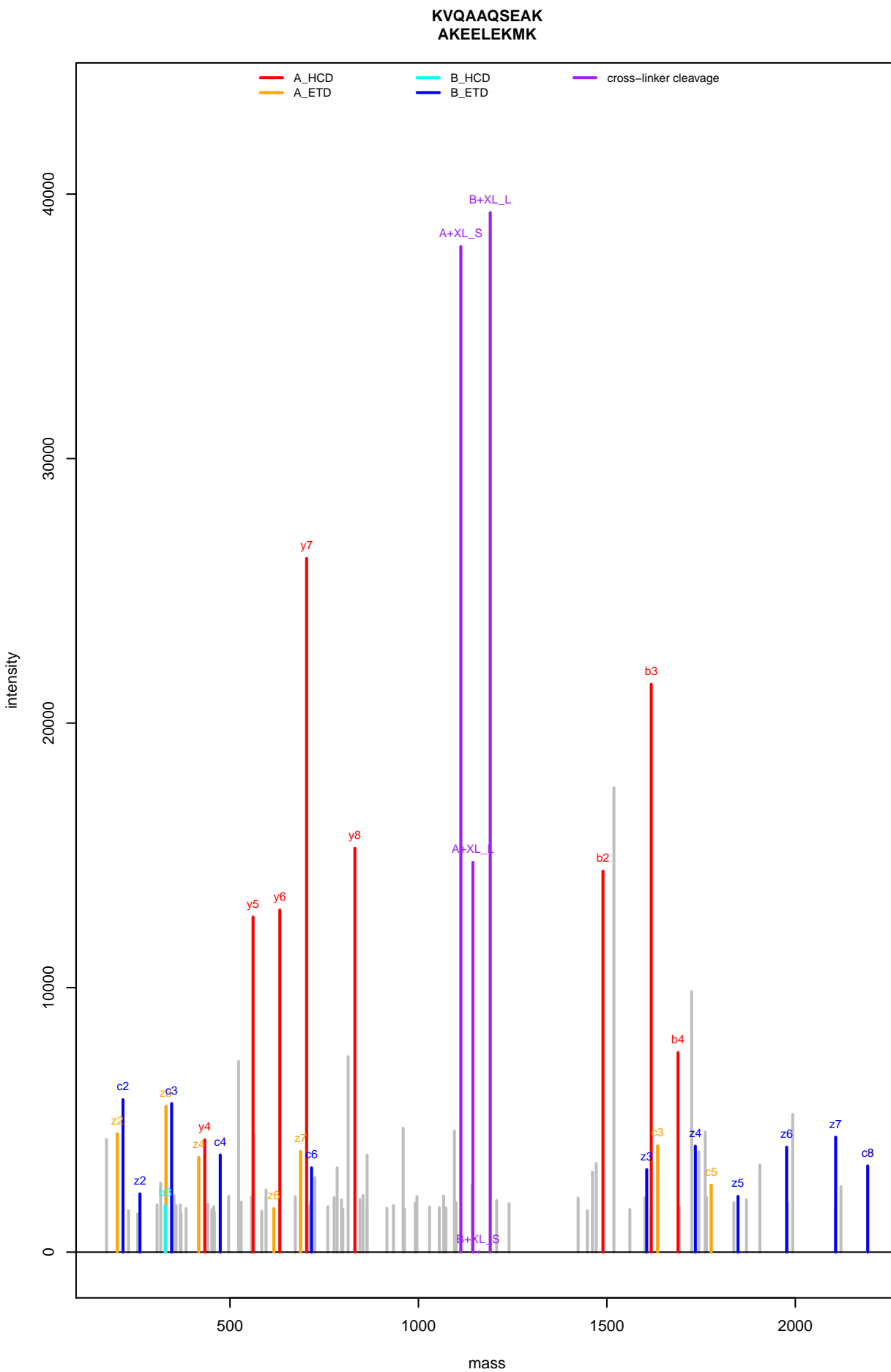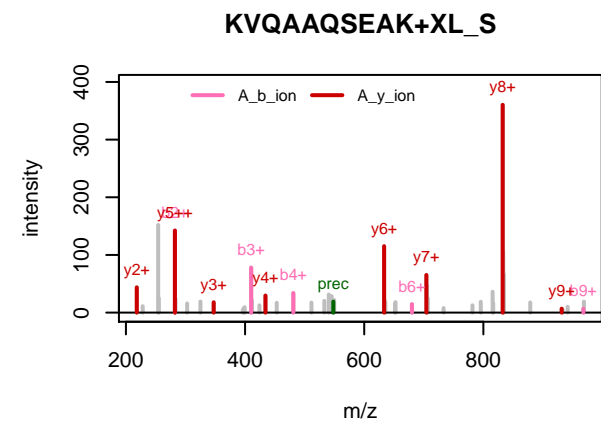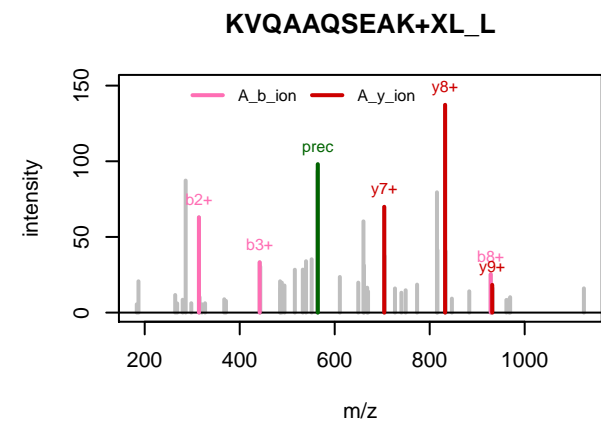

**AKEELEKMK+XL\_S**

**AKEELEKMK+XL\_L**

Supplement: Supplemental Data [file supp_RA117.000470_133922_0_supp_23978_fzffwf.zip › spectra_annotation/mito_DR_spectra_annotation/126-1-7-1-6-1.pdf]

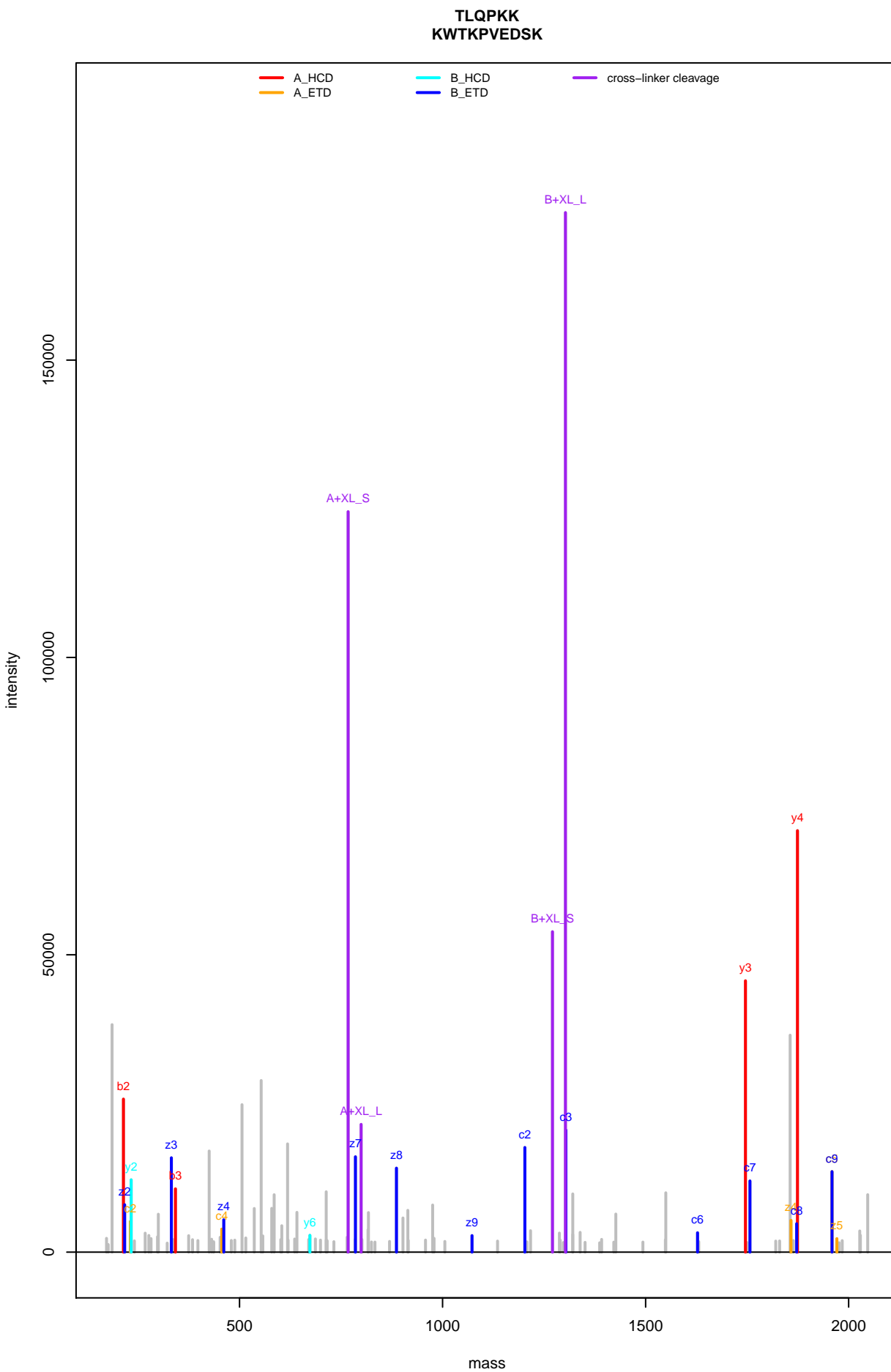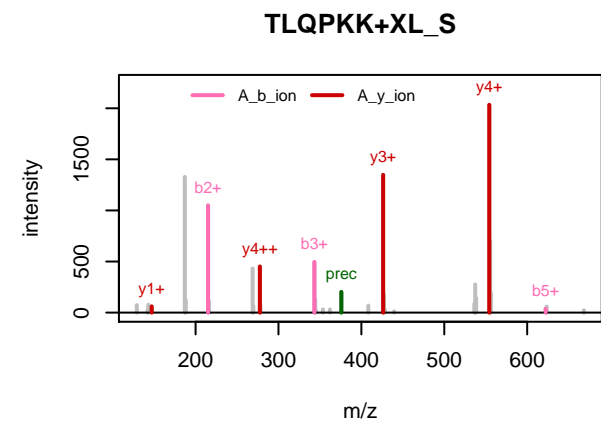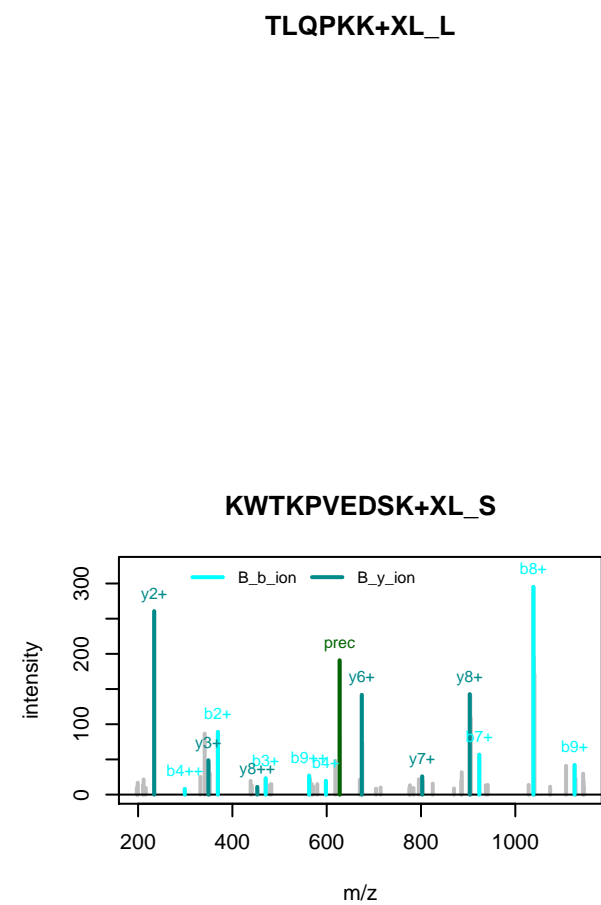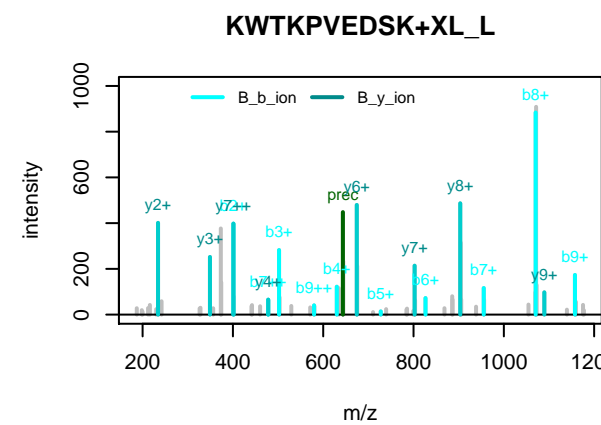

Supplement: Supplemental Data [file supp_RA117.000470_133922_0_supp_23978_fzffwf.zip › spectra_annotation/mito_DR_spectra_annotation/127-1-1-1-23-1.pdf]

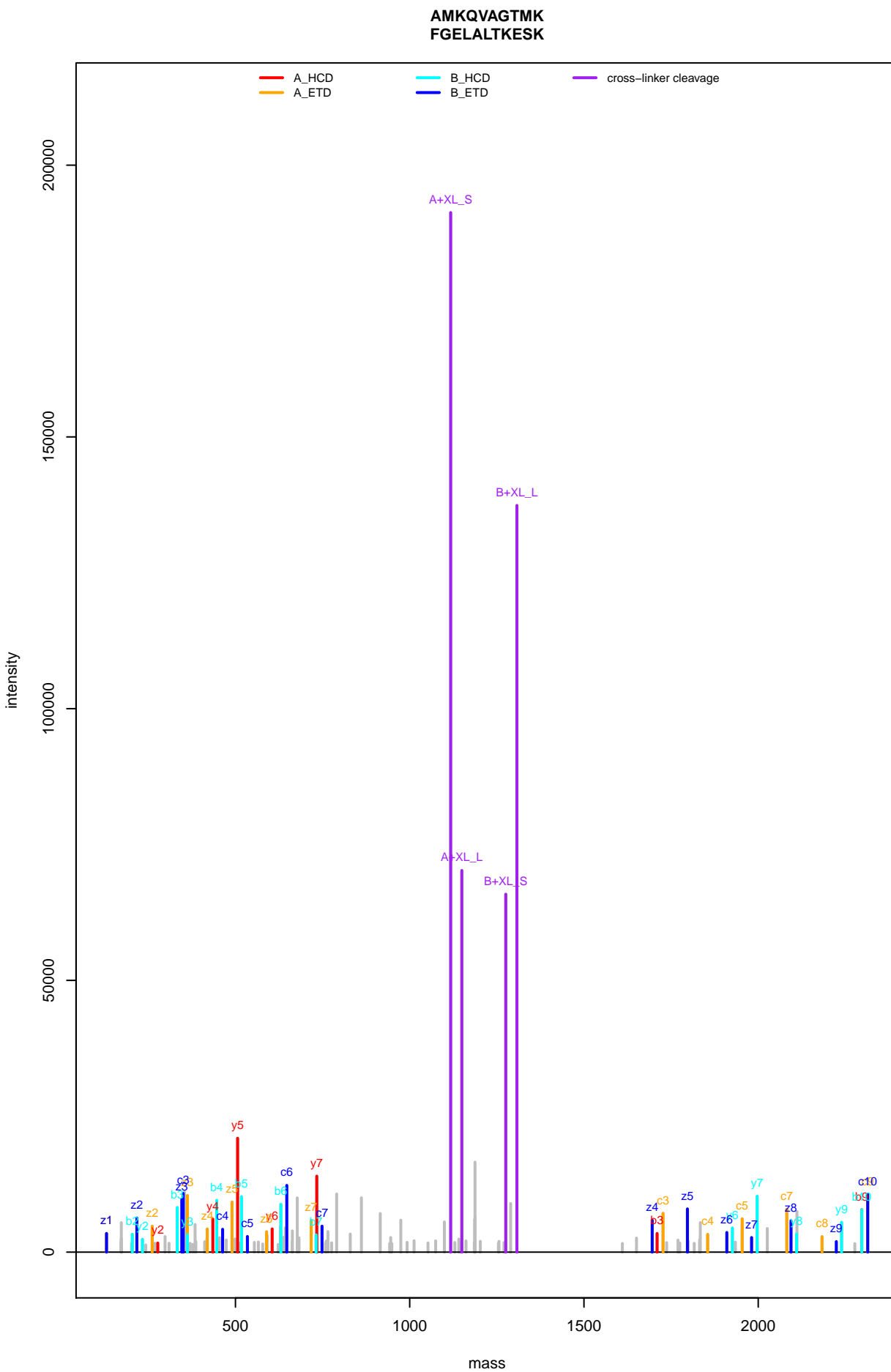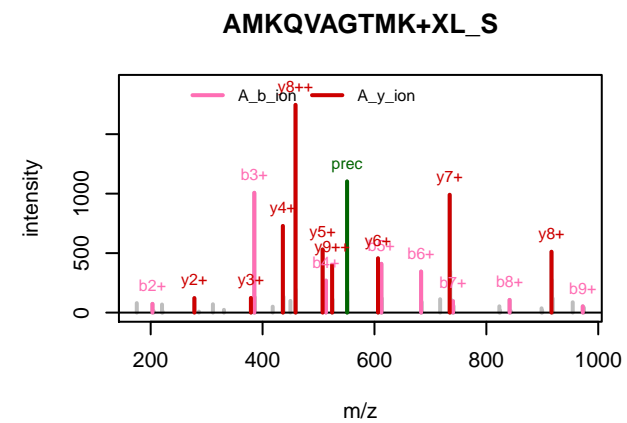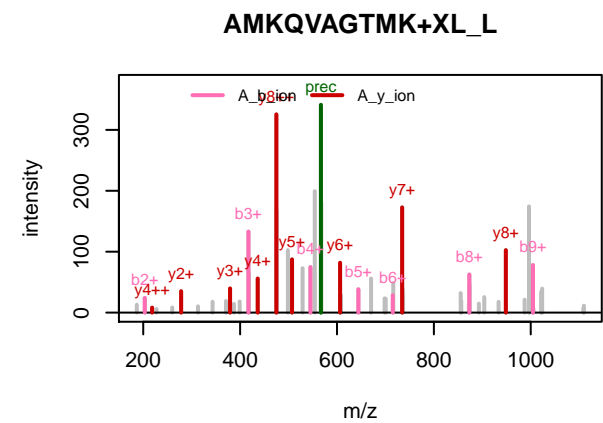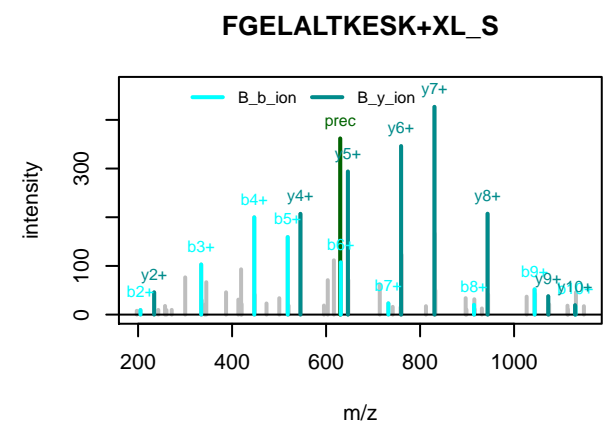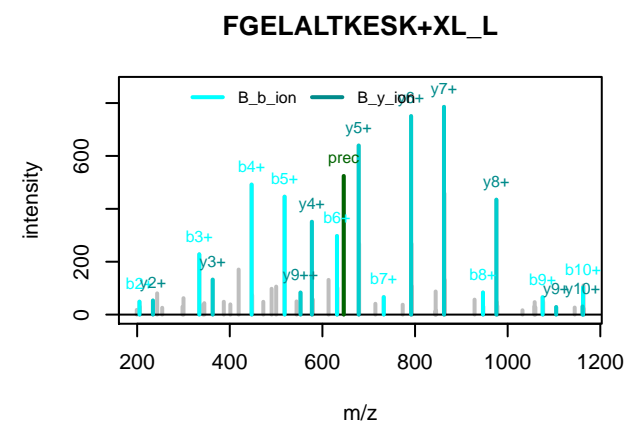

Supplement: Supplemental Data [file supp_RA117.000470_133922_0_supp_23978_fzffwf.zip › spectra_annotation/mito_DR_spectra_annotation/127-1-11-1-11-1.pdf]

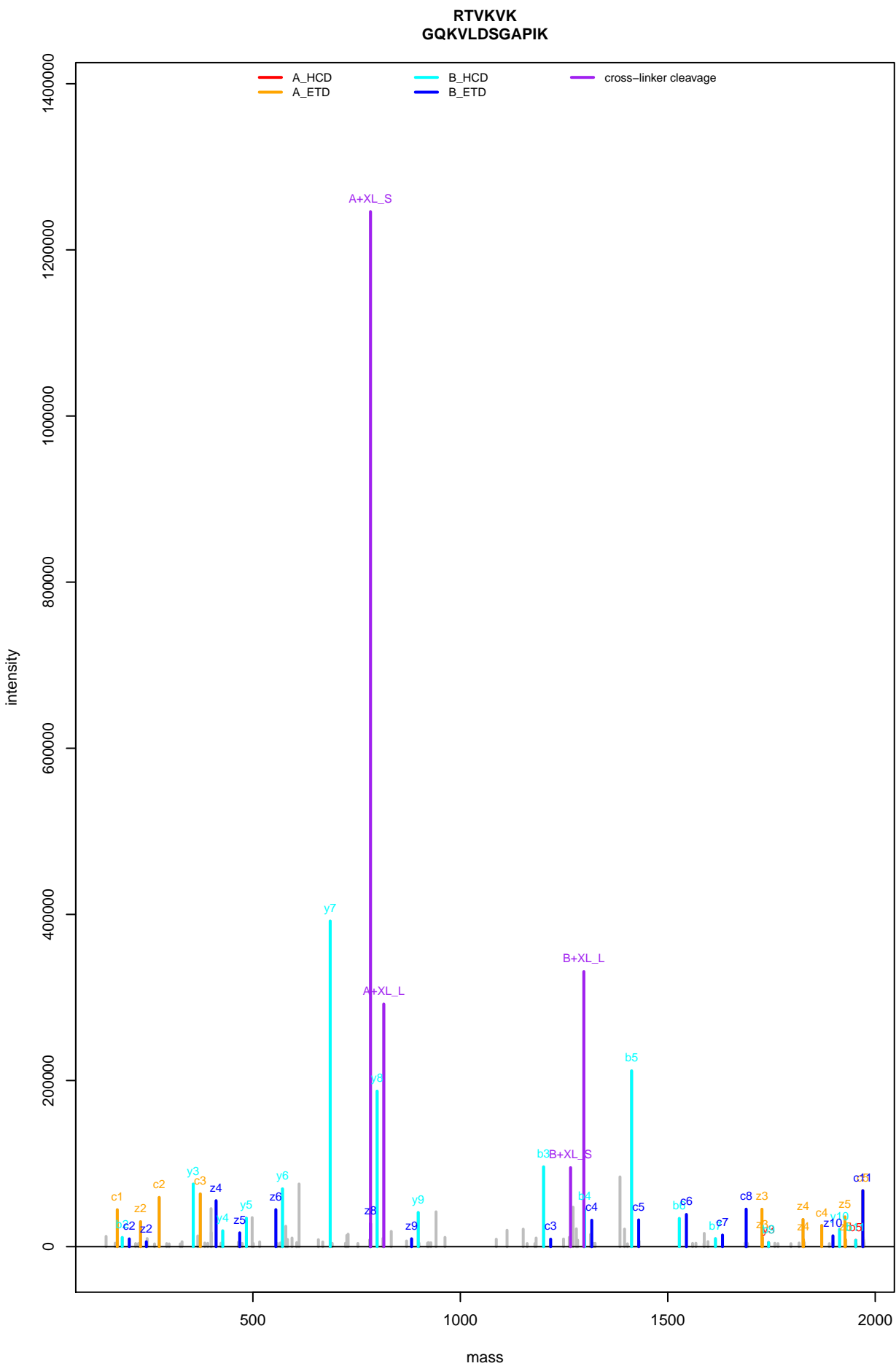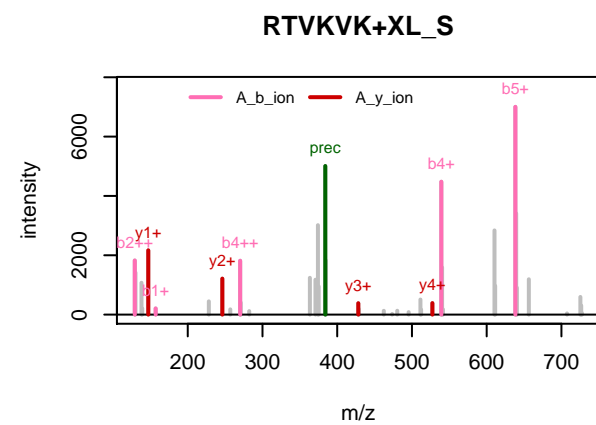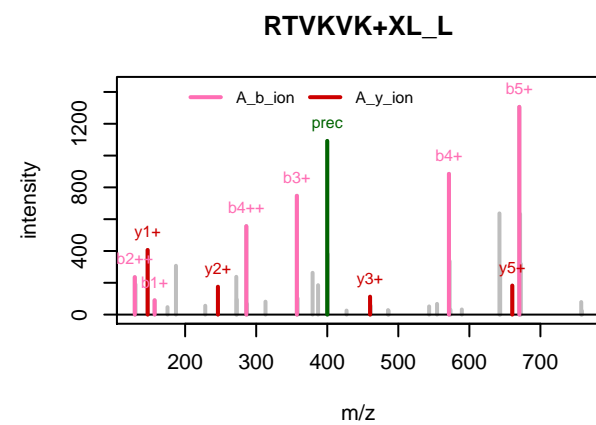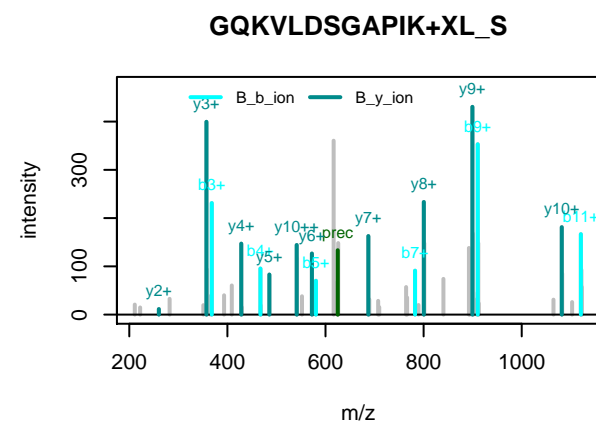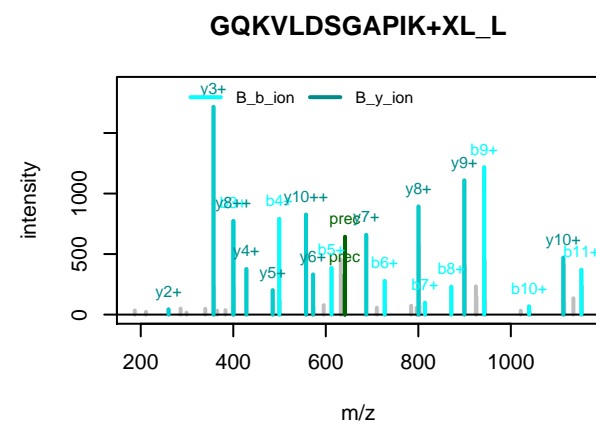

Supplement: Supplemental Data [file supp_RA117.000470_133922_0_supp_23978_fzffwf.zip › spectra_annotation/mito_DR_spectra_annotation/127-1-15-1-1-1.pdf]

GENAKK  
FVRTDEKVER

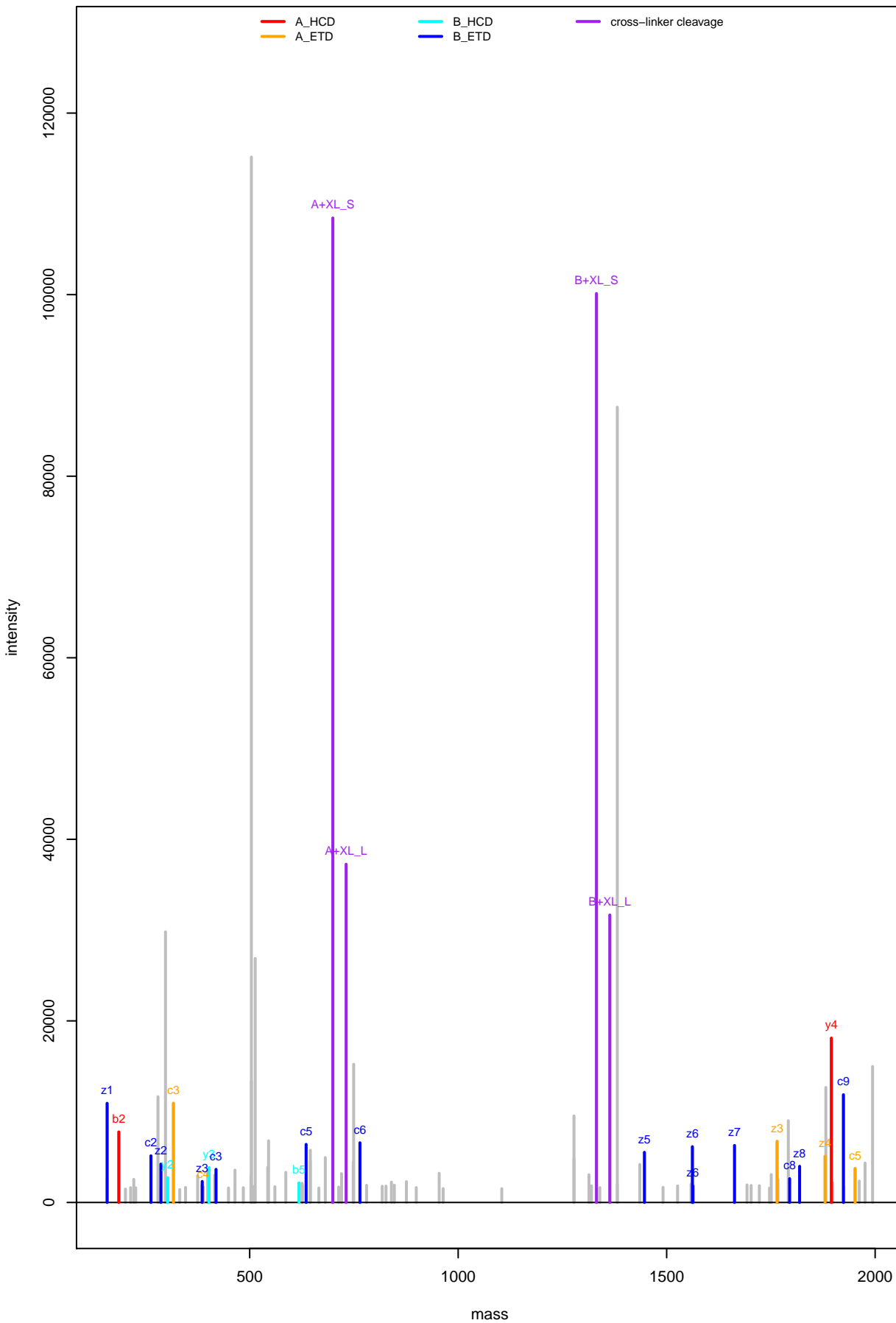

GENAKK+XL\_S

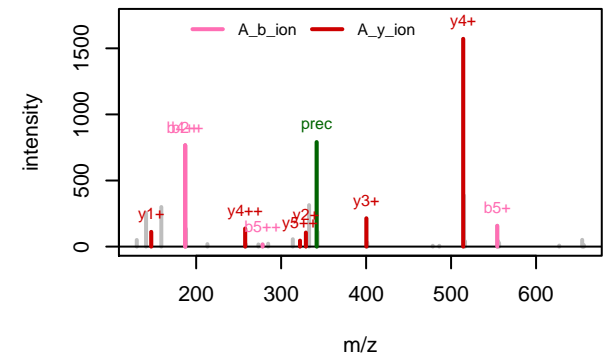

GENAKK+XL\_L

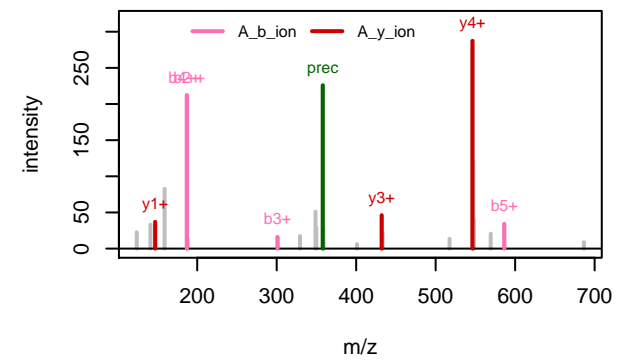

FVRTDEKVER+XL\_S

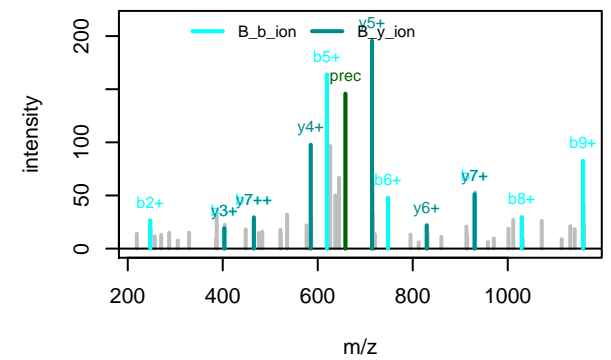

FVRTDEKVER+XL\_L

Supplement: Supplemental Data [file supp_RA117.000470_133922_0_supp_23978_fzffwf.zip › spectra_annotation/mito_DR_spectra_annotation/127-1-2-1-15-1.pdf]

SIKNIQK  
NASDMIDKLTLTfNR

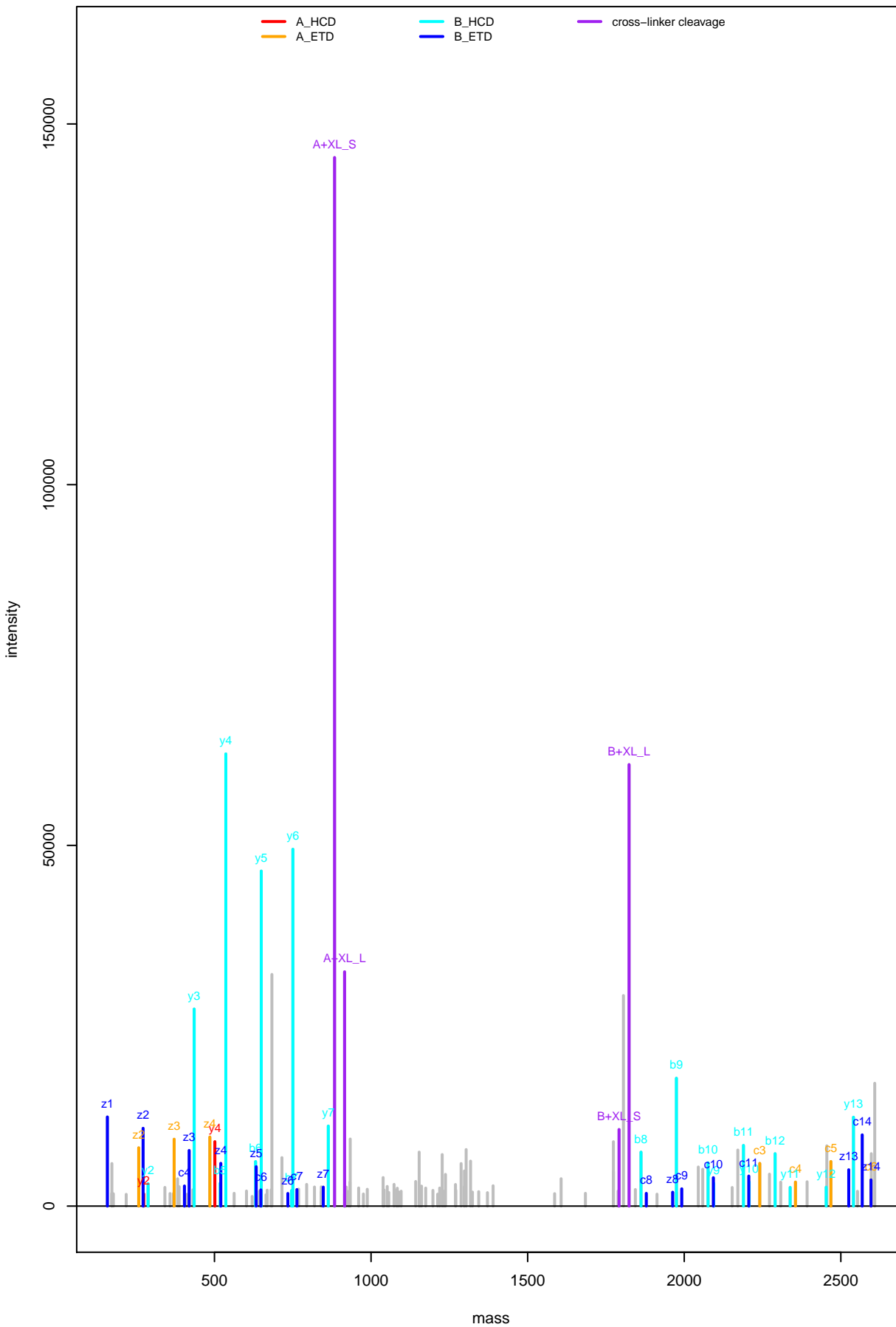

SIKNIQK+XL\_S

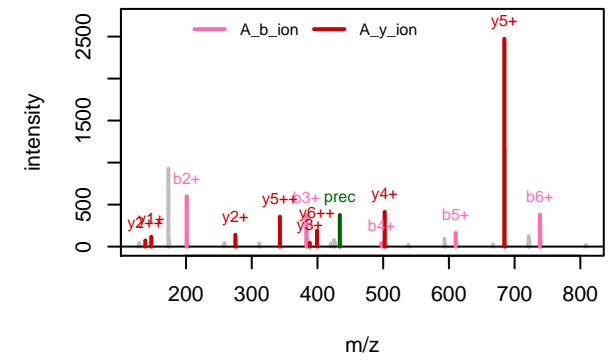

SIKNIQK+XL\_L

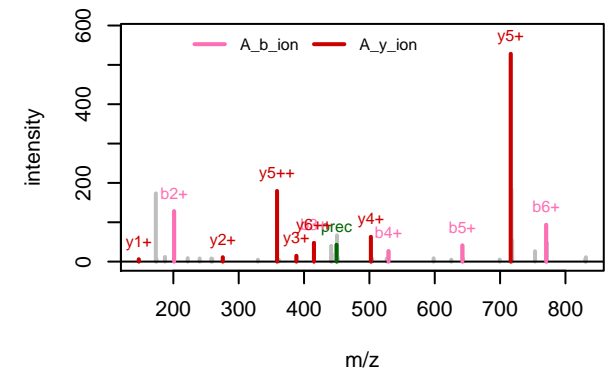

NASDMIDKLTLTfNR+XL\_S

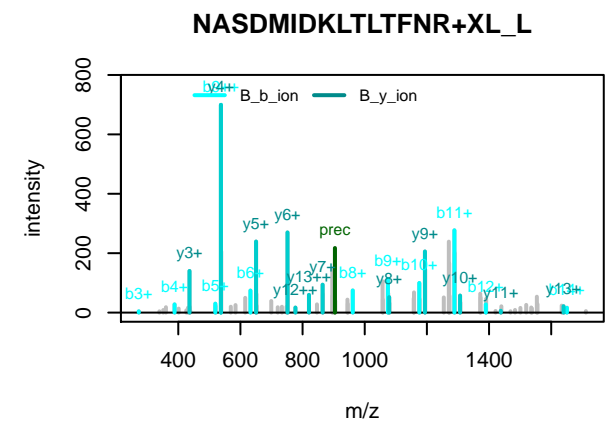

Supplement: Supplemental Data [file supp_RA117.000470_133922_0_supp_23978_fzffwf.zip › spectra_annotation/mito_DR_spectra_annotation/127-1-2-1-4-1.pdf]

**FLEGR**  
**ASQTMKCVK**

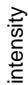

**FLEGKR+XL\_S**

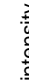

**FLEGKR+XL\_L**

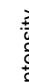

## ASQTMAKCVK+XL\_S

## ASQTMAKCVK+XL\_L

Supplement: Supplemental Data [file supp_RA117.000470_133922_0_supp_23978_fzffwf.zip › spectra_annotation/mito_DR_spectra_annotation/127-1-2-1-6-1.pdf]

**MFEKLEK**  
**RSTVAQLVKR**

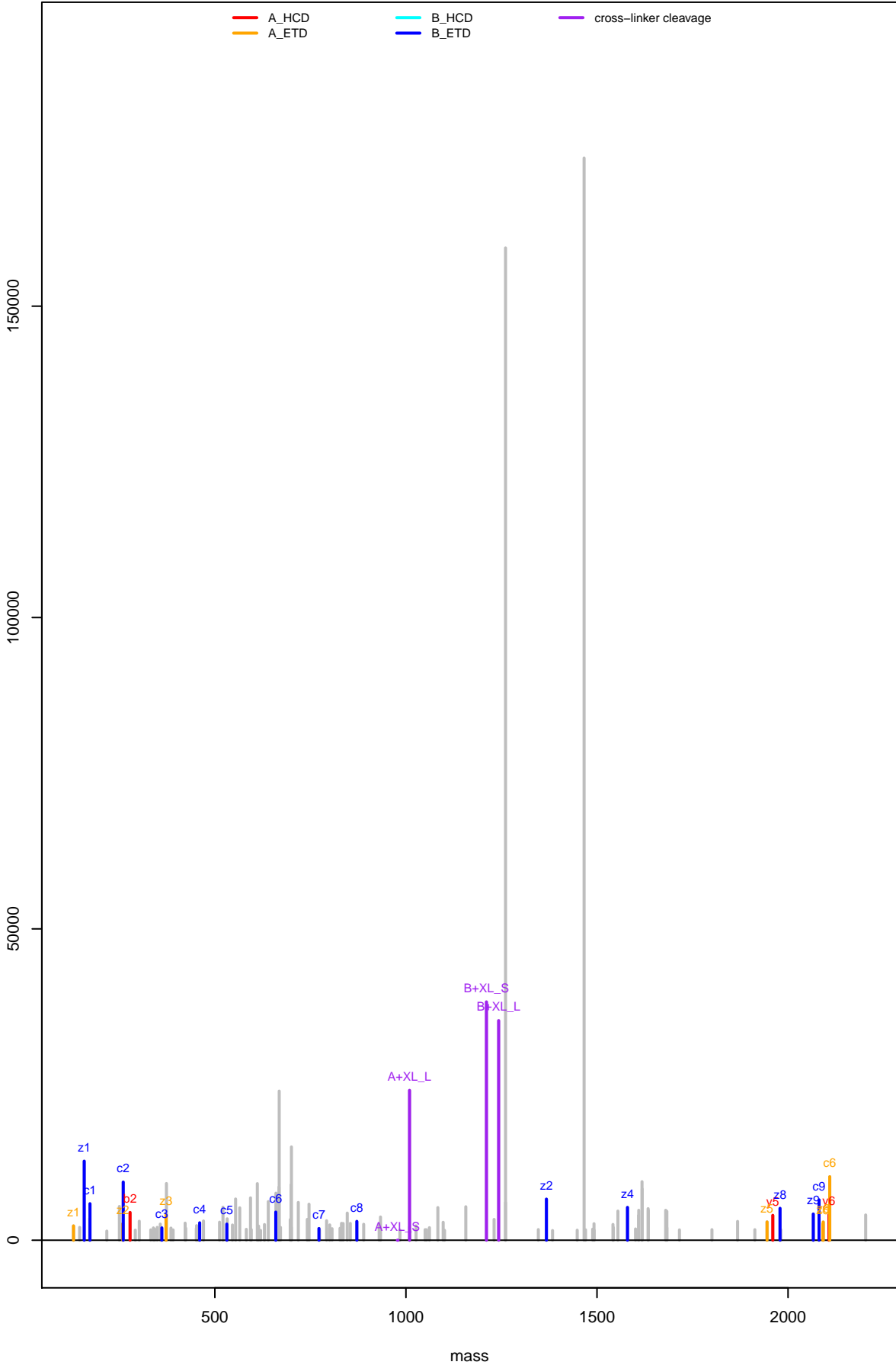

## MFEKLEK+XL\_S

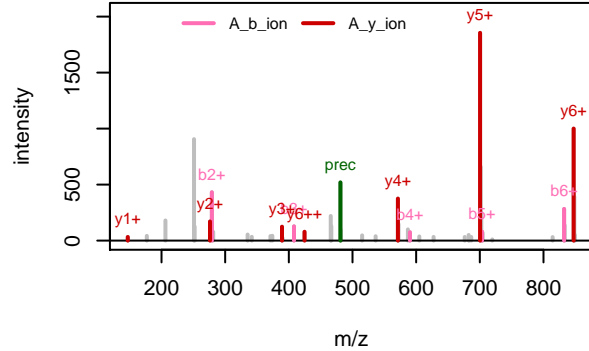

## MFEKLEK+XL\_L

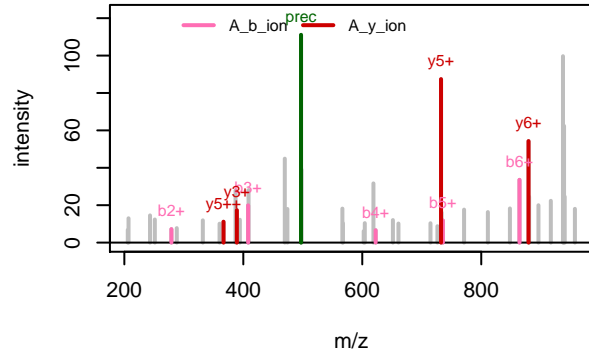

**RSTVAQLVKR+XL\_S**

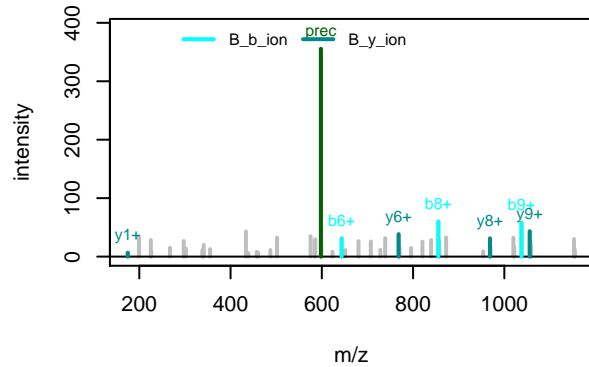

## RSTVAQLVKR+XL\_L

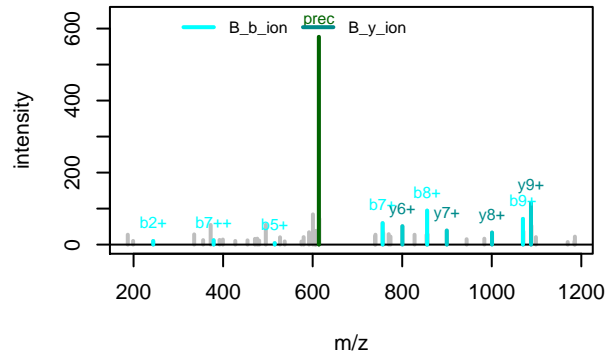

Supplement: Supplemental Data [file supp_RA117.000470_133922_0_supp_23978_fzffwf.zip › spectra_annotation/mito_DR_spectra_annotation/127-1-3-1-7-1.pdf]

**NKFGAPQK**  
**ALMGLYNGQVLCKK**

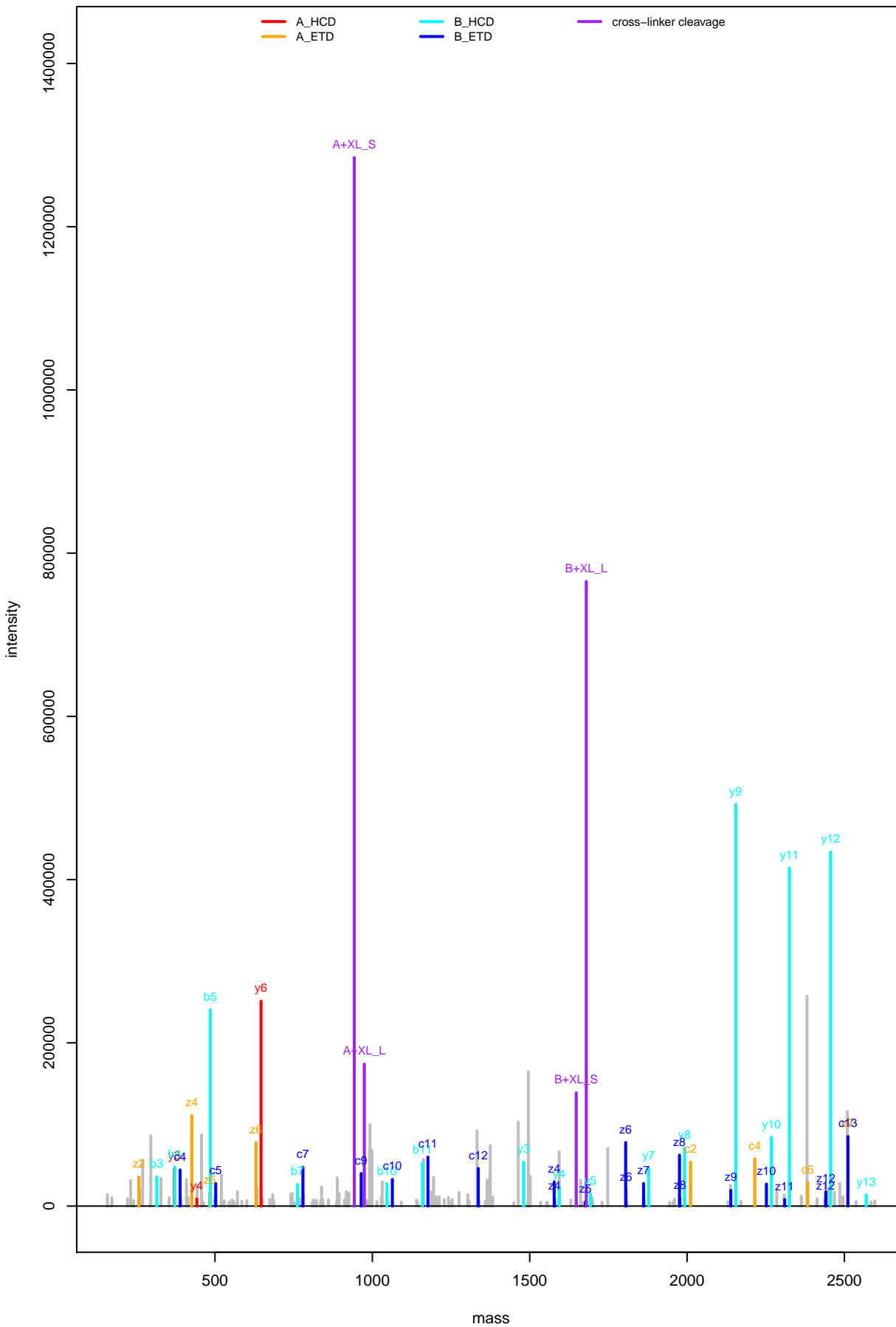

**NKFGAPQK+XL\_S**

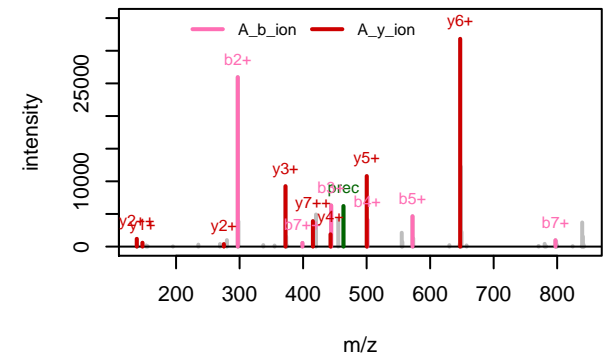

**NKFGAPQK+XL\_L**

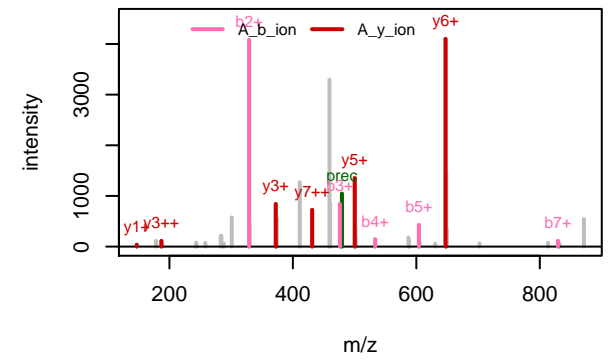

**ALMGLYNGQVLCKK+XL\_S**

**ALMGLYNGQVLCKK+XL\_L**

Supplement: Supplemental Data [file supp_RA117.000470_133922_0_supp_23978_fzffwf.zip › spectra_annotation/mito_DR_spectra_annotation/127-1-4-1-15-1.pdf]

ITTTKK  
GGKIGLFGGAGVGK

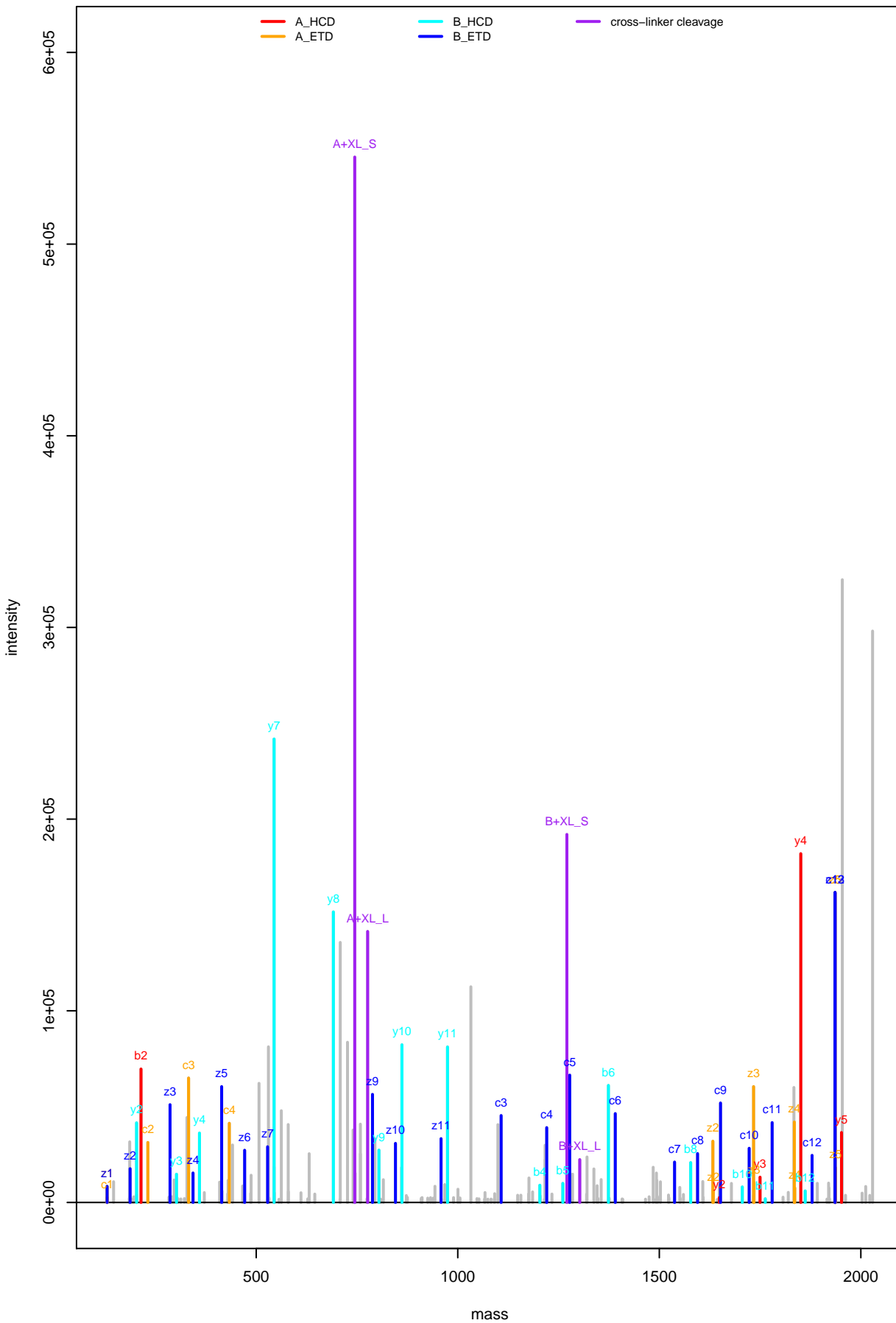

ITTTKK+XL\_S

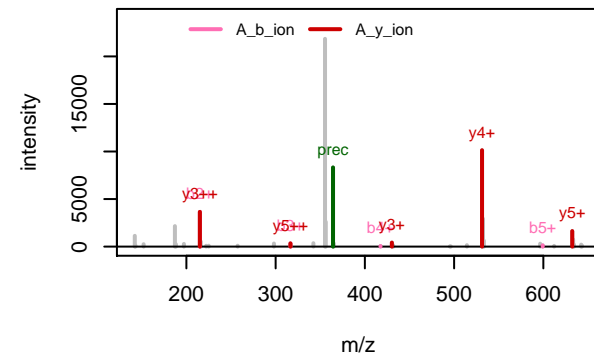

ITTTKK+XL\_L

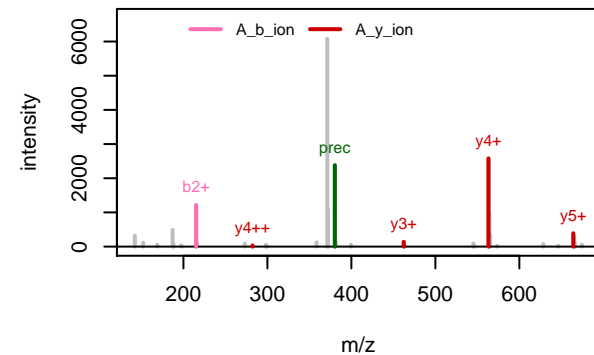

GGKIGLFGGAGVGK+XL\_S

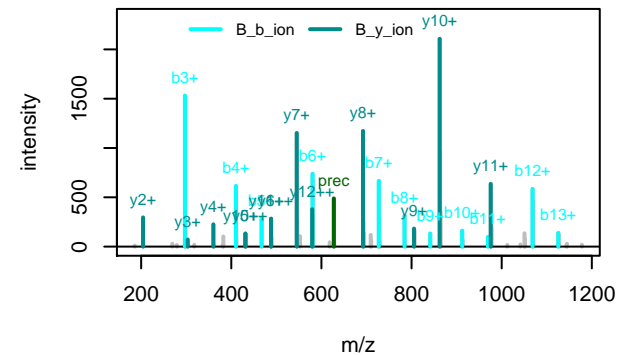

GGKIGLFGGAGVGK+XL\_L

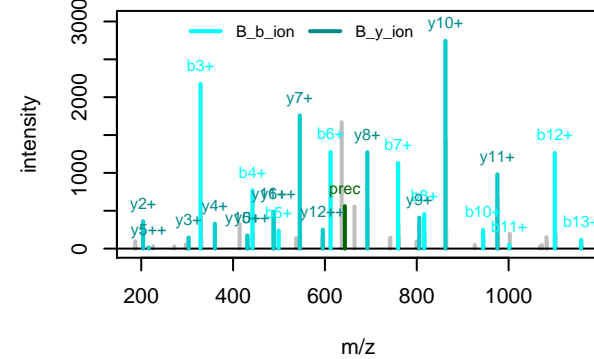

Supplement: Supplemental Data [file supp_RA117.000470_133922_0_supp_23978_fzffwf.zip › spectra_annotation/mito_DR_spectra_annotation/128-1-1-1-21-1.pdf]

**VSAKQSK  
LEPSKITK**

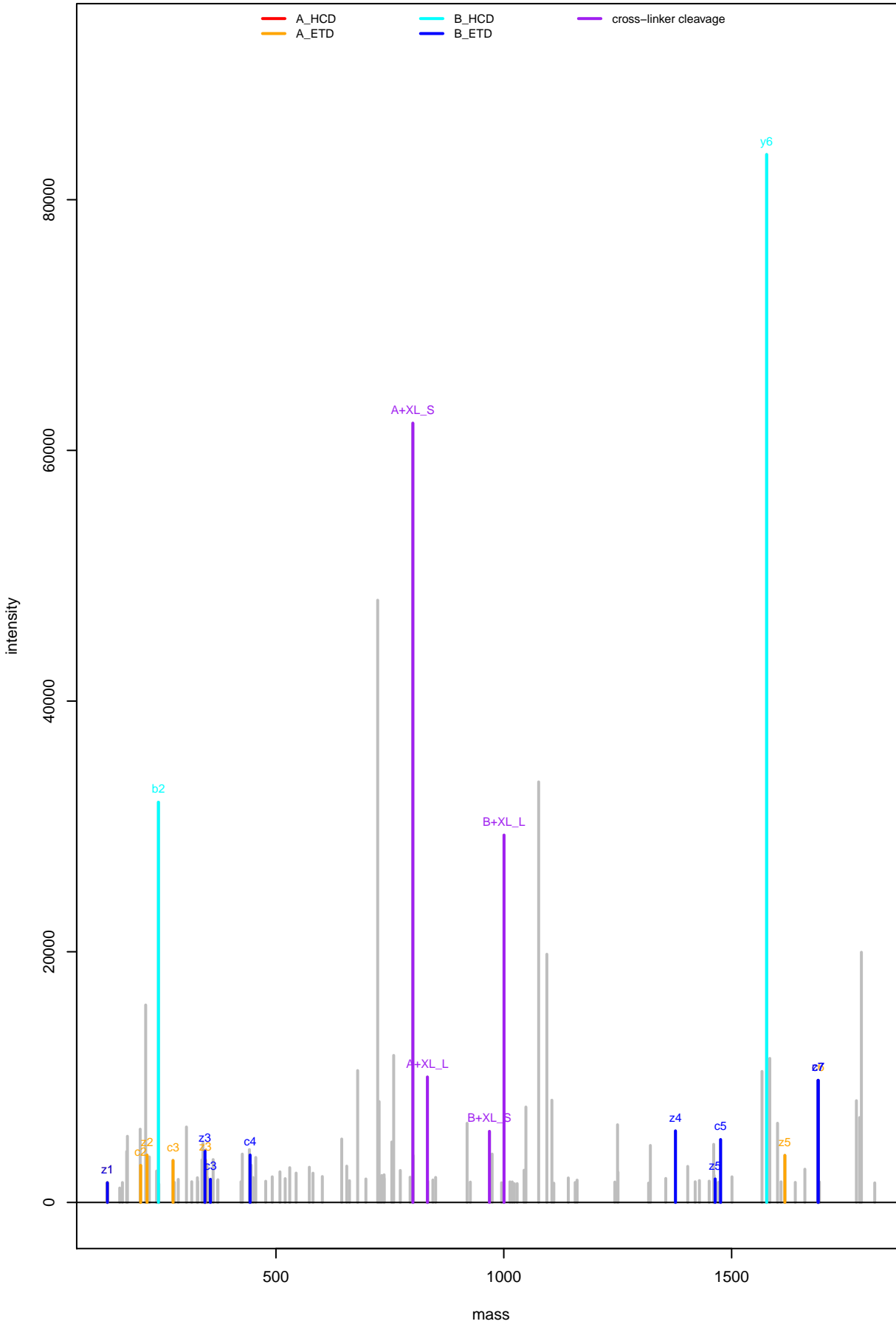

**VSAKQSK+XL\_S**

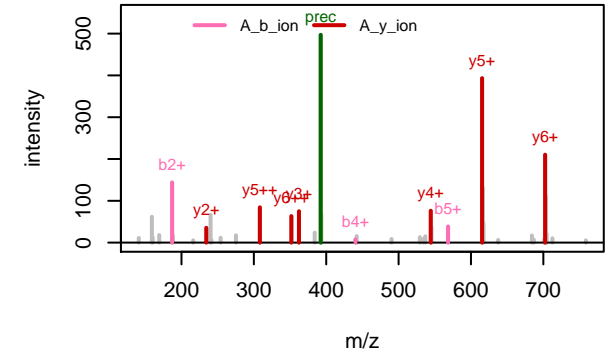

**VSAKQSK+XL\_L**

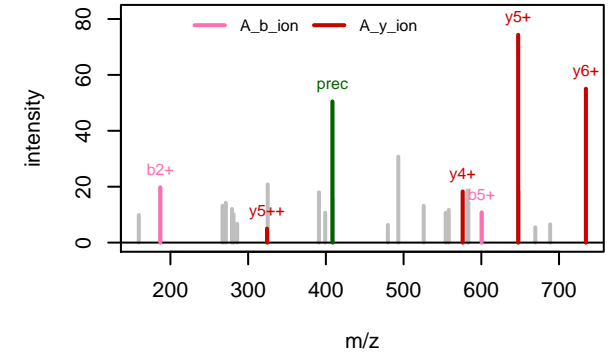

**LEPSKITK+XL\_S**

**LEPSKITK+XL\_L**

Supplement: Supplemental Data [file supp_RA117.000470_133922_0_supp_23978_fzffwf.zip › spectra_annotation/mito_DR_spectra_annotation/128-1-3-1-19-1.pdf]
