# Supplementary material for: The interactome of intact mitochondria by cross-linking mass spectrometry provides evidence for coexisting respiratory supercomplexes
Source: Mol Cell Proteomics. 2017 Dec 8;17(2):216–32. doi: 10.1074/mcp.RA117.000470 (PMC5795388; doi:10.1074/mcp.RA117.000470)
Supplement: Supplemental Data [file 102-1-10-1-4-1.pdf › spectra_annotation/mito_DR_spectra_annotation/102-1-10-1-4-1.pdf]

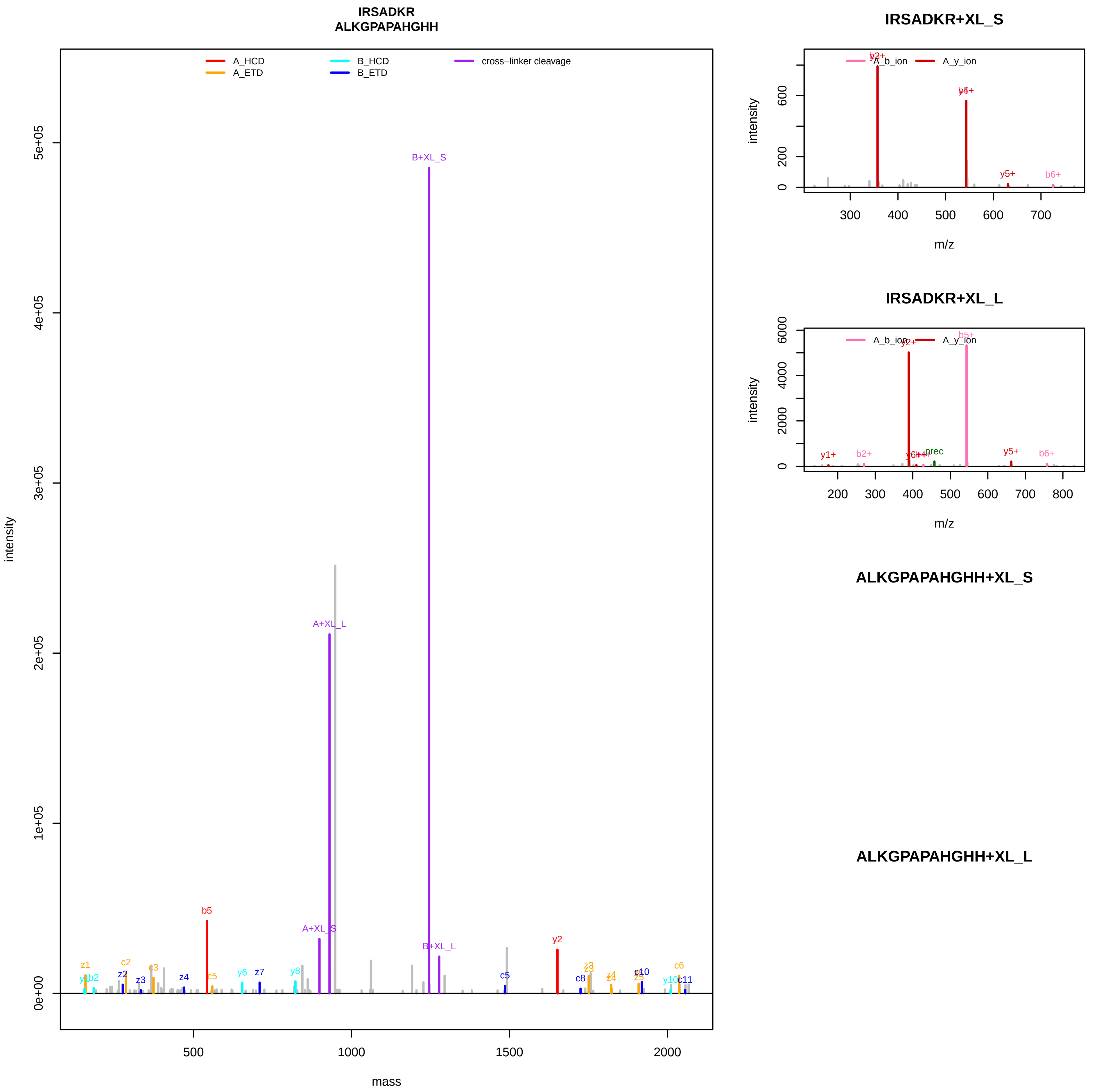
IRSADKR
ALKGPAPAHGHH
A_HCD
A_ETD
B_HCD
B_ETD
cross−linker cleavage
B+XL_S
A+XL_L
A+XL_S
B+XL_L
b5
y2
intensity
mass
IRSADKR+XL_S
A_b_ion
A_y_ion
y2+
y4+
y5+
b6+
m/z
IRSADKR+XL_L
b5+
y1+
b2+
prec
ALKGPAPAHGHH+XL_S
ALKGPAPAHGHH+XL_L
