# Supplementary material for: The interactome of intact mitochondria by cross-linking mass spectrometry provides evidence for coexisting respiratory supercomplexes
Source: Mol Cell Proteomics. 2017 Dec 8;17(2):216–32. doi: 10.1074/mcp.RA117.000470 (PMC5795388; doi:10.1074/mcp.RA117.000470)
Supplement: Supplemental Data [file 104-1-14-1-9-1.pdf › spectra_annotation/mito_DR_spectra_annotation/104-1-14-1-9-1.pdf]

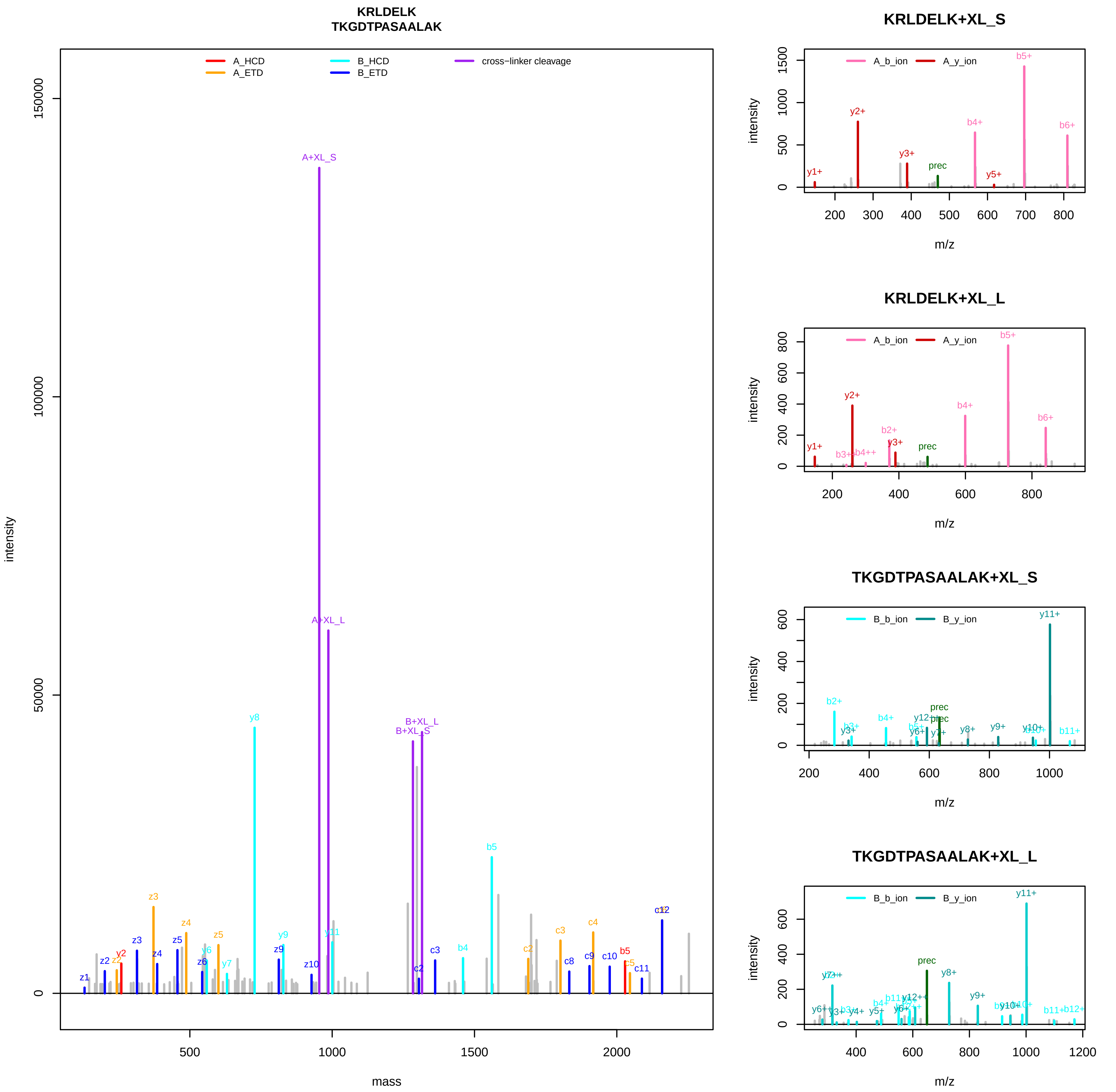

KRLDELK
TKGDTPASAALAK
A_HCD
A_ETD
B_HCD
B_ETD
cross−linker cleavage
intensity
mass
A+XL_S
A+XL_L
B+XL_L
B+XL_S
y8
b5
z1
z2
y2
z3
z4
z5
z6
y7
z9
y9
z10
y11
c2
c3
b4
c4
c8
c9
c10
b5
c5
c11
c12
KRLDELK+XL_S
KRLDELK+XL_L
TKGDTPASAALAK+XL_S
TKGDTPASAALAK+XL_L
A_b_ion
A_y_ion
B_b_ion
B_y_ion
prec
y11+
m/z
