# Supplementary material for: The interactome of intact mitochondria by cross-linking mass spectrometry provides evidence for coexisting respiratory supercomplexes
Source: Mol Cell Proteomics. 2017 Dec 8;17(2):216–32. doi: 10.1074/mcp.RA117.000470 (PMC5795388; doi:10.1074/mcp.RA117.000470)
Supplement: Supplemental Data [file 114-1-7-1-11-1.pdf › spectra_annotation/mito_DR_spectra_annotation/114-1-7-1-11-1.pdf]

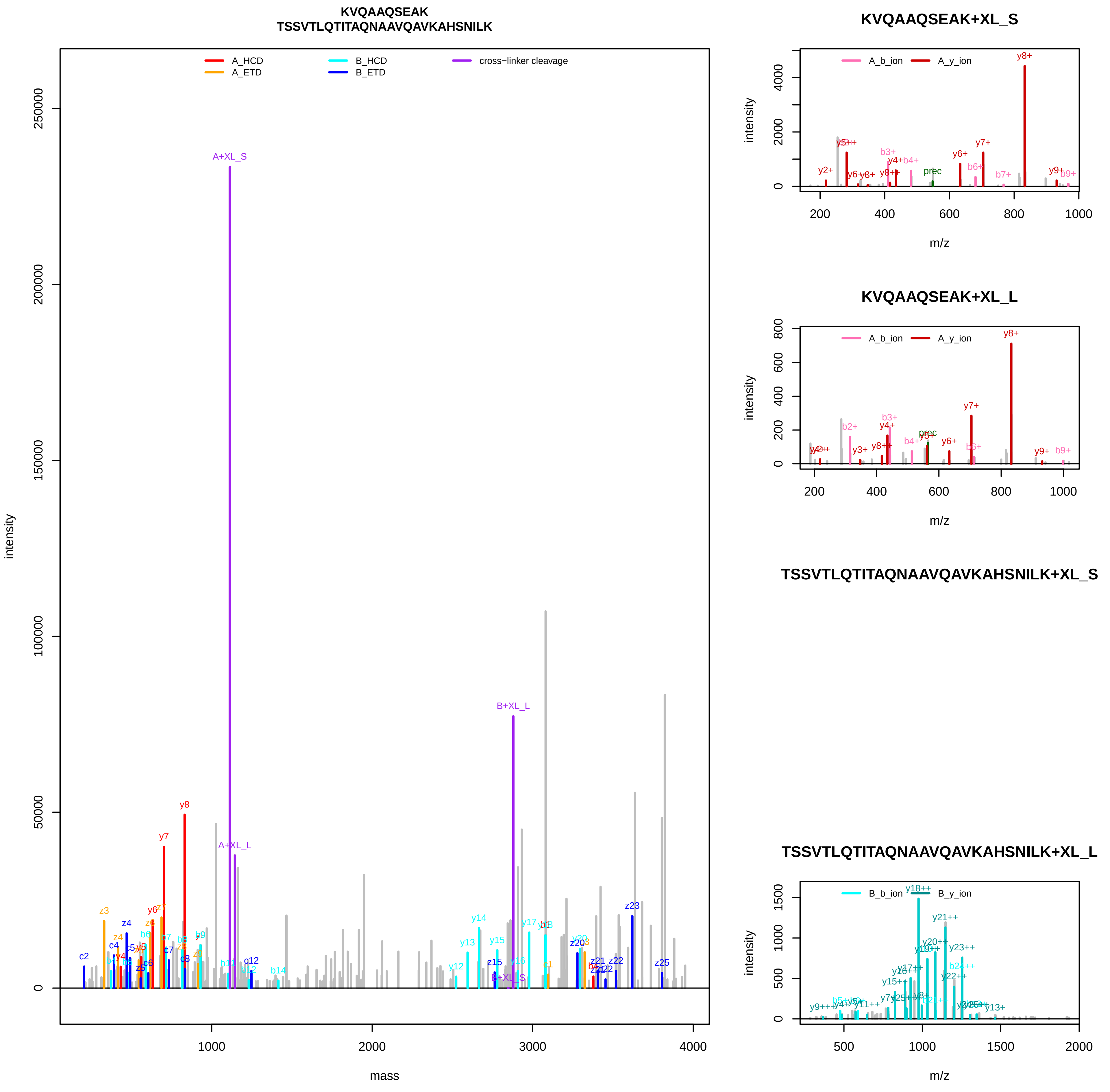
KVQAAQSEAK
TSSVTLQTITAQNAAVQAVKAHSNILK
A_HCD
A_ETD
B_HCD
B_ETD
cross−linker cleavage
A+XL_S
A+XL_L
B+XL_L
intensity
mass
0
50000
100000
150000
200000
250000
1000
2000
3000
4000
KVQAAQSEAK+XL_S
A_b_ion
A_y_ion
m/z
KVQAAQSEAK+XL_L
TSSVTLQTITAQNAAVQAVKAHSNILK+XL_S
TSSVTLQTITAQNAAVQAVKAHSNILK+XL_L
B_b_ion
B_y_ion
