# Supplementary material for: The interactome of intact mitochondria by cross-linking mass spectrometry provides evidence for coexisting respiratory supercomplexes
Source: Mol Cell Proteomics. 2017 Dec 8;17(2):216–32. doi: 10.1074/mcp.RA117.000470 (PMC5795388; doi:10.1074/mcp.RA117.000470)
Supplement: Supplemental Data [file 12-1-1-1-7-1.pdf › spectra_annotation/mito_DR_spectra_annotation/12-1-1-1-7-1.pdf]

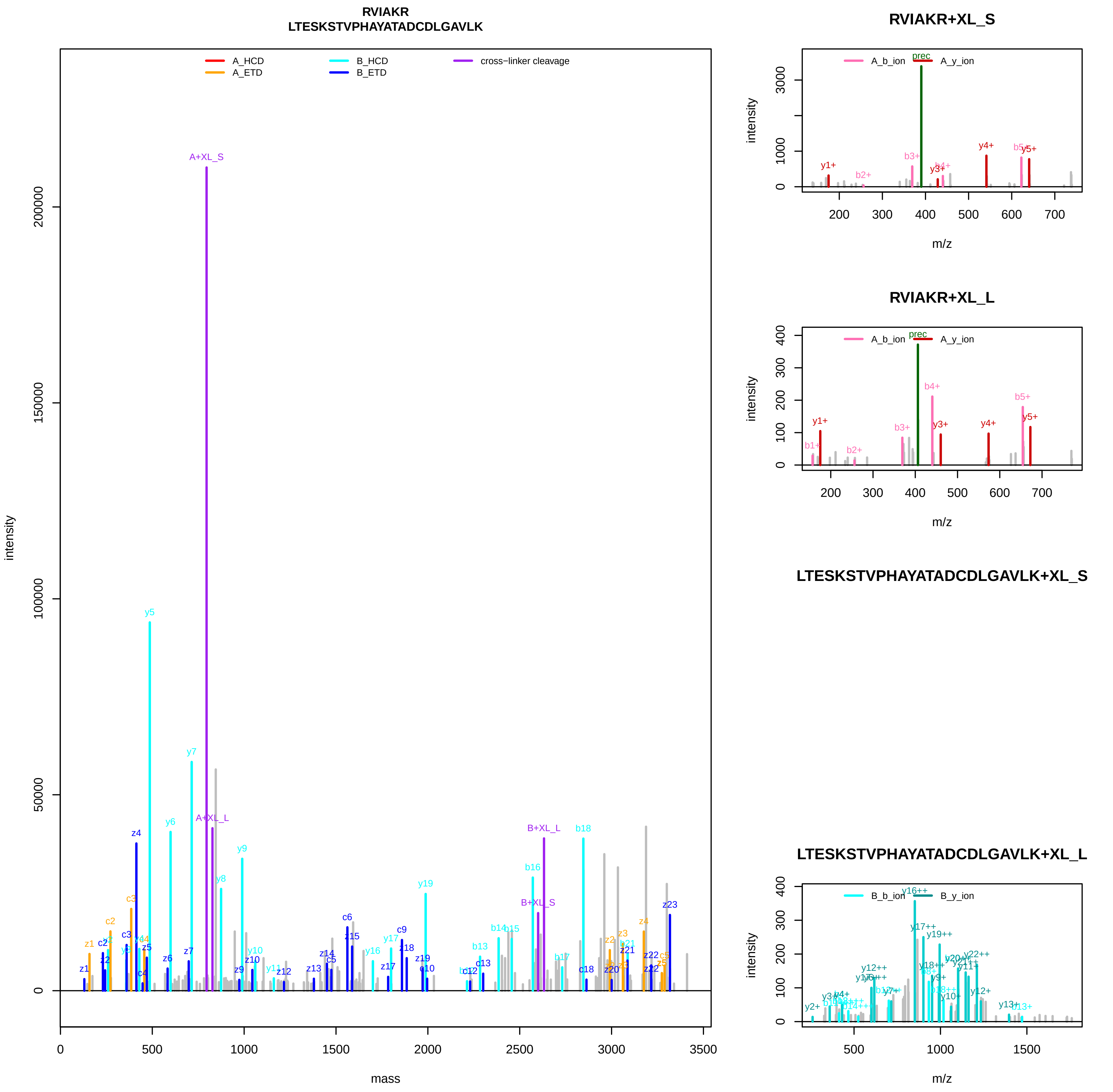

RVIAKR
LTESKSTVPHAYATADCDLGAVLK
A_HCD
A_ETD
B_HCD
B_ETD
cross−linker cleavage
intensity
mass
0
50000
100000
150000
200000
0 500 1000 1500 2000 2500 3000 3500
A+XL_S
A+XL_L
B+XL_L
B+XL_S
y5
y7
b18
RVIAKR+XL_S
A_b_ion
A_y_ion
prec
intensity
m/z
0 1000 3000
200 300 400 500 600 700
RVIAKR+XL_L
A_b_ion
A_y_ion
prec
intensity
m/z
0 100 200 300 400
200 300 400 500 600 700
LTESKSTVPHAYATADCDLGAVLK+XL_S
LTESKSTVPHAYATADCDLGAVLK+XL_L
B_b_ion
B_y_ion
intensity
m/z
0 100 200 300 400
500 1000 1500
