# Supplementary material for: The interactome of intact mitochondria by cross-linking mass spectrometry provides evidence for coexisting respiratory supercomplexes
Source: Mol Cell Proteomics. 2017 Dec 8;17(2):216–32. doi: 10.1074/mcp.RA117.000470 (PMC5795388; doi:10.1074/mcp.RA117.000470)
Supplement: Supplemental Data [file 126-1-13-1-17-1.pdf › spectra_annotation/mito_DR_spectra_annotation/126-1-13-1-17-1.pdf]

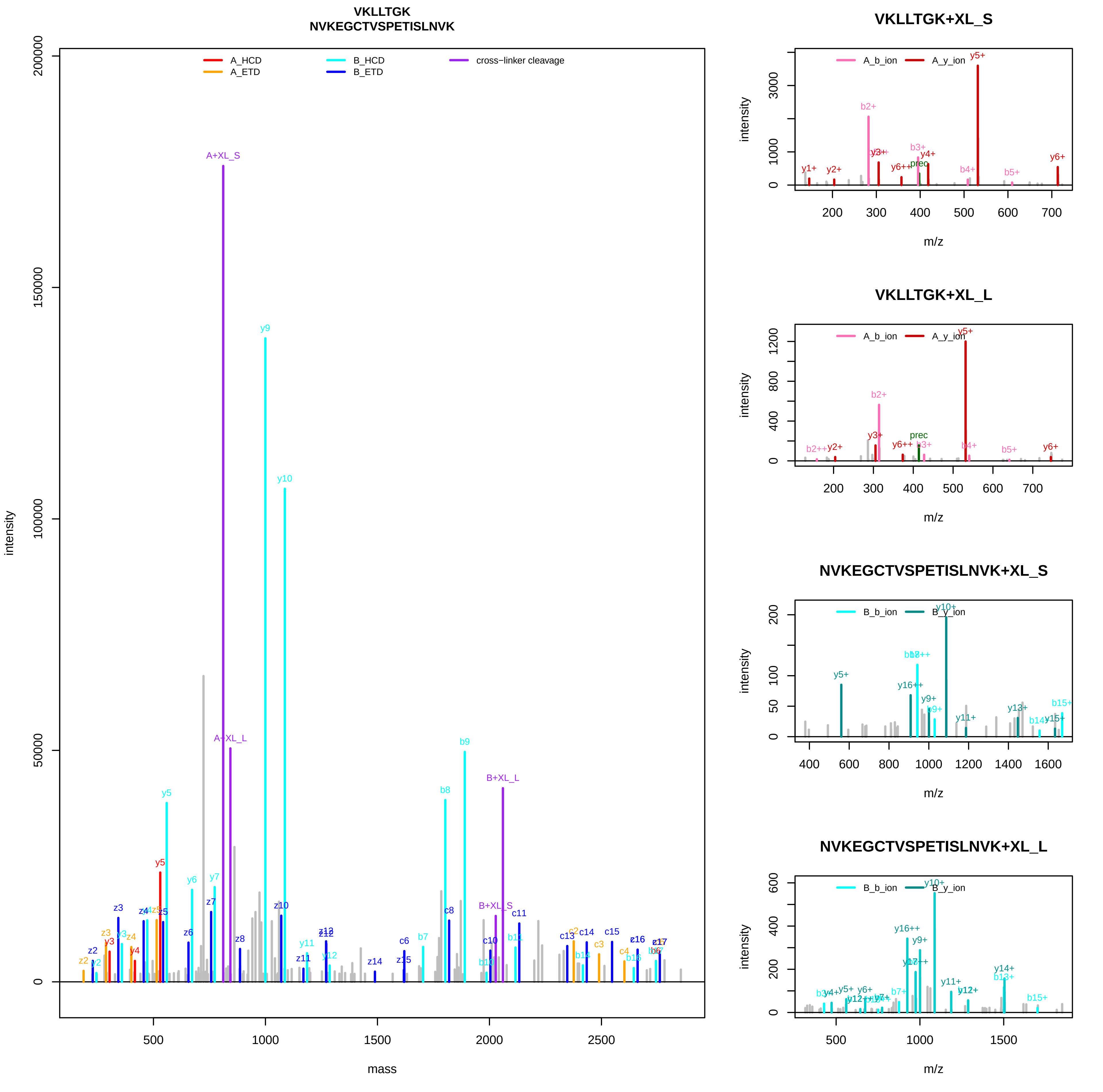
VKLLTGK
NVKEGCTVSPETISLNVK
A_HCD
A_ETD
B_HCD
B_ETD
cross−linker cleavage
A+XL_S
A+XL_L
B+XL_S
B+XL_L
y9
y10
b8
b9
y5
y6
y7
intensity
mass
0
50000
100000
150000
200000
500
1000
1500
2000
2500
VKLLTGK+XL_S
A_b_ion
A_y_ion
b2+
y5+
y6+
prec
m/z
VKLLTGK+XL_L
NVKEGCTVSPETISLNVK+XL_S
B_b_ion
B_y_ion
y10+
NVKEGCTVSPETISLNVK+XL_L
